# Supplementary material for: Enhancing inbreeding estimation and global conservation insights through chromosome-level assemblies of the Chinese and Malayan pangolin
Source: Gigascience. 2025 Feb 14;14:giaf003. doi: 10.1093/gigascience/giaf003 (PMC11825179; doi:10.1093/gigascience/giaf003)
Supplement: giaf003_Supplemental_File [file giaf003_supplemental_file.docx]

**Supplemental Information**

**Enhancing inbreeding estimation and global conservation insights through chromosome-level assemblies of the Chinese and Malayan pangolin**

Tianming Lan^1,2,3,†^, Yinping Tian^3,†^, Minhui Shi^4,5†^, Boyang Liu^3^, Yu Lin^3^, Yanling Xia^3^, Yue Ma^3^, Sunil Kumar Sahu ^4^, Qing Wang^3^, Jun Li^2^, Jin Chen^3^, Fanghui Hou^6,7^, Chuanling Yin^3^, Kai Wang^2^, Yuan Fu^3^, Tengcheng Que^8,9^, Wenjian Liu^8^, Huan Liu^3^, Haimeng Li^3,10,*^ and Yan Hua^2,*^

^1^ BGI Life Science Joint Research Center, Northeast Forestry University, Harbin 150040, China

^2^ Guangdong Provincial Key Laboratory of Silviculture, Protection and Utilization, Guangdong Academy of Forestry, Guangzhou 510520, China

^3^ College of Wildlife and Protected Area, Northeast Forestry University, Harbin 150040, China

^4^ BGI Research, Wuhan 430074, China

^5^ State Key Laboratory of Agricultural Genomics, BGI-Shenzhen, Shenzhen 518083, China

^6^ Guangdong Wildlife Rescue Monitoring Center, Guangzhou 510520, China

^7^ Pangolin Conservation Research Center of National Forestry and Grassland Administration, Guangzhou 510520, China

^8^ Faculty of Data Science City University of Macau, Macau 999078, China

^9^ Guangxi Zhuang Autonomous Terrestrial Wildlife Rescue Research and Epidemic Diseases Monitoring Center, Nanning 530025, China

^10^ Heilongjiang Key Laboratory of Complex Traits and Protein Machines in Organisms, Harbin 150040, China

∗Correspondence address. Yan Hua, Guangdong Provincial Key Laboratory of Silviculture, Protection and Utilization, Guangdong Academy of Forestry, Guangzhou 510520 China. E-mail: [wildlife530@hotmail.com](mailto:wildlife530@hotmail.com); Haimeng Li, College of Wildlife and Protected Area, Northeast Forestry University, Harbin 150040, China. E-mail: lihaimeng66@163.com

†Equal contributions.

**Supplementary Results**

**The evaluation of haplotype-resolved pangolin genomes**

Merqury k-mer analysis indicated high completeness and a low level of artificial duplication for haplotype-resolved chromosome-level genomes of both MJ and MP (Supplementary Fig. S14). The k-mer spectra plot showed that homozygous regions (shared k-mers) and heterozygous regions (haploid specific k-mers) mainly consisted of two-copies (~82X/78X) and one-copy (~ 40X/38X) k-mers (Supplementary Fig. S14). Further, the sequencing depths of the two groups of haplotigs were consistent for both genomes based on the evaluation by both PacBio HiFi reads and DNBSEQ reads (Supplementary Fig. S15), implying the completeness of haplotype-resolved assemblies. As expected, the k-mer completeness for all haploid and diploid genomes attained a very high level (Supplementary Table S17).

The base-level quality evaluation showed that all genomes had high assembly accuracy with the lowest QV score of 55.08, corresponding to an assembly error rate of 3.10e-06 (Supplementary Table S17). Besides, all these assemblies showed high BUSCO scores, with 97.5% and 98.0% BUSCO genes identified in the MJ and MP diploid genomes, respectively. For haploid genomes, the BUSCO score was still as high as 94.8 % even for the lowest MPH1 (Supplementary Table S7). The genome mapping of DNBSEQ short reads, Hi-C short reads, and RNA sequencing data to genome assemblies also presented high mapping rates, especially for DNBSEQ short reads, with the mapping rate of 99.91% and 99.93% for MJ and MP genome, respectively (Supplementary Table S18). Finally, we assessed the structural-level accuracy by mapping long PacBio HiFi reads to the haploid genomes and found that more than 99.24%/99.27% and 98.61%/97.38% of MJ/MP genomes were identified to be correctly assembled with a criterion of $\geq$10 X long reads being mapped at a single position[1].

Sixteen pangolin genomes representing all eight species have been reported to date, including three genomes assembled from ONT long reads (one Chinese pangolin genome[2], one Malayan pangolin genome[2], and one giant pangolin genome[3]) and 13 short-read assembled genomes[4-7]. For the Chinese pangolin, two short-read generated genomes [6, 7] and one ONT long-read assembled genome have been reported[2]. The Chinese pangolin genome assembled in this study presented a contig N50 of 2709.24-fold, 2699.34-fold, 419.42-fold, and 4.02-fold longer than that of the M_pentadactyla-1.1.1_HiC, M_pentadactyla-1.1.1, YNU_ManPten_2.0, and ASM2424420v1 genomes, respectively (Supplementary Table S19). There are also three reported genome assemblies for the Malayan pangolin, including two short-read generated genomes [6, 7] and one ONT long-read assembled genome[2]. The contiguity of the Malayan pangolin genome in this study was even higher than the Chinese pangolin genome we assembled, which is 2827.13-fold, 2822.98-fold, 62.28-fold and 2.92-fold greater than the contig N50 of ManJav1.0_HiC, ManJav1.0, YNU_ManJav_2.0 and ASM2460508v1 genomes, respectively (Supplementary Table S19). Another advantage of our assemblies in this study is the partitioning of the diploid chromosomes into haploid chromosomes, which allowed us to detect genetic differences between haplotigs and provide a better understanding of allele-specific functions[8, 9], which cannot be achieved well with a hybrid reference genome. These advancements are anticipated to be further developed and enhance the precise conservation efforts for pangolins.

**Comparisons of the genomic landscape between haploid chromosomes**

In general, the two haploid genomes of MJ or MP were found to be very similar. The sequence differences (100bp window) showed one peak in the histogram for both MJ and MP genomes, indicating that the proportion of identical sequences between haploid genomes were dominant (Supplementary Table S20 Supplementary Fig. S16). Synteny analysis showed clear one-to-one syntenic blocks between homologous haploid chromosome pairs of the two pangolin species (Supplementary Fig. S17), further showing the high similarity between haploid genomes, which was also reflected in the k-mer analysis with almost all k-mers were shared between haploid genomes (Supplementary Fig. S14).

Nonetheless, we detected many structural variants (SVs) (>50bp) between MJH1 and MJH2, and between MPH1 and MPH2. In general, we found 5,223 (3,673 duplications, 533 translocations, 509 inversions and 508 deletions) and 8,916 (7,718 duplications, 755 translocations, 254 inversions and 189 deletions) SVs between the haploid genomes of MJ and MP (Supplementary Table S21). All these SVs were validated by our assembled contigs (Supplementary Fig. S18). We found 2,136 genes that were distributed in the SVs of the MJ genome, while 4,264 such genes were found in the MP genome (Supplementary Fig. S19, Supplementary Table S22-S25). It is worth noting that four genes were disrupted by breakpoints of SVs and had become pseudogenic in one of the two haploid chromosomes for the MJ, and we further found 20 such genes in the MP genome. Interestingly, several of these genes in the MP genome were vision-related, such as *PXDN*, *NBAS* and *RTN3*. The *PXDN* gene is closely related to eye development, and the loss of *PXDN* gene in mice results in severe eye disorders, including drastically disorganized eye structures and the absence of eyeballs[10]. The *NBAS* gene is estimated to correlate with retinal homeostasis[11]. The *RTN3* gene is likely to play an important role in retinal function, as this gene’s mutation causes retinal dystrophies[12]. Some of the other genes were found to be related to immunity and metabolism (Supplementary Table S25).

**Supplemental Figures**


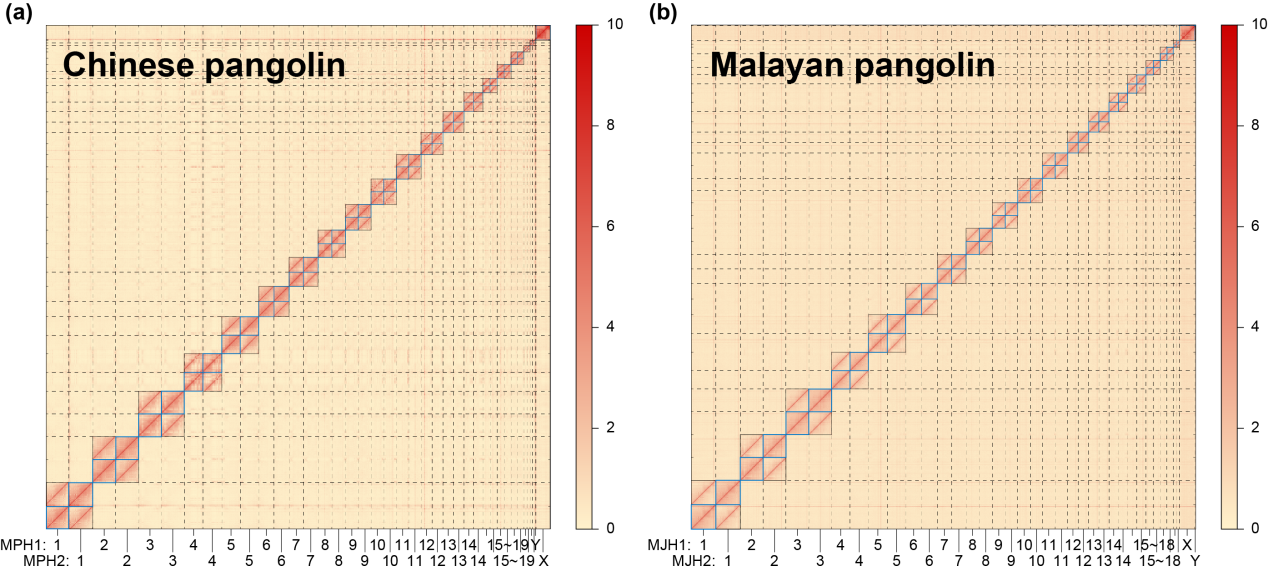


**Figure S1.** The heatmap represents the contact matrices generated by aligning the Hi-C data to the haploid chromosome-level Chinese pangolin (a) and Malayan pangolin (b) genomes.

**
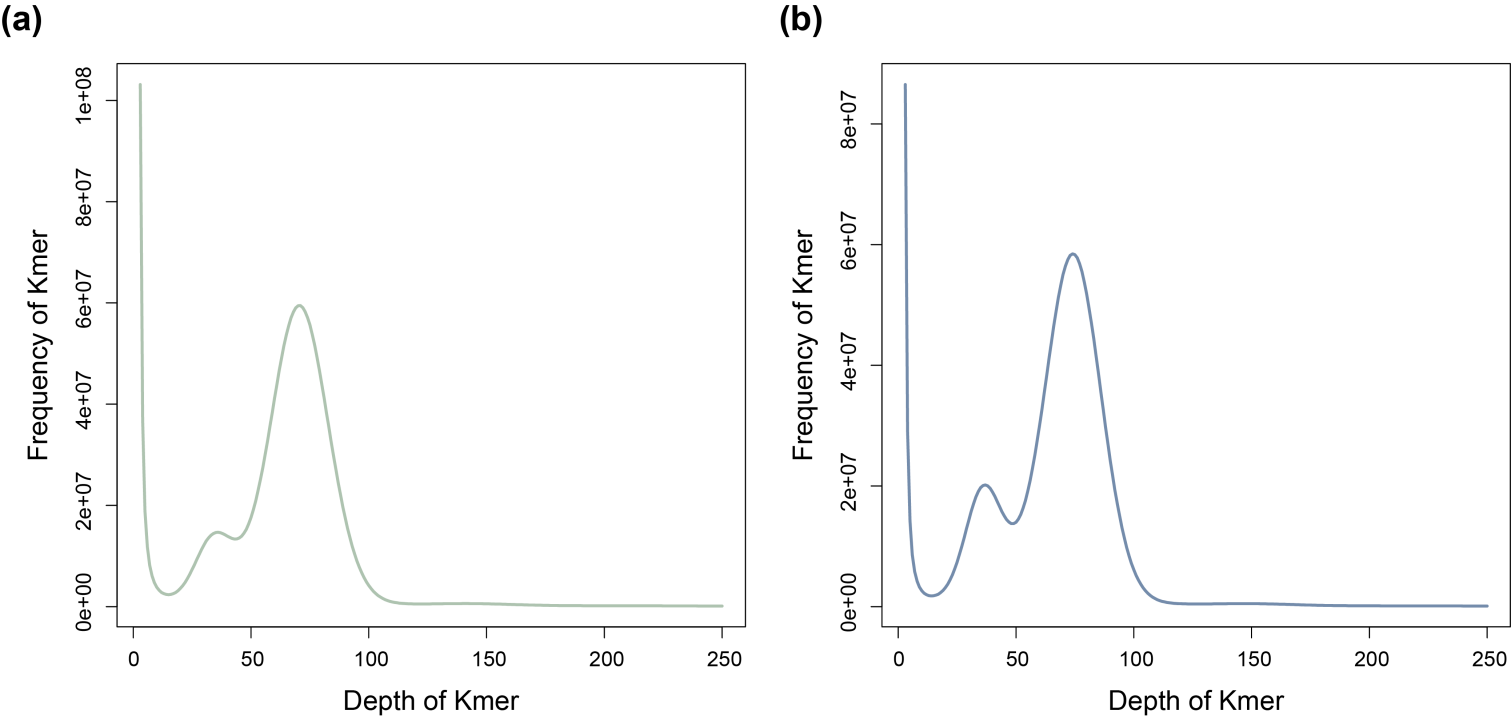
**

**Figure S2.** Estimated genome size of the Chinese and Malayan pangolin genomes by using K-mer frequency analysis with k-mer size of 17. (a) The K-mer spectra of the Chinese pangolin genome. (b) The K-mer spectra of the Malayan pangolin genome.


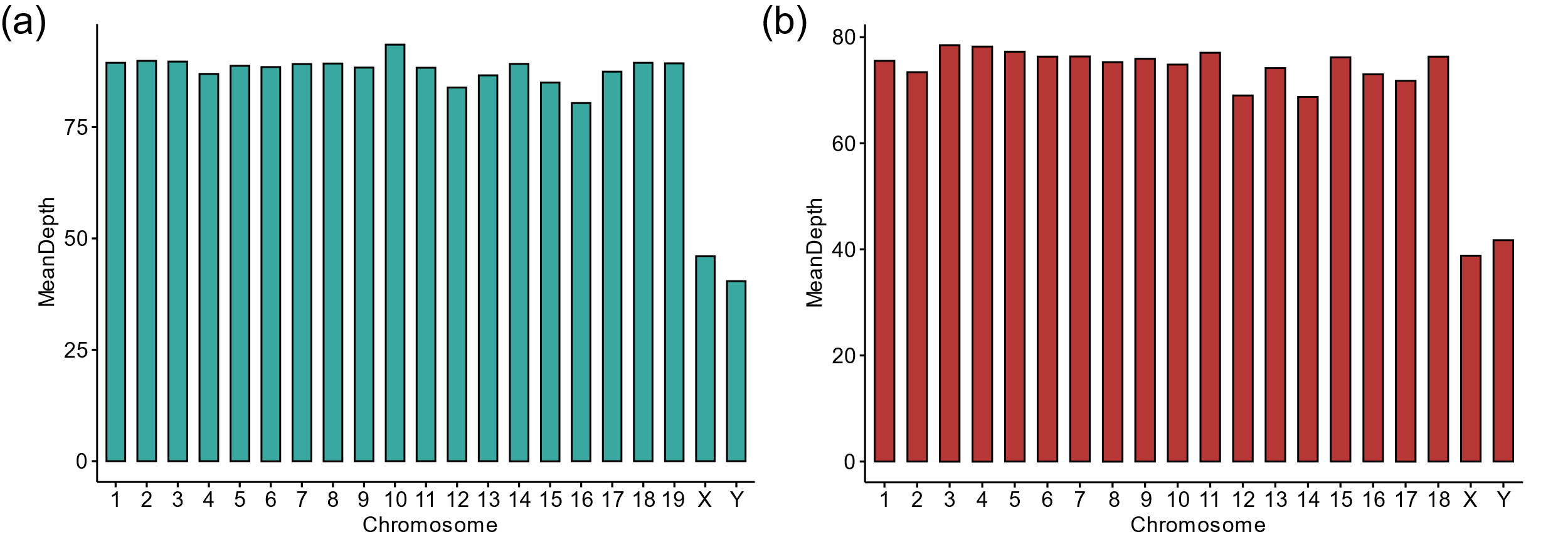


**Figure S3.** Sequencing depths of each pseudo-chromosome. (a) Sequencing depths of the 19 autosomes, X chromosome, and Y chromosome in the Chinese pangolin genome. (b) Sequencing depths of the 18 autosomes, X chromosome, and Y chromosome in the Malayan pangolin genome.

**
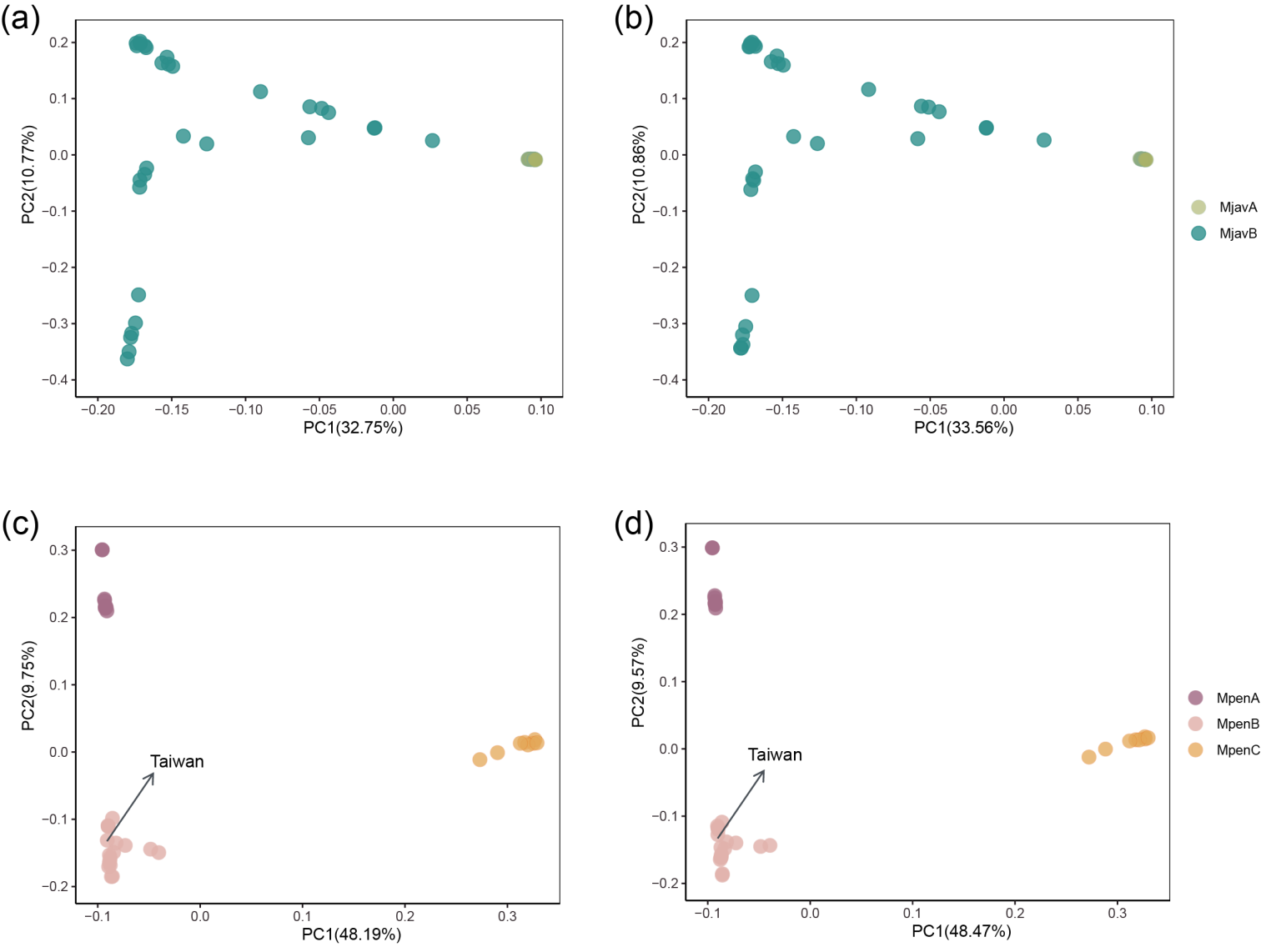
**

**Figure S4.** Comparisons of LG and SG on PCA analysis for both Chinese and Malayan pangolin populations. (a) PCA analysis of Malayan pangolin populations based on SG. (b) PCA analysis of Malayan pangolin populations based on LG. (c) PCA analysis of Chinese pangolin populations based on SG. (d) PCA analysis of Chinese pangolin populations based on LG.

**
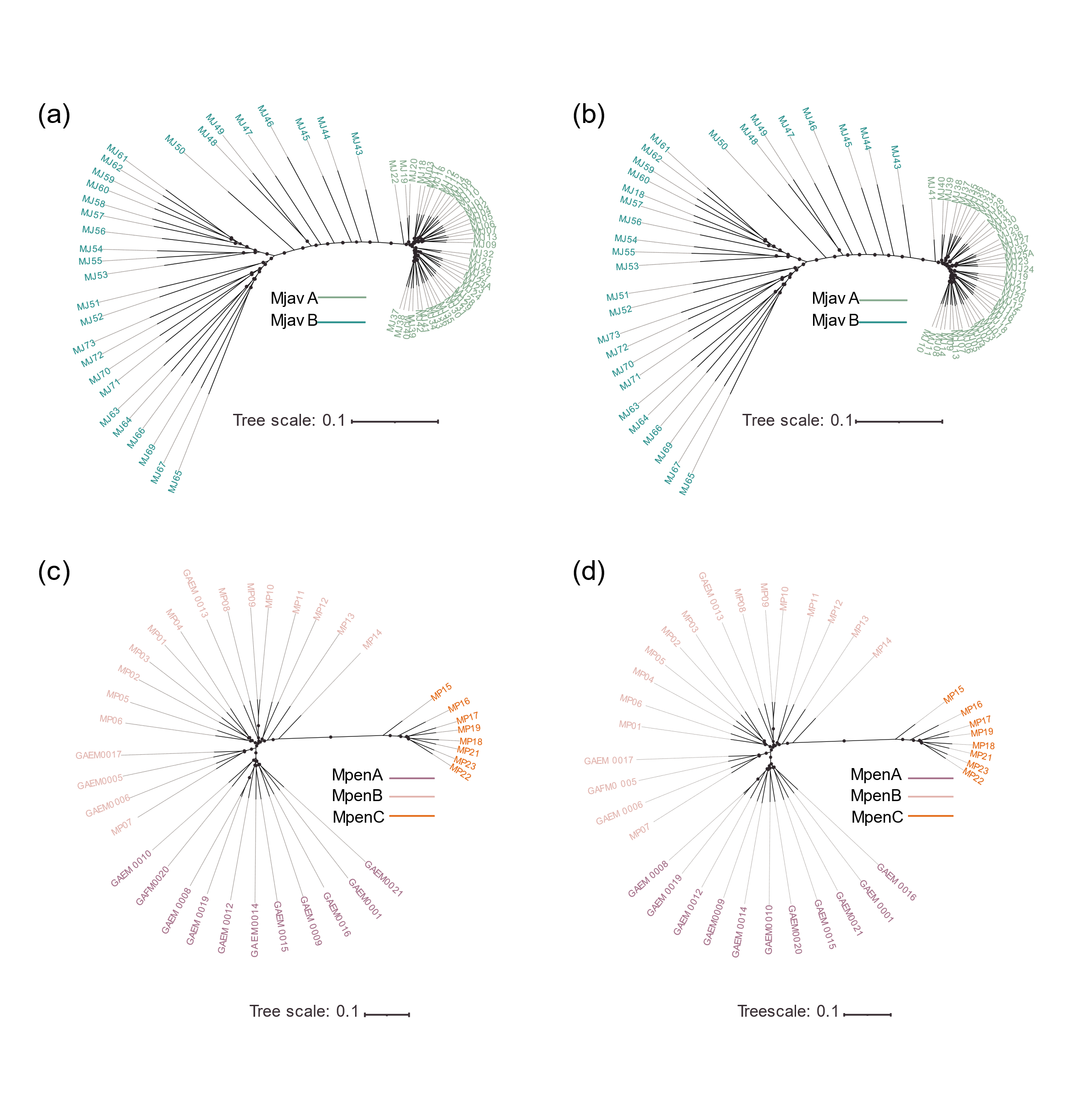
**

**Figure S5.** Comparisons of LG and SG on the construction of phylogenetic tree for both Chinese and Malayan pangolin populations. (a) Phylogenetic tree constructed based on the SG for Malayan pangolin populations. (b) Phylogenetic tree constructed based on the LG for Malayan pangolin populations. (c) Phylogenetic tree constructed based on the SG for Chinese pangolin populations. (d) Phylogenetic tree constructed based on the LG for Chinese pangolin populations.

**
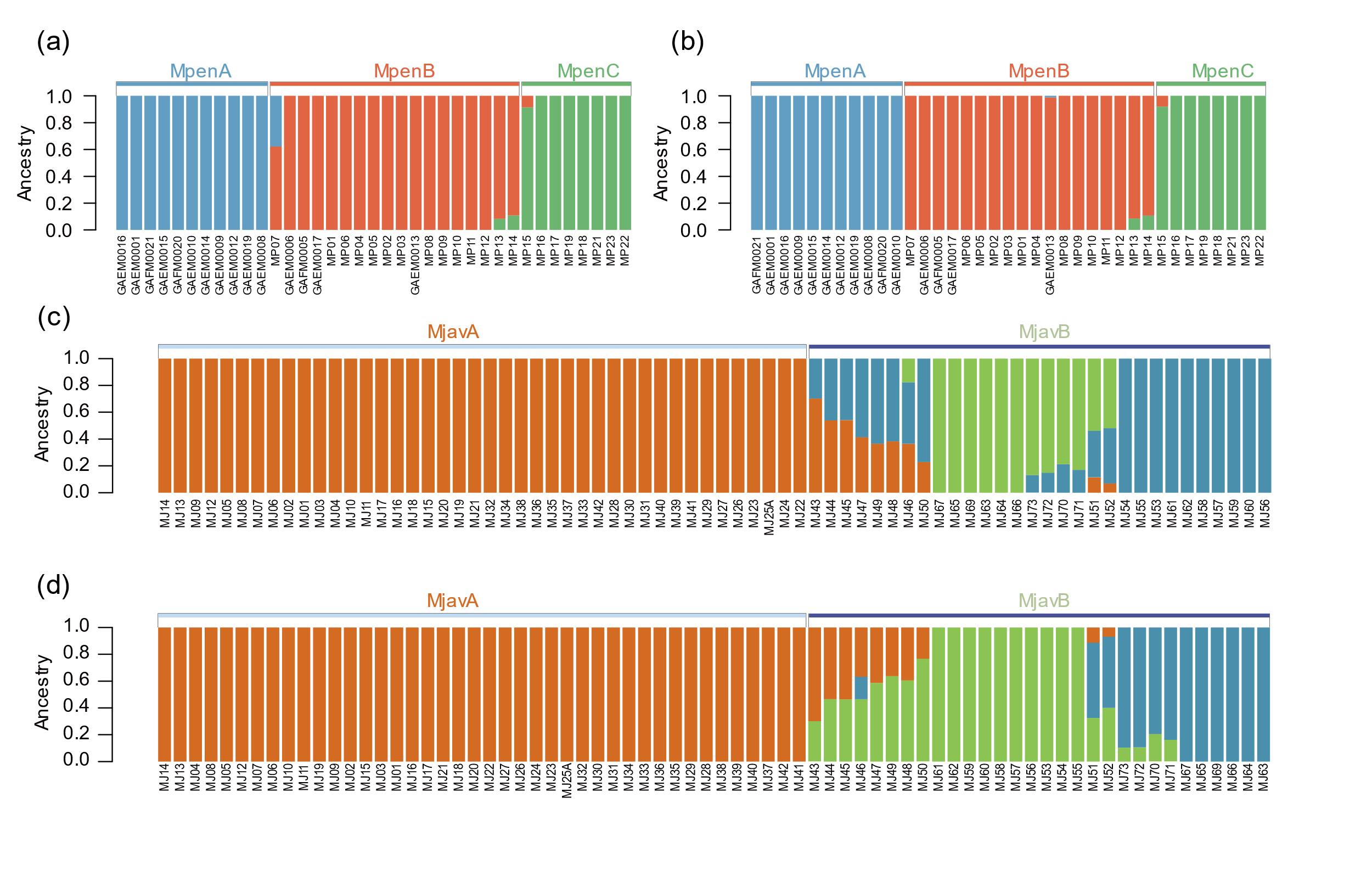
**

**Figure S6.** Comparisons of LG and SG on the admixture analysis for both Chinese and Malayan pangolin populations. (a) Genome-wide admixture analysis for three populations of Chinese pangolin based on the SG. (b) Genome-wide admixture analysis for three populations of Chinese pangolin based on the LG. (c) Genome-wide admixture analysis for two populations of Malayan pangolin based on the SG. (d) Genome-wide admixture analysis for two populations of Malayan pangolin based on the LG.

**
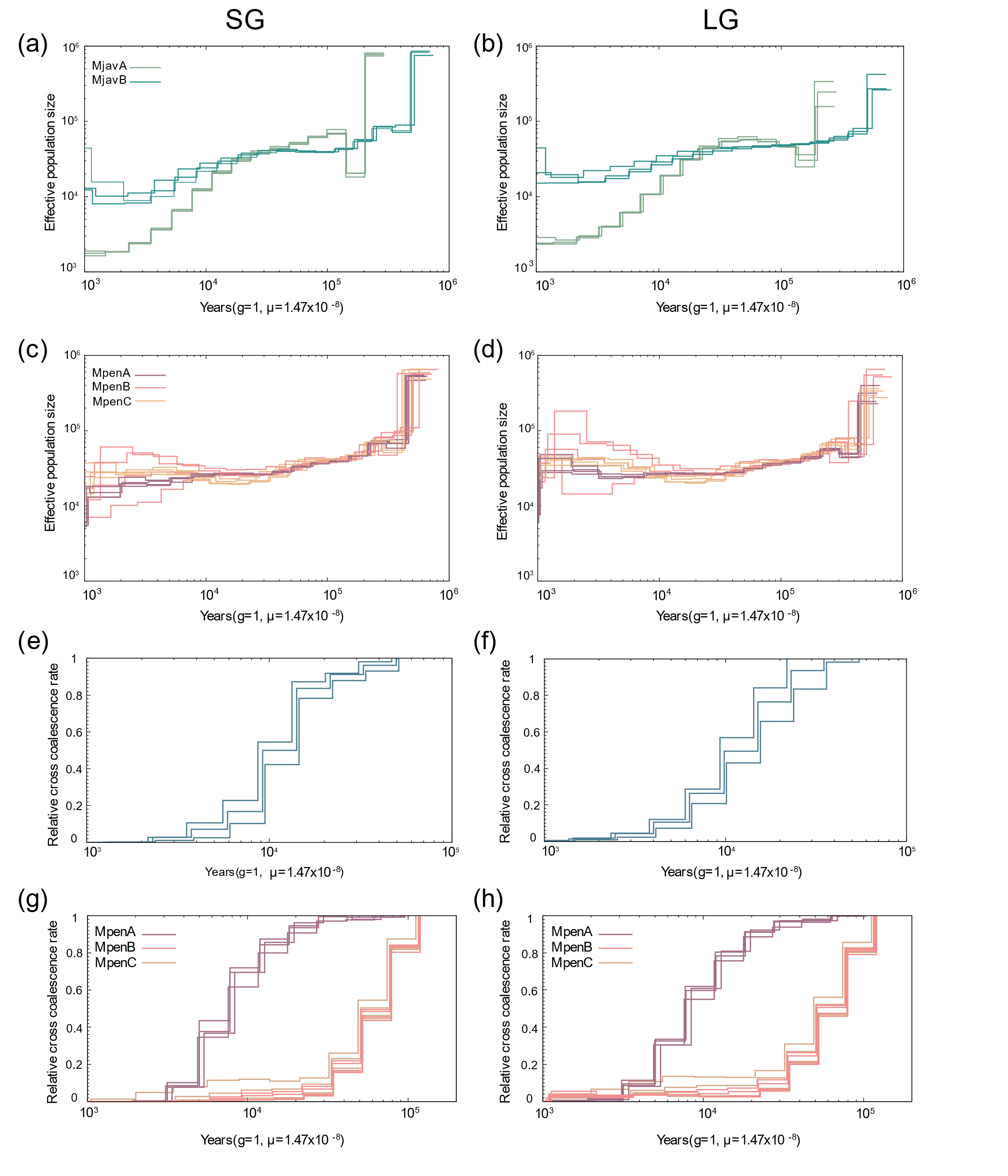
**

**Figure S7. Comparison of LG and SG assembly for analyzing population history and separation in Chinese and Malayan pangolin populations**. (a) The dynamics of effective population size of Malayan pangolin populations analyzed based on the SG. (b) The population size dynamics of Malayan pangolin populations analyzed based on the LG. (c) The population size dynamics of Chinese pangolin populations analyzed based on the SG. (d) The population size dynamics of Chinese pangolin populations analyzed based on the LG. (e) The divergence time between two populations of Malayan pangolin estimated based on the SG. (f) The divergence time between two populations of Malayan pangolin estimated based on the LG. (g) The divergence time among three populations of Chinese pangolin estimated based on the SG. (h) The divergence time among three populations of Chinese pangolin estimated based on the LG.

**
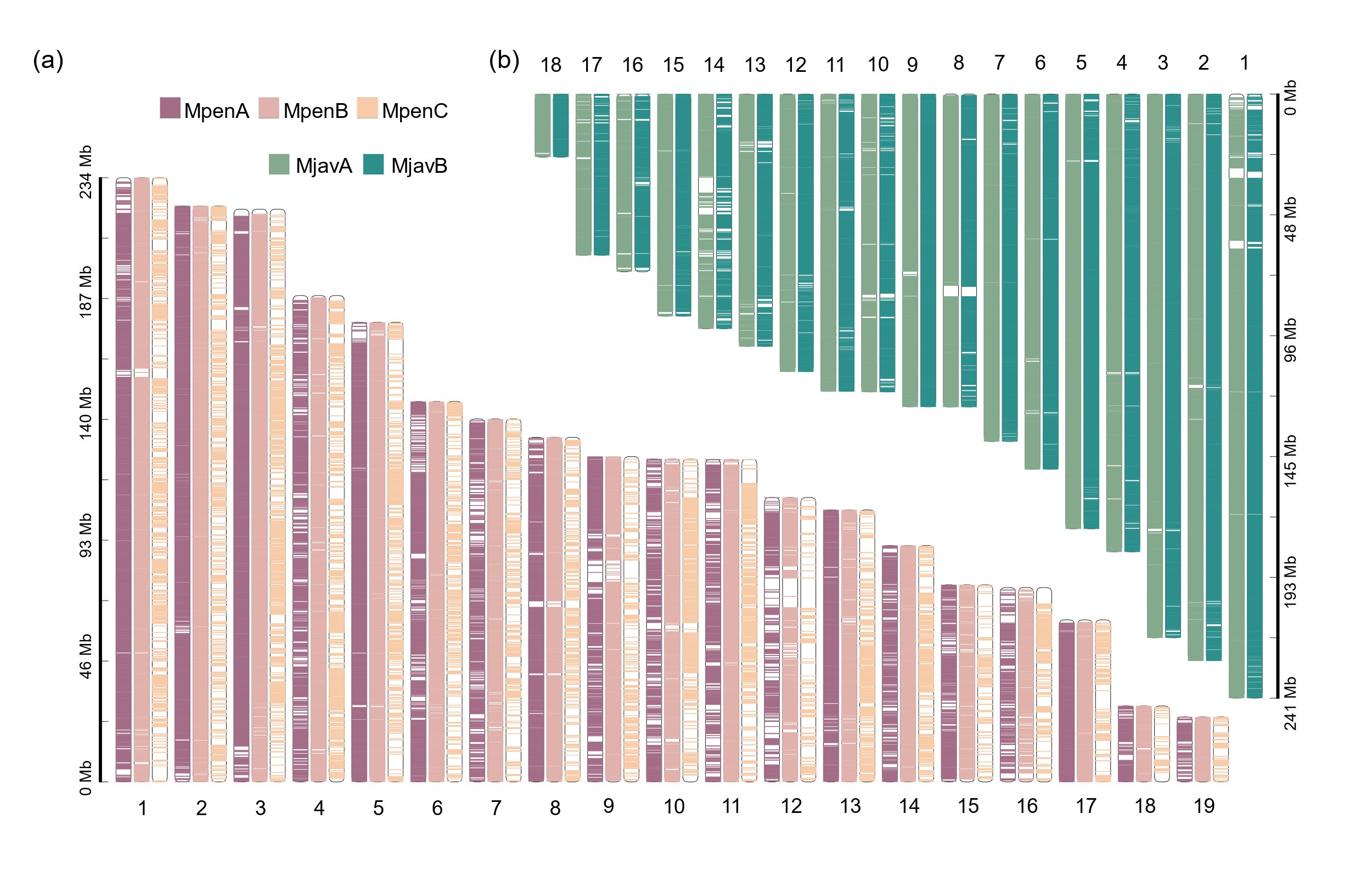
**

**Figure S8.** (a) The population-level ROH distribution in three Chinese pangolin populations. (b) The population-level ROH distribution in two Malayan pangolin populations.


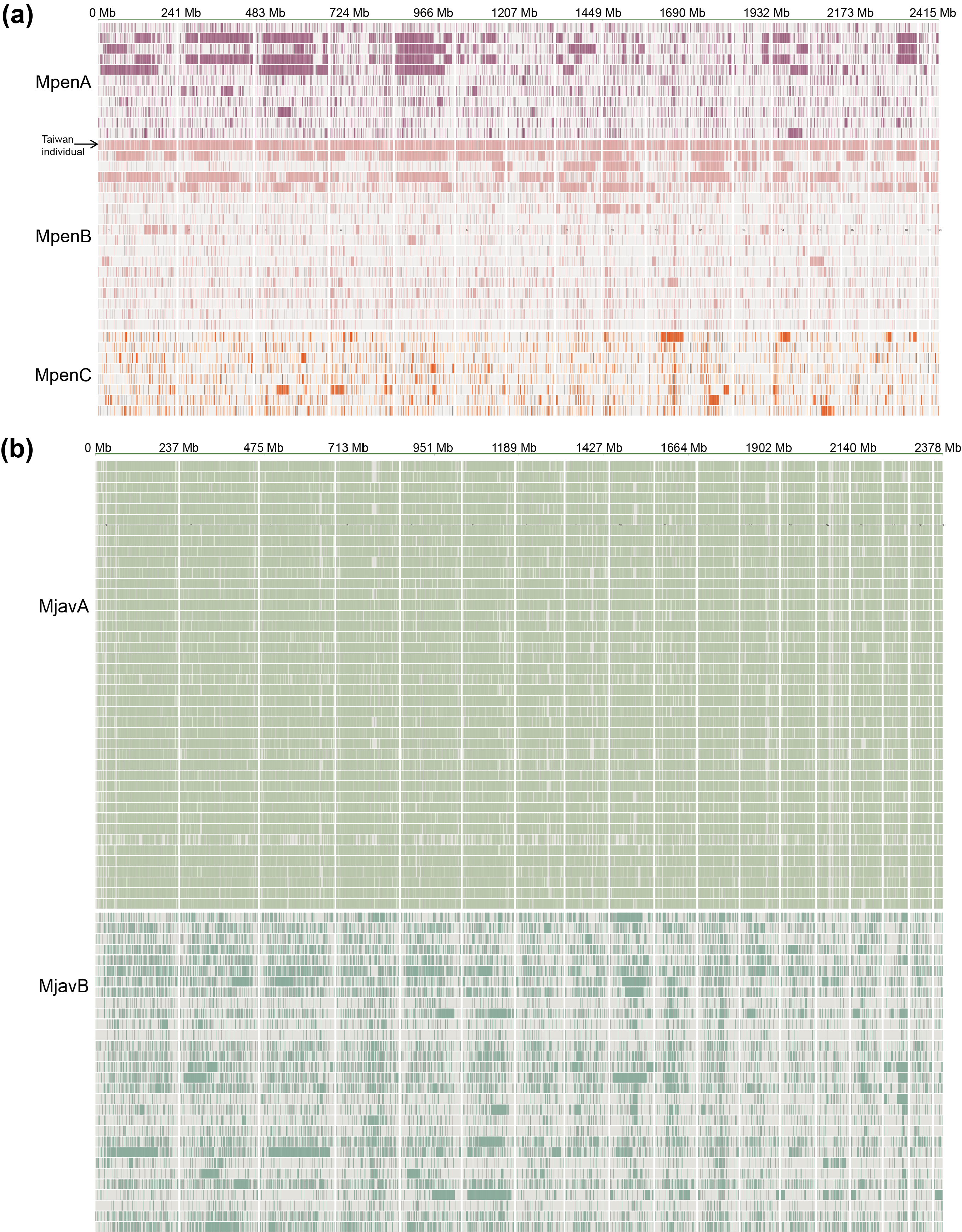


**Figure S9.** (a)The individual-level distribution of ROH larger than 100Kb in Chinese pangolin genomes. (b) The individual-level distribution of ROH larger than 100Kb in Malayan pangolin genomes. Each row represents an individual.


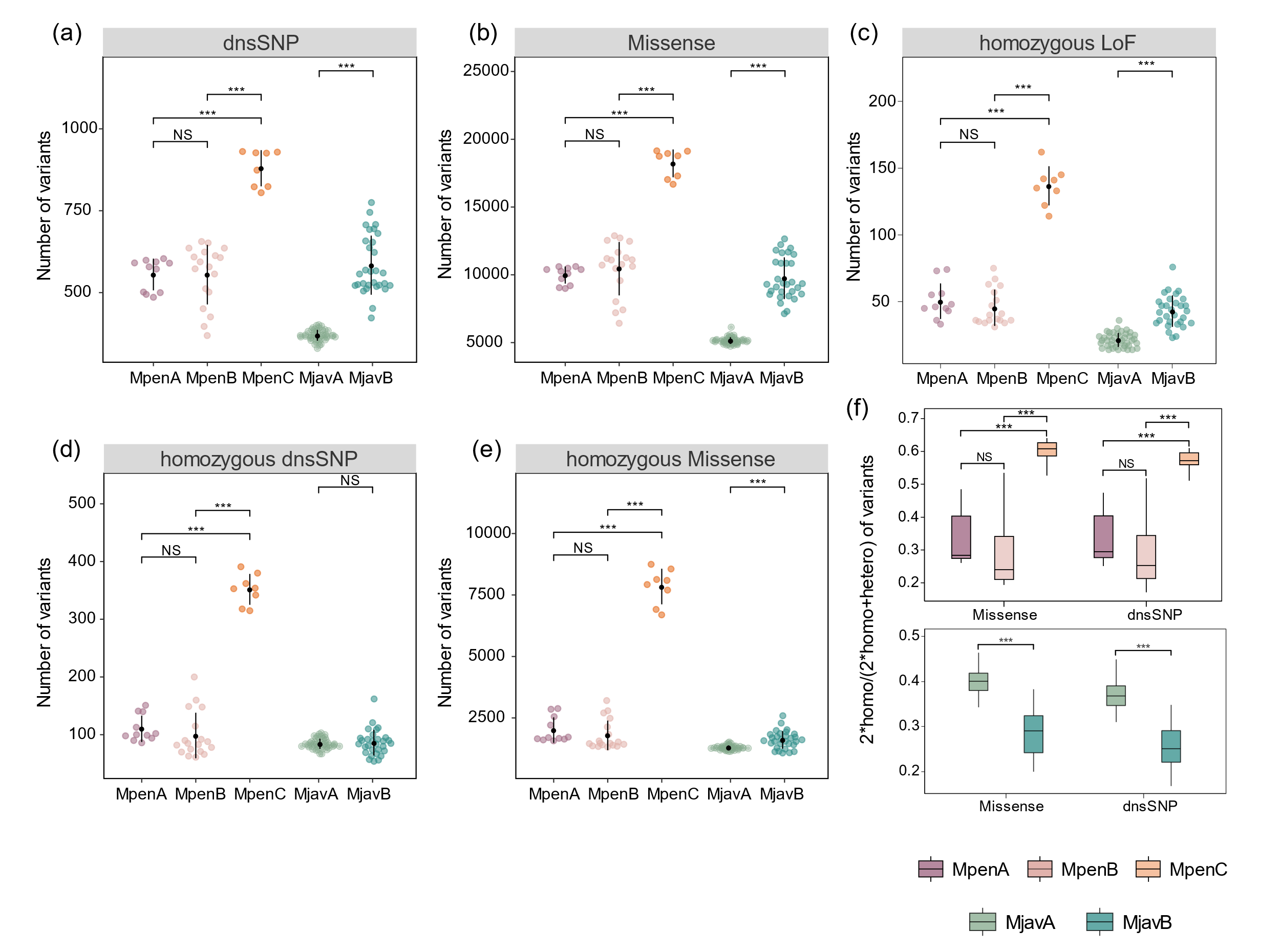


**Figure S10.** Total deleterious nonsynonymous SNP (dnsSNP) (a) and missense (b) mutations at the individual level were assessed across five populations of Chinese and Malayan pangolins. The number of individual-level homozygous LoF(c), dnsSNP (d) and missense (e) mutations across the five pangolin populations. (f) The ratio of homozygous missense mutations and dnsSNPs in Chinese and Malayan pangolin populations was calculated as the formula of : 2 × homozygous sites / (2 × homozygous sites + heterozygous site).

**
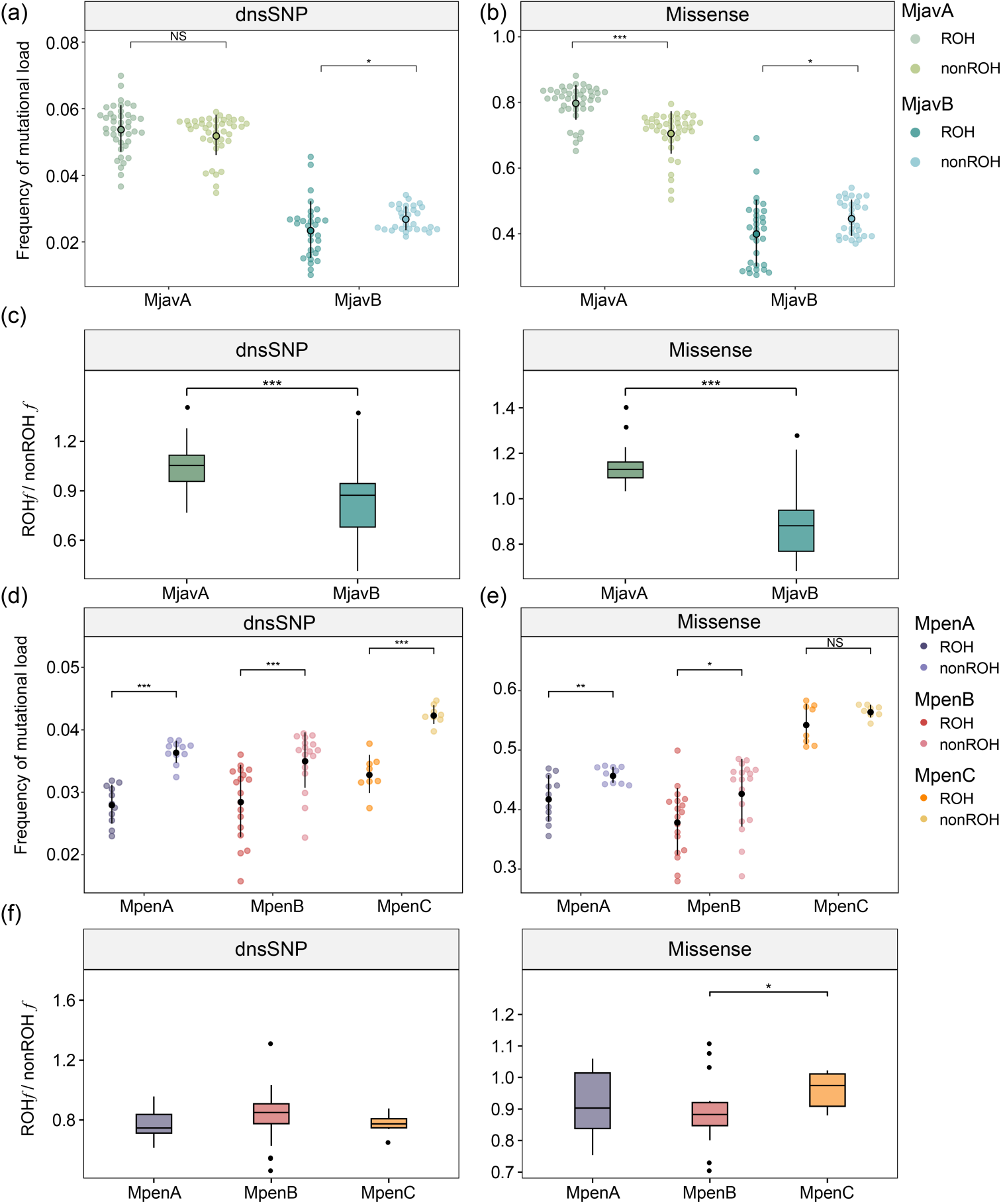
**

**Figure S11.** Dot plot showed the occurrence of dnsSNPs (a) and missense mutations (b) in the two Malayan pangolin populations calculated as the ratio of the number of mutational load to synonymous mutations in the ROH regions (ROH*f* ) or nonROH regions (nonROH*f* ) across the genome. The ratio of ROH*f* to nonROH*f* for dnsSNP and missense mutations in Malayan pangolin populations (c).Dot plot showed the occurrence of dnsSNPs (d) and missense mutations (e) in the three Chinese pangolin populations calculated as the ratio of the number of mutational load to synonymous mutations in the ROH regions or nonROH regions across the genome.The ratio of ROH*f* to nonROH*f* for dnsSNP and missense mutations in Chinese pangolin populations (f).

**
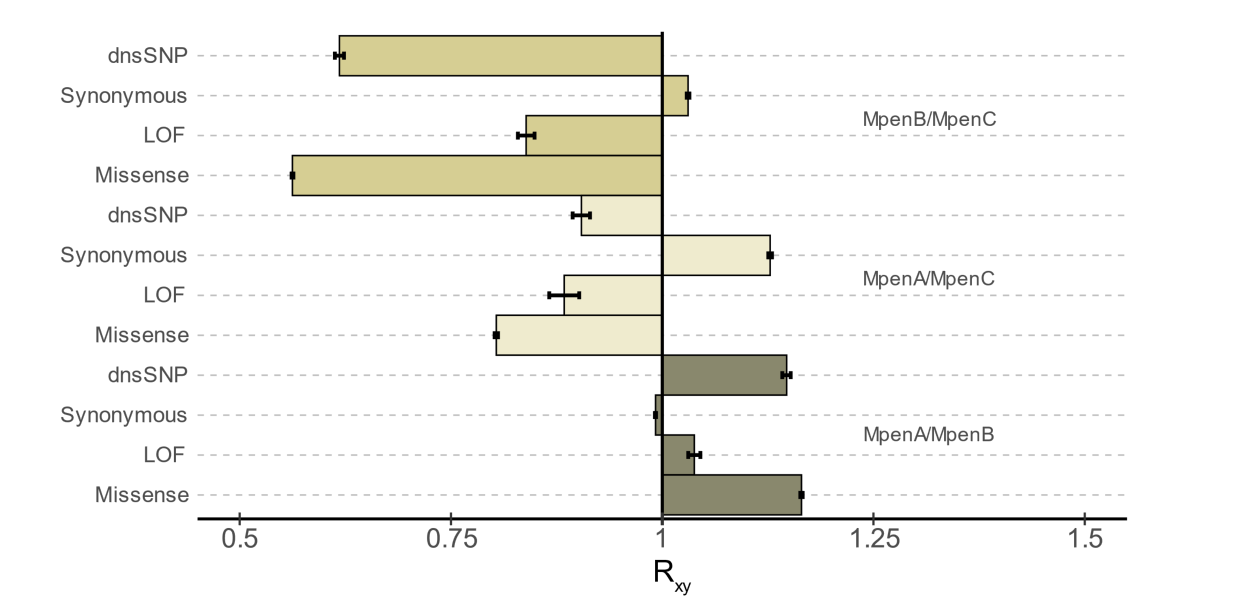
**

**Figure S12.** The Rxy ratio of derived alleles in x population to y population (x/y: MpenB/MpenC; MpenA/MpenC; MpenA/MpenB) for dnsSNP, synonymous, missense and LOF. The Rxy <1 indicated the population y has more derived alleles than population x.

**
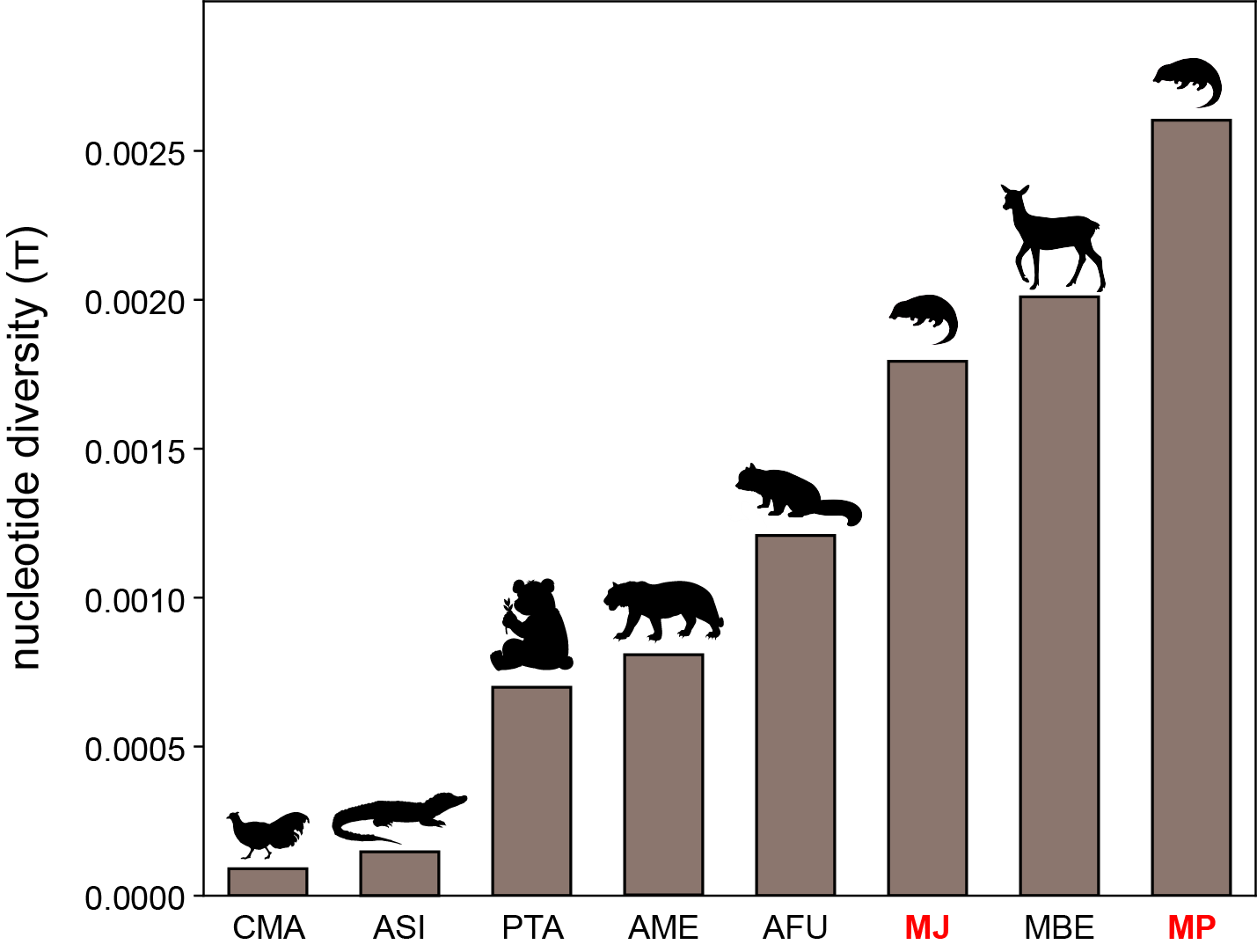
**

**Figure S13.** Comparison of genome-wide π of the Malayan pangolin and Chinese pangolin with other endangered species. Abbreviations along the x-axis are as follows: AFU: red panda (Ailurus fulgens); AME: giant panda (Ailuropoda melanoleuca); ASI: Chinese alligator (Alligator sinensis); CMA: brown eared pheasant (Crossoptilon mantchuricum); MBE: dwarf musk deer (Moschus berezovskii); MJ: Malayan pangolin (Manis javanica); MP: Chinese pangolin (Manis pentadactyla); PTA: Amur tiger (Panthera tigris altaica).


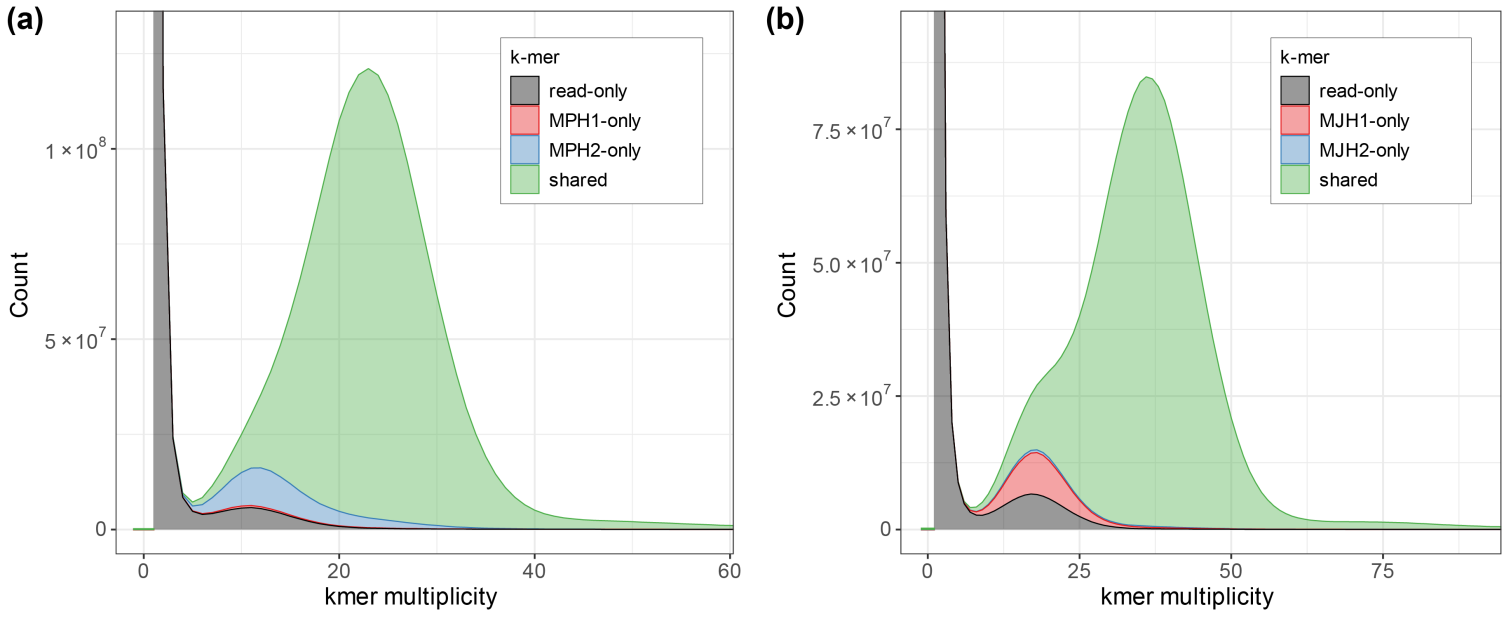


**Figure S14.** K-mer spectra plot estimated by Merqury. (a) K-mer spectra plot for the haplotype-resolved chromosome-level genome of MP. (b) K-mer spectra plot for the haplotype-resolved chromosome-level genome of MJ.


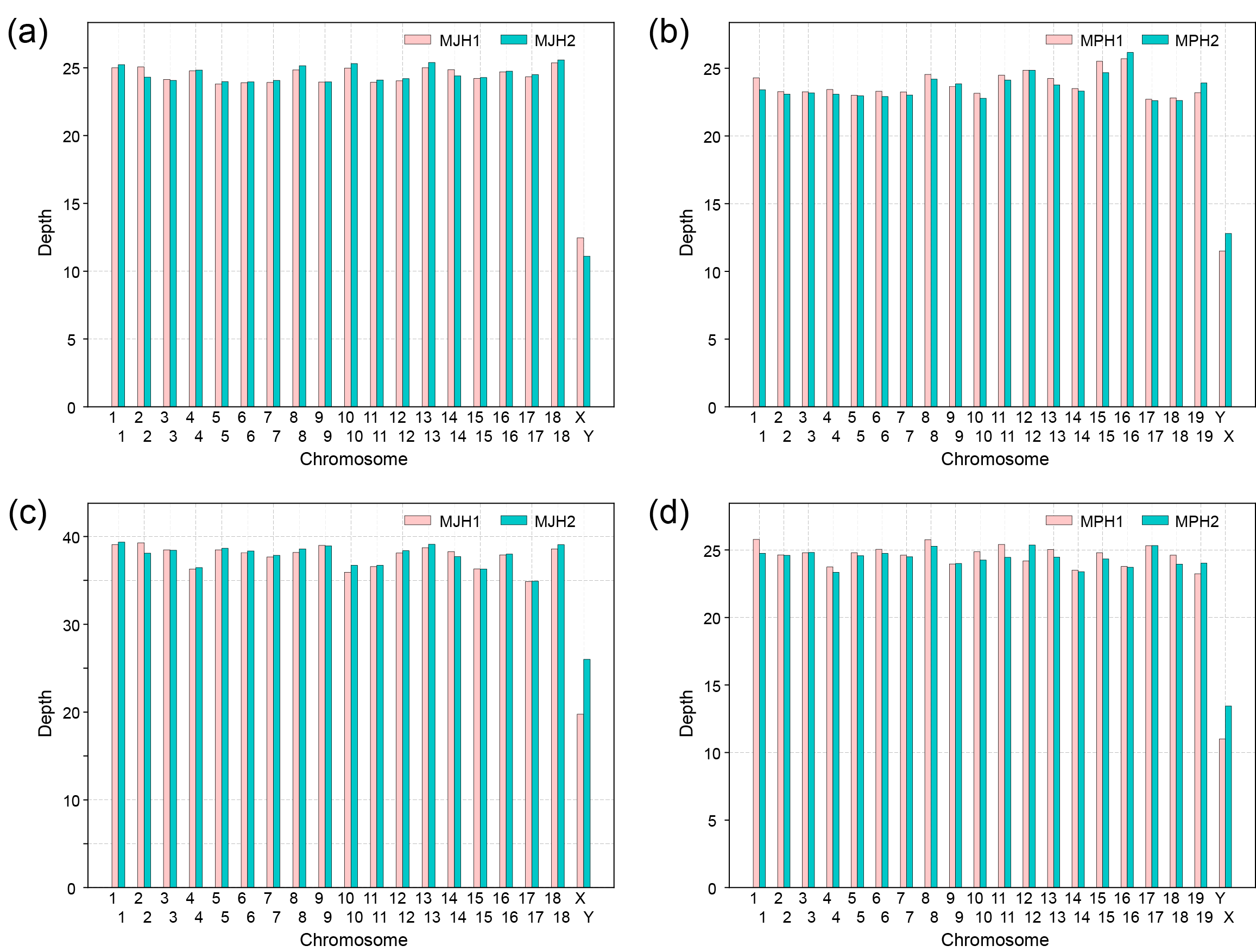


**Figure S15.** The sequencing depths of the two groups of haplotigs in both MJ and MP genomes. (a, b) Alignment with DNBSEQ read. (c, d) Alignment with PacBio HiFi reads.


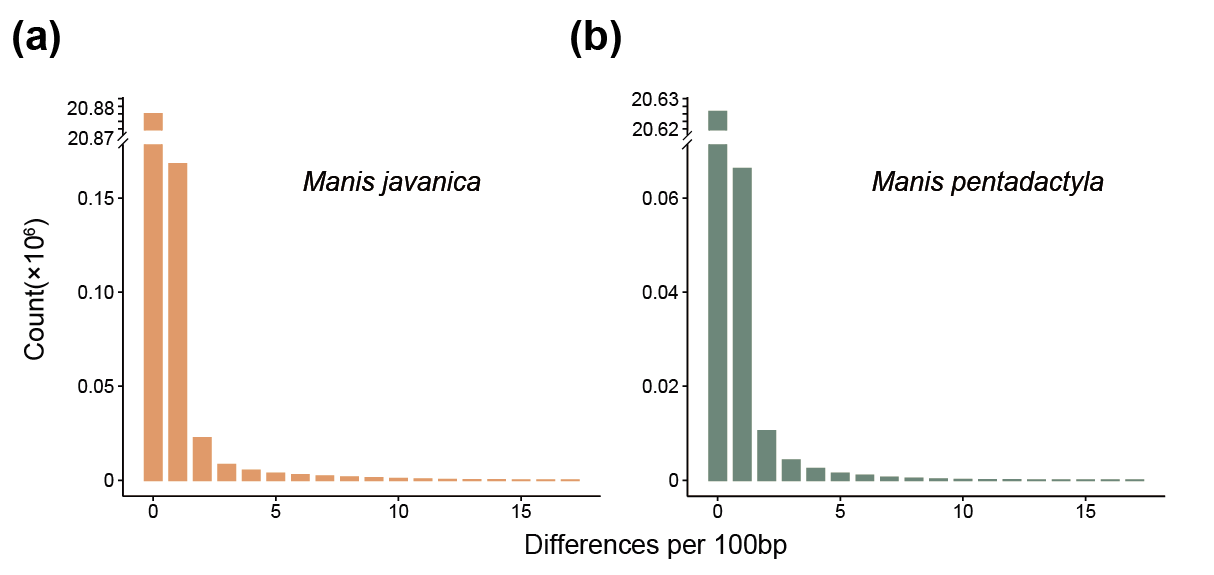


**Figure S16.**  Pairwise differences observed between the haploid genomes of Malayan and Chinese pangolin. The sliding window was set to be 100 bp.


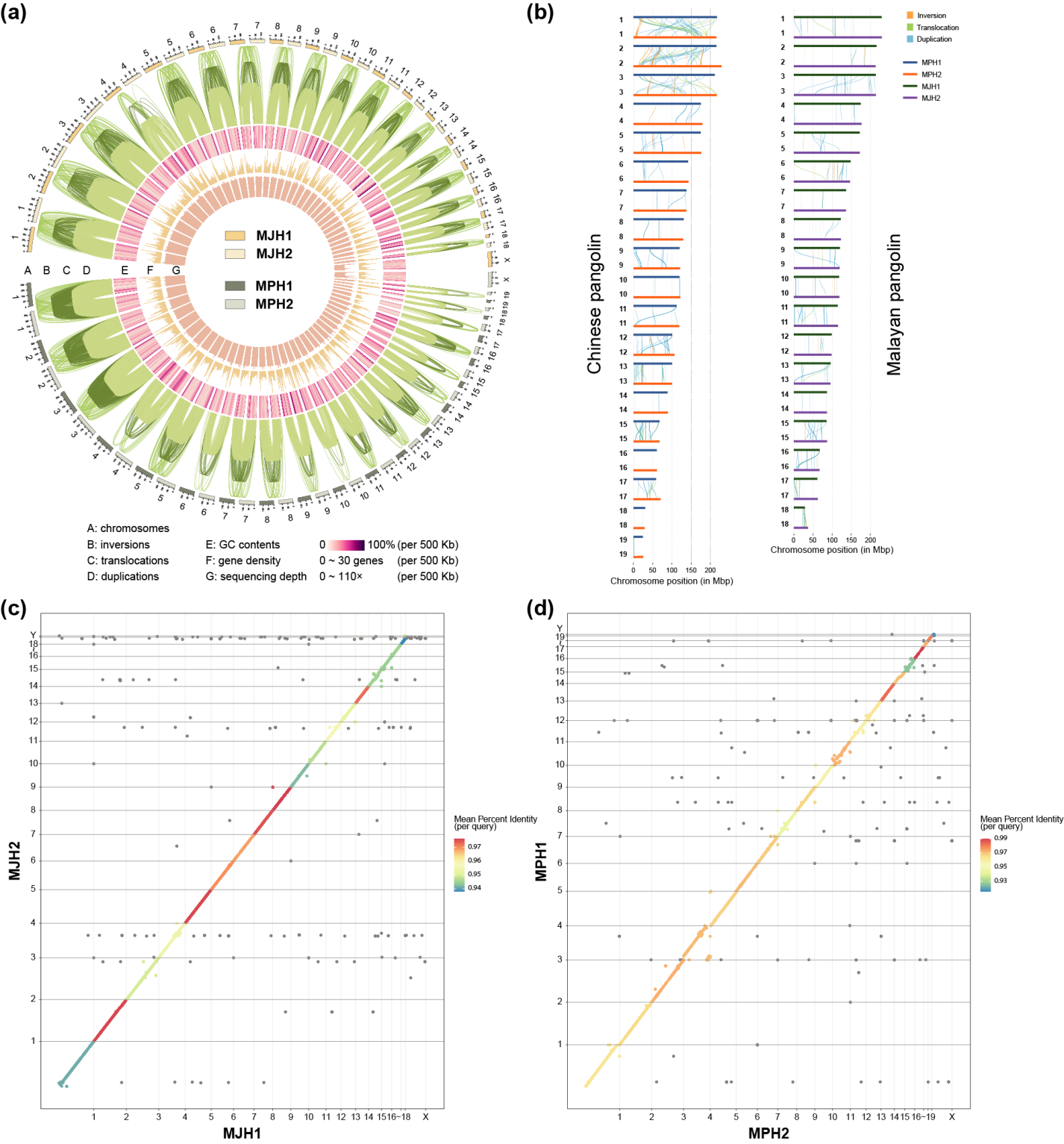


**Figure S17.** (a) Brief introduction and circos diagram of the two pangolin genomes. (b) Structural rearrangements between the two haplotypes of each chromosome in Malayan pangolin and Chinese pangolin genomes. (c) Dot plot between MJH1 (x-axis) and MJH2 (y-axis), plotted by pafCoordsDotPlotly. (d) Dot plot between MPH2 (x-axis) and MPH1 (y-axis), plotted by pafCoordsDotPlotly.


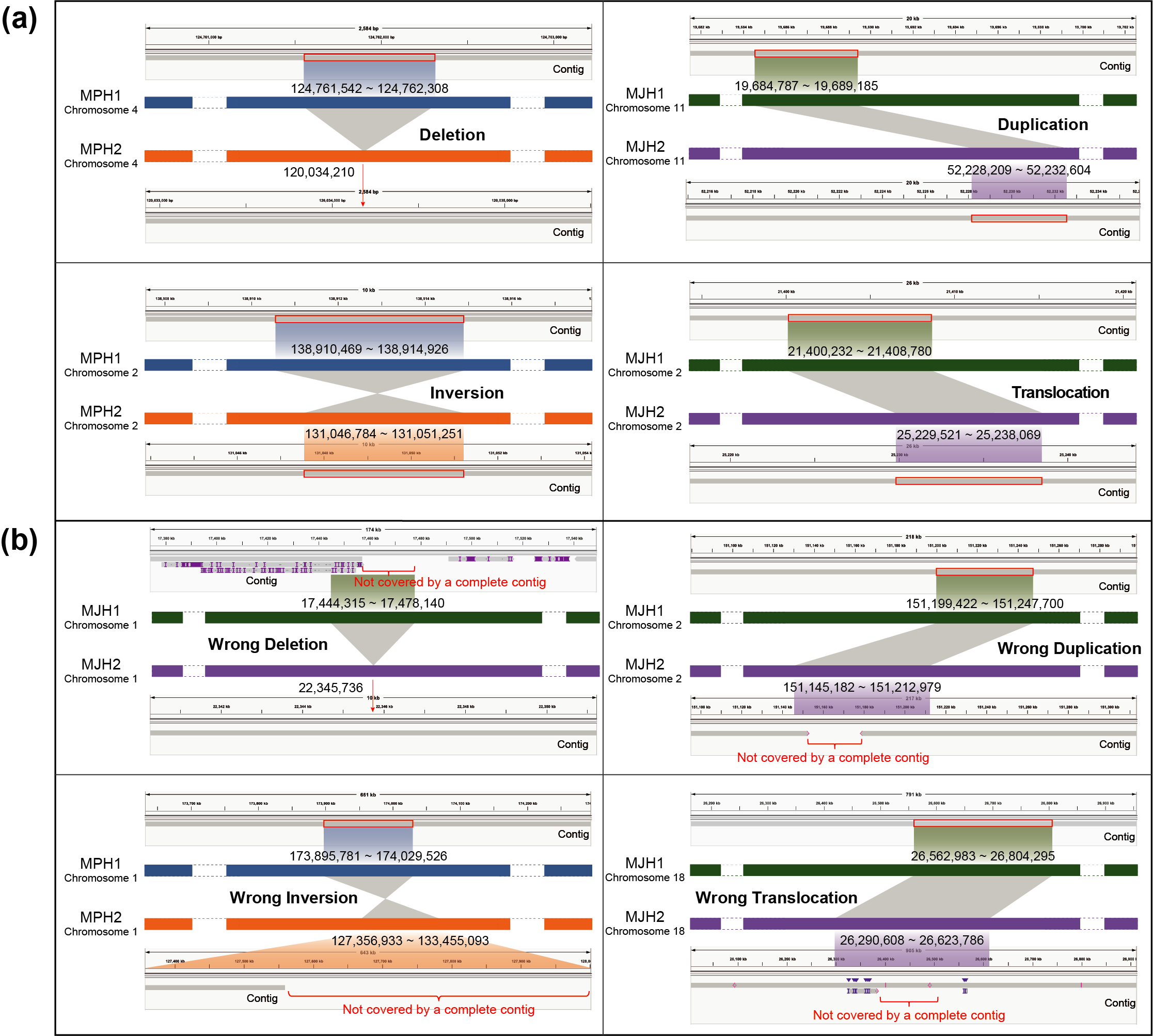


**Figure S18.** Validation of structural variants through contig mapping. Contigs are mapped to both the haploid genomes to verify structural variants and visualized by the IGV software. (a) The correct structural variants between the haploid genomes. The breakpoints of the SVs could be covered by complete contigs in both of the haploid genomes. (b) The incorrect structural variants between the haploid genomes. The breakpoints of the SVs in one of the two haploid genomes could not be covered by the complete contig. In the IGV screenshot, the gray bar represents the contig spanning over the SVs, and the contig corresponding SV regions were marked as the red box. All structural variants have been verified, and several are randomly displayed here.


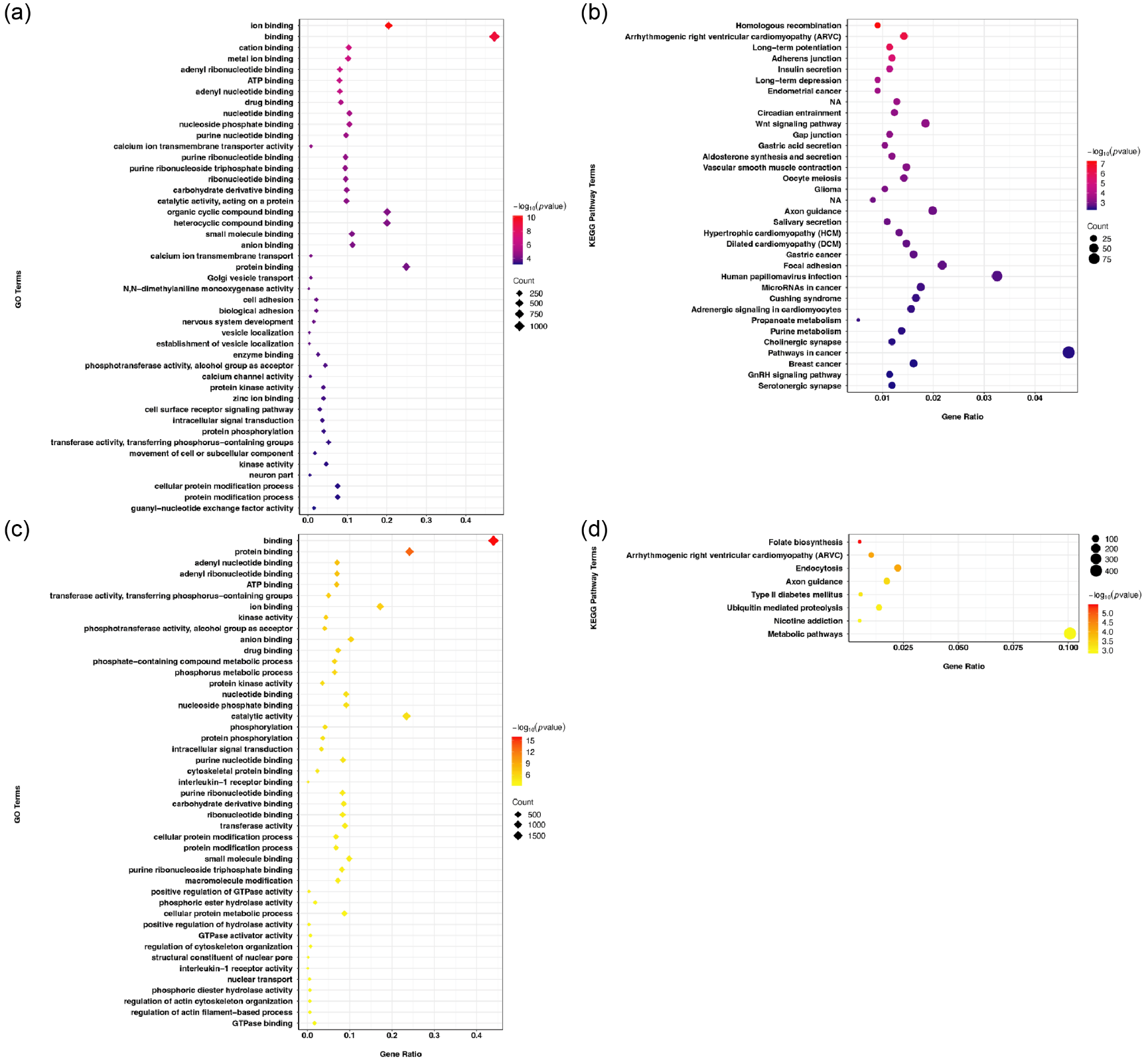


**Figure S19.** (a) The GO enrichment result of genes distributed in structural variants of Malayan pangolin. (b) The KEGG enrichment result of genes distributed in structural variants of Malayan pangolin. (c) The GO enrichment result of genes distributed in structural variants of Chinese pangolin. (d) The KEGG enrichment result of genes distributed in structural variants of Chinese pangolin.

**Supplemental Tables**

**Table S1.** Summarized sample information in this study.

| Database ID | Previous sample ID* | ID (In this study) | Population | Location | Mapping rate (%) | Coverage (%) | Mean Depth |
| --- | --- | --- | --- | --- | --- | --- | --- |
| CNP0001723 | GAEM0001 | GAEM0001 | MpenA | Guangdong, China | 99.48 | 98.34 | 11.85 |
| CNP0001723 | GAEM0008 | GAEM0008 | MpenA | Guangdong, China | 98.75 | 98.43 | 14.94 |
| CNP0001723 | GAEM0009 | GAEM0009 | MpenA | Guangdong, China | 99.64 | 99.09 | 14.00 |
| CNP0001723 | GAEM0010 | GAEM0010 | MpenA | Guangdong, China | 99.09 | 99.22 | 14.48 |
| CNP0001723 | GAEM0012 | GAEM0012 | MpenA | Guangdong, China | 99.68 | 99.07 | 14.98 |
| CNP0001723 | GAEM0014 | GAEM0014 | MpenA | Guangdong, China | 99.08 | 98.62 | 15.73 |
| CNP0001723 | GAEM0015 | GAEM0015 | MpenA | Guangdong, China | 99.36 | 98.53 | 15.77 |
| CNP0001723 | GAEM0016 | GAEM0016 | MpenA | Guangdong, China | 99.46 | 98.35 | 13.08 |
| CNP0001723 | GAEM0019 | GAEM0019 | MpenA | Guangdong, China | 99.22 | 98.46 | 14.68 |
| CNP0001723 | GAFM0020 | GAFM0020 | MpenA | Guangdong, China | 95.29 | 98.51 | 14.83 |
| CNP0001723 | GAFM0021 | GAFM0021 | MpenA | Guangdong, China | 99.48 | 99.22 | 14.99 |
| CNP0001723 | GAEM0006 | GAEM0006 | MpenB | Guangdong, China | 99.39 | 98.36 | 13.37 |
| CNP0001723 | GAEM0013 | GAEM0013 | MpenB | Guangdong, China | 99.70 | 98.38 | 14.12 |
| CNP0001723 | GAEM0017 | GAEM0017 | MpenB | Guangdong, China | 99.49 | 98.99 | 11.57 |
| CNP0001723 | GAFM0005 | GAFM0005 | MpenB | Guangdong, China | 99.13 | 98.51 | 17.91 |
| SRR+ | MP07 | MP07 | MpenB | Taiwan, China | 98.64 | 98.42 | 27.45 |
| SRR9018595 | MP05 | MP05 | MpenB | Yunnan, China | 99.49 | 99.41 | 45.02 |
| SRR9018596 | MP06 | MP06 | MpenB | Yunnan, China | 99.68 | 99.45 | 37.42 |
| SRR9018602 | MP14 | MP14 | MpenB | Yunnan, China | 99.70 | 99.38 | 29.77 |
| SRR9018604 | MP12 | MP12 | MpenB | Yunnan, China | 99.76 | 98.36 | 30.36 |
| SRR9018605 | MP13 | MP13 | MpenB | Yunnan, China | 99.69 | 98.45 | 38.25 |
| SRR9018583 | MP10 | MP10 | MpenB | Yunnan, China | 99.78 | 98.34 | 33.36 |
| SRR9018584 | MP11 | MP11 | MpenB | Yunnan, China | 99.45 | 99.54 | 39.97 |
| SRR9018585 | MP03 | MP03 | MpenB | Yunnan, China | 99.59 | 99.49 | 38.02 |
| SRR9018590 | MP04 | MP04 | MpenB | Yunnan, China | 99.90 | 99.40 | 38.11 |
| SRR9018591 | MP01 | MP01 | MpenB | Yunnan, China | 99.59 | 98.65 | 39.95 |
| SRR9018592 | MP02 | MP02 | MpenB | Yunnan, China | 99.74 | 99.50 | 36.09 |
| SRR9018593 | MP08 | MP08 | MpenB | Yunnan, China | 99.36 | 99.49 | 41.99 |
| SRR9018594 | MP09 | MP09 | MpenB | Yunnan, China | 99.77 | 98.59 | 37.20 |
| SRR9018600 | MP21 | MP21 | MpenC | Yunnan, China | 99.80 | 97.57 | 37.57 |
| SRR9018601 | MP22 | MP22 | MpenC | Yunnan, China | 98.51 | 97.70 | 41.98 |
| SRR9018603 | MP15 | MP15 | MpenC | Yunnan, China | 99.56 | 98.25 | 39.18 |
| SRR9018606 | MP18 | MP18 | MpenC | Sino-Burmese border | 99.80 | 98.59 | 42.52 |
| SRR9018607 | MP19 | MP19 | MpenC | Yunnan, China | 99.80 | 97.56 | 33.44 |
| SRR9018608 | MP16 | MP16 | MpenC | Sino-Burmese border | 99.90 | 98.27 | 35.26 |
| SRR9018609 | MP17 | MP17 | MpenC | Sino-Burmese border | 98.81 | 97.49 | 34.78 |
| SRR9018653 | MP23 | MP23 | MpenC | Yunnan, China | 99.75 | 97.55 | 27.84 |
| SRR9018672 | MJ63 | MJ63 | MjavB | Yunnan, China | 99.44 | 98.96 | 11.77 |
| SRR9018675 | MJ64 | MJ64 | MjavB | Yunnan, China | 99.88 | 98.74 | 11.74 |
| SRR9018674 | MJ65 | MJ65 | MjavB | Yunnan, China | 99.75 | 99.04 | 14.74 |
| SRR9018587 | MJ66 | MJ66 | MjavB | Yunnan, China | 99.83 | 98.80 | 11.72 |
| SRR9018586 | MJ67 | MJ67 | MjavB | Yunnan, China | 99.80 | 98.89 | 9.81 |
| SRR9018620 | MJ58 | MJ58 | MjavB | Yunnan, China | 99.63 | 98.88 | 10.08 |
| SRR9018629 | MJ57 | MJ57 | MjavB | Yunnan, China | 99.25 | 98.80 | 10.68 |
| SRR9018589 | MJ69 | MJ69 | MjavB | Yunnan, China | 99.74 | 99.12 | 12.34 |
| SRR9018597 | MJ73 | MJ73 | MjavB | Yunnan, China | 99.83 | 99.07 | 11.47 |
| SRR9018671 | MJ60 | MJ60 | MjavB | Yunnan, China | 99.85 | 98.88 | 15.65 |
| SRR9018599 | MJ72 | MJ72 | MjavB | Yunnan, China | 99.40 | 98.92 | 10.32 |
| SRR9018670 | MJ61 | MJ61 | MjavB | Yunnan, China | 99.67 | 99.31 | 11.61 |
| SRR9018598 | MJ71 | MJ71 | MjavB | Guangzhou, China | 99.67 | 99.19 | 13.09 |
| SRR9018673 | MJ62 | MJ62 | MjavB | Yunnan, China | 99.78 | 99.13 | 11.66 |
| SRR9018621 | MJ59 | MJ59 | MjavB | Yunnan, China | 99.85 | 99.23 | 12.44 |
| SRR9018588 | MJ70 | MJ70 | MjavB | Guangzhou, China | 99.80 | 99.28 | 15.35 |
| SRR9018627 | MJ55 | MJ55 | MjavB | Yunnan, China | 99.87 | 99.28 | 12.90 |
| SRR9018628 | MJ56 | MJ56 | MjavB | Yunnan, China | 99.75 | 99.02 | 10.44 |
| SRR9018626 | MJ54 | MJ54 | MjavB | Yunnan, China | 99.59 | 99.29 | 11.12 |
| SRR9018625 | MJ53 | MJ53 | MjavB | Yunnan, China | 99.82 | 99.12 | 9.84 |
| SRR9018624 | MJ52 | MJ52 | MjavB | Yunnan, China | 99.77 | 99.09 | 10.77 |
| SRR9018623 | MJ51 | MJ51 | MjavB | Yunnan, China | 99.73 | 98.93 | 9.27 |
| SRR9018622 | MJ50 | MJ50 | MjavB | Yunnan, China | 99.30 | 98.33 | 10.85 |
| SRR9018613 | MJ46 | MJ46 | MjavB | Guangzhou, China | 99.70 | 99.01 | 14.91 |
| SRR9018610 | MJ49 | MJ49 | MjavB | Yunnan, China | 99.52 | 98.72 | 9.42 |
| SRR9018611 | MJ48 | MJ48 | MjavB | Yunnan, China | 99.87 | 99.23 | 11.96 |
| SRR9018612 | MJ47 | MJ47 | MjavB | Yunnan, China | 99.47 | 98.73 | 10.61 |
| SRR9018615 | MJ44 | MJ44 | MjavB | Yunnan, China | 98.60 | 99.08 | 12.07 |
| SRR9018614 | MJ45 | MJ45 | MjavB | Guangzhou, China | 99.83 | 98.65 | 13.59 |
| SRR9018616 | MJ43 | MJ43 | MjavB | Yunnan, China | 99.84 | 98.83 | 11.31 |
| SRR9018617 | MJ42 | MJ42 | MjavA | Sino-Burmese border | 96.73 | 98.83 | 12.84 |
| SRR9018618 | MJ41 | MJ41 | MjavA | Sino-Burmese border | 99.67 | 98.67 | 11.85 |
| SRR9018619 | MJ40 | MJ40 | MjavA | Yunnan, China | 99.80 | 98.98 | 15.89 |
| SRR9018630 | MJ25A | MJ25A | MjavA | Sino-Burmese border | 99.89 | 98.45 | 12.74 |
| SRR9018631 | MJ24 | MJ24 | MjavA | Sino-Burmese border | 99.85 | 97.94 | 12.93 |
| SRR9018632 | MJ27 | MJ27 | MjavA | Yunnan, China | 99.80 | 98.92 | 17.66 |
| SRR9018633 | MJ26 | MJ26 | MjavA | Myanmar | 99.41 | 98.33 | 14.61 |
| SRR9018634 | MJ21 | MJ21 | MjavA | Sino-Burmese border | 91.38 | 98.61 | 10.36 |
| SRR9018635 | MJ20 | MJ20 | MjavA | Yunnan, China | 97.90 | 98.82 | 12.99 |
| SRR9018636 | MJ23 | MJ23 | MjavA | Sino-Burmese border | 99.59 | 98.54 | 14.01 |
| SRR9018637 | MJ22 | MJ22 | MjavA | Sino-Burmese border | 99.85 | 98.60 | 14.83 |
| SRR9018638 | MJ29 | MJ29 | MjavA | Yunnan, China | 99.77 | 98.76 | 11.65 |
| SRR9018639 | MJ28 | MJ28 | MjavA | Sino-Burmese border | 99.77 | 98.76 | 13.05 |
| SRR9018640 | MJ36 | MJ36 | MjavA | Sino-Burmese border | 99.80 | 98.44 | 11.65 |
| SRR9018641 | MJ37 | MJ37 | MjavA | Yunnan, China | 99.86 | 98.90 | 13.80 |
| SRR9018642 | MJ34 | MJ34 | MjavA | Sino-Burmese border | 99.75 | 98.23 | 11.66 |
| SRR9018643 | MJ35 | MJ35 | MjavA | Sino-Burmese border | 99.18 | 97.87 | 10.40 |
| SRR9018644 | MJ32 | MJ32 | MjavA | Sino-Burmese border | 99.81 | 98.22 | 14.56 |
| SRR9018645 | MJ33 | MJ33 | MjavA | Sino-Burmese border | 98.60 | 98.24 | 10.72 |
| SRR9018646 | MJ30 | MJ30 | MjavA | Sino-Burmese border | 99.90 | 98.43 | 12.69 |
| SRR9018647 | MJ31 | MJ31 | MjavA | Yunnan, China | 99.80 | 97.15 | 10.38 |
| SRR9018648 | MJ38 | MJ38 | MjavA | Yunnan, China | 99.74 | 98.51 | 13.25 |
| SRR9018649 | MJ39 | MJ39 | MjavA | Yunnan, China | 99.85 | 98.77 | 11.74 |
| SRR9018650 | MJ03 | MJ03 | MjavA | Sino-Burmese border | 98.86 | 98.76 | 11.66 |
| SRR9018651 | MJ02 | MJ02 | MjavA | Sino-Burmese border | 98.86 | 98.54 | 13.00 |
| SRR9018652 | MJ01 | MJ01 | MjavA | Sino-Burmese border | 99.86 | 98.90 | 13.33 |
| SRR9018654 | MJ07 | MJ07 | MjavA | Yunnan, China | 99.66 | 98.92 | 14.89 |
| SRR9018655 | MJ06 | MJ06 | MjavA | Yunnan, China | 99.75 | 98.30 | 10.45 |
| SRR9018656 | MJ05 | MJ05 | MjavA | Sino-Burmese border | 99.36 | 97.72 | 11.94 |
| SRR9018657 | MJ04 | MJ04 | MjavA | Sino-Burmese border | 99.79 | 98.49 | 12.44 |
| SRR9018658 | MJ09 | MJ09 | MjavA | Yunnan, China | 99.88 | 98.56 | 13.66 |
| SRR9018659 | MJ08 | MJ08 | MjavA | Yunnan, China | 99.76 | 98.77 | 12.21 |
| SRR9018660 | MJ18 | MJ18 | MjavA | Sino-Burmese border | 99.72 | 98.83 | 12.76 |
| SRR9018661 | MJ19 | MJ19 | MjavA | Sino-Burmese border | 99.79 | 98.58 | 13.46 |
| SRR9018662 | MJ14 | MJ14 | MjavA | Sino-Burmese border | 99.84 | 98.51 | 16.24 |
| SRR9018663 | MJ15 | MJ15 | MjavA | Sino-Burmese border | 99.84 | 94.90 | 12.25 |
| SRR9018664 | MJ16 | MJ16 | MjavA | Yunnan, China | 99.86 | 98.78 | 13.32 |
| SRR9018665 | MJ17 | MJ17 | MjavA | Yunnan, China | 99.85 | 98.50 | 14.04 |
| SRR9018666 | MJ10 | MJ10 | MjavA | Sino-Burmese border | 99.74 | 98.54 | 14.34 |
| SRR9018667 | MJ11 | MJ11 | MjavA | Yunnan, China | 99.79 | 98.47 | 11.46 |
| SRR9018668 | MJ12 | MJ12 | MjavA | Sino-Burmese border | 99.81 | 98.50 | 13.83 |
| SRR9018669 | MJ13 | MJ13 | MjavA | Yunnan, China | 99.85 | 98.64 | 11.02 |

*SRR+ represents sequence IDs of SRR770301, SRR770303, SRR770305, SRR770306, SRR770309, SRR770310, SRR770312, SRR770314, SRR770316, SRR770321, SRR770329, SRR770335, SRR770337, SRR770338, SRR770339, SRR770342, SRR770349, SRR770350, SRR770351, SRR770352 in the Choo *et al* ’s study.[7]

**Table S2.** Information of five populations in Chinese and Malayan pangolin included in this study.

| Species | Population | Sampling Range | Number | Coverage (%) | Mean Depth |
| --- | --- | --- | --- | --- | --- |
| Chinese pangolin | MpenA | Guangdong, China | 11 | 98.71±0.36 | 14.48±1.15 |
|  | MpenB | Guangdong/Yunnan/Taiwan, China | 18 | 98.93±0.53 | 31.66±3.94 |
|  | MpenC | Myanmar | 8 | 97.87±0.66 | 36.57±4.59 |
| Malayan pangolin | MjavA | Malaysia, Myanmar | 42 | 98.43±1.13 | 12.92±1.47 |
|  | MjavB | Guangdong, Yunnan, China | 30 | 98.99±0.26 | 11.78±1.73 |

**Table S3.** Statistics of genome assemblies for the Chinese pangolin and Malayan pangolin.

| species | Parameters | scaffold | | contig | | NGenome scaffold | | NGenome contig | |
| --- | --- | --- | --- | --- | --- | --- | --- | --- | --- |
|  |  | length(bp) | number | length(bp) | number | length(bp) | number | length(bp) | number |
| Chinese pangolin | Max length | 234,250,095 |  | 209,591,311 |  |  |  |  |  |
|  | N10 | 223,341,758 | 2 | 172,166,903 | 2 | 223,341,758 | 2 | 172,166,903 | 2 |
|  | N20 | 222,052,874 | 3 | 103,001,611 | 4 | 222,052,874 | 3 | 103,001,611 | 4 |
|  | N30 | 188,561,458 | 4 | 91,678,018 | 6 | 188,561,458 | 4 | 73,071,967 | 7 |
|  | N40 | 147,484,612 | 6 | 64,387,143 | 10 | 147,484,612 | 6 | 64,199,787 | 11 |
|  | N50 | 140,708,684 | 7 | 56,151,610 | 15 | 140,538,157 | 8 | 11,516,433 | 16 |
|  | N60 | 133,589,572 | 9 | 3,038,807 | 54 | 126,095,547 | 10 | 2,049,909 | 81 |
|  | N70 | 125,153,789 | 11 | 644,773 | 260 | 125,067,320 | 12 | 435,651 | 408 |
|  | N80 | 105,454,770 | 14 | 210,985 | 1,052 | 91,621,832 | 15 | 157,024 | 1,536 |
|  | N90 | 75,334,271 | 17 | 82,622 | 3,093 | 62,819,954 | 18 | 52,991 | 4,595 |
|  | Total length | 2,641,556,940 |  | 2,640,920,470 |  |  |  |  |  |
|  | GC rate | 0.41 |  | 0 .41 |  |  |  |  |  |
|  | Estimated genome size: | 2,856,353,075 |  |  |  |  |  |  |  |
| Malayan pangolin | Max length | 241,980,946 |  | 166,563,371 |  |  |  |  |  |
|  | N10 | 226,996,534 | 2 | 105,093,063 | 2 | 226,996,534 | 2 | 105,093,063 | 2 |
|  | N20 | 217,799,483 | 3 | 89,489,852 | 5 | 217,799,483 | 3 | 89,489,852 | 5 |
|  | N30 | 183,362,737 | 4 | 74,196,724 | 8 | 183,362,737 | 4 | 74,196,724 | 8 |
|  | N40 | 174,158,066 | 5 | 59,801,223 | 12 | 174,158,066 | 5 | 59,801,223 | 12 |
|  | N50 | 141,798,549 | 7 | 46,223,514 | 16 | 141,798,549 | 7 | 46,223,514 | 16 |
|  | N60 | 125,332,986 | 9 | 974,394 | 138 | 125,332,986 | 9 | 974,257 | 139 |
|  | N70 | 119,352,310 | 11 | 237,539 | 745 | 119,352,310 | 11 | 236,916 | 750 |
|  | N80 | 111,219,906 | 13 | 97,785 | 2,514 | 111,219,906 | 13 | 97,460 | 2,526 |
|  | N90 | 88,959,520 | 16 | 41,484 | 6,609 | 88,959,520 | 16 | 41,213 | 6,641 |
|  | Total length | 2,560,052,348 |  | 2,559,088,858 |  |  |  |  |  |
|  | GC rate | 0.41 |  | 0.41 |  |  |  |  |  |
|  | Estimated genome size | 2,645,358,752 |  |  |  |  |  |  |  |

**Table S4.** The length of each chromosome in the Chinese pangolin and Malayan pangolin genomes.

| Species | Assembly | Chromosome | Length | Assembly | Chromosome | Length | Assembly | Chromosome | Length | Note |
| --- | --- | --- | --- | --- | --- | --- | --- | --- | --- | --- |
| Chinese pangolin | MP | 1 | 234,250,095 | MPH1 | 1 | 235,368,218 | MPH2 | 1 | 229,045,779 |  |
|  |  | 2 | 223,341,758 |  | 2 | 217,496,584 |  | 2 | 217,150,986 |  |
|  |  | 3 | 222,052,874 |  | 3 | 216,135,000 |  | 3 | 216,110,645 |  |
|  |  | 4 | 188,561,458 |  | 4 | 175,330,372 |  | 4 | 179,857,540 |  |
|  |  | 5 | 178,218,924 |  | 5 | 175,236,041 |  | 5 | 176,442,669 |  |
|  |  | 6 | 147,484,612 |  | 6 | 142,429,255 |  | 6 | 143,338,891 |  |
|  |  | 7 | 140,708,684 |  | 7 | 137,907,067 |  | 7 | 137,779,352 |  |
|  |  | 8 | 133,589,572 |  | 8 | 130,911,117 |  | 8 | 129,403,270 |  |
|  |  | 9 | 126,095,547 |  | 9 | 120,132,553 |  | 9 | 125,966,341 |  |
|  |  | 10 | 125,153,789 |  | 10 | 120,001,735 |  | 10 | 122,024,982 |  |
|  |  | 11 | 125,067,320 |  | 11 | 116,951,043 |  | 11 | 122,797,681 |  |
|  |  | 12 | 110,249,379 |  | 12 | 101,154,256 |  | 12 | 106,854,739 |  |
|  |  | 13 | 105,454,770 |  | 13 | 100,605,500 |  | 13 | 100,647,326 |  |
|  |  | 14 | 91,621,832 |  | 14 | 88,918,068 |  | 14 | 89,667,640 |  |
|  |  | 15 | 76,432,552 |  | 15 | 70,219,463 |  | 15 | 70,769,208 |  |
|  |  | 16 | 75,334,271 |  | 16 | 68,067,788 |  | 16 | 67,816,682 |  |
|  |  | 17 | 62,819,954 |  | 17 | 60,928,134 |  | 17 | 61,094,975 |  |
|  |  | 18 | 29,443,699 |  | 18 | 30,682,851 |  | 18 | 29,195,092 |  |
|  |  | 19 | 25,172,177 |  | 19 | 24,450,634 |  | 19 | 25,614,235 |  |
|  |  | X | 140,538,157 |  | - | - |  | X | 136,637,350 | X |
|  |  | Y | 6,582,812 |  | Y | 6,480,166 |  | - | - | Y-link |
| Malayan pangolin | MJ | 1 | 241,980,946 | MJH1 | 1 | 229,715,409 | MJH2 | 1 | 230,554,844 |  |
|  |  | 2 | 226,996,534 |  | 2 | 217,686,774 |  | 2 | 214,924,526 |  |
|  |  | 3 | 217,799,483 |  | 3 | 216,481,707 |  | 3 | 214,062,000 |  |
|  |  | 4 | 183,362,737 |  | 4 | 175,001,930 |  | 4 | 177,315,679 |  |
|  |  | 5 | 174,158,066 |  | 5 | 172,591,800 |  | 5 | 172,452,380 |  |
|  |  | 6 | 150,327,384 |  | 6 | 148,391,949 |  | 6 | 147,126,060 |  |
|  |  | 7 | 139,175,180 |  | 7 | 136,751,402 |  | 7 | 136,188,358 |  |
|  |  | 8 | 125,332,986 |  | 8 | 123,021,755 |  | 8 | 123,631,466 |  |
|  |  | 9 | 125,305,609 |  | 9 | 122,852,000 |  | 9 | 123,152,000 |  |
|  |  | 10 | 119,352,310 |  | 10 | 120,532,494 |  | 10 | 121,452,060 |  |
|  |  | 11 | 119,084,320 |  | 11 | 118,427,019 |  | 11 | 119,267,393 |  |
|  |  | 12 | 111,219,906 |  | 12 | 99,051,578 |  | 12 | 98,886,362 |  |
|  |  | 13 | 101,123,689 |  | 13 | 96,344,791 |  | 13 | 95,937,502 |  |
|  |  | 14 | 93,993,658 |  | 14 | 87,726,861 |  | 14 | 86,954,087 |  |
|  |  | 15 | 88,959,520 |  | 15 | 86,725,000 |  | 15 | 86,700,498 |  |
|  |  | 16 | 71,213,982 |  | 16 | 67,594,591 |  | 16 | 67,066,185 |  |
|  |  | 17 | 64,558,851 |  | 17 | 61,531,000 |  | 17 | 62,434,790 |  |
|  |  | 18 | 25,285,487 |  | 18 | 29,049,281 |  | 18 | 37,226,000 |  |
|  |  | X | 141,798,549 |  | X | 137,325,962 |  | - | - | X |
|  |  | Y | 6,250,399 |  | - | - |  | Y | 6,328,716 | Y-link |

**Table S5.** The overall statistics of repeats in the Chinese and Malayan pangolin genomes.

| Type | Malayan pangolin | | Chinese pangolin | |
| --- | --- | --- | --- | --- |
|  | Length (bp) | % of genome | Length (bp) | % of genome |
| Trf | 54,517,337 | 2.13 | 47,435,098 | 1.80 |
| Repeatmasker | 605,815,303 | 23.66 | 660,277,835 | 25.00 |
| Proteinmask | 343,222,016 | 13.41 | 369,872,247 | 14.00 |
| *De novo* | 819,343,499 | 32.01 | 889,759,712 | 33.68 |
| Total | 1,260,918,531 | 49.25 | 1,332,409,074 | 50.44 |

**Table S6.** Statistics of the annotated genes in Chinese and Malayan pangolin genomes.

| Item | Chinese pangolin | Malayan pangolin |
| --- | --- | --- |
| the total number of genes | 19,886 | 19,680 |
| the total mRNA length (bp) | 791,490,366 | 853,897,614 |
| the average of mRNA length | 39,801.39 | 43,389.11 |
| the average of CDS length | 1614.86 | 1664.77 |
| the average of exon number | 9.22 | 9.47 |
| the average of exon length | 175.07 | 175.75 |
| the average of intron length | 4643.23 | 4924.91 |
| the total number of exons | 183,431 | 186,411 |
| the total number of introns | 163,545 | 166,731 |
| the total intron length | 759,377,325 | 821,135,031 |

**Table S7.** BUSCO analysis of genome assemblies and gene sets in this study.

| Genome | BUSCO scores | database | Parameter |
| --- | --- | --- | --- |
| MJ | C:97.5% [S:95.6%, D:1.9%], F:1.1%, M:1.4%, n:3354 | vertebrata_odb10 | -m genome |
| MJH1 | C:97.4% [S:95.6%, D:1.8%], F:1.1%, M:1.5%, n:3354 | vertebrata_odb10 | -m genome |
| MJH2 | C:95.9% [S:94.2%, D:1.7%], F:1.2%, M:2.9%, n:3354 | vertebrata_odb10 | -m genome |
| MP | C:98.0% [S:95.4%, D:2.6%], F:0.8%, M:1.2%, n:3354 | vertebrata_odb10 | -m genome |
| MPH1 | C:94.8% [S:92.5%, D:2.3%], F:1.0%, M:4.2%, n:3354 | vertebrata_odb10 | -m genome |
| MPH2 | C:97.8% [S:95.2%, D:2.6%], F:0.8%, M:1.4%, n:3354 | vertebrata_odb10 | -m genome |
| MJ | C:98.0% [S:97.2%, D:0.8%], F:1.0%, M:1.0%, n:3354 | vertebrata_odb10 | -m proteins |
| MP | C:96.7% [S:95.6%, D:1.1%], F:1.9%, M:1.4%, n:3354 | vertebrata_odb10 | -m proteins |

**Table S8.** Statistics of functional annotation for the Malayan and Chinese pangolin’s gene sets.

|  | Values | Total | Swissprot | KEGG | TrEMBL | Interpro | GO | Overall |
| --- | --- | --- | --- | --- | --- | --- | --- | --- |
| MJ | Number | 19,680 | 18,787 | 17,146 | 18,951 | 19,508 | 14,509 | 19,575 |
|  | Percentage | 100% | 95.46% | 87.12% | 96.30% | 99.13% | 73.72% | 99.47% |
| MP | Number | 19,886 | 18,994 | 17,321 | 19,145 | 19,717 | 14,567 | 19,792 |
|  | Percentage | 100% | 95.51% | 87.10% | 96.27% | 99.15% | 73.25% | 99.53% |

**Table S9.** Statistics of ncRNA annotation.

| Species | Category | | number | Average length (bp) | Total length (bp) | % of genome |
| --- | --- | --- | --- | --- | --- | --- |
| Chinese pangolin | miRNA | | 1,348 | 76.55 | 103,188 | 0.0036 |
|  | tRNA | | 350 | 74.94 | 26,230 | 0.0009 |
|  | rRNA | rRNA | 435 | 340.11 | 147,946 | 0.0052 |
|  |  | 18S | 40 | 1,137.75 | 45,510 | 0.0016 |
|  |  | 28S | 260 | 343.15 | 89,218 | 0.0031 |
|  |  | 5.8S | 22 | 155.64 | 3,424 | 0.0001 |
|  |  | 5S | 113 | 86.67 | 9,794 | 0.0003 |
|  | snRNA | snRNA | 1,412 | 120.47 | 170,110 | 0.006 |
|  |  | CD-box | 253 | 91.48 | 23,145 | 0.0008 |
|  |  | HACA-box | 178 | 136.99 | 24,384 | 0.0009 |
|  |  | splicing | 961 | 124.28 | 119,430 | 0.0042 |
| Malayan pangolin | miRNA | | 1,296 | 76.34 | 98,936 | 0.0037 |
|  | tRNA | | 806 | 75.2 | 60,615 | 0.0023 |
|  | rRNA | rRNA | 288 | 277.74 | 79,989 | 0.003 |
|  |  | 18S | 29 | 877.14 | 25,437 | 0.0009 |
|  |  | 28S | 125 | 328.26 | 41,032 | 0.0015 |
|  |  | 5.8S | 11 | 148.18 | 1,630 | 0.0001 |
|  |  | 5S | 123 | 96.67 | 11,890 | 0.0004 |
|  | snRNA | snRNA | 1,521 | 123.44 | 187,755 | 0.007 |
|  |  | CD-box | 227 | 92.37 | 20,968 | 0.0008 |
|  |  | HACA-box | 162 | 136.99 | 22,193 | 0.0008 |
|  |  | splicing | 1,108 | 127.17 | 140,901 | 0.0052 |

**Table S10.** SNPs number, genetic diversity (π), heterozygosity and SNP density calculated based on LG and SG in Malayan and Chinese pangolin populations.

| Genome | Population | SNPs number | π | Het | SNP density | L*_AUTOSOME_* | Sample number |
| --- | --- | --- | --- | --- | --- | --- | --- |
|  |  |  |  |  | (Variants/KB) |  |  |
| MJ-SG | MjavA | 11,200,379 | 0.0007487 | 0.0008706 | 4.49 | 2,389,628,809 | 42 |
|  | MjavB | 29,638,177 | 0.0021221 | 0.0019443 | 11.88 |  | 30 |
|  | Total | 31,054,472 | 0.0016495 | 0.0013180 | 12.45 |  | 72 |
| MJ-LG | MjavA | 10,243,009 | 0.0006801 | 0.0006291 | 4.30 | 2,379,205,476 | 42 |
|  | MjavB | 31,894,738 | 0.0023651 | 0.0018918 | 13.38 |  | 30 |
|  | Total | 33,081,798 | 0.0017935 | 0.0011553 | 13.88 |  | 72 |
| MP-SG | MpenA | 19,045,653 | 0.0016639 | 0.0017153 | 7.45 | 2,375,278,212 | 11 |
|  | MpenB | 25,968,784 | 0.0019240 | 0.0018365 | 10.14 |  | 18 |
|  | MpenC | 15,523,320 | 0.0016665 | 0.0018285 | 6.07 |  | 8 |
|  | Total | 35,023,399 | 0.0024927 | 0.0017988 | 13.68 |  | 37 |
| MP-LG | MpenA | 14,926,355 | 0.0016926 | 0.0015052 | 6.16 | 2,420,955,685 | 11 |
|  | MpenB | 23,943,542 | 0.0020058 | 0.0016544 | 9.87 |  | 18 |
|  | MpenC | 19,288,330 | 0.0017774 | 0.0017467 | 7.95 |  | 8 |
|  | Total | 36,850,227 | 0.0026030 | 0.0016300 | 15.19 |  | 37 |

L*_AUTOSOME_*: The total length of autosomes.

**Table S11.** The comparison of the SG and LG for estimating ROH in Malayan pangolin and Chinese pangolin populations.

**See the attached Excel file.**

**Table S12.** The count and length of ROH fragments in the five populations of Malayan pangolin and Chinese pangolin.

**See the attached Excel file.**

**Table S13.** The F_ROH_ in the five populations of Malayan pangolin and Chinese pangolin.

**See the attached Excel file.**

**Table S14.** The total count of dnsSNPs, LOF, missense and synonymous SNPs in various populations, along with the average number of dnsSNPs, LOF, missense, and synonymous SNPs at the individual level, across different populations of the Malayan and Chinese pangolins.

| Item | MP | MpenA | MpenB | MpenC | MJ | MjavA | MjavB | level |
| --- | --- | --- | --- | --- | --- | --- | --- | --- |
| All dnsSNP | 10,173 | 3513 | 6329 | 4417 | 8620 | 2730 | 7742 | population |
| Derived dnsSNP | 7946 | 2325 | 4691 | 2860 | 6781 | 1524 | 5952 |  |
| All LOF | 3001 | 1019 | 1834 | 1279 | 2432 | 735 | 2159 |  |
| Derived LOF | 2455 | 758 | 1471 | 946 | 2030 | 484 | 1759 |  |
| All missense mutations | 151,589 | 53,903 | 95,331 | 67,722 | 137,966 | 42,559 | 125,991 |  |
| derived missense mutations | 116,958 | 35,049 | 69,432 | 43,543 | 106,412 | 22,127 | 95,182 |  |
| All synonymous SNPs | 165,730 | 57705 | 109,844 | 76,556 | 174,664 | 60,698 | 159,932 |  |
| Derived synonymous SNPs | 137,169 | 47051 | 92,265 | 54,246 | 148,994 | 36798 | 135,484 |  |
| All dnsSNP | 1607.19 | 1315.40 | 1795.72 | 1547.75 | 1407.82 | 1399.00 | 1420.17 | individual |
| Derived dnsSNP | 769.59 | 626.45 | 870.78 | 738.75 | 458.92 | 369.83 | 583.63 |  |
| All LOF | 421.16 | 342.64 | 383.44 | 614.00 | 338.63 | 327.95 | 353.57 |  |
| Derived LOF | 265.54 | 214.45 | 243.28 | 385.88 | 152.06 | 107.07 | 215.03 |  |
| All missense mutations | 25225.70 | 21656.27 | 22350.78 | 36602.25 | 22842.01 | 22320.69 | 23571.87 |  |
| derived missense mutations | 11992.46 | 9982.00 | 10453.17 | 18220.25 | 7064.50 | 5149.45 | 9745.57 |  |
| All synonymous SNPs | 30106.54 | 25203.27 | 26507.28 | 44946.88 | 30868.82 | 29808.95 | 32352.63 |  |
| Derived synonymous SNPs | 21157.43 | 18452.09 | 20398.72 | 26584.38 | 13372.42 | 7141.10 | 22096.27 |  |

**Table S15.** The individual level mutational load estimates in all Chinese pangolin and Malayan pangolin populations.

**See the attached Excel file.**

**Table S16.** Comparison of genome-wide nucleotide diversity (π) of the Malayan pangolin and Chinese pangolin with reference to other endangered species on the IUCN Red List.

| Species | π | Conservation status | Ref |
| --- | --- | --- | --- |
| CMA: Brown eared pheasant  (*Crossoptilon mantchuricum*) | 0.00009 | VU | [13] |
| ASI: Chinese alligator  (*Alligator sinensis*) | 0.00015 | CR | [14] |
| PTA: Amur tiger  (*Panthera tigris altaica*) | 0.00081 | CR | [15] |
| AFU: Red panda  (*Ailurus fulgens*) | 0.00070 | EN | [16] |
| AME: Giant panda  (*Ailuropoda melanoleuca*) | 0.00121 | VU | [17] |
| MJ: Malayan pangolin  (*Manis javanica*) | 0.00179 | CR | This study |
| MP: Chinese pangolin  (*Manis pentadactyla*) | 0.00260 | CR | This study |
| MBE: Dwarf musk deer  (*Moschus berezovskii*) | 0.00201 | EN | [18] |

**Table S17.** Quality assessment of the MP and MJ genomes by the Merqury software.

| Genome | QV | Error rate | Completeness (%) |
| --- | --- | --- | --- |
| MP | 57.06 | 1.96818e-06 | 96.81 |
| MJ | 55.08 | 3.10353e-06 | 95.40 |

**Table S18.** The mapping rates of four types of sequencing data to genomes assembled in this study.

| Genome | DNBSEQ short reads | Hi-C short reads | RNA data | Pacbio HiFi reads |
| --- | --- | --- | --- | --- |
| MJ | 99.91% | 99.40% | 99.77% | 99.99% |
| MJH1 | 99.80% | 99.35% | 99.72% | 99.98% |
| MJH2 | 98.24% | 98.45% | 98.15% | 99.87% |
| MP | 99.93% | 99.43% | 99.65% | 100.00% |
| MPH1 | 97.30% | 97.95% | 97.13% | 99.75% |
| MPH2 | 99.88% | 99.41% | 99.63% | 100.00% |

**Table S19.** Comparison of the assembly statistics with the previously published Chinese pangolin and Malayan pangolin genomes.

| Species | Assembly Level | Parameters | mManpen9.1 | M_pentadactyla-1.1.1 | YNU_ManPten_2.0 | ASM2424420v1 | M_pentadactyla-1.1.1_HiC |
| --- | --- | --- | --- | --- | --- | --- | --- |
| Chinese pangolin | Scaffold | Maximal length (bp) | 234,250,095 | 1,317,973 | 49,856,536 | - | 195,883,037 |
|  |  | N50 (bp) | 140,708,684 | 117,920 | 7,836,868 | - | 111,940,472 |
|  |  | number>=0bp | 89 | 92,772 | 4,694 | - | 71,390 |
|  |  | number>=2kb | 89 | 34,791 | 2,749 | - | 12,179 |
|  |  | Genome size (bp) | 2,641,556,940 | 2,204,732,179 | 2,399,660,488 | - | 2,215,491,158 |
|  | Contig | Maximal length (bp) | 209,591,311 | 292,755 | 1,273,659 | 53,155,020 | 292,755 |
|  |  | N50 (bp) | 56,151,610 | 20,802 | 133,879 | 13,973,343 | 20,726 |
|  |  | Genome size (bp) | 2,641,351,941 | 1,998,547,014 | 2,292,422,457 | 2,536,903,991 | 1,998,530,739 |
| Species | Assembly Level | Parameters | mManJav1.1 | ManJav1.0 | YNU_ManJav_2.0 | ASM2460508v1 | ManJav1.0_HiC |
| Malayan pangolin | Scaffold | Maximal length (bp) | 241,980,946 | 2,314,370 | 60,166,012 | - | 216,609,843 |
|  |  | N50 (bp) | 141,798,549 | 204,728 | 13,854,118 | - | 131,848799 |
|  |  | number>=0bp | 62 | 80,670 | 17,969 | - | 64,789 |
|  |  | number>=2kb | 62 | 50,580 | 4,155 | - | 34,149 |
|  |  | Genome size (bp) | 2,560,052,348 | 2,547,395,906 | 2,438,977,774 | - | 2,555,637,078 |
|  | Contig | Maximal length (bp) | 166,563,371 | 225,448 | 888,417 | 55,857,980 | 225,448 |
|  |  | N50 (bp) | 46,223,514 | 16,374 | 73,807 | 15,813,259 | 16,350 |
|  |  | Genome size (bp) | 2,559,088,858 | 2,348,532,481 | 2,238,249,616 | 2,440679,864 | 2,348,512,846 |

**Table S20.** Pairwise differences observed in comparisons between the haplotype genomes of Chinese and Malayan pangolin, the sliding window was set to be 100bp.

| Differences per 100bp | Chinese pangolin | Malayan pangolin |
| --- | --- | --- |
|  | Count | Count |
| 1 | 20,625,760 | 20,874,962 |
| 2 | 66,255 | 168,110 |
| 3 | 10,494 | 22,488 |
| 4 | 4303 | 8276 |
| 5 | 2471 | 5221 |
| 6 | 1462 | 3698 |
| 7 | 1021 | 2904 |
| 8 | 625 | 2163 |
| 9 | 421 | 1691 |
| 10 | 230 | 1296 |
| 11 | 136 | 913 |
| 12 | 100 | 629 |
| 13 | 58 | 396 |
| 14 | 35 | 245 |
| 15 | 24 | 162 |
| 16 | 17 | 90 |
| 17 | 9 | 45 |

**Table S21**. Chromosomal structural variants (>50bp) of MJH1 and MJH2, and of MPH1 and MPH2.

**See the attached Excel file.**

**Table S22.** Genes distributed in the structural variants of MJ and MP genomes.

| Species | Item | deletion | translocation | duplication | inversion |
| --- | --- | --- | --- | --- | --- |
| Malayan pangolin | Number of genes on structural variation | 248 | 366 | 1483 | 249 |
|  | Number of genes on breakpoints | 248 | 339 | 1073 | 236 |
|  | Number of pseudogenes at breakpoints | 0 | 1 | 3 | 0 |
| Chinese pangolin | Number of genes on structural variation | 95 | 481 | 2535 | 2228 |
|  | Number of genes on breakpoints | 95 | 443 | 2224 | 166 |
|  | Number of pseudogenes at breakpoints | 1 | 2 | 16 | 1 |

**Table S23.** The KEGG enrichment result of genes distributed in the structural variants of MJ/MP.

**See the attached Excel file.**

**Table S24.** The GO enrichment result of genes distributed in the structural variants of MJ/MP.

**See the attached Excel file.**

**Table S25.** Functional description of pseudogenes interrupted by structural variants.

| Species | Gene Name | Source | Homologous Gene | Function |
| --- | --- | --- | --- | --- |
| Malayan pangolin | MJAV01732 | dup | *MRPL13* | *MRPL13* acts as a novel therapeutic target and could promote cell proliferation in non-small cell lung cancer.[19] |
|  | MJAV03320 | dup | *PYCR2* | Loss of *PYCR2* causes neurodegeneration by increasing cerebral Glycine levels via *SHMT2*.[20] |
|  | MJAV06611 | dup | *ZNF337* | Up-regulation of *ZNF337* in rectum adenocarcinoma associated with radiotherapy response and involved in multiple process of cellular biology in tumor. [21] |
|  | MJAV06561 | trans | *IGL1* | *IGL1* has been shown to have both hemolytic and cytotoxic activities that reside in the C-terminus of the protein. [22] |
| Chinese pangolin | MPEN04793 | del | *TOLLIP* | *TOLLIP* promotes hepatocellular carcinoma progression via PI3K/AKT pathway. [23] |
|  | MPEN00748 | dup | *TPO* | *TPO* knockout in zebrafish partially recapitulates clinical manifestations of congenital hypothyroidism and reveals the involvement of TH in proper development of glucose homeostasis.[24] |
|  | MPEN00785 | dup | *KLHL29* | *KLHL29* closely correlated to T cells and M2 macrophages. [25] |
|  | MPEN00790 | dup | *FAM228B* | / |
|  | MPEN01251 | dup | *PXDN* | Biallelic deletion of *PXDN* in mice leads to anophthalmia and severe eye malformation.[10] |
|  | MPEN01258 | dup | *MBOAT2* | Identification of *MBOAT2* as an unfavorable biomarker correlated with KRAS activation and reduced CD8+ T-Cell Infiltration in pancreatic cancer.[26] |
|  | MPEN01259 | dup | *NA* | / |
|  | MPEN01273 | dup | *NBAS* | Involved in Golgi-to-endoplasmic reticulum (ER) retrograde transport upstream of the degranulation pathway.[27] |
|  | MPEN01276 | dup | *SMC6* | siRNA depletion of genes involved in the *SMC5-6* complex results in sensitivity to some DNA damaging agents.[28] |
|  | MPEN01285 | dup | *PUM2* | *PUM2* promotes growth of mature neurons / a gene trap mutation of a murine homolog of the drosophila stem cell factor *Pumilio* results in smaller testes but does not affect litter size or fertility. [29] |
|  | MPEN04191 | dup | *RTN3* | Reticulon 3-mediated Chk2/p53 activation suppresses hepatocellular carcinogenesis and is blocked by hepatitis B virus / Reticulon3 expression in rat optic and olfactory systems.[30] |
|  | MPEN04269 | dup | *GRK2* | Reduction of sympathetic activity via adrenal-targeted *GRK2* gene deletion attenuates heart failure progression and improves cardiac function after myocardial infarction. [31] |
|  | MPEN04319 | dup | *CD81* | *CD81* contributes to tumor growth and metastasis.[32] |
|  | MPEN09991 | dup | *OSGEP* | tRNA N6-adenosine threonylcarbamoyltransferase defect due to *KAE1/TCS3* (*OSGEP*) mutation manifest by neurodegeneration and renal tubulopathy.[33] |
|  | MPEN15189 | dup | *FKBP5* | Expression and regulation of the Fkbp5 gene in the adult mouse brain.[34] |
|  | MPEN15330 | dup | *SKIV2L* | The mammalian SKIV2L RNA exosome is essential for early B cell development.[35] |
|  | MPEN17278 | dup | *CYP3A29* | Xenobiotic metabolism.[36] |
|  | MPEN01257 | inv | *KIDINS220* | Regulates adipocyte differentiation.[37] |
|  | MPEN00758 | trans | *ASAP2* | Arsenic sulfide inhibits the progression of gastric cancer through regulating the circRNA_ASAP2/Wnt/β-catenin pathway. [38] |
|  | MPEN01287 | trans | *LDAH* | Regulate lipophagy and cholesterol efflux in macrophage foam cells.[39] |

**Reference**

1. Qi W, Lim YW, Patrignani A, Schlapfer P, Bratus-Neuenschwander A, Gruter S, et al. The haplotype-resolved chromosome pairs of a heterozygous diploid African cassava cultivar reveal novel pan-genome and allele-specific transcriptome features. Gigascience. 2022;11:giac028. doi:10.1093/gigascience/giac028.

2. Yan D, Luo X, Tang J, Xu S, Huang K, Wang X, et al. High-Quality Genomes of Pangolins: Insights into the Molecular Basis of Scale Formation and Adaption to Myrmecophagous Diet. Mol Biol Evol. 2023;40 1 doi:10.1093/molbev/msac262.

3. Heighton SP, Allio R, Murienne J, Salmona J, Meng H, Scornavacca C, et al. Pangolin genomes offer key insights and resources for the world's most trafficked wild mammals. bioRxiv. 2023; doi:10.1101/2023.02.16.528682.

4. Damas J, Corbo M, Kim J, Turner-Maier J, Farré M, Larkin DM, et al. Evolution of the ancestral mammalian karyotype and syntenic regions. Proceedings of the National Academy of Sciences. 2022;119 40:e2209139119.

5. Cao P, Dai Q, Deng C, Zhao X, Qin S, Yang J, et al. Genome-wide signatures of mammalian skin covering evolution. Science China Life Sciences. 2021;64 10:1765-80.

6. Hu JY, Hao ZQ, Frantz L, Wu SF, Chen W, Jiang YF, et al. Genomic consequences of population decline in critically endangered pangolins and their demographic histories. Natl Sci Rev. 2020;7 4:798-814. doi:10.1093/nsr/nwaa031.

7. Choo SW, Rayko M, Tan TK, Hari R, Komissarov A, Wee WY, et al. Pangolin genomes and the evolution of mammalian scales and immunity. Genome research. 2016;26 10:1312-22.

8. Low WY, Tearle R, Liu R, Koren S, Rhie A, Bickhart DM, et al. Haplotype-resolved genomes provide insights into structural variation and gene content in Angus and Brahman cattle. Nat Commun. 2020;11 1:2071. doi:10.1038/s41467-020-15848-y.

9. Sun H, Jiao WB, Krause K, Campoy JA, Goel M, Folz-Donahue K, et al. Chromosome-scale and haplotype-resolved genome assembly of a tetraploid potato cultivar. Nat Genet. 2022;54 3:342-8. doi:10.1038/s41588-022-01015-0.

10. Kim H-K, Ham KA, Lee S-W, Choi HS, Kim H-S, Kim HK, et al. Biallelic deletion of pxdn in mice leads to anophthalmia and severe eye malformation. International Journal of Molecular Sciences. 2019;20 24:6144.

11. Segarra NG, Ballhausen D, Crawford H, Perreau M, Campos-Xavier B, van Spaendonck-Zwarts K, et al. NBAS mutations cause a multisystem disorder involving bone, connective tissue, liver, immune system, and retina. American Journal of Medical Genetics Part A. 2015;167 12:2902-12. doi:10.1002/ajmg.a.37338.

12. Kumamaru E, Kuo C-H, Fujimoto T, Kohama K, Zeng L-H, Taira E, et al. Reticulon3 expression in rat optic and olfactory systems. Neuroscience Letters. 2004;356 1:17-20. doi:10.1016/j.neulet.2003.11.009.

13. Wang P, Burley JT, Liu Y, Chang J, Chen D, Lu Q, et al. Genomic consequences of long-term population decline in brown eared pheasant. Molecular Biology and Evolution. 2021;38 1:263-73.

14. Yang S, Lan T, Zhang Y, Wang Q, Li H, Dussex N, et al. Genomic investigation of the Chinese alligator reveals wild‐extinct genetic diversity and genomic consequences of their continuous decline. Molecular Ecology Resources. 2023;23 1:294-311.

15. Lan T, Li H, Zhang L, Shi M, Liu B, Cui L, et al. Population genomics reveals extensive inbreeding and purging of mutational load in wild Amur tigers. bioRxiv. 2023:2023.05. 09.539923.

16. Hu Y, Thapa A, Fan H, Ma T and Wei F. Genomic evidence for two phylogenetic species and long-term population bottlenecks in red pandas. Science advances. 6 9:eaax5751.

17. Guang X, Lan T, Wan Q-H, Huang Y, Li H, Zhang M, et al. Chromosome-scale genomes provide new insights into subspecies divergence and evolutionary characteristics of the giant panda. Science Bulletin. 2021;66 19:2002-13. doi:<https://doi.org/10.1016/j.scib.2021.02.002>.

18. Liu G, Zhang BF, Chang J, Hu XL, Li C, Xu TT, et al. Population genomics reveals moderate genetic differentiation between populations of endangered Forest Musk Deer located in Shaanxi and Sichuan. BMC Genomics. 2022;23 1:1-11.

19. Jing C, Fu R, Wang C, Li X and Zhang W. MRPL13 Act as a Novel Therapeutic Target and Could Promote Cell Proliferation in Non-Small Cell Lung Cancer. Cancer Management and Research. 2021:5535-45.

20. Escande-Beillard N, Loh A, Saleem SN, Kanata K, Hashimoto Y, Altunoglu U, et al. Loss of PYCR2 causes neurodegeneration by increasing cerebral glycine levels via SHMT2. Neuron. 2020;107 1:82-94. e6.

21. Zhao P, Zhen H, Zhao H, Huang Y and Cao B. Identification of hub genes and potential molecular mechanisms related to radiotherapy sensitivity in rectal cancer based on multiple datasets. Journal of Translational Medicine. 2023;21 1:1-16.

22. Kato K and Tachibana H. Identification of multiple domains of Entamoeba histolytica intermediate subunit lectin-1 with hemolytic and cytotoxic activities. International Journal of Molecular Sciences. 2022;23 14:7700.

23. Huang L, Yang Q, Chen H, Wang Z, Liu Q and Ai S. Tollip promotes hepatocellular carcinoma progression via PI3K/AKT pathway. Open Medicine. 2022;17 1:626-37.

24. Fang Y, Wan J-P, Zhang R-J, Sun F, Yang L, Zhao S-X, et al. Tpo knockout in zebrafish partially recapitulates clinical manifestations of congenital hypothyroidism and reveals the involvement of TH in proper development of glucose homeostasis. General and Comparative Endocrinology. 2022;323:114033.

25. Wang X, Liu W, Li H, Ding J, Feng Y and Chen Z. Exploring the Role of Obesity in Dilated Cardiomyopathy Based on Bio-informatics Analysis. Journal of Cardiovascular Development and Disease. 2022;9 12:462.

26. Zhai X, Li XY, Wang YJ, Qin KR, Hu JR, Li MN, et al. Fancd2os Reduces Testosterone Production by Inhibiting Steroidogenic Enzymes and Promoting Cellular Apoptosis in Murine Testicular Leydig Cells. Endocrinol Metab (Seoul). 2022;37 3:533-46. doi:10.3803/EnM.2022.1431.

27. Bi X, Zhang Q, Chen L, Liu D, Li Y, Zhao X, et al. NBAS, a gene involved in cytotoxic degranulation, is recurrently mutated in pediatric hemophagocytic lymphohistiocytosis. Journal of Hematology & Oncology. 2022;15 1:1-5.

28. Ju L, Wing J, Taylor E, Brandt R, Slijepcevic P, Horsch M, et al. SMC6 is an essential gene in mice, but a hypomorphic mutant in the ATPase domain has a mild phenotype with a range of subtle abnormalities. DNA repair. 2013;12 5:356-66.

29. Xu EY, Chang R, Salmon NA and Reijo Pera RA. A gene trap mutation of a murine homolog of the Drosophila stem cell factor Pumilio results in smaller testes but does not affect litter size or fertility. Molecular reproduction and development. 2007;74 7:912-21.

30. Song S, Shi Y, Wu W, Wu H, Chang L, Peng P, et al. Reticulon 3-mediated Chk2/p53 activation suppresses hepatocellular carcinogenesis and is blocked by hepatitis B virus. Gut. 2021;70 11:2159-71.

31. Lymperopoulos A, Rengo G, Gao E, Ebert SN, Dorn GW and Koch WJ. Reduction of sympathetic activity via adrenal-targeted GRK2 gene deletion attenuates heart failure progression and improves cardiac function after myocardial infarction. Journal of Biological Chemistry. 2010;285 21:16378-86.

32. Vences-Catalán F, Duault C, Kuo C-C, Rajapaksa R, Levy R and Levy S. CD81 as a tumor target. Biochemical Society Transactions. 2017;45 2:531-5.

33. Edvardson S, Prunetti L, Arraf A, Haas D, Bacusmo JM, Hu JF, et al. tRNA N6-adenosine threonylcarbamoyltransferase defect due to KAE1/TCS3 (OSGEP) mutation manifest by neurodegeneration and renal tubulopathy. European Journal of Human Genetics. 2017;25 5:545-51.

34. Scharf SH, Liebl C, Binder EB, Schmidt MV and Müller MB. Expression and regulation of the Fkbp5 gene in the adult mouse brain. PloS one. 2011;6 2:e16883.

35. Yang K, Han J, Gill JG, Park JY, Sathe MN, Gattineni J, et al. The mammalian SKIV2L RNA exosome is essential for early B cell development. Science immunology. 2022;7 72:eabn2888.

36. Yao M, Dai M, Liu Z, Huang L, Chen D, Wang Y, et al. Comparison of the substrate kinetics of pig CYP3A29 with pig liver microsomes and human CYP3A4. Bioscience reports. 2011;31 3:211-20.

37. Zhang K, Sun W, Liu Y, Lv Y, Hou D, Lin Y, et al. SINO syndrome causative KIDINS220/ARMS gene regulates adipocyte differentiation. Frontiers in Cell and Developmental Biology. 2021;9:619475.

38. Hu J, Hu B, Deng L, Cheng L, Fan Q and Lu C. Arsenic sulfide inhibits the progression of gastric cancer through regulating the circRNA_ASAP2/Wnt/β-catenin pathway. Anti-Cancer Drugs. 2022;33 1:e711.

39. Robichaud S, Fairman G, Vijithakumar V, Mak E, Cook DP, Pelletier AR, et al. Identification of novel lipid droplet factors that regulate lipophagy and cholesterol efflux in macrophage foam cells. Autophagy. 2021;17 11:3671-89.
