# Supplementary material for: Enhancing inbreeding estimation and global conservation insights through chromosome-level assemblies of the Chinese and Malayan pangolin
Source: Gigascience. 2025 Feb 14;14:giaf003. doi: 10.1093/gigascience/giaf003 (PMC11825179; doi:10.1093/gigascience/giaf003)
Supplement: giaf003_GIGA-D-24-00182_Original_Submission [file giaf003_giga-d-24-00182_original_submission.pdf]

GigaScience

Enhancing inbreeding estimation and global conservation insights through HiFi assemblies of Chinese and Malayan pangolin

--Manuscript Draft--

**Manuscript Number:**	GIGA-D-24-00182
**Full Title:**	Enhancing inbreeding estimation and global conservation insights through HiFi assemblies of Chinese and Malayan pangolin
**Article Type:**	Research
**Funding Information:**	National Key Program of Research and Development, Ministry of Science and Technology (No. 2022YFF1301500) — Not applicable<br>the Guangdong Provincial Key Laboratory of Genome Read and Write (No. 2017B030301011) — Not applicable
**Abstract:**	A high-quality reference genome coupled with resequencing data is becoming a promising strategy to address issues in conservation genomics, which has greatly enhanced the development of conservation plans for endangered species. Pangolins are fascinating animals with a range of distinctive features, but unfortunately, they are the world's most trafficked wild animals. Here, we report a haplotype-resolved and chromosome-scale genome for each of the Chinese pangolin and Malayan pangolin, the most representative reference genome for pangolin species. We found a greater improvement in evaluation of genetic diversity and inbreeding based on high-quality genomes and obtained different results in detecting genome-wide extinction risks being compared with short read assembled genomes. Moderate inbreeding and genetic diversity were verified again in these two pangolin species except for one Malayan pangolin population with the high inbreeding and low genetic diversity, which we recommend to pay special attention to the conservation and protection of this population. Additionally, our study is the first to detect relative mild genetic purging in pangolin populations that were analyzed. These two high quality reference genomes will provide valuable genomic resource for future studies on the protection and conservation for pangolins.
**Corresponding Author:**	Haimeng Li<br>Northeast Forestry University<br>Harbin, CHINA
**Corresponding Author Secondary Information:**	
**Corresponding Author's Institution:**	Northeast Forestry University
**Corresponding Author's Secondary Institution:**	
**First Author:**	Tianming Lan
**First Author Secondary Information:**	
**Order of Authors:**	Tianming Lan
	Haimeng Li
	Minhui Shi
	Boyang Liu
	Sahu Sunil Kumar
	Qing Wang
	Jun Li
	Shangchen Yang

Powered by Editorial Manager® and ProduXion Manager® from Aries Systems Corporation

	Jin Chen
	Fanghui Hou
	Chuanling Yin
	Kai Wang
	Liangyu Cui
	Tengcheng Que
	Wenjian Liu
	Yinping Tian
	Huan Liu
	Yan Hua
**Order of Authors Secondary Information:**	
**Additional Information:**	
**Question**	**Response**
Are you submitting this manuscript to a special series or article collection?	No
**Experimental design and statistics**<br><br>Full details of the experimental design and statistical methods used should be given in the Methods section, as detailed in our Minimum Standards Reporting Checklist. Information essential to interpreting the data presented should be made available in the figure legends.<br><br>Have you included all the information requested in your manuscript?	Yes
**Resources**<br><br>A description of all resources used, including antibodies, cell lines, animals and software tools, with enough information to allow them to be uniquely identified, should be included in the Methods section. Authors are strongly encouraged to cite Research Resource Identifiers (RRIDs) for antibodies, model organisms and tools, where possible.<br><br>Have you included the information requested as detailed in our Minimum Standards Reporting Checklist?	Yes

Powered by Editorial Manager® and ProduXion Manager® from Aries Systems Corporation

**Availability of data and materials**<br><br>All datasets and code on which the conclusions of the paper rely must be either included in your submission or deposited in publicly available repositories (where available and ethically appropriate), referencing such data using a unique identifier in the references and in the "Availability of Data and Materials" section of your manuscript.<br><br>Have you have met the above requirement as detailed in our Minimum Standards Reporting Checklist?	Yes

Manuscript Click here to access/download;Manuscript;MainText.docx

Enhancing inbreeding estimation and global conservation insights through HiFi assemblies of Chinese and Malayan pangolin

Tianming Lan[1,2,3,†,*] , Haimeng Li[3,4,†] , Minhui Shi[3,†] , Boyang Liu[3] , Sahu Sunil Kumar[1] , Qing Wang[3] , Jun Li[2] , Shangchen Yang[5] , Jin Chen[3] , Fanghui Hou[6,7] , Chuanling Yin[3] , Kai Wang[2] , Liangyu Cui[3] , Tengcheng Que[8,9] , Wenjian Liu[8] , Yinping Tian[3] , Huan Liu[1] and Yan Hua[2,*]

[1] BGI Life Science Joint Research Center, Northeast Forestry University, Harbin 150040, China

[2] Guangdong Provincial Key Laboratory of Silviculture, Protection and Utilization, Guangdong Academy of Forestry, Guangzhou 510520, China

[3] College of Wildlife and Protected Area, Northeast Forestry University, Harbin 150040, China

[4] Heilongjiang Key Laboratory of Complex Traits and Protein Machines in Organisms, Harbin 150040, China

[5] College of Life Sciences, Zhejiang University, Hangzhou 310058, China

[6] Guangdong Wildlife Rescue Monitoring Center, Guangzhou 510520, China

[7] Pangolin Conservation Research Center of National Forestry and Grassland Administration, Guangzhou 510520, China

[8] Faculty of Data Science City University of Macau, Macau 999078, China

[9] Guangxi Zhuang Autonomous Terrestrial Wildlife Rescue Research and Epidemic Diseases Monitoring Center, Nanning 530025, China

*Correspondence address. Yan Hua, Guangdong Provincial Key Laboratory of Silviculture, Protection and Utilization, Guangdong Academy of Forestry, Guangzhou 510520 China. E-mail: wildlife530@hotmail.com; Tianming Lan, College of Wildlife and Protected Area, Northeast Forestry University, Harbin 150040, China. E-mail: lantianming1314@126.com

†Equal contributions.

Abstract

A high-quality reference genome coupled with resequencing data is becoming a promising strategy to address issues in conservation genomics, which has greatly enhanced the development of conservation plans for endangered species. Pangolins are fascinating animals with a range of distinctive features, but unfortunately, they are the world's most trafficked wild animals. Here, we report a haplotype-resolved and chromosome-scale genome for each of the Chinese pangolin and Malayan pangolin, the most representative reference genome for pangolin species. We found a greater improvement in evaluation of genetic diversity and inbreeding based on high-quality genomes and obtained different results in detecting genome-wide extinction risks being compared with short read assembled genomes. Moderate inbreeding and genetic diversity were verified again in these two pangolin species except for one Malayan pangolin population with the high inbreeding and low genetic diversity, which we recommend to pay special attention to the conservation and protection of this population. Additionally, our study is the first to detect relative mild genetic purging in pangolin populations that were analyzed. These two high quality reference genomes will provide valuable genomic resource for future studies on the protection and conservation for pangolins.

Keywords: Chinese pangolin; Malayan pangolin; inbreeding; genetic purging; conservation genomics

Introduction

From the Human Genome Project[1] to the first Telomere-to-Telomere genome[2], the human genome provides an excellent example of the evolution of a reference genome and how its continuous improvement has shaped significant advancements in biology, medicine, and other related fields[1-5]. The rapid development of third-generation sequencing technologies over the past decade has further revolutionized the quality of genome assembly. This has been accomplished by incorporating long-read (usually >10 kb) capable of spanning complex structures, like complex structure variants, telomers, segmental duplications, or centromere[2, 6-12]. Taking benefits from the decreasing sequencing cost, advanced computational power, and continuously improving assembly algorithms, a

large number of high-quality reference genomes have been assembled by long-read sequencing technologies have been released[13-17].

High-quality reference genomes enable comprehensive analyses of population genomics, and are promising to revolutionize conservation genomics[18]. However, fewer than 1% of threatened species listed on the IUCN red list have a reference genome[19], and this number will further decreases, if long-read assembly is considered. Against the backdrop of the shift from conservation genetics to conservation genomics[20], high-quality reference genomes play a critical role in providing the necessary support for the conservation of endangered species[18, 21]. In particular, the genetic rescue is considered as an important strategy to facilitate gene flow and avoid further inbreeding to increase population fitness [22, 23]. A deep understanding of genome-wide extinction risks and basic genetic background for a given small population is key for making evidence-based plans for genetic rescue. These include population structure, genomic diversity, inbreeding and inbreeding depression, local adaptation, genome-wide mutational load, and population demography [24-28]. A high-quality reference genome usually facilitates population-level studies that are closely related to conservation genomics[18]. For example, the evaluation of inbreeding by measuring runs of homozygosity (ROH) highly depends on the high-quality reference genome with outstanding contiguity. This is due to the ROH in small populations with high-level inbreeding often spanning over several millions of base pairs [28-30], and hardly be detected based on fragmented genomes assembled by short reads.

Pangolin is a prehistoric mammal with many unique biological characteristics[31, 32], such as overlapping keratin scales over the body, a highly specialized diet, a long and muscular tongue, a sensitive olfactory system, and burrowing ability [33, 34]. However, pangolin's scale and meat have been traditionally used for medicine and food by locals across its distribution areas [35]. The overexploitation of pangolins driven by the soaring demand for luxury food and traditional Chinese medicine, is pushing this animal to the edge of extinction[36-39]. At present, the pangolin has been the most heavily trafficked wild animal globally, with more than 900,000 individuals poached over the last two decades and 67 countries from six continents involved in illegal poaching and trade[40]. For better protection, all eight pangolin species have been uplisted in Appendix I of the Convention of International Trade of Endangered Species of Wild Fauna and Flora (CITES) since 2016. Poaching is more rampant for Asian pangolins than for African pangolins, especially the Malayan pangolin (*Manis javanica*, hereafter MJ) and Chinese pangolin (*Manis pentadactyla*, hereafter MP), which are under extreme survival pressure due to a great amount of poaching and trafficking [25]. These two species have been listed as "Critically Endangered" on the International Union for Conservation of Nature (IUCN) Red List since 2014.

Previous studies identified two Malayan populations[25] (hereafter MJ1 and MJ2) and three Chinese pangolin populations[25, 41] (hereafter CPA, CPB and CPC). The MJ1 population was identified from the mainland (China and Myanmar) and diverged from the MJ1 population, which possibly originated from Southeast Asian islands. Among the three Chinese pangolin populations, the CPA population is a newly discovered population distributed in Guangdong, China; the CPB population is distributed in a vast area, including southern China and Thailand; and the CPC population highly diverged from the other two populations, which are very likely to have originated from Myanmar (Fig. 1A, Supplementary Table S1 and S2). The highly divergent pangolin populations indicate that deep isolation may have occurred, which is usually detrimental to the survival of endangered species.

The recently introduced Pacific Biosciences (PacBio) high-fidelity (HiFi) sequencing technology combined with a HiFi-specific assembler can generate high-quality and haplotype-resolved *de novo* assemblies, representing one of the most promising strategies for genome assembling [42]. This may further facilitate more comprehensive and accurate evaluations of genome-wide genetic risks, specifically for the evaluation of inbreeding. Given the urgent

need for the genomic background to support their conservation, several reference genomes of pangolins have been assembled, annotated, and published[25, 33, 43-46]. However, high-quality reference genomes assembled from HiFi long-reads are non-existent for pangolins. Here we report two chromosome-scale and haplotype-resolved genomes of the Malayan and Chinese pangolin. These genomes are the highest-quality and the most representative reference genomes for pangolin species to date. Based on the superiority of high-quality reference genomes in population genomics, we systematically investigated genomic backgrounds and evaluated genome-wide extinction risks for the five pangolin populations.

Results

The first haplotype-resolved genome assembly for the pangolin

To obtain high-quality reference genomes for both the Malayan pangolin and Chinese pangolin, we combined PacBio HiFi long reads, Hi-C reads and DNBSEQ short reads for the genome assembly (Fig. 1B). We first generated phased contigs for both species (Table 1), and the haploid-resolved contigs were further linked at chromosome-level by combining HiFi long reads and Hi-C reads. We finally assembled 19 and 20 chromosome-scale pseudomolecules for MJ and MP, respectively (Fig. 1C, Supplementary Fig. S1). This finding is consistent with the karyotypic analysis, even though the karyotype in pangolins is variable[47]. The diploid genome sizes we assembled for MJ and MP were ~2.56 Gb and ~2.64 Gb, representing 99.98% and 96.01% of the estimated genome size (~2.56 Gb for MJ and ~2.75 Gb for MP), respectively (Supplementary Fig S2). The contig and scaffold NG50 of the MJ/MP were 61.24 Mb/39.47 Mb and 141.80 Mb/140.53 Mb, respectively (Table 1, Supplementary Table S3) . Furthermore, we identified the X-chromosome and Y-linked regions in both genomes (Supplementary Fig. S3).

Table 1: Summary Statistics for the Genome Sequences

Item	Category	Chinese pangolin (MP)	Malayan pangolin (MJ)
**Sequencing Data**	WGS (Gb)	254.05	245.34
	HiFi (Gb)	65.81	95.99
	Hi-C (Gb)	239.77	227.48
	RNA-seq (Gb)	6.21	6.35
**Genome Assemblies**	Estimated genome size (Gb)	2.75	2.56
	Assembled genome size (Gb)	2.64	2.56
	Contig number	294	154
	Contigs N50 (Mb)	40.35	61.24
	Longest contig (Mb)	184.35	123.33
	Scaffold number	89	62
	Scaffolds N50 (Mb)	140.71	141.8
	Longest scaffold (Mb)	234.25	241.98
	Pseudo-chromosomes Number	20	19
	Percentage of Pseudo-chromosomes to the assembly	96.97%	98.48%
**Assembly Quality**	Assembly error rate	1.09E-05	1.39E-05
	QV score for bases in the assembly	49.63	48.56
	Repeat content	41.62%	37.62%
	BUSCO assessment	98.00%	97.50%

The hifiasm assembler simultaneously resulted in two groups of haplotigs for each of the MJ (hereafter MJH1, MJH2) and MP (hereafter MPH1, MPH2) genome (Supplementary Table S4 and S5). Merqury k-mer analysis indicated high completeness and a low level of artificial duplication for haplotype-resolved genomes of both MJ and MP (Supplementary Fig. S4). The k-mer spectra plot showed that homozygous regions (shared k-mers) and heterozygous

regions (haploid specific k-mers) mainly consisted of two-copies (~82X/78X) and one-copy (~ 40X/38X) k-mers (Supplementary Fig. S4). Further, the sequencing depths of the two groups of haplotigs were consistent for both genomes evaluated by PacBio HiFi reads and DNBSEQ reads (Supplementary Fig. S5), implying the completeness of haplotype-resolved assemblies. As expected, the k-mer completeness for all haploid and diploid genomes attained a very high level (Supplementary Table S6).

The base-level quality evaluation showed that all genomes (2 diploid assemblies and 4 haploid assemblies) had high assembly accuracy with the lowest QV score of 48.33, corresponding to an accuracy higher than 99.99% (Supplementary Table S6). Besides, all these assemblies showed high BUSCO scores, with 97.5% and 98.0% BUSCO genes identified in the MJ and MP diploid genomes, respectively. For haploid genomes, the BUSCO score was still as high as 94.8 % even for the lowest MPH1 (Supplementary Table S7). The genome mapping of DNBSEQ short reads, Hi-C short reads, and RNA sequencing data to genome assemblies also presented high mapping rates, especially DNBSEQ short reads, with the mapping rate of 99.91% and 99.93% for MJ and MP genome, respectively (Supplementary Table S8). Finally, we assessed the structural-level accuracy by mapping long PacBio HiFi reads to the haploid genomes and found more than 99.24%/99.27% and 98.61%/97.38% of MJ/MP genomes were identified to be correctly assembled with a criterion of ≥10 X long reads mapping at a single position[48].

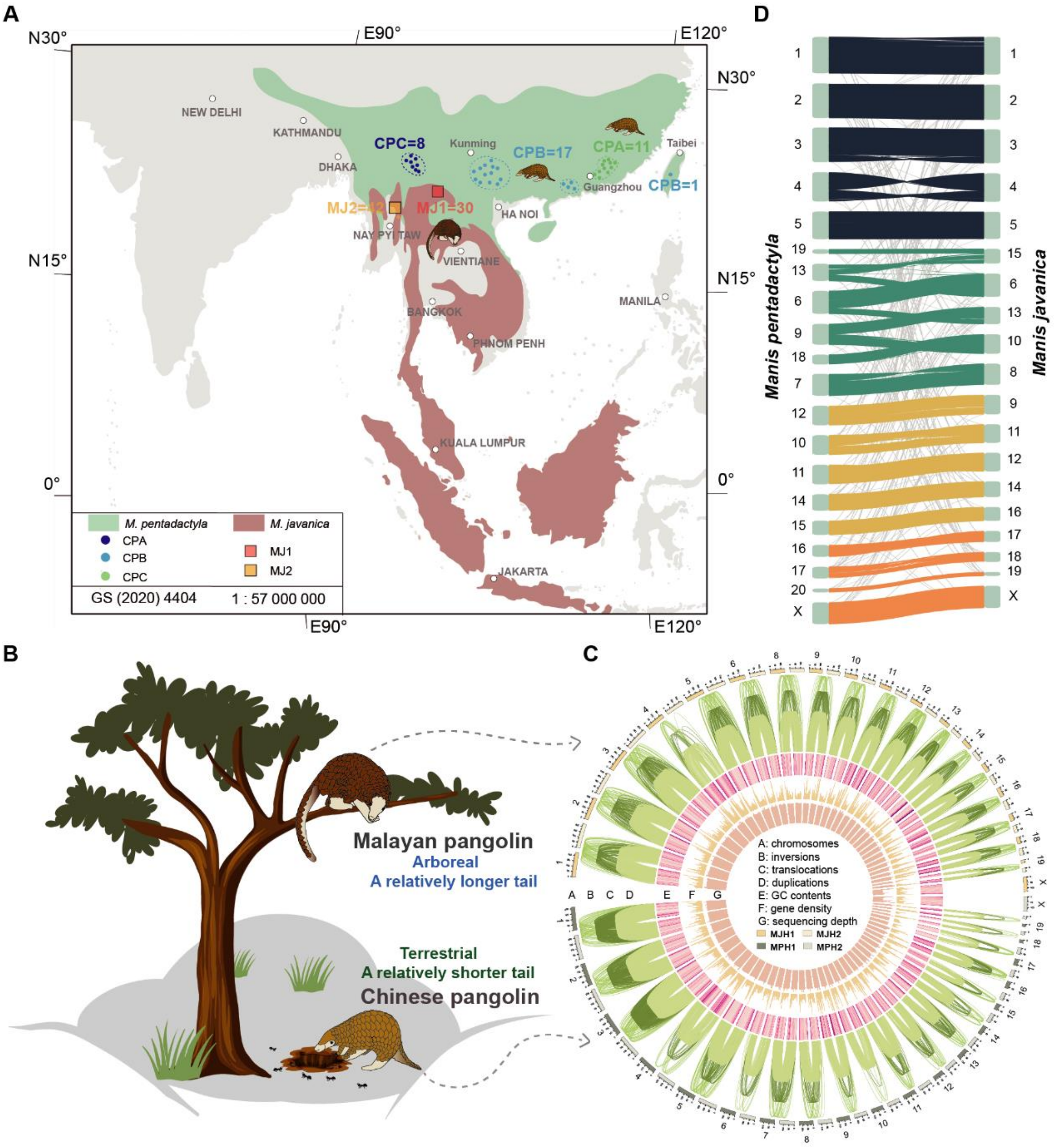

Figure 1: Genomic landscape of the Malayan pangolin and Chinese pangolin genomes. (A) The distribution area and sampling sites of Chinese and Malayan pangolins in this study. The circles represent sampling sites of Chinese pangolins reported in Wang *et al*,.[41], and samples without detailed locations were not showed on this map. (B) The Malayan pangolin and Chinese pangolin with their species-specific biological characteristics. (C) Brief introduction and circos diagram of the two pangolin genomes. (D) The chromosome-scale synteny analysis between the Malayan pangolin and Chinese pangolin genomes.

We also found high collinearity between the MP and MJ genomes, with 4 fissions and 3 fusions in the MJ genome when compared to the MP genome (Fig. 1D), which was consistent with previous karyotypic analysis[47], showing that these two genomes were accurately assembled at the chromosome-level. Overall, we are confident that we provided two new, high-quality, and representative reference genomes for pangolins.

Genome annotation

The total length of the repeat elements reached 1,260.92 Mb and 1,332.41 Mb, accounting for 49.25% and 50.44% of MJ and MP genomes, respectively (Supplementary Table S9). The composition of repeats in the MJ and MP genomes was similar, with the most abundant repeat element being LINE (MJ: 35.41%, MP: 37.36%), followed by

LTR (MJ: 11.14%, MP: 13.61%), DNA element (MJ: 2.23%, MP: 2.11%) and SINE (MJ: 0.96%, MP: 0.91%) (Supplementary Table S10). We predicted 19,680 and 19,886 gene models in the MJ and MP genomes, respectively (Supplementary Table S11). The gene regions spanned over 853.90 Mb and 791.49 Mb, composing 33.36% and 29.96% of the MJ and MP genomes, respectively (Supplementary Table S11). The average gene length, exon length, and intron length were 43.39 kb, 175.75 bp and 4.92 kb for the MJ genome, and these three parameters were 39.80 kb, 175.07 bp and 4.64 kb for the MP genome (Supplementary Table S11). The BUSCO analysis showed high completeness for gene sets of both genomes, with the lowest BUSCO score higher than 96% (Supplementary Table S12). Overall, 19,575 (99.47%) and 19,792 (99.53%) genes were functionally annotated respectively in MJ and MP genomes, respectively (Supplementary Table S13). In addition, we predicted 288/435 rRNA, 1,296/1,348 miRNA, 806/350 tRNA, and 1,521/1,412 snRNA in the MJ/MP genomes (Supplementary Table S14).

Comparisons of the genomic landscape between haploid chromosomes

In general, the two haploid genomes of MJ or MP were found to be very similar. The sequence differences (100bp window) showed one peak in the histogram for both MJ and MP genomes, indicating that the proportion of identical sequences between haploid genomes were dominant (Supplementary Table S15, Supplementary Fig. S6). Synteny analysis showed clear one-to-one syntenic blocks between homologous haploid chromosome pairs of the two pangolin species (Supplementary Fig. S7 and S8), further showing the high similarity between haploid genomes, which was also reflected in the k-mer analysis with almost all k-mers were shared between haploid genomes (Supplementary Fig. S4).

Nonetheless, we detected many chromosomal SVs (>50bp) between MJH1 and MJH2, and between MPH1 and MPH2. In general, we found 5,233 (3,681 duplications, 534 translocations, 509 inversions and 509 deletions) and 8,957 (7,738 duplications, 760 translocations, 269 inversions and 190 deletions) SVs between the haploid genomes of MJ and MP (Fig. 1C, Supplementary Table S16). All these SVs were validated by our assembled contigs (Supplementary Fig. S9). We found 2,136 genes that were distributed in the SVs of the MJ genome, while 4,264 such genes were found in the MP genome (Supplementary Fig. S10-S13, Supplementary Table S17-S19). It is worth noting that four genes were disrupted by breakpoints of SVs and had become pseudogenic in one of the two haploid chromosomes for the MJ, and we further found 20 such genes in the MP genome. Interestingly, several of these genes in the MP genome were vision-related, such as *PXDN*, *NBAS* and *RTN3*. *PXDN* gene is closely related to eye development, and the loss of *PXDN* gene in mice results in severe eye disorders, including drastically disorganized eye structures and the absence of eyeballs[49]. *NBAS* gene is estimated to correlate with retinal homeostasis[50]. *RTN3* gene is likely to play an important role in retinal function, as this gene's mutation causes retinal dystrophies[51]. Some of the other genes were found to be related to immunity and metabolism (Supplementary Table S20).

The HiFi genome improves the evaluation of genetic diversity and inbreeding

We compared the short-read assembled genome and long-read assembled genome (PacBio HiFi in this study) for the evaluation of commonly used genetic parameters in population genomics, particularly in conservation genomics, including population structure, population history and separation, genetic diversity, and inbreeding (Supplementary Table S21). In this comparison, we found that the population structure (PCA, phylogenetic tree and admixture), population history, and population separation (inferred by MSMC2) were almost not affected by the quality of the reference genome (Supplementary Fig. S14-S18). For genetic diversity, the difference between π values calculated by LG and by SG was not large, but this difference is significant with higher π values calculated based on the LG (Fig. 2A). It is undeniable that the LG could reflect much more detailed information on genetic diversity than SG (Supplementary Table S21). Furthermore, by screening ROH across the genome to evaluate the inbreeding, we found more significant differences between SG and LG. The ROH is an important genetic factor that can reflect the inbreeding level in a population, while it was very sensitive to the quality of the reference genome. For Malayan

pangolin populations, we found that F_{ROH} calculated by LG were obviously higher than those calculated by SG (Fig. 2B, Supplementary Table S22-S26). However, for the Chinese pangolin, this difference was found to be significant only for ROHs larger than 1 Mb, although F_{ROH} were larger for the LG under other conditions (Fig. 2C).

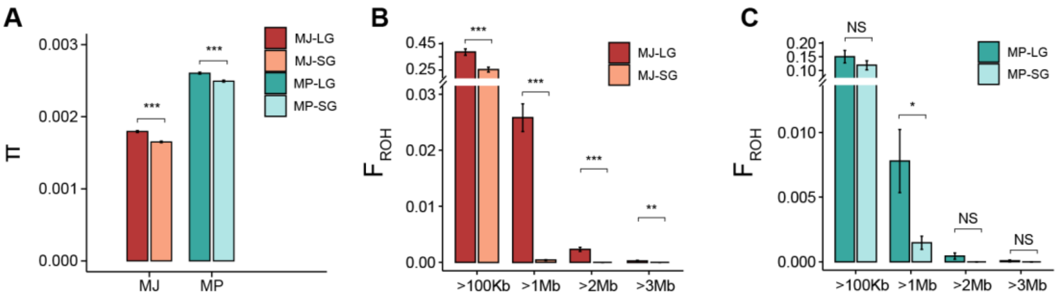

Figure 2: Comparisons of genome-wide genetic diversity and inbreeding estimated based on LG (long-read HiFi assembled genome) and SG (short-read assembled genome). (A) The comparison of genome-wide π calculated based on the LG and SG in Chinese and Malayan pangolin genomes. (B) Comparison of F_{ROH} calculated based on LG and SG in the Malayan pangolin genomes. (C) Comparison of F_{ROH} calculated based on LG and SG in Chinese pangolin genomes. Note: NS: $p \geq 0.5$, *$p < 0.05$, **$p < 0.01$, ***$p < 0.001$.

Genome-wide genetic diversity and inbreeding

Considering the improvement in evaluating the genetic diversity and inbreeding based on the high-quality HIFI genomes, we performed the reassessment and found the genome-wide genetic diversity (π) of the Chinese pangolin and Malayan pangolin, based on the HiFi genome were 0.0026 and 0.0018, respectively, which were both higher than those calculated based on the SG (π_{MP}=0.0025, π_{MJ}=0.0016) (Supplementary Table S21). For Chinese pangolin populations, the CPB harbored the highest genetic diversity (π_{CPB} = 0.0020), followed by the CPC (π_{CPC} = 0.0018) and CPA (π_{CPA} = 0.0017). Among the Malayan pangolin populations, the genetic diversity of MJ1 (π_{MJ1} = 0.0024) was obviously higher than that of the MJ2 (π_{MJ2} = 0.0007). The average genetic diversity of the Chinese pangolin was higher than the Malayan pangolin (Supplementary Table S21).

Inbreeding in small populations increases genome-wide homozygosity, and the resulting depression accelerates the loss of genetic diversity. In the MJ and MP population, the average number of ROH fragments in each individual was 3,659.22±215.96 and 1,543.43±186.90, respectively. For both species, ROHs were restricted to relatively small fragments, and ROHs larger than 1Mb only accounted for 0.94% and 1.27% of ROH fragments for the MP and MJ genomes, respectively. The total length of ROH larger than 1Mb also accounted for a small proportion in the two genomes (MP: 5.19%; MJ: 6.69%) (Fig. 3A and 3B). We did not find any ROH fragments larger than 5Mb in both species. The F_{ROH} is similar to the ROH number, with F_{ROH} values being of 0.39 ± 0.024 and 0.15 ± 0.023 in the MJ and MP populations, respectively (Supplementary Table S25 and S26), and F_{ROH} longer than 1Mb sharply reduced to 0.026±0.003 and 0.0078±0.002 for the MJ and MP, respectively (Supplementary Table S25 and S26). We further compared the ROH distribution in different populations of these two species (Supplementary Fig. S19-S22). In Malayan pangolins, the inbreeding in MJ2 population (F_{ROH}=0.55±0.007) was more serious than in MJ1 population (F_{ROH}=0.15±0.012) (Fig. 3B-3D, Supplementary Table S25). Although this difference became smaller for ROH larger than 1Mb, it was still significant (Fig. 3D). Among the three Chinese pangolin populations, the inbreeding of the CPA and CPB populations were comparable (CPA: 0.18±0.036; CPB: 0.17±0.039), but much worse than the CPC population (F_{ROH}=0.06±0.005) (Fig. 3A and 3E, Supplementary Table S26). As the Malayan pangolin populations, the difference of inbreeding level among these three populations is reduced for ROH larger than 1Mb (Fig 3E, Supplementary Table S26). Noteworthy, the F_{ROH} of the Taiwan individual is much higher than all other individuals in the MP population (F_{ROH}=0.54) (Fig. 3C), although most of the ROH fragments were less than 1Mb (Supplementary Table S24, Supplementary Fig. S20). Additionally, the F_{ROH} varies greatly among individuals in the CPA or CPB population, but this was not found in the other three populations.

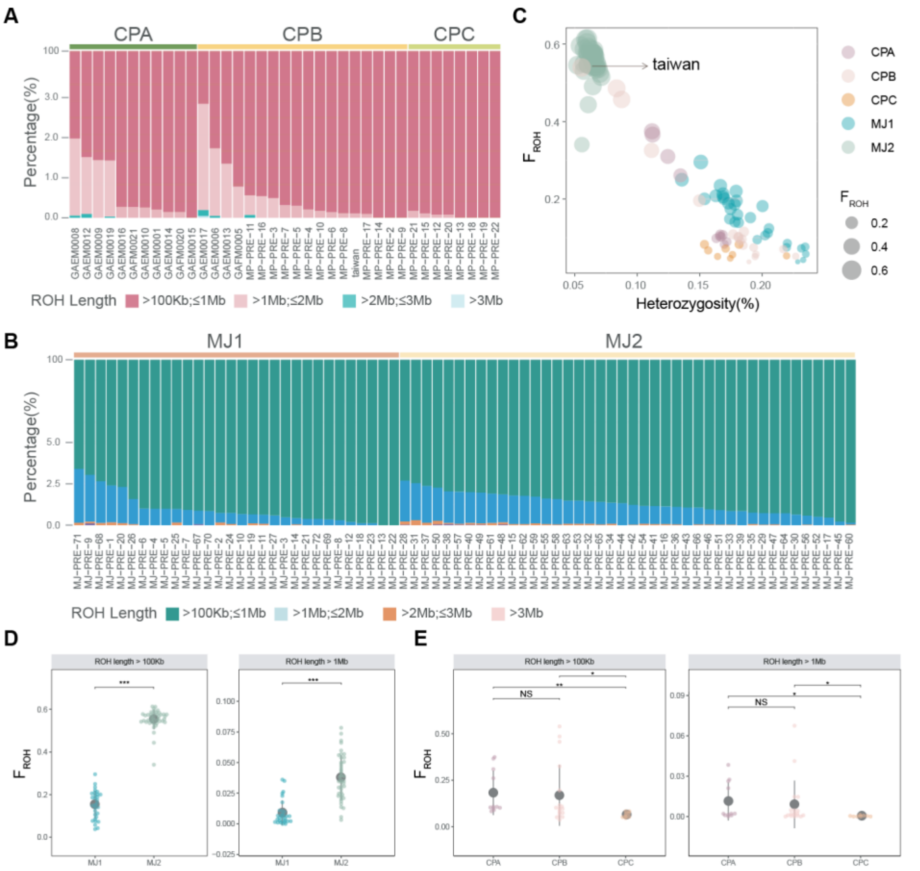

Figure 3: Genome-wide inbreeding estimated by ROH in the Chinese and Malayan pangolin populations. (A) The length distribution of ROH across the genome in the Chinese pangolin population. (B) The length distribution of ROH across the genome in the Malayan pangolin population. (C) Genome-wide heterozygosity and inbreeding estimates (F_{ROH}) for all five pangolin populations. (D) The comparison of averaged F_{ROH} in MJ1 and MJ2 populations of Malayan pangolins. (E) The comparison of averaged F_{ROH} in CPA, CPB and CPC populations of Chinese pangolins.

The genome-wide mutational load

The mutation load is the burden of deleterious variants carried by a population, and can reflect the evolutionary fitness of a population[52]. Although studies have been explored the distribution of mutational loads for Chinese and Malayan pangolins[25, 41], the HIFI genomes in this study provided new insights into the accumulation of mutational load in pangolins. Here, we screened three categories of mutational load (loss of function, LOF; missense mutation; deleterious nonsynonymous mutation, dnsSNP) based on HiFi genomes for both Chinese and Malayan pangolins (Supplementary Table S27 and S28). We calculated the individual level derived mutational load in each population to avoid bias introduced by different population size and found that individuals in the CPC population harbored the most mutational load, which was significantly more than that in the CPB and CPA populations (Fig. 4A). The missense mutations and dnsSNPs in the CPA population were comparable to those in the CPB population (Supplementary Fig. S23), but the CPB population harbored many more LOFs than did the CPA population (Fig. 4A). We then focused on the derived homozygous mutational load (DHMD) and found that the CPC population still harbored the most DHMD (Fig. 4B, Supplementary Fig. S24). The proportion of DHMD was also found to be the highest in the CPC population (Fig 4C, Supplementary Fig. S25). The CPA and CPB harbored the comparable but significantly fewer DHMD than did the CPC population (Fig 4B and 4C, Supplementary Fig. S24, Supplementary Table S29). For Malayan pangolins, the MJ1 population harbored much more derived mutational load than did the

MJ2 population (Fig. 4A, Supplementary Fig. S23, Supplementary Table S27 and S28). On the contrary, the MJ2 population has much more DHMD than that of the MJ1 population for the dnsSNP and missense mutations (Supplementary Fig. S26). However, the proportion of DHMD in the MJ2 population for the LOF was comparable to that in the MJ1 population (Fig. 4D, Supplementary Table S30), possibly because of the genetic purging of strongly deleterious mutations. In the GERP analysis, we obtained a highly similar result with the CPC population harboring the most relative mutational load in the MP populations and the MJ2 population harboring more relative mutational load than that in the MJ1 population (Fig. 4E and 4F).

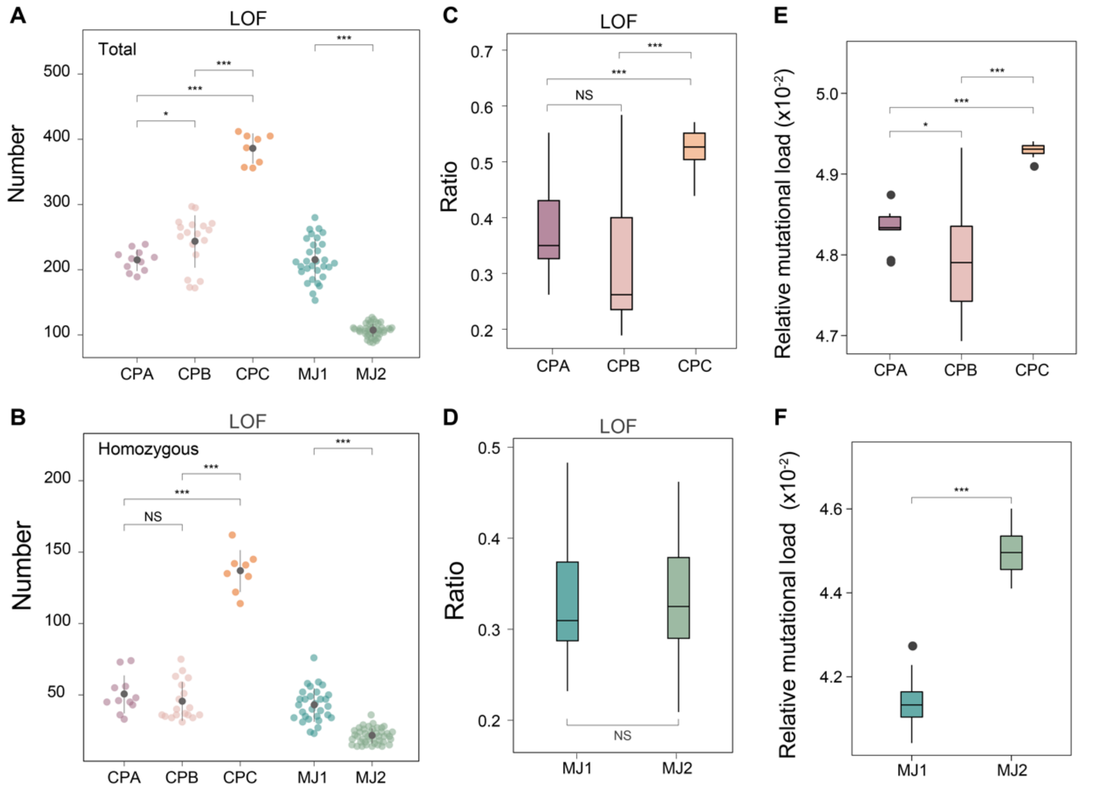

Figure 4: Mutational load in the Chinese and Malayan pangolin populations. (A) Total number of individual-level LOF mutations across five pangolin populations. (B) The number of homozygous LOF mutations at the individual-level across five pangolin populations. (C) The ratio of homozygous LOF mutations in Chinese pangolin populations was calculated as the formula of : 2 × homozygous sites / (2 × homozygous sites + heterozygous site). (D) The ratio of homozygous LOF mutations in Malayan pangolin populations was calculated as the same formula calculated for the Chinese pangolin. (E) Relative mutational load in Chinese pangolin populations (top 0.1% of GERP scores). (F) Relative mutational load in Malayan pangolin populations (top 0.1% of GERP scores). The LOF here means loss-of-function mutations.

SFS (site frequency spectrum) analysis showed that 7.91% and 9.07% of putatively damaging and neutral alleles, respectively, were fixed in the CPC population, and these two ratios were 6.84% and 9.53%, respectively, in the MJ2 population. However, the proportion of fixed allele in other three populations (CPA, CPB and MJ1) were much smaller (Fig. 5A and 5B). This indicated that the CPC and MJ2 populations may have experienced population bottleneck events, and the genetic drift drove more rare alleles to fix in these two populations [24]. By comparing SFS lines between the Chinese and Malayan pangolins, we found that the SFS lines were flatter for polymorphic loci (fixed alleles excluded) in the Malayan pangolins, while the flattest SFS line was found for the MJ2 population, indicating the possibility of more serious bottlenecks in the evolutionary history of the MJ2 population.

Genetic purging in pangolin populations

Genetic purging is an important genetic factor that have impact on the accumulation of deleterious mutations in the

population, but few studies have ever discussed this issue for pangolins. To check whether these pangolin populations are affected by the genetic purging as a consequences of inbreeding, we first compared the occurrence of mutational load (LOF, missense mutation and dnsSNP) in ROH regions (ROH*f*, the ratio of the number of mutational load to synonymous mutations in the ROH regions across the genome) and outside ROH regions (nonROH*f*, the ratio of the number of mutational load to synonymous mutations in the non-ROH regions across the genome)[27]. In the Malayan pangolin populations, the ROH*f* of highly deleterious mutations (LOF) was significantly lower than that of nonROH*f* in both the MJ1 and MJ2 population (Fig. 5C), but the difference (nonROH*f* / ROH*f*) was more pronounced in the MJ1 population than that in the MJ2 population (Fig. 5D). This denoted that many heterozygous deleterious mutations still existed in the genome[27, 28], which indicated that the genetic purging in the MJ1 and MJ2 populations were both less efficient, but stronger in the MJ2 population, which might be promoted by the higher-level inbreeding in the MJ2 population. The dnsSNP and missense mutations could reflect the same situation (Supplementary Fig. S27 and S28). The relatively lower proportion of derived homozygous LOF in the MJ2 population also presented a decrease of highly deleterious mutations that might be caused by genetic purging (Fig. 4D). However, the Rxy analysis detected the excess of mutational load in the MJ2 populations than that in the MJ1 populations (Supplementary Fig. S29), with the lowest degree of this excess found in the LOF, further supporting that the genetic purging is limited, but tends to remove highly damaging mutations.

In the Chinese pangolin populations, the ROH*f* for LOF was lower than nonROH*f* in all three populations. The values of non-OH*f* and ROH*f* in the CPA population were highly close, but the non-OH*f* was significantly higher than the ROH*f* in both of the CPB and CPC populations (Fig. 5E and 5F, Supplementary Fig. S30), indicating that the purging of highly deleterious alleles in the CPA population was more efficient than that in the CPB and CPC populations. The Rxy also reflected that the CPA population harbored the least deleterious mutations, followed by the CPB and the CPC populations (Supplementary Fig. S31). However, we did not detect an obvious reduction in DHMD in any of the three Chinese pangolin populations as we detected in the MJ2 population, suggesting a less efficient genetic purging in the MP populations than in the MJ populations. Additionally, the number of fixed damaging alleles was not significantly less than neutral alleles in all five populations (Fig. 5A and 5B), further implying that the genetic purging in pangolin populations is weak and not sufficient to clear a large number of deleterious mutations.

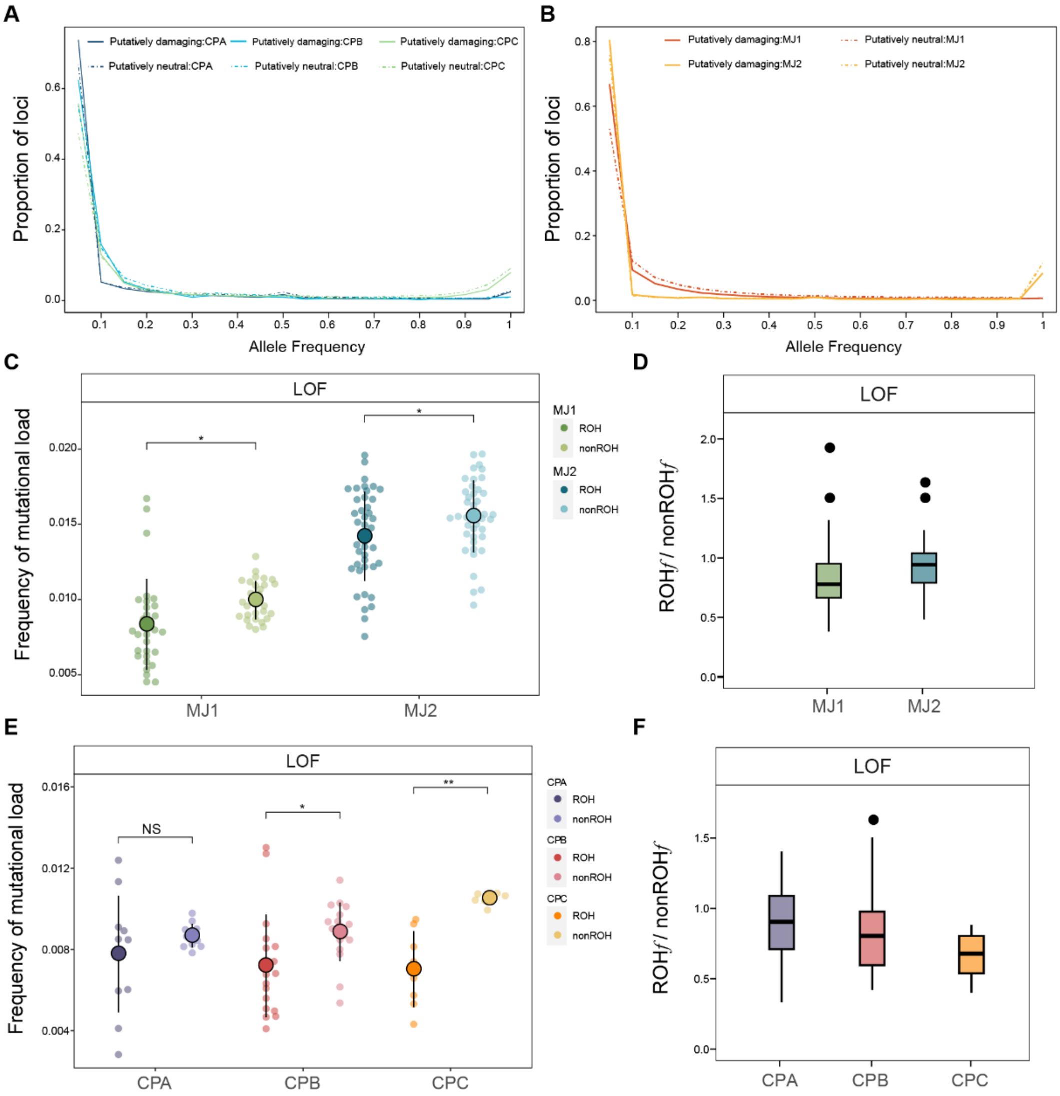

Figure 5: The SFS and genetic signals of genetic purging in pangolin populations. (A) SFS for putatively damaging (LOF and missense mutations) and neutral mutations (intergenic variants) in CPA, CPB, and CPC populations. (B) SFS for putatively damaging and neutral mutations in MJ1 and MJ2 populations. (C) Dot plot showed the occurrence of LOF mutations in the two Malayan pangolin populations calculated as the ratio of the number of mutational load to synonymous mutations in the ROH regions (ROH*f*) or nonROH (nonROH*f*) regions across the genome. (D) Dot plot showed the occurrence of LOF mutations in the three Chinese pangolin populations calculated as that in (c). (E) The ratio of ROH*f* to nonROH*f* for the LOF in the two Malayan pangolin population. (F) The ratio of ROH*f* to nonROH*f* for the LOF in the three Chinese pangolin populations.

Discussion

The first haplotype-resolved and chromosome-scale pangolin genome

Sixteen pangolin genomes representing all eight species have been reported to date, including three genomes assembled from ONT long reads (one Chinese pangolin genome[46], one Malayan pangolin genome[46], and one giant pangolin genome[45]) and 13 short-read assembled genomes[25, 33, 43, 44]. For the Chinese pangolin, two short-read generated genomes [25, 33] and one ONT long-read assembled genome have been reported[46]. The Chinese pangolin genome we assembled in this study has much better contiguity, which is 1,939.53-fold, 301.36-

fold and 2.89-fold longer than the M_pentadactyla-1.1.1, YNU_ManPten_2.0 and ASM2424420v1 genomes, respectively (Supplementary Table S31). There are also three reported genome assemblies for the Malayan pangolin, including two short-read generated genomes [25, 33] and one ONT long-read assembled genome[46]. The contiguity of the Malayan pangolin genome in this study was even higher than the Chinese pangolin genome we assembled, which is 3,740.34-fold, 829.79-fold and 3.87-fold greater than the ManJav1.0, YNU_ManJav_2.0 and ASM2460508v1 genomes, respectively (Supplementary Table S32). The contig number of the two pangolin genomes (Chinese pangolin: 294; Malayan pangolin: 154) we assembled were much fewer than the ONT long reads assembled genomes (Chinese pangolin: 6,002; Malayan pangolin: 2,568). Another advantage of our assemblies in this study is the partitioning of the diploid chromosomes into haploid chromosomes, which allowed us to detect genetic differences between haplotigs and provide a better understanding of allele-specific functions[53, 54], which cannot be achieved well with a hybrid reference genome. These advancements are anticipated to be further developed and enhance the precise conservation efforts for pangolins.

HiFi genomes improve the evaluation of genetic diversity and inbreeding

Accurate and precise evaluation of genome-wide extinction risks by measuring a series of genetic parameters is the central issue in conservation genomics and highly depends on the quality of the reference genome[18]. However, what genetic parameters could be improved the most by a better reference genome? Here we showed that the two genetic parameters most promoted by a higher-quality reference genome were genetic diversity (π) and inbreeding (ROH). We could detect more variants across the genome based on the LG than the SG, because long reads could 1) span much more complex genomic regions[18] and 2) generate much longer contigs than short reads[55, 56]. Many genomic regions that cannot be assembled from short reads can be assembled from long reads, and these regions may contain important variants. Additionally, longer contigs facilitate a higher number of reads aligning accurately to the reference genome. Both of these aspects could contribute to enhancing the accuracy of genetic diversity calculations. However, it may not make sense to compare genetic diversity between different populations based on SG genomes with similar contiguity. The estimation of inbreeding by detecting ROH across the genome highly depends on the genome contiguity, because short contigs in the SG cannot span over long ROH fragments. As anticipated, the inbreeding level detected using the LG were significantly higher than those identified using the SG for ROH fragments larger than 1Mb. When we focused on ROH larger than 100kb, this difference in F_{ROH} was still significant in the Malayan pangolin population but not in the Chinese pangolin population, which we inferred should have been resulted from the different contiguities of the two pangolin genomes. Indeed, the contig N50 of the SG for the Chinese pangolin (133.77 kb) was substantially longer than that of the SG for the Malayan pangolin (73.8 kb), allowing for the detection of ROH longer than 100 kb. In contrast, the scaffold N50 of the Malayan pangolin genome was longer than that of the Chinese pangolin genome, suggesting that contiguity contributed more than scaffold contiguity to the detection of long ROH across the genome.

Genome-wide extinction risks in pangolin populations

Although the Chinese and Malayan pangolin are listed as the Critically Endangered species by the IUCN Red List, the genome-wide genetic diversity of these two species is moderate and even higher than other endangered flagship species[25, 57], such as tiger[55], giant panda[58], golden snub-nosed monkey[59], and kākāpo[28]. In this study, we discovered even higher genetic diversity than the previous report[25, 57] in the two pangolin species (Supplementary Fig. S32, Supplementary Table S33). Although the pangolin populations have been declining for a long time, we inferred that the recent population decline caused by poaching and illegal trade maybe more serious than ever before, leading to much smaller and more isolated pangolin populations. Therefore, genetic drift and inbreeding may have not a serious effect of reducing the genetic diversity. As expected, even though the high-quality HiFi genome improved the estimation of inbreeding, the F_{ROH} in both species was still lower than many other endangered species[24, 55], indicating a fine intrinsic genetic background for these pangolin populations.

However, both the genetic diversity (π_{MJ2}=0.0007) and inbreeding ($F_{ROH>100kb}$=0.55) in the MJ2 Malayan population were much worse than those in other pangolin populations, which may be caused by the isolation and limited gene flow with other populations, because the MJ2 population is distributed across Southeast Asia[25] and the gene flow is easily separated by islands. In addition, we also found a much faster and sharper population decline for the MJ2 population within the most recent 10,000 years, when compared with the other four pangolin populations (Supplementary Fig. S17), indicating that the MJ2 population may facing more extinction risks. However, we cannot precisely locate this population because of the lack of accurate sampling locations[25], leaving a question for future conservation work.

The derived mutational load in all five pangolin populations were greater than Amur tiger and South China tiger populations, even with their lower inbreeding levels[55]. For the Chinese pangolin, the CPC population had the highest proportion of mutational load, and we speculated that the CPC population represents an ancient and isolated Chinese pangolin population in Yunnan Province and is less disturbed by human activity but has accumulated a large number of mutational load over its evolutionary history, because we detected stronger drift in the CPC population, which can cause a reduced efficacy of purifying selection to remove deleterious mutations[28]. For the Malayan pangolin, the MJ2 population showed low genetic diversity and high inbreeding. Although the high inbreeding in the MJ2 population may promote the purging of deleterious mutations, we still found a large amount of DHMD in the MJ2 population, which may be explained by the less efficient genetic purging of deleterious mutations, particularly for less damaging alleles.

Novel implications for the global conservation of these two pangolin species

High-volume poaching and trafficking have resulted in the overexploitation of pangolins, and the wild population, particularly the Chinese and Malayan pangolins, has plummeted to the edge of extinction[25, 36-39]. Although moderate inbreeding and genetic diversity for these two species indicated a fine intrinsic genetic status, the population differentiation (F_{ST}) among both Chinese and Malayan pangolin populations are large[25, 57, 60], even larger than many subspecies level genetic differences [61-63], and genome-wide risks in different populations of Chinese and Malayan pangolin are also different[25, 57]. Therefore, in the pursuit of protecting and conserving pangolins, addressing, and managing the issues of illicit poaching and trafficking are just as crucial as genetic rescue efforts. Implementing timely protective and conservation measures for both the Chinese and Malayan pangolins will additionally contribute to facilitating genetic rescue initiatives. Notably, both the genetic diversity and inbreeding in the MJ2 population are much worse than those in other pangolin populations, suggesting that the MJ2 population may suffer more serious survival pressures than other pangolin populations, and should receive more attention and protection measures.

Materials and Methods

Samples, Re-sequencing Data and Ethics Statements

The Chinese pangolin and Malayan pangolin used for genome assembly were wild rescued individuals by the Guangdong Wildlife Rescue Center. During routine examinations, 5 mL of blood was collected with an anticoagulant tube and immediately transferred to liquid nitrogen and stored at -80°C. The sample collection, experiment, and research design were all approved by the Institutional Review Board of BGI (BGI-IRB E22017). We strictly adhered to the guidelines provided by the BGI-IRB for all procedures conducted in this study. The whole-genome sequencing data of 37 Chinese pangolin and 72 Malayan pangolin individuals were downloaded from National Center for Biotechnology Information (NCBI) and China National GeneBank DataBase (CNGBdb) for population genomic analysis in this study[25, 33, 57].

Nucleic Acid Extraction, Library Preparation, and Sequencing

Total genomic DNA was extracted using a DNeasy Blood & Tissue Kit (Qiagen, USA) for whole genome sequence (WGS) library preparation. Total RNA was extracted from blood with Trizol reagent (Invitrogen, USA) from blood, and 250-300 bp reverse transcribed cDNA fragments were used for DNA library construction. Two Hi-C libraries were prepared with *Dpn*II restriction endonuclease. DNA libraries were subjected to the Illumina HiSeq X Ten platform at Novogene (Tianjin, China) for paired-end sequencing. For high-molecular-weight genomic DNA, the isolation was performed using the sodium dodecyl sulfate (SDS)-based method, and the purification was carried out by the Qiagen Genomic Kit. A 15k library was constructed using high-quality DNA samples (main band > 30kb) and sequenced with the PacBio Sequel II platform at Novogene (Tianjin, China).

Genome Assembly and Assessment

To estimate the genome size, a total of ~100 Gb WGS short reads were used for analysis by the kmerfreq method[64] (v5.0). Primary genomes were assembled using hifiasm[42] (v0.16.1) with PacBio HiFi and Hi-C sequencing data. Genome redundancy was removed by the software of Purge_dups[65] (v1.2.5). Then, the Hi-C sequencing reads are mapped to the primary genomes by the *mem* algorithm of Burrows-Wheeler Aligner (BWA, v0.7.17) [66, 67], while Hi-C data quality control was conducted by the Juicer[68] (v1.5). The 3d-DNA pipeline (v190716) was finally used to concatenate and review the scaffolds to chromosome-scale genomes[69].

The genome completeness was evaluated by BUSCO (Benchmarking Universal Single-Copy Orthologs) (v5.2.2) software with the vertebrata_odb10 data set [70]. We carried out Merqury[71] (release 20200430) k-mer analysis and alignment of whole genome sequencing reads to the reference genome to evaluate the accuracy of genome assembly. Genome regions covered by PacBio long-read greater than 10-fold were considered as accurately assembled regions[48]. The identification of syntenic blocks between pangolin genomes was primarily performed by the NUCmer program in MUMmer[72] (v4.0.0rc1), followed by filtration using the delta-filter program in MUMmer (v4.0.0rc1) with parameters "-i 90 -l 5000".

Genome Annotation.

Repeat elements in the genome were annotated using *de novo* and homology-based methods. *De novo* repeats were first annotated using LTR finder[73] (v1.0.6) and RepeatModeler2[74] (v2.0.1), and the identified repeats were then merged into the RepBase library as known elements. Transposable elements were identified and classified by RepeatMasker (v4.0.5) with a conserved BLASTN search against the RepBase library [75]. The RepeatProteinMask program in the RepeatMasker (v4.0.5) was used to identify repeat proteins [75]. Tandem repeats were annotated using Tandem Repeats Finder[76] (v4.07).

Protein-coding genes were annotated using *de novo*, homology-based, and transcript-based approaches after masking repeat elements. For the *de novo* method, we used the Augustus[77] (v3.0.3), GlimmerHMM[78] (v3.0.1), and SNAP[79] (v11/29/2013) to predict gene models. For the transcript-based prediction, transcripts were mapped to the reference genome using the HISAT2[80] (v2.1.0) and then assembled using StringTie[81] (v1.3.3b) based on clean RNA-seq data. Homology-based gene annotation was performed by using Blastall[82] (v2.2.26) with an E-value cut-off of 1e-5 to align against the protein sequences of *Homo sapiens*, *Mus musculus*, *Canis lupus familiaris*, and *Felis catus*. The final protein-coding gene set was generated using the MAKER[83] (v3.01.03) pipeline by combining high-quality homology-based, *de novo* and RNA-seq supported genes.

Functional annotation was performed by using BLAST (v2.13.0) search against the SwissProt, TrEMBL, and Kyoto Encyclopedia of Genes and Genomes (KEGG) databases with an E-value cut-off of 1e-5. InterProScan[84] (v5.52-86.0) was used to predict motifs, domains, and Gene Ontology (GO) terms. tRNA genes were identified using tRNAscan-SE[85] (v1.3.1), snRNA and miRNA genes were detected by searching the reference sequences against

the content of the Rfam database (Release 12.0) using the BLAST (v2.13.0) and the program cmsearch from infernal (v1.1.1) software.

Detection of Structural Variants (SVs) in the Pangolin Genome

To identify sequence differences between parental genomes, sequence alignment was performed using Mummer (v4.0.0rc1) with the parameters 'nucmer --maxmatch -c 500 -b 500 -l 100' [72]. SVs were detected based on the alignment results using SyRi[86] (v1.3). To verify the accuracy of the detected SVs, we aligned the PacBio long reads to the reference genome using BLAST (v2.13.0) to determine whether the reads could cross the breakpoints. Moreover, we extracted 300 bp of upstream/downstream flanking sequences of each breakpoint and manually verified them using DNBSEQ short reads by IGV[87] (v2.13.3) software. To identify gene loss in haploid genome, we screened pseudogenes interrupted by SVs using Mummer alignment and checked whether these genes had other copies across the entire genome.

Genome-wide Variant Calling and Quality Control

The BWA *mem* algorithm (v0.7.17) [66] was applied to map whole-genome resequencing data of 72 Malayan pangolins and 37 Chinese pangolins to each of their reference genomes with default parameters. Sentieon[88] (v202010.01) was then used to sort, reorder, and deduplicate the alignment files for variant calling. Variants were detected for each individual using the Sentieon DNAseq Haplotyper pipeline, which is similar to the Genome Analysis Toolkit (GATK) HaplotypeCaller pipeline. Joint variant calling was conducted using Sentieon DNAseq GVCFtyper with all gVCF files to generate a VCF file. To prepare for downstream analysis, the variant set was filtered to remove InDels and multi-allelic variants. For variant quality control, a stringent filtering step was performed with the following parameters: "QD < 2.0 || FS > 60.0 || MQ < 40.0 || MQRankSum < -12.5 || ReadPosRankSum < -8.0". Additionally, we filtered SNPs that were missed in more than 20% of individuals in a population. In this part, we used both the short-read assembled genome (SG) and long-read assembled genome (LG) as references to generate variant sets for downstream comparison.

Population Structure Analysis

Before we performed principal component analysis(PCA), the VCF file was converted to PLINK format using PLINK software[89] (v1.90b6.10), then the genome-wide complex trait analysis (GCTA)[90] (v1.92.2) software was used for PCA analysis with default parameters. To construct a phylogenetic tree, vcf2phylip[91] (v2.7) was used to convert the VCF file into PHYLIP format. Then, the best substitution model was calculated using jModelTest[92] (v2.1.10), and the maximum likelihood phylogenetic tree was constructed using the IQ-TREE[93] (v1.6.12) software with default parameters. ADMIXTURE[94] (v1.3.0) was used to determine the ancestry proportion with a specified number of clusters (K) ranging from 1 to 10. In this analysis, we used both the SG and LG as the reference genome to generate two groups of results.

Population Demography Inference

SMC++[95] (v1.5.1) was used to infer the historical changes of effective population size of different pangolin populations. The SMC++ results were visualized by scaling the time to real years using a generation time of one year and a mutation rate of $\mu = 1.47 \times 10^{-8}$[25, 33] for both the Malayan and Chinese pangolins. Meanwhile, we used MSMC2[96] (v2.1.1) to infer the changes in effective population size over evolutionary history with four randomly selected individuals from each pangolin population. SNPs were first phased by Beagle[97] (v5.1) and then subjected to MSMC2 for inference of population history. We used both the SG and LG as the reference genomes to generate two group of results for comparison.

ROH and Genetic Diversity

To detect ROH fragments, multi-individual VCF files were converted into PLINK bfile format using the PLINK[89] (v1.90b6.10) software. The ROH was then detected using the PLINK[89] (v1.90b6.10) software with the parameters "--homozyg --homozyg-window-snp 20 --homozyg-kb 10 --homozyg-density 50" [98]. ROHs shorter than 100 kb were excluded from the downstream analysis. F_{ROH} was calculated as $F_{ROH} = L_{ROH}/L_{AUTOSOME}$, where L_{ROH} represents the total length of ROHs in each genome and $L_{AUTOSOME}$ represents the total length of the autosomes. Genome-wide genetic diversity (π) was calculated using vcftools[99] (v0.1.16) with the parameters "vcftools --gzvcf vcf.gz --window-pi 500000 -out result". Genome-wide heterozygosity was calculated using vcftools[99] (v0.1.16) with "vcftools --gzvcf vcf.gz --het --out result" parameters. For both ROH and genetic diversity analysis, we used both the SG and LG as reference genomes for comparison, but only the results calculated based on the LG were used for further discussion.

Mutational Load and Genetic Purging Analysis

To identify mutational load in protein-coding genes, variants were first annotated using ANNOVAR[100] (v20191024) and SnpEff[101] (v.5.0e). Variants annotated as stop gained, splice acceptor variant, or splice donor by SnpEff[101] were predicted to be loss-of-function (LOF) mutations. Nonsynonymous variants with ⩾150 Grantham Score were considered as deleterious mutations (dnsSNP) [102]. To determine the derived allele, the Malayan pangolin genome was split into 100 bp reads and mapped to the Chinese pangolin genome. If an allele was found within the Malayan pangolin genome and simultaneously represented the predominant allele (with an allele frequency exceeding 50%) within the Chinese pangolin population, we designated the allele as the ancestral state within the Chinese pangolin genome[103]. The same approach was used to determine the ancestral state of variants of the Malayan pangolin.

The occurrence of mutational load in ROH and nonROH region (${}_{ROH}f$ and ${}_{nonROH}f$) for each individual genome was calculated by dividing the total number of deleterious mutations (N_m) within ROH or nonROH region by the number of synonymous mutations in the same region (S_{ROH} and S_{nonROH}):

$${}_{ROH}f = \frac{N_m}{S_{ROH}}$$

$${}_{nonROH}f = \frac{N_m}{S_{nonROH}}$$

To estimate relative excess of deleterious mutations in one pangolin population compare to another population, we performed the Rxy analysis for dnsSNP, missense mutations, LOF, and synonymous mutations between pairs of pangolin populations [27]. We calculated the Rxy value as the following formula:

$$L_X = \frac{\sum_{i\in C}(m_X^i/s_X^i)(1 - m_Y^i/s_Y^i)}{\sum_{i\in I}(m_X^i/s_X^i)(1 - m_Y^i/s_Y^i)}$$

$$R_{X/Y} = L_X/L_Y$$

The m_X^i represented the count of derived alleles for the above-mentioned mutations observed at each site (*i*) within one population (*X*); the m_Y^i represented that in another population (*Y*). s_X^i and s_Y^i represented the total number of alleles at each site (*i*) of the population (*X* or *Y*). *C* represented the above-mentioned category of protein-coding sites, while *I* denoted the intergenic sites. Here, we employed the jackknife method during the calculation and obtain a standard error measurement. If Rxy = 1, both populations have the same level of derived mutation load, whereas if Rxy < 1, then population Y has more derived load than X and vice versa if Rxy > 1.

Genomic Evolutionary Rate Profiling (GERP) Scores

It is difficult to estimate genetic load without fitness data, here we calculated the relative mutational load in each individual genome. First, we screened derived alleles distributed in the highly conserved genome region of these two pangolin species by the genomic evolutionary rate profiling scores (GERP) method. To calculated the GERP scores, we selected the genomes of 37 species (*Acinonyx jubatus*, *Bos taurus*, *Callithrix jacchus*, *Canis lupus*, *Cavia porcellus*, *Choloepus hoffmanni*, *Dasypus novemcinctus*, *Dipodomys ordii*, *Echinops telfairi*, *Equus caballus*, *Erinaceus europaeus*, *Felis catus*, *Homo sapiens*, *Loxodonta africana*, *Lynx canadensis*, *Mus musculus*, *Myotis lucifugus*, *Ochotona princeps*, *Oryctolagus cuniculus*, *Panthera pardus orientalis*, *Panthera tigris*, *Pan troglodytes*, *Prionailurus bengalensis*, *Procavia capensis*, *Pteropus vampyrus*, *Puma concolor*, *Rattus norvegicus*, *Sorex Araneus*, *Spermophilus tridecemlineatus*, *Tupaia belangeri*, *Tursiops truncates*, *Vicugna pacos*, *Manis javanica*, *Manis pentadactyla*, *Tamandua tetradactyla*, *Ovis aries*, and *Vulpes lagopus*) for screening ultra-conserved genome regions. We split these genomes into 100 bp reads to generate fastq files. Then, we respectively aligned these fastq files to the Malayan pangolin and Chinese pangolin genome using the *mem* algorithm in BWA (v0.7.17-r1188) with "-B 3" parameter. GERP scores were then calculated by the program gerpcol from the GERP++[104] software (http://mendel.stanford.edu/sidowlab/downloads/gerp/index.html) based on above-mentioned alignment files. In general, low GERP scores (<1) usually represent putatively neutral genome regions, but high GERP scores (>1) indicate conserved genome regions [28]. Derived alleles in more conserved genome regions (those with higher GERP scores), these alleles are likely to be more deleterious. In this study, we calculated the relative mutational load with mutations having the top 0.1% GERP scores to select more deleterious alleles distributed in the highly conserved genome regions [24], and the relative mutational load was calculated by the formula: the sum of all homozygous and heterozygous derived alleles multiplied by their conservation score over the total number of derived alleles, with the heterozygous counted as one allele and homozygous sites counted as two alleles [28]. Therefore, a higher relative mutation load indicates a relatively larger proportion of derived alleles could be found at more conserved genomic regions.

Site-Frequency Spectrum (SFS) Analysis

For SFS analysis, we calculated the frequency of each type of mutations at every site in different pangolin populations. We considered intergenic variants as neutral, while LOF and missense variants as putatively damaging mutations[24]. For SFS in each pangolin population, we subsampled nonmissing derived alleles from each locus for calculation[24]. Fixed (frequency=1) and missing (frequency=0) alleles were included in the SFS for the five populations. We used LG as the reference genome for SFS analysis.

Additional Files

Supplementary Fig. S1. The heatmap represents the contact matrices generated by aligning the Hi-C data to the haplotype-resolved MJ and MP genomes. (A) The Hi-C map of the diploid Malayan pangolin genome. (B) The Hi-C map of the diploid Chinese pangolin genome.

Supplementary Fig. S2. Estimated genome size of the Chinese and Malayan pangolin genomes by using K-mer frequency analysis with k-mer size of 17. (A) The K-mer spectra of the Chinese pangolin genome. (B) The K-mer spectra of the Malayan pangolin genome.

Supplementary Fig. S3. Sequencing depths of each pseudo-chromosome. (A) Sequencing depths of the 19 autosomes, X chromosome (Hic_scaffold_8), and Y chromosome (Hic_scaffold_22) in the MP genome. (B) Sequencing depths of the 18 autosomes, X chromosome (Hic_scaffold_7), and Y chromosome (Hic_scaffold_20) in

the MJ genome.

Supplementary Fig. S4. K-mer spectra plot estimated by Merqury. (A) K-mer spectra plot for the haplotype-resolved genome of MP. (B) K-mer spectra plot for the haplotype-resolved genome of MJ.

Supplementary Fig. S5. The sequencing depths of the two groups of haplotigs in both MJ and MP genomes. (a, b) Alignment with DNBSEQ read. (c, d) Alignment with PacBio HiFi reads.

Supplementary Fig. S6. Pairwise differences observed between the haploid genomes of Malayan and Chinese pangolin. The sliding window was set to be 100 bp.

Supplementary Fig. S7. Dot plot between MJH1 (x-axis) and MJH2 (y-axis), plotted by pafCoordsDotPlotly.

Supplementary Fig. S8. Dot plot between MPH1 (x-axis) and MPH2 (y-axis), plotted by pafCoordsDotPlotly.

Supplementary Fig. S9. Schematic diagram of the method we validated the structural variants across the genome.

Supplementary Fig. S10. The GO enrichment result of genes distributed in structural variants of MJ.

Supplementary Fig. S11. The KEGG enrichment result of genes distributed in structural variants of MJ.

Supplementary Fig. S12. The GO enrichment result of genes distributed in structural variants of MP.

Supplementary Fig. S13. The KEGG enrichment result of genes distributed in structural variants of MP.

Supplementary Fig. S14. Comparisons of LG and SG on PCA analysis for both Chinese and Malayan pangolin populations. (A) PCA analysis of Malayan pangolin populations based on SG. (B) PCA analysis of Malayan pangolin populations based on LG. C PCA analysis of Chinese pangolin populations based on SG. (D) PCA analysis of Chinese pangolin populations based on LG.

Supplementary Fig. S15. Comparisons of LG and SG on the construction of phylogenetic tree for both Chinese and Malayan pangolin populations. (A) Phylogenetic tree constructed based on the SG for Malayan pangolin populations. (B) Phylogenetic tree constructed based on the LG for Malayan pangolin populations. (C) Phylogenetic tree constructed based on the SG for Chinese pangolin populations. (D) Phylogenetic tree constructed based on the LG for Chinese pangolin populations.

Supplementary Fig. S16. Comparisons of LG and SG on the admixture analysis for both Chinese and Malayan pangolin populations. (A) Genome-wide admixture analysis for three populations of Chinese pangolin based on the SG. (B) Genome-wide admixture analysis for three populations of Chinese pangolin based on the LG. (C) Genome-wide admixture analysis for two populations of Malayan pangolin based on the SG. (D) Genome-wide admixture analysis for two populations of Malayan pangolin based on the LG.

Supplementary Fig. S17. Comparisons of LG and SG on the population history analysis for both Chinese and Malayan pangolin populations. (A) The dynamics of effective population size of Malayan pangolin populations analyzed based on the SG. (B) The population size dynamics of Malayan pangolin populations analyzed based on the LG. (C) The population size dynamics of Chinese pangolin populations analyzed based on the SG. (D) The population size dynamics of Chinese pangolin populations analyzed based on the LG.

Supplementary Fig. S18. Comparisons of LG and SG on the inference of population separation among Chinese and Malayan pangolin populations. (A) The divergence time between two populations of Malayan pangolin estimated based on the LG. (B) The divergence time between two populations of Malayan pangolin estimated based on the LG. (C) The divergence time among three populations of Chinese pangolin estimated based on the SG. (D) The divergence time among three populations of Chinese pangolin estimated based on the LG.

Supplementary Fig. S19. The population-level ROH distribution in three Chinese pangolin populations.

Supplementary Fig. S20. The individual-level distribution of ROH larger than 100Kb in Chinese pangolin genomes.

Supplementary Fig. S21. The population-level ROH distribution in two Malayan pangolin populations.

Supplementary Fig. S22. The individual-level distribution of ROH larger than 100Kb in Malayan pangolin genomes.

Supplementary Fig. S23. Total deleterious nonsynonymous SNP (dnsSNP) (A) and missense (B) mutations at the

individual level were assessed across five populations of Chinese and Malayan pangolins.

Supplementary Fig. S24. The number of individual-level homozygous dnsSNP (A) and missense (B) mutations across the five pangolin populations.

Supplementary Fig. S25. The ratio of homozygous missense mutations and dnsSNPs in Chinese pangolin populations was calculated as the formula of : 2 × homozygous sites / (2 × homozygous sites + heterozygous site).

Supplementary Fig. S26. The ratio of homozygous missense mutations and dnsSNPs in Malayan pangolin populations was calculated as the formula of : 2 × homozygous sites / (2 × homozygous sites + heterozygous site).

Supplementary Fig. S27. Dot plot showed the occurrence of dnsSNPs (A) and missense mutations (B) in the two Malayan pangolin populations calculated as the ratio of the number of mutational load to synonymous mutations in the ROH regions or nonROH regions across the genome.

Supplementary Fig. S28. The ratio of ROH*f* to nonROH*f* for dnsSNP and missense mutations in Chinses pangolin populations (A) and Malayan pangolin populations (B).

Supplementary Fig. S29. The Rxy ratio of derive alleles in MJ2 (x) to MJ1 (y) for dnsSNP, synonymous, missense and LOF. The Rxy <1 indicated the population y has more derived alleles than population x.

Supplementary Fig. S30. Dot plot showed the occurrence of dnsSNPs (A) and missense mutations (B) in the three Chinese pangolin populations calculated as the ratio of the number of mutational load to synonymous mutations in the ROH regions or nonROH regions across the genome.

Supplementary Fig. S31. The Rxy ratio of derive alleles in x population to y population (x/y: CPB/CPC; CPA/CPC; CPA/CPB) for dnsSNP, synonymous, missense and LOF. The Rxy <1 indicated the population y has more derived alleles than population x.

Supplementary Fig. S32. Comparison of genome-wide π of the Malayan pangolin and Chinese pangolin with other endangered species. Abbreviations along the X-axis are as follows: CMA: Brown eared pheasant (*Crossoptilon mantchuricum*), ASI: Chinese alligator (*Alligator sinensis*), PTA: Amur tiger (*Panthera tigris altaica*), AFU: Red panda (*Ailurus fulgens*), AME: Giant panda (*Ailuropoda melanoleuca*), MJ: Malayan pangolin (*Manis javanica*), MBE: Dwarf musk deer (*Moschus berezovskii*), MP: Chinese pangolin (*Manis pentadactyla*).

Supplementary Table S1. Summarized sample information in this study.

Supplementary Table S2. Information of five populations in Chinese and Malayan pangolin included in this study.

Supplementary Table S3. Statistics of genome assemblies for the Chinese pangolin and Malayan pangolin.

Supplementary Table S4. The length of each chromosome in the Chinese pangolin genomes.

Supplementary Table S5. The length of each chromosome in the Malayan pangolin genomes.

Supplementary Table S6. Quality assessment of the MP and MJ genomes by the Merqury software.

Supplementary Table S7. BUSCO analysis of genome assemblies in this study.

Supplementary Table S8. The mapping rates of four types of sequencing data to genomes assembled in this study.

Supplementary Table S9. The overall statistics of repeats in the Chinese and Malayan pangolin genomes.

Supplementary Table S10. Statistics of repeat elements identified by *de novo* method in Chinese and Malayan pangolin genomes.

Supplementary Table S11. Statistics of the annotated genes in Chinese and Malayan pangolin genomes.

Supplementary Table S12. BUSCO analysis of gene sets for Chinese and Malayan pangolin.

Supplementary Table S13. Statistics of functional annotation for the Malayan and Chinese pangolin's gene sets.

Supplementary Table S14. Statistics of ncRNA annotation.

Supplementary Table S15. Pairwise differences observed in comparisons between the haplotype genomes of Chinese and Malayan pangolin, the sliding window was set to be 100bp.

Supplementary Table S16. Chromosomal structural variants (>50bp) of MJH1 and MJH2, and of MPH1 and MPH2.

Supplementary Table S17. Genes distributed in the structural variants of MJ and MP genomes.
Supplementary Table S18. The KEGG enrichment result of genes distributed in the structural variants of MJ/MP.
Supplementary Table S19. The GO enrichment result of genes distributed in the structural variants of MJ/MP.
Supplementary Table S20. Functional description of pseudogenes interrupted by structural variants.
Supplementary Table S21. SNPs number, genetic diversity (π), heterozygosity and SNP density calculated based on LG and SG in Malayan and Chinese pangolin populations.
Supplementary Table S22. The comparison of the SG and LG for estimating ROH in Malayan pangolin and Chinese pangolin populations
Supplementary Table S23. The count and length of ROH fragments in the two populations of Malayan pangolin.
Supplementary Table S24. The count and length of ROH fragments in the three populations of Chinese pangolin.
Supplementary Table S25. The F_{ROH} in the two populations of Malayan pangolin.
Supplementary Table S26. The F_{ROH} in the three populations of Chinese pangolin.
Supplementary Table S27. The number of dnsSNP, LOF, missense mutations and synonymous SNPs in different populations of the Malayan and Chinese pangolin.
Supplementary Table S28. The number of dnsSNP, LOF, missense mutations and synonymous SNPs at individual level in different populations of the Malayan and Chinese pangolin.
Supplementary Table S29. The individual level genetic load estimates in all Chinese pangolin populations.
Supplementary Table S30. The individual level genetic load estimates in all Malayan pangolin populations.
Supplementary Table S31. Comparison of the assembly statistics with the previously published Chinese pangolin genomes.
Supplementary Table S32. Comparison of the assembly statistics with the previously published Malayan pangolin genomes.
Supplementary Table S33. Comparison of genome-wide nucleotide diversity (π) of the Malayan pangolin and Chinese pangolin with reference to other endangered species on the IUCN Red List.

Data Availability

Bioproject and biosample for the genomic data of this study were submitted to NCBI under accession numbers PRJNA1114675. The data that support the findings in this study also have been deposited into CNGB Sequence Archive (CNSA) [105]of China National GeneBank DataBase (CNGBdb) [106] with accession number CNP0004630. The resequencing data in this study were retrieved from earlier studies (37 Chinese pangolins: CNP0001723, CNGBdb; PRJNA529540 and PRJNA20331, NCBI Read Archive. 72 Malayan pangolins: PRJNA529540, NCBI Read Archive) [25, 33, 57].

Abbreviations

WGS: Whole genome sequence; RNA-seq: RNA sequence; BUSCO: Benchmarking Universal Single-Copy Orthologs; HiFi: High fidelity; Hi-C: High-throughput/resolution chromosome conformation capture; PCA: Principal component analysis; SNP: Single-nucleotide polymorphism; TE: Transposable element; KEGG: Kyoto Encyclopedia of Genes and Genomes; GO: Gene Ontology; GATK: Genome Analysis Toolkit; SG: Short-read assembled genome; LG: Long-read assembled genome; ROH: Runs of homozygosity; LOF: Loss-of-function;

dnsSNP: Deleterious nonsynonymous mutation; GERP: Genomic evolutionary rate profiling; SFS: Site-Frequency Spectrum.

Author Contributions

Tianming Lan, Huan Liu, Yinping Tian and Yan Hua conceived and initiated the project. Yan Hua, Jun Li, Fanghui Hou, Tengcheng Que, Wenjian Liu and Kai Wang collected the samples. Shangchen Yang, Jin Chen, Chuanling Yin, Liangyu Cui and Yinping Tian performed DNA isolation, library preparation and genome sequencing. Haimeng Li, Minhui Shi, Boyang Liu and Qing Wang assembled the genomes and conducted the genomics analysis. Tianming Lan coordinated the genomic analysis. Tianming Lan and Haimeng Li wrote the manuscript. Sunil Kumar Sahu, Minhui Shi, and Boyang Liu reviewed and edited the manuscript. Tianming Lan and Yan Hua made important contributions to the revision of the manuscript. All the authors read and approved the final manuscript.

Competing interests

The authors declare no competing interests.

Acknowledgments

This study was supported by the National Key Program of Research and Development, Ministry of Science and Technology (Grant No. 2022YFF1301500) and the Guangdong Provincial Key Laboratory of Genome Read and Write (Grant No. 2017B030301011). This work was also supported by China National GeneBank (CNGB). We thank Kinanti Seraphina Larasati and Shiqing Wang for their help in reviewing and polishing the language. We thank Hui Liu and Tian Xia from the Shenzhen Safari Park Co., Ltd. for assisting with sample collection. Finally, we thank all the researchers (Shiqing Wang, Dongyi Yang, Jieyao Yu, Jiale Fan, Yuting Huang, Yingna Zhou, Tianlu Liu, Jiatong Cheng, Chen Lin and Shiyu Liu) involved in sample collection, genome sequencing and analysis.

References

1. Venter JC, Adams MD, Myers EW, Li PW, Mural RJ, Sutton GG, et al. The sequence of the human genome. Science. 2001;291 5507:1304-51. doi:10.1126/science.1058040.
2. Nurk S, Koren S, Rhie A, Rautiainen M, Bzikadze AV, Mikheenko A, et al. The complete sequence of a human genome. Science. 2022;376 6588:44-53. doi:10.1126/science.abj6987.
3. Gibbs RA. The Human Genome Project changed everything. Nature Reviews Genetics. 2020;21 10:575-6. doi:10.1038/s41576-020-0275-3.
4. Hood L and Rowen L. The human genome project: big science transforms biology and medicine. Genome Medicine. 2013;5 9:79. doi:10.1186/gm483.
5. Genomes Project Consortium. A global reference for human genetic variation. Nature. 2015;526 7571:68-

74. doi:10.1038/nature15393.
6. De Coster W, De Rijk P, De Roeck A, De Pooter T, D'Hert S, Strazisar M, et al. Structural variants identified by Oxford Nanopore PromethION sequencing of the human genome. Genome research. 2019;29 7:1178-87. doi:10.1101/gr.244939.118.
7. Bredemeyer KR, Hillier L, Harris AJ, Hughes GM, Foley NM, Lawless C, et al. Single-haplotype comparative genomics provides insights into lineage-specific structural variation during cat evolution. Nature genetics. 2023;55 11:1953-63. doi:10.1038/s41588-023-01548-y.
8. Amarasinghe SL, Su S, Dong X, Zappia L, Ritchie ME and Gouil Q. Opportunities and challenges in long-read sequencing data analysis. Genome biology. 2020;21 1:30. doi:10.1186/s13059-020-1935-5.
9. Bentley DR, Balasubramanian S, Swerdlow HP, Smith GP, Milton J, Brown CG, et al. Accurate whole human genome sequencing using reversible terminator chemistry. Nature. 2008;456 7218:53-9. doi:10.1038/nature07517.
10. Rothberg JM, Hinz W, Rearick TM, Schultz J, Mileski W, Davey M, et al. An integrated semiconductor device enabling non-optical genome sequencing. Nature. 2011;475 7356:348-52. doi:10.1038/nature10242.
11. Eid J, Fehr A, Gray J, Luong K, Lyle J, Otto G, et al. Real-time DNA sequencing from single polymerase molecules. Science. 2009;323 5910:133-8. doi:10.1126/science.1162986.
12. Jain M, Olsen HE, Paten B and Akeson M. The Oxford Nanopore MinION: delivery of nanopore sequencing to the genomics community. Genome biology. 2016;17 1:239. doi:10.1186/s13059-016-1103-0.
13. Rhie A, McCarthy SA, Fedrigo O, Damas J, Formenti G, Koren S, et al. Towards complete and error-free genome assemblies of all vertebrate species. Nature. 2021;592 7856:737-46. doi:10.1038/s41586-021-03451-0.
14. Kolora SRR, Owens GL, Vazquez JM, Stubbs A, Chatla K, Jainese C, et al. Origins and evolution of extreme life span in Pacific Ocean rockfishes. Science. 2021;374 6569:842-7. doi:10.1126/science.abg5332.
15. Wang K, Wang J, Zhu C, Yang L, Ren Y, Ruan J, et al. African lungfish genome sheds light on the vertebrate water-to-land transition. Cell. 2021;184 5:1362-76 e18. doi:10.1016/j.cell.2021.01.047.
16. Bi X, Wang K, Yang L, Pan H, Jiang H, Wei Q, et al. Tracing the genetic footprints of vertebrate landing in non-teleost ray-finned fishes. Cell. 2021;184 5:1377-91 e14. doi:10.1016/j.cell.2021.01.046.
17. Lan T, Li H, Yang S, Shi M, Han L, Sahu SK, et al. The chromosome-scale genome of the raccoon dog: Insights into its evolutionary characteristics. iScience. 2022;25 10:105117. doi:10.1016/j.isci.2022.105117.
18. Formenti G, Theissinger K, Fernandes C, Bista I, Bombarely A, Bleidorn C, et al. The era of reference genomes in conservation genomics. Trends in ecology & evolution. 2022;37 3:197-202. doi:10.1016/j.tree.2021.11.008.
19. Kitts PA, Church DM, Thibaud-Nissen F, Choi J, Hem V, Sapojnikov V, et al. Assembly: a resource for assembled genomes at NCBI. Nucleic acids research. 2016;44 D1:D73-80. doi:10.1093/nar/gkv1226.
20. Ouborg NJ, Pertoldi C, Loeschcke V, Bijlsma RK and Hedrick PW. Conservation genetics in transition to conservation genomics. Trends in genetics : TIG. 2010;26 4:177-87. doi:10.1016/j.tig.2010.01.001.
21. Brandies P, Peel E, Hogg CJ and Belov K. The Value of Reference Genomes in the Conservation of Threatened Species. Genes. 2019;10 11 doi:10.3390/genes10110846.
22. Frankham R. Genetic rescue of small inbred populations: meta-analysis reveals large and consistent benefits of gene flow. Molecular ecology. 2015;24 11:2610-8. doi:10.1111/mec.13139.
23. Weeks AR, Heinze D, Perrin L, Stoklosa J, Hoffmann AA, van Rooyen A, et al. Genetic rescue increases

fitness and aids rapid recovery of an endangered marsupial population. Nature communications. 2017;8 1:1071. doi:10.1038/s41467-017-01182-3.

24. Khan A, Patel K, Shukla H, Viswanathan A, van der Valk T, Borthakur U, et al. Genomic evidence for inbreeding depression and purging of deleterious genetic variation in Indian tigers. Proceedings of the National Academy of Sciences of the United States of America. 2021;118 49 doi:10.1073/pnas.2023018118.

25. Hu JY, Hao ZQ, Frantz L, Wu SF, Chen W, Jiang YF, et al. Genomic consequences of population decline in critically endangered pangolins and their demographic histories. National science review. 2020;7 4:798-814. doi:10.1093/nsr/nwaa031.

26. von Seth J, Dussex N, Diez-Del-Molino D, van der Valk T, Kutschera VE, Kierczak M, et al. Genomic insights into the conservation status of the world's last remaining Sumatran rhinoceros populations. Nature communications. 2021;12 1:2393. doi:10.1038/s41467-021-22386-8.

27. Xue Y, Prado-Martinez J, Sudmant PH, Narasimhan V, Ayub Q, Szpak M, et al. Mountain gorilla genomes reveal the impact of long-term population decline and inbreeding. Science. 2015;348 6231:242-5. doi:10.1126/science.aaa3952.

28. Nicolas D, Tom vdV, Hernán E. M, Christopher W. W, David D-d-M, Johanna vS, et al. Population genomics of the critically endangered kākāpō. Cell Genomics. 2021;1 1:100002. doi:10.1016/j.xgen.2021.100002.

29. Saremi NF, Supple MA, Byrne A, Cahill JA, Coutinho LL, Dalen L, et al. Puma genomes from North and South America provide insights into the genomic consequences of inbreeding. Nature communications. 2019;10 1:4769. doi:10.1038/s41467-019-12741-1.

30. Xie HX, Liang XX, Chen ZQ, Li WM, Mi CR, Li M, et al. Ancient Demographics Determine the Effectiveness of Genetic Purging in Endangered Lizards. Molecular biology and evolution. 2022;39 1 doi:10.1093/molbev/msab359.

31. Hua L, Gong S, Wang F, Li W, Ge Y, Li X, et al. Captive breeding of pangolins: current status, problems and future prospects. ZooKeys. 2015; 507:99-114. doi:10.3897/zookeys.507.6970.

32. Kondrashov P and Agadjanian AK. A nearly complete skeleton ofErnanodon(Mammalia, Palaeanodonta) from Mongolia: morphofunctional analysis. Journal of Vertebrate Paleontology. 2012;32 5:983-1001. doi:10.1080/02724634.2012.694319.

33. Choo SW, Rayko M, Tan TK, Hari R, Komissarov A, Wee WY, et al. Pangolin genomes and the evolution of mammalian scales and immunity. Genome research. 2016;26 10:1312-22.

34. Ferreira-Cardoso S, Billet G, Gaubert P, Delsuc F and Hautier L. Skull shape variation in extant pangolins (Pholidota: Manidae): allometric patterns and systematic implications. Zoological Journal of the Linnean Society. 2019; doi:10.1093/zoolinnean/zlz096.

35. Heinrich S, Wittman TA, Ross JV, Shepherd CR, Challender DWS and Cassey P. THE GLOBAL TRAFFICKING OF PANGOLINS: A comprehensive summary of seizures and trafficking routes from 2010–2015. Petaling Jaya: TRAFFIC, Southeast Asia Regional Office. 2017.

36. Zhang F, Wu S and Cen P. The past, present and future of the pangolin in Mainland China. Global Ecology and Conservation. 2022;33:e01995. doi:10.1016/j.gecco.2021.e01995.

37. Challender DWS, Harrop SR and MacMillan DC. Understanding markets to conserve trade-threatened species in CITES. Biological Conservation. 2015;187:249-59. doi:10.1016/j.biocon.2015.04.015.

38. Nijman V, Zhang MX and Shepherd CR. Pangolin trade in the Mong La wildlife market and the role of Myanmar in the smuggling of pangolins into China. Global Ecology and Conservation. 2016;5:118-26. doi:10.1016/j.gecco.2015.12.003.

39. Cheng W, Xing S and Bonebrake TC. Recent Pangolin Seizures in China Reveal Priority Areas for Intervention. Conservation Letters. 2017;10 6:757-64. doi:10.1111/conl.12339.

40. Zhang F, Yu Y, Wu S, Mahmood A, Yu J and Min Y. Reducing Pangolin Demand by Understanding Motivations for Human Consumption in Guangdong, China. Frontiers in Ecology and Evolution. 2020;8 doi:10.3389/fevo.2020.574161.

41. Wang Q, Lan T, Li H, Sahu SK, Shi M, Zhu Y, et al. Whole-genome resequencing of Chinese pangolins reveals a population structure and provides insights into their conservation. Communications biology. 2022;5 1:821. doi:10.1038/s42003-022-03757-3.

42. Cheng H, Concepcion GT, Feng X, Zhang H and Li H. Haplotype-resolved de novo assembly using phased assembly graphs with hifiasm. Nature methods. 2021;18 2:170-5.

43. Cao P, Dai Q, Deng C, Zhao X, Qin S, Yang J, et al. Genome-wide signatures of mammalian skin covering evolution. Science China Life Sciences. 2021;64 10:1765-80.

44. Damas J, Corbo M, Kim J, Turner-Maier J, Farré M, Larkin DM, et al. Evolution of the ancestral mammalian karyotype and syntenic regions. Proceedings of the National Academy of Sciences. 2022;119 40:e2209139119.

45. Heighton SP, Allio R, Murienne J, Salmona J, Meng H, Scornavacca C, et al. Pangolin genomes offer key insights and resources for the world's most trafficked wild mammals. bioRxiv. 2023; doi:10.1101/2023.02.16.528682.

46. Yan D, Luo X, Tang J, Xu S, Huang K, Wang X, et al. High-Quality Genomes of Pangolins: Insights into the Molecular Basis of Scale Formation and Adaption to Myrmecophagous Diet. Molecular biology and evolution. 2023;40 1 doi:10.1093/molbev/msac262.

47. Nie W, Wang J, Su W, Wang Y and Yang F. Chromosomal rearrangements underlying karyotype differences between Chinese pangolin (Manis pentadactyla) and Malayan pangolin (Manis javanica) revealed by chromosome painting. Chromosome research : an international journal on the molecular, supramolecular and evolutionary aspects of chromosome biology. 2009;17 3:321-9. doi:10.1007/s10577-009-9027-0.

48. Qi W, Lim YW, Patrignani A, Schlapfer P, Bratus-Neuenschwander A, Gruter S, et al. The haplotype-resolved chromosome pairs of a heterozygous diploid African cassava cultivar reveal novel pan-genome and allele-specific transcriptome features. GigaScience. 2022;11:giac028. doi:10.1093/gigascience/giac028.

49. Kim H-K, Ham KA, Lee S-W, Choi HS, Kim H-S, Kim HK, et al. Biallelic deletion of pxdn in mice leads to anophthalmia and severe eye malformation. International Journal of Molecular Sciences. 2019;20 24:6144.

50. Segarra NG, Ballhausen D, Crawford H, Perreau M, Campos-Xavier B, van Spaendonck-Zwarts K, et al. NBAS mutations cause a multisystem disorder involving bone, connective tissue, liver, immune system, and retina. American Journal of Medical Genetics Part A. 2015;167 12:2902-12. doi:10.1002/ajmg.a.37338.

51. Kumamaru E, Kuo C-H, Fujimoto T, Kohama K, Zeng L-H, Taira E, et al. Reticulon3 expression in rat optic and olfactory systems. Neuroscience letters. 2004;356 1:17-20. doi:10.1016/j.neulet.2003.11.009.

52. Henn BM, Botigué LR, Bustamante CD, Clark AG and Gravel S. Estimating the mutation load in human genomes. Nature Reviews Genetics. 2015;16 6:333-43. doi:10.1038/nrg3931.

53. Low WY, Tearle R, Liu R, Koren S, Rhie A, Bickhart DM, et al. Haplotype-resolved genomes provide insights into structural variation and gene content in Angus and Brahman cattle. Nature communications. 2020;11 1:2071. doi:10.1038/s41467-020-15848-y.

54. Sun H, Jiao WB, Krause K, Campoy JA, Goel M, Folz-Donahue K, et al. Chromosome-scale and haplotype-resolved genome assembly of a tetraploid potato cultivar. Nature genetics. 2022;54 3:342-8. doi:10.1038/s41588-022-01015-0.

55. Zhang L, Lan T, Lin C, Fu W, Yuan Y, Lin K, et al. Chromosome-scale genomes reveal genomic consequences of inbreeding in the South China tiger: A comparative study with the Amur tiger. Molecular ecology resources. 2022; doi:10.1111/1755-0998.13669.

56. Yang S, Lan T, Zhang Y, Wang Q, Li H, Dussex N, et al. Genomic investigation of the Chinese alligator reveals wild-extinct genetic diversity and genomic consequences of their continuous decline. Molecular ecology resources. 2022; doi:10.1111/1755-0998.13702.

57. Wang Q, Lan T, Li H, Sahu SK, Shi M, Zhu Y, et al. Whole-genome resequencing of Chinese pangolins reveals a population structure and provides insights into their conservation. Communications Biology. 2022;5 1:821. doi:10.1038/s42003-022-03757-3.

58. Zhao S, Zheng P, Dong S, Zhan X, Wu Q, Guo X, et al. Whole-genome sequencing of giant pandas provides insights into demographic history and local adaptation. Nature genetics. 2013;45 1:67-71. doi:10.1038/ng.2494.

59. Kuang WM, Ming C, Li HP, Wu H, Frantz L, Roos C, et al. The Origin and Population History of the Endangered Golden Snub-Nosed Monkey (Rhinopithecus roxellana). Molecular biology and evolution. 2019;36 3:487-99. doi:10.1093/molbev/msy220.

60. Hu J, Roos C, Lv X, Kuang W and Yu L. Molecular Genetics Supports a Potential Fifth Asian Pangolin Species (Mammalia, Pholidota, Manis). Zoological science. 2020;37 6:538-43. doi:10.2108/zs200084.

61. Guang X, Lan T, Wan Q-H, Huang Y, Li H, Zhang M, et al. Chromosome-scale genomes provide new insights into subspecies divergence and evolutionary characteristics of the giant panda. Science Bulletin. 2021;66 19:2002-13. doi:10.1016/j.scib.2021.02.002.

62. Pecnerova P, Garcia-Erill G, Liu X, Nursyifa C, Waples RK, Santander CG, et al. High genetic diversity and low differentiation reflect the ecological versatility of the African leopard. Current biology : CB. 2021;31 9:1862-71 e5. doi:10.1016/j.cub.2021.01.064.

63. Carneiro M, Albert FW, Afonso S, Pereira RJ, Burbano H, Campos R, et al. The genomic architecture of population divergence between subspecies of the European rabbit. PLoS genetics. 2014;10 8:e1003519. doi:10.1371/journal.pgen.1003519.

64. Liu B, Shi Y, Yuan J, Hu X, Zhang H, Li N, et al. Estimation of genomic characteristics by analyzing k-mer frequency in de novo genome projects. arXiv: Genomics. 2013.

65. Guan D, McCarthy SA, Wood J, Howe K, Wang Y and Durbin R. Identifying and removing haplotypic duplication in primary genome assemblies. Bioinformatics. 2020;36 9:2896-8.

66. Li H. Aligning sequence reads, clone sequences and assembly contigs with BWA-MEM. arXiv:13033997 [q-bioGN]. 2013;0 0:3.

67. Li H and Durbin R. Fast and accurate long-read alignment with Burrows–Wheeler transform. Bioinformatics. 2010;26 5:589-95.

68. Durand NC, Shamim MS, Machol I, Rao SS, Huntley MH, Lander ES, et al. Juicer provides a one-click system for analyzing loop-resolution Hi-C experiments. Cell systems. 2016;3 1:95-8.

69. Dudchenko O, Batra SS, Omer AD, Nyquist SK, Hoeger M, Durand NC, et al. De novo assembly of the Aedes aegypti genome using Hi-C yields chromosome-length scaffolds. Science. 2017;356 6333:92-5.

70. Manni M, Berkeley MR, Seppey M, Simão FA, Zdobnov EM and Kelley J. BUSCO Update: Novel and Streamlined Workflows along with Broader and Deeper Phylogenetic Coverage for Scoring of Eukaryotic, Prokaryotic, and Viral Genomes. Molecular biology and evolution. 2021;38 10:4647-54.

doi:10.1093/molbev/msab199.
71. Rhie A, Walenz BP, Koren S and Phillippy AM. Merqury: reference-free quality, completeness, and phasing assessment for genome assemblies. Genome biology. 2020;21 1 doi:10.1186/s13059-020-02134-9.
72. Marçais G, Delcher AL, Phillippy AM, Coston R, Salzberg SL and Zimin A. MUMmer4: A fast and versatile genome alignment system. PLoS computational biology. 2018;14 1:e1005944.
73. Xu Z and Wang H. LTR_FINDER: an efficient tool for the prediction of full-length LTR retrotransposons. Nucleic Acids Res. 2007;35 Web Server issue:W265-8. doi:10.1093/nar/gkm286.
74. Flynn JM, Hubley R, Goubert C, Rosen J, Clark AG, Feschotte C, et al. RepeatModeler2 for automated genomic discovery of transposable element families. Proceedings of the National Academy of Sciences of the United States of America. 2020;117 17:9451-7. doi:10.1073/pnas.1921046117.
75. Chen N. Using Repeat Masker to identify repetitive elements in genomic sequences. Current protocols in bioinformatics. 2004;5 1:4.10. 1-4.. 4.
76. Benson G. Tandem repeats finder: a program to analyze DNA sequences. Nucleic acids research. 1999;27 2:573-80. doi:10.1093/nar/27.2.573.
77. Stanke M, Steinkamp R, Waack S and Morgenstern B. AUGUSTUS: a web server for gene finding in eukaryotes. Nucleic acids research. 2004;32 Web Server issue:W309-12. doi:10.1093/nar/gkh379.
78. Majoros WH, Pertea M and Salzberg SL. TigrScan and GlimmerHMM: two open source ab initio eukaryotic gene-finders. Bioinformatics. 2004;20 16:2878-9. doi:10.1093/bioinformatics/bth315.
79. Korf I. Gene finding in novel genomes. BMC bioinformatics. 2004;5:59. doi:10.1186/1471-2105-5-59.
80. Kim D, Langmead B and Salzberg SL. HISAT: a fast spliced aligner with low memory requirements. Nature methods. 2015;12 4:357-60.
81. Pertea M, Pertea GM, Antonescu CM, Chang T-C, Mendell JT and Salzberg SL. StringTie enables improved reconstruction of a transcriptome from RNA-seq reads. Nature biotechnology. 2015;33 3:290-5.
82. Mount DW. Using the Basic Local Alignment Search Tool (BLAST). CSH protocols. 2007;2007:pdb top17. doi:10.1101/pdb.top17.
83. Campbell MS, Holt C, Moore B and Yandell M. Genome Annotation and Curation Using MAKER and MAKER-P. Current protocols in bioinformatics. 2014;48:4 11 1-39. doi:10.1002/0471250953.bi0411s48.
84. Jones P, Binns D, Chang H-Y, Fraser M, Li W, McAnulla C, et al. InterProScan 5: genome-scale protein function classification. Bioinformatics. 2014;30 9:1236-40. doi:10.1093/bioinformatics/btu031.
85. Lowe TM and Eddy SR. tRNAscan-SE: a program for improved detection of transfer RNA genes in genomic sequence. Nucleic acids research. 1997;25 5:955-64.
86. Goel M, Sun H, Jiao W-B and Schneeberger K. SyRI: finding genomic rearrangements and local sequence differences from whole-genome assemblies. Genome biology. 2019;20 1:1-13.
87. Robinson JT, Thorvaldsdóttir H, Turner D and Mesirov JP. igv. js: an embeddable JavaScript implementation of the Integrative Genomics Viewer (IGV). BioRxiv. 2022:2020.05. 03.075499.
88. Freed D, Aldana R, Weber JA and Edwards JS. The Sentieon Genomics Tools-A fast and accurate solution to variant calling from next-generation sequence data. BioRxiv. 2017:115717.
89. Purcell S, Neale B, Todd-Brown K, Thomas L, Ferreira MA, Bender D, et al. PLINK: a tool set for whole-genome association and population-based linkage analyses. The American journal of human genetics. 2007;81 3:559-75.
90. Yang J, Lee SH, Goddard ME and Visscher PM. GCTA: a tool for genome-wide complex trait analysis. The American Journal of Human Genetics. 2011;88 1:76-82.
91. Ortiz E. vcf2phylip v2. 0: convert a VCF matrix into several matrix formats for phylogenetic analysis.

URL https://doi org/105281/zenodo. 2019;2540861.

92. Darriba D, Taboada GL, Doallo R and Posada D. jModelTest 2: more models, new heuristics and parallel computing. Nature methods. 2012;9 8:772. doi:10.1038/nmeth.2109.

93. Nguyen LT, Schmidt HA, von Haeseler A and Minh BQ. IQ-TREE: a fast and effective stochastic algorithm for estimating maximum-likelihood phylogenies. Molecular biology and evolution. 2015;32 1:268-74. doi:10.1093/molbev/msu300.

94. Alexander DH, Novembre J and Lange K. Fast model-based estimation of ancestry in unrelated individuals. Genome research. 2009;19 9:1655-64.

95. Terhorst J, Kamm JA and Song YS. Robust and scalable inference of population history from hundreds of unphased whole genomes. Nature genetics. 2017;49 2:303-9.

96. Schiffels S and Durbin R. Inferring human population size and separation history from multiple genome sequences. Nature genetics. 2014;46 8:919-25. doi:10.1038/ng.3015.

97. Browning SR and Browning BL. Rapid and accurate haplotype phasing and missing-data inference for whole-genome association studies by use of localized haplotype clustering. The American Journal of Human Genetics. 2007;81 5:1084-97.

98. Dobrynin P, Liu S, Tamazian G, Xiong Z, Yurchenko AA, Krasheninnikova K, et al. Genomic legacy of the African cheetah, Acinonyx jubatus. Genome biology. 2015;16 1:1-20.

99. Danecek P, Auton A, Abecasis G, Albers CA, Banks E, DePristo MA, et al. The variant call format and VCFtools. Bioinformatics. 2011;27 15:2156-8.

100. Wang K, Li M and Hakonarson H. ANNOVAR: functional annotation of genetic variants from high-throughput sequencing data. Nucleic acids research. 2010;38 16:e164-e.

101. Cingolani P, Platts A, Wang LL, Coon M, Nguyen T, Wang L, et al. A program for annotating and predicting the effects of single nucleotide polymorphisms, SnpEff: SNPs in the genome of Drosophila melanogaster strain w1118; iso-2; iso-3. Fly. 2012;6 2:80-92.

102. Grantham R. Amino acid difference formula to help explain protein evolution. science. 1974;185 4154:862-4.

103. Feng S, Fang Q, Barnett R, Li C, Han S, Kuhlwilm M, et al. The genomic footprints of the fall and recovery of the crested ibis. Current Biology. 2019;29 2:340-9. e7.

104. Davydov EV, Goode DL, Sirota M, Cooper GM, Sidow A and Batzoglou S. Identifying a high fraction of the human genome to be under selective constraint using GERP++. PLoS computational biology. 2010;6 12:e1001025.

105. Guo X, Chen F, Gao F, Li L, Liu K, You L, et al. CNSA: a data repository for archiving omics data. Database. 2020;2020 doi:10.1093/database/baaa055.

106. Chen F, You L, Yang F, Wang L, Guo X, Gao F, et al. CNGBdb: China National GeneBank DataBase. Hereditas (Beijing). 2020;42:799-809. doi:10.16288/j.yczz.20-080.

Figure 1

Click here to access/download;Figure;FIG1.pdf

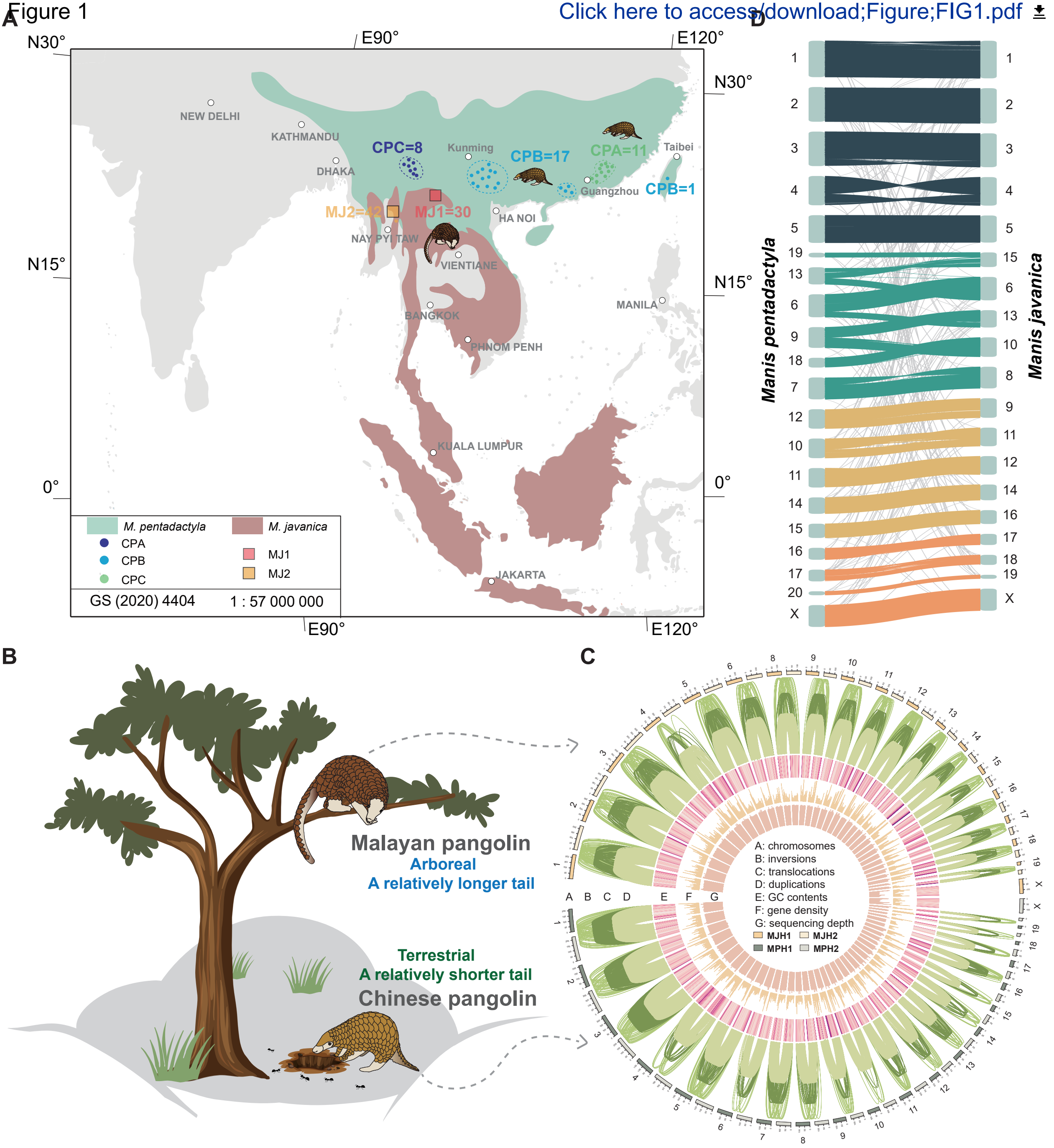

Figure 2

Click here to access/download;Figure;FIG2.pdf

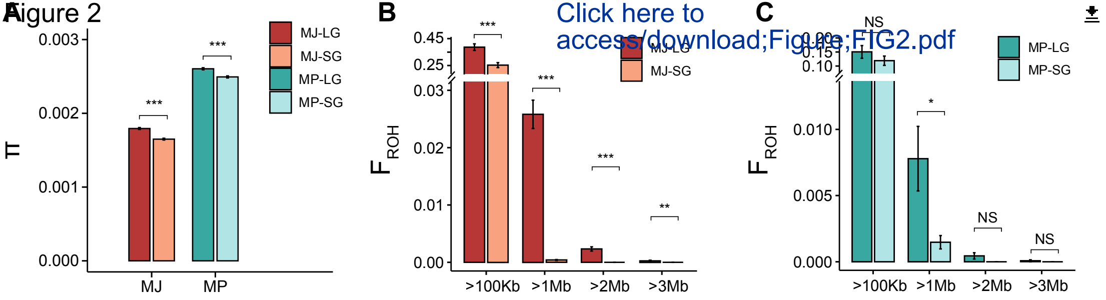

Figure 3

Click here to access/download;Figure;FIG3.pdf

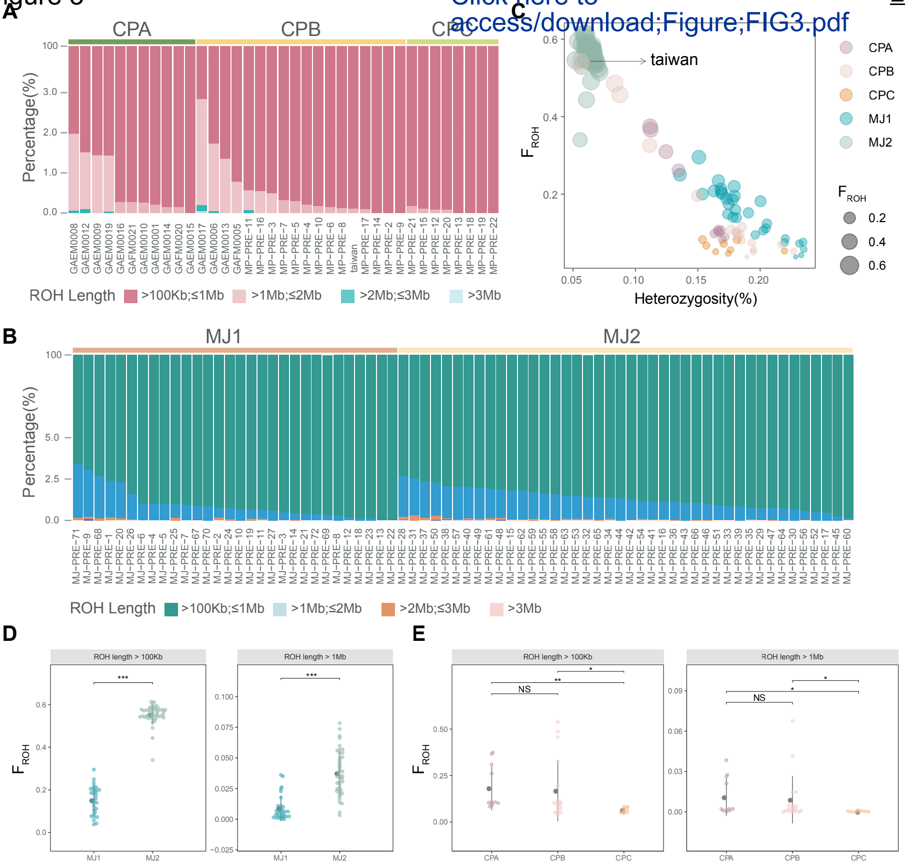

Click here to access/download;Figure;FIG4.pdf

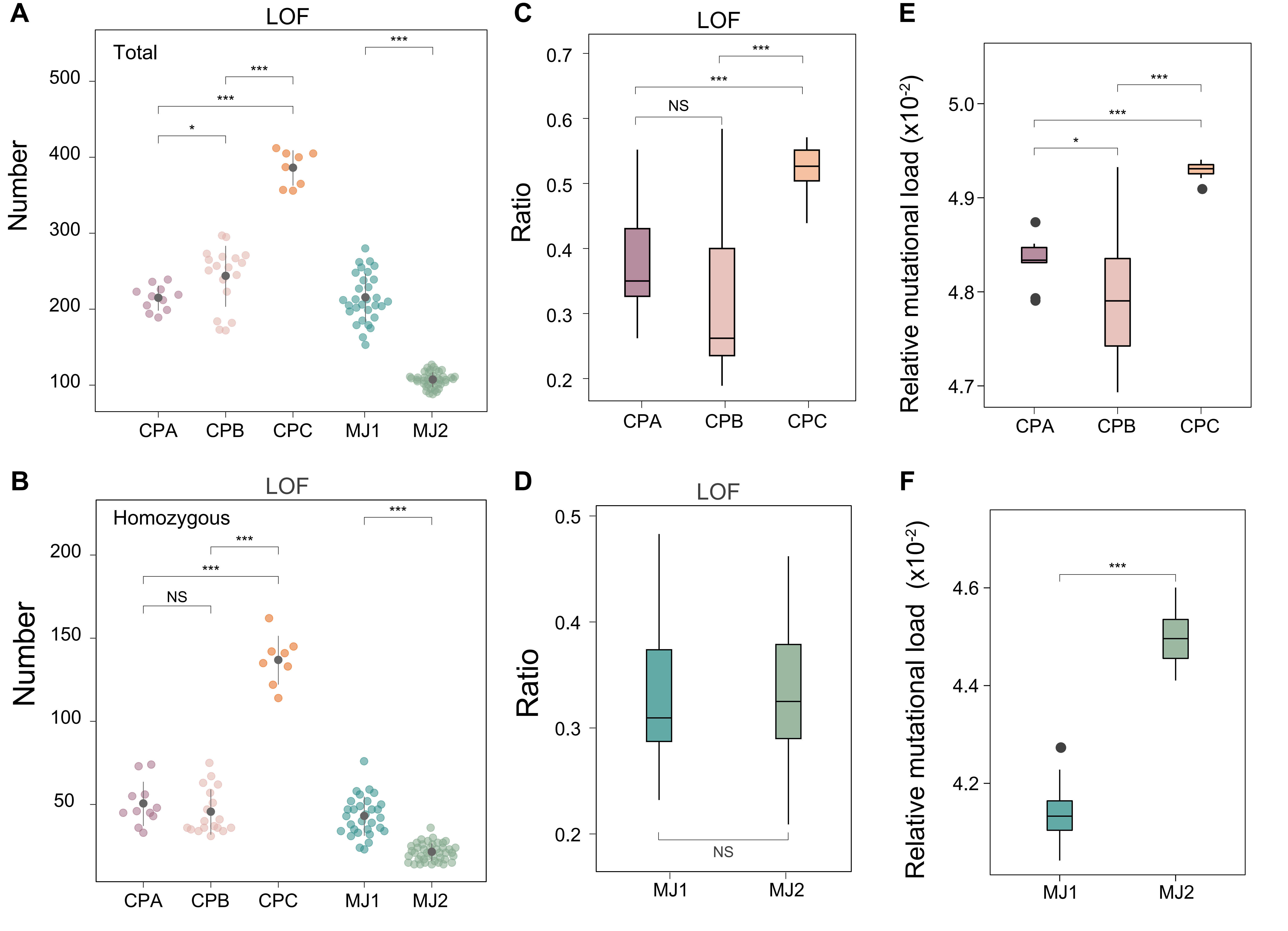

Figure 5

Click here to access/download;Figure;FIG5.pdf

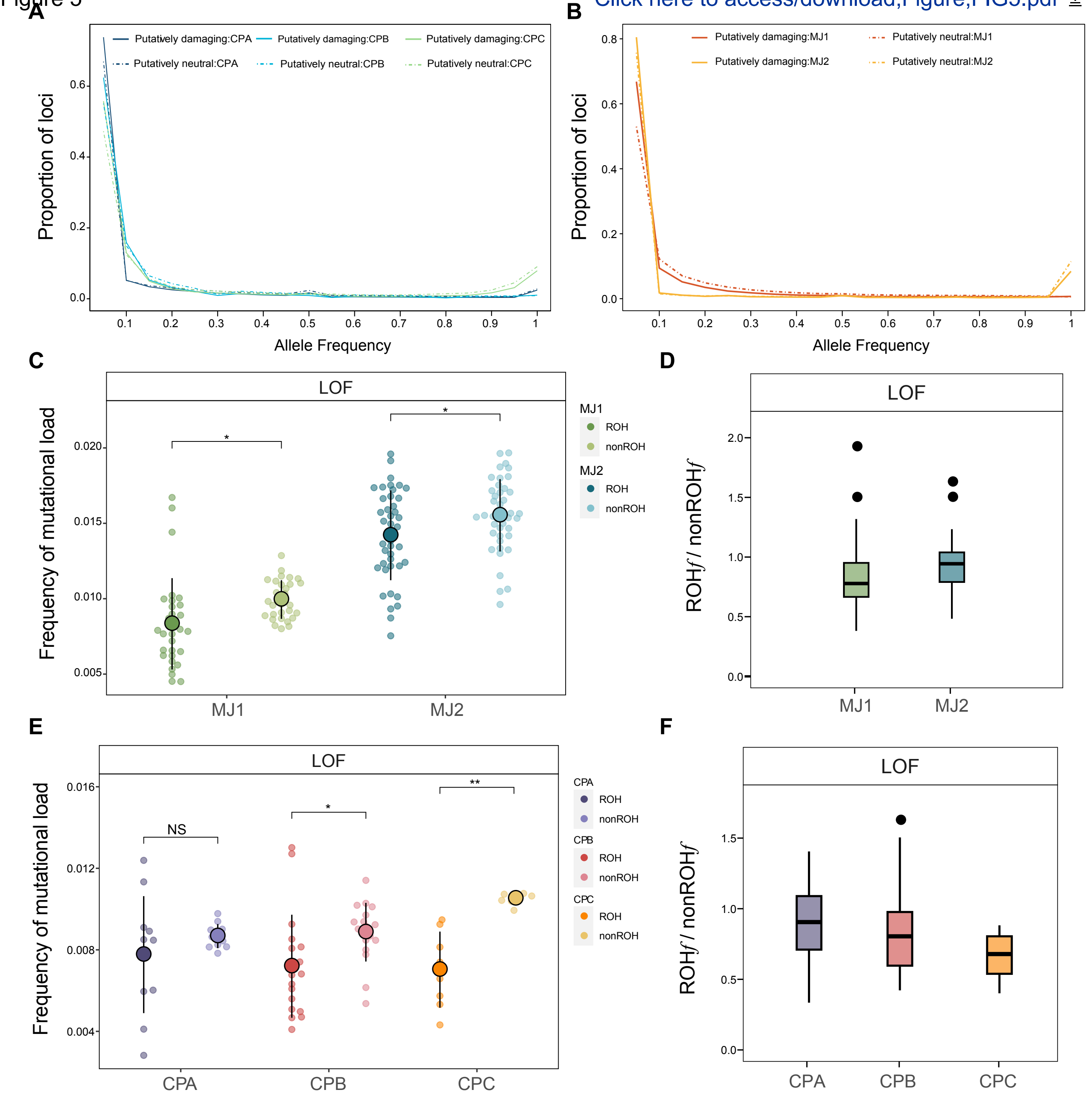

Click here to access/download
Supplementary Material
Supplemental Information.docx

Click here to access/download
Supplementary Material
Supplementary tables.xlsx
