# Supplementary material for: Enhancing inbreeding estimation and global conservation insights through chromosome-level assemblies of the Chinese and Malayan pangolin
Source: Gigascience. 2025 Feb 14;14:giaf003. doi: 10.1093/gigascience/giaf003 (PMC11825179; doi:10.1093/gigascience/giaf003)
Supplement: giaf003_GIGA-D-24-00182_Revision_1 [file giaf003_giga-d-24-00182_revision_1.pdf]

GigaScience

Enhancing inbreeding estimation and global conservation insights through HiFi assemblies of the Chinese and Malayan pangolin

--Manuscript Draft--

**Manuscript Number:**	GIGA-D-24-00182R1
**Full Title:**	Enhancing inbreeding estimation and global conservation insights through HiFi assemblies of the Chinese and Malayan pangolin
**Article Type:**	Research
**Funding Information:**	National Key Program of Research and Development, Ministry of Science and Technology (No. 2022YFF1301500) — Not applicable<br>the Guangdong Provincial Key Laboratory of Genome Read and Write (No. 2017B030301011) — Not applicable<br>Start-up Scientific Foundation of Northeast Forestry University (60201524043) — Prof Tianming Lan
**Abstract:**	A high-quality reference genome coupled with resequencing data is a promising strategy to address issues in conservation genomics. This has greatly enhanced the development of conservation plans for endangered species. Pangolins are fascinating animals with a variety of unique features,. Unfortunately, they are the most trafficked wild animal in the world. In this study, we assembled a chromosome-scale genome with HiFi long reads for the Chinese and Malayan pangolin and provided two new representative reference genomes for the pangolin species. We found a great improvement in the evaluation of genetic diversity and inbreeding based on these high-quality genomes and obtained different results for the detection of genome-wide extinction risks compared with genomes assembled using short reads. Moderate inbreeding and genetic diversity were reverified in these two pangolin species, except for one Malayan pangolin population with high inbreeding and low genetic diversity. Moreover, we identified a much higher inbreeding level (FROH=0.54) in the Chinese pangolin individual from Taiwan Province compared with that from mainland China, but more than 99.6% runs of homozygosity (ROH) fragments were restricted to less than 1Mb, indicating that the high FROH in Taiwan Chinese pangolins may have accumulated from historical inbreeding events. Furthermore, our study is the first to detect relatively mild genetic purging in pangolin populations. These two high-quality reference genomes will provide valuable genetic resources for future studies and contribute to the protection and conservation of pangolins.
**Corresponding Author:**	Haimeng Li<br>Northeast Forestry University<br>Harbin, CHINA
**Corresponding Author Secondary Information:**	
**Corresponding Author's Institution:**	Northeast Forestry University
**Corresponding Author's Secondary Institution:**	
**First Author:**	Tianming Lan
**First Author Secondary Information:**	
**Order of Authors:**	Tianming Lan<br>Yinping Tian<br>Minhui Shi<br>Boyang Liu<br>Yu Lin

Powered by Editorial Manager® and ProduXion Manager® from Aries Systems Corporation

	Yanling Xia
	Yue Ma
	Sahu Sunil Kumar
	Qing Wang
	Jun Li
	Jin Chen
	Fanghui Hou
	Chuanling Yin
	Kai Wang
	Tengcheng Que
	Wenjian Liu
	Huan Liu
	Haimeng Li
	Yan Hua
**Order of Authors Secondary Information:**	
**Response to Reviewers:**	Dear Editor and Reviewers,<br>Thank you for the many insightful comments and suggestions. We have made revisions to address all the comments. We have carefully considered each of your comments and have made the necessary revisions to address them thoroughly. Below, you will find a detailed list of our responses to the specific comments you raised:<br>Reviewer #1: The manuscript describes two novel VGP style genome assemblies of Malayan and Chinese pangolins. New assemblies provide a significant improvement to previously published short read references. Sequenced data and software used for assembly are reasonable. In addition to the genome assembly, the authors performed some population analysis using previously published resequencing data. I see no issues with the results of this part of the manuscript, but the discussion (comparison with previously published results) should be enhanced (as a part of review process). Title of the manuscript implies that such a comprehensive comparison is inside the text.<br>Major comments.<br>Q1.As I understood, no parental data was generated for genome assembly, and only HiFi + HiC data were generated from the reference individuals. It means that resulting assemblies are not completely haplotype resolved. Combination of HiFi and HiC allows to (nearly) completely resolve haplotypes only at the level of chromosomes, but chromosomal scaffolds are still mixed between haplotypes. It is a very common mistake to treat such an assembly as haplotype- resolved. I encourage authors to somehow underline it in the text, for example by adding "at chromosomal level" where it is necessary<br>Response: Thank you for pointing out this. Yes, our study did not generate parental data for genome assembly, we only utilized HiFi and HiC data from the same individual for genome assembly. We agree that the combination of HiFi and HiC data allows for nearly complete resolution of haplotypes at the chromosomal level, the chromosomal scaffolds are still mixed between haplotypes[1]. We appreciate your suggestion and will emphasize this point in the revised manuscript by including a clarification in the text, such as specifying “at the chromosomal level” where appropriate, to avoid any misinterpretation. Line 100.<br>Q2. QV scores look relatively low (<50). I would expect something like 55-60+ for such input data.<br>Response: Thank you for this comment. In the original version of this manuscript, we utilized short reads sequencing data to calculate the QV scores (MJ: 48.56; MJ: 49.63) by the Merqury software. In the revised manuscript, we polished the assemblies by HiFi long reads and WGS short reads, and used the high quality HiFi reads to calculate the QV scores by the Merqury software [3, 4]. The result showed that the QV score for the MP and MJ were 57.06 and 55.08, respectively, which means ~ 0.00019% and 0.00031% of base calling error rate, respectively, which could reflect a very high

accuracy for our genome assembly. In addition, we have conducted an extensive search of published papers describing genomes with QV analysis by the Merqury software. We found that a number of published genomes showed QV values ranging from the 30 to 50 [5-7], which means the error rate of the base calling ranged from 0.1% to 0.001%. Therefore, we agree with you, a QV score of 55-60+ would be much better than < 50 in our previously assembled genomes.
Q3. Figure 1C is very difficult to understand. I encourage author to remove it completely or do a linear plot (or set of them) similar to Figure 1D
Response: Thank you to point out this. We have moved Figure 1C to the supplementary materials (Fig S17a) and added a linear plot here, as you suggested (Fig S17b-d).
Q4. row 162-163: "All these SVs were validated by our assembled contigs (Supplementary Fig. S9)". Figure is very unclear, and a reader may expect some results to be present on the figure and not the methods. Also I would expect some inversions encompassing whole contigs (especially at the end of chromosomes, where coverage often drops and contigs are shorter). Such inversions might be an artifacts of the HiC scaffolding and are very common. Please confirm that you have found none of them.
Response: Thanks for your suggestion. In response to the issue with the unclear figure, we have added a result description in the supplementary Fig. S18. This figure is intended to illustrate the validation of SVs by contigs mapping. For the potential issue of inversions, especially at the end of chromosome, we have meticulously re-examined our data and have made the necessary revisions to the structural variant (SV) results, we have taken great care to ensure that the SVs are accurately represented and inversions that are potentially be artifacts of HiC scaffolding have been all corrected in our analysis (Table S21).

Response figure 1. Validation of structural variants through contig mapping. Contigs are mapped to both the haploid genomes to verify structural variants and visualized by the IGV software. (a) The correct structural variants between the haploid genomes. The breakpoints of the SVs could be covered by complete contigs in both of the haploid genomes. (b) The incorrect structural variants between the haploid genomes. The breakpoints of the SVs in one of the two haploid genomes could not be covered by the complete contig. In the IGV screenshot, the gray bar represents the contig spanning over the SVs, and the contig corresponding SV regions were marked as the red box. All structural variants have been verified, and several are randomly displayed here.
Q5. row 311-316. A bit of a strange wording. "Much better contiguity, which is … longer". Please, simplify. For example by just comparing N50s in the main text
Response: Thank you. We agree that comparing the N50 values would provide a clearer and more straightforward comparison. We've revised the description here to make it more readable. Please refer to the supplementary material, lines 42-48, for the updated information.
Q6. Manuscript lacks a comparison (and even mention of) with chromosome-length assemblies of malayan and Chinese pangolins generated by DNAzoo team (upgrade of two draft assemblies from NCBI). They are available at dnazoo.org. Such a comparison makes more sense than comparing with old drafts.
Response: Thank you for your reminder. We download the genomes of Malayan pangolin and Chinese pangolin from the DNAzoo website. And we added the comparison with all assemblies and presented the result in Supplementary Table S19. By this comparison, we found that the scaffold N50 of the genomes we assembled in this study are remarkably higher than those of the genomes published by DNAzoo. This comparison further showed the superiority of our assemblies in contiguity, thank you.
Q7. Paragraphs "Genome-wide extinction risks in pangolin populations" and "Novel implications for the global conservation of these two pangolin species" confuse me a bit. Authors state that they have not generated the resequencing data, but downloaded it from public databases. Dataset set includes data from three publications (references [25, 33, 57]). Majority of it (nearly all Malayan pangolin samples and >60% of Chinese pangolin) were analyzed in [25]. All Chinese pangolin samples were analyzed in [57]. Authors cite both publications several times, but text lacks a more detailed comparison between the manuscript's results and previously published ones. It is especially crucial and interesting for Chinese pangolin as there is a 100% overlap in samples with [57]. The title of the manuscript requires an extensive comparison with previous publications

	in all the discussion.<br>Response: We appreciate your comment and we agree. One of the most important points in this this manuscript is to discuss how a high-quality genome could improve the evaluation of genetic parameters in conservation genomics or in population genomics, particularly in evaluation of genetic diversity and inbreeding by screening ROH across the genome, because a reference genome with low genome contiguity cannot well support the detection of long fragments of runs of homozygosity (ROH):<br>1) We found much higher genetic diversity than that reported by[8, 9], indicating that we may have detected more variants across the genome based on high-quality reference genome assembled in this study, because HiFi long reads could span much more complex genomic regions[10] and generate longer contigs [11, 12], and many genomic regions that cannot be assembled by short reads can be assembled by long reads, and these regions may contain important variants.<br>2) The inbreeding level (ROH detection) detected based on the reference genome we assembled in this study was significantly higher than that identified using the short reads assembled genome, particularly for ROHs larger than 1Mb, which is largely due to that the short contigs in the reference genome cannot span over long ROH fragments. By comparing with previously published short-read assembled genomes, findings in this study could tell the reader the superiority of a reference genome with better contiguity on the detection of genetic diversity and ROH fragments.<br>In addition, we have incorporated comparisons of our results with the results published by other studies, which can be found in many places throughout the manuscript, particularly in the results section:<br>1)The trajectory of the change of effective population size of Malayan pangolins presented in this study is different from that reported by Hu et al. We inferred that the MjavA (MJA in Hu et al.) population experienced a continuous population decline and the population size was lower than that of the MjavB (MJB in Hu et al.) population within the most recent 10,000 years, which could be well matched with the lower genetic diversity and higher genome-wide inbreeding in the MjavA population. But in the previous report, Hu et al,. found that the population size of the MjavA was much greater than the MjavB population from 10 kya to 3.2 kya. This difference is largely due to the different methods we used, and we did not used the method in Hu et al. because we have compared these two methods in our previous study[13] and found that this method is not as stable as the method we used in our study.<br>2)The parameters used in the filtering of genome-wide SNPs in Hu et al,. is too strict, SNPs with a minor allele frequency less than 0.2 will be all removed from the data set, which will overestimate the inbreeding across the population by erroneously introducing long ROH fragments (>1Mb). And in our study, we found much less long ROH fragments (>1Mb) than that in the Hu et al,. even if we used HiFi genome for reference. The parameters used for identifying the ancestral state in Wang et al,. is relatively rough by considering alleles in the Malayan pangolin genome as the ancestry state. In this study, we used the more commonly used method to identify the ancestral allele, which is more reliable. Therefore, the accumulation of derived mutational load for the Chinese pangolin populations in our study is different from that in Wang et al. We added this comparison in the maintext.<br>3)The genetic purging is a very important issue in the conservation genomics, genetic purging always exists in a population under purifying selection[14]. In general, deleterious alleles affecting fitness and viability are initially rare and may be hidden in the heterozygous state, but are more easily to be cleared from the population when these deleterious alleles are exposed in a homozygous state (Purging). This is an indicator for the evaluation of the extinction risk of endangered species (Response figure 2). However, in previous study (Hu et al, Wang et al and Wei et al), they did not mention this process in pangolins. In this study we discussed genetic purging in both Chinese and Malayan pangolins.<br><br>Response figure 2. The relationship between fitness and inbreeding[15].<br>Minor comments:<br>Q8. row 36-44: first paragraph is redundant and can be safely removed. It describes very basic things and facts.<br>Response: Thank you for your comment. We removed this paragraph in the revised manuscript. Lines 41-49.<br>Q9. row 60: Pangolin is a prehistoric mammal = "prehistoric" doesn't look like a good term here. Maybe 'living fossil' is better<br>Response: Thank you and we revised. Line 67.

Powered by Editorial Manager® and ProduXion Manager® from Aries Systems Corporation

	Q10. row 81: remove 'recently introduced'<br>Response: Removed, thank you. Line 92.<br>Q11. row 82: "HiFi-specific assembler can generate high-quality and haplotype-resolved de novo assemblies" HiFi data itself (without HiC or parental data) can't produce haplotype-resolved assembly. Please, Correct<br>Response: Thank you for your careful reminder. We have modified the description to make this part more rigorous. Line 93.<br>Reviewer #2: This paper presents two new chromosome-level assemblies for two species of Pangolin, alongside some population genetic data from different populations of each. The quality of the genomes appears to be very high, and these data will indeed "provide valuable genomic resource for future studies on the protection and conservation for pangolins". However, this is Research Article and not a Data Note, and so it also needs a clear narrative relating to the biological insights and which aspects of the data are of most interest. This is currently hampered by numerous language errors, inconsistent formatting, and excessive reference to relatively uninteresting supplementary data. Having some clearer aims/questions beyond a better genome would be helpful in this regard.<br>If there is no clear message, the authors could consider a data note instead. Either way, I think a lot of the results should be consolidated and presented as Supplementary Results to emphasise the important/interesting data. There is also excessive comparison of results using their long-read and an unknown short-read genome, which should be reduced. (See detailed comments, below.) The manuscript would greatly benefit from professional editing or detailed proof-reading by a native English speaker.<br>Response: Thank you for your insightful comments for this manuscript. We are glad that the reviewer noticed the high quality of the chromosome-level assemblies for the two pangolin species and the potential value of our data as a resource for future conservation efforts.<br>We understand your concern about the need for a clearer narrative that highlights the biological insights and the most significant aspects of our data. In response to your suggestion, we have further revised the discussion section to better articulate the key research questions and the significance of our results. We removed the first part of the discussion and the description of the genome assembly in the result part to the supplementary data to help emphasize the most important findings.<br>Additionally, we have invited a native speaker to polish the revised manuscript and we believe that the language has been much improved.<br>MAJOR COMMENTS<br>Q12. For a Research Article, the main narrative is very unclear and the reader has to do far too much work to extract the relevant insights. The abstract hints at a "greater(?) improvement" and "different results in detecting genome-wide extinction risks being compared with short read assembled genome", though also results "were verified again" so are not actually different to previous findings? Except maybe one population (MJ2). This is all vague and confusing. Please clearly identify the key take-home messages from the new data, be explicit about it, and present a unified narrative from Abstract to Conclusions. Is the "relative mild genetic purging in pangolin populations" important? Is the purging itself the new finding, or the fact that it is relatively mild? I found the entire section (L268-294) to be rather confusing with a lack of clarity regarding population-specific versus general conclusions. The narrative would be aided greatly if the subheadings highlighted the key results/points rather than simply breaking the manuscript up into topics.<br>Response: Thank you for pointing out this issue, which we think could help improve this manuscript a lot. We try to answer your question and meanwhile re-organize the manuscript to make it more logic and clearer.<br>Yes, we obtained different results on the detection of genetic diversity, inbreeding, and accumulation of derived mutational load, based on the long-read assembled genome we showed in this study, when compared with studies reported by Hu et al, Wang et al and Wei et al. However, we did not directly compare with their studies to avoid over overemphasis this difference to showed superiority in this study? We just state our results, however, we almost didn’t show duplicated results with previous study. In the revised manuscript, we added some direct comparison to make it clearer, which could be found at many places throughout the manuscript.<br>Actually, this manuscript mainly focused on how a high-quality genome could improve the study of conservation genomics, and this manuscript is the first time (as far as I know) to compare the short-read assembled genome and long-read assembled

	genome on the evaluation of genetic parameters of conservation genomics or population genomics, although the comparison may be relative limited. In this manuscript, we prepared whole paragraph to discuss this comparison (Lines 417-440). Based on this comparison, we showed the superiority of the long-reads assembled genome to evaluate genetic diversity and inbreeding, further discussed genetic diversity and inbreeding we calculated based on the high-quality genome in this study, which could help us re-understand the genome-wide extinction risk of the two pangolin species. We also updated the result of ROH screening and accumulation of mutational load by using more appropriate parameters, please refer to Q7.<br>Furthermore, as you mentioned, we also explored the genetic purging in the five pangolin populations. The genetic purging always exists in a population under purifying selection[14], and is a very important issue in the conservation genomics. In general, deleterious alleles affecting fitness and viability are initially rare and may be hidden in the heterozygous state, but are more easily to be cleared from the population when these deleterious alleles are exposed in a homozygous state (Purging). This is an indicator for the evaluation of the extinction risk of endangered species. However, the genetic purging has been never discussed in (Hu et al, Wang et al and Wei et al). In this study we detected and discussed genetic purging in both Chinese and Malayan pangolin populations. Therefore, both the genetic purging and the "mild" are new findings in this study. We also revised the subheading to highlighted the key results/points in this study. Thank you again.<br>Q13. There are far too many supplementary figures and tables. I recommend trying to consolidate some (eg. S23-S28) into fewer multipanel figures that address the same question. Likewise, many of the tables could be combined and consolidated as the same data is presented for MJ and MP in two table, or tables with the same rows (samples) could be combined into a bigger table. Ideally, some of the descriptive results could be moved from the main manuscript body into a Supplementary Results section along with many of the references to these supplementary data. It would be helpful if the figures could be more carefully selected and organised/labelled to support the narrative.<br>Response: We appreciate your suggestions regarding the supplementary figures and tables. We have addressed these issues as follows: 1) We combined several groups of figures into multipanel figures, including you mentioned S23-S28. 2) We have reviewed the tables and combined those tables presenting similar data for MJ and MP into single tables where appropriate. Additionally, we merged tables with overlapping rows (samples) to create bigger tables. 3) We moved some descriptive results from the main manuscript to a Supplementary Results section to enhance the readability of the main text. We also adjust references to supplementary data accordingly. 4) We carefully selected and organized the figures, ensuring that they are well-labeled and directly support the narrative of the manuscript. We believe that the narrative of the revised manuscript has been much improved.<br>Q14. There is insufficient information and discussion about the previous pangolin genomes. Most important, genome "SG" does not seem to be defined anywhere. Is this a previously published genome? Or a short-read assembly of new data? There is a bit of discussion about the previous genomes (L306-323) but the comparisons in Tables S31 and S32 are unclear - are the new genomes missing from these tables? (What are they called?) I think this should be the first Results section. As these are phased chromosome-level vertebrate genomes, assembly statistics should be presented in the context of VGP/EBP standards. There should also be data presented for both haplotypes in Table 1.<br>Response: Thank you for your concern. "SG" stands for "short-read assembled genome", referring to a genome that was assembled based on the short-read sequencing technology. In this study, the SG genomes specifically referred to the genome of the Malayan pangolin of the YNU_ManPten_2.0 and of the Chinese pangolin of the YNU_ManJav_2.0 (collected from the NCBI). These two genomes have been widely used in previous studies, and we want to use this genome as a representative for the short-read assembled genome with low contiguity for comparison with the long-read assembled genome we prepared in this study. We defined it in Figure legend and Abbreviations section, we have added further explanations in the revised manuscript. Lines 242 and 957<br>To facilitate the reading and understanding of the differences between these published pangolin genomes and genomes assembled in this study, we added required information of our newly assembled genomes in Supplementary Tables 19. We have named our assemblies following the Vertebrate Genome Project (VGP) conventions

Powered by Editorial Manager® and ProduXion Manager® from Aries Systems Corporation

	and ToLIDs. Specifically, the Malayan pangolin genome assembly was named as mManJav1.1, and the Chinese pangolin genome assembly was named as mManPen9.1, and we have registered in the ToLIDs. In the Results section, we have provided detailed description of characteristics of our new assemblies in Table S3 and S4, and included a detailed description of assembly statistics in accordance with VGP/EBP standards (Table 1).<br>Q15. The SG vs LG comparison is largely a distraction and floods the paper with boring SI. It is no surprise that LG is better for SVs and long ROH. This is a research paper about pangolins, not a methods paper about LR vs SR genomes. Please reduce this to a clearer question: have there been any previous conclusions based on the other genomes that have been contradicted or (in)validated by the new genomes? Please give your new assemblies names that distinguish them from both each other and the previous assemblies - I recommend getting ToLIDs for your samples and naming the assemblies according to VGP conventions.<br>Response: Thank you for pointing out this. As we mentioned in the Q12, how a high-quality genome could improve the study of conservation genomics is a very important part in this manuscript. I agree with you that it is no surprise that LG is better for SVs and long ROH. As you mentioned, we can predict that a LG genome would be better than SG genome for the detection of SVs and long ROH fragments. However, many researchers may don’t know under what situation a LG genome will present a better performance than a SG genome, and to what extent a SG genome worse than a LG genome. As you could see in many conservation genomics papers, the inbreeding, genetic diversity and the accumulation of mutational load are all very very important genetic factors that could help wildlife managers (maybe the government) to make conservation decisions. An accurate evaluation of these genetic factors is a big thing in this research field. But unfortunately, I did not see a comprehensive evaluation and comparison between the SG and LG on the impact of evaluating of parameters in conservation genetics. In addition, as you know, for many people who used genome as a tool to guild conservation practice are actually not experts on genomics or freshman in this field, they need a guild to do their works? As far as I know, this is the first time we comprehensively compared the impact of the SG and LG on the evaluation of conservation genomics related parameters. Although it is not surprising that LG is better for SVs and long ROH, but we provided many detailed things other than a simple conclusion that the LG is better than the SG, which we think is very useful for wildlife conservation researchers and workers? Yes, we still agree with your points and we have tried to shorten this comparison in this revised manuscript.<br>Actually, in this manuscript, we almost only showed the results that were different or contradict (at least improved) with that reported in Hu et al and Wang et al:<br>1) We obtained different results on the detection of genetic diversity, inbreeding, and accumulation of derived mutational load, based on the long-read assembled genome we showed in this study, when compared with studies reported by Hu et al and Wang et al. Based on this comparison, we showed the superiority of the long-reads assembled genome to evaluate genetic diversity and inbreeding, further discussed genetic diversity and inbreeding based on the high-quality genome assembled in this study. Another point is that, we showed the very detail map of ROH distribution across each chromosome in the genome, which is not showed in Hu et al, Wang et al and Wei et al, and this information is very helpful for guiding the future genetic rescue of pangolin populations. For example: the breeding of Chinese pangolin in China is very successful and there are more than 100 captive individuals by far in the breeding center, but if we consider the reintroduction program, which individual should be selected to the wild? We could then select more appropriate candidate by referring this ROH map? But not to refer a very rough or a general inbreeding coefficient.<br>However, we did not directly compare result in this manuscript with previous studies (Hu et al and Wang et al) to avoid over overemphasis this difference to showed superiority in this study?, because the science and technology are moving forward. Although we just state our results, we almost didn’t show duplicated results which has been reported in previous studies. But you are right, in the revised manuscript, we added some direct comparison to make it clearer.<br>2) Furthermore, we also explored the genetic purging in the five pangolin populations, which are never discussed in Hu et al, Wang et al and Wei et al. In general, deleterious alleles affecting fitness and viability are initially rare and may be hidden in the heterozygous state, but are more easily to be cleared from the population when these deleterious alleles are exposed in a homozygous state, we could say that this process is genetic purging. The genetic purging always exists in a population under purifying

selection[14], and is a very important issue in the conservation genomics.
3) In addition, we have updated the results reported in previous study as we mentioned in Q7 and Q12.
To ensure clear distinction and proper identification of our new assemblies in the context of existing genomic data, we assigned unique names to our assemblies by following your advice. We obtained ToLIDs (Taxonomic IDs of Life) for our samples (MJ: mManJav1.1; MP:mManPen9.1), which will facilitate their recognition and propagate across the scientific community. In line with the VGP (Vertebrate Genomes Project) conventions, we renamed our assemblies accordingly, and incorporated these new names into the revised manuscript and ensure they are used consistently throughout the text, tables, and figures.
Q16. Do the new genetic data support the previous population groupings? (Was this based on genetic data itself?) To my eye, the genetic data presented in Fig S15 and S16 do not entirely support the existing population definitions. This needs more discussion. Is only a subset of MJ1 truly different to MJ2? Is there a fourth subpopulation of MP including the Taiwan sample?
Response: Yes, the genetic data supported the previous population grouping and this is almost based on genetic data itself, because the sampling locations for these samples are not all very clear.
In this manuscript, we mainly focused on the comparison of the grouping results (PCA, phylogenetic tree and admixture) generated based on the SG and LG, and to explore the potential impact of the reference genome to the genetic structure analysis. We found that the two groups of results are almost the same, and were very consistent with that reported in Hu et al and Wang et al (Supplementary Fig. S4-S7). Therefore, we did not present very detailed discussion about the genetic structure in this study to avoid repeated discussion. However, we still added a short description about the Taiwan individual in Lines 224-225.
Q17. Is it possible to clarify the "variable" karyotype from ref 47? Is this biological or technical variation in results? Is both species or just Mpen? Do your results clarify the karyotype in this population - the variation is geographical, right?
Response: As description in reference 47, the Chinese pangolin has been documented to exhibit four different groups of karyotypes, with the chromosome numbers varying from 2n = 36 to 2n = 42 across published research[16-18]. Yes, we cannot confirm from the reference 47 whether the Chinese pangolin's karyotype is variable, because 1) the 2n = 36 karyotype found in India may actually belong to M. crassicaudata[19], 2) the 2n = 42 from Taiwan could be due to a counting error[18]. It appears that the variability of the Chinese pangolin's karyotype cannot be confirmed based on reference 47. Therefore, We have therefore modified this description.
It is difficult to definitively determine whether the variable karyotype of the Chinese pangolin is resulted from biological variation or technical variation. This could be attributed to the different methods used for counting chromosomes, the techniques for preparing and staining the chromosomes, or the criteria used for identifying and counting, and so forth. Some of the variation might also stem from misidentification of species. As mentioned, the 2n = 36 karyotype found in India might actually belong to M. crassicaudata rather than M. pentadactyla.
The discussion in the ref 47 is specific to M. pentadactyla, and does not mention the M. javanica or other species in the context of the variations in diploid numbers. Also, we do not have karyotype data or HiC data from individuals from different populations, so we cannot confirm whether the karyotype variation (if have) existed in different populations.
Can you also confirm that the apparent swapping of chromosome arms on Chr4 between the species is not just a scaffolding error. Are these karyotypes consistent in both haplotypes?
Response: Thank you for your reminder. We used contigs to verify whether the swapping of chromosome arms on Chr4 was a scaffolding error (Response figure 3). The verification is as follows:

Response figure 3. Contig alignment across the region confirms the accurate rearrangement of Chromosome 4 swapping.
As shown in the figure above, by aligning contigs to Chromosome 4, we can see that both the breakpoints in the Chinese pangolin genome and the Malayan pangolin genome could be covered by a complete contig, which confirmed the accuracy of the assembly at this region, verifying the presence of the swapping showed in the main figure.

Powered by Editorial Manager® and ProduXion Manager® from Aries Systems Corporation

	Yes, the karyotypes is consistent in both haplotypes. In Fig S1, we have showed the Hi-C heatmaps for each haplotype to demonstrate that both haplotypes possess the same karyotype numbers. We also compared the haploid and diploid genomes separately, and the collinearity of all chromosomes is consistent (Response figure 4), further confirming the karyotypic concordance between the haploid and diploid genomes.<br><br>Response figure 4. (a) Synteny analysis reveals genomic consistency between haploid (MJH1) and diploid (MJG) Malayan pangolin genomes. (b) Synteny analysis reveals genomic consistency between haploid (MJH2) and diploid (MJG) Malayan pangolin genomes. (c) Synteny analysis reveals genomic consistency between haploid (MPH1) and diploid (MPG) Chinese pangolin genomes. (d) Synteny analysis reveals genomic consistency between haploid (MPH2) and diploid (MPG) Chinese pangolin genomes.<br>It would be good to see the contact maps (Fig S1) and synteny plots (Fig 1D) of the haplotypes against each other and have the telomeres marked to verify the orientation of arms where possible.<br>Response: The synteny plots between haplotypes were presented in Fig S17 (c) and (d). Following your suggestion, we have made every effort to identify the telomere status of our assembled genomes; telomeres in some chromosomes could be assembled, which are marked in Response table 1. Due to the limitations of sequencing technology, this study does not represent a complete telomere-to-telomere (T2T) pangolin genomes, therefore, telomeres sequences in some chromosomes could not be identified.<br>Response table 1. The distribution of telomeres in the genomes. “no” means no telomere sequence is identified, “left” means telomere sequence in the left end of the chromosome is identified, “right” represents telomere sequence in the right end of the chromosome is identified, “both” means telomere sequence in both ends of the chromosome is identified.<br><br>Is Fig S1 both haplotypes? If so, please mark the actual chromosome boundaries and orient the pairs in the same direction. Is the X pattern a pair of chromosomes inverted relative to each other, or is it interactions between chromosome arms of one haplotype?<br>Response: Yes, Fig S1 displayed the Hi-C heatmap of chromatin fibers from both haploid genomes. The X pattern you mentioned represents the interactions between chromosome arms of the two haplotypes, rather than a pair of chromosomes inverted relative to each other. Such interactions are common as it reveals the organization and physical contacts of chromatin in three-dimensional space. The actual chromosome boundaries you mentioned have been marked in the figure (Fig S1). The X pattern in the Hi-C heatmap is visualized solely to represent the interaction between the two sets of chromatin fibers. To avoid ambiguity and better display the karyotypes of the two haplotypes, we adjusted the sequences of the two haplotype chromosomes to make them initiate in the same direction, which could be seen in the revised Fig S1.<br>The parallel MP Chr5 pattern makes me think the former, but I initially assumed it was one haplotype.<br>Response: For the Chr5, the Hi-C interaction map reflected the interactions between the two haplotypes. In our revised version, the orientation of all pairs of haploid chromosomes have been adjusted to a better illustration of the interaction between the two haplotypes (Fig S1).<br>Where are the X and Y on the dot plots (Fig S7/S8)? Have both been moved into H1? It would also be clearer if Fig S3 used chromosome labels not scaffold numbers. How were the X and Y identified?<br>Response: In the original versions of Fig S7 and S8, the sex chromosomes were not depicted, and it was not the case that the sex chromosomes were all moved to H1 assembly. In the revised revision, we have incorporated the visualization of synteny between the sex chromosomes, which can be seen in Fig S19 (c) and (d). In this study, the assembled regions related to the Y chromosome of the Chinese pangolin and the Malayan pangolin are approximately 6Mb in length, and a complete Y chromosome assembly was not achieved, largely due to the limitations of sequencing and assembly technologies, as well as the highly repetitive and complex structure of the Y chromosome. There are relatively few homologous regions between the X and Y chromosomes, and the collinearity regions that can be visualized are also relatively limited. Therefore, the sex chromosomes were not depicted in the original FigS7 and S8. In the revised version, we have included the collinearity between the sex

Powered by Editorial Manager® and ProduXion Manager® from Aries Systems Corporation

	chromosomes in FigS19 (c) and (d).<br>For clarity, we updated Fig S3 by using chromosome labels instead of scaffold numbers in the revised manuscript.<br>We confirmed the X chromosome and Y-linked regions by two methods: 1) The presence of the SRY gene was used to identify Y-linked regions; 2) Based on the truth that the sequencing depth and coverage of the sex chromosomes is half of that of the autosomes for a male pangolin individual, as shown in Figure S3. For clarity, we have included a description of the identification of sex chromosomes in the methods section.<br>I think Fig 1C tries to present some of these data too but it is really unclear, being both too small and lacking any scales/legends.<br>Response: Thank you for your suggestion. We have revised Figure 1C by adjusting the panel size and adding legends and scale to clearly present the genomic information of the two pangolin genomes. Additionally, we have moved this figure to the appendix as Fig S19(a).<br>Given the questions over karyotype and the risk of scaffolding errors, I would like to see a deeper analysis of the proposed sites of fusion/fission.<br>Response: Thank you for your comments. To minimize the likelihood of false positive fusions and fissions that may arise from errors during scaffolding, we have conducted validation for all fission and fusions by aligning contigs to the genome assembly. This additional step ensures that the proposed fusion and fission sites are supported by more evidence, thereby preventing potential inaccuracies in scaffolding. The verification results are detailed as below (Response figure 5):<br><br>Response figure 5. Validation of the fusion, fission and main breakpoints between Chinese pangolin and Malayan pangolin genomes by aligning contigs to the two reference genomes. (a) Verification of fusion and fission positions. The left panel represents the synteny relationship, the left panel shows the support of contigs against the breakpoints of the fission and fusion events, the gray bar represents the contig and the red dot represents the breakpoint. (b) Verification of the other four significant breakpoints. In the IGV screenshot, the red mark represents the relative position of the SV breakpoint in the chromosome, and the gray band represents the contig at this position.<br>B. MINOR COMMENTS<br>Q18. There are a lot of typos. Most of these just impact readability, but some make the message unclear.<br>Response: Thank you for pointing out this question. We have reviewed and corrected these typos. Additionally, we invited a native speaker to polish the language throughout the manuscript to ensure clarity and improve readability.<br>Q19. I found the use of MJ, MP and CP to be a bit confusing at first. (MP could be Malayan Pangolin vs CP, but it isnt.) Can the MP/CP for the Chinese Pangolin be made consistent? Mjav and Mpen might be clearer. Likewise, it would be good to be consistent with the ordering and colours used for the species and populations when presented in tables and figures.<br>Response: Thank you for this comment. Yes, we agree with your suggestion, the Mjav and Mpen are clearer, and we changed the population name in the revised manuscript as the Response Table 1. We have also reviewed our manuscript and ensure that the ordering and colors were consistent throughout all tables and figures.<br><br>Q20. Page 4: "the most promising strategies for genome assembling" - This is combining long-read and HiC data. Whether ONT or PacBio is better is subject to debate.<br>Response: Yes, we agree. It is debated that whether ONT or PacBio is the better technology for long read sequencing, and both of the two sequencing technologies are among the best strategies for genome assembly at present. Therefore, we used ‘one of’ in the manuscript. Thank you! Line 94.<br>Q21. L214: " Among the three Chinese pangolin populations, the inbreeding of the CPA and CPB populations were comparable (CPA: 0.18±0.036; CPB: 0.17±0.039), but much worse than the CPC population (FROH=0.06±0.005) (Fig. 3A and 3E, Supplementary Table S26)." Does this relate to population size and genetic diversity? The heterozygosity of the reference genomes looks low from the Merqury kmer plots. Are they representation of the population heterozygosity?<br>Response: Thank you for this question. The ROH fragments could well reflect the inbreeding level in a population. In general, small populations are more tend to occur the inbreeding, and an inbred population often presents low genetic diversity (π) and

Powered by Editorial Manager® and ProduXion Manager® from Aries Systems Corporation

small population size. If a population is large enough and under the normal mating, it is almost impossible to present severe inbreeding. However, we have to keep in mind that, the inbreeding will not directly cause the low genetic diversity, but could make the genome homolyzed, and the homolyzed genome will further expose the recessive deleterious mutations, and some of these exposed deleterious mutations will cause severe consequences, such as death before mature, reproductivity problem or malformation….. Therefore, the loss of effective individuals in a population will take the genetic diversity away, then we can often see a low genetic diversity in an inbreeding population. But this is not always true, it is a very complicated thing if we discuss it as a specific issue.

The low heterozygosity showed in the Merqury kmer plots of the reference genome could reflect the genetic diversity of a single individual (usually can represent this species), but couldn't represent the population level genetic diversity. The nucleotide diversity (π) is a genetic parameter that could well reflect the population level genetic diversity.

Q22. L218: "Noteworthy, the FROH of the Taiwan individual is much higher than all other individuals in the MP population (FROH=0.54) (Fig. 3C)". There is no MP population. There are three. Confusing. Why is this noteworthy?

Response: Thank you. The MP population we mentioned in this manuscript represents the whole Chinese pangolin populations, including the CPA (MpenA), CPB (MpenB) and CPC (MpenC) populations. We mentioned the "MP" population in several places in the manuscript. To avoid the misunderstanding and make the manuscript more readable, we changed the "MP population" to the "the three Chinese pangolin populations" throughout the manuscript. Here we say "Noteworthy" is not because of the high inbreeding level in this individual, but because almost all the ROH fragments in this individual restricted to less than 1Mb (benefiting from the detailed distribution map of ROH fragments we detected in this study), which is not a normal thing for endangered species, but is underestimated in the study of Hu et al (please refer Q7).

Q23. Fig 3. Please make the colours and ordering match. Fig 3C is not clear. The size mapping is superfluous as it is just the y-axis. Would be better to use symbols/fill to more clearly differentiate the populations/species. It is not clear from this plot why Taiwan is singled out.

Response: Thank you for this reminder. We have revised Fig 3C to make the colors and ordering match each other. We removed the size mapping, and we now used the different colors to represent different populations. Notably, the FROH for the Taiwan individual is significantly higher compared to all other individuals in the Chinese pangolin population, then we showed the Taiwan individual to help reader to trace this individual.

Q24. L390. "The Chinese pangolin and Malayan pangolin used for genome assembly were wild rescued individuals by the Guangdong Wildlife Rescue Center. " As these are proposed reference genomes, is it possible to proved any photos or metadata to validate the species IDs? What populations are they from?

Response: Thank you to point out this issue. We have discussed with people in Guangdong Wildlife Rescue Center for this issue. Unfortunately, the geographical origin of these two individuals used in this study is not very clear, as both individuals were confiscated from criminals, and these individuals have been passed around many times, making it difficult to track the detailed geographical location for these two individuals.

Q25. L410: "Then, the Hi-C sequencing reads are mapped to the primary genomes by the mem algorithm of Burrows-Wheeler Aligner (BWA, v0.7.17) [66, 67], while Hi-C data quality control was conducted by the Juicer[68] (v1.5). The 3d-DNA pipeline (v190716) was finally used to concatenate and review the scaffolds to chromosome-scale genomes[69]. " How were phased assemblies produced if only the primary genome was scaffolded? Why were both haplotypes not scaffolded independently? I am confused how you can comment on haplotype synteny if not. Was manual editing of the scaffolding performed as part of the assembly curation?

Response: Thank you for this comment. The Hi-C sequencing data was used for two times in the genome assembly: 1) Phasing: we could feed the hifiasm software with both the HiFi sequencing data and Hi-C sequencing data, and then the hifiasm software will output three genomes at the same time: the hybrid genome, and the two haploid genomes (for a diploid species). The hifiasm software could automatically phase the hybrid genome into two haploid genomes, even we have no sequencing data from the trio samples. 2) Concatenating: the Hi-C data will be again used for contaminated the scaffolds generated in the previous step to the chromosome-scale

Powered by Editorial Manager® and ProduXion Manager® from Aries Systems Corporation

	genome. We have revised the description for the assembly of the genome to make it clearer (Lines 531-534). Yes, we will manually adjust the genome according to the Hi-C interacting map.<br>Q26. L460. What does “SNPs that were missed” mean? No coverage? Filtered?<br>Response: Thank you. In the VCF (Variant Call Format) file, a genotype at a specific genome position of all individuals will be listed in a row in the VCF file (Response figure 6), and if no sequencing reads could well support the base calling in this specific genome position in an individual (due to low sequencing coverage, low quality sequencing data, or failed base calling in complex genome regions……), we identified this as a missing genotype at this specific genome position for this individual. If the missing rate exceed 20% at a specific genome position, this row will be deleted from the VCF file. For example, there are 100 individuals in a VCF file, and the genotype at a genome position should be successfully called in more than 80 individuals, or this site will be filtered from the VCF file.<br><br>Response figure 6. A normal VCF file.<br>Reviewer #3: This manuscript by Lan et al. presents high-quality, haplotype-resolved, and chromosome-scale reference genomes for Chinese and Malayan pangolins, providing crucial insights into genetic diversity, inbreeding, and extinction risks. The study highlights moderate inbreeding in most populations, identifies a highly inbred Malayan pangolin population needing targeted conservation, and detects mild genetic purging. These findings contribute valuable genomic resources and new perspectives for pangolin conservation efforts. In general, the manuscript has clear logic, detailed description and comprehensive data analysis. Therefore, I recommend a minor revision before acceptance. There are some minor comments and suggestions as following :<br>Q27. 1. The authors used abbreviation of the Latin names for the Malayan pangolin (MJ) and Chinese pangolin (MP) in the third paragraph. However, they changed the way of abbreviation for the Chinese pangolin populations (CPA, CPB and CPC) later on. It's not a problem, but a little bit confusing for the readers to catch up the logic of describing.<br>Response: Thank you for pointing out this question. Yes, we agree. We used the CPA, CPB and CPC to keep consistence with the published papers, but as you mentioned, this will make the manuscript hard to be follow. Therefore, we changed the abbreviation name of the Chinese pangolin and Malayan pangolin to make them more readable (Response Table 1).<br><br>Q28. Lines 74-75, "The MJ1 population was …… diverged from the MJ1 population"???<br>Response: Thank you for pointing out this issue. We have corrected the description error in Lines 84.<br>Q29. 3. In Figure 1A, the legend is inconsistent with what have been shown in the figure. I suppose the authors used wrong colors to indicate CPA and CPC populations. Moreover, chromosome numbers, Hic_scaffold numbers in Figure 1C, Figure 1D, Figure S3 and Figure S5 are not in consistency. Please double check chr 7, chr 8, chr 20 and X chromosome in these figures.<br>Response: Thank you for your kind reminder. We have corrected the legend and the colors represent the population in Figure 1. In the original manuscript, Figure 1C shows the synteny between the two pangolin species, which was achieved by aligning and comparing the hybrid genomes of the two pangolin species. Figure 1D presents an analysis of the structural variation (SV) across the genomes of each pangolin species, specifically focusing on that between the two haplotype chromosomes. So, the numbering in these two figures is not consistent. We have carefully revised these figures to ensure that the chromosome and Hic_scaffold numbers are consistent across all mentioned figures. The revised figures can be found in Supplementary Figure 19a, Figure 1B, Supplementary Figure 3, and Supplementary Figure 15.<br>Q30...
**Additional Information:**	
**Question**	**Response**
Are you submitting this manuscript to a special series or article collection?	No

Powered by Editorial Manager® and ProduXion Manager® from Aries Systems Corporation

**Experimental design and statistics**<br><br>Full details of the experimental design and statistical methods used should be given in the Methods section, as detailed in our Minimum Standards Reporting Checklist. Information essential to interpreting the data presented should be made available in the figure legends.<br><br>Have you included all the information requested in your manuscript?	Yes
**Resources**<br><br>A description of all resources used, including antibodies, cell lines, animals and software tools, with enough information to allow them to be uniquely identified, should be included in the Methods section. Authors are strongly encouraged to cite Research Resource Identifiers (RRIDs) for antibodies, model organisms and tools, where possible.<br><br>Have you included the information requested as detailed in our Minimum Standards Reporting Checklist?	Yes
**Availability of data and materials**<br><br>All datasets and code on which the conclusions of the paper rely must be either included in your submission or deposited in publicly available repositories (where available and ethically appropriate), referencing such data using a unique identifier in the references and in the "Availability of Data and Materials" section of your manuscript.<br><br>Have you have met the above requirement as detailed in our Minimum Standards Reporting Checklist?	Yes

Revised manuscript - clean copy Click here to access/download;Manuscript;MainText-clean.docx

Enhancing inbreeding estimation and global conservation insights through HiFi assemblies of the Chinese and Malayan pangolin

Tianming Lan[1,2,3,†], Yinping Tian[3,†], Minhui Shi[3,†], Boyang Liu[3], Yu Lin[3],Yanling Xia[3], Yue Ma[3], Sahu Sunil Kumar[1], Qing Wang[3], Jun Li[2], Jin Chen[3], Fanghui Hou[4,5], Chuanling Yin[3], Kai Wang[2], Tengcheng Que[6,7], Wenjian Liu[6], Huan Liu[1], Haimeng Li[3,8,*] and Yan Hua[2,*]

[1] BGI Life Science Joint Research Center, Northeast Forestry University, Harbin 150040, China

[2] Guangdong Provincial Key Laboratory of Silviculture, Protection and Utilization, Guangdong Academy of Forestry, Guangzhou 510520, China

[3] College of Wildlife and Protected Area, Northeast Forestry University, Harbin 150040, China

[4] Guangdong Wildlife Rescue Monitoring Center, Guangzhou 510520, China

[5] Pangolin Conservation Research Center of National Forestry and Grassland Administration, Guangzhou 510520, China

[6] Faculty of Data Science City University of Macau, Macau 999078, China

[7] Guangxi Zhuang Autonomous Terrestrial Wildlife Rescue Research and Epidemic Diseases Monitoring Center, Nanning 530025, China

[8] Heilongjiang Key Laboratory of Complex Traits and Protein Machines in Organisms, Harbin 150040, China

*Correspondence address. Yan Hua, Guangdong Provincial Key Laboratory of Silviculture, Protection and Utilization, Guangdong Academy of Forestry, Guangzhou 510520 China. E-mail: wildlife530@hotmail.com; Haimeng Li, College of Wildlife and Protected Area, Northeast Forestry University, Harbin 150040, China. E-mail: lihaimeng66@163.com

†Equal contributions.

Abstract

A high-quality reference genome coupled with resequencing data is a promising strategy to address issues in conservation genomics. This has greatly enhanced the development of conservation plans for endangered species. Pangolins are fascinating animals with a variety of unique features,. Unfortunately, they are the most trafficked wild animal in the world. In this study, we assembled a chromosome-scale genome with HiFi long reads for the Chinese and Malayan pangolin and provided two new representative reference genomes for the pangolin species. We found a great improvement in the evaluation of genetic diversity and inbreeding based on these high-quality genomes and obtained different results for the detection of genome-wide extinction risks compared with genomes assembled using short reads. Moderate inbreeding and genetic diversity were reverified in these two pangolin species, except for one Malayan pangolin population with high inbreeding and low genetic diversity. Moreover, we identified a much higher inbreeding level (F_{ROH}=0.54) in the Chinese pangolin individual from Taiwan Province compared with that from mainland China, but more than 99.6% runs of homozygosity (ROH) fragments were restricted to less than 1Mb, indicating that the high F_{ROH} in Taiwan Chinese pangolins may have accumulated from historical inbreeding events. Furthermore, our study is the first to detect relatively mild genetic purging in pangolin populations. These two high-quality reference genomes will provide valuable genetic resources for future studies and contribute to the protection and conservation of pangolins.

Keywords: Chinese pangolin; Malayan pangolin; inbreeding; genetic purging; conservation genomics

Introduction

High-quality reference genomes enable the comprehensive analysis of population genomics and contribute to the revolution in conservation genomics[1]. However, fewer than 1% of the threatened species listed on the International Union for Conservation of Nature (IUCN) red list have a reference genome[2], and this number will further decrease if long-read assembly is considered. In the shift from conservation genetics to conservation genomics[3], high-quality reference genomes play an important role in providing the necessary support for the conservation of endangered species[1, 4]. In particular, genetic rescue is considered an important strategy to facilitate gene flow

and avoid inbreeding to increase the health of a population [5, 6]. A deep understanding of the genome-wide extinction risks and the basic genetic background of a small population is necessary for developing evidence-based strategies for genetic rescue. These include population structure, genomic diversity, genome-wide inbreeding, mutational load, and population demography[7-11]. A high-quality reference genome usually facilitates population-level studies in conservation genomics[1]. For example, the evaluation of inbreeding by measuring ROH relies on a high-quality reference genome with outstanding contiguity, because the long ROH fragments in small populations with high-level inbreeding often span several millions of base pairs [11-13], and can hardly be detected based on the fragmented genomes assembled by short reads.

The pangolin is a living fossil with many unique biological characteristics [14, 15], such as overlapping keratin scales covering the body, a specialized diet, a long and muscular tongue, a sensitive olfactory system, and burrowing ability [16, 17]. Locals across its distribution areas have traditionally used its scales and meat for medicine and food [18]. The overexploitation of pangolins driven by the soaring demand for luxury food and traditional Chinese medicine, has pushed this animal to the edge of extinction [19-22]. Currently, the pangolin is the most heavily trafficked wild animal worldwide, with more than 900,000 individuals poached over the last two decades and 67 countries from six continents involved in illegal poaching and trade [23]. For protection, all pangolin species have been placed into Appendix I of the Convention of International Trade of Endangered Species of Wild Fauna and Flora (CITES) as of 2016. Poaching is more rampant for Asian pangolins compare with African pangolins, particularly the Malayan pangolin (*Manis javanica*, hereafter MJ) and the Chinese pangolin (*Manis pentadactyla*, hereafter MP), which are under extreme survival pressure because of significant poaching and trafficking [8]. These two species have been listed as “Critically Endangered” on the IUCN Red List since 2014.

Previous studies identified two main Malayan populations[8] (hereafter MjavA and MjavB) and three main Chinese pangolin populations (hereafter MpenA, MpenB and MpenC). The MjavA population was identified from the mainland (China and Myanmar) and diverged from the MjavB population, which may have originated from the Southeast Asian islands. Among the three Chinese pangolin populations, the MpenA population is a newly discovered population distributed in Guangdong, China. The MpenB population is distributed over a vast area, including southern China and Thailand. The MpenC population highly diverged from the other two populations, which are likely to have originated from Myanmar (Fig. 1A, Supplementary Table S1 and S2). Although the varying population of the Chinese pangolin is still controversial[25], the highly divergent pangolin populations indicate that deep isolation may have occurred, which is usually detrimental to the survival of endangered species.

Pacific Biosciences (PacBio) high-fidelity (HiFi) sequencing technology combined with a HiFi-specific assembler and Hi-C or parental sequencing data, can generate high-quality , haplotype-resolved *de novo* assemblies, which represents one of the most promising strategies for genome assembly [26, 27]. This technique may facilitate a more accurate analysis of genome-wide genetic risks, specifically with respect to inbreeding. Because of the urgent need for establishing genomic backgrounds to support conservation, several reference genomes of pangolins have been assembled, annotated, and published[8, 16, 28-31]. However, high-quality reference genomes assembled from HiFi long-reads do not exist for pangolins. In this study, we present the genomes of the Malayan and Chinese pangolins at the chromosome-scale with haplotypes resolved down to the chromosomal-level, which provides new representative reference genomes for the pangolin species. We systematically examined the genomic backgrounds and evaluated the genome-wide extinction risks for five pangolin populations based on these two reference genomes.

Results

New representative reference genomes for the pangolin

To obtain high-quality reference genomes for both the Malayan and Chinese pangolin, we combined PacBio HiFi long reads, Hi-C reads and DNBSEQ short reads for genome assembly (Table. 1). We first generated phased contigs for both species and the haploid-resolved contigs were further linked at the chromosome-level by combining the HiFi long reads and Hi-C reads. We assembled 19 and 20 chromosome-scale pseudomolecules for MJ and MP, respectively (Fig. 1B, Supplementary Fig. S1), which was consistent with the karyotypic analysis[32], although the karyotype in pangolins may vary[32]. The diploid genome sizes assembled for MJ and MP were ~2.56 Gb and ~2.64 Gb, and represented 99.98% and 96.01% of the estimated genome size (~2.56 Gb for MJ and ~2.75 Gb for MP), respectively (Supplementary Fig S2). The contig and scaffold NG50 of the MJ/MP were 46.22Mb/56.16 Mb and 141.80 Mb/140.71 Mb, respectively (Table 1, Supplementary Table S3). In addition, we identified the X-chromosome and Y-linked regions of both genomes (Supplementary Fig. S3). The hifiasm assembler simultaneously yielded in two groups of haplotigs for each of the MJ (hereafter MJH1, MJH2) and MP (hereafter MPH1, MPH2) genomes (Supplementary Table S4). Multiple lines of evidence support the high completeness and low level of artificial duplication of the haplotype-resolved assemblies. Both the base-level quality evaluation and the structural-level assessment showed that all genomes (2 diploid assemblies and 4 haploid assemblies) had high assembly accuracy (see the Supplementary Material online). High collinearity between the MP and MJ genomes was also observed with 4 fissions and 3 fusions in the MJ genome compared with the MP genome (Fig. 1B), which was consistent with a previous karyotypic analysis [47] showing that these two genomes were accurately assembled at the chromosome level. Overall, we are confident in these two new, high-quality, and representative reference genomes for pangolins.

Table 1: Summary statistics for the genome sequences

Category	Metric	Chinese pangolin (MP)	Malayan pangolin (MJ)
**Sequencing Data**	WGS (Gb)	254.05	245.34
	HiFi (Gb)	65.81	95.99
	Hi-C (Gb)	239.77	227.48
	RNA-seq (Gb)	6.21	6.35
**Continuity**	Scaffold NG50[a] (Mb)	140.71	141.80
	Scaffold number	89	62
	Longest scaffold (Mb)	234.25	241.98
**Structural accuracy**	Reliable blocks[b]	97.16%	99.14%
	False duplications[c]	0.40%	0.48%
	Curation	manual	manual
**Base accuracy**	Base pair QV	57.06	55.08
	k-mer completeness	96.81	95.40
**Functional completeness**	BUSCO[d] assessment	98.00% complete	97.50% complete
	Transcript mappability	97.77%	97.26%
**Chromosome status**	Assigned[e]	97.22	98.72
	Pseudo-chromosomes Number	20	19

[a]Scaffold NG50: This metric represents the minimum length of a scaffold such that when all scaffolds are ranked by size, the cumulative length of scaffolds exceeding this threshold accounts for at least half of the estimated genome size. [b]Reliable blocks:These refer to genomic regions that are robustly supported by a minimum of 10 PacBio HiFi reads, ensuring a reliable assembly[33]. [c]False duplications:These are additional copies of k-mers present in a genome assembly beyond the expected count, as indicated by the k-mer histogram derived from the original high-fidelity reads[34]. [d]BUSCO: Benchmarking Universal Single-Copy Orthologs . [e]Assigned: This term denotes the percentage of the genome assembly that has been confidently assigned to specific chromosomes.

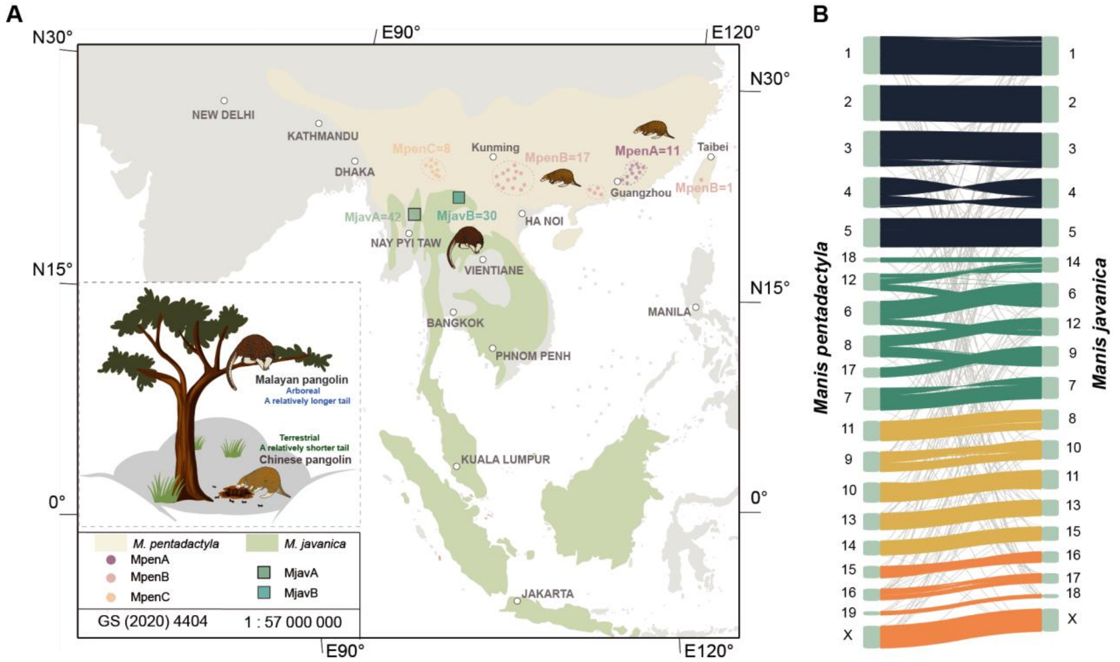

Figure 1: Introduction to the species distribution and chromosome synteny of the Chinese and Malayan pangolins. (A) The distribution area and sampling sites of the Chinese and Malayan pangolins in this study. The circles represent sampling sites of the Chinese pangolins reported by Wang *et al.*[24] Samples without detailed locations are not shown on the map. (B) The chromosome-scale synteny analysis between the Malayan pangolin and Chinese pangolin genomes.

Genome annotation

The total length of the repeat elements reached 1,260.92 Mb and 1,332.41 Mb, which accounted for 49.25% and 50.44% of the MJ and MP genomes, respectively (Supplementary Table S5). The composition of repeats in the MJ and MP genomes was similar, with the most abundant repeat element being LINE (MJ: 35.41%, MP: 37.36%), followed by LTR (MJ: 11.14%, MP: 13.61%), DNA element (MJ: 2.23%, MP: 2.11%) and SINE (MJ: 0.96%, MP: 0.91%). We predicted 19,680 and 19,886 gene models in the MJ and MP genomes, respectively (Supplementary Table S6). The gene regions spanned over 853.90 Mb and 791.49 Mb, which comprised 33.36% and 29.96% of the MJ and MP genomes, respectively (Supplementary Table S6). The average gene length, exon length, and intron length were 43.39 kb, 175.75 bp and 4.92 kb for the MJ genome and 39.80 kb, 175.07 bp and 4.64 kb for the MP genome (Supplementary Table S6). The BUSCO analysis revealed high completeness for the gene sets of both genomes, with the lowest BUSCO score greater than 95% (Supplementary Table S7). Overall, 19,575 (99.47%) and 19,792 (99.53%) genes were functionally annotated in the MJ and MP genomes, respectively (Supplementary Table S8). In addition, we predicted 288/435 rRNA, 1,296/1,348 miRNA, 806/350 tRNA, and 1,521/1,412 snRNA in the MJ/MP genomes (Supplementary Table S9).

The HiFi genome improves the evaluation of genetic diversity and inbreeding

We compared the short-read assembled genome (hereafter SG, the YNU_ManPten_2.0 and YNU_ManJav_2.0 were used here to represent the Chinese pangolin and Malayan pangolin genome, respectively) and the long-read assembled genome (PacBio HiFi assembled genome in this study, hereafter long-read assembled genome (LG)) to evalute for the evaluation of commonly used genetic parameters in population genomics, particularly in conservation genomics, including population structure, population history and separation, genetic diversity, and inbreeding (Supplementary Table S10).We found that the population structure (principal component analysis (PCA), phylogenetic tree, and admixture), population history, and population separation (inferred by MSMC2) were not significantly affected by these two types of reference genomes (Supplementary Fig. S4-S7), because the results

calculated based on the LG and SG were the same and consistent with previous reports. In contrast to other studies, however, we did not observe a distinct separation between the individual from Taiwan Province and other Chinese pangolins, although the Chinese pangolin in Taiwan Province is considered a subspecies of the Chinese pangolin[38] Therefore, either the genetic differentiation is still relatively small between the Chinese pangolin in Taiwan Province and mainland China, or the Taiwan individual was recently translocated from mainland China. For genetic diversity, the difference between π values calculated by LG and by SG was not large; however, this difference was significant, with a higher π value calculated based on the LG compare with that for the SG (Fig. 2A, Supplementary Table S10). Moreover, the ROH is an important genetic factor that reflects the inbreeding level in a population, whereas it was very sensitive to the quality of the reference genome. By screening ROHs across the genome to evaluate genome-wide inbreeding, we identified significant differences between the SG and LG (Table S11-S12). For the Malayan pangolin populations, the F_{ROH} calculated based on the LG was markedly higher compare with those calculated based on the SG (Fig. 2B, Supplementary Table S13). However, for the Chinese pangolin, this difference was significant only for ROHs larger than 1 Mb, although F_{ROH} was larger for the LG at other ROH lengths (Fig. 2C).

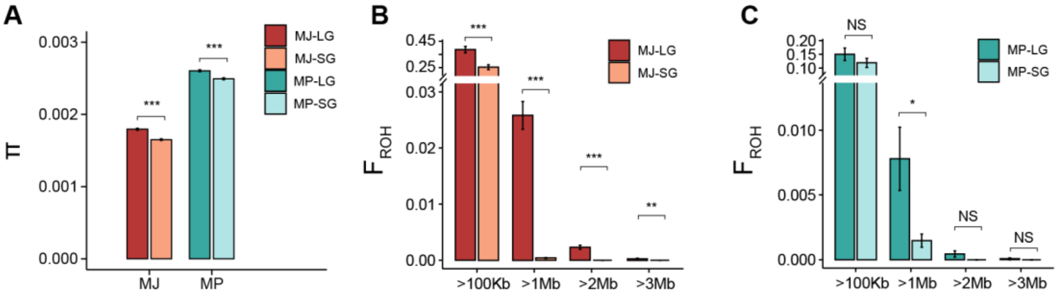

Figure 2: Comparison of the genome-wide genetic diversity and inbreeding estimated based on the LG (long-read HiFi assembled genome) and SG (short-read assembled genome). (A) Comparison of genome-wide π calculated based on the LG and SG in the Chinese and Malayan pangolin genomes. (B) Comparison of F_{ROH} calculated based on LG and SG in the Malayan pangolin genomes. (C) Comparison of F_{ROH} calculated based on LG and SG in the Chinese pangolin genomes. Note: NS: $p \geq 0.5$, *$p < 0.05$, **$p < 0.01$, ***$p < 0.001$.

Genome-wide genetic diversity and inbreeding

Considering the improvement in evaluating the genetic diversity and inbreeding based on the high-quality HiFi genomes, we performed a reassessment based on the LG and found that the genome-wide genetic diversity (π) of the Chinese pangolin and Malayan pangolin were 0.0026 and 0.0018, respectively, which were both higher compare than those calculated based on the SG (π_{MP}=0.0025, π_{MJ}=0.0016) (Supplementary Table S10). For the Chinese pangolin populations, MpenB exhibited the highest genetic diversity (π_{MpenB} = 0.0020), followed by the MpenC (π_{MpenC} = 0.0018) and MpenA (π_{MpenA} = 0.0017), which was consistent with previous study, but with higher π values[39]. Among the Malayan pangolin populations, the genetic diversity of MjavB (π_{MjavA} = 0.0024) was higher than that of the MjavA (π_{MjavB} = 0.0007). The mean *He* of the MjavA and MjavB were 0.063% and 0.189%, respectively, which were higher than the 0.043% and 0.141% values in a previous study[8]. The average genetic diversity of the Chinese pangolin was higher compared with that of the Malayan pangolin (Supplementary Table S10).

Inbreeding in small populations increases genome-wide homozygosity, and the resulting depression accelerates the loss of genetic diversity. For the MJ and MP populations, the average number of ROH fragments in each individual was 3,659.22±215.96 and 1,543.43±186.90, respectively. For both species, the ROHs were restricted to relatively small fragments (<1Mb), and the number of ROHs longer than 1Mb only accounted for 0.94% and 1.27% of the ROH fragments for the MP and MJ genomes, respectively[8]. The total length of ROHs larger than 1Mb also accounted for a small proportion of the two genomes (MP: 5.19%; MJ: 6.69%) (Fig. 3A and 3B). [40]We did not

observe any ROH fragments greater than 5Mb in either species. Similarly, the F_{ROH} in the MJ genomes (0.39±0.024) was higher than that in the MP genomes (0.15±0.023) (Supplementary Table S13). For ROHs longer than 1Mb, however, the F_{ROH} was sharply reduced to 0.026±0.003 and 0.0078±0.002 for the MJ and MP, respectively (Supplementary Table S13), which were much lower than that reported in a previous study[8, 40]. The very high minor allele frequency of 0.2 and other abnormally stringent filtering parameters used in the previous study as a threshold to filter the SNP may severely dilute the SNPs across the genome, resulting in an overestimation of the inbreeding in the genome.

We further compared the ROH distribution in different populations of these two species (Supplementary Fig. S8-S9). For Malayan pangolins, the inbreeding in the MjavA population (F_{ROH}=0.55±0.007) was more serious compare with that in the MjavB population (F_{ROH}=0.15±0.012) (Fig. 3C-3D, Supplementary Table S13). Although this difference became smaller for ROHs greater than 1Mb, it was still significant (Fig. 3D). Among the three Chinese pangolin populations, the inbreeding of the MpenA (F_{ROH}=0.18±0.036) and MpenB (F_{ROH}=0.17±0.039) populations were comparable, but much worse compare with that of the MpenC population (F_{ROH}=0.06±0.005) (Fig. 3C and 3E, Supplementary Table S13). Similarly, the differences in F_{ROH} between the three MP populations were reduced for ROHs longer than 1Mb (Fig 3E, Supplementary Table S13). As reported in a previous study[8], we found that the F_{ROH} of the Taiwan individual (F_{ROH}=0.54) was much higher than all other individuals in the MP population (Fig. 3C). Notably, we found that 99.6% of the ROH fragments in the genome of the Taiwan individual were less than 1Mb, which was much higher than that reported in the previous study (Supplementary Table S13). In addition, the F_{ROH} varied greatly among individuals in the MpenA or MpenB population, but this was not observed in the MpenC, MjavA and MjavB populations.

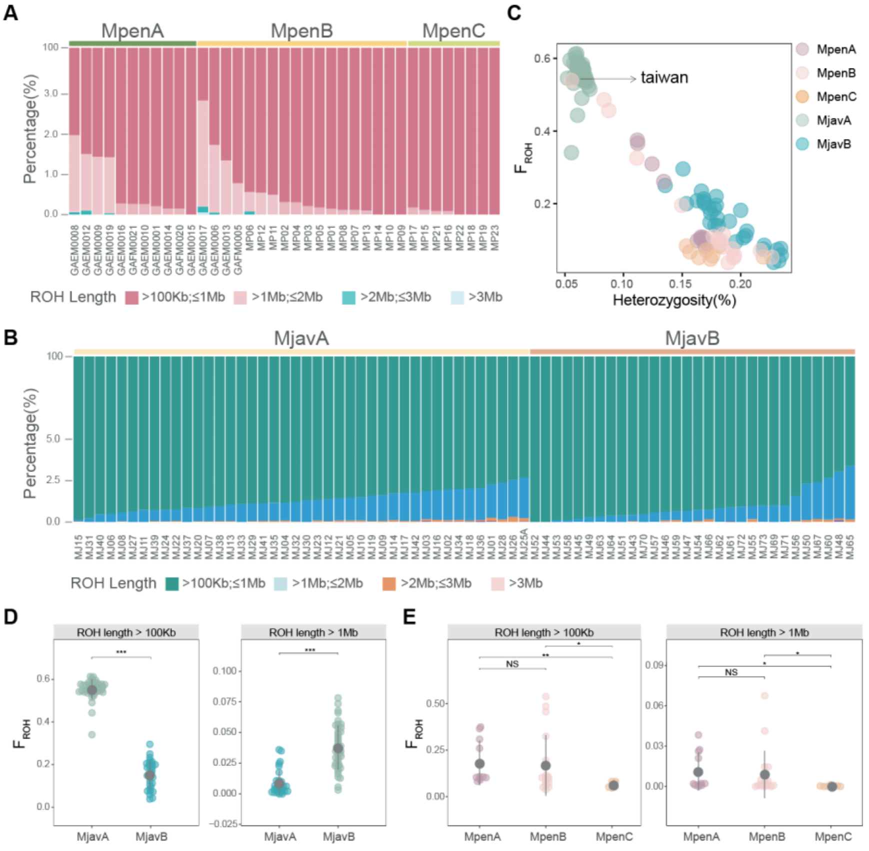

Figure 3: Genome-wide inbreeding estimated by ROH in the Chinese and Malayan pangolin populations. (A) The length distribution of ROH across the genome in the Chinese pangolin population. (B) The length distribution of ROH across the genome in the Malayan pangolin population. (C) Genome-wide heterozygosity and inbreeding estimates (F_{ROH}) for all five pangolin populations. (D) Comparison of the

averaged F_{ROH} in the MjavA and MjavB populations of the Malayan pangolins. (E) Comparison of the averaged F_{ROH} in the MpenA, MpenB and MpenC populations of Chinese pangolins.

Genome-wide mutational load

The mutational load is the burden of deleterious variants carried by a population, and reflects the evolutionary fitness of a population[41]. Although studies have examined the distribution of mutational load for the Chinese and Malayan pangolins[8, 24, 40], the HiFi genomes in this study provide new insight into the accumulation of mutational load in pangolins. We screened three categories of mutational load (loss of function, LoF; missense mutation; deleterious nonsynonymous mutation, dnsSNP) based on the HiFi genomes for the Chinese and Malayan pangolins (Fig.4A and 4B, Supplementary Table S14 and S15). We calculated the individual-level derived mutational load for each population to avoid bias introduced by different population sizes. In a previous study, the MpenC population was to harbor the most mutational load. In this study, however, we found that individuals in the MpenC population harbored the most mutational load, which was significantly more than that in the MpenB and MpenA populations (Fig. 4A). This may be the result of the HiFi reference genome used in this study and the different methods for the identifying the derived allele across the genome. The missense mutations and dnsSNPs in the MpenA population were comparable to those in the MpenB population (Supplementary Fig. S10a and S10b); however the MpenB population harbored many more LoFs compared with the MpenA population (Fig. 4A). Next, we focused on the derived homozygous mutational load (DHMD) and found that the MpenC population harbored the most DHMD, whereas MpenA and MpenB contained comparable DHMD (Fig. 4C, Supplementary Fig. S10c-S10f). For Malayan pangolins, the MjavB population harbored much more derived LoF compared with that of the MjavA population (Fig. 4B, Supplementary Table S14 and S15). However, the proportion of DHMD in the MjavA population for the LoF was comparable to that in the MjavB population (Fig. 4D).The MjavA population exhibited a higher proportion of DHMD for the dnsSNP and missense mutations than that of the MjavB population (Supplementary Fig. S10f), possibly because of the more efficient genetic purging of large-effect deleterious mutations (LoF), which was not investigated in previous studies. In the Genomic Evolutionary Rate Profiling (GERP) analysis, we obtained a highly similar result with the MpenC population harboring the most relative mutational load among the MP populations and the MjavA population harboring more relative mutational load than that in the MjavB population (Fig. 4E and 4F).

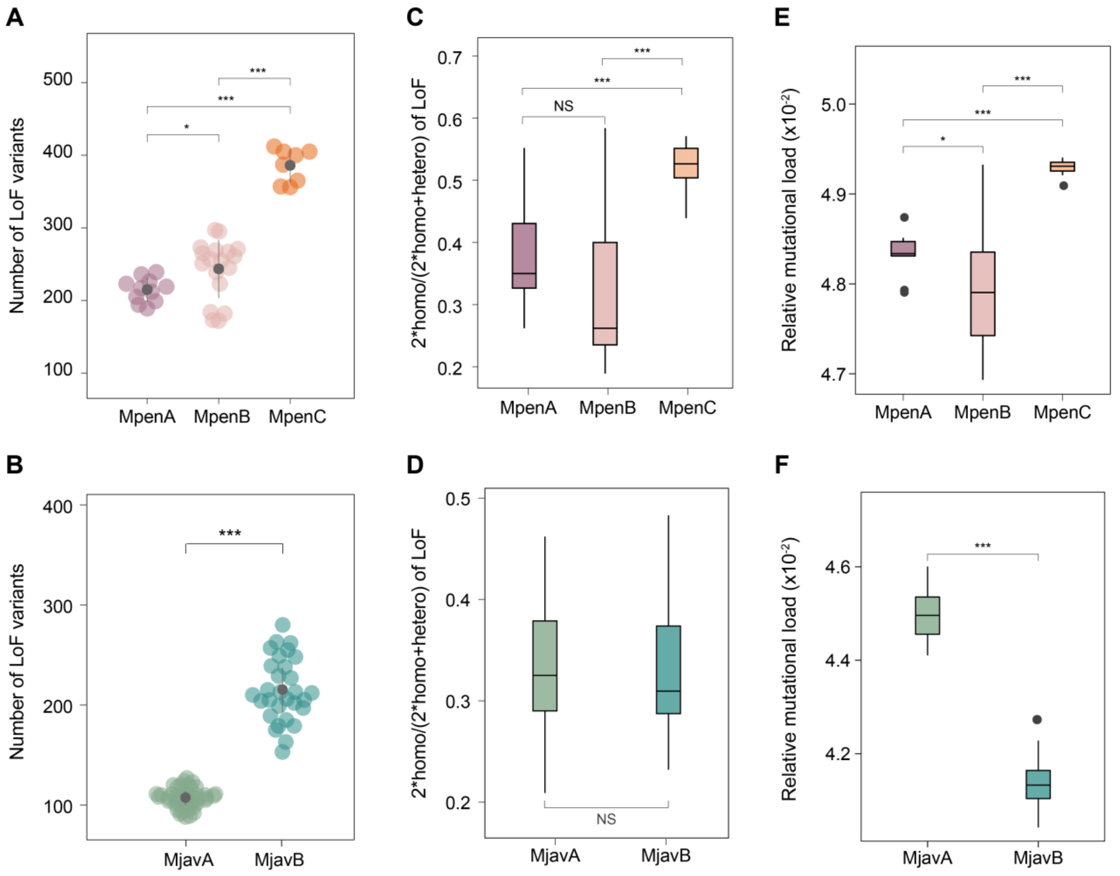

Figure 4: Mutational load in the Chinese and Malayan pangolin populations. (A) Total number of individual-level LoF mutations across the Chinese pangolin populations. (B) Total number of individual-level LoF mutations across the Malayan pangolin populations. (C) The ratio of homozygous LoF mutations in the Chinese pangolin populations was calculated by the formula: 2 × homozygous sites / (2 × homozygous sites + heterozygous site). (D) The ratio of homozygous LoF mutations in the Malayan pangolin populations was calculated using the same formula as that for the Chinese pangolin. (E) Relative mutational load in the Chinese pangolin populations (top 0.1% of GERP scores). (F) Relative mutational load in the Malayan pangolin populations (top 0.1% of GERP scores). The LoF here means loss-of-function mutations.

SFS (site frequency spectrum) analysis revealed that 7.91% and 9.07% of putatively damaging and neutral alleles, respectively, were fixed in the MpenC population. These two ratios were 6.84% and 9.53%, respectively, in the MjavA population; however, the proportion of the fixed alleles in the other three populations (MpenA, MpenB and MjavB) was much smaller (Fig. 5A and 5B). This indicates that the MpenC and MjavA populations may have experienced population bottleneck events, and genetic drift drove more rare alleles to fix into these two populations [7]. By comparing SFS lines between the Chinese and Malayan pangolins, we found that the SFS lines were flatter for polymorphic loci (fixed alleles excluded) in the Malayan pangolins, whereas the flattest SFS line was observed in the MjavA population, indicating the possibility of more serious bottlenecks in the evolutionary history of the MjavA population.

Genetic purging in pangolin populations

Genetic purging is an important process that has an impact on the accumulation of deleterious mutations in the population and it is usually more evident in small populations; however, few studies have discussed this issue in pangolins. To determine whether genetic purging has occurred in these pangolin populations facilitated by inbreeding, we first compared the occurrence of mutational load (LoF, missense mutation and dnsSNP) in the ROH regions (ROH*f*, the ratio of the number of mutational load to synonymous mutations in the ROH regions across the genome) and outside of the ROH regions (nonROH*f*, the ratio of the number of mutational load to synonymous mutations in

the nonROH regions across the genome)[10]. In the Malayan pangolin populations, the ROH*f* for highly deleterious mutations (LoF) was significantly lower than that of nonROH*f* in both the MjavA and MjavB population (Fig. 5C), but the difference (nonROH*f* / ROH*f*) was more pronounced in the MjavB population than that in the MjavA population (Fig. 5D). This denoted that many heterozygous deleterious mutations still existed in the genome[10, 11], which indicated that the genetic purging in the MjavA and MjavB populations was less efficient, but stronger in the MjavA population. This may be promoted by the higher-level inbreeding in the MjavA population. The dnsSNP and missense mutations showed the same situation (Supplementary Fig. S11a-S11c). The relatively lower proportion of derived homozygous LoF in the MjavA population also presented a decrease in highly deleterious mutations that may be caused by genetic purging (Fig. 4D, Supplementary Table S14). However, the Rxy analysis revealed an excess of mutational load in the MjavA populations than that in the MjavB populations (Supplementary Fig. S12a), with the lowest degree of excess found in the LoF. This suggests that genetic purging is limited, but tends to remove highly damaging mutations.

In the Chinese pangolin populations, the ROH*f* for LoF was lower than nonROH*f* in all three populations. The values of nonROH*f* and ROH*f* in the MpenA population were very similar, but the nonROH*f* was significantly higher compared with that in the ROH*f* in both the MpenB and MpenC populations (Fig. 5E and 5F), indicating that the purging of deleterious alleles in the MpenA population was more efficient than that in the MpenB and MpenC populations for the LoF mutations. However, this was not obvious for the relatively small-effect dnsSNP and missense mutations (Supplementary Fig. S11d-S11f). . The Rxy also indicated that the MpenA population harbored the fewest deleterious mutations, followed by the MpenB and the MpenC populations (Supplementary Fig. S12b). In addition, the number of fixed damaging alleles was not significantly less than neutral alleles in all five populations (Fig. 5A and 5B), further suggesting that the genetic purging in the pangolin populations is weak and not sufficient to clear a large number of deleterious mutations.

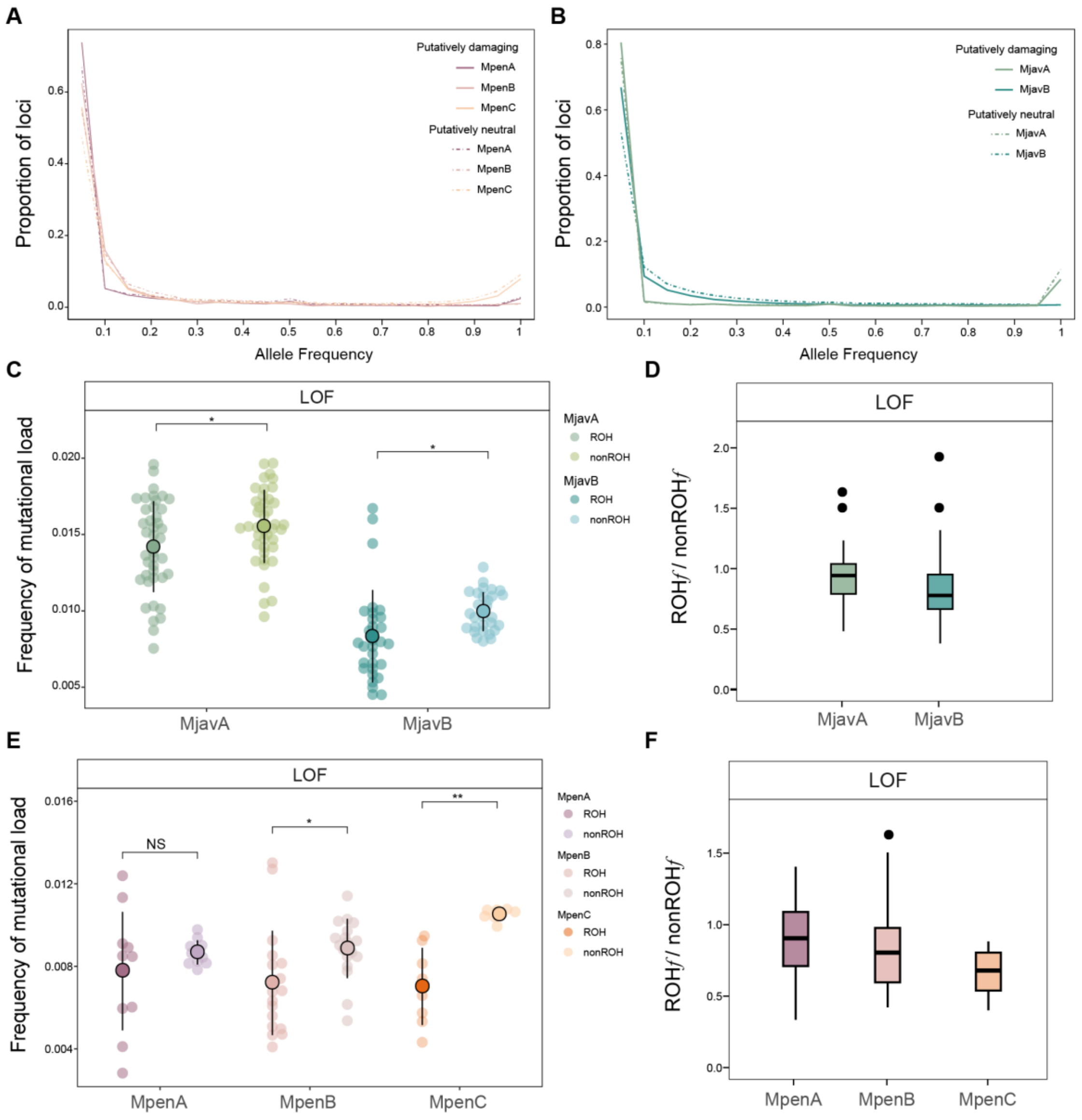

Figure 5: The SFS and genetic signals of genetic purging in pangolin populations. (A) SFS for putatively damaging (LoF and missense mutations) and neutral mutations (intergenic variants) in the MpenA, MpenB, and MpenC populations. (B) SFS for putatively damaging and neutral mutations in the MjavA and MjavB populations. (C) Dot plot showing the occurrence of LoF mutations in the two Malayan pangolin populations calculated as the ratio of the number of the mutational load to synonymous mutations in the ROH regions (ROH*f*) or nonROH (nonROH*f*) regions across the genome. (D) The ratio of ROH*f* to nonROH*f* for the LoF in the two Malayan pangolin populations. (E) Dot plot showing the occurrence of LoF mutations in the three Chinese pangolin populations calculated as that in (C). (F) The ratio of ROH*f* to nonROH*f* for the LoF in the three Chinese pangolin populations.

Discussion

HiFi genomes improve the evaluation of genetic diversity and inbreeding

Accurate and precise evaluation of genome-wide extinction risks by measuring a series of genetic parameters is the central issue in conservation genomics and primarily depends on the quality of the reference genome [1]. However, what genetic parameters can be improved the most with a better reference genome? Here, we showed that the two genetic parameters promoted by a higher-quality reference genome are genetic diversity (π) and inbreeding (ROH) (Fig. 3A-3C). We detected more variants across the genome based on the LG than the SG because long reads could 1) span much more complex genomic regions[1] and 2) generate much longer contigs than short reads[44, 45]. Many

genomic regions that cannot be assembled by short reads can be assembled by long reads, and these regions may contain important variants. In addition, longer contigs facilitate a higher number of reads that align accurately with the reference genome. Both of these may contribute to enhancing the accuracy of genetic diversity calculations. Therefore, we do not recommend comparing the genetic diversity calculated based on the SG to that calculated based on the LG; however, it may still make sense to compare the genetic diversity that was calculated based on the SG genomes between different populations.

The estimation of inbreeding by detecting ROH across the genome highly depends on genome contiguity, because short contigs in the SG can hardly span over long ROH fragments. As predicted, the inbreeding level detected using the LG were significantly higher than those identified using the SG for ROH fragments larger than 1Mb. When we focused on ROHs larger than 100kb, this difference in F_{ROH} was still significant in the Malayan pangolin population but not in the Chinese pangolin population (Fig 3B and 3C), which we inferred should have resulted from the different contiguities of the two pangolin genomes. Indeed, the contig N50 of the SG for the Chinese pangolin (133.77 kb) was much longer than that of the SG for the Malayan pangolin (73.8 kb), allowing for the detection of ROH longer than 100 kb. In contrast, the scaffold N50 of the Malayan pangolin genome was longer than that of the Chinese pangolin genome, suggesting that contiguity contributes more than scaffold contiguity to the detection of long ROH across the genome.

Genome-wide extinction risks in different pangolin populations

Although the Chinese and Malayan pangolin are listed as Critically Endangered species by the IUCN Red List, the genome-wide genetic diversity of these two species is moderate and even higher than other endangered flagship species[8, 46], such as the tiger [44], giant panda[47], golden snub-nosed monkey[48], and kākāpo[11]. In this study, we found an even higher genetic diversity than the previous reports[8, 46] for the two pangolin species (Supplementary Fig. S13, Supplementary Table S16). Although the pangolin populations have been declining for a long time, the recent population decline caused by poaching and illegal trade is more serious than ever, which has resulted in a very rapid decline in population size. Therefore, genetic drift and inbreeding may not have resulted in a substantial decrease in genetic diversity. Although the high-quality HiFi genome improved the estimation of inbreeding, the F_{ROH} in both species was still lower than many other endangered species[7, 44], indicating a relatively fine intrinsic genetic background for these pangolin populations.

We observed a much faster and sharper population decline for the MjavA population within the most recent 10,000 years compared with the other four pangolin populations (Supplementary Fig. S7), indicating that this Malayan pangolin population has a serious extinction risk. The LG-based population genomic analysis also revealed that both the genetic diversity (π_{MjavA}=0.0007) and inbreeding ($F_{ROH>100kb}$=0.55) in the MjavA Malayan population were much worse than those in other pangolin population as which may be caused by the isolation and limited gene flow with other populations, because the MjavA population is distributed across Southeast Asia [8] and the gene flow is easily separated by islands. However, we cannot precisely locate this population because of the lack of accurate sampling locations [8], which should be the subject of future conservation work. Another aspect to consider is that the Taiwan Chinese pangolin individual exhibited a very high level of inbreeding, with ROH fragments longer than 1Mb accounting for only ~0.4%, much less than the 8.04% reported in a previous study[8]. This may be attributed to the harsh filtration of SNPs in the previous study and the overestimation of ROH longer than 1Mb. Two possibilities may explain this phenomenon: 1) the Chinese population in Taiwan Province has ever been extensively inbred, but this situation has gradually improved, and the long ROH fragments have been broken by recombination over generations; and 2) this was a descendant of some highly inbred individuals translocated by humans from mainland China to the Taiwan Province. The repeated mating with the native Taiwan pangolin population broke the long ROH segments into small fragments. However, we cannot rule out that the Taiwan population may still face serious survival risks, and more Taiwan Chinese pangolin individuals need to be added to the analysis to draw a clear

conclusion.

The derived mutational load in all five pangolin populations was greater than Amur tiger and South China tiger populations, even with their lower inbreeding levels[44]. For the Chinese pangolin, the MpenC population had the highest proportion of mutational load. We hypothesized that the MpenC population represents an ancient and isolated Chinese pangolin population in Yunnan Province and is less disturbed by human activity, but has accumulated a large number of mutational load over its evolutionary history. We detected a stronger drift in the MpenC population, which can cause reduced efficacy of purifying selection to remove deleterious mutations[11]. In the Malayan pangolin, MjavA population showed low genetic diversity and high inbreeding. Although the high inbreeding in the MjavA population may promote the purging of deleterious mutations, we still observed a large amount of DHMD in this population. The significantly lower frequency of LoF inside compare with outside the ROH regionsindicates that many strongly recessive deleterious mutations remain in the nonROH regions in a heterozygous state and have not been effectively removed. This may be explained by the less efficient genetic purging in the pangolin populations.

Novel implications for the global conservation of the two pangolin species

High-volume poaching and trafficking have resulted in the overexploitation of pangolins; thus, the wild population, particularly the Chinese and Malayan pangolins, has plummeted to near extinction[8, 19-22]. Although moderate inbreeding and genetic diversity for these two species indicate a fine intrinsic genetic status, the population differentiation (F_{ST}) among the Chinese and Malayan pangolin populations remains large, which was also reported in other studies [8, 46, 49], even larger than the genetic differences of many subsoecies [50-52]. Genome-wide risks in different populations of Chinese and Malayan pangolins are also different[8, 46]. Therefore, in the effort of protecting and conserving pangolins, addressing, and managing the issues of illicit poaching and trafficking are just as important as genetic rescue efforts. Implementing timely protective and conservation measures for both the Chinese and Malayan pangolins will contribute to facilitating genetic rescue initiatives. Notably, both the genetic diversity and inbreeding of the MjavA population and the Taiwan individual are much worse than those in other pangolin populations, suggesting that the MjavA population, as well as the Taiwan Chinese pangolins may suffer more serious survival pressures than other pangolin populations, and should receive more attention and protection.

Materials and Methods

Samples, Resequencing Data and Ethics Statements

The Chinese and Malayan pangolins used for genome assembly were wild individuals rescued by the Guangdong Wildlife Rescue Center. During a routine examination, 5 mL of blood was collected with an anticoagulant tube, immediately transferred to liquid nitrogen, and stored at -80°C. The sample collection, experiment, and research design were all approved by the Institutional Review Board of BGI (BGI-IRB E22017). We strictly adhered to the guidelines provided by the BGI-IRB for all procedures. The whole-genome sequencing data of 37 Chinese pangolin and 72 Malayan pangolin individuals were downloaded from the National Center for Biotechnology Information (NCBI) and the China National GeneBank DataBase (CNGBdb) for population genomic analysis in this study[8, 16, 46].

Nucleic Acid Extraction, Library Preparation, and Sequencing

Total genomic DNA was extracted using the DNeasy Blood & Tissue Kit (Qiagen, USA) for whole genome sequencing (WGS) library preparation. Total RNA was extracted from blood using Trizol reagent (Invitrogen, USA) from blood, and 250-300 bp reverse transcribed cDNA fragments were used for DNA library construction. Two Hi-C libraries were prepared with *Dpn*II restriction endonuclease. DNA libraries were subjected to the Illumina HiSeq X Ten platform at Novogene (Tianjin, China) for paired-end sequencing. For high-molecular-weight genomic DNA,

the isolation was performed using the sodium dodecyl sulfate (SDS)-based method, and purification was carried out by the Qiagen Genomic Kit. A 15k library was constructed using high-quality DNA samples (main band > 30kb) and sequenced with the PacBio Sequel II platform at Novogene (Tianjin, China).

Genome Assembly and Assessment

To estimate the genome size, a total of ~100 Gb WGS short reads were used for analysis by the kmerfreq method[53] (v5.0). The hifiasm [26] (v0.16.1) software was used to generate the primary genome with PacBio HiFi and Hi-C sequencing data. Hifiasm utilized Hi-C sequencing data to achieve chromosome-level phasing in a method that does not require parental data[27]. This process can also phase the primary contigs at the same time into two sets of haplotigs, representing the two haploid genomes of a diploid genome. Genome redundancy was removed by the software Purge_dups[54] (v1.2.5). Then, the Hi-C sequencing reads were re-mapped to the primary genomes, after quality control by the Juicer[55] (v1.5)and the *mem* algorithm of Burrows-Wheeler Aligner (BWA, v0.7.17) [56, 57]. The 3d-DNA pipeline (v190716) was finally used to concatenate and review the primary scaffolds to chromosome-scale genomes[58]. We identified the X chromosome and Y-linked regions using SRY genes and WGS short readsand confirmed that the sequencing depth of the sex chromosomes was approximately half that of the autosomes.

The genome completeness was evaluated by BUSCO (v5.2.2) software using the vertebrata_odb10 data set [59]. We conducted a Merqury[60] (release 20200430) k-mer analysis and alignment of the whole genome sequencing reads to the reference genome to evaluate the accuracy of the genome assembly. Genome regions covered by PacBio long-read greater than 10-fold were considered accurately assembled regions[33]. The identification of syntenic blocks between pangolin genomes was primarily performed by the NUCmer program in MUMmer[61] (v4.0.0rc1), followed by filtration using the delta-filter program in MUMmer (v4.0.0rc1) with parameters "-i 90 -l 5000".

Genome Annotation.

Repeat elements in the genome were annotated using *de novo* and homology-based methods. *De novo* repeats were first annotated using the LTR finder[62] (v1.0.6) and RepeatModeler2[63] (v2.0.1), and the identified repeats were then merged into the RepBase library as known elements. Transposable elements were identified and classified using RepeatMasker (v4.0.5) with a conserved BLASTN search against the RepBase library [64]. The RepeatProteinMask program in RepeatMasker (v4.0.5) was used to identify repeat proteins [64]. The tandem repeats were annotated using Tandem Repeats Finder[65] (v4.07).

Protein-coding genes were annotated using *de novo*, homology-based, and transcript-based approaches after masking the repeat elements. For the *de novo* method, we used Augustus[66] (v3.0.3), GlimmerHMM[67] (v3.0.1), and SNAP[68] (v11/29/2013) to predict the gene models. For transcript-based prediction, the transcripts were mapped to the reference genome using HISAT2[69] (v2.1.0) and then assembled using StringTie[70] (v1.3.3b) based on clean RNA-seq data. Homology-based gene annotation was performed by using Blastall[71] (v2.2.26) with an E-value cut-off of 1e-5 towith against the protein sequences of *Homo sapiens*, *Mus musculus*, *Canis lupus familiaris*, and *Felis catus*. The final protein-coding gene set was generated using the MAKER[72] (v3.01.03) pipeline by combining high-quality homology-based, *de novo* and RNA-seq supported genes.

Functional annotation was performed by a BLAST (v2.13.0) search against the SwissProt, TrEMBL, and Kyoto Encyclopedia of Genes and Genomes (KEGG) databases with an E-value cut-off of 1e-5. InterProScan[73] (v5.52-86.0) was used to predict motifs, domains, and Gene Ontology (GO) terms. The tRNA genes were identified using tRNAscan-SE[74] (v1.3.1), whereas the snRNA and miRNA genes were detected by searching the reference sequences against the Rfam database (Release 12.0) using the BLAST (v2.13.0) and the program cmsearch from infernal (v1.1.1) software.

Detection of Structural Variants (SVs) in the Pangolin Genome

To identify sequence differences between the parental genomes, sequence alignment was performed using Mummer (v4.0.0rc1) with the parameters 'nucmer --maxmatch -c 500 -b 500 -l 100' [61]. SVs were detected based on the alignment results using SyRi[75] (v1.3). To verify the accuracy of the detected SVs, we aligned the PacBio long reads to the reference genome using BLAST (v2.13.0) to determine whether the reads crossed the breakpoints. Moreover, we extracted 300 bp of upstream/downstream flanking sequences for each breakpoint and manually verified them using DNBSEQ short reads by IGV[76] (v2.13.3) software. To identify gene loss in the haploid genome, we screened for pseudogenes interrupted by SVs using Mummer alignment and checked whether these genes had other copies across the entire genome.

Genome-wide Variant Calling and Quality Control

The BWA *mem* algorithm (v0.7.17) [56] was applied to map the whole-genome resequencing data of 72 Malayan pangolins and 37 Chinese pangolins to each of their reference genomes with default parameters. Sentieon[77] (v202010.01) was then used to sort, reorder, and deduplicate the alignment files for variant calling. Variants were detected for each individual using the Sentieon DNAseq Haplotyper pipeline, which is similar to the Genome Analysis Toolkit (GATK) HaplotypeCaller pipeline. Joint variant calling was performed using the Sentieon DNAseq GVCFtyper with all gVCF files to generate a VCF file. To prepare for downstream analysis, the variant set was filtered to remove InDels and multiallelic variants. For variant quality control, a stringent filtering step was performed using the following parameters: "QD <2.0 || FS > 60.0 || MQ < 40.0 || MQRankSum < -12.5 || ReadPosRankSum < -8.0". Additionally, we filtered SNPs that were missed in more than 20% of the individuals in a population. We used both the short-read assembled genome (SG) and long-read assembled genome (LG) as references to generate variant sets for downstream comparison.

Population Structure Analysis

Before we performed PCA, the VCF file was converted to PLINK format using PLINK software[78] (v1.90b6.10.Genome-wide complex trait analysis (GCTA)[79] (v1.92.2) software was used for PCA analysis using the default parameters. To construct a phylogenetic tree, vcf2phylip[80] (v2.7) was used to convert the VCF file into PHYLIP format. The best substitution model was then calculated using jModelTest[81] (v2.1.10), and the maximum likelihood phylogenetic tree was constructed using IQ-TREE[82] (v1.6.12) software with default parameters. ADMIXTURE[83] (v1.3.0) was used to determine the ancestry proportion with a specified number of clusters (K) ranging from 1 to 10. For this analysis, we used both the SG and LG as the reference genome to generate two groups of results.

Population Demography Inference

SMC++[84] (v1.5.1) was used to infer the historical changes in the effective population size of the varouus pangolin populations. The SMC++ results were visualized by scaling the time to real years using a generation time of one year and a mutation rate of $\mu = 1.47 \times 10^{-8}$[8, 16] for both the Malayan and Chinese pangolins. MSMC2 [85] (v2.1.1) was used to infer the changes in the effective population size over the evolutionary history with four randomly selected individuals from each pangolin population. SNPs were first phased by Beagle[86] (v5.1) and then subjected to MSMC2 for inference of the population history. We used the SG and LG as the reference genomes to generate two groups of results for comparison.

ROH and Genetic Diversity

To detect ROH fragments, multi-individual VCF files were converted into PLINK bfile format using the PLINK[78] (v1.90b6.10) software. The ROH was then detected using the PLINK[78] (v1.90b6.10) software with the parameters

"--homozyg --homozyg-window-snp 20 --homozyg-kb 10 --homozyg-density 50" [87]. ROHs shorter than 100 kb were excluded from the downstream analysis. F_{ROH} was calculated as $F_{ROH} = L_{ROH}/L_{AUTOSOME}$, where L_{ROH} represents the total length of ROHs in each genome and $L_{AUTOSOME}$ represents the total length of the autosomes. Genome-wide genetic diversity (π) was calculated using vcftools[88] (v0.1.16) using the parameters "vcftools --gzvcf vcf.gz --window-pi 500000 -out result". Genome-wide heterozygosity was calculated using vcftools[88] (v0.1.16) with "vcftools --gzvcf vcf.gz --het --out result" parameters. For ROH and genetic diversity analysis, we used both the SG and LG as reference genomes for comparison, but only the results calculated based on the LG were used for further discussion.

Mutational Load and Genetic Purging Analysis

To identify the mutational load in the protein-coding genes, the variants were first annotated using ANNOVAR[89] (v20191024) and SnpEff[90] (v.5.0e). Variants annotated as stop gained, splice acceptor variant, or splice donor by SnpEff[90] were predicted to be loss-of-function (LoF) mutations. Nonsynonymous variants with Grantham Score ⩾150 were considered deleterious mutations (dnsSNP) [91]. To determine the derived allele, the Malayan pangolin genome was split into 100 bp reads and mapped to the Chinese pangolin genome. If an allele was found within the Malayan pangolin genome and simultaneously represented the predominant allele (with an allele frequency exceeding 50%) within the Chinese pangolin population, it was designated as the ancestral state within the Chinese pangolin genome[92]. The same approach was used to determine the ancestral state of variants of the Malayan pangolin.

The occurrence of the mutational load in the ROH and non-ROH regions (${}_{ROH}f$ and ${}_{nonROH}f$) for each individual genome was calculated by dividing the total number of deleterious mutations (N_m) within the ROH or non-ROH region by the number of synonymous mutations in the same region (S_{ROH} and S_{nonROH}) as follows:

$$ {}_{ROH}f = \frac{N_m}{S_{ROH}} $$

$$ {}_{nonROH}f = \frac{N_m}{S_{nonROH}} $$

To estimate the relative excess of deleterious mutations in one pangolin population compare with another, we performed the Rxy analysis for dnsSNP, missense mutations, LoF, and synonymous mutations between pairs of pangolin populations [10]. We calculated the Rxy value using the following formula:

$$ L_X = \frac{\sum_{i\in C}(m_X^i/s_X^i)(1 - m_Y^i/s_Y^i)}{\sum_{i\in I}(m_X^i/s_X^i)(1 - m_Y^i/s_Y^i)} $$

$$ R_{X/Y} = L_X/L_Y $$

Where m_X^i represents the count of derived alleles for the above mentioned mutations observed at each site (*i*) within one population (*X*) and m_Y^i represents that in another population (*Y*). s_X^i and s_Y^i represent the total number of alleles at each site (*i*) of the population (*X* or *Y*). *C* represents the above-mentioned category of protein-coding sites, whereas *I* denotes the intergenic sites. We used the jackknife method during the calculation to obtain a standard error measurement. If Rxy = 1, both populations have the same level of derived mutation load, whereas if Rxy < 1, then population Y has more derived load than X and vice versa if Rxy > 1.

Genomic Evolutionary Rate Profiling (GERP) Scores

It is difficult to estimate the genetic load without fitness data. Therefore, we calculated the relative mutational load

for each individual genome. First, we screened the derived alleles distributed in the highly conserved genome region of the two pangolin species using the genomic evolutionary rate profiling scores (GERP) method. To calculate the GERP scores, we selected the genomes of 37 species (*Acinonyx jubatus*, *Bos taurus*, *Callithrix jacchus*, *Canis lupus*, *Cavia porcellus*, *Choloepus hoffmanni*, *Dasypus novemcinctus*, *Dipodomys ordii*, *Echinops telfairi*, *Equus caballus*, *Erinaceus europaeus*, *Felis catus*, *Homo sapiens*, *Loxodonta africana*, *Lynx canadensis*, *Mus musculus*, *Myotis lucifugus*, *Ochotona princeps*, *Oryctolagus cuniculus*, *Panthera pardus orientalis*, *Panthera tigris*, *Pan troglodytes*, *Prionailurus bengalensis*, *Procavia capensis*, *Pteropus vampyrus*, *Puma concolor*, *Rattus norvegicus*, *Sorex Araneus*, *Spermophilus tridecemlineatus*, *Tupaia belangeri*, *Tursiops truncates*, *Vicugna pacos*, *Manis javanica*, *Manis pentadactyla*, *Tamandua tetradactyla*, *Ovis aries*, and *Vulpes lagopus*) for screening ultra-conserved genomic regions. We split these genomes into 100 bp reads to generate fastq files. Then, we respectively aligned these fastq files to the Malayan and Chinese pangolin genomes using the *mem* algorithm in BWA (v0.7.17-r1188) with "-B 3" parameter. GERP scores were then calculated using the gerpcol program from the GERP++[93] software (http://mendel.stanford.edu/sidowlab/downloads/gerp/index.html) based on the above-mentioned alignment files. In general, low GERP scores (<1) usually represent putatively neutral genome regions, whereas high GERP scores (>1) indicate conserved genome regions [11]. Derived alleles in more conserved genome regions (those with higher GERP scores), these alleles are likely to be more deleterious. In this study, we calculated the relative mutational load with mutations having the top 0.1% GERP scores to select more deleterious alleles distributed in highly conserved genome regions [7]. The relative mutational load was calculated by the following formula: the sum of all homozygous and heterozygous derived alleles multiplied by their conservation score over the total number of derived alleles, with the heterozygous counted as one allele and homozygous sites counted as two alleles [11]. Therefore, a higher relative mutation load indicates that a relatively larger proportion of derived alleles may be found in more conserved genomic regions.

Site-Frequency Spectrum (SFS) Analysis

For SFS analysis, we calculated the frequency of each type of mutations at every site in the various pangolin populations. We considered intergenic variants as neutral, whereas LoF and missense variants were consider putatively damaging mutations[7]. For SFS in each pangolin population, we subsampled nonmissing derived alleles from each locus for calculation[7]. Fixed (frequency=1) and missing (frequency=0) alleles were included in the SFS for the five populations. We used LG as the reference genome for SFS analysis.

Additional Files

Supplementary Fig. S1. The heatmap represents the contact matrices generated by aligning the Hi-C data to the haploid chromosome-level Chinese pangolin (a) and Malayan pangolin (b) genomes.

Supplementary Fig. S2. Estimated genome size of the Chinese and Malayan pangolin genomes by using K-mer frequency analysis with k-mer size of 17. (a) The K-mer spectra of the Chinese pangolin genome. (b) The K-mer spectra of the Malayan pangolin genome.

Supplementary Fig. S3. Sequencing depths of each pseudo-chromosome. (a) Sequencing depths of the 19 autosomes, X chromosome, and Y chromosome in the Chinese pangolin genome. (b) Sequencing depths of the 18 autosomes, X chromosome, and Y chromosome in the Malayan pangolin genome.

Supplementary Fig. S4. Comparisons of LG and SG on PCA analysis for both Chinese and Malayan pangolin populations. (a) PCA analysis of Malayan pangolin populations based on SG. (b) PCA analysis of Malayan pangolin populations based on LG. (c) PCA analysis of Chinese pangolin populations based on SG. (d) PCA analysis of

Chinese pangolin populations based on LG.

Supplementary Fig. S5. Comparisons of LG and SG on the construction of phylogenetic tree for both Chinese and Malayan pangolin populations. (a) Phylogenetic tree constructed based on the SG for Malayan pangolin populations. (b) Phylogenetic tree constructed based on the LG for Malayan pangolin populations. (c) Phylogenetic tree constructed based on the SG for Chinese pangolin populations. (d) Phylogenetic tree constructed based on the LG for Chinese pangolin populations.

Supplementary Fig. S6. Comparisons of LG and SG on the admixture analysis for both Chinese and Malayan pangolin populations. (a) Genome-wide admixture analysis for three populations of Chinese pangolin based on the SG. (b) Genome-wide admixture analysis for three populations of Chinese pangolin based on the LG. (c) Genome-wide admixture analysis for two populations of Malayan pangolin based on the SG. (d) Genome-wide admixture analysis for two populations of Malayan pangolin based on the LG.

Supplementary Fig. S7. Comparison of LG and SG assembly for analyzing population history and separation in Chinese and Malayan pangolin populations. (a) The dynamics of effective population size of Malayan pangolin populations analyzed based on the SG. (b) The population size dynamics of Malayan pangolin populations analyzed based on the LG. (c) The population size dynamics of Chinese pangolin populations analyzed based on the SG. (d) The population size dynamics of Chinese pangolin populations analyzed based on the LG. (e) The divergence time between two populations of Malayan pangolin estimated based on the SG. (f) The divergence time between two populations of Malayan pangolin estimated based on the LG. (g) The divergence time among three populations of Chinese pangolin estimated based on the SG. (h) The divergence time among three populations of Chinese pangolin estimated based on the LG.

Supplementary Fig. S8. (a) The population-level ROH distribution in three Chinese pangolin populations. (b) The population-level ROH distribution in two Malayan pangolin populations.

Supplementary Fig. S9. (a)The individual-level distribution of ROH larger than 100Kb in Chinese pangolin genomes. (b) The individual-level distribution of ROH larger than 100Kb in Malayan pangolin genomes. Each row represents an individual.

Supplementary Fig. S10. Total deleterious nonsynonymous SNP (dnsSNP) (a) and missense (b) mutations at the individual level were assessed across five populations of Chinese and Malayan pangolins. The number of individual-level homozygous LoF(c), dnsSNP (d) and missense (e) mutations across the five pangolin populations. (f) The ratio of homozygous missense mutations and dnsSNPs in Chinese and Malayan pangolin populations was calculated as the formula of : $2 \times$ homozygous sites / ($2 \times$ homozygous sites + heterozygous site).

Supplementary Fig. S11. Dot plot showed the occurrence of dnsSNPs (a) and missense mutations (b) in the two Malayan pangolin populations calculated as the ratio of the number of mutational load to synonymous mutations in the ROH regions (ROH*f*) or nonROH regions (nonROH*f*) across the genome. The ratio of ROH*f* to nonROH*f* for dnsSNP and missense mutations in Malayan pangolin populations (c).Dot plot showed the occurrence of dnsSNPs (d) and missense mutations (e) in the three Chinese pangolin populations calculated as the ratio of the number of mutational load to synonymous mutations in the ROH regions or nonROH regions across the genome.The ratio of ROH*f* to nonROH*f* for dnsSNP and missense mutations inChinese pangolin populations (f)

Supplementary Fig. S12. (a) The Rxy ratio of derive alleles in MjavA (x) to MjavB (y) for dnsSNP, synonymous, missense and LoF. The Rxy <1 indicated the population y has more derived alleles than population x. (b) The Rxy ratio of derive alleles in x population to y population (x/y: MpenB/MpenC; MpenA/MpenC; MpenA/MpenB) for dnsSNP, synonymous, missense and LoF. The Rxy <1 indicated the population y has more derived alleles than population x.

Supplementary Fig. S13. Comparison of genome-wide π of the Malayan pangolin and Chinese pangolin with other endangered species. Abbreviations along the X-axis are as follows: CMA: Brown eared pheasant (*Crossoptilon*

mantchuricum), ASI: Chinese alligator (*Alligator sinensis*), PTA: Amur tiger (*Panthera tigris altaica*), AFU: Red panda (*Ailurus fulgens*), AME: Giant panda (*Ailuropoda melanoleuca*), MJ: Malayan pangolin (*Manis javanica*), MBE: Dwarf musk deer (*Moschus berezovskii*), MP: Chinese pangolin (*Manis pentadactyla*).

Supplementary Fig. S14. K-mer spectra plot estimated by Merqury. (a) K-mer spectra plot for the haplotype-resolved chromosome-level genome of MP. (b) K-mer spectra plot for the haplotype-resolved chromosome-level genome of MJ.

Supplementary Fig. S15. The sequencing depths of the two groups of haplotigs in both MJ and MP genomes. (a, b) Alignment with DNBSEQ read. (c, d) Alignment with PacBio HiFi reads.

Supplementary Fig. S16. Pairwise differences observed between the haploid genomes of Malayan and Chinese pangolin. The sliding window was set to be 100 bp.

Supplementary Fig. S17. (a) Brief introduction and circos diagram of the two pangolin genomes. (b) Structural rearrangements between the two haplotypes of each chromosome in Malayan pangolin and Chinese pangolin genomes. (c) Dot plot between MJH1 (x-axis) and MJH2 (y-axis), plotted by pafCoordsDotPlotly. (d) Dot plot between MPH2 (x-axis) and MPH1 (y-axis), plotted by pafCoordsDotPlotly.

Supplementary Fig. S18. Validation of structural variants through contig mapping. Contigs are mapped to both the haploid genomes to verify structural variants and visualized by the IGV software. (a) The correct structural variants between the haploid genomes. The breakpoints of the SVs could be covered by complete contigs in both of the haploid genomes. (b) The incorrect structural variants between the haploid genomes. The breakpoints of the SVs in one of the two haploid genomes could not be covered by the complete contig. In the IGV screenshot, the gray bar represents the contig spanning over the SVs, and the contig corresponding SV regions were marked as the red box. All structural variants have been verified, and several are randomly displayed here.

Supplementary Fig. S19. (a) The GO enrichment result of genes distributed in structural variants of Malayan pangolin. (b) The KEGG enrichment result of genes distributed in structural variants of Malayan pangolin. (c) The GO enrichment result of genes distributed in structural variants of Chinese pangolin. (d) The KEGG enrichment result of genes distributed in structural variants of Chinese pangolin.

Supplementary Table S1. Summarized sample information in this study.

Supplementary Table S2. Information of five populations in Chinese and Malayan pangolin included in this study.

Supplementary Table S3. Statistics of genome assemblies for the Chinese pangolin and Malayan pangolin.

Supplementary Table S4. The length of each chromosome in the Chinese pangolin and Malayan pangolin genomes.

Supplementary Table S5. The overall statistics of repeats in the Chinese and Malayan pangolin genomes.

Supplementary Table S6. Statistics of the annotated genes in Chinese and Malayan pangolin genomes.

Supplementary Table S7. BUSCO analysis of genome assemblies and gene sets in this study.

Supplementary Table S8. Statistics of functional annotation for the Malayan and Chinese pangolin's gene sets.

Supplementary Table S9. Statistics of ncRNA annotation.

Supplementary Table S10. SNPs number, genetic diversity (π), heterozygosity and SNP density calculated based on LG and SG in Malayan and Chinese pangolin populations.

Supplementary Table S11. The comparison of the SG and LG for estimating ROH in Malayan pangolin and Chinese pangolin populations

Supplementary Table S12. The count and length of ROH fragments in the five populations of Malayan pangolin and Chinese pangolin.

Supplementary Table S13. The F_{ROH} in the five populations of Malayan pangolin and Chinese pangolin.

Supplementary Table S14. The total count of dnsSNPs, LoF, missense and synonymous SNPs in various populations, along with the average number of dnsSNPs, LoF, missense, and synonymous SNPs at the individual level, across different populations of the Malayan and Chinese pangolins.

Supplementary Table S15. The individual level mutational load estimates in all Chinese pangolin and Malayan pangolin populations.

Supplementary Table S16. Comparison of genome-wide nucleotide diversity (π) of the Malayan pangolin and Chinese pangolin with reference to other endangered species on the IUCN Red List.

Supplementary Table S17. Quality assessment of the MP and MJ genomes by the Merqury software.

Supplementary Table S18. The mapping rates of four types of sequencing data to genomes assembled in this study.

Supplementary Table S19. Comparison of the assembly statistics with the previously published Chinese pangolin and Malayan pangolin genomes.

Supplementary Table S20. Pairwise differences observed in comparisons between the haplotype genomes of Chinese and Malayan pangolin, the sliding window was set to be 100bp.

Supplementary Table S21. Chromosomal structural variants (>50bp) of MJH1 and MJH2, and of MPH1 and MPH2.

Supplementary Table S22. Genes distributed in the structural variants of MJ and MP genomes.

Supplementary Table S22. The KEGG enrichment result of genes distributed in the structural variants of MJ/MP.

Supplementary Table S23. The GO enrichment result of genes distributed in the structural variants of MJ/MP.

Supplementary Table S24. Functional description of pseudogenes interrupted by structural variants.

Data Availability

Bioproject and biosample for the genomic data of this study were submitted to NCBI under accession numbers PRJNA1114675. The accession number of genomes are GCA_040802235.1 and GCA_040802205.1. The data that support the findings in this study also have been deposited into CNGB Sequence Archive (CNSA) [94]of China National GeneBank DataBase (CNGBdb) [95] with accession number CNP0004630. The resequencing data in this study were retrieved from earlier studies (37 Chinese pangolins: CNP0001723, CNGBdb; PRJNA529540 and PRJNA20331, NCBI Read Archive. 72 Malayan pangolins: PRJNA529540, NCBI Read Archive) [8, 16, 46].

Abbreviations

WGS: Whole genome sequence; RNA-seq: RNA sequence; BUSCO: Benchmarking Universal Single-Copy Orthologs; HiFi: High fidelity; Hi-C: High-throughput/resolution chromosome conformation capture; PCA: Principal component analysis; SNP: Single-nucleotide polymorphism; TE: Transposable element; KEGG: Kyoto Encyclopedia of Genes and Genomes; GO: Gene Ontology; GATK: Genome Analysis Toolkit; SG: Short-read assembled genome; LG: Long-read assembled genome; ROH: Runs of homozygosity; LoF: Loss-of-function;

dnsSNP: Deleterious nonsynonymous mutation; GERP: Genomic evolutionary rate profiling; SFS: Site-Frequency Spectrum.

Author Contributions

Tianming Lan, Huan Liu, Yinping Tian and Yan Hua conceived and initiated the project. Yan Hua, Jun Li, Fanghui Hou, Yue Ma,Tengcheng Que, Wenjian Liu and Kai Wang collected the samples. Jin Chen, Chuanling Yin and Yinping Tian performed DNA isolation, library preparation and genome sequencing. Haimeng Li, Minhui Shi, Boyang Liu and Qing Wang assembled the genomes and conducted the genomics analysis. Tianming Lan coordinated the genomic analysis. Tianming Lan and Haimeng Li wrote the manuscript. Sunil Kumar Sahu, Minhui Shi, Yanling Xia and Boyang Liu reviewed and edited the manuscript. Tianming Lan and Yan Hua made important contributions to the revision of the manuscript. All the authors read and approved the final manuscript.

Competing interests

The authors declare no competing interests.

Acknowledgments

This study was supported by the National Key Program of Research and Development, Ministry of Science and Technology (Grant No. 2022YFF1301500), the Guangdong Provincial Key Laboratory of Genome Read and Write (Grant No. 2017B030301011) and the Start-up Scientific Foundation of Northeast Forestry University (60201524043). This work was also supported by China National GeneBank (CNGB). We thank Kinanti Seraphina Larasati and Shiqing Wang for their help in reviewing and polishing the language. We thank Hui Liu and Tian Xia from the Shenzhen Safari Park Co., Ltd. for assisting with sample collection. Finally, we thank all the researchers (Shiqing Wang, Dongyi Yang, Jieyao Yu, Jiale Fan, Yuting Huang, Yingna Zhou, Tianlu Liu, Jiatong Cheng, Chen Lin and Shiyu Liu) involved in sample collection, genome sequencing and analysis.

References

1. Formenti G, Theissinger K, Fernandes C, Bista I, Bombarely A, Bleidorn C, et al. The era of reference genomes in conservation genomics. Trends in ecology & evolution. 2022;37 3:197-202. doi:10.1016/j.tree.2021.11.008.
2. Kitts PA, Church DM, Thibaud-Nissen F, Choi J, Hem V, Sapojnikov V, et al. Assembly: a resource for assembled genomes at NCBI. Nucleic acids research. 2016;44 D1:D73-80. doi:10.1093/nar/gkv1226.
3. Ouborg NJ, Pertoldi C, Loeschcke V, Bijlsma RK and Hedrick PW. Conservation genetics in

transition to conservation genomics. Trends in genetics : TIG. 2010;26 4:177-87. doi:10.1016/j.tig.2010.01.001.

4. Brandies P, Peel E, Hogg CJ and Belov K. The Value of Reference Genomes in the Conservation of Threatened Species. Genes. 2019;10 11 doi:10.3390/genes10110846.

5. Frankham R. Genetic rescue of small inbred populations: meta-analysis reveals large and consistent benefits of gene flow. Molecular ecology. 2015;24 11:2610-8. doi:10.1111/mec.13139.

6. Weeks AR, Heinze D, Perrin L, Stoklosa J, Hoffmann AA, van Rooyen A, et al. Genetic rescue increases fitness and aids rapid recovery of an endangered marsupial population. Nature communications. 2017;8 1:1071. doi:10.1038/s41467-017-01182-3.

7. Khan A, Patel K, Shukla H, Viswanathan A, van der Valk T, Borthakur U, et al. Genomic evidence for inbreeding depression and purging of deleterious genetic variation in Indian tigers. Proceedings of the National Academy of Sciences of the United States of America. 2021;118 49 doi:10.1073/pnas.2023018118.

8. Hu JY, Hao ZQ, Frantz L, Wu SF, Chen W, Jiang YF, et al. Genomic consequences of population decline in critically endangered pangolins and their demographic histories. National science review. 2020;7 4:798-814. doi:10.1093/nsr/nwaa031.

9. von Seth J, Dussex N, Diez-Del-Molino D, van der Valk T, Kutschera VE, Kierczak M, et al. Genomic insights into the conservation status of the world's last remaining Sumatran rhinoceros populations. Nature communications. 2021;12 1:2393. doi:10.1038/s41467-021-22386-8.

10. Xue Y, Prado-Martinez J, Sudmant PH, Narasimhan V, Ayub Q, Szpak M, et al. Mountain gorilla genomes reveal the impact of long-term population decline and inbreeding. Science. 2015;348 6231:242-5. doi:10.1126/science.aaa3952.

11. Nicolas D, Tom vdV, Hernán E. M, Christopher W. W, David D-d-M, Johanna vS, et al. Population genomics of the critically endangered kākāpō. Cell Genomics. 2021;1 1:100002. doi:10.1016/j.xgen.2021.100002.

12. Saremi NF, Supple MA, Byrne A, Cahill JA, Coutinho LL, Dalen L, et al. Puma genomes from North and South America provide insights into the genomic consequences of inbreeding. Nature communications. 2019;10 1:4769. doi:10.1038/s41467-019-12741-1.

13. Xie HX, Liang XX, Chen ZQ, Li WM, Mi CR, Li M, et al. Ancient Demographics Determine the Effectiveness of Genetic Purging in Endangered Lizards. Molecular biology and evolution. 2022;39 1 doi:10.1093/molbev/msab359.

14. Hua L, Gong S, Wang F, Li W, Ge Y, Li X, et al. Captive breeding of pangolins: current status, problems and future prospects. ZooKeys. 2015; 507:99-114. doi:10.3897/zookeys.507.6970.

15. Kondrashov P and Agadjanian AK. A nearly complete skeleton ofErnanodon(Mammalia, Palaeanodonta) from Mongolia: morphofunctional analysis. Journal of Vertebrate Paleontology. 2012;32 5:983-1001. doi:10.1080/02724634.2012.694319.

16. Choo SW, Rayko M, Tan TK, Hari R, Komissarov A, Wee WY, et al. Pangolin genomes and the evolution of mammalian scales and immunity. Genome research. 2016;26 10:1312-22.

17. Ferreira-Cardoso S, Billet G, Gaubert P, Delsuc F and Hautier L. Skull shape variation in extant pangolins (Pholidota: Manidae): allometric patterns and systematic implications. Zoological Journal of the Linnean Society. 2019; doi:10.1093/zoolinnean/zlz096.

18. Heinrich S, Wittman TA, Ross JV, Shepherd CR, Challender DWS and Cassey P. THE GLOBAL TRAFFICKING OF PANGOLINS: A comprehensive summary of seizures and trafficking routes from 2010–2015. Petaling Jaya: TRAFFIC, Southeast Asia Regional Office. 2017.
19. Zhang F, Wu S and Cen P. The past, present and future of the pangolin in Mainland China. Global Ecology and Conservation. 2022;33:e01995. doi:10.1016/j.gecco.2021.e01995.
20. Challender DWS, Harrop SR and MacMillan DC. Understanding markets to conserve trade-threatened species in CITES. Biological Conservation. 2015;187:249-59. doi:10.1016/j.biocon.2015.04.015.
21. Nijman V, Zhang MX and Shepherd CR. Pangolin trade in the Mong La wildlife market and the role of Myanmar in the smuggling of pangolins into China. Global Ecology and Conservation. 2016;5:118-26. doi:10.1016/j.gecco.2015.12.003.
22. Cheng W, Xing S and Bonebrake TC. Recent Pangolin Seizures in China Reveal Priority Areas for Intervention. Conservation Letters. 2017;10 6:757-64. doi:10.1111/conl.12339.
23. Zhang F, Yu Y, Wu S, Mahmood A, Yu J and Min Y. Reducing Pangolin Demand by Understanding Motivations for Human Consumption in Guangdong, China. Frontiers in Ecology and Evolution. 2020;8 doi:10.3389/fevo.2020.574161.
24. Wang Q, Lan T, Li H, Sahu SK, Shi M, Zhu Y, et al. Whole-genome resequencing of Chinese pangolins reveals a population structure and provides insights into their conservation. Communications biology. 2022;5 1:821. doi:10.1038/s42003-022-03757-3.
25. Wei S, Fan H, Zhou W, Huang G, Hua Y, Wu S, et al. Conservation genomics of the critically endangered Chinese pangolin. Science China Life Sciences. 2024; doi:10.1007/s11427-023-2540-y.
26. Cheng H, Concepcion GT, Feng X, Zhang H and Li H. Haplotype-resolved de novo assembly using phased assembly graphs with hifiasm. Nature methods. 2021;18 2:170-5.
27. Cheng H, Jarvis ED, Fedrigo O, Koepfli K-P, Urban L, Gemmell NJ, et al. Haplotype-resolved assembly of diploid genomes without parental data. Nature Biotechnology. 2022;40 9:1332-5.
28. Cao P, Dai Q, Deng C, Zhao X, Qin S, Yang J, et al. Genome-wide signatures of mammalian skin covering evolution. Science China Life Sciences. 2021;64 10:1765-80.
29. Damas J, Corbo M, Kim J, Turner-Maier J, Farré M, Larkin DM, et al. Evolution of the ancestral mammalian karyotype and syntenic regions. Proceedings of the National Academy of Sciences. 2022;119 40:e2209139119.
30. Heighton SP, Allio R, Murienne J, Salmona J, Meng H, Scornavacca C, et al. Pangolin genomes offer key insights and resources for the world's most trafficked wild mammals. bioRxiv. 2023; doi:10.1101/2023.02.16.528682.
31. Yan D, Luo X, Tang J, Xu S, Huang K, Wang X, et al. High-Quality Genomes of Pangolins: Insights into the Molecular Basis of Scale Formation and Adaption to Myrmecophagous Diet. Molecular biology and evolution. 2023;40 1 doi:10.1093/molbev/msac262.
32. Nie W, Wang J, Su W, Wang Y and Yang F. Chromosomal rearrangements underlying karyotype differences between Chinese pangolin (Manis pentadactyla) and Malayan pangolin (Manis javanica) revealed by chromosome painting. Chromosome research : an international journal on the molecular, supramolecular and evolutionary aspects of chromosome biology. 2009;17 3:321-9. doi:10.1007/s10577-009-9027-0.

33. Qi W, Lim YW, Patrignani A, Schlapfer P, Bratus-Neuenschwander A, Gruter S, et al. The haplotype-resolved chromosome pairs of a heterozygous diploid African cassava cultivar reveal novel pan-genome and allele-specific transcriptome features. GigaScience. 2022;11:giac028. doi:10.1093/gigascience/giac028.

34. Rhie A, McCarthy SA, Fedrigo O, Damas J, Formenti G, Koren S, et al. Towards complete and error-free genome assemblies of all vertebrate species. Nature. 2021;592 7856:737-46.

35. Kim H-K, Ham KA, Lee S-W, Choi HS, Kim H-S, Kim HK, et al. Biallelic deletion of pxdn in mice leads to anophthalmia and severe eye malformation. International Journal of Molecular Sciences. 2019;20 24:6144.

36. Segarra NG, Ballhausen D, Crawford H, Perreau M, Campos-Xavier B, van Spaendonck-Zwarts K, et al. NBAS mutations cause a multisystem disorder involving bone, connective tissue, liver, immune system, and retina. American Journal of Medical Genetics Part A. 2015;167 12:2902-12. doi:10.1002/ajmg.a.37338.

37. Kumamaru E, Kuo C-H, Fujimoto T, Kohama K, Zeng L-H, Taira E, et al. Reticulon3 expression in rat optic and olfactory systems. Neuroscience letters. 2004;356 1:17-20. doi:10.1016/j.neulet.2003.11.009.

38. Challender DW, Nash HC and Waterman C. Pangolins: science, society and conservation. Academic Press; 2019.

39. Wang Q, Lan T, Li H, Sahu SK, Shi M, Zhu Y, et al. Whole-genome resequencing of Chinese pangolins reveals a population structure and provides insights into their conservation. Communications Biology. 2022;5 1:821.

40. Wei S, Fan H, Zhou W, Huang G, Hua Y, Wu S, et al. Conservation genomics of the critically endangered Chinese pangolin. Science China Life Sciences. 2024:1-11.

41. Henn BM, Botigué LR, Bustamante CD, Clark AG and Gravel S. Estimating the mutation load in human genomes. Nature Reviews Genetics. 2015;16 6:333-43. doi:10.1038/nrg3931.

42. Low WY, Tearle R, Liu R, Koren S, Rhie A, Bickhart DM, et al. Haplotype-resolved genomes provide insights into structural variation and gene content in Angus and Brahman cattle. Nature communications. 2020;11 1:2071. doi:10.1038/s41467-020-15848-y.

43. Sun H, Jiao WB, Krause K, Campoy JA, Goel M, Folz-Donahue K, et al. Chromosome-scale and haplotype-resolved genome assembly of a tetraploid potato cultivar. Nature genetics. 2022;54 3:342-8. doi:10.1038/s41588-022-01015-0.

44. Zhang L, Lan T, Lin C, Fu W, Yuan Y, Lin K, et al. Chromosome-scale genomes reveal genomic consequences of inbreeding in the South China tiger: A comparative study with the Amur tiger. Molecular ecology resources. 2022; doi:10.1111/1755-0998.13669.

45. Yang S, Lan T, Zhang Y, Wang Q, Li H, Dussex N, et al. Genomic investigation of the Chinese alligator reveals wild-extinct genetic diversity and genomic consequences of their continuous decline. Molecular ecology resources. 2022; doi:10.1111/1755-0998.13702.

46. Wang Q, Lan T, Li H, Sahu SK, Shi M, Zhu Y, et al. Whole-genome resequencing of Chinese pangolins reveals a population structure and provides insights into their conservation. Communications Biology. 2022;5 1:821. doi:10.1038/s42003-022-03757-3.

47. Zhao S, Zheng P, Dong S, Zhan X, Wu Q, Guo X, et al. Whole-genome sequencing of giant pandas provides insights into demographic history and local adaptation. Nature

genetics. 2013;45 1:67-71. doi:10.1038/ng.2494.
48. Kuang WM, Ming C, Li HP, Wu H, Frantz L, Roos C, et al. The Origin and Population History of the Endangered Golden Snub-Nosed Monkey (Rhinopithecus roxellana). Molecular biology and evolution. 2019;36 3:487-99. doi:10.1093/molbev/msy220.
49. Hu J, Roos C, Lv X, Kuang W and Yu L. Molecular Genetics Supports a Potential Fifth Asian Pangolin Species (Mammalia, Pholidota, Manis). Zoological science. 2020;37 6:538-43. doi:10.2108/zs200084.
50. Guang X, Lan T, Wan Q-H, Huang Y, Li H, Zhang M, et al. Chromosome-scale genomes provide new insights into subspecies divergence and evolutionary characteristics of the giant panda. Science Bulletin. 2021;66 19:2002-13. doi:10.1016/j.scib.2021.02.002.
51. Pecnerova P, Garcia-Erill G, Liu X, Nursyifa C, Waples RK, Santander CG, et al. High genetic diversity and low differentiation reflect the ecological versatility of the African leopard. Current biology : CB. 2021;31 9:1862-71 e5. doi:10.1016/j.cub.2021.01.064.
52. Carneiro M, Albert FW, Afonso S, Pereira RJ, Burbano H, Campos R, et al. The genomic architecture of population divergence between subspecies of the European rabbit. PLoS genetics. 2014;10 8:e1003519. doi:10.1371/journal.pgen.1003519.
53. Liu B, Shi Y, Yuan J, Hu X, Zhang H, Li N, et al. Estimation of genomic characteristics by analyzing k-mer frequency in de novo genome projects. arXiv: Genomics. 2013.
54. Guan D, McCarthy SA, Wood J, Howe K, Wang Y and Durbin R. Identifying and removing haplotypic duplication in primary genome assemblies. Bioinformatics. 2020;36 9:2896-8.
55. Durand NC, Shamim MS, Machol I, Rao SS, Huntley MH, Lander ES, et al. Juicer provides a one-click system for analyzing loop-resolution Hi-C experiments. Cell systems. 2016;3 1:95-8.
56. Li H. Aligning sequence reads, clone sequences and assembly contigs with BWA-MEM. arXiv:13033997 [q-bioGN]. 2013;0 0:3.
57. Li H and Durbin R. Fast and accurate long-read alignment with Burrows–Wheeler transform. Bioinformatics. 2010;26 5:589-95.
58. Dudchenko O, Batra SS, Omer AD, Nyquist SK, Hoeger M, Durand NC, et al. De novo assembly of the Aedes aegypti genome using Hi-C yields chromosome-length scaffolds. Science. 2017;356 6333:92-5.
59. Manni M, Berkeley MR, Seppey M, Simão FA, Zdobnov EM and Kelley J. BUSCO Update: Novel and Streamlined Workflows along with Broader and Deeper Phylogenetic Coverage for Scoring of Eukaryotic, Prokaryotic, and Viral Genomes. Molecular biology and evolution. 2021;38 10:4647-54. doi:10.1093/molbev/msab199.
60. Rhie A, Walenz BP, Koren S and Phillippy AM. Merqury: reference-free quality, completeness, and phasing assessment for genome assemblies. Genome biology. 2020;21 1 doi:10.1186/s13059-020-02134-9.
61. Marçais G, Delcher AL, Phillippy AM, Coston R, Salzberg SL and Zimin A. MUMmer4: A fast and versatile genome alignment system. PLoS computational biology. 2018;14 1:e1005944.
62. Xu Z and Wang H. LTR_FINDER: an efficient tool for the prediction of full-length LTR retrotransposons. Nucleic Acids Res. 2007;35 Web Server issue:W265-8. doi:10.1093/nar/gkm286.
63. Flynn JM, Hubley R, Goubert C, Rosen J, Clark AG, Feschotte C, et al. RepeatModeler2 for

automated genomic discovery of transposable element families. Proceedings of the National Academy of Sciences of the United States of America. 2020;117 17:9451-7. doi:10.1073/pnas.1921046117.

64. Chen N. Using Repeat Masker to identify repetitive elements in genomic sequences. Current protocols in bioinformatics. 2004;5 1:4.10. 1-4.. 4.

65. Benson G. Tandem repeats finder: a program to analyze DNA sequences. Nucleic acids research. 1999;27 2:573-80. doi:10.1093/nar/27.2.573.

66. Stanke M, Steinkamp R, Waack S and Morgenstern B. AUGUSTUS: a web server for gene finding in eukaryotes. Nucleic acids research. 2004;32 Web Server issue:W309-12. doi:10.1093/nar/gkh379.

67. Majoros WH, Pertea M and Salzberg SL. TigrScan and GlimmerHMM: two open source ab initio eukaryotic gene-finders. Bioinformatics. 2004;20 16:2878-9. doi:10.1093/bioinformatics/bth315.

68. Korf I. Gene finding in novel genomes. BMC bioinformatics. 2004;5:59. doi:10.1186/1471-2105-5-59.

69. Kim D, Langmead B and Salzberg SL. HISAT: a fast spliced aligner with low memory requirements. Nature methods. 2015;12 4:357-60.

70. Pertea M, Pertea GM, Antonescu CM, Chang T-C, Mendell JT and Salzberg SL. StringTie enables improved reconstruction of a transcriptome from RNA-seq reads. Nature biotechnology. 2015;33 3:290-5.

71. Mount DW. Using the Basic Local Alignment Search Tool (BLAST). CSH protocols. 2007;2007:pdb top17. doi:10.1101/pdb.top17.

72. Campbell MS, Holt C, Moore B and Yandell M. Genome Annotation and Curation Using MAKER and MAKER-P. Current protocols in bioinformatics. 2014;48:4 11 1-39. doi:10.1002/0471250953.bi0411s48.

73. Jones P, Binns D, Chang H-Y, Fraser M, Li W, McAnulla C, et al. InterProScan 5: genome-scale protein function classification. Bioinformatics. 2014;30 9:1236-40. doi:10.1093/bioinformatics/btu031.

74. Lowe TM and Eddy SR. tRNAscan-SE: a program for improved detection of transfer RNA genes in genomic sequence. Nucleic acids research. 1997;25 5:955-64.

75. Goel M, Sun H, Jiao W-B and Schneeberger K. SyRI: finding genomic rearrangements and local sequence differences from whole-genome assemblies. Genome biology. 2019;20 1:1-13.

76. Robinson JT, Thorvaldsdóttir H, Turner D and Mesirov JP. igv. js: an embeddable JavaScript implementation of the Integrative Genomics Viewer (IGV). BioRxiv. 2022:2020.05. 03.075499.

77. Freed D, Aldana R, Weber JA and Edwards JS. The Sentieon Genomics Tools-A fast and accurate solution to variant calling from next-generation sequence data. BioRxiv. 2017:115717.

78. Purcell S, Neale B, Todd-Brown K, Thomas L, Ferreira MA, Bender D, et al. PLINK: a tool set for whole-genome association and population-based linkage analyses. The American journal of human genetics. 2007;81 3:559-75.

79. Yang J, Lee SH, Goddard ME and Visscher PM. GCTA: a tool for genome-wide complex trait analysis. The American Journal of Human Genetics. 2011;88 1:76-82.

80. Ortiz E. vcf2phylip v2. 0: convert a VCF matrix into several matrix formats for phylogenetic analysis. URL https://doi org/105281/zenodo. 2019;2540861.
81. Darriba D, Taboada GL, Doallo R and Posada D. jModelTest 2: more models, new heuristics and parallel computing. Nature methods. 2012;9 8:772. doi:10.1038/nmeth.2109.
82. Nguyen LT, Schmidt HA, von Haeseler A and Minh BQ. IQ-TREE: a fast and effective stochastic algorithm for estimating maximum-likelihood phylogenies. Molecular biology and evolution. 2015;32 1:268-74. doi:10.1093/molbev/msu300.
83. Alexander DH, Novembre J and Lange K. Fast model-based estimation of ancestry in unrelated individuals. Genome research. 2009;19 9:1655-64.
84. Terhorst J, Kamm JA and Song YS. Robust and scalable inference of population history from hundreds of unphased whole genomes. Nature genetics. 2017;49 2:303-9.
85. Schiffels S and Durbin R. Inferring human population size and separation history from multiple genome sequences. Nature genetics. 2014;46 8:919-25. doi:10.1038/ng.3015.
86. Browning SR and Browning BL. Rapid and accurate haplotype phasing and missing-data inference for whole-genome association studies by use of localized haplotype clustering. The American Journal of Human Genetics. 2007;81 5:1084-97.
87. Dobrynin P, Liu S, Tamazian G, Xiong Z, Yurchenko AA, Krasheninnikova K, et al. Genomic legacy of the African cheetah, Acinonyx jubatus. Genome biology. 2015;16 1:1-20.
88. Danecek P, Auton A, Abecasis G, Albers CA, Banks E, DePristo MA, et al. The variant call format and VCFtools. Bioinformatics. 2011;27 15:2156-8.
89. Wang K, Li M and Hakonarson H. ANNOVAR: functional annotation of genetic variants from high-throughput sequencing data. Nucleic acids research. 2010;38 16:e164-e.
90. Cingolani P, Platts A, Wang LL, Coon M, Nguyen T, Wang L, et al. A program for annotating and predicting the effects of single nucleotide polymorphisms, SnpEff: SNPs in the genome of Drosophila melanogaster strain w1118; iso-2; iso-3. Fly. 2012;6 2:80-92.
91. Grantham R. Amino acid difference formula to help explain protein evolution. science. 1974;185 4154:862-4.
92. Feng S, Fang Q, Barnett R, Li C, Han S, Kuhlwilm M, et al. The genomic footprints of the fall and recovery of the crested ibis. Current Biology. 2019;29 2:340-9. e7.
93. Davydov EV, Goode DL, Sirota M, Cooper GM, Sidow A and Batzoglou S. Identifying a high fraction of the human genome to be under selective constraint using GERP++. PLoS computational biology. 2010;6 12:e1001025.
94. Guo X, Chen F, Gao F, Li L, Liu K, You L, et al. CNSA: a data repository for archiving omics data. Database. 2020;2020 doi:10.1093/database/baaa055.
95. Chen F, You L, Yang F, Wang L, Guo X, Gao F, et al. CNGBdb: China National GeneBank DataBase. Hereditas (Beijing). 2020;42:799-809. doi:10.16288/j.yczz.20-080.

Manuscript that includes a version with tracked changes

Click here to access/download;Manuscript;MainText-highlight.docx

Enhancing inbreeding estimation and global conservation insights through HiFi assemblies of the Chinese and Malayan pangolin

Tianming Lan[1,2,3,†], Yinping Tian[3,†], Minhui Shi[3,†], Boyang Liu[3], Yu Lin[3],Yanling Xia[3], Yue Ma[3], Sahu Sunil Kumar[1], Qing Wang[3], Jun Li[2], Jin Chen[3], Fanghui Hou[4,5], Chuanling Yin[3], Kai Wang[2], Tengcheng Que[6,7], Wenjian Liu[6], Huan Liu[1], Haimeng Li[3,8,*] and Yan Hua[2,*]

[1] BGI Life Science Joint Research Center, Northeast Forestry University, Harbin 150040, China

[2] Guangdong Provincial Key Laboratory of Silviculture, Protection and Utilization, Guangdong Academy of Forestry, Guangzhou 510520, China

[3] College of Wildlife and Protected Area, Northeast Forestry University, Harbin 150040, China

[4] Guangdong Wildlife Rescue Monitoring Center, Guangzhou 510520, China

[5] Pangolin Conservation Research Center of National Forestry and Grassland Administration, Guangzhou 510520, China

[6] Faculty of Data Science City University of Macau, Macau 999078, China

[7] Guangxi Zhuang Autonomous Terrestrial Wildlife Rescue Research and Epidemic Diseases Monitoring Center, Nanning 530025, China

[8] Heilongjiang Key Laboratory of Complex Traits and Protein Machines in Organisms, Harbin 150040, China

*Correspondence address. Yan Hua, Guangdong Provincial Key Laboratory of Silviculture, Protection and Utilization, Guangdong Academy of Forestry, Guangzhou 510520 China. E-mail: wildlife530@hotmail.com; Haimeng Li, College of Wildlife and Protected Area, Northeast Forestry University, Harbin 150040, China. E-mail: lihaimeng66@163.com

†Equal contributions.

Abstract

A high-quality reference genome coupled with resequencing data is ~~becoming~~ a promising strategy to address issues in conservation genomics~~,~~. This~~which~~ has greatly enhanced the development of conservation plans for endangered species. Pangolins are fascinating animals with a variety~~range~~ of ~~distinctive~~ unique features~~, but unfortunately~~. Unfortunately, they are the ~~world's~~ most trafficked wild animal in the world. In this study~~animals. Here~~, we ~~report~~ assembled a ~~haplotype-resolved and~~ chromosome-scale genome with HiFi long reads for ~~each of~~ the Chinese ~~pangolin~~ and Malayan pangolin and provided~~, providing~~ two new~~the most~~ representative reference genomes for the pangolin species. We found a great~~er~~ improvement in the evaluation of genetic diversity and inbreeding based on these high-quality genomes and obtained different results for the detection of ~~in detecting~~ genome-wide extinction risks ~~being~~ compared with genomes assembled ~~by~~using short reads~~ assembled genomes~~. Moderate inbreeding and genetic diversity were reverified ~~again~~ in these two pangolin species, except for one Malayan pangolin population with ~~the~~ high inbreeding and low genetic diversity~~, which we recommend to pay special attention to the conservation and protection of this population~~. ~~Furthermore~~Moreover, we identified~~found~~ a much higher inbreeding level (F_{ROH}=0.54) in the Chinese pangolin individual from Taiwan Province compared with~~than~~ that from mainland China, but more than 99.6% runs of homozygosity (ROH)~~ROH~~ fragments were restricted to ~~that~~ less than 1Mb, indicating that the high F_{ROH} in Taiwan Chinese pangolins may have ~~been~~ accumulated from ~~the~~ historical inbreeding events. Furthermore~~Additionally~~, our study is the first to detect relatively mild genetic purging in pangolin populations~~ that were analyzed~~. These two ~~high~~ high-quality reference genomes will provide valuable ~~genomic~~ genetic resources for future studies ~~on~~ and contribute to the protection and conservation ~~for~~ of pangolins.

Keywords: Chinese pangolin; Malayan pangolin; inbreeding; genetic purging; conservation genomics

Introduction

~~From the Human Genome Project to the first Telomere-to-Telomere genome, the human genome provides an excellent example of the evolution of a reference genome and how its continuous improvement has shaped~~

Formatted: Font: 9 pt

Formatted: Not Highlight

Formatted: Not Highlight

Formatted: Subscript

~~significant advancements in biology, medicine, and other related fields. The rapid development of third-generation sequencing technologies over the past decade has further revolutionized the quality of genome assembly. This has been accomplished by incorporating long-read (usually >10 kb) capable of spanning complex structures, like complex structure variants, telomers, segmental duplications, or centromer Taking benefits from the decreasing sequencing cost, advanced computational power, and continuously improving assembly algorithms, a large number of high-quality reference genomes have been assembled by long-read sequencing technologies have been released.~~

High-quality reference genomes enable the comprehensive ~~analyses~~ analysis of population genomics~~,~~ and ~~are~~ contribute~~promising~~ to the revolution in~~revolutionize~~ conservation genomics[1]. However, fewer than 1% of the threatened species listed on the International Union for Conservation of Nature (IUCN) red list have a reference genome[2], and this number will further decrease~~s,~~ if long-read assembly is considered. ~~Against the backdrop of~~In the shift from conservation genetics to conservation genomics[3], high-quality reference genomes play an important~~a critical~~ role in providing the necessary support for the conservation of endangered species[1, 4]. In particular, ~~the~~ genetic rescue is considered ~~as~~ an important strategy to facilitate gene flow and avoid ~~further~~ inbreeding to increase the health of a population ~~fitness~~ [5, 6]. A deep understanding of the genome-wide extinction risks and the basic genetic background ~~for~~ of a ~~given~~ small population is ~~key~~ necessary for ~~making~~ developing evidence-based strategies~~plans~~ for genetic rescue. These include population structure, genomic diversity, genome-wide inbreeding, ~~and~~ mutational load ~~and inbreeding depression~~, ~~local adaptation, genome-wide mutational load,~~ and population demography [7-11]. A high-quality reference genome usually facilitates population-level studies ~~that are closely related to~~in conservation genomics[1]. For example, the evaluation of inbreeding by measuring ~~runs of homozygosity (~~ROH ~~)~~ ~~highly depends~~relies on ~~the~~ a high-quality reference genome with outstanding contiguity~~. This is due to~~ , because the long ROH fragments in small populations with high-level inbreeding often span~~ning~~ ~~over~~ several millions of base pairs [11-13], and can ~~be~~ hardly be detected based on the fragmented genomes assembled by short reads.

The ~~P~~pangolin is a ~~mammal of~~ living fossil~~prehistoric~~ ~~mammal~~ with many unique biological characteristics [14, 15], such as overlapping keratin scales ~~over~~ covering the body, a ~~highly~~ specialized diet, a long and muscular tongue, a sensitive olfactory system, and burrowing ability [16, 17]. Locals across its distribution areas ~~However, pangolin's scale and meat~~ have ~~been~~ traditionally used its scales and meat for medicine and food ~~by locals across its distribution areas~~ [18]. The overexploitation of pangolins driven by the soaring demand for luxury food and traditional Chinese medicine, ~~is pushing~~has pushed this animal to the edge of extinction [19-22]. Currently~~At present~~, the pangolin ~~has been~~is the most heavily trafficked wild animal ~~globally~~worldwide, with more than 900,000 individuals poached over the last two decades and 67 countries from six continents involved in illegal poaching and trade [23]. For ~~better~~ protection, all ~~eight~~ pangolin species have been ~~uplisted in~~placed into Appendix I of the Convention of International Trade of Endangered Species of Wild Fauna and Flora (CITES) ~~since~~ as of 2016. Poaching is more rampant for Asian pangolins ~~than for~~compare with African pangolins, particularly~~especially~~ the Malayan pangolin (*Manis javanica*, hereafter MJ) and the Chinese pangolin (*Manis pentadactyla*, hereafter MP), which are under extreme survival pressure ~~due to a great amount~~because of significant poaching and trafficking [8]. These two species have been listed as "Critically Endangered" on the ~~International Union for Conservation of Nature (~~IUCN ~~)~~ Red List since 2014.

Previous studies identified two main Malayan populations[8] (hereafter ~~MJ1~~MjavA and ~~MJ2~~MjavB) and three main Chinese pangolin populations[8, 24, 25] (hereafter ~~CPA~~MpenA, ~~CPB~~MpenB and ~~CPC~~MpenC). The ~~MJ1~~MjavA population was identified from the mainland (China and Myanmar) and diverged from the ~~MJ12~~MjavB population, which may have~~possibly~~ originated from the Southeast Asian islands. Among the three Chinese pangolin populations,

the ~~CPA~~MpenA population is a newly discovered population distributed in Guangdong, China. The ~~CPB~~MpenB population is distributed ~~in~~ over a vast area, including southern China and Thailand~~; and the~~. The ~~CPC~~MpenC population highly diverged from the other two populations, which are ~~very~~ likely to have originated from Myanmar (Fig. 1A, Supplementary Table S1 and S2). Although the varying population ~~differentiation~~ of the Chinese pangolin is still controversial~~in {debate~~ [25], ~~T~~the highly divergent pangolin populations indicate that deep isolation may have occurred, which is usually detrimental to the survival of endangered species.

Pacific Biosciences (PacBio) high-fidelity (HiFi) sequencing technology combined with a HiFi-specific assembler and Hi-C or parental sequencing data, can generate high-quality ~~and~~ , haplotype-resolved *de novo* assemblies, which represents~~representing~~ one of the most promising strategies for genome ~~assembling~~ assembly [26, 27]. This technique may ~~further~~ facilitate a more ~~comprehensive and~~ accurate ~~evaluations~~ analysis of genome-wide genetic risks, specifically ~~for the evaluation of~~with respect to inbreeding. Because of~~Given~~ the urgent need for establishing ~~the investigation of~~ genomic backgrounds to support ~~their~~ conservation, several reference genomes of pangolins have been assembled, annotated, and published[8, 16, 28-31]. However, high-quality reference genomes assembled from HiFi long-reads ~~are non-existent~~do not exist for pangolins. In this ~~report~~study, we present the genomes of the Malayan and Chinese pangolins~~, both~~ at the chromosome-scale with haplotypes resolved down to the chromosomal-level.~~Here we report two chromosome-scale and haplotype-resolved genomes of the Malayan and Chinese pangolin. These genomes are~~ , ~~providing~~which provides ~~the highest-quality and the most~~new representative reference genomes for the pangolin species~~to date~~. We systematically examined the~~investigated~~ genomic backgrounds and evaluated the genome-wide extinction risks for ~~the~~ five pangolin populations based on these two~~Based on the superiority of high-quality~~ reference genomes~~in population genomics.~~, ~~we systematically investigated genomic backgrounds and evaluated genome-wide extinction risks for the five pangolin populations.~~

Formatted: fontstyle01, Font: 10.5 pt

Results

New representative ~~The first haplotype-resolved~~reference ~~chromosomal-level~~ genomes ~~assembly~~ for the pangolin

To obtain high-quality reference genomes for both the Malayan ~~pangolin~~ and Chinese pangolin, we combined PacBio HiFi long reads, Hi-C reads and DNBSEQ short reads for ~~the~~ genome assembly (~~Fig~~Table. 1~~B~~). We first generated phased contigs for both species ~~(Table 1),~~ and the haploid-resolved contigs were further linked at the chromosome-level by combining the HiFi long reads and Hi-C reads. We ~~finally~~ assembled 19 and 20 chromosome-scale pseudomolecules for MJ and MP, respectively (Fig. 1~~C~~B, Supplementary Fig. S1), which was~~. This finding is~~ consistent with the karyotypic analysis[32], ~~even though~~although the karyotype in pangolins ~~is~~ may ~~be variable~~vary[32]. The diploid genome sizes ~~we~~ assembled for MJ and MP were ~2.56 Gb and ~2.64 Gb, and represented~~ing~~ 99.98% and 96.01% of the estimated genome size (~2.56 Gb for MJ and ~2.75 Gb for MP), respectively (Supplementary Fig S2). The contig and scaffold NG50 of the MJ/MP were 46.22Mb/56.16 Mb and 141.80 Mb/140.71 Mb, respectively (Table 1, Supplementary Table S3)~~-~~. ~~Furthermore~~In addition, we identified the X-chromosome and Y-linked regions ~~in~~ of both genomes (Supplementary Fig. S3). The hifiasm assembler simultaneously ~~resulted~~yielded in two groups of haplotigs for each of the MJ (hereafter MJH1, MJH2) and MP (hereafter MPH1, MPH2) genomes (Supplementary Table S4). Multiple lines of evidence support~~ed~~ the high completeness and ~~a~~ low level of artificial duplication of the haplotype-resolved assemblies. Both the base-level quality evaluation and the structural-level assessment showed that all genomes (2 diploid assemblies and 4 haploid assemblies) had high assembly accuracy (see the Supplementary Material online). High collinearity between the MP and MJ genomes was also observed with 4 fissions and 3 fusions in the MJ genome compared with the MP genome

Formatted: Not Highlight

(Fig. 1B), which was consistent with a previous karyotypic analysis [47] showing that these two genomes were accurately assembled at the chromosome level. Overall, we are confident in these two new, high-quality, and representative reference genomes for pangolins.

We also found high collinearity between the MP and MJ genomes, with 4 fissions and 3 fusions in the MJ genome when compared to the MP genome (Fig. 1BSupplementary Fig. S6a), which was consistent with previous karyotypic analysis[32], showing that these two genomes were accurately assembled at the chromosome level. Overall, we are confident that we provided two new, high-quality, and representative reference genomes for pangolins.

Table 1: Summary Sstatistics for the Ggenome Ssequences

Category	Metric	Chinese pangolin (MP)	Malayan pangolin (MJ)
**Sequencing Data**	WGS (Gb)	254.05	245.34
	HiFi (Gb)	65.81	95.99
	Hi-C (Gb)	239.77	227.48
	RNA-seq (Gb)	6.21	6.35
**Continuity**	Scaffold NG50[a] (Mb)	140.71	141.80
	Scaffold number	89	62
	Longest scaffold (Mb)	234.25	241.98
**Structural accuracy**	Reliable blocks[b]	97.16%	99.14%
	False duplications[c]	0.40%	0.48%
	Curation	manual	manual
**Base accuracy**	Base pair QV	57.06	55.08
	k-mer completeness	96.81	95.40
**Functional completeness**	BUSCO[d] assessment	98.00% complete	97.50% complete
	Transcript mappability	97.77%	97.26%
**Chromosome status**	Assigned[de]	97.22	98.72
	Pseudo-chromosomes Number	20	19

[a]Scaffold NG50: This metric represents the minimum length of a scaffold such that when all scaffolds are ranked by size, the cumulative length of scaffolds exceeding this threshold accounts for at least half of the estimated genome size. [b]Reliable blocks:These refer to genomic regions that are robustly supported by a minimum of 10 PacBio HiFi reads, ensuring a reliable assembly[33]. [c]False duplications:These are additional copies of k-mers present in a genome assembly beyond the expected count, as indicated by the k-mer histogram derived from the original high-fidelity reads[34]. [d]BUSCO: Benchmarking Universal Single-Copy Orthologs . [e]Assigned: This term denotes the percentage of the genome assembly that has been confidently assigned to specific chromosomes.

Item	Category	Chinese pangolin (MP)	Malayan pangolin (MJ)
**Sequencing Data**	WGS (Gb)	254.05	245.34
	HiFi (Gb)	65.81	95.99
	Hi-C (Gb)	239.77	227.48
	RNA-seq (Gb)	6.21	6.35
**Genome Assemblies**	Estimated genome size (Gb)	2.75	2.56
	Assembled genome size (Gb)	2.64	2.56
	Contig number	294	154
	Contigs N50 (Mb)	40.35	61.24
	Longest contig (Mb)	184.35	123.33
	Scaffold number	89	62
	Scaffolds N50 (Mb)	140.71	141.8
	Longest scaffold (Mb)	234.25	241.98
	Pseudo-chromosomes Number	20	19
	Percentage of Pseudo-chromosomes to the assembly	96.97%	98.48%
**Assembly Quality**	Assembly error rate	1.09E-05	1.39E-05
	QV score for bases in the assembly	49.63	48.56
	Repeat content	41.62%	37.62%
	BUSCO assessment	98.00%	97.50%

Formatted Table

Formatted: Superscript

Formatted: Superscript

Formatted: Superscript

The hifiasm assembler simultaneously resulted in two groups of haplotigs for each of the MJ (hereafter MJH1, MJH2) and MP (hereafter MPH1, MPH2) genome (Supplementary Table S4 and S5). Merqury k-mer analysis indicated high completeness and a low level of artificial duplication for haplotype-resolved chromosome-level genomes of both MJ and MP (Supplementary Fig. S4). The k-mer spectra plot showed that homozygous regions (shared k-mers) and heterozygous regions (haploid specific k-mers) mainly consisted of two-copies (~82X/78X) and one-copy (~40X/38X) k-mers (Supplementary Fig. S4). Further, the sequencing depths of the two groups of haplotigs were consistent for both genomes evaluated by PacBio HiFi reads and DNBSEQ reads (Supplementary Fig. S5), implying the completeness of haplotype-resolved assemblies. As expected, the k-mer completeness for all haploid and diploid genomes attained a very high level (Supplementary Table S65).

The base-level quality evaluation showed that all genomes (2 diploid assemblies and 4 haploid assemblies) had high assembly accuracy with the lowest QV score of 48.33, corresponding to an accuracy higher than 99.99% (Supplementary Table S65). Besides, all these assemblies showed high BUSCO scores, with 97.5% and 98.0% BUSCO genes identified in the MJ and MP diploid genomes, respectively. For haploid genomes, the BUSCO score was still as high as 94.8 % even for the lowest MPH1 (Supplementary Table S76). The genome mapping of DNBSEQ short reads, Hi-C short reads, and RNA sequencing data to genome assemblies also presented high mapping rates, especially DNBSEQ short reads, with the mapping rate of 99.91% and 99.93% for MJ and MP genome, respectively (Supplementary Table S87). Finally, we assessed the structural-level accuracy by mapping long PacBio HiFi reads to the haploid genomes and found more than 99.24%/99.27% and 98.61%/97.38% of MJ/MP genomes were identified to be correctly assembled with a criterion of ≥10 X long reads mapping at a single position.

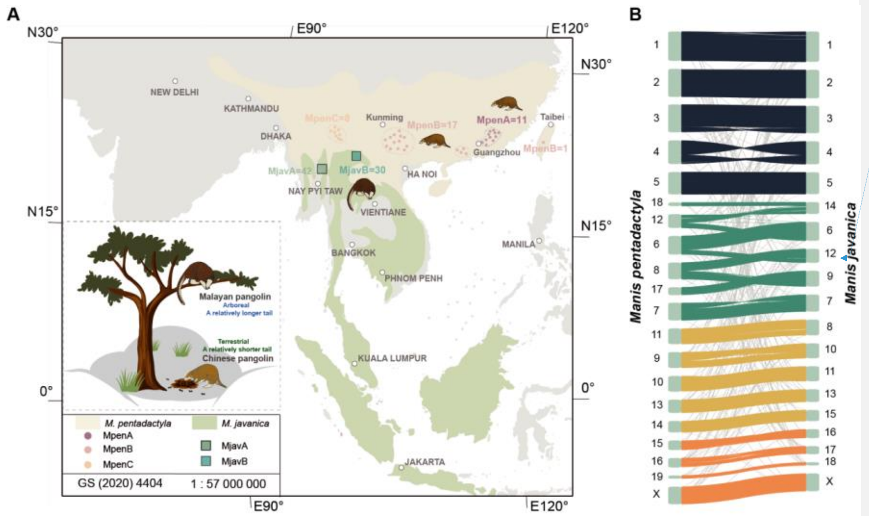

Formatted: Centered, Indent: Left: 0.15", Right: 0.15"

Figure 1: Introduction to the species distribution and chromosome synteny of the Chinese and Malayan pangolins. Genomic landscape of the Malayan pangolin and Chinese pangolin genomes. (A) The distribution area and sampling sites of the Chinese and Malayan pangolins in this study. The circles represent sampling sites of the Chinese pangolins reported byin Wang *et al*.,[24], and samples Samples without detailed locations were are not showed shown on thethis map. (B) The Malayan pangolin and Chinese pangolin with their species-specific biological characteristics. (C) Brief introduction and circos diagram of the two pangolin genomes. (D) The chromosome-scale synteny analysis between the Malayan pangolin and Chinese pangolin genomes.

We also found high collinearity between the MP and MJ genomes, with 4 fissions and 3 fusions in the MJ genome when compared to the MP genome (Supplementary Fig. SFig. 16aD), which was consistent with previous karyotypic analysis[32], showing that these two genomes were accurately assembled at the chromosome level. Overall, we are confident that we provided two new, high quality, and representative reference genomes for pangolins.

Genome annotation

The total length of the repeat elements reached 1,260.92 Mb and 1,332.41 Mb, accounting which accounted for 49.25% and 50.44% of the MJ and MP genomes, respectively (Supplementary Table S958)). The composition of repeats in the MJ and MP genomes was similar, with the most abundant repeat element being LINE (MJ: 35.41%, MP: 37.36%), followed by LTR (MJ: 11.14%, MP: 13.61%), DNA element (MJ: 2.23%, MP: 2.11%) and SINE (MJ: 0.96%, MP: 0.91%). We predicted 19,680 and 19,886 gene models in the MJ and MP genomes, respectively (Supplementary Table S1169). The gene regions spanned over 853.90 Mb and 791.49 Mb, which comprisedcomposing 33.36% and 29.96% of the MJ and MP genomes, respectively (Supplementary Table S6911). The average gene length, exon length, and intron length were 43.39 kb, 175.75 bp and 4.92 kb for the MJ genome, and these three parameters were 39.80 kb, 175.07 bp and 4.64 kb for the MP genome (Supplementary Table S1169). The BUSCO analysis showed revealed high completeness for the gene sets of both genomes, with the lowest BUSCO score higher greater than 956% (Supplementary Table S1720). Overall, 19,575 (99.47%) and 19,792 (99.53%) genes were functionally annotated respectively in the MJ and MP genomes, respectively (Supplementary Table S1831). In addition, we predicted 288/435 rRNA, 1,296/1,348 miRNA, 806/350 tRNA, and 1,521/1,412 snRNA in the MJ/MP genomes (Supplementary Table S1942).

Comparisons of the genomic landscape between haploid chromosomes

In general, the two haploid genomes of MJ or MP were found to be very similar. The sequence differences (100bp window) showed one peak in the histogram for both MJ and MP genomes, indicating that the proportion of identical sequences between haploid genomes were dominant (Supplementary Table S15,3 Supplementary Fig. S67). Synteny analysis showed clear one-to-one syntenic blocks between homologous haploid chromosome pairs of the two pangolin species (Supplementary Fig. S76b and S6c8), further showing the high similarity between haploid genomes, which was also reflected in the k-mer analysis with almost all k-mers were shared between haploid genomes (Supplementary Fig. S4).

Nonetheless, we detected many chromosomal SVs (>50bp) between MJH1 and MJH2, and between MPH1 and MPH2. In general, we found 5,233 (3,681 duplications, 534 translocations, 509 inversions and 509 deletions) and 8,957 (7,738 duplications, 760 translocations, 269 inversions and 190 deletions) SVs between the haploid genomes of MJ and MP (Fig. 1C, Supplementary Table S164). All these SVs were validated by our assembled contigs (Supplementary Fig. S98). We found 2,136 genes that were distributed in the SVs of the MJ genome, while 4,264 such genes were found in the MP genome (Supplementary Fig. S910-S13, Supplementary Table S175-S197). It is worth noting that four genes were disrupted by breakpoints of SVs and had become pseudogenic in one of the two haploid chromosomes for the MJ, and we further found 20 such genes in the MP genome. Interestingly, several of these genes in the MP genome were vision-related, such as *PXDN*, *NBAS* and *RTN3*. *PXDN* gene is closely related to eye development, and the loss of *PXDN* gene in mice results in severe eye disorders, including drastically disorganized eye structures and the absence of eyeballs[35]. *NBAS* gene is estimated to correlate with retinal homeostasis[36]. *RTN3* gene is likely to play an important role in retinal function, as this gene's mutation causes retinal dystrophies[37]. Some of the other genes were found to be related to immunity and metabolism (Supplementary Table S2018).

The HiFi genome improves the evaluation of genetic diversity and inbreeding

We compared the short-read assembled genome (hereafter SG, the YNU_ManPten_2.0 and YNU_ManJav_2.0 were used here to represent the Chinese pangolin and Malayan pangolin genome, respectively) and the long-read assembled genome (PacBio HiFi assembled genome in this study, hereafter long-read assembled genome (LG))~~LG)~~ to evalute for the evaluation of commonly used genetic parameters in population genomics, particularly in conservation genomics, including population structure, population history and separation, genetic diversity, and inbreeding (Supplementary Table S~~21~~109). ~~In this comparison, we~~We found that the population structure (principal component analysis (PCA)~~PCA~~, phylogenetic tree, and admixture), population history, and population separation (inferred by MSMC2) were ~~almost~~ not significantly affected by ~~the quality of the reference~~these two types~~kinds~~ of reference genomes (Supplementary Fig. S4-S7), because ~~these~~the results calculated based on the LG and SG were ~~totally~~ the same~~,~~ and ~~very~~ consistent with previous reports. ~~However, in~~In contrast to other studies, however, we did not ~~find~~observe a ~~very~~ distinct separation between the individual from Taiwan Province and other Chinese pangolins, although the Chinese pangolin in Taiwan Province ~~wa~~is considered ~~as~~ a subspecies of the Chinese pangolin[38]~~,~~ ~~which could be explained as,~~ Therefore, either the genetic differentiation is still relatively small between the Chinese pangolin in Taiwan Province and mainland China, or the~~that this~~ Taiwan individual was recently translocated from mainland China. ~~.~~For genetic diversity, the difference between π values calculated by LG ~~(long-read HiFi assembled genome)~~ and by SG ~~(short-read assembled genome)~~ was not large~~, but~~; however, this difference ~~is~~ was significant, with a ~~significantly~~ ~~with~~ higher π value ~~π values~~ calculated based on the LG ~~than~~compare with that ~~calculated based on~~for the SG (Fig. 2A, Supplementary Table S1~~9~~0). Moreover, ~~T~~the ROH is an important genetic factor that ~~can~~ reflects the inbreeding level in a population, ~~while~~whereas it was very sensitive to the quality of the reference genome. ~~It is undeniable that the LG could reflect much more detailed information on genetic diversity than SG (Supplementary Table S2119). Furthermore, b~~By screening ROHs across the genome to evaluate ~~the~~ genome-wide inbreeding, we ~~found more~~identified significant differences between the SG and LG (Table S11-S12). ~~The ROH is an important genetic factor that can reflect the inbreeding level in a population, while it was very sensitive to the quality of the reference genome.~~ For the Malayan pangolin populations, ~~we found that~~ the F_{ROH} calculated ~~by~~ based on the LG ~~were obviously~~was markedly higher ~~than~~ compare with those calculated ~~by~~ based on the SG (Fig. 2B, Supplementary Table S13). However, for the Chinese pangolin, this difference was ~~found to be~~ significant only for ROHs larger than 1 Mb, although F_{ROH} ~~were~~ was larger for the LG ~~under~~ at other ~~conditions~~ ROH lengths (Fig. 2C).

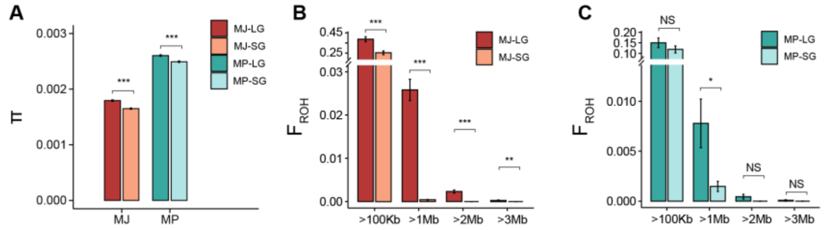

Figure 2: Comparison~~s~~ of the genome-wide genetic diversity and inbreeding estimated based on the LG (long-read HiFi assembled genome) and SG (short-read assembled genome). (A) ~~The c~~Comparison of genome-wide π calculated based on the LG and SG in the Chinese and Malayan pangolin genomes. (B) Comparison of F_{ROH} calculated based on LG and SG in the Malayan pangolin genomes. (C) Comparison of F_{ROH} calculated based on LG and SG in the Chinese pangolin genomes. Note: NS: p ≥ 0.5, *p < 0.05, **p < 0.01, ***p < 0.001.

Genome-wide genetic diversity and inbreeding

Considering the improvement in evaluating the genetic diversity and inbreeding based on the high-quality H~~I~~iFi~~I~~ genomes, we performed a~~the~~ reassessment based on the LG and found that the genome-wide genetic diversity (π) of the Chinese pangolin and Malayan pangolin~~, based on the HiFi genome~~ were 0.0026 and 0.0018, respectively, which were both higher ~~than~~ compare than those calculated based on the SG (π_{MP}=0.0025, π_{MJ}=0.0016) (Supplementary

Formatted: Font color: Auto
Formatted: Font: 9 pt, Font color: Auto
Formatted: Font color: Auto
Formatted: Font: 9 pt, Font color: Auto
Formatted: Font color: Auto
Formatted: Not Highlight
Formatted: Not Highlight
Formatted: Not Highlight
Formatted: Not Highlight
Formatted: Font: (Default) Times New Roman, 9 pt
Formatted: Not Highlight
Formatted: Not Highlight
Formatted: Not Highlight
Formatted: Highlight

Table S21109). For the Chinese pangolin populations, the CPBMpenB exhibitedharbored the highest genetic diversity ($\pi_{CPBMpenB}$ = 0.0020), followed by the CPCMpenC ($\pi_{CPCMpenC}$ = 0.0018) and CPAMpenA ($\pi_{CPAMpenA}$ = 0.0017), which was consistent with previous study, but with higher π values[39]. Among the Malayan pangolin populations, the genetic diversity of MJ1MjavAB ($\pi_{MJ1MjavA}$ = 0.0024) was obviously higher than that of the MJ2MjavBA ($\pi_{MJ2MjavB}$ = 0.0007). The mean *He* of the MjavA and MjavB were 0.063% and 0.189%, respectively, which were higher than the 0.043% and 0.141% values in a previous study[8]. The average genetic diversity of the Chinese pangolin was higher compared with that ofthan the Malayan pangolin (Supplementary Table S21109).

Formatted: Font: Italic

Inbreeding in small populations increases genome-wide homozygosity, and the resulting depression accelerates the loss of genetic diversity. In For the MJ and MP populations, the average number of ROH fragments in each individual was 3,659.22±215.96 and 1,543.43±186.90, respectively. For both species, the ROHs were restricted to relatively small fragments (<1Mb), and the number of ROHs larger longer than 1Mb only accounted for 0.94% and 1.27% of the ROH fragments for the MP and MJ genomes, respectively[8]. The total length of ROHs larger than 1Mb also accounted for a small proportion in of the two genomes (MP: 5.19%; MJ: 6.69%) (Fig. 3A and 3B). [40]We did not find observe any ROH fragments larger greater than 5Mb in both either species. The F_{ROH} is sSimilar to the ROH numberly, with the F_{ROH} in the MJ genomes (0.39±0.024) was higher than that in the MP genomes (0.15±0.023)values being of 0.39±0.024 and 0.15±0.023 in the MJ and MP populations, respectively (Supplementary Table S25 and S26213). For ROHs longer than 1Mb, however, the , and F_{ROH} longer than 1Mb was sharply reduced to 0.026±0.003 and 0.0078±0.002 for the MJ and MP, respectively (Supplementary Table S25 and S21362), which were much lower than that reported in a previous study[8, 40]. The very high minor allele frequency of 0.2 and other abnormally stringent filtering parameters used in the previous study as a threshold to filter the SNP couldmay severely dilute the SNPs across the genome, resulting in an overestimation ofand then may overestimate the inbreeding in the genome.

We further compared the ROH distribution in different populations of these two species (Supplementary Fig. S1985-S22198). In For Malayan pangolins, the inbreeding in the MJ2MjavBA population (F_{ROH}=0.55±0.007) was more serious than compare with that in the MJ1MjavAB population (F_{ROH}=0.15±0.012) (Fig. 3CB-3D, Supplementary Table S25213). Although this difference became smaller for ROH largerROHs greater than 1Mb, it was still significant (Fig. 3D). Among the three Chinese pangolin populations, the inbreeding of the CPAMpenA (F_{ROH}=0.18±0.036) and CPBMpenB (F_{ROH}=0.17±0.039) populations were comparable (CPAMpenA: 0.18±0.036; CPBMpenB: 0.17±0.039), but were much worse than compare with that of the CPCMpenC population (F_{ROH}=0.06±0.005) (Fig. 3CA and 3E, Supplementary Table S21362). }As the Malayan pangolin populationsSimilarly, the differences of in inbreeding levelF_{ROH} among between these three MP populations is were reduced for ROHs larger longer than 1Mb (Fig 3E, Supplementary Table S13262). NoteworthyAs reported in a previous study[8], we also found that the F_{ROH} of the Taiwan individual (F_{ROH}=0.54) is was much higher than all other individuals in the MP population (F_{ROH}=0.54) (Fig. 3C). NoteworthyNotably, we found in this study that 99.6% of , the ROH fragments in the genome of the Taiwan individual although most of the ROH fragments were less than 1Mb, which was much higher than that reported byin the previous study (Supplementary Table S24113, Supplementary Fig. S1620). AdditionallyIn addition, the F_{ROH} varies varied greatly among individuals in the CPAMpenA or CPBMpenB population, but this was not found observed in the MpenC, MjavA and MjavBother three populations.

Formatted: Subscript

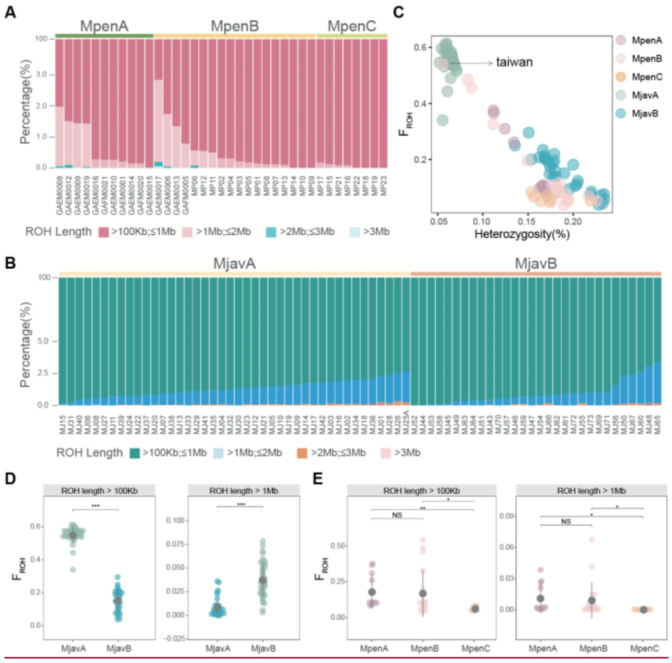

Figure 3: Genome-wide inbreeding estimated by ROH in the Chinese and Malayan pangolin populations. (A) The length distribution of ROH across the genome in the Chinese pangolin population. (B) The length distribution of ROH across the genome in the Malayan pangolin population. (C) Genome-wide heterozygosity and inbreeding estimates (F_{ROH}) for all five pangolin populations. (D) ~~The c~~Comparison of the averaged F_{ROH} in the ~~MJ1~~MjavA and ~~MJ2~~MjavB populations of the Malayan pangolins. (E) ~~The c~~Comparison of the averaged F_{ROH} in the ~~CPA~~MpenA, ~~CPB~~MpenB and ~~CPC~~MpenC populations of Chinese pangolins.

~~The genome~~Genome-wide mutational load

The mutational load is the burden of deleterious variants carried by a population, and ~~can~~ reflects the evolutionary fitness of a population[41]. Although studies have ~~been explored~~ examined the distribution of mutational loads for the Chinese and Malayan pangolins[8, 24, 40], the ~~HIFI~~ HiFi genomes in this study provide~~d~~ new insights~~s~~ into the accumulation of mutational load in pangolins. ~~Here, w~~We screened three categories of mutational load (loss of function, ~~LOF~~LoF; missense mutation; deleterious nonsynonymous mutation, dnsSNP) based on the HiFi genomes for ~~both~~ the Chinese and Malayan pangolins (Fig.4A and 4B, Supplementary Table S14~~27~~3 and S15~~28~~4). We calculated the individual-level derived mutational load ~~in~~ for each population to avoid bias introduced by different population sizes ~~and~~. In a previous study, the MpenC population was ~~reported~~ to harbor the most mutational load. In this study, however, we found ~~found~~ that individuals in the ~~CPC~~MpenC population harbored the most mutational load, which was significantly more than that in the ~~CPB~~MpenB and ~~CPA~~MpenA populations (Fig. 4A). This may be ~~largely due to~~ the result of the HiFi reference genome ~~we~~ used in this study and the different methods for the identifying the derived allele across the genome. –The missense mutations and dnsSNPs in the ~~CPA~~MpenA population were comparable to those in the ~~CPB~~MpenB population (Supplementary Fig. S10a and S10b~~23~~~~19a and S19b~~)~~, but~~; however the ~~CPB~~MpenB population harbored many ~~many~~ more ~~LOF~~LoFs ~~than did~~compared with the ~~CPA~~MpenA population (Fig. 4A). ~~We then~~Next, we focused on the derived homozygous mutational load (DHMD)~~the derived homozygous mutational load (DHMD)~~ and found that the ~~CPC~~MpenC population ~~still~~ harbored the most DHMD, ~~and the~~whereas ~~CPA~~MpenA and ~~CPB~~MpenB ~~harbored the~~contained comparable ~~but significantly~~

fewer DHMD (Fig. 4C, Supplementary Fig. S10c-S10fFig. 4B and 4C, Supplementary Fig. S19c, S19d and 20a) than did the CPCMpenC population (Fig 4B and 4C, Supplementary Fig. S2419c and S19d, Supplementary Table S29). For Malayan pangolins, the MJ1MjavAB population harbored much more derived mutational loadLOFLoF than didcompared with that of the MJ2MjavBA population (Fig. 4A and 4B, Supplementary Table S1274 and S125 and S28). However, the proportion of DHMD in the MjavA population for the LOFLoF was comparable to that in the MjavB population (Fig. 4D), and.On the contrary, tThe MJ2MjavBA population has exhibited much a higher proportion of more DHMD for the dnsSNP and missense mutations than that of the MJ1MjavAB population for the dnsSNP and missense mutations (Supplementary Fig. S10f260b). However, the proportion of DHMD in the MJ2MjavB population for the LOF was comparable to that in the MJ1MjavA population (Fig. 4D, Supplementary Table S30),, possibly because of the more efficient genetic purging of large-effect strongly deleterious mutations (LOFLoF), which was not investigated in previous studies. In the Genomic Evolutionary Rate Profiling (GERP) analysis, we obtained a highly similar result with the CPCMpenC population harboring the most relative mutational load in among the MP populations and the MJ2MjavBA population harboring more relative mutational load than that in the MJ1MjavAB population (Fig. 4E and 4F).

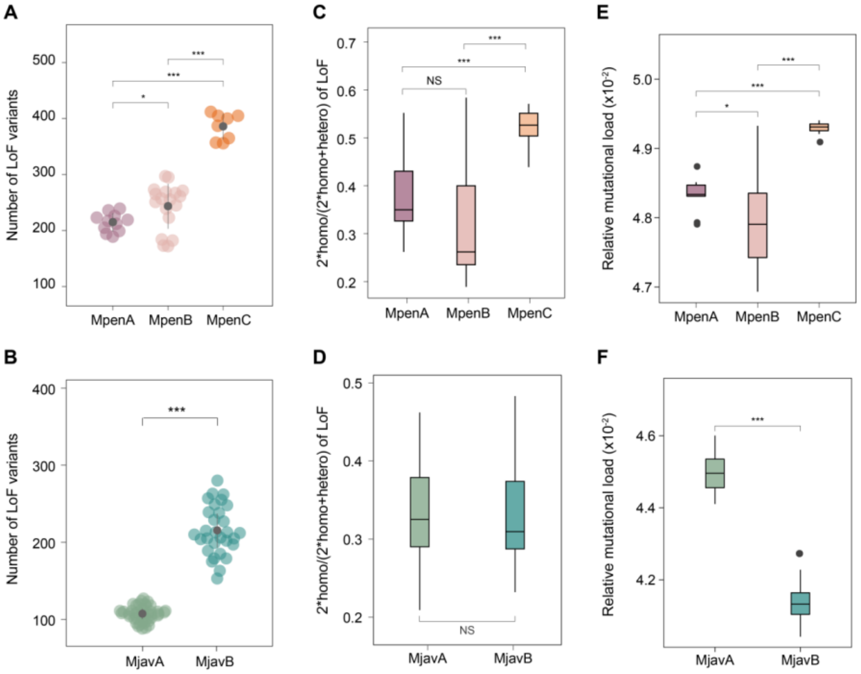

Figure 4: Mutational load in the Chinese and Malayan pangolin populations. (A) Total number of individual-level LOFLoF mutations across the fiveChinese pangolin populations. (B) Total number of individual-level LoF mutations across the Malayan pangolin populations. The number of homozygous LOF mutations at the individual-level across five pangolin populations. (C) The ratio of homozygous LOFLoF mutations in the Chinese pangolin populations was calculated as by the formula of : 2 × homozygous sites / (2 × homozygous sites + heterozygous site). (D) The ratio of homozygous LOFLoF mutations in the Malayan pangolin populations was calculated asusing the same formula calculated as that for the Chinese pangolin. (E) Relative mutational load in the Chinese pangolin populations (top 0.1% of GERP scores). (F) Relative mutational load in the Malayan pangolin populations (top 0.1% of GERP scores). The LOFLoF here means loss-of-function mutations.

SFS (site frequency spectrum) analysis ~~showed~~ revealed that 7.91% and 9.07% of putatively damaging and neutral alleles, respectively, were fixed in the ~~CPC~~MpenC population~~, and these~~. These two ratios were 6.84% and 9.53%, respectively, in the ~~MJ2~~Mjav~~B~~A population~~. However,~~; however, the proportion of the fixed alleles in the other three populations (~~CPA~~MpenA, ~~CPB~~MpenB and ~~MJ1~~Mjav~~A~~B) ~~were~~ was much smaller (Fig. 5A and 5B). This ~~indicated~~ indicates that the ~~CPC~~MpenC and ~~MJ2~~Mjav~~B~~A populations may have experienced population bottleneck events, and ~~the~~ genetic drift drove more rare alleles to fix into these two populations [7]. By comparing SFS lines between the Chinese and Malayan pangolins, we found that the SFS lines were flatter for polymorphic loci (fixed alleles excluded) in the Malayan pangolins, ~~while~~ whereas the flattest SFS line was observed in~~found for~~ the ~~MJ2~~Mjav~~B~~A population, indicating the possibility of more serious bottlenecks in the evolutionary history of the ~~MJ2~~Mjav~~B~~A population.

Genetic purging in pangolin populations

Genetic purging is an important ~~genetic factor~~process that ~~have~~ has an impact on the accumulation of deleterious mutations in ~~the~~ the population~~,~~ and it is usually more evident in small populations~~.~~; ~~butH~~however, ~~-~~few studies have ~~ever~~ discussed this issue ~~for~~ in pangolins. To determine~~Here, Tto check~~ whether ~~the~~ genetic purging has ~~been~~ occurred in these pangolin populations~~ are affected by the genetic purging as a consequences of~~facilitated by ~~the~~ inbreeding, we first compared the occurrence of mutational load (~~LOF~~LoF, missense mutation and dnsSNP) in the ROH regions (ROH*f*, the ratio of the number of mutational load to synonymous mutations in the ROH regions across the genome) and outside of the ROH regions (nonROH*f*, the ratio of the number of mutational load to synonymous mutations in the non-ROH regions across the genome)[10]. In the Malayan pangolin populations, the ROH*f* ~~of~~ for highly deleterious mutations (~~LOF~~LoF) was significantly lower than that of nonROH*f* in both the ~~MJ1~~MjavA and ~~MJ2~~MjavB population (Fig. 5C), but the difference (nonROH*f* / ROH*f*) was more pronounced in the ~~MJ1~~Mjav~~A~~B population than that in the ~~MJ2~~Mjav~~B~~A population (Fig. 5D). This denoted that many heterozygous deleterious mutations still existed in the genome[10, 11], which indicated that the genetic purging in the ~~MJ1~~MjavA and ~~MJ2~~MjavB populations ~~were both~~was less efficient, but stronger in the ~~MJ2~~Mjav~~B~~A population~~, which might~~. This may be promoted by the higher-level inbreeding in the ~~MJ2~~Mjav~~B~~A population. The dnsSNP and missense mutations ~~could~~ ~~were under~~showed~~reflect~~ the same situation (Supplementary Fig. ~~S~~S~~27 and S28~~11a-S11c). The relatively lower proportion of derived homozygous ~~LOF~~LoF in the ~~MJ2~~Mjav~~B~~A population also presented a decrease ~~of~~ in highly deleterious mutations that ~~might~~ may be caused by genetic purging (Fig. 4D, Supplementary Table S14). However, the Rxy analysis ~~detected the~~revealed an excess of mutational load in the ~~MJ2~~Mjav~~B~~A populations than that in the ~~MJ1~~Mjav~~A~~B populations (Supplementary Fig. S12a~~292~~), with the lowest degree of ~~this~~ excess found in the ~~LOF~~LoF~~, further supporting~~. This suggests that ~~the~~ genetic purging is limited, but tends to remove highly damaging mutations.

In the Chinese pangolin populations, the ROH*f* for ~~LOF~~LoF was lower than nonROH*f* in all three populations. The values of non-ROH*f* and ROH*f* in the ~~CPA~~MpenA population were ~~highly close~~very similar, but the non-ROH*f* was significantly higher ~~than~~ compared with that in the ROH*f* in both ~~of~~ the ~~CPB~~MpenB and ~~CPC~~MpenC populations (Fig. 5E and 5F~~, Supplementary Fig. S3023~~), indicating that the purging of ~~highly~~ deleterious alleles in the ~~CPA~~MpenA population was more efficient than that in the ~~CPB~~MpenB and ~~CPC~~MpenC populations for the LoF mutations. However, this was not obvious for the ~~relative~~relatively small-effect dnsSNP and missense mutations (Supplementary Fig. S11d-S11f). . The Rxy also ~~reflected~~ indicated that the ~~CPA~~MpenA population harbored the ~~least~~ fewest deleterious mutations, followed by the ~~CPB~~MpenB and the ~~CPC~~MpenC populations (Supplementary Fig. S12b~~2431~~). ~~However, we did not detect an obvious reduction in the derived homozygous mutational load (DHMD) in any of the three Chinese pangolin populations as we detected in the MJ2MjavB population, suggesting a less efficient genetic purging in the MP populations than in the MJ populations.~~ ~~Additionally~~In addition, the number of fixed damaging alleles was not significantly less than neutral alleles in all five populations (Fig. 5A and 5B),

further ~~implying~~ suggesting that the genetic purging in the pangolin populations is weak and not sufficient to clear a large number of deleterious mutations.

Formatted: English (United States)

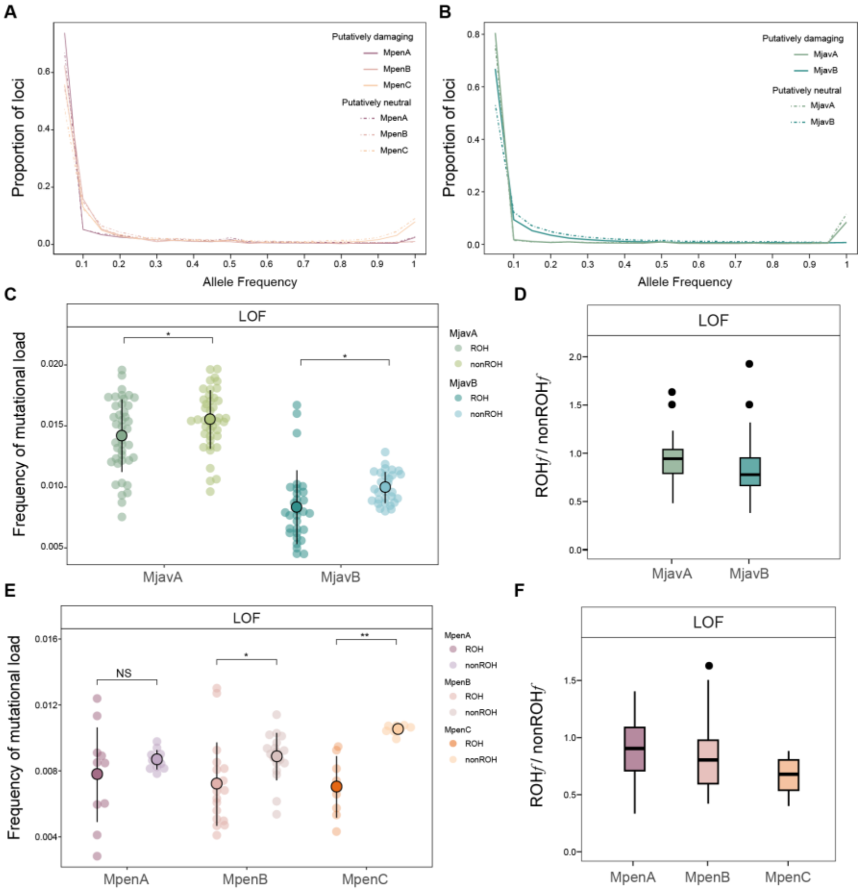

Figure 5: The SFS and genetic signals of genetic purging in pangolin populations. (A) SFS for putatively damaging (~~LOF~~LoF and missense mutations) and neutral mutations (intergenic variants) in the ~~CPA~~MpenA, ~~CPB~~MpenB, and ~~CPC~~MpenC populations. (B) SFS for putatively damaging and neutral mutations in the ~~MJ1~~MjavA and ~~MJ2~~MjavB populations. (C) Dot plot showing~~ed~~ the occurrence of ~~LOF~~LoF mutations in the two Malayan pangolin populations calculated as the ratio of the number of the mutational load to synonymous mutations in the ROH regions (ROH*f*) or nonROH (nonROH*f*) regions across the genome. (D) The ratio of ROH*f* to nonROH*f* for the LoF in the two Malayan pangolin populations. (E)~~(D)~~ Dot plot ~~showed~~ showing the occurrence of ~~LOF~~LoF mutations in the three Chinese pangolin populations calculated as that in (~~c~~C). ~~(E) The ratio of ROH*f* to nonROH*f* for the LOF in the two Malayan pangolin population.~~ (F) The ratio of ROH*f* to nonROH*f* for the ~~LOF~~LoF in the three Chinese pangolin populations.

Discussion

~~**The first haplotype-resolved chromosome-level and chromosome-scale pangolin genome**~~

~~Sixteen pangolin genomes representing all eight species have been reported to date, including three genomes~~

assembled from ONT long reads (one Chinese pangolin genome[31], one Malayan pangolin genome[31], and one giant pangolin genome[30]) and 13 short-read assembled genomes[8, 16, 28, 29]. For the Chinese pangolin, two short-read generated genomes [8, 16] and one ONT long-read assembled genome have been reported[31]. The Chinese pangolin genome assembled in this study exhibits significantly improved contiguity, being 1,939.53-fold, 301.36-fold, and 2.89-fold longer than the contig N50 of the M_pentadactyla-1.1.1, YNU_ManPten_2.0, and ASM2424420v1 genomes, respectively.The Chinese pangolin genome we assembled in this study has much better contiguity, which is 1,939.53-fold, 301.36-fold and 2.89-fold longer than the M_pentadactyla-1.1.1, YNU_ManPten_2.0 and ASM2424420v1 genomes, respectively (Supplementary Table S3126). There are also three reported genome assemblies for the Malayan pangolin, including two short-read generated genomes [8, 16] and one ONT long-read assembled genome[31]. The contiguity of the Malayan pangolin genome in this study was even higher than the Chinese pangolin genome we assembled, which is 3,740.34-fold, 829.79-fold and 3.87-fold greater than the ManJav1.0, YNU_ManJav_2.0 and ASM2460508v1 genomes, respectively (Supplementary Table S3226). The contig number of the two pangolin genomes (Chinese pangolin: 294; Malayan pangolin: 154) we assembled were much fewer than the ONT long-reads assembled genomes (Chinese pangolin: 6,002; Malayan pangolin: 2,568). Another advantage of our assemblies in this study is the partitioning of the diploid chromosomes into haploid chromosomes, which allowed us to detect genetic differences between haplotigs and provide a better understanding of allele-specific functions[42, 43], which cannot be achieved well with a hybrid reference genome. These advancements are anticipated to be further developed and enhance the precise conservation efforts for pangolins.

HiFi genomes improve the evaluation of genetic diversity and inbreeding

Accurate and precise evaluation of genome-wide extinction risks by measuring a series of genetic parameters is the central issue in conservation genomics and highly primarily depends on the quality of the reference genome [1]. However, what genetic parameters could can be improved the most by with a better reference genome? Here, we showed that the two genetic parameters most promoted by a higher-quality reference genome were are genetic diversity (π) and inbreeding (ROH) (Fig. 3A-3C). We could detected more variants across the genome based on the LG than the SG, because long reads could 1) span much more complex genomic regions[1] and 2) generate much longer contigs than short reads[44, 45]. Many genomic regions that cannot be assembled from by short reads can be assembled from by long reads, and these regions may contain important variants. AdditionallyIn addition, longer contigs facilitate a higher number of reads aligning that align accurately to with the reference genome. Both of these aspects couldmay contribute to enhancing the accuracy of genetic diversity calculations. Therefore, we don't do not recommend to comparecomparing the genetic diversity calculated based on the SG to that calculated based on the LG. However; however, it may still make sense to compare the genetic diversity that were bothwas calculated based on the SG genomes between different populations.

not make sense to compare genetic diversity between different populations based on SG genomes with similar contiguity. The estimation of inbreeding by detecting ROH across the genome highly depends on the genome contiguity, because short contigs in the SG cannot can hardly span over long ROH fragments. As anticipatedpredicted, the inbreeding level detected using the LG were significantly higher than those identified using the SG for ROH fragments larger than 1Mb. When we focused on ROHs larger than 100kb, this difference in F_{ROH} was still significant in the Malayan pangolin population but not in the Chinese pangolin population (Fig 3B and 3C), which we inferred should have been resulted from the different contiguities of the two pangolin genomes. Indeed, the contig N50 of the SG for the Chinese pangolin (133.77 kb) was substantially much longer than that of the SG for the Malayan pangolin (73.8 kb), allowing for the detection of ROH longer than 100 kb. In contrast, the scaffold N50 of the Malayan pangolin genome was longer than that of the Chinese pangolin genome, suggesting that contiguity contributed contributes more than scaffold contiguity to the detection of long ROH across the genome.

Genome-wide extinction risks in different pangolin populations

Although the Chinese and Malayan pangolin are listed as the Critically Endangered species by the IUCN Red List, the genome-wide genetic diversity of these two species is moderate and even higher than other endangered flagship species[8, 46], such as the tiger [44], giant panda[47], golden snub-nosed monkey[48], and kākāpo[11]. In this study, we discovered found an even higher genetic diversity than the previous reports[8, 46] in for the two pangolin species (Supplementary Fig. S322513, Supplementary Table S332716). Although the pangolin populations have been declining for a long time, we inferred that the recent population decline due tocaused by recentpoaching and illegal trade should beis population decline caused by poaching and illegal trade maybe more serious than ever before, leading to, which has resulted in a very rapid decliningе in much smaller and more isolated pangolin population sizes. Therefore, the genetic drift and genetic drift and inbreeding may not have not yet a timely respond to lead resulted in a substantial decrease in genetic diversityserious effect of reducing the genetic diversity. As expected, even thoughAlthough the high-quality HiFi genome improved the estimation of inbreeding, the F_{ROH} in both species was still lower than many other endangered species[7, 44], indicating a relatively fine intrinsic genetic background for these pangolin populations.

However, we foundWe observed a much faster and sharper population decline for the MjavA population within the most recent 10,000 years, when compared with the other four pangolin populations (Supplementary Fig. S713), indicating that this Malayan pangolin population may face morehas a serious extinction risks. The LG-‑based population genomic analysis also foundrevealed that both the genetic diversity ($\pi_{MJ2MjavBA}$=0.0007) and inbreeding ($F_{ROH>100kb}$=0.55) in the MJ2MjavBA Malayan population were much worse than those in other pangolin populations,population as which may be caused by the isolation and limited gene flow with other populations, because the MJ2MjavB A population is distributed across Southeast Asia [8] and the gene flow is easily separated by islands. In addition, we also found a much faster and sharper population decline for the MJ2MjavB population within the most recent 10,000 years, when compared with the other four pangolin populations (Supplementary Fig. S173), indicating that the MJ2MjavB population may facing more extinction risks. However, we cannot precisely locate this population because of the lack of accurate sampling locations [8], leaving a question forwhich should be the subject of future conservation work. Another thing worth paying attention toaspect to consider is that the Taiwan Chinese pangolin individual presentedexhibited a very high level of inbreeding, with the ROH fragments longer than 1Mb accounting for only ~0.4%, much less than athe 8.04% reported byin a previous study[8],. Thiswhich may be attributed to the harsh filtration of SNPs in the previous study and overestimated the overestimation of ROH longer than 1Mb. Two possibilities may explain for this phenomenon: 1) the Chinese population in Taiwan Province has ever been extensively inbred, but this situation has gradually improved, and the long ROH fragments hashave been broken by recombination over generations; and 2) this was a descendant of some highly inbred individuals translocated by humans from mainland China to the Taiwan Province, and the. The repeated mating with the native Taiwan pangolin population has broken the long ROH segments into small fragments. However, we do notcannot rule out that the Taiwan population may still face serious survival risks, and more Taiwan Chinese pangolin individuals are neededneed to be added intoto the analysis to help draw a clear conclusion.

The derived mutational load in all five pangolin populations were was greater than Amur tiger and South China tiger populations, even with their lower inbreeding levels[44]. For the Chinese pangolin, the CPCMpenC population had the highest proportion of mutational load, and we speculated . We hypothesized that the CPCMpenC population represents an ancient and isolated Chinese pangolin population in Yunnan Province and is less disturbed by human activity, but has accumulated a large number of mutational load over its evolutionary history,. We because we detected a stronger drift in the CPCMpenC population, which can cause a reduced efficacy of purifying selection to remove deleterious mutations[11]. For In the Malayan pangolin, the MJ2MjavBA population showed low genetic diversity and high inbreeding. Although the high inbreeding in the MJ2MjavBA population may promote the purging

of deleterious mutations, we still ~~found~~ observed a large amount of DHMD in ~~the~~ this population. ~~Associated with the fact of a~~The significantly lower frequency of ~~LOF~~LoF inside ~~ROH than~~ compare with outside the ROH regions~~,~~ indicates~~ing~~ that many strongly recessive deleterious mutations ~~still harbored~~remain in the nonROH regions in a ~~the~~ heterozygous state and ~~had~~have not been effectively removed~~, , which~~. This may be explained by the less efficient genetic purging ~~of deleterious mutations, particularly for less damaging alleles~~ in the pangolin populations. ~~Recently, another study led by *Wei et al.* has investigated the genetic structure and population status of the Chinese pangolin. This research was published while our study was under review.~~

Formatted: Font: Italic

Novel implications for the global conservation of ~~these~~ the two pangolin species

High-volume poaching and trafficking have resulted in the overexploitation of pangolins~~, and~~; thus, the wild population, particularly the Chinese and Malayan pangolins, has plummeted to ~~the edge of~~near extinction[8, 19-22]. Although moderate inbreeding and genetic diversity for these two species indicate~~d~~ a fine intrinsic genetic status, the population differentiation (F_{ST}) among ~~both~~ the Chinese and Malayan pangolin populations ~~are~~ remains large, which was also reported in other studies [8, 46, 49], even larger than ~~many subspecies level~~the genetic differences of many subsoecies [50-52]~~, and~~. G~~g~~enome-wide risks in different populations of Chinese and Malayan pangolins are also different[8, 46]. Therefore, in the ~~pursuit~~ effort of protecting and conserving pangolins, addressing, and managing the issues of illicit poaching and trafficking are just as ~~crucial~~ important as genetic rescue efforts. Implementing timely protective and conservation measures for both the Chinese and Malayan pangolins will ~~additionally~~ contribute to facilitating genetic rescue initiatives. Notably, both the genetic diversity and inbreeding ~~in~~ of the ~~MJ2~~Mjav~~B~~A population and the Taiwan individual are much worse than those in other pangolin populations, suggesting that the ~~MJ2~~Mjav~~B~~A population, as well as the Taiwan Chinese pangolins may suffer more serious survival pressures than other pangolin populations, and should receive more attention and protection~~ measures~~.

Materials and Methods

Samples, Re-sequencing Data and Ethics Statements

The Chinese ~~pangolin~~ and Malayan pangolins used for genome assembly were wild ~~rescued~~ individuals rescued by the Guangdong Wildlife Rescue Center. During a routine examination~~s~~, 5 mL of blood was collected with an anticoagulant tube~~ and~~, immediately transferred to liquid nitrogen, and stored at -80°C. The sample collection, experiment, and research design were all approved by the Institutional Review Board of BGI (BGI-IRB E22017). We strictly adhered to the guidelines provided by the BGI-IRB for all procedures~~ conducted in this study~~. The whole-genome sequencing data of 37 Chinese pangolin and 72 Malayan pangolin individuals were downloaded from the National Center for Biotechnology Information (NCBI) and the China National GeneBank DataBase (CNGBdb) for population genomic analysis in this study[8, 16, 46].

Nucleic Acid Extraction, Library Preparation, and Sequencing

Total genomic DNA was extracted using ~~a~~ the DNeasy Blood & Tissue Kit (Qiagen, USA) for whole genome sequencing~~e~~ (WGS) library preparation. Total RNA was extracted from blood ~~with~~ using Trizol reagent (Invitrogen, USA) from blood, and 250-300 bp reverse transcribed cDNA fragments were used for DNA library construction. Two Hi-C libraries were prepared with *Dpn*II restriction endonuclease. DNA libraries were subjected to the Illumina HiSeq X Ten platform at Novogene (Tianjin, China) for paired-end sequencing. For high-molecular-weight genomic DNA, the isolation was performed using the sodium dodecyl sulfate (SDS)-based method, and ~~the~~ purification was

carried out by the Qiagen Genomic Kit. A 15k library was constructed using high-quality DNA samples (main band > 30kb) and sequenced with the PacBio Sequel II platform at Novogene (Tianjin, China).

Genome Assembly and Assessment

To estimate the genome size, a total of ~100 Gb WGS short reads were used for analysis by the kmerfreq method[53] (v5.0). The hifiasm [26] (v0.16.1) software was used to generate the p~~P~~rimary genome~~s were assembled using hifiasm~~[26] ~~(v0.16.1)~~ with PacBio HiFi ~~-~~and Hi-C sequencing data. Hifiasm utilized~~employed~~ Hi-C sequencing data to achieve chromosome-level phasing in a method that does not require parental data[27]. This process ~~could~~can also phase the primary contigs at the same time into two sets of haplotigs, representing the two haploid genomes of ~~the~~a diploid genome. G~~-G~~enome redundancy was removed by the software ~~of~~ Purge_dups[54] (v1.2.5). Then, the Hi-C sequencing reads ~~are~~ were ~~again~~ re-mapped to the primary genomes, ~~-~~after quality control by the Juicer[55] (v1.5)~~, by~~and the *mem* algorithm of Burrows-Wheeler Aligner (BWA, v0.7.17) [56, 57]~~, while Hi-C data quality control was conducted by the Juicer~~[55] ~~(v1.5)~~. The 3d-DNA pipeline (v190716) was finally used to concatenate and review the primary scaffolds to chromosome-scale genomes[58]. We ~~have~~ identified the X chromosome and Y-linked regions using SRY genes and WGS short reads~~, confirming~~and confirmed that the sequencing depth of the sex chromosomes ~~is~~was approximately half that of the autosomes.

The genome completeness was evaluated by BUSCO ~~(Benchmarking Universal Single-Copy Orthologs)~~ (v5.2.2) software ~~with~~ using the vertebrata_odb10 data set [59]. We ~~carried out~~conducted a Merqury[60] (release 20200430) k-mer analysis and alignment of the whole genome sequencing reads to the reference genome to evaluate the accuracy of the genome assembly. Genome regions covered by PacBio long-read greater than 10-fold were considered ~~as~~ accurately assembled regions[33]. The identification of syntenic blocks between pangolin genomes was primarily performed by the NUCmer program in MUMmer[61] (v4.0.0rc1), followed by filtration using the delta-filter program in MUMmer (v4.0.0rc1) with parameters "-i 90 -l 5000".

Genome Annotation.

Repeat elements in the genome were annotated using *de novo* and homology-based methods. *De novo* repeats were first annotated using the LTR finder[62] (v1.0.6) and RepeatModeler2[63] (v2.0.1), and the identified repeats were then merged into the RepBase library as known elements. Transposable elements were identified and classified ~~by~~ using RepeatMasker (v4.0.5) with a conserved BLASTN search against the RepBase library [64]. The RepeatProteinMask program in ~~the~~ RepeatMasker (v4.0.5) was used to identify repeat proteins [64]. The ~~T~~tandem repeats were annotated using Tandem Repeats Finder[65] (v4.07).

Protein-coding genes were annotated using *de novo*, homology-based, and transcript-based approaches after masking the repeat elements. For the *de novo* method, we used ~~the~~ Augustus[66] (v3.0.3), GlimmerHMM[67] (v3.0.1), and SNAP[68] (v11/29/2013) to predict the gene models. For ~~the~~ transcript-based prediction, the transcripts were mapped to the reference genome using ~~the~~ HISAT2[69] (v2.1.0) and then assembled using StringTie[70] (v1.3.3b) based on clean RNA-seq data. Homology-based gene annotation was performed by using Blastall[71] (v2.2.26) with an E-value cut-off of 1e-5 to ~~align~~with against the protein sequences of *Homo sapiens*, *Mus musculus*, *Canis lupus familiaris*, and *Felis catus*. The final protein-coding gene set was generated using the MAKER[72] (v3.01.03) pipeline by combining high-quality homology-based, *de novo* and RNA-seq supported genes.

Functional annotation was performed by ~~using~~ a BLAST (v2.13.0) search against the SwissProt, TrEMBL, and Kyoto Encyclopedia of Genes and Genomes (KEGG) databases with an E-value cut-off of 1e-5. InterProScan[73] (v5.52-86.0) was used to predict motifs, domains, and Gene Ontology (GO) terms. The tRNA genes were identified using tRNAscan-SE[74] (v1.3.1), whereas the snRNA and miRNA genes were detected by searching the reference sequences against the ~~content of the~~ Rfam database (Release 12.0) using the BLAST (v2.13.0) and the program

cmsearch from infernal (v1.1.1) software.

Detection of Structural Variants (SVs) in the Pangolin Genome

To identify sequence differences between the parental genomes, sequence alignment was performed using Mummer (v4.0.0rc1) with the parameters 'nucmer --maxmatch -c 500 -b 500 -l 100' [61]. SVs were detected based on the alignment results using SyRi[75] (v1.3). To verify the accuracy of the detected SVs, we aligned the PacBio long reads to the reference genome using BLAST (v2.13.0) to determine whether the reads ~~could cross~~crossed the breakpoints. Moreover, we extracted 300 bp of upstream/downstream flanking sequences ~~of~~ for each breakpoint and manually verified them using DNBSEQ short reads by IGV[76] (v2.13.3) software. To identify gene loss in the haploid genome, we screened for pseudogenes interrupted by SVs using Mummer alignment and checked whether these genes had other copies across the entire genome.

Genome-wide Variant Calling and Quality Control

The BWA *mem* algorithm (v0.7.17) [56] was applied to map the whole-genome resequencing data of 72 Malayan pangolins and 37 Chinese pangolins to each of their reference genomes with default parameters. Sentieon[77] (v202010.01) was then used to sort, reorder, and deduplicate the alignment files for variant calling. Variants were detected for each individual using the Sentieon DNAseq Haplotyper pipeline, which is similar to the Genome Analysis Toolkit (GATK) HaplotypeCaller pipeline. Joint variant calling was ~~conducted~~ performed using the Sentieon DNAseq GVCFtyper with all gVCF files to generate a VCF file. To prepare for downstream analysis, the variant set was filtered to remove InDels and multiallelic~~multi-allelic~~ variants. For variant quality control, a stringent filtering step was performed ~~with~~ using the following parameters: "QD <2.0 || FS > 60.0 || MQ < 40.0 || MQRankSum < -12.5 || ReadPosRankSum < -8.0". Additionally, we filtered SNPs that were missed in more than 20% of the individuals in a population. ~~In this part, w~~We used both the short-read assembled genome (SG) and long-read assembled genome (LG) as references to generate variant sets for downstream comparison.

Population Structure Analysis

Before we performed ~~principal component analysis(~~PCA~~)~~, the VCF file was converted to PLINK format using PLINK software[78] (v1.90b6.10~~), then the genome~~. Genome-wide complex trait analysis (GCTA)[79] (v1.92.2) software was used for PCA analysis ~~with~~ using the default parameters. To construct a phylogenetic tree, vcf2phylip[80] (v2.7) was used to convert the VCF file into PHYLIP format. T~~hen, t~~he best substitution model was then calculated using jModelTest[81] (v2.1.10), and the maximum likelihood phylogenetic tree was constructed using ~~the~~ IQ-TREE[82] (v1.6.12) software with default parameters. ADMIXTURE[83] (v1.3.0) was used to determine the ancestry proportion with a specified number of clusters (K) ranging from 1 to 10. For~~In~~ this analysis, we used both the SG and LG as the reference genome to generate two groups of results.

Population Demography Inference

SMC++[84] (v1.5.1) was used to infer the historical changes ~~of~~ in the effective population size of ~~different~~ the varouus pangolin populations. The SMC++ results were visualized by scaling the time to real years using a generation time of one year and a mutation rate of $\mu = 1.47 \times 10^{-8}$[8, 16] for both the Malayan and Chinese pangolins. ~~Meanwhile, we used~~ MSMC2 [85] (v2.1.1) was used to infer the changes in the effective population size over the evolutionary history with four randomly selected individuals from each pangolin population. SNPs were first phased by Beagle[86] (v5.1) and then subjected to MSMC2 for inference of the population history. We used ~~both~~ the SG and LG as the reference genomes to generate two groups of results for comparison.

ROH and Genetic Diversity

To detect ROH fragments, multi-individual VCF files were converted into PLINK bfile format using the PLINK[78] (v1.90b6.10) software. The ROH was then detected using the PLINK[78] (v1.90b6.10) software with the parameters "--homozyg --homozyg-window-snp 20 --homozyg-kb 10 --homozyg-density 50" [87]. ROHs shorter than 100 kb were excluded from the downstream analysis. F_{ROH} was calculated as $F_{ROH} = L_{ROH}/L_{AUTOSOME}$, where L_{ROH} represents the total length of ROHs in each genome and $L_{AUTOSOME}$ represents the total length of the autosomes. Genome-wide genetic diversity (π) was calculated using vcftools[88] (v0.1.16) ~~with~~ using the parameters "vcftools --gzvcf vcf.gz --window-pi 500000 -out result". Genome-wide heterozygosity was calculated using vcftools[88] (v0.1.16) with "vcftools --gzvcf vcf.gz --het --out result" parameters. For ~~both~~ ROH and genetic diversity analysis, we used both the SG and LG as reference genomes for comparison, but only the results calculated based on the LG were used for further discussion.

Mutational Load and Genetic Purging Analysis

To identify the mutational load in the protein-coding genes, the variants were first annotated using ANNOVAR[89] (v20191024) and SnpEff[90] (v.5.0e). Variants annotated as stop gained, splice acceptor variant, or splice donor by SnpEff[90] were predicted to be loss-of-function (~~LOF~~LoF) mutations. Nonsynonymous variants with ~~⩾150~~ Grantham Score ⩾150 were considered ~~as~~ deleterious mutations (dnsSNP) [91]. To determine the derived allele, the Malayan pangolin genome was split into 100 bp reads and mapped to the Chinese pangolin genome. If an allele was found within the Malayan pangolin genome and simultaneously represented the predominant allele (with an allele frequency exceeding 50%) within the Chinese pangolin population, ~~we~~ it was designated ~~the allele~~ as the ancestral state within the Chinese pangolin genome[92]. The same approach was used to determine the ancestral state of variants of the Malayan pangolin.

The occurrence of the mutational load in the ROH and non-ROH regions (${}_{ROH}f$ and ${}_{nonROH}f$) for each individual genome was calculated by dividing the total number of deleterious mutations (N_m) within the ROH or non-ROH region by the number of synonymous mutations in the same region (S_{ROH} and S_{nonROH}) as follows:

$$ {}_{ROH}f = \frac{N_m}{S_{ROH}} $$

$$ {}_{nonROH}f = \frac{N_m}{S_{nonROH}} $$

To estimate the relative excess of deleterious mutations in one pangolin population compare ~~to~~ with another ~~population~~, we performed the Rxy analysis for dnsSNP, missense mutations, ~~LOF~~LoF, and synonymous mutations between pairs of pangolin populations [10]. We calculated the Rxy value ~~as~~ using the following formula:

$$ L_X = \frac{\sum_{i\in C}(m_X^i/s_X^i)(1-m_Y^i/s_Y^i)}{\sum_{i\in I}(m_X^i/s_X^i)(1-m_Y^i/s_Y^i)} $$

$$ R_{X/Y} = L_X/L_Y $$

~~The~~ Where m_X^i ~~represented~~ represents the count of derived alleles for the above-mentioned mutations observed at each site (i) within one population (X)~~; the~~ and m_Y^i ~~represented~~ represents that in another population (Y). s_X^i and s_Y^i represent~~ed~~ the total number of alleles at each site (i) of the population (X or Y). C ~~represented~~ represents the above-mentioned category of protein-coding sites, ~~while~~ whereas I ~~denoted~~ denotes the intergenic sites. ~~Here, we employed~~We used the jackknife method during the calculation ~~and~~ to obtain a standard error measurement. If Rxy = 1, both populations have the same level of derived mutation load, whereas if Rxy < 1, then population Y has more derived load than X and vice versa if Rxy > 1.

Genomic Evolutionary Rate Profiling (GERP) Scores

It is difficult to estimate the genetic load without fitness data~~, here~~. Therefore, we calculated the relative mutational load ~~in~~ for each individual genome. First, we screened the derived alleles distributed in the highly conserved genome region of ~~these~~ the two pangolin species ~~by~~ using the genomic evolutionary rate profiling scores (GERP) method. To calculate~~d~~ the GERP scores, we selected the genomes of 37 species (*Acinonyx jubatus*, *Bos taurus*, *Callithrix jacchus*, *Canis lupus*, *Cavia porcellus*, *Choloepus hoffmanni*, *Dasypus novemcinctus*, *Dipodomys ordii*, *Echinops telfairi*, *Equus caballus*, *Erinaceus europaeus*, *Felis catus*, *Homo sapiens*, *Loxodonta africana*, *Lynx canadensis*, *Mus musculus*, *Myotis lucifugus*, *Ochotona princeps*, *Oryctolagus cuniculus*, *Panthera pardus orientalis*, *Panthera tigris*, *Pan troglodytes*, *Prionailurus bengalensis*, *Procavia capensis*, *Pteropus vampyrus*, *Puma concolor*, *Rattus norvegicus*, *Sorex Araneus*, *Spermophilus tridecemlineatus*, *Tupaia belangeri*, *Tursiops truncates*, *Vicugna pacos*, *Manis javanica*, *Manis pentadactyla*, *Tamandua tetradactyla*, *Ovis aries*, and *Vulpes lagopus*) for screening ultra-conserved ~~genome~~ genomic regions. We split these genomes into 100 bp reads to generate fastq files. Then, we respectively aligned these fastq files to the Malayan ~~pangolin~~ and Chinese pangolin genomes using the *mem* algorithm in BWA (v0.7.17-r1188) with "-B 3" parameter. GERP scores were then calculated ~~by~~ using the gerpcol program ~~gerpcol~~ from the GERP++[93] software (http://mendel.stanford.edu/sidowlab/downloads/gerp/index.html) based on the above-mentioned alignment files. In general, low GERP scores (<1) usually represent putatively neutral genome regions, ~~but~~ whereas high GERP scores (>1) indicate conserved genome regions [11]. Derived alleles in more conserved genome regions (those with higher GERP scores), these alleles are likely to be more deleterious. In this study, we calculated the relative mutational load with mutations having the top 0.1% GERP scores to select more deleterious alleles distributed in ~~the~~ highly conserved genome regions [7]. ~~, and the~~ The relative mutational load was calculated by the following formula: the sum of all homozygous and heterozygous derived alleles multiplied by their conservation score over the total number of derived alleles, with the heterozygous counted as one allele and homozygous sites counted as two alleles [11]. Therefore, a higher relative mutation load indicates that a relatively larger proportion of derived alleles ~~could~~ may be found ~~at~~ in more conserved genomic regions.

Site-Frequency Spectrum (SFS) Analysis

For SFS analysis, we calculated the frequency of each type of mutations at every site in ~~different~~ the various pangolin populations. We considered intergenic variants as neutral, ~~while~~ whereas ~~LOF~~LoF and missense variants ~~as~~ were consider putatively damaging mutations[7]. For SFS in each pangolin population, we subsampled nonmissing derived alleles from each locus for calculation[7]. Fixed (frequency=1) and missing (frequency=0) alleles were included in the SFS for the five populations. We used LG as the reference genome for SFS analysis.

Additional Files

Supplementary Fig. S1. The heatmap represents the contact matrices generated by aligning the Hi-C data to the haploid chromosome-level Chinese pangolin (a) and Malayan pangolin (b) genomes.

Supplementary Fig. S2. Estimated genome size of the Chinese and Malayan pangolin genomes by using K-mer frequency analysis with k-mer size of 17. (a) The K-mer spectra of the Chinese pangolin genome. (b) The K-mer spectra of the Malayan pangolin genome.

Supplementary Fig. S3. Sequencing depths of each pseudo-chromosome. (a) Sequencing depths of the 19 autosomes, X chromosome, and Y chromosome in the Chinese pangolin genome. (b) Sequencing depths of the 18 autosomes, X chromosome, and Y chromosome in the Malayan pangolin genome.

Supplementary Fig. S4. Comparisons of LG and SG on PCA analysis for both Chinese and Malayan pangolin

populations. (a) PCA analysis of Malayan pangolin populations based on SG. (b) PCA analysis of Malayan pangolin populations based on LG. (c) PCA analysis of Chinese pangolin populations based on SG. (d) PCA analysis of Chinese pangolin populations based on LG.

Supplementary Fig. S5. Comparisons of LG and SG on the construction of phylogenetic tree for both Chinese and Malayan pangolin populations. (a) Phylogenetic tree constructed based on the SG for Malayan pangolin populations. (b) Phylogenetic tree constructed based on the LG for Malayan pangolin populations. (c) Phylogenetic tree constructed based on the SG for Chinese pangolin populations. (d) Phylogenetic tree constructed based on the LG for Chinese pangolin populations.

Supplementary Fig. S6. Comparisons of LG and SG on the admixture analysis for both Chinese and Malayan pangolin populations. (a) Genome-wide admixture analysis for three populations of Chinese pangolin based on the SG. (b) Genome-wide admixture analysis for three populations of Chinese pangolin based on the LG. (c) Genome-wide admixture analysis for two populations of Malayan pangolin based on the SG. (d) Genome-wide admixture analysis for two populations of Malayan pangolin based on the LG.

Supplementary Fig. S7. Comparison of LG and SG assembly for analyzing population history and separation in Chinese and Malayan pangolin populations. (a) The dynamics of effective population size of Malayan pangolin populations analyzed based on the SG. (b) The population size dynamics of Malayan pangolin populations analyzed based on the LG. (c) The population size dynamics of Chinese pangolin populations analyzed based on the SG. (d) The population size dynamics of Chinese pangolin populations analyzed based on the LG. (e) The divergence time between two populations of Malayan pangolin estimated based on the SG. (f) The divergence time between two populations of Malayan pangolin estimated based on the LG. (g) The divergence time among three populations of Chinese pangolin estimated based on the SG. (h) The divergence time among three populations of Chinese pangolin estimated based on the LG.

Supplementary Fig. S8. (a) The population-level ROH distribution in three Chinese pangolin populations. (b) The population-level ROH distribution in two Malayan pangolin populations.

Supplementary Fig. S9. (a)The individual-level distribution of ROH larger than 100Kb in Chinese pangolin genomes. (b) The individual-level distribution of ROH larger than 100Kb in Malayan pangolin genomes. Each row represents an individual.

Supplementary Fig. S10. Total deleterious nonsynonymous SNP (dnsSNP) (a) and missense (b) mutations at the individual level were assessed across five populations of Chinese and Malayan pangolins. The number of individual-level homozygous LoF(c), dnsSNP (d) and missense (e) mutations across the five pangolin populations. (f) The ratio of homozygous missense mutations and dnsSNPs in Chinese and Malayan pangolin populations was calculated as the formula of : 2 × homozygous sites / (2 × homozygous sites + heterozygous site).

Supplementary Fig. S11. Dot plot showed the occurrence of dnsSNPs (a) and missense mutations (b) in the two Malayan pangolin populations calculated as the ratio of the number of mutational load to synonymous mutations in the ROH regions (ROH*f*) or nonROH regions (nonROH*f*) across the genome. The ratio of ROH*f* to nonROH*f* for dnsSNP and missense mutations in Malayan pangolin populations (c).Dot plot showed the occurrence of dnsSNPs (d) and missense mutations (e) in the three Chinese pangolin populations calculated as the ratio of the number of mutational load to synonymous mutations in the ROH regions or nonROH regions across the genome.The ratio of ROH*f* to nonROH*f* for dnsSNP and missense mutations inChinese pangolin populations (f)

Supplementary Fig. S12. (a) The Rxy ratio of derive alleles in MjavA (x) to MjavB (y) for dnsSNP, synonymous, missense and LoF. The Rxy <1 indicated the population y has more derived alleles than population x. (b) The Rxy ratio of derive alleles in x population to y population (x/y: MpenB/MpenC; MpenA/MpenC; MpenA/MpenB) for dnsSNP, synonymous, missense and LoF. The Rxy <1 indicated the population y has more derived alleles than population x.

Supplementary Fig. S13. Comparison of genome-wide π of the Malayan pangolin and Chinese pangolin with other endangered species. Abbreviations along the X-axis are as follows: CMA: Brown eared pheasant (*Crossoptilon mantchuricum*), ASI: Chinese alligator (*Alligator sinensis*), PTA: Amur tiger (*Panthera tigris altaica*), AFU: Red panda (*Ailurus fulgens*), AME: Giant panda (*Ailuropoda melanoleuca*), MJ: Malayan pangolin (*Manis javanica*), MBE: Dwarf musk deer (*Moschus berezovskii*), MP: Chinese pangolin (*Manis pentadactyla*).

Supplementary Fig. S14. K-mer spectra plot estimated by Merqury. (a) K-mer spectra plot for the haplotype-resolved chromosome-level genome of MP. (b) K-mer spectra plot for the haplotype-resolved chromosome-level genome of MJ.

Supplementary Fig. S15. The sequencing depths of the two groups of haplotigs in both MJ and MP genomes. (a, b) Alignment with DNBSEQ read. (c, d) Alignment with PacBio HiFi reads.

Supplementary Fig. S16. Pairwise differences observed between the haploid genomes of Malayan and Chinese pangolin. The sliding window was set to be 100 bp.

Supplementary Fig. S17. (a) Brief introduction and circos diagram of the two pangolin genomes. (b) Structural rearrangements between the two haplotypes of each chromosome in Malayan pangolin and Chinese pangolin genomes. (c) Dot plot between MJH1 (x-axis) and MJH2 (y-axis), plotted by pafCoordsDotPlotly. (d) Dot plot between MPH2 (x-axis) and MPH1 (y-axis), plotted by pafCoordsDotPlotly.

Supplementary Fig. S18. Validation of structural variants through contig mapping. Contigs are mapped to both the haploid genomes to verify structural variants and visualized by the IGV software. (a) The correct structural variants between the haploid genomes. The breakpoints of the SVs could be covered by complete contigs in both of the haploid genomes. (b) The incorrect structural variants between the haploid genomes. The breakpoints of the SVs in one of the two haploid genomes could not be covered by the complete contig. In the IGV screenshot, the gray bar represents the contig spanning over the SVs, and the contig corresponding SV regions were marked as the red box. All structural variants have been verified, and several are randomly displayed here.

Supplementary Fig. S19. (a) The GO enrichment result of genes distributed in structural variants of Malayan pangolin. (b) The KEGG enrichment result of genes distributed in structural variants of Malayan pangolin. (c) The GO enrichment result of genes distributed in structural variants of Chinese pangolin. (d) The KEGG enrichment result of genes distributed in structural variants of Chinese pangolin.

Supplementary Table S1. Summarized sample information in this study.

Supplementary Table S2. Information of five populations in Chinese and Malayan pangolin included in this study.

Supplementary Table S3. Statistics of genome assemblies for the Chinese pangolin and Malayan pangolin.

Supplementary Table S4. The length of each chromosome in the Chinese pangolin and Malayan pangolin genomes.

Supplementary Table S5. The overall statistics of repeats in the Chinese and Malayan pangolin genomes.

Supplementary Table S6. Statistics of the annotated genes in Chinese and Malayan pangolin genomes.

Supplementary Table S7. BUSCO analysis of genome assemblies and gene sets in this study.

Supplementary Table S8. Statistics of functional annotation for the Malayan and Chinese pangolin's gene sets.

Supplementary Table S9. Statistics of ncRNA annotation.

Supplementary Table S10. SNPs number, genetic diversity (π), heterozygosity and SNP density calculated based

on LG and SG in Malayan and Chinese pangolin populations.

Supplementary Table S11. The comparison of the SG and LG for estimating ROH in Malayan pangolin and Chinese pangolin populations

Supplementary Table S12. The count and length of ROH fragments in the five populations of Malayan pangolin and Chinese pangolin.

Supplementary Table S13. The F_{ROH} in the five populations of Malayan pangolin and Chinese pangolin.

Supplementary Table S14. The total count of dnsSNPs, LoF, missense and synonymous SNPs in various populations, along with the average number of dnsSNPs, LoF, missense, and synonymous SNPs at the individual level, across different populations of the Malayan and Chinese pangolins.

Supplementary Table S15. The individual level mutational load estimates in all Chinese pangolin and Malayan pangolin populations.

Supplementary Table S16. Comparison of genome-wide nucleotide diversity (π) of the Malayan pangolin and Chinese pangolin with reference to other endangered species on the IUCN Red List.

Supplementary Table S17. Quality assessment of the MP and MJ genomes by the Merqury software.

Supplementary Table S18. The mapping rates of four types of sequencing data to genomes assembled in this study.

Supplementary Table S19. Comparison of the assembly statistics with the previously published Chinese pangolin and Malayan pangolin genomes.

Supplementary Table S20. Pairwise differences observed in comparisons between the haplotype genomes of Chinese and Malayan pangolin, the sliding window was set to be 100bp.

Supplementary Table S21. Chromosomal structural variants (>50bp) of MJH1 and MJH2, and of MPH1 and MPH2.

Supplementary Table S22. Genes distributed in the structural variants of MJ and MP genomes.

Supplementary Table S22. The KEGG enrichment result of genes distributed in the structural variants of MJ/MP.

Supplementary Table S23. The GO enrichment result of genes distributed in the structural variants of MJ/MP.

Supplementary Table S24. Functional description of pseudogenes interrupted by structural variants.

~~**Supplementary Fig. S1.** The heatmap represents the contact matrices generated by aligning the Hi-C data to the haplotype-resolved chromosome-level MJ and MP genomes. (A) The Hi-C map of the diploid Malayan pangolin genome. (B) The Hi-C map of the diploid Chinese pangolin genome.~~

~~**Supplementary Fig. S2.** Estimated genome size of the Chinese and Malayan pangolin genomes by using K-mer frequency analysis with k-mer size of 17. (A) The K-mer spectra of the Chinese pangolin genome. (B) The K-mer spectra of the Malayan pangolin genome.~~

~~**Supplementary Fig. S3.** Sequencing depths of each pseudo-chromosome. (A) Sequencing depths of the 19 autosomes, X chromosome (Hic_scaffold_8), and Y chromosome (Hic_scaffold_22) in the MP genome. (B) Sequencing depths of the 18 autosomes, X chromosome (Hic_scaffold_7), and Y chromosome (Hic_scaffold_20) in the MJ genome.~~

~~**Supplementary Fig. S4.** K-mer spectra plot estimated by Merqury. (A) K-mer spectra plot for the haplotype-resolved chromosome-level genome of MP. (B) K-mer spectra plot for the haplotype-resolved chromosome-level genome of MJ.~~

~~**Supplementary Fig. S5.** The sequencing depths of the two groups of haplotigs in both MJ and MP genomes. (a, b) Alignment with DNBSEQ read. (c, d) Alignment with PacBio HiFi reads.~~

~~**Figure S6.** (a) The chromosome-scale synteny analysis between the Malayan pangolin and Chinese pangolin genomes. (b) Dot plot between MJH1 (x-axis) and MJH2 (y-axis), plotted by pafCoordsDotPlotly. (c) Dot plot between MPH1 (x-axis) and MPH2 (y-axis), plotted by pafCoordsDotPlotly.~~

Supplementary Fig. S6. Pairwise differences observed between the haploid genomes of Malayan and Chinese pangolin. The sliding window was set to be 100 bp.

Supplementary Fig. S7. Pairwise differences observed between the haploid genomes of Malayan and Chinese pangolin. The sliding window was set to be 100 bp.Dot plot between MJH1 (x-axis) and MJH2 (y-axis), plotted by pafCoordsDotPlotly.

Supplementary Fig. S8. Dot plot between MPH1 (x-axis) and MPH2 (y-axis), plotted by pafCoordsDotPlotly.

Supplementary Fig. S98. Schematic diagram of the method we validated the structural variants across the genome.

Supplementary Fig. S109. (a) The GO enrichment result of genes distributed in structural variants of MJ. (b) The KEGG enrichment result of genes distributed in structural variants of MJ. (c) The GO enrichment result of genes distributed in structural variants of MP. (d) The KEGG enrichment result of genes distributed in structural variants of MP. The GO enrichment result of genes distributed in structural variants of MJ.

Supplementary Fig. S11. The KEGG enrichment result of genes distributed in structural variants of MJ.

Supplementary Fig. S12. The GO enrichment result of genes distributed in structural variants of MP.

Supplementary Fig. S13. The KEGG enrichment result of genes distributed in structural variants of MP.

Supplementary Fig. S140. Comparisons of LG and SG on PCA analysis for both Chinese and Malayan pangolin populations. (A) PCA analysis of Malayan pangolin populations based on SG. (B) PCA analysis of Malayan pangolin populations based on LG. C PCA analysis of Chinese pangolin populations based on SG. (D) PCA analysis of Chinese pangolin populations based on LG.

Supplementary Fig. S151. Comparisons of LG and SG on the construction of phylogenetic tree for both Chinese and Malayan pangolin populations. (A) Phylogenetic tree constructed based on the SG for Malayan pangolin populations. (B) Phylogenetic tree constructed based on the LG for Malayan pangolin populations. (C) Phylogenetic tree constructed based on the SG for Chinese pangolin populations. (D) Phylogenetic tree constructed based on the LG for Chinese pangolin populations.

Supplementary Fig. S162. Comparisons of LG and SG on the admixture analysis for both Chinese and Malayan pangolin populations. (A) Genome-wide admixture analysis for three populations of Chinese pangolin based on the SG. (B) Genome-wide admixture analysis for three populations of Chinese pangolin based on the LG. (C) Genome-wide admixture analysis for two populations of Malayan pangolin based on the SG. (D) Genome-wide admixture analysis for two populations of Malayan pangolin based on the LG.

Supplementary Fig. S173. Comparisons of LG and SG on the population history analysis for both Chinese and Malayan pangolin populations. (A) The dynamics of effective population size of Malayan pangolin populations analyzed based on the SG. (B) The population size dynamics of Malayan pangolin populations analyzed based on the LG. (C) The population size dynamics of Chinese pangolin populations analyzed based on the SG. (D) The population size dynamics of Chinese pangolin populations analyzed based on the LG.

Supplementary Fig. S184. Comparisons of LG and SG on the inference of population separation among Chinese and Malayan pangolin populations. (A) The divergence time between two populations of Malayan pangolin estimated based on the LG. (B) The divergence time between two populations of Malayan pangolin estimated based on the LG. (C) The divergence time among three populations of Chinese pangolin estimated based on the SG. (D) The divergence time among three populations of Chinese pangolin estimated based on the LG.

Supplementary Fig. S195. The population-level ROH distribution in three Chinese pangolin populations.

Supplementary Fig. S2016. The individual level distribution of ROH larger than 100Kb in Chinese pangolin genomes.

Supplementary Fig. S2117. The population-level ROH distribution in two Malayan pangolin populations.

Supplementary Fig. S2218. The individual level distribution of ROH larger than 100Kb in Malayan pangolin

genomes.

Supplementary Fig. S1923. Total deleterious nonsynonymous SNP (dnsSNP) (a) and missense (b) mutations at the individual level were assessed across five populations of Chinese and Malayan pangolins. The number of individual-level homozygous dnsSNP (c) and missense (d) mutations across the five pangolin populations.Total deleterious nonsynonymous SNP (dnsSNP) (A) and missense (B) mutations at the individual level were assessed across five populations of Chinese and Malayan pangolins.

Supplementary Fig. S240. The ratio of homozygous missense mutations and dnsSNPs in Chinese pangolin populations was calculated as the formula of : 2 × homozygous sites / (2 × homozygous sites + heterozygous site). (b) The ratio of homozygous missense mutations and dnsSNPs in Malayan pangolin populations was calculated as the formula of : 2 × homozygous sites / (2 × homozygous sites + heterozygous site).

The number of individual-level homozygous dnsSNP (A) and missense (B) mutations across the five pangolin populations.

Supplementary Fig. S251. Dot plot showed the occurrence of dnsSNPs (a) and missense mutations (b) in the two Malayan pangolin populations calculated as the ratio of the number of mutational load to synonymous mutations in the ROH regions or nonROH regions across the genome. The ratio of ROHf to nonROHf for dnsSNP and missense mutations in Chinses pangolin populations (c) and Malayan pangolin populations (d).The ratio of homozygous missense mutations and dnsSNPs in Chinese pangolin populations was calculated as the formula of : 2 × homozygous sites / (2 × homozygous sites + heterozygous site).

Supplementary Fig. S26. The ratio of homozygous missense mutations and dnsSNPs in Malayan pangolin populations was calculated as the formula of : 2 × homozygous sites / (2 × homozygous sites + heterozygous site).

Supplementary Fig. S27. Dot plot showed the occurrence of dnsSNPs (A) and missense mutations (B) in the two Malayan pangolin populations calculated as the ratio of the number of mutational load to synonymous mutations in the ROH regions or nonROH regions across the genome.

Supplementary Fig. S28. The ratio of ROH*f* to nonROH*f* for dnsSNP and missense mutations in Chinses pangolin populations (A) and Malayan pangolin populations (B).

Supplementary Fig. S292. The Rxy ratio of derive alleles in MJ2MjavB (x) to MJ1MjavA (y) for dnsSNP, synonymous, missense and LOF. The Rxy <1 indicated the population y has more derived alleles than population x.

Supplementary Fig. S3023. Dot plot showed the occurrence of dnsSNPs (A) and missense mutations (B) in the three Chinese pangolin populations calculated as the ratio of the number of mutational load to synonymous mutations in the ROH regions or nonROH regions across the genome.

Supplementary Fig. S3124. The Rxy ratio of derive alleles in x population to y population (x/y: CPBMpenB/CPCMpenC; CPAMpenA/CPCMpenC; CPAMpenA/CPBMpenB) for dnsSNP, synonymous, missense and LOF. The Rxy <1 indicated the population y has more derived alleles than population x.

Supplementary Fig. S3225. Comparison of genome-wide π of the Malayan pangolin and Chinese pangolin with other endangered species. Abbreviations along the X-axis are as follows: CMA: Brown eared pheasant (*Crossoptilon mantchuricum*), ASI: Chinese alligator (*Alligator sinensis*), PTA: Amur tiger (*Panthera tigris altaica*), AFU: Red panda (*Ailurus fulgens*), AME: Giant panda (*Ailuropoda melanoleuca*), MJ: Malayan pangolin (*Manis javanica*), MBE: Dwarf musk deer (*Moschus berezovskii*), MP: Chinese pangolin (*Manis pentadactyla*).

Supplementary Table S1. Summarized sample information in this study.

Supplementary Table S2. Information of five populations in Chinese and Malayan pangolin included in this study.

Supplementary Table S3. Statistics of genome assemblies for the Chinese pangolin and Malayan pangolin.

Supplementary Table S4. The length of each chromosome in the Chinese pangolin and Malayan pangolin genomes.

Formatted: Justified

Supplementary Table S5. The length of each chromosome in the Malayan pangolin genomes.

Supplementary Table S65. Quality assessment of the MP and MJ genomes by the Merqury software.

Supplementary Table S76. BUSCO analysis of genome assemblies in this study.

Supplementary Table S87. The mapping rates of four types of sequencing data to genomes assembled in this study.

Supplementary Table S98. The overall statistics of repeats in the Chinese and Malayan pangolin genomes.

Supplementary Table S10. Statistics of repeat elements identified by *de novo* method in Chinese and Malayan pangolin genomes.

Supplementary Table S119. Statistics of the annotated genes in Chinese and Malayan pangolin genomes.

Supplementary Table S102. BUSCO analysis of gene sets for Chinese and Malayan pangolin.

Supplementary Table S131. Statistics of functional annotation for the Malayan and Chinese pangolin's gene sets.

Supplementary Table S124. Statistics of ncRNA annotation.

Supplementary Table S153. Pairwise differences observed in comparisons between the haplotype chromosome-level genomes of Chinese and Malayan pangolin, the sliding window was set to be 100bp.

Supplementary Table S164. Chromosomal structural variants (>50bp) of MJH1 and MJH2, and of MPH1 and MPH2.

Supplementary Table S175. Genes distributed in the structural variants of MJ and MP genomes.

Supplementary Table S186. The KEGG enrichment result of genes distributed in the structural variants of MJ/MP.

Supplementary Table S197. The GO enrichment result of genes distributed in the structural variants of MJ/MP.

Supplementary Table S2018. Functional description of pseudogenes interrupted by structural variants.

Supplementary Table S2119. SNPs number, genetic diversity (*π*), heterozygosity and SNP density calculated based on LG and SG in Malayan and Chinese pangolin populations.

Supplementary Table S220. The comparison of the SG and LG for estimating ROH in Malayan pangolin and Chinese pangolin populations

Supplementary Table S231. The count and length of ROH fragments in the twofive populations of Malayan pangolin and Chinese pangolin.

Supplementary Table S24. The count and length of ROH fragments in the three populations of Chinese pangolin.

Supplementary Table S252. The F_{ROH} in the twofive populations of Malayan pangolin and Chinese pangolin.

Supplementary Table S26. The F_{ROH} in the three populations of Chinese pangolin.

Supplementary Table S273. The number of dnsSNP, LOF, missense mutations and synonymous SNPs in different populations of the Malayan and Chinese pangolin.

Supplementary Table S284. The number of dnsSNP, LOF, missense mutations and synonymous SNPs at individual level in different populations of the Malayan and Chinese pangolin.

Supplementary Table S295. The individual level geneticmutational load estimates in all Chinese pangolin and Malayan pangolin populations.

Supplementary Table S30. The individual level genetic load estimates in all Malayan pangolin populations.

Supplementary Table S3126. Comparison of the assembly statistics with the previously published Chinese pangolin and Malayan pangolin genomes.

Supplementary Table S32. Comparison of the assembly statistics with the previously published Malayan pangolin genomes.

Supplementary Table S2733. Comparison of genome wide nucleotide diversity (*π*) of the Malayan pangolin and Chinese pangolin with reference to other endangered species on the IUCN Red List.

Formatted: Justified

Data Availability

Bioproject and biosample for the genomic data of this study were submitted to NCBI under accession numbers PRJNA1114675. The accession number of genomes are GCA_040802235.1 and GCA_040802205.1. The data that support the findings in this study also have been deposited into CNGB Sequence Archive (CNSA) [94]of China National GeneBank DataBase (CNGBdb) [95] with accession number CNP0004630. The resequencing data in this study were retrieved from earlier studies (37 Chinese pangolins: CNP0001723, CNGBdb; PRJNA529540 and PRJNA20331, NCBI Read Archive. 72 Malayan pangolins: PRJNA529540, NCBI Read Archive) [8, 16, 46].

Abbreviations

WGS: Whole genome sequence; RNA-seq: RNA sequence; BUSCO: Benchmarking Universal Single-Copy Orthologs; HiFi: High fidelity; Hi-C: High-throughput/resolution chromosome conformation capture; PCA: Principal component analysis; SNP: Single-nucleotide polymorphism; TE: Transposable element; KEGG: Kyoto Encyclopedia of Genes and Genomes; GO: Gene Ontology; GATK: Genome Analysis Toolkit; SG: Short-read assembled genome; LG: Long-read assembled genome; ROH: Runs of homozygosity; ~~LOF~~LoF: Loss-of-function; dnsSNP: Deleterious nonsynonymous mutation; GERP: Genomic evolutionary rate profiling; SFS: Site-Frequency Spectrum.

Author Contributions

Tianming Lan, Huan Liu, Yinping Tian and Yan Hua conceived and initiated the project. Yan Hua, Jun Li, Fanghui Hou, Yue Ma,Tengcheng Que, Wenjian Liu and Kai Wang collected the samples. Jin Chen, Chuanling Yin and Yinping Tian performed DNA isolation, library preparation and genome sequencing. Haimeng Li, Minhui Shi, Boyang Liu and Qing Wang assembled the genomes and conducted the genomics analysis. Tianming Lan coordinated the genomic analysis. Tianming Lan and Haimeng Li wrote the manuscript. Sunil Kumar Sahu, Minhui Shi, Yanling Xia and Boyang Liu reviewed and edited the manuscript. Tianming Lan and Yan Hua made important contributions to the revision of the manuscript. All the authors read and approved the final manuscript.

Competing interests

The authors declare no competing interests.

Acknowledgments

This study was supported by the National Key Program of Research and Development, Ministry of Science and Technology (Grant No. 2022YFF1301500), the Guangdong Provincial Key Laboratory of Genome Read and Write

(Grant No. 2017B030301011) and the Start-up Scientific Foundation of Northeast Forestry University (60201524043). This work was also supported by China National GeneBank (CNGB). We thank Kinanti Seraphina Larasati and Shiqing Wang for their help in reviewing and polishing the language. We thank Hui Liu and Tian Xia from the Shenzhen Safari Park Co., Ltd. for assisting with sample collection. Finally, we thank all the researchers (Shiqing Wang, Dongyi Yang, Jieyao Yu, Jiale Fan, Yuting Huang, Yingna Zhou, Tianlu Liu, Jiatong Cheng, Chen Lin and Shiyu Liu) involved in sample collection, genome sequencing and analysis.

References

1. Formenti G, Theissinger K, Fernandes C, Bista I, Bombarely A, Bleidorn C, et al. The era of reference genomes in conservation genomics. Trends in ecology & evolution. 2022;37 3:197-202. doi:10.1016/j.tree.2021.11.008.
2. Kitts PA, Church DM, Thibaud-Nissen F, Choi J, Hem V, Sapojnikov V, et al. Assembly: a resource for assembled genomes at NCBI. Nucleic acids research. 2016;44 D1:D73-80. doi:10.1093/nar/gkv1226.
3. Ouborg NJ, Pertoldi C, Loeschcke V, Bijlsma RK and Hedrick PW. Conservation genetics in transition to conservation genomics. Trends in genetics : TIG. 2010;26 4:177-87. doi:10.1016/j.tig.2010.01.001.
4. Brandies P, Peel E, Hogg CJ and Belov K. The Value of Reference Genomes in the Conservation of Threatened Species. Genes. 2019;10 11 doi:10.3390/genes10110846.
5. Frankham R. Genetic rescue of small inbred populations: meta-analysis reveals large and consistent benefits of gene flow. Molecular ecology. 2015;24 11:2610-8. doi:10.1111/mec.13139.
6. Weeks AR, Heinze D, Perrin L, Stoklosa J, Hoffmann AA, van Rooyen A, et al. Genetic rescue increases fitness and aids rapid recovery of an endangered marsupial population. Nature communications. 2017;8 1:1071. doi:10.1038/s41467-017-01182-3.
7. Khan A, Patel K, Shukla H, Viswanathan A, van der Valk T, Borthakur U, et al. Genomic evidence for inbreeding depression and purging of deleterious genetic variation in Indian tigers. Proceedings of the National Academy of Sciences of the United States of America. 2021;118 49 doi:10.1073/pnas.2023018118.
8. Hu JY, Hao ZQ, Frantz L, Wu SF, Chen W, Jiang YF, et al. Genomic consequences of population decline in critically endangered pangolins and their demographic histories. National science review. 2020;7 4:798-814. doi:10.1093/nsr/nwaa031.
9. von Seth J, Dussex N, Diez-Del-Molino D, van der Valk T, Kutschera VE, Kierczak M, et al. Genomic insights into the conservation status of the world's last remaining Sumatran rhinoceros populations. Nature communications. 2021;12 1:2393. doi:10.1038/s41467-021-22386-8.
10. Xue Y, Prado-Martinez J, Sudmant PH, Narasimhan V, Ayub Q, Szpak M, et al. Mountain gorilla genomes reveal the impact of long-term population decline and inbreeding. Science. 2015;348 6231:242-5. doi:10.1126/science.aaa3952.
11. Nicolas D, Tom vdV, Hernán E. M, Christopher W. W, David D-d-M, Johanna vS, et al. Population genomics of the critically endangered kākāpō. Cell Genomics. 2021;1 1:100002. doi:10.1016/j.xgen.2021.100002.

12. Saremi NF, Supple MA, Byrne A, Cahill JA, Coutinho LL, Dalen L, et al. Puma genomes from North and South America provide insights into the genomic consequences of inbreeding. Nature communications. 2019;10 1:4769. doi:10.1038/s41467-019-12741-1.
13. Xie HX, Liang XX, Chen ZQ, Li WM, Mi CR, Li M, et al. Ancient Demographics Determine the Effectiveness of Genetic Purging in Endangered Lizards. Molecular biology and evolution. 2022;39 1 doi:10.1093/molbev/msab359.
14. Hua L, Gong S, Wang F, Li W, Ge Y, Li X, et al. Captive breeding of pangolins: current status, problems and future prospects. ZooKeys. 2015; 507:99-114. doi:10.3897/zookeys.507.6970.
15. Kondrashov P and Agadjanian AK. A nearly complete skeleton ofErnanodon(Mammalia, Palaeanodonta) from Mongolia: morphofunctional analysis. Journal of Vertebrate Paleontology. 2012;32 5:983-1001. doi:10.1080/02724634.2012.694319.
16. Choo SW, Rayko M, Tan TK, Hari R, Komissarov A, Wee WY, et al. Pangolin genomes and the evolution of mammalian scales and immunity. Genome research. 2016;26 10:1312-22.
17. Ferreira-Cardoso S, Billet G, Gaubert P, Delsuc F and Hautier L. Skull shape variation in extant pangolins (Pholidota: Manidae): allometric patterns and systematic implications. Zoological Journal of the Linnean Society. 2019; doi:10.1093/zoolinnean/zlz096.
18. Heinrich S, Wittman TA, Ross JV, Shepherd CR, Challender DWS and Cassey P. THE GLOBAL TRAFFICKING OF PANGOLINS: A comprehensive summary of seizures and trafficking routes from 2010–2015. Petaling Jaya: TRAFFIC, Southeast Asia Regional Office. 2017.
19. Zhang F, Wu S and Cen P. The past, present and future of the pangolin in Mainland China. Global Ecology and Conservation. 2022;33:e01995. doi:10.1016/j.gecco.2021.e01995.
20. Challender DWS, Harrop SR and MacMillan DC. Understanding markets to conserve trade-threatened species in CITES. Biological Conservation. 2015;187:249-59. doi:10.1016/j.biocon.2015.04.015.
21. Nijman V, Zhang MX and Shepherd CR. Pangolin trade in the Mong La wildlife market and the role of Myanmar in the smuggling of pangolins into China. Global Ecology and Conservation. 2016;5:118-26. doi:10.1016/j.gecco.2015.12.003.
22. Cheng W, Xing S and Bonebrake TC. Recent Pangolin Seizures in China Reveal Priority Areas for Intervention. Conservation Letters. 2017;10 6:757-64. doi:10.1111/conl.12339.
23. Zhang F, Yu Y, Wu S, Mahmood A, Yu J and Min Y. Reducing Pangolin Demand by Understanding Motivations for Human Consumption in Guangdong, China. Frontiers in Ecology and Evolution. 2020;8 doi:10.3389/fevo.2020.574161.
24. Wang Q, Lan T, Li H, Sahu SK, Shi M, Zhu Y, et al. Whole-genome resequencing of Chinese pangolins reveals a population structure and provides insights into their conservation. Communications biology. 2022;5 1:821. doi:10.1038/s42003-022-03757-3.
25. Wei S, Fan H, Zhou W, Huang G, Hua Y, Wu S, et al. Conservation genomics of the critically endangered Chinese pangolin. Science China Life Sciences. 2024; doi:10.1007/s11427-023-2540-y.
26. Cheng H, Concepcion GT, Feng X, Zhang H and Li H. Haplotype-resolved de novo assembly using phased assembly graphs with hifiasm. Nature methods. 2021;18 2:170-5.
27. Cheng H, Jarvis ED, Fedrigo O, Koepfli K-P, Urban L, Gemmell NJ, et al. Haplotype-resolved assembly of diploid genomes without parental data. Nature Biotechnology.

2022;40 9:1332-5.

28. Cao P, Dai Q, Deng C, Zhao X, Qin S, Yang J, et al. Genome-wide signatures of mammalian skin covering evolution. Science China Life Sciences. 2021;64 10:1765-80.

29. Damas J, Corbo M, Kim J, Turner-Maier J, Farré M, Larkin DM, et al. Evolution of the ancestral mammalian karyotype and syntenic regions. Proceedings of the National Academy of Sciences. 2022;119 40:e2209139119.

30. Heighton SP, Allio R, Murienne J, Salmona J, Meng H, Scornavacca C, et al. Pangolin genomes offer key insights and resources for the world's most trafficked wild mammals. bioRxiv. 2023; doi:10.1101/2023.02.16.528682.

31. Yan D, Luo X, Tang J, Xu S, Huang K, Wang X, et al. High-Quality Genomes of Pangolins: Insights into the Molecular Basis of Scale Formation and Adaption to Myrmecophagous Diet. Molecular biology and evolution. 2023;40 1 doi:10.1093/molbev/msac262.

32. Nie W, Wang J, Su W, Wang Y and Yang F. Chromosomal rearrangements underlying karyotype differences between Chinese pangolin (Manis pentadactyla) and Malayan pangolin (Manis javanica) revealed by chromosome painting. Chromosome research : an international journal on the molecular, supramolecular and evolutionary aspects of chromosome biology. 2009;17 3:321-9. doi:10.1007/s10577-009-9027-0.

33. Qi W, Lim YW, Patrignani A, Schlapfer P, Bratus-Neuenschwander A, Gruter S, et al. The haplotype-resolved chromosome pairs of a heterozygous diploid African cassava cultivar reveal novel pan-genome and allele-specific transcriptome features. GigaScience. 2022;11:giac028. doi:10.1093/gigascience/giac028.

34. Rhie A, McCarthy SA, Fedrigo O, Damas J, Formenti G, Koren S, et al. Towards complete and error-free genome assemblies of all vertebrate species. Nature. 2021;592 7856:737-46.

35. Kim H-K, Ham KA, Lee S-W, Choi HS, Kim H-S, Kim HK, et al. Biallelic deletion of pxdn in mice leads to anophthalmia and severe eye malformation. International Journal of Molecular Sciences. 2019;20 24:6144.

36. Segarra NG, Ballhausen D, Crawford H, Perreau M, Campos-Xavier B, van Spaendonck-Zwarts K, et al. NBAS mutations cause a multisystem disorder involving bone, connective tissue, liver, immune system, and retina. American Journal of Medical Genetics Part A. 2015;167 12:2902-12. doi:10.1002/ajmg.a.37338.

37. Kumamaru E, Kuo C-H, Fujimoto T, Kohama K, Zeng L-H, Taira E, et al. Reticulon3 expression in rat optic and olfactory systems. Neuroscience letters. 2004;356 1:17-20. doi:10.1016/j.neulet.2003.11.009.

38. Challender DW, Nash HC and Waterman C. Pangolins: science, society and conservation. Academic Press; 2019.

39. Wang Q, Lan T, Li H, Sahu SK, Shi M, Zhu Y, et al. Whole-genome resequencing of Chinese pangolins reveals a population structure and provides insights into their conservation. Communications Biology. 2022;5 1:821.

40. Wei S, Fan H, Zhou W, Huang G, Hua Y, Wu S, et al. Conservation genomics of the critically endangered Chinese pangolin. Science China Life Sciences. 2024:1-11.

41. Henn BM, Botigué LR, Bustamante CD, Clark AG and Gravel S. Estimating the mutation load in human genomes. Nature Reviews Genetics. 2015;16 6:333-43. doi:10.1038/nrg3931.

42. Low WY, Tearle R, Liu R, Koren S, Rhie A, Bickhart DM, et al. Haplotype-resolved genomes provide insights into structural variation and gene content in Angus and Brahman cattle. Nature communications. 2020;11 1:2071. doi:10.1038/s41467-020-15848-y.
43. Sun H, Jiao WB, Krause K, Campoy JA, Goel M, Folz-Donahue K, et al. Chromosome-scale and haplotype-resolved genome assembly of a tetraploid potato cultivar. Nature genetics. 2022;54 3:342-8. doi:10.1038/s41588-022-01015-0.
44. Zhang L, Lan T, Lin C, Fu W, Yuan Y, Lin K, et al. Chromosome-scale genomes reveal genomic consequences of inbreeding in the South China tiger: A comparative study with the Amur tiger. Molecular ecology resources. 2022; doi:10.1111/1755-0998.13669.
45. Yang S, Lan T, Zhang Y, Wang Q, Li H, Dussex N, et al. Genomic investigation of the Chinese alligator reveals wild-extinct genetic diversity and genomic consequences of their continuous decline. Molecular ecology resources. 2022; doi:10.1111/1755-0998.13702.
46. Wang Q, Lan T, Li H, Sahu SK, Shi M, Zhu Y, et al. Whole-genome resequencing of Chinese pangolins reveals a population structure and provides insights into their conservation. Communications Biology. 2022;5 1:821. doi:10.1038/s42003-022-03757-3.
47. Zhao S, Zheng P, Dong S, Zhan X, Wu Q, Guo X, et al. Whole-genome sequencing of giant pandas provides insights into demographic history and local adaptation. Nature genetics. 2013;45 1:67-71. doi:10.1038/ng.2494.
48. Kuang WM, Ming C, Li HP, Wu H, Frantz L, Roos C, et al. The Origin and Population History of the Endangered Golden Snub-Nosed Monkey (Rhinopithecus roxellana). Molecular biology and evolution. 2019;36 3:487-99. doi:10.1093/molbev/msy220.
49. Hu J, Roos C, Lv X, Kuang W and Yu L. Molecular Genetics Supports a Potential Fifth Asian Pangolin Species (Mammalia, Pholidota, Manis). Zoological science. 2020;37 6:538-43. doi:10.2108/zs200084.
50. Guang X, Lan T, Wan Q-H, Huang Y, Li H, Zhang M, et al. Chromosome-scale genomes provide new insights into subspecies divergence and evolutionary characteristics of the giant panda. Science Bulletin. 2021;66 19:2002-13. doi:10.1016/j.scib.2021.02.002.
51. Pecnerova P, Garcia-Erill G, Liu X, Nursyifa C, Waples RK, Santander CG, et al. High genetic diversity and low differentiation reflect the ecological versatility of the African leopard. Current biology : CB. 2021;31 9:1862-71 e5. doi:10.1016/j.cub.2021.01.064.
52. Carneiro M, Albert FW, Afonso S, Pereira RJ, Burbano H, Campos R, et al. The genomic architecture of population divergence between subspecies of the European rabbit. PLoS genetics. 2014;10 8:e1003519. doi:10.1371/journal.pgen.1003519.
53. Liu B, Shi Y, Yuan J, Hu X, Zhang H, Li N, et al. Estimation of genomic characteristics by analyzing k-mer frequency in de novo genome projects. arXiv: Genomics. 2013.
54. Guan D, McCarthy SA, Wood J, Howe K, Wang Y and Durbin R. Identifying and removing haplotypic duplication in primary genome assemblies. Bioinformatics. 2020;36 9:2896-8.
55. Durand NC, Shamim MS, Machol I, Rao SS, Huntley MH, Lander ES, et al. Juicer provides a one-click system for analyzing loop-resolution Hi-C experiments. Cell systems. 2016;3 1:95-8.
56. Li H. Aligning sequence reads, clone sequences and assembly contigs with BWA-MEM. arXiv:13033997 [q-bioGN]. 2013;0 0:3.
57. Li H and Durbin R. Fast and accurate long-read alignment with Burrows–Wheeler transform. Bioinformatics. 2010;26 5:589-95.

58. Dudchenko O, Batra SS, Omer AD, Nyquist SK, Hoeger M, Durand NC, et al. De novo assembly of the Aedes aegypti genome using Hi-C yields chromosome-length scaffolds. Science. 2017;356 6333:92-5.

59. Manni M, Berkeley MR, Seppey M, Simão FA, Zdobnov EM and Kelley J. BUSCO Update: Novel and Streamlined Workflows along with Broader and Deeper Phylogenetic Coverage for Scoring of Eukaryotic, Prokaryotic, and Viral Genomes. Molecular biology and evolution. 2021;38 10:4647-54. doi:10.1093/molbev/msab199.

60. Rhie A, Walenz BP, Koren S and Phillippy AM. Merqury: reference-free quality, completeness, and phasing assessment for genome assemblies. Genome biology. 2020;21 1 doi:10.1186/s13059-020-02134-9.

61. Marçais G, Delcher AL, Phillippy AM, Coston R, Salzberg SL and Zimin A. MUMmer4: A fast and versatile genome alignment system. PLoS computational biology. 2018;14 1:e1005944.

62. Xu Z and Wang H. LTR_FINDER: an efficient tool for the prediction of full-length LTR retrotransposons. Nucleic Acids Res. 2007;35 Web Server issue:W265-8. doi:10.1093/nar/gkm286.

63. Flynn JM, Hubley R, Goubert C, Rosen J, Clark AG, Feschotte C, et al. RepeatModeler2 for automated genomic discovery of transposable element families. Proceedings of the National Academy of Sciences of the United States of America. 2020;117 17:9451-7. doi:10.1073/pnas.1921046117.

64. Chen N. Using Repeat Masker to identify repetitive elements in genomic sequences. Current protocols in bioinformatics. 2004;5 1:4.10. 1-4.. 4.

65. Benson G. Tandem repeats finder: a program to analyze DNA sequences. Nucleic acids research. 1999;27 2:573-80. doi:10.1093/nar/27.2.573.

66. Stanke M, Steinkamp R, Waack S and Morgenstern B. AUGUSTUS: a web server for gene finding in eukaryotes. Nucleic acids research. 2004;32 Web Server issue:W309-12. doi:10.1093/nar/gkh379.

67. Majoros WH, Pertea M and Salzberg SL. TigrScan and GlimmerHMM: two open source ab initio eukaryotic gene-finders. Bioinformatics. 2004;20 16:2878-9. doi:10.1093/bioinformatics/bth315.

68. Korf I. Gene finding in novel genomes. BMC bioinformatics. 2004;5:59. doi:10.1186/1471-2105-5-59.

69. Kim D, Langmead B and Salzberg SL. HISAT: a fast spliced aligner with low memory requirements. Nature methods. 2015;12 4:357-60.

70. Pertea M, Pertea GM, Antonescu CM, Chang T-C, Mendell JT and Salzberg SL. StringTie enables improved reconstruction of a transcriptome from RNA-seq reads. Nature biotechnology. 2015;33 3:290-5.

71. Mount DW. Using the Basic Local Alignment Search Tool (BLAST). CSH protocols. 2007;2007:pdb top17. doi:10.1101/pdb.top17.

72. Campbell MS, Holt C, Moore B and Yandell M. Genome Annotation and Curation Using MAKER and MAKER-P. Current protocols in bioinformatics. 2014;48:4 11 1-39. doi:10.1002/0471250953.bi0411s48.

73. Jones P, Binns D, Chang H-Y, Fraser M, Li W, McAnulla C, et al. InterProScan 5: genome-scale protein function classification. Bioinformatics. 2014;30 9:1236-40.

doi:10.1093/bioinformatics/btu031.

74. Lowe TM and Eddy SR. tRNAscan-SE: a program for improved detection of transfer RNA genes in genomic sequence. Nucleic acids research. 1997;25 5:955-64.

75. Goel M, Sun H, Jiao W-B and Schneeberger K. SyRI: finding genomic rearrangements and local sequence differences from whole-genome assemblies. Genome biology. 2019;20 1:1-13.

76. Robinson JT, Thorvaldsdóttir H, Turner D and Mesirov JP. igv. js: an embeddable JavaScript implementation of the Integrative Genomics Viewer (IGV). BioRxiv. 2022:2020.05. 03.075499.

77. Freed D, Aldana R, Weber JA and Edwards JS. The Sentieon Genomics Tools-A fast and accurate solution to variant calling from next-generation sequence data. BioRxiv. 2017:115717.

78. Purcell S, Neale B, Todd-Brown K, Thomas L, Ferreira MA, Bender D, et al. PLINK: a tool set for whole-genome association and population-based linkage analyses. The American journal of human genetics. 2007;81 3:559-75.

79. Yang J, Lee SH, Goddard ME and Visscher PM. GCTA: a tool for genome-wide complex trait analysis. The American Journal of Human Genetics. 2011;88 1:76-82.

80. Ortiz E. vcf2phylip v2. 0: convert a VCF matrix into several matrix formats for phylogenetic analysis. URL https://doi org/105281/zenodo. 2019;2540861.

81. Darriba D, Taboada GL, Doallo R and Posada D. jModelTest 2: more models, new heuristics and parallel computing. Nature methods. 2012;9 8:772. doi:10.1038/nmeth.2109.

82. Nguyen LT, Schmidt HA, von Haeseler A and Minh BQ. IQ-TREE: a fast and effective stochastic algorithm for estimating maximum-likelihood phylogenies. Molecular biology and evolution. 2015;32 1:268-74. doi:10.1093/molbev/msu300.

83. Alexander DH, Novembre J and Lange K. Fast model-based estimation of ancestry in unrelated individuals. Genome research. 2009;19 9:1655-64.

84. Terhorst J, Kamm JA and Song YS. Robust and scalable inference of population history from hundreds of unphased whole genomes. Nature genetics. 2017;49 2:303-9.

85. Schiffels S and Durbin R. Inferring human population size and separation history from multiple genome sequences. Nature genetics. 2014;46 8:919-25. doi:10.1038/ng.3015.

86. Browning SR and Browning BL. Rapid and accurate haplotype phasing and missing-data inference for whole-genome association studies by use of localized haplotype clustering. The American Journal of Human Genetics. 2007;81 5:1084-97.

87. Dobrynin P, Liu S, Tamazian G, Xiong Z, Yurchenko AA, Krasheninnikova K, et al. Genomic legacy of the African cheetah, Acinonyx jubatus. Genome biology. 2015;16 1:1-20.

88. Danecek P, Auton A, Abecasis G, Albers CA, Banks E, DePristo MA, et al. The variant call format and VCFtools. Bioinformatics. 2011;27 15:2156-8.

89. Wang K, Li M and Hakonarson H. ANNOVAR: functional annotation of genetic variants from high-throughput sequencing data. Nucleic acids research. 2010;38 16:e164-e.

90. Cingolani P, Platts A, Wang LL, Coon M, Nguyen T, Wang L, et al. A program for annotating and predicting the effects of single nucleotide polymorphisms, SnpEff: SNPs in the genome of Drosophila melanogaster strain w1118; iso-2; iso-3. Fly. 2012;6 2:80-92.

91. Grantham R. Amino acid difference formula to help explain protein evolution. science.

1974;185 4154:862-4.
92. Feng S, Fang Q, Barnett R, Li C, Han S, Kuhlwilm M, et al. The genomic footprints of the fall and recovery of the crested ibis. Current Biology. 2019;29 2:340-9. e7.
93. Davydov EV, Goode DL, Sirota M, Cooper GM, Sidow A and Batzoglou S. Identifying a high fraction of the human genome to be under selective constraint using GERP++. PLoS computational biology. 2010;6 12:e1001025.
94. Guo X, Chen F, Gao F, Li L, Liu K, You L, et al. CNSA: a data repository for archiving omics data. Database. 2020;2020 doi:10.1093/database/baaa055.
95. Chen F, You L, Yang F, Wang L, Guo X, Gao F, et al. CNGBdb: China National GeneBank DataBase. Hereditas (Beijing). 2020;42:799-809. doi:10.16288/j.yczz.20-080.

Figure1

Click here to access/download;Figure;FIG1-2024.10.4.pdf

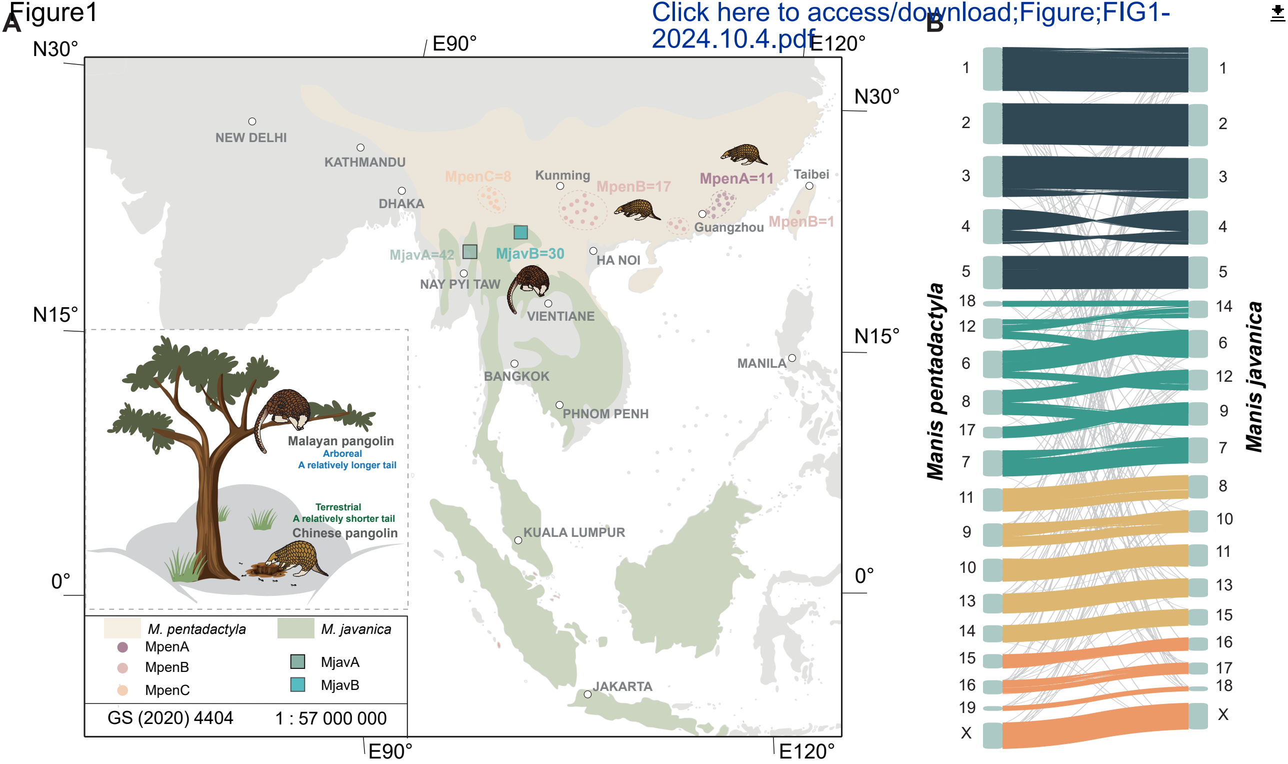

Figure2

Click here to access/download;Figure;FIG2-

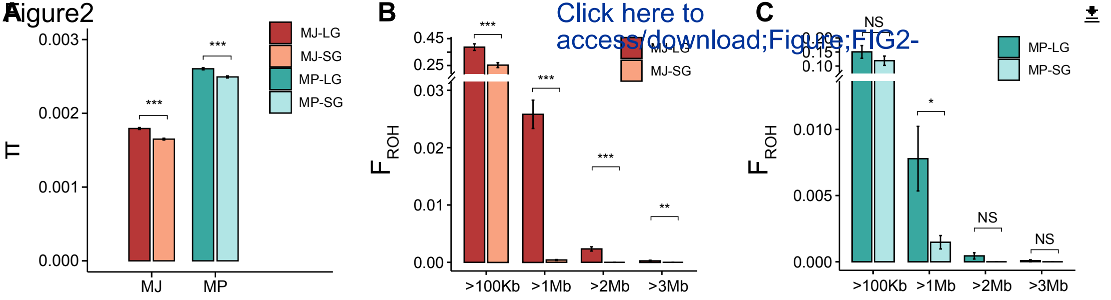

Figure 3

Click here to access/download;Figure;FIG3-

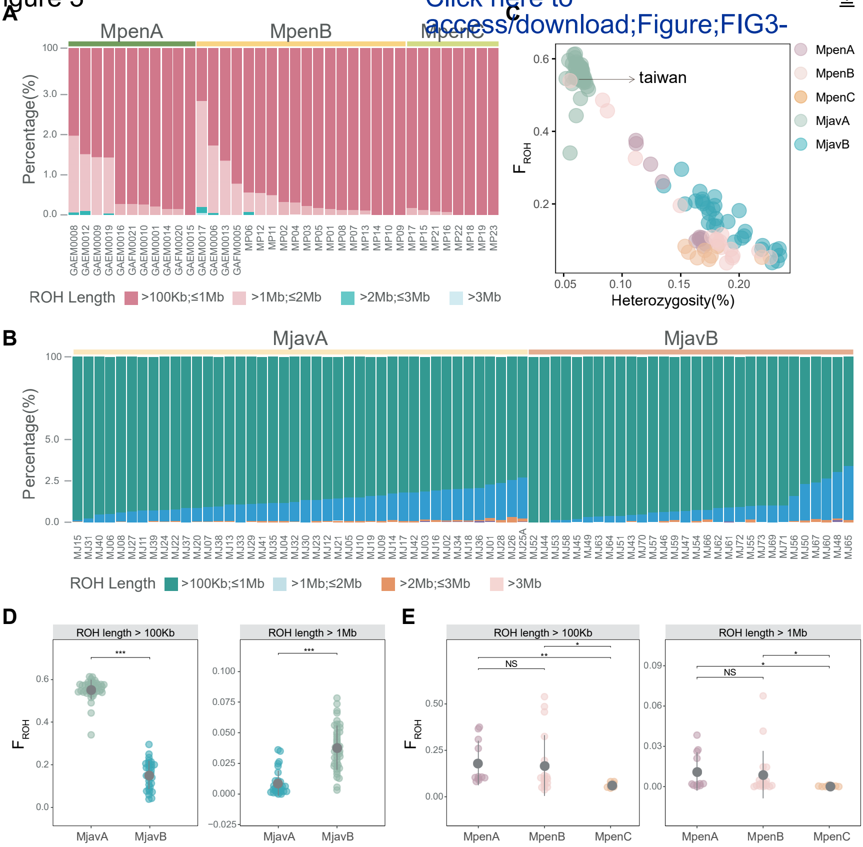

Figure4

Click here to access/download;Figure;FIG4-2024.10.5.pdf

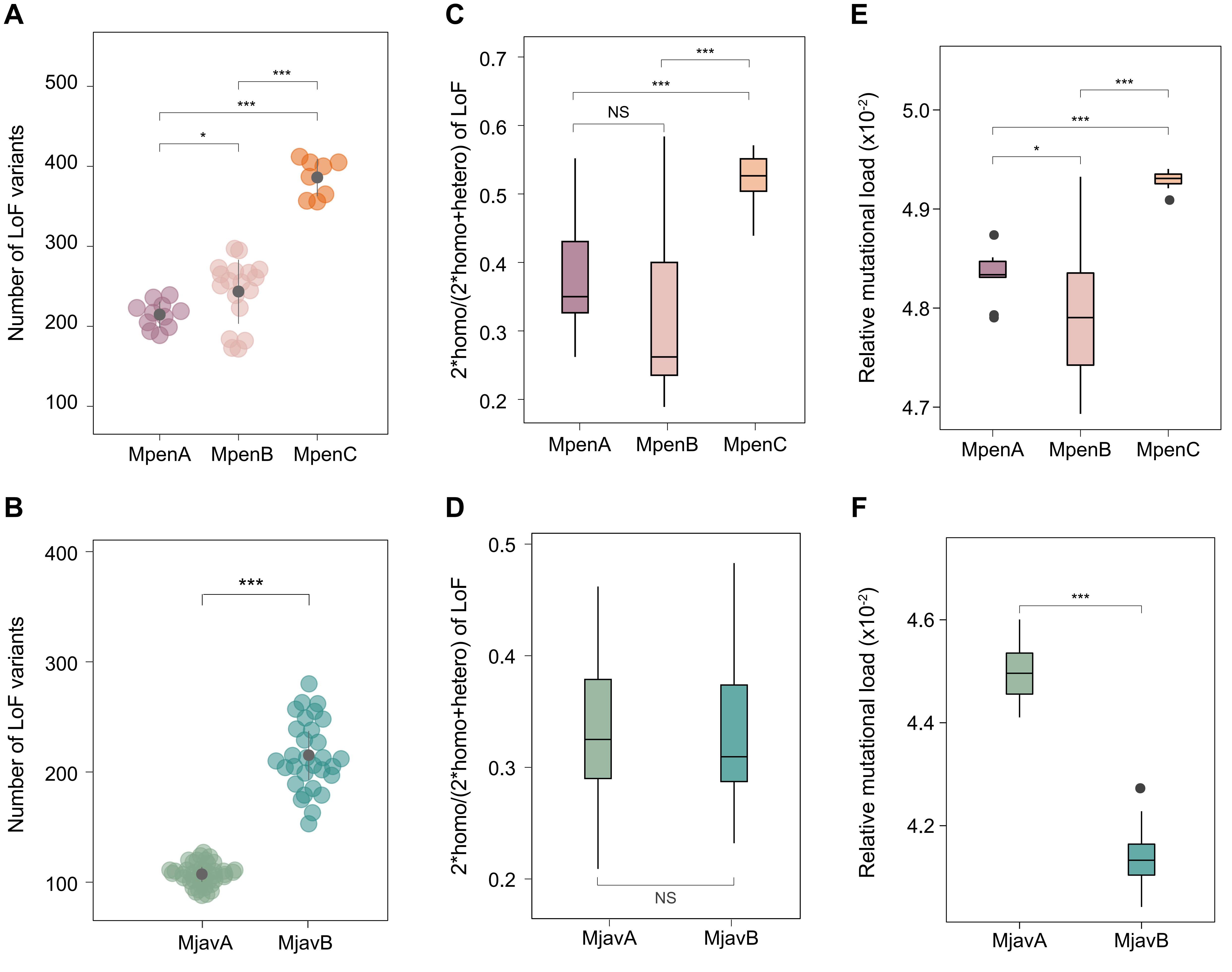

Figure5

Click here to access/download;Figure;FIG5-2024.10.4.pdf

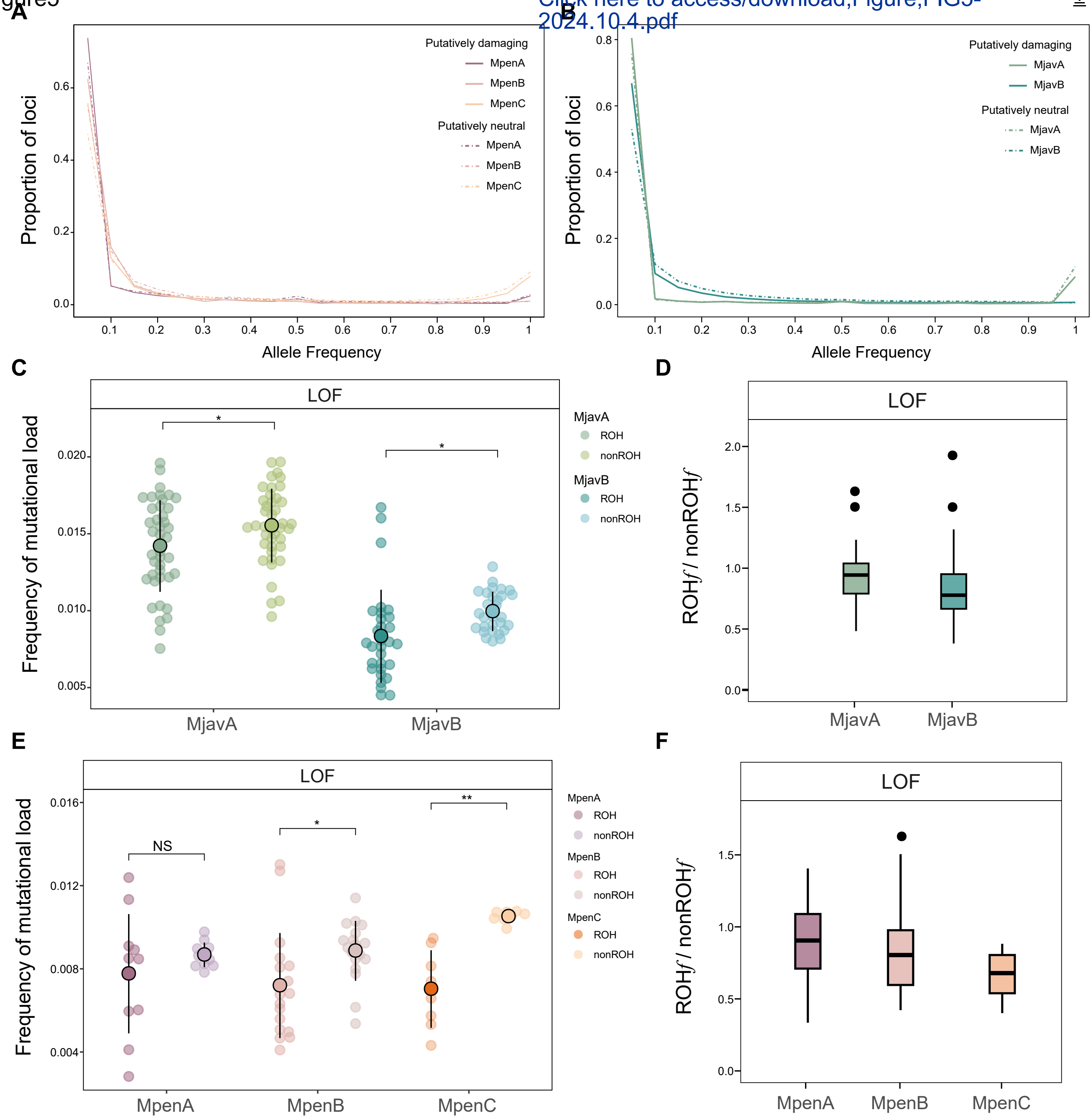

Click here to access/download
Supplementary Material
Supplemental Information-clean.docx

Click here to access/download
Supplementary Material
Supplementary tables.xlsx

Personal Cover Click here to access/download;Personal Cover;Response to reviewers.docx

Dear Editor and Reviewers,

Thank you for the many insightful comments and suggestions. We have made revisions to address all the comments. We have carefully considered each of your comments and have made the necessary revisions to address them thoroughly. Below, you will find a detailed list of our responses to the specific comments you raised:

Reviewer #1: The manuscript describes two novel VGP style genome assemblies of Malayan and Chinese pangolins. New assemblies provide a significant improvement to previously published short read references. Sequenced data and software used for assembly are reasonable. In addition to the genome assembly, the authors performed some population analysis using previously published resequencing data. I see no issues with the results of this part of the manuscript, but the discussion (comparison with previously published results) should be enhanced (as a part of review process). Title of the manuscript implies that such a comprehensive comparison is inside the text.

Major comments.

Q1. As I understood, no parental data was generated for genome assembly, and only HiFi + HiC data were generated from the reference individuals. It means that resulting assemblies are not completely haplotype resolved. Combination of HiFi and HiC allows to (nearly) completely resolve haplotypes only at the level of chromosomes, but chromosomal scaffolds are still mixed between haplotypes. It is a very common mistake to treat such an assembly as haplotype- resolved. I encourage authors to somehow underline it in the text, for example by adding "at chromosomal level" where it is necessary

Response: Thank you for pointing out this. Yes, our study did not generate parental data for genome assembly, we only utilized HiFi and HiC data from the same individual for genome assembly. We agree that the combination of HiFi and HiC data allows for nearly complete resolution of haplotypes at the chromosomal level, the chromosomal scaffolds are still mixed between haplotypes[1]. We appreciate your suggestion and will emphasize this point in the revised manuscript by including a clarification in the text, such as specifying “at the chromosomal level” where appropriate, to avoid any misinterpretation. Line 100.

Q2. QV scores look relatively low (<50). I would expect something like 55-60+ for such input data.

Response: Thank you for this comment. In the original version of this manuscript, we utilized short reads sequencing data to calculate the QV scores (MJ: 48.56; MJ: 49.63) by the Merqury software. In the revised manuscript, we polished the assemblies by HiFi long reads and WGS short reads, and used the high quality HiFi reads to calculate the QV scores by the Merqury software [3, 4]. The result showed that the QV score for the MP and MJ were 57.06 and 55.08, respectively, which means ~ 0.00019% and 0.00031% of base calling error rate, respectively, which could reflect a very high accuracy for our genome assembly. In addition, we have conducted an extensive search of published papers describing genomes with QV analysis by the Merqury software. We found that a number of published genomes showed QV values ranging from the 30 to 50 [5-7], which means the error rate of the base calling ranged from 0.1% to 0.001%. Therefore, we agree with you, a QV score of 55-60+ would be much better than < 50 in our previously assembled genomes.

Q3. Figure 1C is very difficult to understand. I encourage author to remove it completely or do a

linear plot (or set of them) similar to Figure 1D

Response: Thank you to point out this. We have moved Figure 1C to the supplementary materials (Fig S17a) and added a linear plot here, as you suggested (Fig S17b-d).

Q4. row 162-163: "All these SVs were validated by our assembled contigs (Supplementary Fig. S9)". Figure is very unclear, and a reader may expect some results to be present on the figure and not the methods. Also I would expect some inversions encompassing whole contigs (especially at the end of chromosomes, where coverage often drops and contigs are shorter). Such inversions might be an artifacts of the HiC scaffolding and are very common. Please confirm that you have found none of them.

Response: Thanks for your suggestion. In response to the issue with the unclear figure, we have added a result description in the supplementary Fig. S18. This figure is intended to illustrate the validation of SVs by contigs mapping. For the potential issue of inversions, especially at the end of chromosome, we have meticulously re-examined our data and have made the necessary revisions to the structural variant (SV) results, we have taken great care to ensure that the SVs are accurately represented and inversions that are potentially be artifacts of HiC scaffolding have been all corrected in our analysis (Table S21).

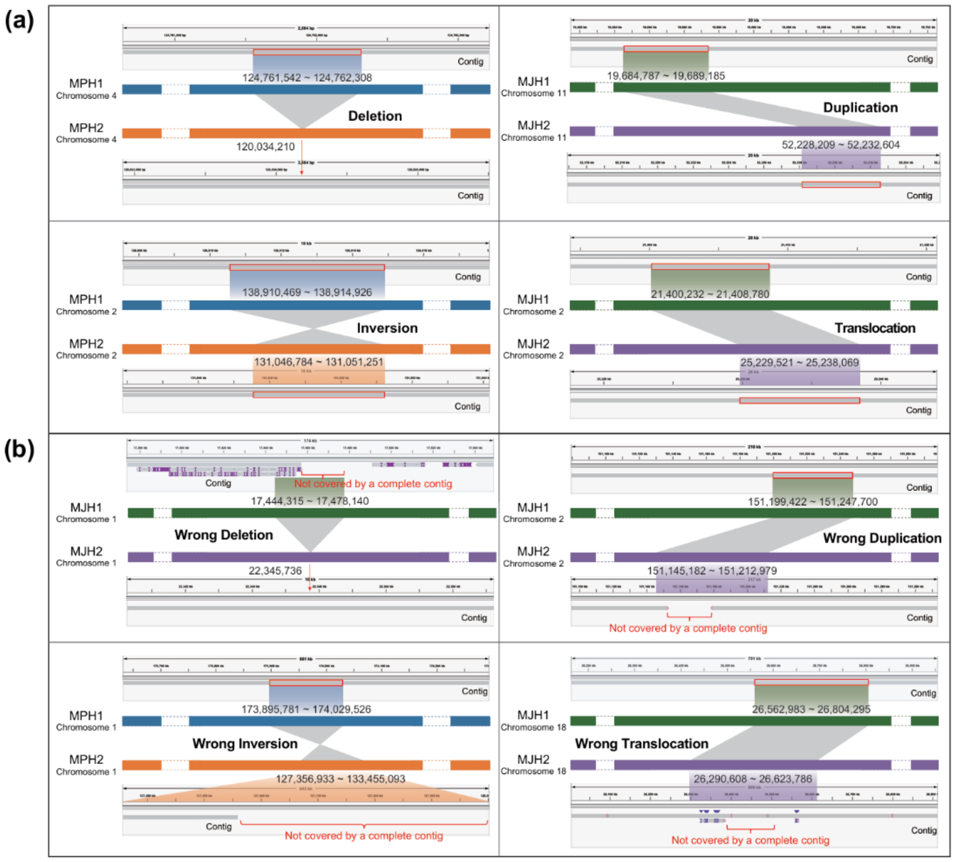

Response figure 1. Validation of structural variants through contig mapping. Contigs are mapped to both the haploid genomes to verify structural variants and visualized by the IGV software. (a) The correct structural variants between the haploid genomes. The breakpoints of the SVs could be covered by complete contigs in both of the haploid genomes. (b) The incorrect structural variants between the haploid genomes. The breakpoints of the SVs in one of the two haploid genomes could not be covered by the complete contig. In the IGV screenshot, the gray bar represents

the contig spanning over the SVs, and the contig corresponding SV regions were marked as the red box. All structural variants have been verified, and several are randomly displayed here.

Q5. row 311-316. A bit of a strange wording. "Much better contiguity, which is … longer". Please, simplify. For example by just comparing N50s in the main text

Response: Thank you. We agree that comparing the N50 values would provide a clearer and more straightforward comparison. We've revised the description here to make it more readable. Please refer to the supplementary material, lines 42-48, for the updated information.

Q6. Manuscript lacks a comparison (and even mention of) with chromosome-length assemblies of malayan and Chinese pangolins generated by DNAzoo team (upgrade of two draft assemblies from NCBI). They are available at dnazoo.org. Such a comparison makes more sense than comparing with old drafts.

Response: Thank you for your reminder. We download the genomes of Malayan pangolin and Chinese pangolin from the DNAzoo website. And we added the comparison with all assemblies and presented the result in Supplementary Table S19. By this comparison, we found that the scaffold N50 of the genomes we assembled in this study are remarkably higher than those of the genomes published by DNAzoo. This comparison further showed the superiority of our assemblies in contiguity, thank you.

Q7. Paragraphs "Genome-wide extinction risks in pangolin populations" and "Novel implications for the global conservation of these two pangolin species" confuse me a bit. Authors state that they have not generated the resequencing data, but downloaded it from public databases. Dataset set includes data from three publications (references [25, 33, 57]). Majority of it (nearly all Malayan pangolin samples and >60% of Chinese pangolin) were analyzed in [25]. All Chinese pangolin samples were analyzed in [57]. Authors cite both publications several times, but text lacks a more detailed comparison between the manuscript's results and previously published ones. It is especially crucial and interesting for Chinese pangolin as there is a 100% overlap in samples with [57]. The title of the manuscript requires an extensive comparison with previous publications in all the discussion.

Response: We appreciate your comment and we agree. One of the most important points in this this manuscript is to discuss how a high-quality genome could improve the evaluation of genetic parameters in conservation genomics or in population genomics, particularly in evaluation of genetic diversity and inbreeding by screening ROH across the genome, because a reference genome with low genome contiguity cannot well support the detection of long fragments of runs of homozygosity (ROH):

1) We found much higher genetic diversity than that reported by[8, 9], indicating that we may have detected more variants across the genome based on high-quality reference genome assembled in this study, because HiFi long reads could span much more complex genomic regions[10] and generate longer contigs [11, 12], and many genomic regions that cannot be assembled by short reads can be assembled by long reads, and these regions may contain important variants.

2) The inbreeding level (ROH detection) detected based on the reference genome we assembled in this study was significantly higher than that identified using the short reads assembled genome,

particularly for ROHs larger than 1Mb, which is largely due to that the short contigs in the reference genome cannot span over long ROH fragments. By comparing with previously published short-read assembled genomes, findings in this study could tell the reader the superiority of a reference genome with better contiguity on the detection of genetic diversity and ROH fragments.

In addition, we have incorporated comparisons of our results with the results published by other studies, which can be found in many places throughout the manuscript, particularly in the results section:

1) The trajectory of the change of effective population size of Malayan pangolins presented in this study is different from that reported by Hu et al. We inferred that the MjavA (MJA in Hu et al.) population experienced a continuous population decline and the population size was lower than that of the MjavB (MJB in Hu et al.) population within the most recent 10,000 years, which could be well matched with the lower genetic diversity and higher genome-wide inbreeding in the MjavA population. But in the previous report, Hu et al,. found that the population size of the MjavA was much greater than the MjavB population from 10 kya to 3.2 kya. This difference is largely due to the different methods we used, and we did not used the method in Hu et al. because we have compared these two methods in our previous study[13] and found that this method is not as stable as the method we used in our study.

2) The parameters used in the filtering of genome-wide SNPs in Hu et al,. is too strict, SNPs with a minor allele frequency less than 0.2 will be all removed from the data set, which will overestimate the inbreeding across the population by erroneously introducing long ROH fragments (>1Mb). And in our study, we found much less long ROH fragments (>1Mb) than that in the Hu et al,. even if we used HiFi genome for reference. The parameters used for identifying the ancestral state in Wang et al,. is relatively rough by considering alleles in the Malayan pangolin genome as the ancestry state. In this study, we used the more commonly used method to identify the ancestral allele, which is more reliable. Therefore, the accumulation of derived mutational load for the Chinese pangolin populations in our study is different from that in Wang et al. We added this comparison in the maintext.

3) The genetic purging is a very important issue in the conservation genomics, genetic purging always exists in a population under purifying selection[14]. In general, deleterious alleles affecting fitness and viability are initially rare and may be hidden in the heterozygous state, but are more easily to be cleared from the population when these deleterious alleles are exposed in a homozygous state (Purging). This is an indicator for the evaluation of the extinction risk of endangered species (Response figure 2). However, in previous study (Hu et al, Wang et al and Wei et al), they did not mention this process in pangolins. In this study we discussed genetic purging in both Chinese and Malayan pangolins.

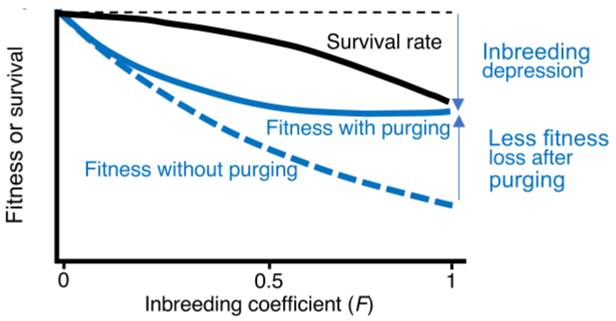

Response figure 2. The relationship between fitness and inbreeding[15].

Minor comments:

Q8. row 36-44: first paragraph is redundant and can be safely removed. It describes very basic things and facts.

Response: Thank you for your comment. We removed this paragraph in the revised manuscript. Lines 41-49.

Q9. row 60: Pangolin is a prehistoric mammal = "prehistoric" doesn't look like a good term here. Maybe 'living fossil' is better

Response: Thank you and we revised. Line 67.

Q10. row 81: remove 'recently introduced'

Response: Removed, thank you. Line 92.

Q11. row 82: "HiFi-specific assembler can generate high-quality and haplotype-resolved de novo assemblies" HiFi data itself (without HiC or parental data) can't produce haplotype-resolved assembly. Please, Correct

Response: Thank you for your careful reminder. We have modified the description to make this part more rigorous. Line 93.

Reviewer #2: This paper presents two new chromosome-level assemblies for two species of Pangolin, alongside some population genetic data from different populations of each. The quality of the genomes appears to be very high, and these data will indeed "provide valuable genomic resource for future studies on the protection and conservation for pangolins". However, this is Research Article and not a Data Note, and so it also needs a clear narrative relating to the biological insights and which aspects of the data are of most interest. This is currently hampered by numerous language errors, inconsistent formatting, and excessive reference to relatively uninteresting supplementary data. Having some clearer aims/questions beyond a better genome would be helpful in this regard.

If there is no clear message, the authors could consider a data note instead. Either way, I think a lot of the results should be consolidated and presented as Supplementary Results to emphasise the important/interesting data. There is also excessive comparison of results using their long-read and an unknown short-read genome, which should be reduced. (See detailed comments, below.) The manuscript would greatly benefit from professional editing or detailed proof-reading by a native English speaker.

Response: Thank you for your insightful comments for this manuscript. We are glad that the reviewer noticed the high quality of the chromosome-level assemblies for the two pangolin species and the potential value of our data as a resource for future conservation efforts.

We understand your concern about the need for a clearer narrative that highlights the biological insights and the most significant aspects of our data. In response to your suggestion, we have further revised the discussion section to better articulate the key research questions and the significance of our results. We removed the first part of the discussion and the description of the genome assembly in the result part to the supplementary data to help emphasize the most important findings.

Additionally, we have invited a native speaker to polish the revised manuscript and we believe that the language has been much improved.

MAJOR COMMENTS

Q12. For a Research Article, the main narrative is very unclear and the reader has to do far too much work to extract the relevant insights. The abstract hints at a "greater(?) improvement" and "different results in detecting genome-wide extinction risks being compared with short read assembled genome", though also results "were verified again" so are not actually different to previous findings? Except maybe one population (MJ2). This is all vague and confusing. Please clearly identify the key take-home messages from the new data, be explicit about it, and present a unified narrative from Abstract to Conclusions. Is the "relative mild genetic purging in pangolin populations" important? Is the purging itself the new finding, or the fact that it is relatively mild? I found the entire section (L268-294) to be rather confusing with a lack of clarity regarding population-specific versus general conclusions. The narrative would be aided greatly if the subheadings highlighted the key results/points rather than simply breaking the manuscript up into topics.

Response: Thank you for pointing out this issue, which we think could help improve this manuscript a lot. We try to answer your question and meanwhile re-organize the manuscript to make it more logic and clearer.

Yes, we obtained different results on the detection of genetic diversity, inbreeding, and accumulation of derived mutational load, based on the long-read assembled genome we showed in this study, when compared with studies reported by Hu et al, Wang et al and Wei et al. However, we did not directly compare with their studies to avoid over overemphasis this difference to showed superiority in this study? We just state our results, however, we almost didn't show duplicated results with previous study. In the revised manuscript, we added some direct comparison to make it clearer, which could be found at many places throughout the manuscript.

Actually, this manuscript mainly focused on how a high-quality genome could improve the study of conservation genomics, and this manuscript is the first time (as far as I know) to compare the short-read assembled genome and long-read assembled genome on the evaluation of genetic parameters of conservation genomics or population genomics, although the comparison may be relative limited. In this manuscript, we prepared whole paragraph to discuss this comparison (Lines 417-440).

Based on this comparison, we showed the superiority of the long-reads assembled genome to evaluate genetic diversity and inbreeding, further discussed genetic diversity and inbreeding we calculated based on the high-quality genome in this study, which could help us re-understand the

genome-wide extinction risk of the two pangolin species. We also updated the result of ROH screening and accumulation of mutational load by using more appropriate parameters, please refer to **Q7**.

Furthermore, as you mentioned, we also explored the genetic purging in the five pangolin populations. The genetic purging always exists in a population under purifying selection[14], and is a very important issue in the conservation genomics. In general, deleterious alleles affecting fitness and viability are initially rare and may be hidden in the heterozygous state, but are more easily to be cleared from the population when these deleterious alleles are exposed in a homozygous state (Purging). This is an indicator for the evaluation of the extinction risk of endangered species. However, the genetic purging has been never discussed in (Hu et al, Wang et al and Wei et al). In this study we detected and discussed genetic purging in both Chinese and Malayan pangolin populations. Therefore, both the genetic purging and the "mild" are new findings in this study. We also revised the subheading to highlighted the key results/points in this study. Thank you again.

Q13. There are far too many supplementary figures and tables. I recommend trying to consolidate some (eg. S23-S28) into fewer multipanel figures that address the same question. Likewise, many of the tables could be combined and consolidated as the same data is presented for MJ and MP in two table, or tables with the same rows (samples) could be combined into a bigger table. Ideally, some of the descriptive results could be moved from the main manuscript body into a Supplementary Results section along with many of the references to these supplementary data. It would be helpful if the figures could be more carefully selected and organised/labelled to support the narrative.

Response: We appreciate your suggestions regarding the supplementary figures and tables. We have addressed these issues as follows: 1) We combined several groups of figures into multipanel figures, including you mentioned S23-S28. 2) We have reviewed the tables and combined those tables presenting similar data for MJ and MP into single tables where appropriate. Additionally, we merged tables with overlapping rows (samples) to create bigger tables. 3) We moved some descriptive results from the main manuscript to a Supplementary Results section to enhance the readability of the main text. We also adjust references to supplementary data accordingly. 4) We carefully selected and organized the figures, ensuring that they are well-labeled and directly support the narrative of the manuscript. We believe that the narrative of the revised manuscript has been much improved.

Q14. There is insufficient information and discussion about the previous pangolin genomes. Most important, genome "SG" does not seem to be defined anywhere. Is this a previously published genome? Or a short-read assembly of new data? There is a bit of discussion about the previous genomes (L306-323) but the comparisons in Tables S31 and S32 are unclear - are the new genomes missing from these tables? (What are they called?) I think this should be the first Results section. As these are phased chromosome-level vertebrate genomes, assembly statistics should be presented in the context of VGP/EBP standards. There should also be data presented for both haplotypes in Table 1.

Response: Thank you for your concern. "SG" stands for "short-read assembled genome", referring to a genome that was assembled based on the short-read sequencing technology. In this study, the SG genomes specifically referred to the genome of the Malayan pangolin of the YNU_ManPten_2.0 and of the Chinese pangolin of the YNU_ManJav_2.0 (collected from the NCBI). These two genomes have been widely used in previous studies, and we want to use this genome as a

representative for the short-read assembled genome with low contiguity for comparison with the long-read assembled genome we prepared in this study. We defined it in Figure legend and Abbreviations section, we have added further explanations in the revised manuscript. Lines 242 and 957

To facilitate the reading and understanding of the differences between these published pangolin genomes and genomes assembled in this study, we added required information of our newly assembled genomes in Supplementary Tables 19. We have named our assemblies following the Vertebrate Genome Project (VGP) conventions and ToLIDs. Specifically, the Malayan pangolin genome assembly was named as mManJav1.1, and the Chinese pangolin genome assembly was named as mManPen9.1, and we have registered in the ToLIDs. In the Results section, we have provided detailed description of characteristics of our new assemblies in Table S3 and S4, and included a detailed description of assembly statistics in accordance with VGP/EBP standards (Table 1).

Q15. The SG vs LG comparison is largely a distraction and floods the paper with boring SI. It is no surprise that LG is better for SVs and long ROH. This is a research paper about pangolins, not a methods paper about LR vs SR genomes. Please reduce this to a clearer question: have there been any previous conclusions based on the other genomes that have been contradicted or (in)validated by the new genomes? Please give your new assemblies names that distinguish them from both each other and the previous assemblies - I recommend getting ToLIDs for your samples and naming the assemblies according to VGP conventions.

Response: Thank you for pointing out this. As we mentioned in the **Q12**, how a high-quality genome could improve the study of conservation genomics is a very important part in this manuscript. I agree with you that it is no surprise that LG is better for SVs and long ROH. As you mentioned, we can predict that a LG genome would be better than SG genome for the detection of SVs and long ROH fragments. However, many researchers may don't know under what situation a LG genome will present a better performance than a SG genome, and to what extent a SG genome worse than a LG genome. As you could see in many conservation genomics papers, the inbreeding, genetic diversity and the accumulation of mutational load are all very very important genetic factors that could help wildlife managers (maybe the government) to make conservation decisions. An accurate evaluation of these genetic factors is a big thing in this research field. But unfortunately, I did not see a comprehensive evaluation and comparison between the SG and LG on the impact of evaluating of parameters in conservation genetics. In addition, as you know, for many people who used genome as a tool to guild conservation practice are actually not experts on genomics or freshman in this field, they need a guild to do their works? As far as I know, this is the first time we comprehensively compared the impact of the SG and LG on the evaluation of conservation genomics related parameters. Although it is not surprising that LG is better for SVs and long ROH, but we provided many detailed things other than a simple conclusion that the LG is better than the SG, which we think is very useful for wildlife conservation researchers and workers? Yes, we still agree with your points and we have tried to shorten this comparison in this revised manuscript.

Actually, in this manuscript, we almost only showed the results that were different or contradict (at least improved) with that reported in Hu et al and Wang et al:

1) We obtained different results on the detection of genetic diversity, inbreeding, and accumulation

of derived mutational load, based on the long-read assembled genome we showed in this study, when compared with studies reported by Hu et al and Wang et al. Based on this comparison, we showed the superiority of the long-reads assembled genome to evaluate genetic diversity and inbreeding, further discussed genetic diversity and inbreeding based on the high-quality genome assembled in this study. Another point is that, we showed the very detail map of ROH distribution across each chromosome in the genome, which is not showed in Hu et al, Wang et al and Wei et al, and this information is very helpful for guiding the future genetic rescue of pangolin populations. For example: the breeding of Chinese pangolin in China is very successful and there are more than 100 captive individuals by far in the breeding center, but if we consider the reintroduction program, which individual should be selected to the wild? We could then select more appropriate candidate by referring this ROH map? But not to refer a very rough or a general inbreeding coefficient.

However, we did not directly compare result in this manuscript with previous studies (Hu et al and Wang et al) to avoid over overemphasis this difference to showed superiority in this study?, because the science and technology are moving forward. Although we just state our results, we almost didn't show duplicated results which has been reported in previous studies. But you are right, in the revised manuscript, we added some direct comparison to make it clearer.

2) Furthermore, we also explored the genetic purging in the five pangolin populations, which are never discussed in Hu et al, Wang et al and Wei et al. In general, deleterious alleles affecting fitness and viability are initially rare and may be hidden in the heterozygous state, but are more easily to be cleared from the population when these deleterious alleles are exposed in a homozygous state, we could say that this process is genetic purging. The genetic purging always exists in a population under purifying selection[14], and is a very important issue in the conservation genomics.

3) In addition, we have updated the results reported in previous study as we mentioned in **Q7 and Q12**.

To ensure clear distinction and proper identification of our new assemblies in the context of existing genomic data, we assigned unique names to our assemblies by following your advice. We obtained ToLIDs (Taxonomic IDs of Life) for our samples (MJ: mManJav1.1; MP:mManPen9.1), which will facilitate their recognition and propagate across the scientific community. In line with the VGP (Vertebrate Genomes Project) conventions, we renamed our assemblies accordingly, and incorporated these new names into the revised manuscript and ensure they are used consistently throughout the text, tables, and figures.

Q16. Do the new genetic data support the previous population groupings? (Was this based on genetic data itself?) To my eye, the genetic data presented in Fig S15 and S16 do not entirely support the existing population definitions. This needs more discussion. Is only a subset of MJ1 truly different to MJ2? Is there a fourth subpopulation of MP including the Taiwan sample?

Response: Yes, the genetic data supported the previous population grouping and this is almost based on genetic data itself, because the sampling locations for these samples are not all very clear.

In this manuscript, we mainly focused on the comparison of the grouping results (PCA, phylogenetic tree and admixture) generated based on the SG and LG, and to explore the potential impact of the reference genome to the genetic structure analysis. We found that the two groups of results are almost the same, and were very consistent with that reported in Hu et al and Wang et al

(Supplementary Fig. S4-S7). Therefore, we did not present very detailed discussion about the genetic structure in this study to avoid repeated discussion. However, we still added a short description about the Taiwan individual in Lines 224-225.

Q17. Is it possible to clarify the "variable" karyotype from ref 47? Is this biological or technical variation in results? Is both species or just Mpen? Do your results clarify the karyotype in this population - the variation is geographical, right?

Response: As description in reference 47, the Chinese pangolin has been documented to exhibit four different groups of karyotypes, with the chromosome numbers varying from 2n = 36 to 2n = 42 across published research[16-18]. Yes, we cannot confirm from the reference 47 whether the Chinese pangolin's karyotype is variable, because 1) the 2n = 36 karyotype found in India may actually belong to *M. crassicaudata*[19], 2) the 2n = 42 from Taiwan could be due to a counting error[18]. It appears that the variability of the Chinese pangolin's karyotype cannot be confirmed based on reference 47. Therefore, We have therefore modified this description.

It is difficult to definitively determine whether the variable karyotype of the Chinese pangolin is resulted from biological variation or technical variation. This could be attributed to the different methods used for counting chromosomes, the techniques for preparing and staining the chromosomes, or the criteria used for identifying and counting, and so forth. Some of the variation might also stem from misidentification of species. As mentioned, the 2n = 36 karyotype found in India might actually belong to *M. crassicaudata* rather than *M. pentadactyla*.

The discussion in the ref 47 is specific to *M. pentadactyla*, and does not mention the *M. javanica* or other species in the context of the variations in diploid numbers. Also, we do not have karyotype data or HiC data from individuals from different populations, so we cannot confirm whether the karyotype variation (if have) existed in different populations.

Can you also confirm that the apparent swapping of chromosome arms on Chr4 between the species is not just a scaffolding error. Are these karyotypes consistent in both haplotypes?

Response: Thank you for your reminder. We used contigs to verify whether the swapping of chromosome arms on Chr4 was a scaffolding error (Response figure 3). The verification is as follows:

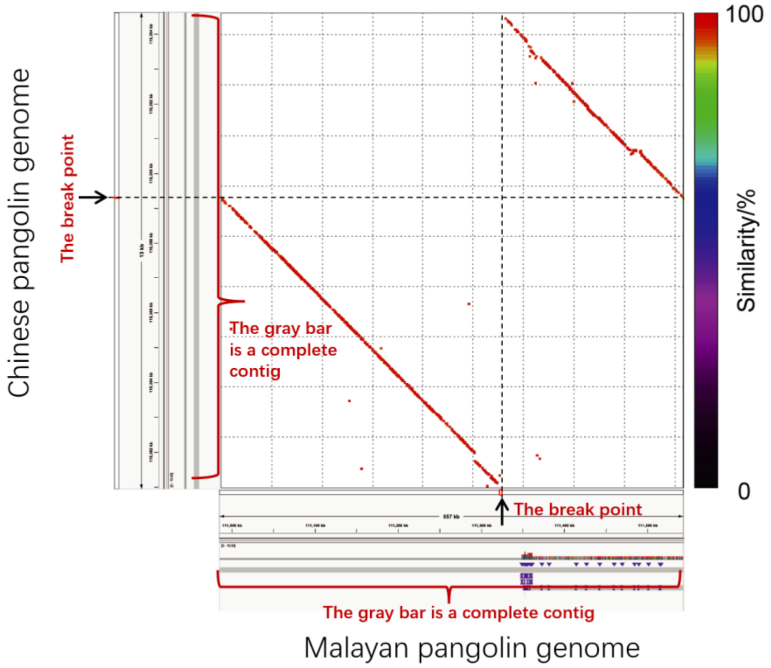

Response figure 3. Contig alignment across the region confirms the accurate rearrangement of Chromosome 4 swapping.

As shown in the figure above, by aligning contigs to Chromosome 4, we can see that both the breakpoints in the Chinese pangolin genome and the Malayan pangolin genome could be covered by a complete contig, which confirmed the accuracy of the assembly at this region, verifying the presence of the swapping showed in the main figure.

Yes, the karyotypes is consistent in both haplotypes. In Fig S1, we have showed the Hi-C heatmaps for each haplotype to demonstrate that both haplotypes possess the same karyotype numbers. We also compared the haploid and diploid genomes separately, and the collinearity of all chromosomes is consistent (Response figure 4), further confirming the karyotypic concordance between the haploid and diploid genomes.

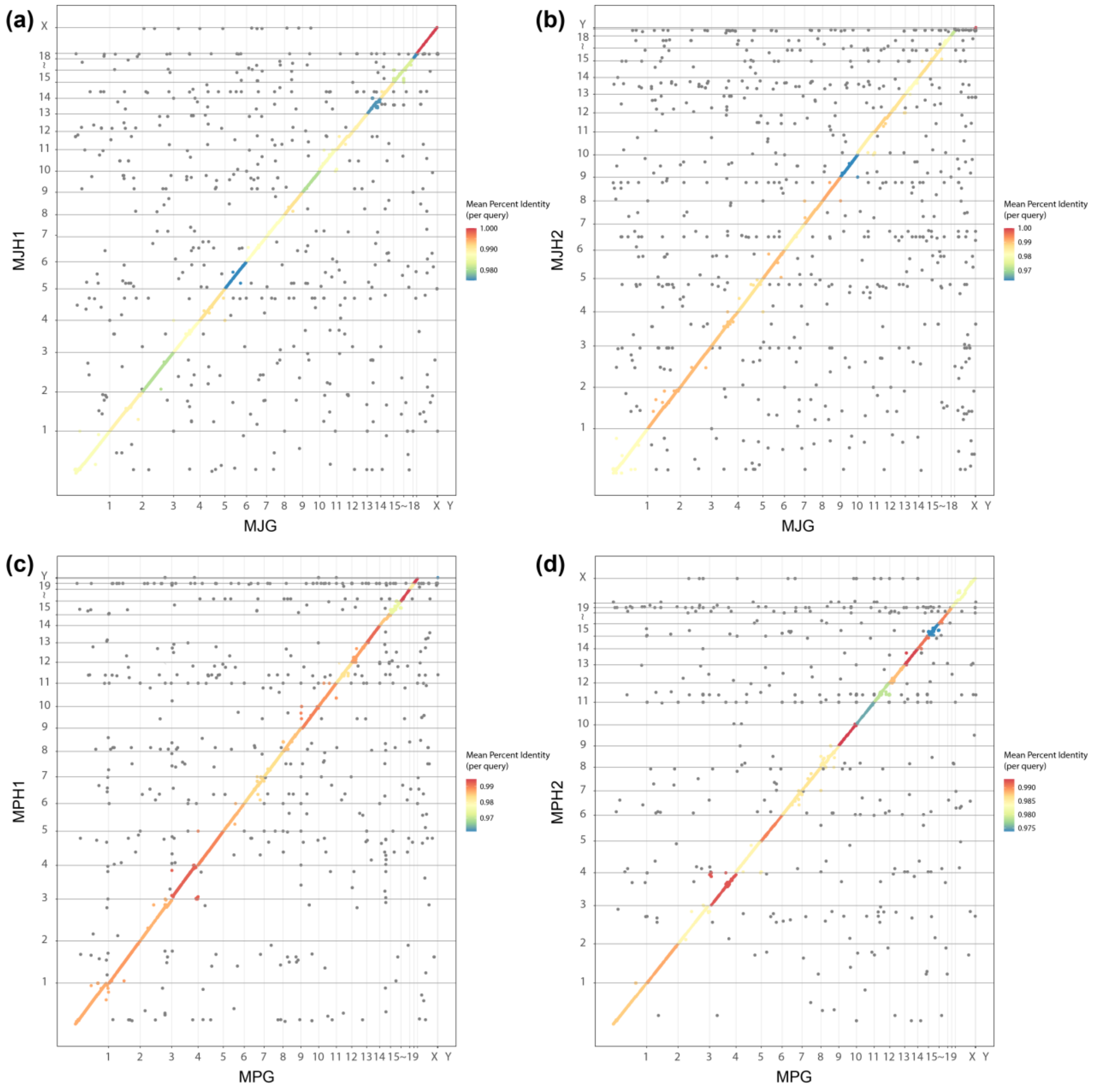

Response figure 4. (a) Synteny analysis reveals genomic consistency between haploid (MJH1) and diploid (MJG) Malayan pangolin genomes. (b) Synteny analysis reveals genomic consistency between haploid (MJH2) and diploid (MJG) Malayan pangolin genomes. (c) Synteny analysis reveals genomic consistency between haploid (MPH1) and diploid (MPG) Chinese pangolin genomes. (d) Synteny analysis reveals genomic consistency between haploid (MPH2) and diploid (MPG) Chinese pangolin genomes.

It would be good to see the contact maps (Fig S1) and synteny plots (Fig 1D) of the haplotypes against each other and have the telomeres marked to verify the orientation of arms where possible.

Response: The synteny plots between haplotypes were presented in Fig S17 (c) and (d). Following your suggestion, we have made every effort to identify the telomere status of our assembled genomes; telomeres in some chromosomes could be assembled, which are marked in Response table 1. Due to the limitations of sequencing technology, this study does not represent a complete telomere-to-telomere (T2T) pangolin genomes, therefore, telomeres sequences in some chromosomes could not be identified.

Response table 1. The distribution of telomeres in the genomes. “no” means no telomere sequence is identified, “left” means telomere sequence in the left end of the chromosome is identified, “right” represents telomere sequence in

the right end of the chromosome is identified, “both” means telomere sequence in both ends of the chromosome is identified.

MJH1	status	MJH2	status	MPH1	status	MPH2	status
1	right	1	no	1	no	1	both
2	no	2	no	2	right	2	both
3	no	3	no	3	no	3	both
4	left	4	no	4	no	4	no
5	no	5	no	5	no	5	no
6	no	6	no	6	right	6	left
7	no	7	no	7	no	7	both
8	no	8	no	8	no	8	no
9	no	9	no	9	no	9	both
10	no	10	no	10	no	10	left
11	no	11	no	11	no	11	no
12	no	12	no	12	no	12	left
13	no	13	no	13	left	13	right
14	no	14	no	14	no	14	no
15	no	15	no	15	no	15	both
16	right	16	no	16	no	16	no
17	no	17	no	17	left	17	left
18	no	18	no	18	no	18	right
19	no	19	no	19	no	19	both
				20	no	20	both

Is Fig S1 both haplotypes? If so, please mark the actual chromosome boundaries and orient the pairs in the same direction. Is the X pattern a pair of chromosomes inverted relative to each other, or is it interactions between chromosome arms of one haplotype?

Response: Yes, Fig S1 displayed the Hi-C heatmap of chromatin fibers from both haploid genomes. The X pattern you mentioned represents the interactions between chromosome arms of the two haplotypes, rather than a pair of chromosomes inverted relative to each other. Such interactions are common as it reveals the organization and physical contacts of chromatin in three-dimensional space. The actual chromosome boundaries you mentioned have been marked in the figure (Fig S1). The X pattern in the Hi-C heatmap is visualized solely to represent the interaction between the two sets of chromatin fibers. To avoid ambiguity and better display the karyotypes of the two haplotypes, we adjusted the sequences of the two haplotype chromosomes to make them initiate in the same direction, which could be seen in the revised Fig S1.

The parallel MP Chr5 pattern makes me think the former, but I initially assumed it was one haplotype.

Response: For the Chr5, the Hi-C interaction map reflected the interactions between the two haplotypes. In our revised version, the orientation of all pairs of haploid chromosomes have been adjusted to a better illustration of the interaction between the two haplotypes (Fig S1).

Where are the X and Y on the dot plots (Fig S7/S8)? Have both been moved into H1? It would also be clearer if Fig S3 used chromosome labels not scaffold numbers. How were the X and Y identified?

Response: In the original versions of Fig S7 and S8, the sex chromosomes were not depicted, and

it was not the case that the sex chromosomes were all moved to H1 assembly. In the revised revision, we have incorporated the visualization of synteny between the sex chromosomes, which can be seen in Fig S19 (c) and (d). In this study, the assembled regions related to the Y chromosome of the Chinese pangolin and the Malayan pangolin are approximately 6Mb in length, and a complete Y chromosome assembly was not achieved, largely due to the limitations of sequencing and assembly technologies, as well as the highly repetitive and complex structure of the Y chromosome. There are relatively few homologous regions between the X and Y chromosomes, and the collinearity regions that can be visualized are also relatively limited. Therefore, the sex chromosomes were not depicted in the original FigS7 and S8. In the revised version, we have included the collinearity between the sex chromosomes in FigS19 (c) and (d).

For clarity, we updated Fig S3 by using chromosome labels instead of scaffold numbers in the revised manuscript.

We confirmed the X chromosome and Y-linked regions by two methods: 1) The presence of the SRY gene was used to identify Y-linked regions; 2) Based on the truth that the sequencing depth and coverage of the sex chromosomes is half of that of the autosomes for a male pangolin individual, as shown in Figure S3. For clarity, we have included a description of the identification of sex chromosomes in the methods section.

I think Fig 1C tries to present some of these data too but it is really unclear, being both too small and lacking any scales/legends.

Response: Thank you for your suggestion. We have revised Figure 1C by adjusting the panel size and adding legends and scale to clearly present the genomic information of the two pangolin genomes. Additionally, we have moved this figure to the appendix as Fig S19(a).

Given the questions over karyotype and the risk of scaffolding errors, I would like to see a deeper analysis of the proposed sites of fusion/fission.

Response: Thank you for your comments. To minimize the likelihood of false positive fusions and fissions that may arise from errors during scaffolding, we have conducted validation for all fission and fusions by aligning contigs to the genome assembly. This additional step ensures that the proposed fusion and fission sites are supported by more evidence, thereby preventing potential inaccuracies in scaffolding. The verification results are detailed as below (Response figure 5):

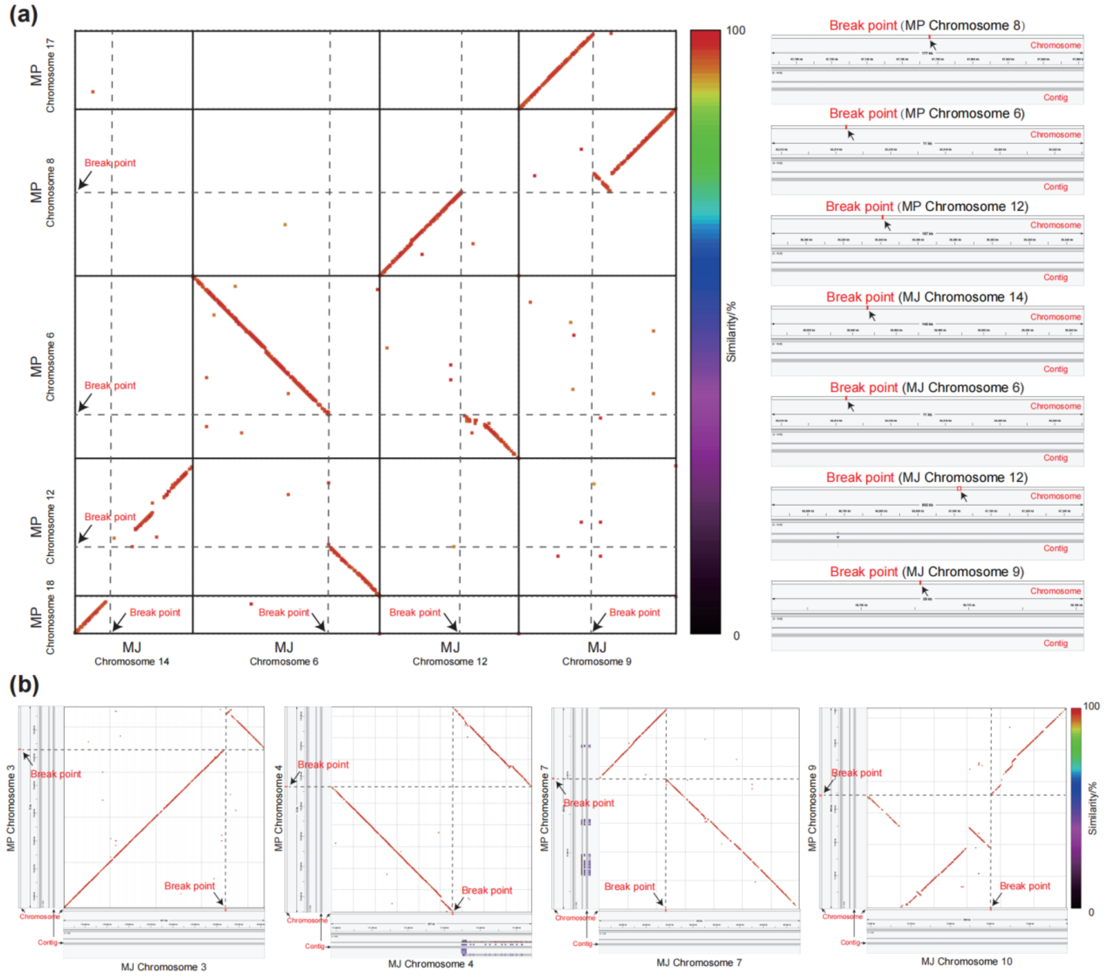

Response figure 5. Validation of the fusion, fission and main breakpoints between Chinese pangolin and Malayan pangolin genomes by aligning contigs to the two reference genomes. (a) Verification of fusion and fission positions. The left panel represents the synteny relationship, the left panel shows the support of contigs against the breakpoints of the fission and fusion events, the gray bar represents the contig and the red dot represents the breakpoint. (b) Verification of the other four significant breakpoints. In the IGV screenshot, the red mark represents the relative position of the SV breakpoint in the chromosome, and the gray band represents the contig at this position.

B. MINOR COMMENTS

Q18. There are a lot of typos. Most of these just impact readability, but some make the message unclear.

Response: Thank you for pointing out this question. We have reviewed and corrected these typos. Additionally, we invited a native speaker to polish the language throughout the manuscript to ensure clarity and improve readability.

Q19. I found the use of MJ, MP and CP to be a bit confusing at first. (MP could be Malayan Pangolin vs CP, but it isnt.) Can the MP/CP for the Chinese Pangolin be made consistent? Mjav and Mpen might be clearer. Likewise, it would be good to be consistent with the ordering and colours used for the species and populations when presented in tables and figures.

Response: Thank you for this comment. Yes, we agree with your suggestion, the Mjav and Mpen

are clearer, and we changed the population name in the revised manuscript as the Response Table 1. We have also reviewed our manuscript and ensure that the ordering and colors were consistent throughout all tables and figures.

Species	Revised group information	previous population information
Malayan pangolin (MJ)	MjavA	MJ2
	MjavB	MJ1
Chinese pangolin (MP)	MpenA	CPA
	MpenB	CPB
	MpenC	CPC

Q20. Page 4: "the most promising strategies for genome assembling" - This is combining long-read and HiC data. Whether ONT or PacBio is better is subject to debate.

Response: Yes, we agree. It is debated that whether ONT or PacBio is the better technology for long read sequencing, and both of the two sequencing technologies are among the best strategies for genome assembly at present. Therefore, we used 'one of' in the manuscript. Thank you! Line 94.

Q21. L214: " Among the three Chinese pangolin populations, the inbreeding of the CPA and CPB populations were comparable (CPA: 0.18±0.036; CPB: 0.17±0.039), but much worse than the CPC population (FROH=0.06±0.005) (Fig. 3A and 3E, Supplementary Table S26)." Does this relate to population size and genetic diversity? The heterozygosity of the reference genomes looks low from the Merqury kmer plots. Are they representation of the population heterozygosity?

Response: Thank you for this question. The ROH fragments could well reflect the inbreeding level in a population. In general, small populations are more tend to occur the inbreeding, and an inbred population often presents low genetic diversity (π) and small population size. If a population is large enough and under the normal mating, it is almost impossible to present severe inbreeding. However, we have to keep in mind that, the inbreeding will not directly cause the low genetic diversity, but could make the genome homolyzed, and the homolyzed genome will further expose the recessive deleterious mutations, and some of these exposed deleterious mutations will cause severe consequences, such as death before mature, reproductivity problem or malformation….. Therefore, the loss of effective individuals in a population will take the genetic diversity away, then we can often see a low genetic diversity in an inbreeding population. But this is not always true, it is a very complicated thing if we discuss it as a specific issue.

The low heterozygosity showed in the Merqury kmer plots of the reference genome could reflect the genetic diversity of a single individual (usually can represent this species), but couldn't represent the population level genetic diversity. The nucleotide diversity (π) is a genetic parameter that could well reflect the population level genetic diversity.

Q22. L218: "Noteworthy, the FROH of the Taiwan individual is much higher than all other individuals in the MP population (FROH=0.54) (Fig. 3C)". There is no MP population. There are three. Confusing. Why is this noteworthy?

Response: Thank you. The MP population we mentioned in this manuscript represents the whole Chinese pangolin populations, including the CPA (MpenA), CPB (MpenB) and CPC (MpenC) populations. We mentioned the "MP" population in several places in the manuscript. To avoid the misunderstanding and make the manuscript more readable, we changed the "MP population" to the "the three Chinese pangolin populations" throughout the manuscript. Here we say "Noteworthy" is not because of the high inbreeding level in this individual, but because almost all the ROH fragments in this individual restricted to less than 1Mb (benefiting from the detailed distribution map of ROH fragments we detected in this study), which is not a normal thing for endangered species, but is underestimated in the study of Hu et al (please refer **Q7**).

Q23. Fig 3. Please make the colours and ordering match. Fig 3C is not clear. The size mapping is superfluous as it is just the y-axis. Would be better to use symbols/fill to more clearly differentiate the populations/species. It is not clear from this plot why Taiwan is singled out.

Response: Thank you for this reminder. We have revised Fig 3C to make the colors and ordering match each other. We removed the size mapping, and we now used the different colors to represent different populations. Notably, the F_{ROH} for the Taiwan individual is significantly higher compared to all other individuals in the Chinese pangolin population, then we showed the Taiwan individual to help reader to trace this individual.

Q24. L390. "The Chinese pangolin and Malayan pangolin used for genome assembly were wild rescued individuals by the Guangdong Wildlife Rescue Center. " As these are proposed reference genomes, is it possible to proved any photos or metadata to validate the species IDs? What populations are they from?

Response: Thank you to point out this issue. We have discussed with people in Guangdong Wildlife Rescue Center for this issue. Unfortunately, the geographical origin of these two individuals used in this study is not very clear, as both individuals were confiscated from criminals, and these individuals have been passed around many times, making it difficult to track the detailed geographical location for these two individuals.

Q25. L410: "Then, the Hi-C sequencing reads are mapped to the primary genomes by the mem algorithm of Burrows-Wheeler Aligner (BWA, v0.7.17) [66, 67], while Hi-C data quality control was conducted by the Juicer[68] (v1.5). The 3d-DNA pipeline (v190716) was finally used to concatenate and review the scaffolds to chromosome-scale genomes[69]. " How were phased assemblies produced if only the primary genome was scaffolded? Why were both haplotypes not scaffolded independently? I am confused how you can comment on haplotype synteny if not. Was manual editing of the scaffolding performed as part of the assembly curation?

Response: Thank you for this comment. The Hi-C sequencing data was used for two times in the genome assembly: **1) Phasing**: we could feed the hifiasm software with both the HiFi sequencing data and Hi-C sequencing data, and then the hifiasm software will output three genomes at the same time: the hybrid genome, and the two haploid genomes (for a diploid species). The hifiasm software could automatically phase the hybrid genome into two haploid genomes, even we have no

sequencing data from the trio samples. **2) Concatenating:** the Hi-C data will be again used for contaminated the scaffolds generated in the previous step to the chromosome-scale genome. We have revised the description for the assembly of the genome to make it clearer (Lines 531-534). Yes, we will manually adjust the genome according to the Hi-C interacting map.

Q26. L460. What does “SNPs that were missed” mean? No coverage? Filtered?

Response: Thank you. In the VCF (Variant Call Format) file, a genotype at a specific genome position of all individuals will be listed in a row in the VCF file (Response figure 6), and if no sequencing reads could well support the base calling in this specific genome position in an individual (due to low sequencing coverage, low quality sequencing data, or failed base calling in complex genome regions……), we identified this as a missing genotype at this specific genome position for this individual. If the missing rate exceed 20% at a specific genome position, this row will be deleted from the VCF file. For example, there are 100 individuals in a VCF file, and the genotype at a genome position should be successfully called in more than 80 individuals, or this site will be filtered from the VCF file.

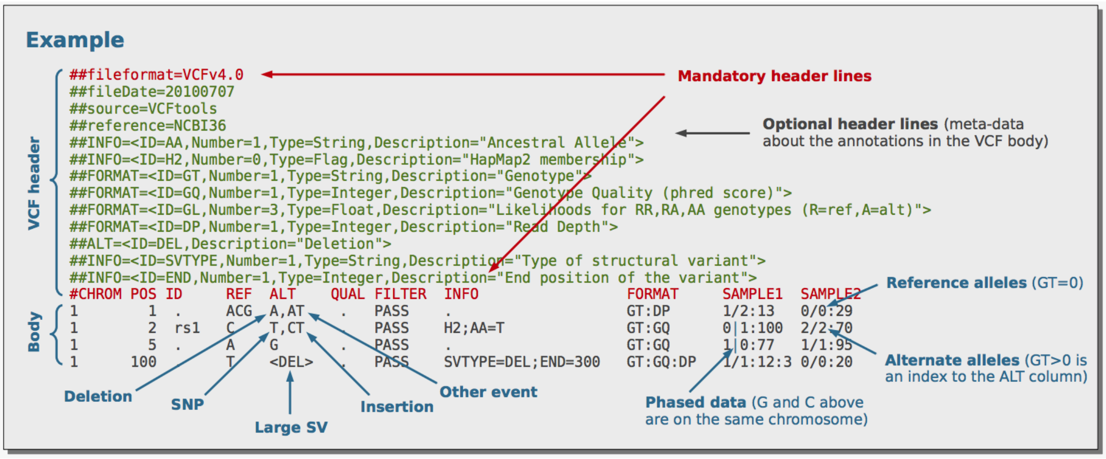

Response figure 6. A normal VCF file.

Reviewer #3: This manuscript by Lan et al. presents high-quality, haplotype-resolved, and chromosome-scale reference genomes for Chinese and Malayan pangolins, providing crucial insights into genetic diversity, inbreeding, and extinction risks. The study highlights moderate inbreeding in most populations, identifies a highly inbred Malayan pangolin population needing targeted conservation, and detects mild genetic purging. These findings contribute valuable genomic resources and new perspectives for pangolin conservation efforts. In general, the manuscript has clear logic, detailed description and comprehensive data analysis. Therefore, I recommend a minor revision before acceptance. There are some minor comments and suggestions as following：

Q27. 1. The authors used abbreviation of the Latin names for the Malayan pangolin (MJ) and Chinese pangolin (MP) in the third paragraph. However, they changed the way of abbreviation for the Chinese pangolin populations (CPA, CPB and CPC) later on. It's not a problem, but a little bit confusing for the readers to catch up the logic of describing.

Response: Thank you for pointing out this question. Yes, we agree. We used the CPA, CPB and CPC to keep consistence with the published papers, but as you mentioned, this will make the manuscript hard to be follow. Therefore, we changed the abbreviation name of the Chinese pangolin and

Malayan pangolin to make them more readable (Response Table 1).

Species	Revised group information	previous population information
Malayan pangolin (MJ)	MjavA	MJ2
	MjavB	MJ1
Chinese pangolin (MP)	MpenA	CPA
	MpenB	CPB
	MpenC	CPC

Q28. Lines 74-75, "The MJ1 population was …… diverged from the MJ1 population"???

Response: Thank you for pointing out this issue. We have corrected the description error in Lines 84.

Q29. 3. In Figure 1A, the legend is inconsistent with what have been shown in the figure. I suppose the authors used wrong colors to indicate CPA and CPC populations. Moreover, chromosome numbers, Hic_scaffold numbers in Figure 1C, Figure 1D, Figure S3 and Figure S5 are not in consistency. Please double check chr 7, chr 8, chr 20 and X chromosome in these figures.

Response: Thank you for your kind reminder. We have corrected the legend and the colors represent the population in Figure 1. In the original manuscript, Figure 1C shows the synteny between the two pangolin species, which was achieved by aligning and comparing the hybrid genomes of the two pangolin species. Figure 1D presents an analysis of the structural variation (SV) across the genomes of each pangolin species, specifically focusing on that between the two haplotype chromosomes. So, the numbering in these two figures is not consistent. We have carefully revised these figures to ensure that the chromosome and Hic_scaffold numbers are consistent across all mentioned figures. The revised figures can be found in Supplementary Figure 19a, Figure 1B, Supplementary Figure 3, and Supplementary Figure 15.

Q30. Usually, the mean depth of sequence data has an impact on the genomics analyses. In this study, the sequencing depths of the samples provided by the authors were basically between 10-20-fold, but depths of the published data of the population CPC and most of the population CPB were significantly higher than the author's own data (20-40-fold). I suggest that differences in sequencing depth should be considered when comparing the genetic characteristics of different MP populations.

Response: Thank you for this comment.

1. Yes, we agree, the sequencing depth will influence the detection of variants and then influence the following bioinformatic analysis based on these variants. We investigated how the sequencing depth impact the detection rate and accuracy for genome-wide SNPs by investigating the published papers. Current research suggests that a depth of 10X represents an optimal practical target for achieving comprehensive coverage and for the reliable discovery of

variants, ensuring that the sequencing covers more than 99% of the genome[21]. This depth not only ensures broad genomic analysis but also maintains a balance between cost and information yield. As illustrated in the Response figure 7, the sequencing coverage increased with the sequencing depth, though not linearly. There was a sharp rise in coverage from 60% to 95% as depth increased from 1X to 4X. After this, coverage increased more gradually from 95% to 99% as depth increased from 5X to 10X, stabilizing at around 99% at a depth of 10X. This level of coverage closely approximates the results obtained at sequencing depths exceeding 10X, where the genome coverage exceeds 99%. Therefore, 10X seems to be an appropriate depth for accurate genotyping and population genetic studies, and may be no impact on the comparison of genetic characteristics?

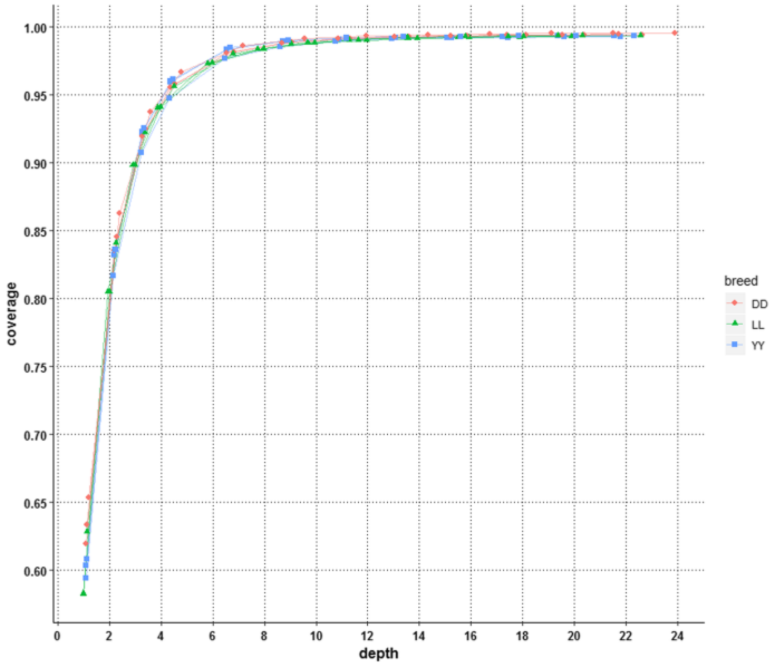

Response figure 7. Coverage with sequencing depth for each downsampled genome. Whole-genome coverage as a function of sequencing depth for each downsampled genome[21].

2. To further confirm whether the different sequencing depth will influence the analysis in this study, we randomly extract reads from individuals of the three Chinese pangolin populations, and to control that all individuals reached a sequencing coverage of 10X and then re-do the analysis we performed in this study. We finally found that the results we obtained based on the 10X data are very consistent with that we calculated in this manuscript with 10 to 40X data (Response figure 8-10).

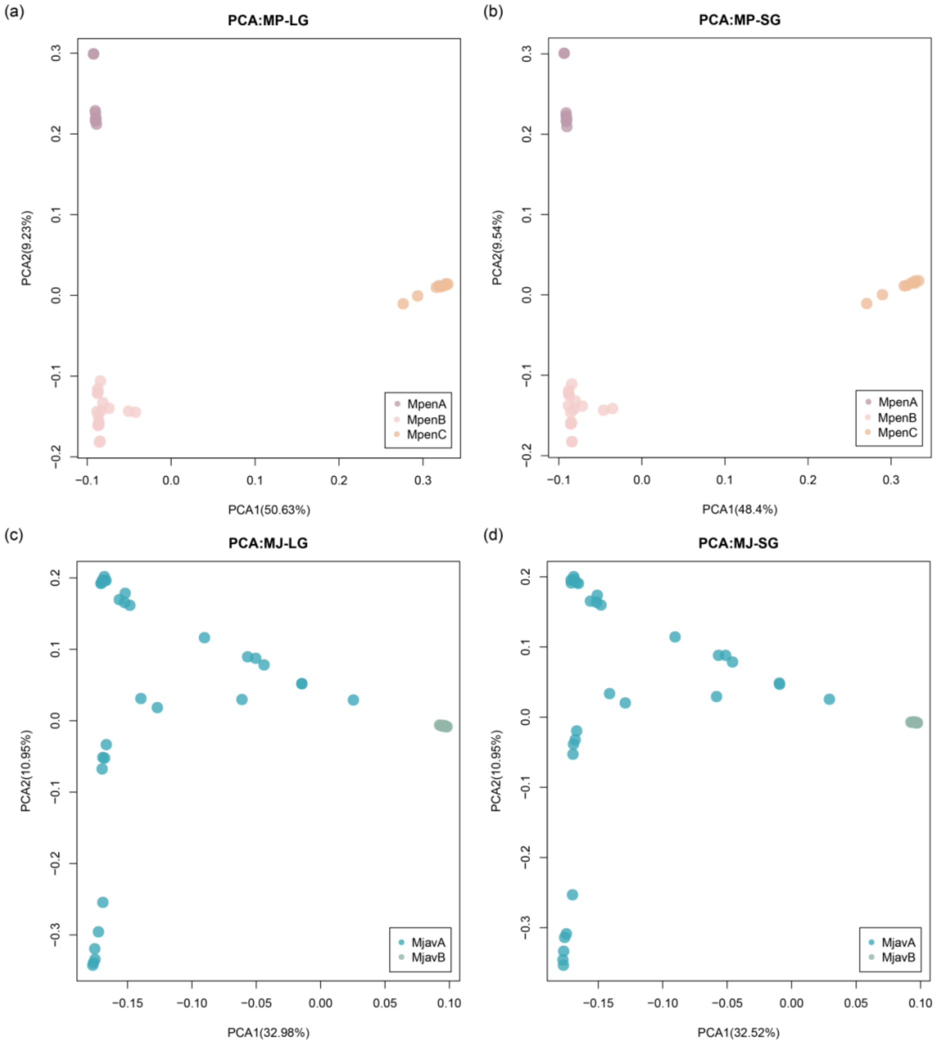

Response figure 8. PCA analysis of Malayan pangolin and Chinese pangolin populations based on SG(short-read assembled genome) and LG(long-read HiFi assembled genome) with randomly extracted 10X sequencing reads.

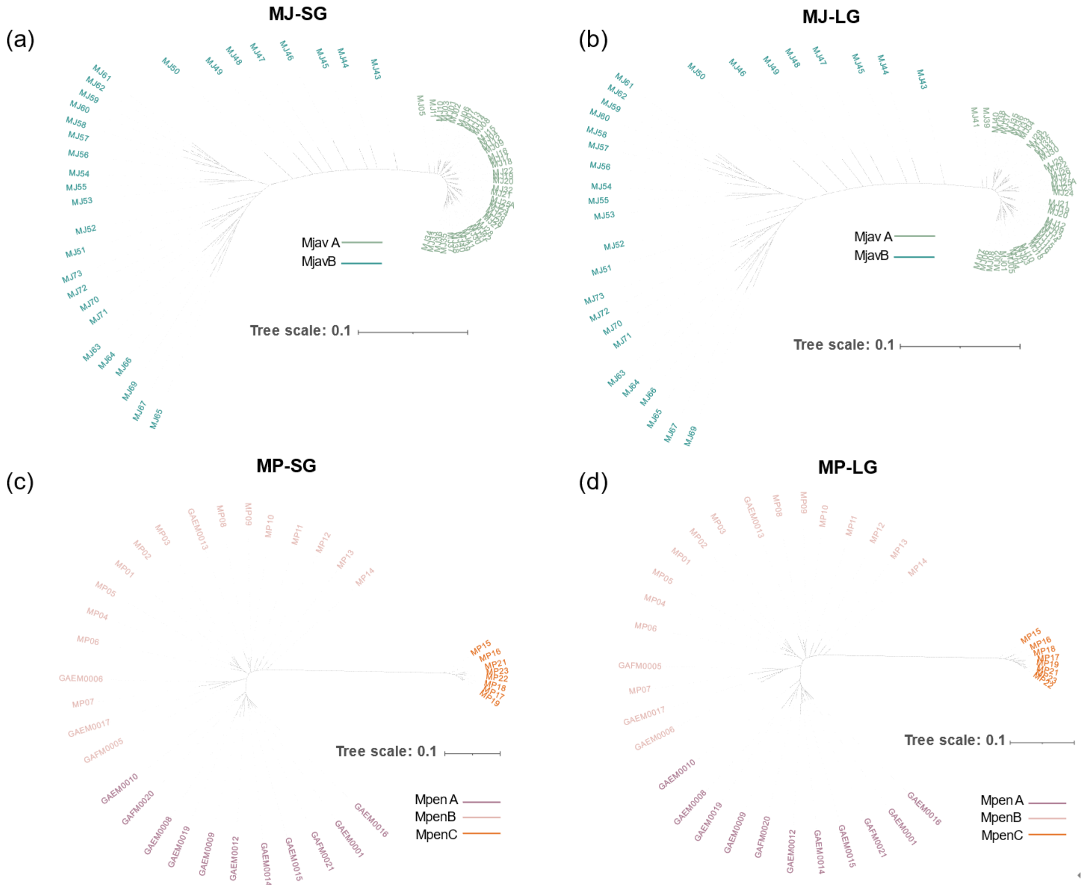

Response figure 9. Phylogenetic tree of Malayan pangolin and Chinese pangolin populations based on SG and LG with randomly extracted 10X sequencing reads.

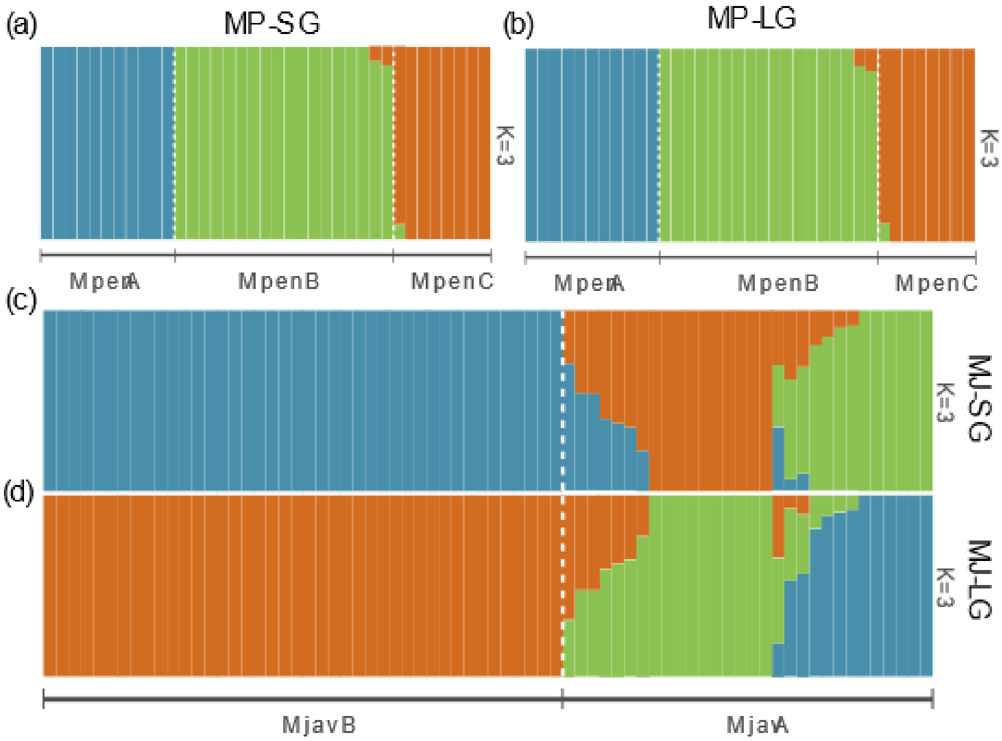

Response figure 10. Admixture analysis of Malayan pangolin and Chinese pangolin populations based on SG and LG with randomly extracted 10X sequencing reads.

3. We also tested that how the sequencing coverage could influence the detection of ROH across the genome by simulating sequencing data with different sequencing depth. We found that the F_{ROH} that could be detected will be very close to the true state. Therefore, we conclude that a minimal sequencing depth of 10X could compare with that of even 40X (Response figure 11).

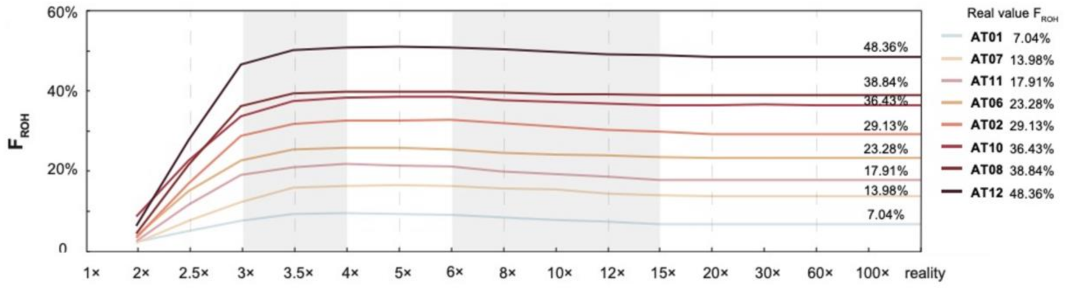

Response figure 11. The impact of sequencing depth on the detection of ROH across the genome. The inbreeding coefficient (F_{ROH}) as the function of different sequencing depths. The legend on the far right showed the real F_{ROH} value of the simulated individual genome.

Q31. In the result section (lines 218-221), the authors described that "the FROH of the Taiwan individuals was higher than other individuals in the MP population…… the FROH varies greatly among individuals in the CPA or CPB population". I am expecting relevant discussion on these results, which I have not found.

Response: Thank you to point out this issue. Yes, the Taiwan individual is very special among the Chinese pangolin population. The F_{ROH} in the Taiwan individual is also very strange, because this

individual presented the highest F_{ROH} value but almost all ROH fragments (>99%) were less than 1Mb, which is not consistent with the value calculated by previous study (please refer **Q12**), and is not a very normal situation in endangered species. We have discussed the Taiwan individual in the revised manuscript in both result and discussion part. Lines 466-476.

Q32. Lines 374 and downstream contents: with regard to the conservation, there is a new publication by Wei et al., 2024 in SCIENCE CHINA Life Sciences. I recommend the authors cite this paper to see if they agree to each other in terms of MP conservation.

Response: Thank you for this reminder. The study of Wei et al., mainly explored the genetic structure and population status of Chinese pangolin populations, including genetic diversity, inbreeding level, gene flow, and mutational load. The study has conducted a comprehensive genomic analysis of 94 individuals across three populations. The research indicates that while historical climate change has played a role in shaping these populations, it is the recent human activities that have precipitously reduced the numbers pangolin populations, leading to increased inbreeding.

We did not include the samples of Wei et al, because: 1) at the outset and during the submission of this study, the aforementioned research had not yet been published, therefore it was not included in the original version for comparison and discussion. 2) Because the data is not synchronous released with the publication of the paper, we were unable to directly compare our results with their study at that time. 3) There is still ongoing debate regarding the population structure of the Chinese pangolin, and we are preparing a manuscript covering much more samples to settle down this controversy. 4) We need to reanalyze the entire article if we now added their samples in the current study, however, this could not influence much about the main conclusions of the manuscript. Therefore, this study did not prepare extensive comparison and discussions with the research of Wei et al, but we have cited this paper and make a brief comparison with their study in the revised manuscript (Line 71). We appreciate of the reviewer's understanding.

Q33. Line 461: Please provide the accession number of reference genomes.

Response: Thank you. The accession number of reference genomes in this study are GCA_040802235.1 and GCA_040802205.1. And we have added this information in "Data Availability" section.

Q34. Figure S18, (a) should be "…based on the SG".

Response: Thank you for your attention to detail. We have modified the legend of Figure S7e.

Q35. English needs to be double checked to avoid mistakes. For example, in line 29, "with the high inbreeding and ….." , "the" should be deleted; in Line 328, "the two genetic parameters ……were……", "were" should be "are".

Response: Thank you for this comment. We have corrected all the descriptions mentioned above and also had the entire manuscript proofread by the native speaker. Line 30

1. Cheng, H., et al., *Haplotype-resolved de novo assembly using phased assembly graphs with hifiasm.* Nature methods, 2021. **18**(2): p. 170-175.
2. Rhie, A., et al., *Merqury: reference-free quality, completeness, and phasing assessment for genome assemblies.* Genome biology, 2020. **21**: p. 1-27.

3. Li, M., et al., *Chromosome-level genome assembly of Aquilaria yunnanensis.* Scientific Data, 2024. **11**(1): p. 790.
4. Fitz-Gibbon, S., et al., *Reference genome of California walnut, Juglans californica, and resemblance with other genomes in the order Fagales.* Journal of Heredity, 2023. **114**(5): p. 570-579.
5. Flack, N., et al., *Chromosome-level, nanopore-only genome and allele-specific DNA methylation of Pallas's cat, Otocolobus manul.* NAR Genomics and Bioinformatics, 2023. **5**(2): p. lqad033.
6. Kim, H.-s., et al., *KOREF_S1: phased, parental trio-binned Korean reference genome using long reads and Hi-C sequencing methods.* GigaScience, 2022. **11**: p. giac022.
7. Mead, A., et al., *The genome assembly of Island Oak (Quercus tomentella), a relictual island tree species.* Journal of Heredity, 2024. **115**(2): p. 221-229.
8. Wang, Q., et al., *Whole-genome resequencing of Chinese pangolins reveals a population structure and provides insights into their conservation.* Commun Biol, 2022. **5**(1): p. 821.
9. Hu, J.Y., et al., *Genomic consequences of population decline in critically endangered pangolins and their demographic histories.* Natl Sci Rev, 2020. **7**(4): p. 798-814.
10. Formenti, G., et al., *The era of reference genomes in conservation genomics.* Trends Ecol Evol, 2022. **37**(3): p. 197-202.
11. Zhang, L., et al., *Chromosome-scale genomes reveal genomic consequences of inbreeding in the South China tiger: A comparative study with the Amur tiger.* Mol Ecol Resour, 2022.
12. Yang, S., et al., *Genomic investigation of the Chinese alligator reveals wild-extinct genetic diversity and genomic consequences of their continuous decline.* Mol Ecol Resour, 2022.
13. Lan, T., et al., *Large-scale genome sequencing of giant pandas improves the understanding of population structure and future conservation initiatives.* Proceedings of the National Academy of Sciences, 2024. **121**(36).
14. Dussex, N., et al., *Purging and accumulation of genetic load in conservation.* Trends in Ecology & Evolution, 2023. **38**(10): p. 961-969.
15. Dussex, N., et al., *Purging and accumulation of genetic load in conservation.* Trends Ecol Evol, 2023. **38**(10): p. 961-969.
16. Ray-Chaudhuri, S., et al., *Chromosomes and the karyotype of the pangolin, Manis pentadactyla L.(Pholidota-mammalia).* Experientia, 1969. **25**: p. 1167-1168.
17. CHEN, Q., et al., *Studies on the mitotic chromosomes and meiotic synaptonemal complexes (SC) of Chinese pangolin (Manis pentadactyla).* Zoological Research, 1991. **12**(3): p. 299-304.
18. Wu, S.-H., et al., *Cytogenetic analysis of the Formosan pangolin, Manis pentadactyla pentadactyla (Mammalia: Pholidota).* Zoological Studies, 2007. **46**(4): p. 389-396.
19. NV, A., *Chromosomes and karyotype of the Indian pangolin, Manis crassicaudata Gray (Pholidota-Mammalia).* Cytologia, 2000. **65**(4): p. 379-382.
20. Cheng, H., et al., *Haplotype-resolved assembly of diploid genomes without parental data.* Nature Biotechnology, 2022. **40**(9): p. 1332-1335.
21. Jiang, Y., et al., *Optimal sequencing depth design for whole genome re-sequencing in pigs.* Bmc Bioinformatics, 2019. **20**: p. 1-12.
