# Supplementary material for: Enhancing inbreeding estimation and global conservation insights through chromosome-level assemblies of the Chinese and Malayan pangolin
Source: Gigascience. 2025 Feb 14;14:giaf003. doi: 10.1093/gigascience/giaf003 (PMC11825179; doi:10.1093/gigascience/giaf003)
Supplement: giaf003_GIGA-D-24-00182_Revision_2 [file giaf003_giga-d-24-00182_revision_2.pdf]

GigaScience

Enhancing inbreeding estimation and global conservation insights through chromosome-level assemblies of the Chinese and Malayan pangolin

--Manuscript Draft--

**Manuscript Number:**	GIGA-D-24-00182R2
**Full Title:**	Enhancing inbreeding estimation and global conservation insights through chromosome-level assemblies of the Chinese and Malayan pangolin
**Article Type:**	Research
**Funding Information:**	National Key Program of Research and Development, Ministry of Science and Technology (No. 2022YFF1301500) — Not applicable<br>the Guangdong Provincial Key Laboratory of Genome Read and Write (No. 2017B030301011) — Not applicable<br>Start-up Scientific Foundation of Northeast Forestry University (60201524043) — Prof Tianming Lan
**Abstract:**	A high-quality reference genome coupled with resequencing data is a promising strategy to address issues in conservation genomics. This has greatly enhanced the development of conservation plans for endangered species. Pangolins are fascinating animals with a variety of unique features. Unfortunately, they are the most trafficked wild animal in the world. In this study, we assembled a chromosome-scale genome with HiFi long reads and Hi-C short reads for the Chinese and Malayan pangolin and provided two new representative reference genomes for the pangolin species. We found a great improvement in the evaluation of genetic diversity and inbreeding based on these high-quality genomes and obtained different results for the detection of genome-wide extinction risks compared with genomes assembled using short reads. Moderate inbreeding and genetic diversity were reverified in these two pangolin species, except for one Malayan pangolin population with high inbreeding and low genetic diversity. Moreover, we identified a much higher inbreeding level (FROH=0.54) in the Chinese pangolin individual from Taiwan Province compared with that from mainland China, but more than 99.6% runs of homozygosity (ROH) fragments were restricted to less than 1Mb, indicating that the high FROH in Taiwan Chinese pangolins may have accumulated from historical inbreeding events. Furthermore, our study is the first to detect relatively mild genetic purging in pangolin populations. These two high-quality reference genomes will provide valuable genetic resources for future studies and contribute to the protection and conservation of pangolins.
**Corresponding Author:**	Haimeng Li<br>Northeast Forestry University<br>Harbin, CHINA
**Corresponding Author Secondary Information:**	
**Corresponding Author's Institution:**	Northeast Forestry University
**Corresponding Author's Secondary Institution:**	
**First Author:**	Tianming Lan
**First Author Secondary Information:**	
**Order of Authors:**	Tianming Lan<br>Yinping Tian<br>Minhui Shi<br>Boyang Liu<br>Yu Lin

Powered by Editorial Manager® and ProduXion Manager® from Aries Systems Corporation

Yanling Xia
Yue Ma
Sahu Sunil Kumar
Qing Wang
Jun Li
Jin Chen
Fanghui Hou
Chuanling Yin
Kai Wang
Yuan Fu
Tengcheng Que
Wenjian Liu
Huan Liu
Haimeng Li
Yan Hua

Order of Authors Secondary Information:

Response to Reviewers:

Reviewer #2: The authors have engaged positively and comprehensively with reviewer feedback, and I think the manuscript is a lot clearer as a consequence. I only have a few minor outstanding questions/comments.
MINOR COMMENTS
1.I suggest stressing the chromosome-level nature of the assembly in the title.
Response：Thank you. To address your suggestion, we have revised the title of our manuscript to better reflect the chromosome-level nature of our assembly. The new title is:
“Enhancing inbreeding estimation and global conservation insights through chromosome-level assemblies of the Chinese and Malayan pangolin”
2.Abstract - mention the HiC as well as HiFi reads.
Response：We have revised the description of Abstract in Lines 25-26.
3.L97: "all genomes (2 diploid assemblies and 4 haploid assemblies)" - are these not the same thing? Surely, the "diploid" assemblies are just the combined pair of haploid assemblies?
Response：Thank you. When we refer to "2 diploid assemblies and 4 haploid assemblies," we are indicating two different types of genome assemblies. Diploid assemblies are not simply a combined pair of haploid assemblies.
The diploid genome is the genome assembly without genome phasing, is a hybrid genome mixed with the genetic information from both parents, which represents the most common form of genome assembly. The haploid assembly, meticulously constructed with HiFi long reads and Hi-C data, is designed to distinguish the haplotype of each parent[1]. Therefore, the most important process for the assembly of the haploid genome is the phasing process to separate the genome segment from each parent. To make it clearer, I prepared a picture to show this:

Response figure 1. Schematic diagram for the genomic sequence assembly
4.In Table 1, it would be useful to have the total assembly size and sequencing depth. Given the comparison with the short-read genomes, it would be informative to have their stats in the table too, which would really show where the improvements were made.
Response：Thank you for your suggestion to enhance Table 1 with additional statistical information. The current version of the Table 1 has been modified based on the previous reviewer's comments, and the comparison with other genomes has also been displayed in Table S19 following the reviewer's suggestions.
We have updated Table 1 to incorporate the total assembly size and sequencing depth.
5.I don't think it's strictly true to say that 10 PacBio HiFi reads ensures a reliable

Powered by Editorial Manager® and ProduXion Manager® from Aries Systems Corporation

assembly. It could still be false duplications or contamination. 10 reads might also be a bit strict, depending on the depth of coverage. I can see what the authors are trying to do here, but wonder whether a slightly different descriptor could/should be found.
Response：The definition and calculation of the reliable assembly genome regions in the manuscript followed the previously published research[2]. Based on your suggestions, we have modified the calculation method for the reliable blocks. We consider the regions in the genome that are supported by two sequencing technologies (Hi-C and HiFi) as reliable blocks[3]. This method is more stringent, and we believe its reliability will meet the requirements of the analysis. In lines 107 and 109-110.
6.Figure 1. What are the colours in panel B? The details of Panel B are a bit lost. Having a full-width landscape plot with a different colour per chromosome might be clearer.
Response：Thanks for your comments. We have assigned a unique color to each chromosome.

Response figure 2. Introduction to the species distribution and chromosome synteny of the Chinese and Malayan pangolins. (A) The distribution area and sampling sites of the Chinese and Malayan pangolins in this study. The circles represent sampling sites of the Chinese pangolins reported by Wang et al.[4] Samples without detailed locations are not shown on the map. (B) The chromosome-scale synteny analysis between the Malayan pangolin and Chinese pangolin genomes.
7.L145-150. The LG vs SG analysis is much clearer now, but with the ROH analysis I was still left feeling a little unsure of the deeper implications. Unless I have misunderstood, ROH is a relative measure of inbreeding and therefore the absolute changes are not of that much interest. Rather than ROH being bigger with LG vs SG (as expected), is the bigger finding not the apparent reversal in the relative recent inbreeding of MJ and MP as marked by ROH > 1Mb in LG (MP>MJ) compared to SG (MJ>MP)?
Response：Thank you for this comment. I am sorry that I could not well understand the “relative” you mentioned in the comment. I think the ROH is a direct measurement for the inbreeding, a longer ROH fragment can reflect a more recent inbreeding event, and the time of the inbreeding occurred could be also inferred with the known recombination rate and generation time for a species. The Froh could reflect the general inbreeding for a population, and the Froh for different length of ROHs could help track the inbreeding history of a species. The LG genome could contribute more for the accurately dating back of recent inbreeding history, which we think is the most important thing for the LG genome.
Yes, as you mentioned, one of the findings in this study is the reversal in the recent inbreeding between the MJ (Malayan Pangolin) and MP (Chinese Pangolin) population. This is indeed marked by the presence of ROH segments greater than 1Mb, where in the LG, MJ exhibits a higher frequency of such segments compared to MP (MJ>MP). Conversely, in the SG, MP has a more of these segments (MP>MJ). However, it is difficult to compare inbreeding between different species, because there are too many factors could influence the detection of ROH for different species, such as the different sequencing quality and depth, the different quality of reference genome, the different recombination rate, the different mutation rate, and so forth. Therefore, we did not discuss much about this difference. But we agree with you, this reversal is also an important observation that underscores the superior capability of high-quality reference genomes assembled from long-read sequences in uncovering the real level of inbreeding, when compared to those assembled from short-read sequences. Then, the use of long-read assemblies is crucial to avoid erroneous interpretations, such as the misconception that the Chinese pangolin is more inbred than the Malayan pangolin in the recent past, as might be suggested by findings based on short-read assemblies. By leveraging the detailed insights provided by long-read genomes, we can ensure that our conclusions regarding the inbreeding status of these species are both genuine and reliable. Following your comment, we have included additional discussion on this significant finding. In lines 310-313
8.I found the "Genetic Purging" section a little confusing and hard to follow. L253-4: "which indicated that the genetic purging in the MjavA and MjavB populations was less efficient, but stronger in the MjavA population" - less efficient but stronger than what? Can this section be simplified a little, perhaps with each result introduced in terms of what that particular result is testing and how it related to relative(/absolute?) genetic purging.
Response：Thank you for your comments regarding the "Genetic Purging" section of

our manuscript. As your suggestion, we have rephrased the whole paragraph to make it clearer, and we deleted some confused and meaningless analysis from the revised manuscript to make this section simplified. Here we showed the revised content: In the Malayan pangolin populations, the ROHf of highly deleterious mutations (LoF) was significantly lower than the nonROHf in both the MjavA and MjavB population (Fig. 5C). This denoted that many large-effect deleterious alleles (e.g. LoF) still existed but are masked in nonROH regions in both the two populations, indicating that the purging was less efficient to remove recessive deleterious mutations across the whole genome. Further inspection we found the difference between the ROHf and nonROHf was smaller in the MjavA population than that in the MjavB population (Fig. 5D and Supplementary Fig. S11a-S11c), indicating that the higher-level of inbreeding in the MjavA population may have facilitated the exposing of more recessive deleterious alleles to be homozygous, which has resulted in a more efficient purging of deleterious mutations in this population than in the MjavB population [5, 6].

9.Figure 5. Why are C&E and D&F visualised differently? I am not familiar with dot plots (that are not synteny plots) - the legend should explain what the different parts of the plot (lines and bubbles) mean. The boxes and whiskers should also be explained in the box plots. (i.e. what range do they represent.)

Response: Thank you for your attention to the details in our figures.

In our study, Figures 5C and 5E compared the frequency of LoF mutations (LoF/synonymous mutations) in ROH (ROHf) to that of non-ROH (nonROHf) regions within populations. Box plots (Figures 5D and 5F) was set to compare the ratio of ROHf to nonROHf of LoF mutations in the population to further clarify the differences between the LoF frequency in ROH and that in nonROH regions.

To further enhance the clarity and comprehension of this figures, we have expanded upon the legend for Figure 5 in the revised manuscript. This includes a detailed elucidation of the elements within both the dot plots and box plots. In lines 280-285.

Response figure 3. (C) Dot plot showing the occurrence of LoF mutations in the two Malayan pangolin populations calculated as the ratio of the number of the mutational load to synonymous mutations in the ROH regions (ROHf) or nonROH (nonROHf) regions across the genome. Each point signifies the LoF frequency of an individual. The large dots represent the average LoF frequency for the population, while the lines indicate the standard deviation range around the mean. (D) The ratio of ROHf to nonROHf for the LoF in the two Malayan pangolin populations. The box represents the interquartile range (IQR), stretching from the first quartile (Q1) to the third quartile (Q3). The line that bisects the box indicates the median value. The whiskers extended from the box to show the variability of the data, typically reaching to the minimum and maximum values that fall within 1.5 times of the IQR from Q1 and Q3, respectively.

10.L385: Typo: "readsand"

Response：Thank you for the correction, it has been amended. In line 397

11.L469-470. Why was there the requirement for ancestral alleles to be the "predominant" allele? (This should be "Major" allele, I think.) I can see no reason why an ancestral allele could not be the minor allele.

Response：Yes, you are right here we mean the major allele, and we changed in the manuscript to avoid further misunderstanding. In line 482

Ancestral alleles are those that persist in their initial state, while derived alleles are the result of mutations that have diverged from this initial state. Under the neutral theory, it is common to consider the major allele of population as the ancestral one, especially conserved genomic regions across species[7, 8]. This is because the major allele, is more likely to be the one that has been preserved through evolutionary history, unless there is strong evidence of positive selection favoring a derived allele[9]. Another common approach to identifying ancestral alleles is by looking at the alleles present in closely related species[10]. This method is based on the assumption that mutations occur only once at a given position and that they happened after the species divergence[8]. Alleles that are shared among multiple species are more likely to be ancestral, as they have not diverged from the initial state. This shared state often corresponds to the major allele in the population [11].

While it is known that major alleles are not always ancestral, using major or common alleles as a proxy for ancestral alleles is a practical approach in the absence of a comprehensive list of ancestral alleles. Furthermore, we have integrated additional evidence from closely related species to further validate and refine our identification of ancestral alleles, ensuring greater accuracy in our determination. This inference method is widely used in genomics studies, including research on pangolins[8, 12-14].

	12.L524. Why was k=17 selected? This seems very small for a vertebrate. There are only 17.2 billion unique DNA kmers, so the chance of random convergence in a >2Gbp genome is quite high. k=31 is the de facto standard, although k=21 or k=27 are also used quite often.<br>Response：Thank you for your comment on the choice of k-mer size in our analysis. We have updated our analysis by using k-mer size of 31 and included the results in the Fig S2.<br>13.L569: Typo: "derive"<br>Response：Thank you for your reminder. We have corrected it. In lines 578 and 580.<br><br>1.Cheng H, Concepcion GT, Feng X, Zhang H and Li H. Haplotype-resolved de novo assembly using phased assembly graphs with hifiasm. Nature methods. 2021;18 2:170-5.<br>2.Qi W, Lim Y-W, Patrignani A, Schläpfer P, Bratus-Neuenschwander A, Grüter S, et al. The haplotype-resolved chromosome pairs of a heterozygous diploid African cassava cultivar reveal novel pan-genome and allele-specific transcriptome features. GigaScience. 2022;11:giac028.<br>3.Rhie A, McCarthy SA, Fedrigo O, Damas J, Formenti G, Koren S, et al. Towards complete and error-free genome assemblies of all vertebrate species. Nature. 2021;592 7856:737-46.<br>4.Wang Q, Lan T, Li H, Sahu SK, Shi M, Zhu Y, et al. Whole-genome resequencing of Chinese pangolins reveals a population structure and provides insights into their conservation. Commun Biol. 2022;5 1:821. doi:10.1038/s42003-022-03757-3.<br>5.Nicolas D, Tom vdV, Hernán E. M, Christopher W. W, David D-d-M, Johanna vS, et al. Population genomics of the critically endangered kākāpō. Cell Genomics. 2021;1 1:100002. doi:10.1016/j.xgen.2021.100002.<br>6.Xue Y, Prado-Martinez J, Sudmant PH, Narasimhan V, Ayub Q, Szpak M, et al. Mountain gorilla genomes reveal the impact of long-term population decline and inbreeding. Science. 2015;348 6231:242-5. doi:10.1126/science.aaa3952.<br>7.Watterson G and Guess HA. Is the most frequent allele the oldest? Theoretical population biology. 1977;11 2:141-60.<br>8.Sabeti PC, Schaffner SF, Fry B, Lohmueller J, Varilly P, Shamovsky O, et al. Positive natural selection in the human lineage. science. 2006;312 5780:1614-20.<br>9.Fay JC and Wu C-I. Hitchhiking under positive Darwinian selection. Genetics. 2000;155 3:1405-13.<br>10.Rocha D, Billerey C, Samson F, Boichard D and Boussaha M. Identification of the putative ancestral allele of bovine single-nucleotide polymorphisms. Journal of Animal Breeding and Genetics. 2014;131 6:483-6.<br>11.Hacia JG, Fan J-B, Ryder O, Jin L, Edgemon K, Ghandour G, et al. Determination of ancestral alleles for human single-nucleotide polymorphisms using high-density oligonucleotide arrays. Nature genetics. 1999;22 2:164-7.<br>12.Naji MM, Utsunomiya YT, Sölkner J, Rosen BD and Mészáros G. Investigation of ancestral alleles in the Bovinae subfamily. BMC genomics. 2021;22:1-12.<br>13.Khan A, Patel K, Shukla H, Viswanathan A, van der Valk T, Borthakur U, et al. Genomic evidence for inbreeding depression and purging of deleterious genetic variation in Indian tigers. Proceedings of the National Academy of Sciences. 2021;118 49:e2023018118.<br>14.Hu J-Y, Hao Z-Q, Frantz L, Wu S-F, Chen W, Jiang Y-F, et al. Genomic consequences of population decline in critically endangered pangolins and their demographic histories. National Science Review. 2020;7 4:798-814.
**Additional Information:**	
**Question**	**Response**
Are you submitting this manuscript to a special series or article collection?	No
**Experimental design and statistics**<br><br>Full details of the experimental design and statistical methods used should be given in the Methods section, as detailed in our	Yes

Minimum Standards Reporting Checklist. Information essential to interpreting the data presented should be made available in the figure legends.<br><br>Have you included all the information requested in your manuscript?	
**Resources**<br><br>A description of all resources used, including antibodies, cell lines, animals and software tools, with enough information to allow them to be uniquely identified, should be included in the Methods section. Authors are strongly encouraged to cite Research Resource Identifiers (RRIDs) for antibodies, model organisms and tools, where possible.<br><br>Have you included the information requested as detailed in our Minimum Standards Reporting Checklist?	Yes
**Availability of data and materials**<br><br>All datasets and code on which the conclusions of the paper rely must be either included in your submission or deposited in publicly available repositories (where available and ethically appropriate), referencing such data using a unique identifier in the references and in the "Availability of Data and Materials" section of your manuscript.<br><br>Have you have met the above requirement as detailed in our Minimum Standards Reporting Checklist?	Yes

Powered by Editorial Manager® and ProduXion Manager® from Aries Systems Corporation

Manuscript

Click here to access/download;Manuscript;MainText_Jan 3.docx

Enhancing inbreeding estimation and global conservation insights through chromosome-level assemblies of the Chinese and Malayan pangolin

Tianming Lan[1,2,3,†], Yinping Tian[3,†], Minhui Shi[4,5†],, Boyang Liu[3], Yu Lin[3], Yanling Xia[3], Yue Ma[3], Sahu Sunil Kumar[4], Qing Wang[3], Jun Li[2], Jin Chen[3], Fanghui Hou[6,7], Chuanling Yin[3], Kai Wang[2], Yuan Fu[3], Tengcheng Que[8,9], Wenjian Liu[8], Huan Liu[3], Haimeng Li[3,10,*] and Yan Hua[2,*]

[1] BGI Life Science Joint Research Center, Northeast Forestry University, Harbin 150040, China

[2] Guangdong Provincial Key Laboratory of Silviculture, Protection and Utilization, Guangdong Academy of Forestry, Guangzhou 510520, China

[3] College of Wildlife and Protected Area, Northeast Forestry University, Harbin 150040, China

[4] BGI Research, Wuhan 430074, China

[5] State Key Laboratory of Agricultural Genomics, BGI-Shenzhen, Shenzhen 518083, China

[6] Guangdong Wildlife Rescue Monitoring Center, Guangzhou 510520, China

[7] Pangolin Conservation Research Center of National Forestry and Grassland Administration, Guangzhou 510520, China

[8] Faculty of Data Science City University of Macau, Macau 999078, China

[9] Guangxi Zhuang Autonomous Terrestrial Wildlife Rescue Research and Epidemic Diseases Monitoring Center, Nanning 530025, China

[10] Heilongjiang Key Laboratory of Complex Traits and Protein Machines in Organisms, Harbin 150040, China

*Correspondence address. Yan Hua, Guangdong Provincial Key Laboratory of Silviculture, Protection and Utilization, Guangdong Academy of Forestry, Guangzhou 510520 China. E-mail: wildlife530@hotmail.com; Haimeng Li, College of Wildlife and Protected Area, Northeast Forestry University, Harbin 150040, China. E-mail: lihaimeng66@163.com

†Equal contributions.

ORCID iDs: Tianming Lan [0000-0002-6934-0439]; Yinping Tian; Minhui Shi [0009-0002-9582-6018]; Boyang Liu [0000-0001-7378-3454]; Sahu Sunil Kumar [0000-0002-4742- 9870]; Qing Wang [0000-0003-4744-8729]; Jun Li [0000-0002-7083-5359]; Jin Chen [0009-0005-2453-6912]; Fanghui Hou [0000-0002-6611-1198]; Kai Wang [0000-0003-0371-348X]; Huan Liu [0000-0002-6902-9931]; Haimeng Li [0000-0003-4355-5906]; Yan Hua [0000-0001-8316-3937].

Abstract

A high-quality reference genome coupled with resequencing data is a promising strategy to address issues in conservation genomics. This has greatly enhanced the development of conservation plans for endangered species. Pangolins are fascinating animals with a variety of unique features. Unfortunately, they are the most trafficked wild animal in the world. In this study, we assembled a chromosome-scale genome with HiFi long reads and Hi-C short reads for the Chinese and Malayan pangolin and provided two new representative reference genomes for the pangolin species. We found a great improvement in the evaluation of genetic diversity and inbreeding based on these high-quality genomes and obtained different results for the detection of genome-wide extinction risks compared with genomes assembled using short reads. Moderate inbreeding and genetic diversity were reverified in these two pangolin species, except for one Malayan pangolin population with high inbreeding and low genetic diversity. Moreover, we identified a much higher inbreeding level (F_{ROH}=0.54) in the Chinese pangolin individual from Taiwan Province compared with that from mainland China, but more than 99.6% runs of homozygosity (ROH) fragments were restricted to less than 1Mb, indicating that the high F_{ROH} in Taiwan Chinese pangolins may have accumulated from historical inbreeding events. Furthermore, our study is the first to detect relatively mild genetic purging in pangolin populations. These two high-quality reference genomes will provide valuable genetic resources for future studies and contribute to the protection and conservation of pangolins.

Keywords: Chinese pangolin; Malayan pangolin; inbreeding; genetic purging; conservation genomics

Introduction

High-quality reference genomes enable the comprehensive analysis of population genomics and contribute to the

revolution in conservation genomics[1]. However, fewer than 1% of the threatened species listed on the International Union for Conservation of Nature (IUCN) red list have a reference genome[2], and this number will further decrease if long-read assembly is considered. In the shift from conservation genetics to conservation genomics[3], high-quality reference genomes play an important role in providing the necessary support for the conservation of endangered species[1, 4]. In particular, genetic rescue is considered an important strategy to facilitate gene flow and avoid inbreeding to increase the health of a population [5, 6]. A deep understanding of the genome-wide extinction risks and the basic genetic background of a small population is necessary for developing evidence-based strategies for genetic rescue. These include population structure, genomic diversity, genome-wide inbreeding, mutational load, and population demography[7-11]. A high-quality reference genome usually facilitates population-level studies in conservation genomics[1]. For example, the evaluation of inbreeding by measuring ROH relies on a high-quality reference genome with outstanding contiguity, because the long ROH fragments in small populations with high-level inbreeding often span several millions of base pairs [11-13], and can hardly be detected based on the fragmented genomes assembled by short reads.

The pangolin is a living fossil with many unique biological characteristics [14, 15], such as overlapping keratin scales covering the body, a specialized diet, a long and muscular tongue, a sensitive olfactory system, and burrowing ability [16, 17]. Locals across its distribution areas have traditionally used its scales and meat for medicine and food [18]. The overexploitation of pangolins driven by the soaring demand for luxury food and traditional Chinese medicine, has pushed this animal to the edge of extinction [19-22]. Currently, the pangolin is the most heavily trafficked wild animal worldwide, with more than 900,000 individuals poached over the last two decades and 67 countries from six continents involved in illegal poaching and trade [23]. For protection, all pangolin species have been placed into Appendix I of the Convention of International Trade of Endangered Species of Wild Fauna and Flora (CITES) as of 2016. Poaching is more rampant for Asian pangolins compare with African pangolins, particularly the Malayan pangolin (*Manis javanica*, hereafter MJ; NCBI:txid9974) and the Chinese pangolin (*Manis pentadactyla*, hereafter MP; NCBI:txid143292), which are under extreme survival pressure because of significant poaching and trafficking [8]. These two species have been listed as “Critically Endangered” on the IUCN Red List since 2014.

Previous studies identified two main Malayan populations[8] (hereafter MjavA and MjavB) and three main Chinese pangolin populations (hereafter MpenA, MpenB and MpenC). The MjavA population was identified from the mainland (China and Myanmar) and diverged from the MjavB population, which may have originated from the Southeast Asian islands. Among the three Chinese pangolin populations, the MpenA population is a newly discovered population distributed in Guangdong, China. The MpenB population is distributed over a vast area, including southern China and Thailand. The MpenC population highly diverged from the other two populations, which are likely to have originated from Myanmar (Fig. 1A, Supplementary Table S1 and S2). Although the varying population of the Chinese pangolin is still controversial[25], the highly divergent pangolin populations indicate that deep isolation may have occurred, which is usually detrimental to the survival of endangered species.

Pacific Biosciences (PacBio) high-fidelity (HiFi) sequencing technology combined with a HiFi-specific assembler and Hi-C or parental sequencing data, can generate high-quality , haplotype-resolved *de novo* assemblies, which represents one of the most promising strategies for genome assembly [24, 25]. This technique may facilitate a more accurate analysis of genome-wide genetic risks, specifically with respect to inbreeding. Because of the urgent need for establishing genomic backgrounds to support conservation, several reference genomes of pangolins have been assembled, annotated, and published[8, 16, 26-29]. However, high-quality reference genomes assembled from HiFi long-reads do not exist for pangolins. In this study, we present the genomes of the Malayan and Chinese pangolins at the chromosome-scale with haplotypes resolved down to the chromosomal-level, which provides new

representative reference genomes for the pangolin species. We systematically examined the genomic backgrounds and evaluated the genome-wide extinction risks for five pangolin populations based on these two reference genomes.

Results

New representative reference genomes for the pangolin

To obtain high-quality reference genomes for both the Malayan and Chinese pangolin, we combined PacBio HiFi long reads, Hi-C reads and DNBSEQ short reads for genome assembly (Table. 1). We first generated phased contigs for both species and the haploid-resolved contigs were further linked at the chromosome-level by combining the HiFi long reads and Hi-C reads. We assembled 19 and 20 chromosome-scale pseudomolecules for MJ and MP, respectively (Fig. 1B, Supplementary Fig. S1), which was consistent with the karyotypic analysis[30], although the karyotype in pangolins may vary[30]. The diploid genome sizes assembled for MJ and MP were ~2.56 Gb and ~2.64 Gb, and represented 96.78% and 92.48% of the estimated genome size (~2.65 Gb for MJ and ~2.86 Gb for MP), respectively (Supplementary Fig S2). The contig and scaffold NG50 of the MJ/MP were 46.22Mb/56.16 Mb and 141.80 Mb/140.71 Mb, respectively (Table 1, Supplementary Table S3). In addition, we identified the X-chromosome and Y-linked regions of both genomes (Supplementary Fig. S3). The hifiasm assembler simultaneously yielded in two groups of haplotigs for each of the MJ (hereafter MJH1, MJH2) and MP (hereafter MPH1, MPH2) genomes (Supplementary Table S4). Multiple lines of evidence support the high completeness and low level of artificial duplication of the haplotype-resolved assemblies. Both the base-level quality evaluation and the structural-level assessment showed that all genomes (2 diploid assemblies and 4 haploid assemblies) had high assembly accuracy (see the Supplementary Material online). High collinearity between the MP and MJ genomes was also observed with 4 fissions and 3 fusions in the MJ genome compared with the MP genome (Fig. 1B), which was consistent with a previous karyotypic analysis [47] showing that these two genomes were accurately assembled at the chromosome level. Overall, we are confident in these two new, high-quality, and representative reference genomes for pangolins.

Table 1: Summary statistics for the genome sequences

Category	Metric	Chinese pangolin (MP)	Malayan pangolin (MJ)
**Sequencing Data**	WGS (Gb)/depth (X)	254.05/96.18	245.34/95.87
	HiFi (Gb)/depth (X)	65.81/25.20	95.99/37.51
	Hi-C (Gb)/depth (X)	239.77/90.78	227.48/88.89
	RNA-seq (Gb)	6.21	6.35
**Continuity**	Genome size (Gb)	2.64	2.56
	Scaffold NG50[a] (Mb)	140.71	141.80
	Scaffold number	89	62
	Longest scaffold (Mb)	234.25	241.98
**Structural accuracy**	Reliable blocks[b]	96.56%	98.09%
	False duplications[c]	0.40%	0.48%
	Curation	manual	manual
**Base accuracy**	Base pair QV	57.06	55.08
	k-mer completeness	96.81	95.40
**Functional completeness**	BUSCO[d] assessment	98.00% complete	97.50% complete
	Transcript mappability	97.77%	97.26%
**Chromosome status**	Assigned[e]	97.22	98.72
	Pseudo-chromosomes Number	20	19

[a]Scaffold NG50: This metric represents the minimum length of a scaffold such that when all scaffolds are ranked by size, the cumulative length of scaffolds exceeding this threshold accounts for at least half of the estimated genome size. [b]Reliable blocks: These refer to genomic regions that are robustly supported by both Hi-C and HiFi sequencing reads, ensuring a reliable assembly[31]. [c]False duplications:These are additional copies of k-mers present in a genome assembly beyond the expected count, as indicated by the k-mer histogram derived from the original high-fidelity reads[31]. [d]BUSCO: Benchmarking Universal Single-Copy Orthologs . [e]Assigned: This term denotes the percentage of the genome assembly that has been confidently assigned to specific chromosomes.

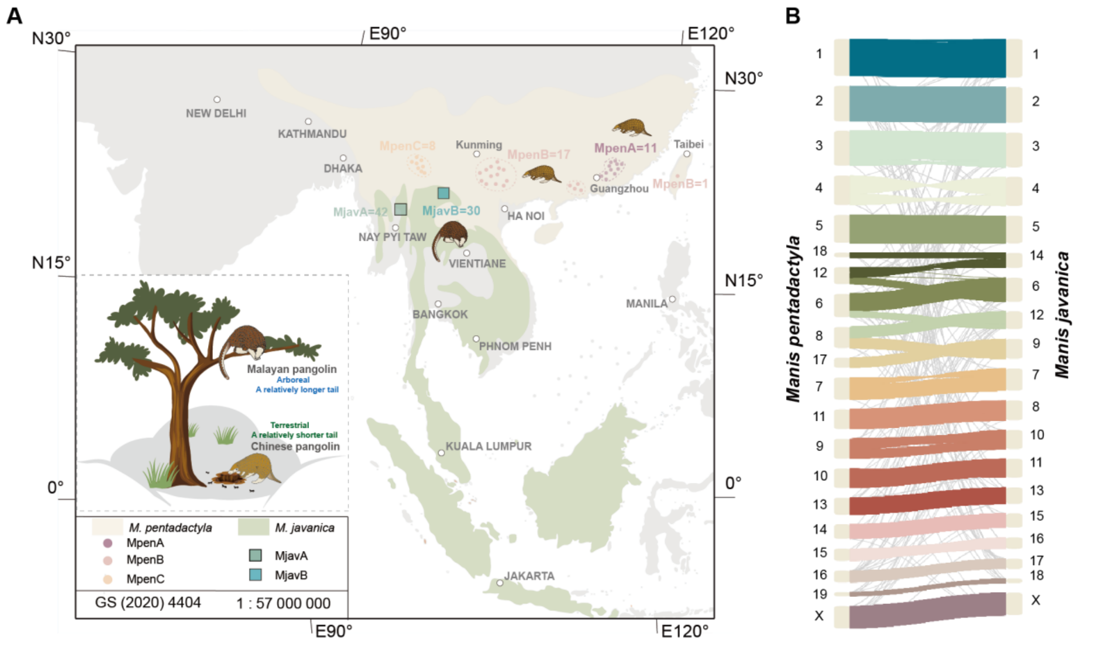

Figure 1: Introduction to the species distribution and chromosome synteny of the Chinese and Malayan pangolins. (A) The distribution area and sampling sites of the Chinese and Malayan pangolins in this study. The circles represent sampling sites of the Chinese pangolins reported by Wang *et al.*[32] Samples without detailed locations are not shown on the map. (B) The chromosome-scale synteny analysis between the Malayan pangolin and Chinese pangolin genomes.

Genome annotation

The total length of the repeat elements reached 1,260.92 Mb and 1,332.41 Mb, which accounted for 49.25% and 50.44% of the MJ and MP genomes, respectively (Supplementary Table S5). The composition of repeats in the MJ and MP genomes was similar, with the most abundant repeat element being LINE (MJ: 35.41%, MP: 37.36%), followed by LTR (MJ: 11.14%, MP: 13.61%), DNA element (MJ: 2.23%, MP: 2.11%) and SINE (MJ: 0.96%, MP: 0.91%). We predicted 19,680 and 19,886 gene models in the MJ and MP genomes, respectively (Supplementary Table S6). The gene regions spanned over 853.90 Mb and 791.49 Mb, which comprised 33.36% and 29.96% of the MJ and MP genomes, respectively (Supplementary Table S6). The average gene length, exon length, and intron length were 43.39 kb, 175.75 bp and 4.92 kb for the MJ genome and 39.80 kb, 175.07 bp and 4.64 kb for the MP genome (Supplementary Table S6). The BUSCO analysis revealed high completeness for the gene sets of both genomes, with the lowest BUSCO score greater than 95% (Supplementary Table S7). Overall, 19,575 (99.47%) and 19,792 (99.53%) genes were functionally annotated in the MJ and MP genomes, respectively (Supplementary Table S8). In addition, we predicted 288/435 rRNA, 1,296/1,348 miRNA, 806/350 tRNA, and 1,521/1,412 snRNA in the MJ/MP genomes (Supplementary Table S9).

The HiFi genome improves the evaluation of genetic diversity and inbreeding

We compared the short-read assembled genome (hereafter SG, the YNU_ManPten_2.0 and YNU_ManJav_2.0 were used here to represent the Chinese pangolin and Malayan pangolin genome, respectively) and the long-read assembled genome (PacBio HiFi assembled genome in this study, hereafter long-read assembled genome (LG)) to evalute for the evaluation of commonly used genetic parameters in population genomics, particularly in conservation genomics, including population structure, population history and separation, genetic diversity, and inbreeding (Supplementary Table S10).We found that the population structure (principal component analysis (PCA), phylogenetic tree, and admixture), population history, and population separation (inferred by MSMC2) were not

significantly affected by these two types of reference genomes (Supplementary Fig. S4-S7), because the results calculated based on the LG and SG were the same and consistent with previous reports. In contrast to other studies, however, we did not observe a distinct separation between the individual from Taiwan Province and other Chinese pangolins, although the Chinese pangolin in Taiwan Province is considered a subspecies of the Chinese pangolin[33] Therefore, either the genetic differentiation is still relatively small between the Chinese pangolin in Taiwan Province and mainland China, or the Taiwan individual was recently translocated from mainland China. For genetic diversity, the difference between π values calculated by LG and by SG was not large; however, this difference was significant, with a higher π value calculated based on the LG compare with that for the SG (Fig. 2A, Supplementary Table S10). Moreover, the ROH is an important genetic factor that reflects the inbreeding level in a population, whereas it was very sensitive to the quality of the reference genome. By screening ROHs across the genome to evaluate genome-wide inbreeding, we identified significant differences between the SG and LG (Table S11-S12). For the Malayan pangolin populations, the F_{ROH} calculated based on the LG was markedly higher compare with those calculated based on the SG (Fig. 2B, Supplementary Table S13). However, for the Chinese pangolin, this difference was significant only for ROHs larger than 1 Mb, although F_{ROH} was larger for the LG at other ROH lengths (Fig. 2C).

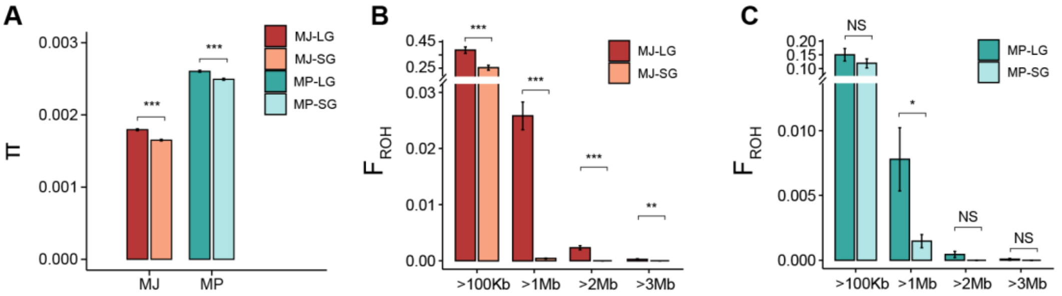

Figure 2: Comparison of the genome-wide genetic diversity and inbreeding estimated based on the LG (long-read HiFi assembled genome) and SG (short-read assembled genome). (A) Comparison of genome-wide π calculated based on the LG and SG in the Chinese and Malayan pangolin genomes. (B) Comparison of F_{ROH} calculated based on LG and SG in the Malayan pangolin genomes. (C) Comparison of F_{ROH} calculated based on LG and SG in the Chinese pangolin genomes. Note: NS: $p \geq 0.5$, $*p < 0.05$, $**p < 0.01$, $***p < 0.001$.

Genome-wide genetic diversity and inbreeding

Considering the improvement in evaluating the genetic diversity and inbreeding based on the high-quality HiFi genomes, we performed a reassessment based on the LG and found that the genome-wide genetic diversity (π) of the Chinese pangolin and Malayan pangolin were 0.0026 and 0.0018, respectively, which were both higher compare than those calculated based on the SG (π_{MP}=0.0025, π_{MJ}=0.0016) (Supplementary Table S10). For the Chinese pangolin populations, MpenB exhibited the highest genetic diversity (π_{MpenB} = 0.0020), followed by the MpenC (π_{MpenC} = 0.0018) and MpenA (π_{MpenA} = 0.0017), which was consistent with previous study, but with higher π values[34]. Among the Malayan pangolin populations, the genetic diversity of MjavB (π_{MjavA} = 0.0024) was higher than that of the MjavA (π_{MjavB} = 0.0007). The mean *He* of the MjavA and MjavB were 0.063% and 0.189%, respectively, which were higher than the 0.043% and 0.141% values in a previous study[8]. The average genetic diversity of the Chinese pangolin was higher compared with that of the Malayan pangolin (Supplementary Table S10).

Inbreeding in small populations increases genome-wide homozygosity, and the resulting depression accelerates the loss of genetic diversity. For the MJ and MP populations, the average number of ROH fragments in each individual was 3,659.22±215.96 and 1,543.43±186.90, respectively. For both species, the ROHs were restricted to relatively small fragments (<1Mb), and the number of ROHs longer than 1Mb only accounted for 0.94% and 1.27% of the ROH fragments for the MP and MJ genomes, respectively[8]. The total length of ROHs larger than 1Mb also

accounted for a small proportion of the two genomes (MP: 5.19%; MJ: 6.69%) (Fig. 3A and 3B). [35]We did not observe any ROH fragments greater than 5Mb in either species. Similarly, the F_{ROH} in the MJ genomes (0.39±0.024) was higher than that in the MP genomes (0.15±0.023) (Supplementary Table S13). For ROHs longer than 1Mb, however, the F_{ROH} was sharply reduced to 0.026±0.003 and 0.0078±0.002 for the MJ and MP, respectively (Supplementary Table S13), which were much lower than that reported in a previous study[8, 35]. The very high minor allele frequency of 0.2 and other abnormally stringent filtering parameters used in the previous study as a threshold to filter the SNP may severely dilute the SNPs across the genome, resulting in an overestimation of the inbreeding in the genome.

We further compared the ROH distribution in different populations of these two species (Supplementary Fig. S8-S9). For Malayan pangolins, the inbreeding in the MjavA population (F_{ROH}=0.55±0.007) was more serious compare with that in the MjavB population (F_{ROH}=0.15±0.012) (Fig. 3C-3D, Supplementary Table S13). Although this difference became smaller for ROHs greater than 1Mb, it was still significant (Fig. 3D). Among the three Chinese pangolin populations, the inbreeding of the MpenA (F_{ROH}=0.18±0.036) and MpenB (F_{ROH}=0.17±0.039) populations were comparable, but much worse compare with that of the MpenC population (F_{ROH}=0.06±0.005) (Fig. 3C and 3E, Supplementary Table S13). Similarly, the differences in F_{ROH} between the three MP populations were reduced for ROHs longer than 1Mb (Fig 3E, Supplementary Table S13). As reported in a previous study[8], we found that the F_{ROH} of the Taiwan individual (F_{ROH}=0.54) was much higher than all other individuals in the MP population (Fig. 3C). Notably, we found that 99.6% of the ROH fragments in the genome of the Taiwan individual were less than 1Mb, which was much higher than that reported in the previous study (Supplementary Table S13). In addition, the F_{ROH} varied greatly among individuals in the MpenA or MpenB population, but this was not observed in the MpenC, MjavA and MjavB populations.

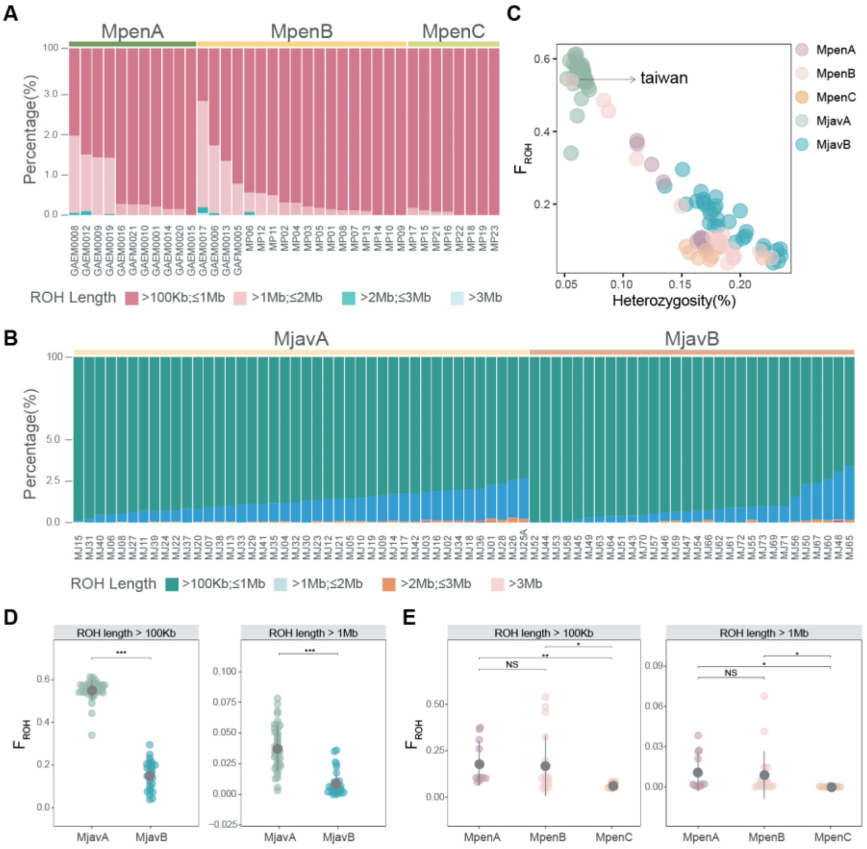

Figure 3: Genome-wide inbreeding estimated by ROH in the Chinese and Malayan pangolin populations. (A) The length distribution of ROH across the genome in the Chinese pangolin population. (B) The length distribution of ROH across the genome in the Malayan pangolin

population. (C) Genome-wide heterozygosity and inbreeding estimates (F_{ROH}) for all five pangolin populations. (D) Comparison of the averaged F_{ROH} in the MjavA and MjavB populations of the Malayan pangolins. (E) Comparison of the averaged F_{ROH} in the MpenA, MpenB and MpenC populations of Chinese pangolins.

Genome-wide mutational load

The mutational load is the burden of deleterious variants carried by a population, and reflects the evolutionary fitness of a population[36]. Although studies have examined the distribution of mutational load for the Chinese and Malayan pangolins[8, 32, 35], the HiFi genomes in this study provide new insight into the accumulation of mutational load in pangolins. We screened three categories of mutational load (loss of function, LoF; missense mutation; deleterious nonsynonymous mutation, dnsSNP) based on the HiFi genomes for the Chinese and Malayan pangolins (Fig.4A and 4B, Supplementary Table S14 and S15). We calculated the individual-level derived mutational load for each population to avoid bias introduced by different population sizes. In a previous study, the MpenC population was to harbor the most mutational load. In this study, however, we found that individuals in the MpenC population harbored the most mutational load, which was significantly more than that in the MpenB and MpenA populations (Fig. 4A). This may be the result of the HiFi reference genome used in this study and the different methods for the identifying the derived allele across the genome. The missense mutations and dnsSNPs in the MpenA population were comparable to those in the MpenB population (Supplementary Fig. S10a and S10b); however the MpenB population harbored many more LoFs compared with the MpenA population (Fig. 4A). Next, we focused on the derived homozygous mutational load (DHMD) and found that the MpenC population harbored the most DHMD, whereas MpenA and MpenB contained comparable DHMD (Fig. 4C, Supplementary Fig. S10c-S10f). For Malayan pangolins, the MjavB population harbored much more derived LoF compared with that of the MjavA population (Fig. 4B, Supplementary Table S14 and S15). However, the proportion of DHMD in the MjavA population for the LoF was comparable to that in the MjavB population (Fig. 4D). The MjavA population exhibited a higher proportion of DHMD for the dnsSNP and missense mutations than that of the MjavB population (Supplementary Fig. S10f), possibly because of the more efficient genetic purging of large-effect deleterious mutations (LoF), which was not investigated in previous studies. In the Genomic Evolutionary Rate Profiling (GERP) analysis, we obtained a highly similar result with the MpenC population harboring the most relative mutational load among the MP populations and the MjavA population harboring more relative mutational load than that in the MjavB population (Fig. 4E and 4F).

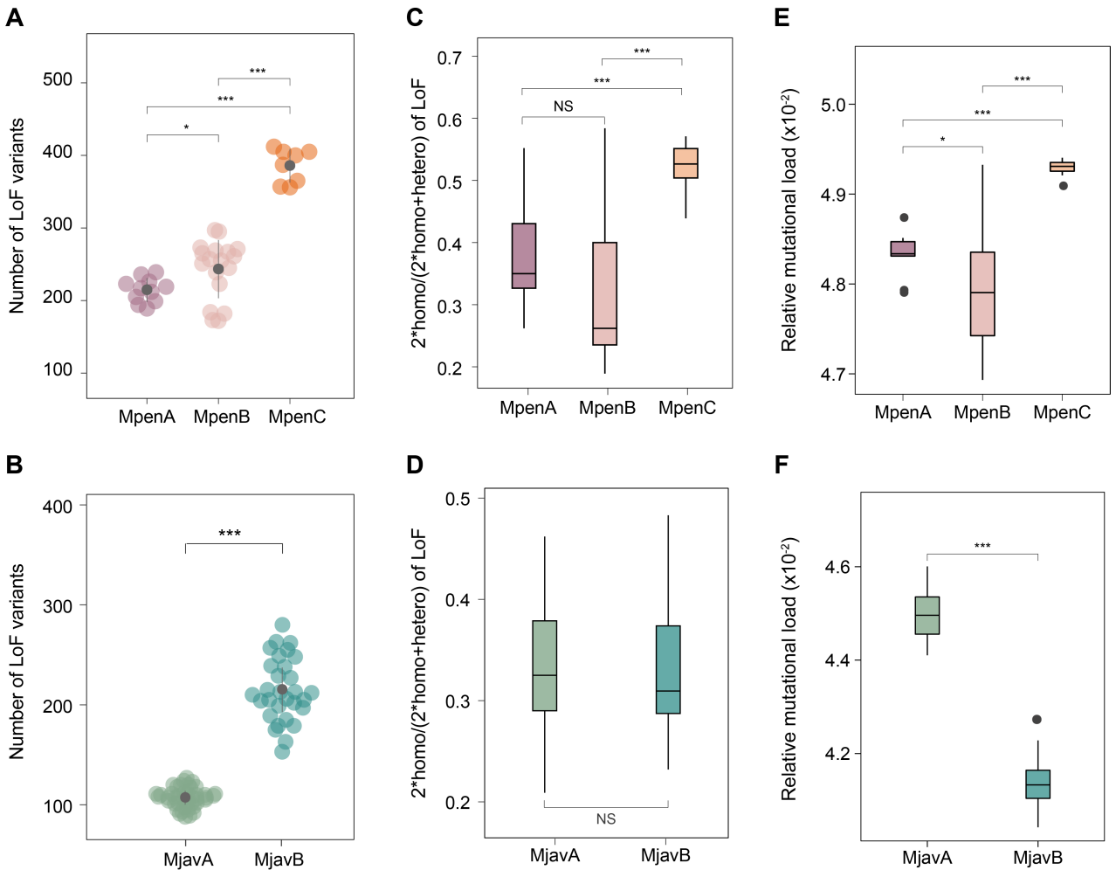

Figure 4: Mutational load in the Chinese and Malayan pangolin populations. (A) Total number of individual-level LoF mutations across the Chinese pangolin populations. (B) Total number of individual-level LoF mutations across the Malayan pangolin populations. (C) The ratio of homozygous LoF mutations in the Chinese pangolin populations was calculated by the formula: 2 × homozygous sites / (2 × homozygous sites + heterozygous site). (D) The ratio of homozygous LoF mutations in the Malayan pangolin populations was calculated using the same formula as that for the Chinese pangolin. (E) Relative mutational load in the Chinese pangolin populations (top 0.1% of GERP scores). (F) Relative mutational load in the Malayan pangolin populations (top 0.1% of GERP scores). The LoF here means loss-of-function mutations.

SFS (site frequency spectrum) analysis revealed that 7.91% and 9.07% of putatively damaging and neutral alleles, respectively, were fixed in the MpenC population. These two ratios were 6.84% and 9.53%, respectively, in the MjavA population; however, the proportion of the fixed alleles in the other three populations (MpenA, MpenB and MjavB) was much smaller (Fig. 5A and 5B). This indicates that the MpenC and MjavA populations may have experienced population bottleneck events, and genetic drift drove more rare alleles to fix into these two populations [7]. By comparing SFS lines between the Chinese and Malayan pangolins, we found that the SFS lines were flatter for polymorphic loci (fixed alleles excluded) in the Malayan pangolins, whereas the flattest SFS line was observed in the MjavA population, indicating the possibility of more serious bottlenecks in the evolutionary history of the MjavA population.

Genetic purging in pangolin populations

Genetic purging is an important process that has an impact on the accumulation of deleterious mutations in the population and it is usually more evident in small populations; however, few studies have discussed this issue in pangolins. To determine whether genetic purging has occurred in these pangolin populations facilitated by inbreeding, we first compared the occurrence of mutational load (LoF, missense mutation and dnsSNP) in the ROH regions (ROH*f*, the ratio of the number of mutational load to synonymous mutations in the ROH regions across the genome) and outside of the ROH regions (nonROH*f*, the ratio of the number of mutational load to synonymous mutations in

the nonROH regions across the genome)[10]. In the Malayan pangolin populations, the ROH*f* of highly deleterious mutations (LoF) was significantly lower than the nonROH*f* in both the MjavA and MjavB population (Fig. 5C). This denoted that many large-effect deleterious alleles (e.g. LoF) still existed but are masked in nonROH regions in both the two populations, indicating that the purging was less efficient to remove recessive deleterious mutations across the whole genome. Further inspection we found the difference between the ROH*f* and nonROH*f* was smaller in the MjavA population than that in the MjavB population (Fig. 5D and Supplementary Fig. S11a-S11c), indicating that the higher-level of inbreeding in the MjavA population may have facilitated the exposing of more recessive deleterious alleles to be homozygous, which has resulted in a more efficient purging of deleterious mutations in this population than in the MjavB population [10, 11].

In the Chinese pangolin populations, the ROH*f* for LoF was lower than nonROH*f* in all three populations. The values of nonROH*f* and ROH*f* in the MpenA population were very similar, but the nonROH*f* was significantly higher compared with that in the ROH*f* in both the MpenB and MpenC populations (Fig. 5E and 5F), indicating that the purging of deleterious alleles in the MpenA population might be more efficient than that in the MpenB and MpenC populations for the LoF mutations. However, this was not obvious for the relatively small-effect dnsSNP and missense mutations (Supplementary Fig. S11d-S11f). The Rxy also indicated that the MpenA population harbored the fewest deleterious mutations, followed by the MpenB and the MpenC populations (Supplementary Fig. S12). In addition, the number of fixed damaging alleles was not significantly less than neutral alleles in all five populations (Fig. 5A and 5B), further suggesting that the genetic purging in the pangolin populations is weak and not sufficient to clear a large number of deleterious mutations.

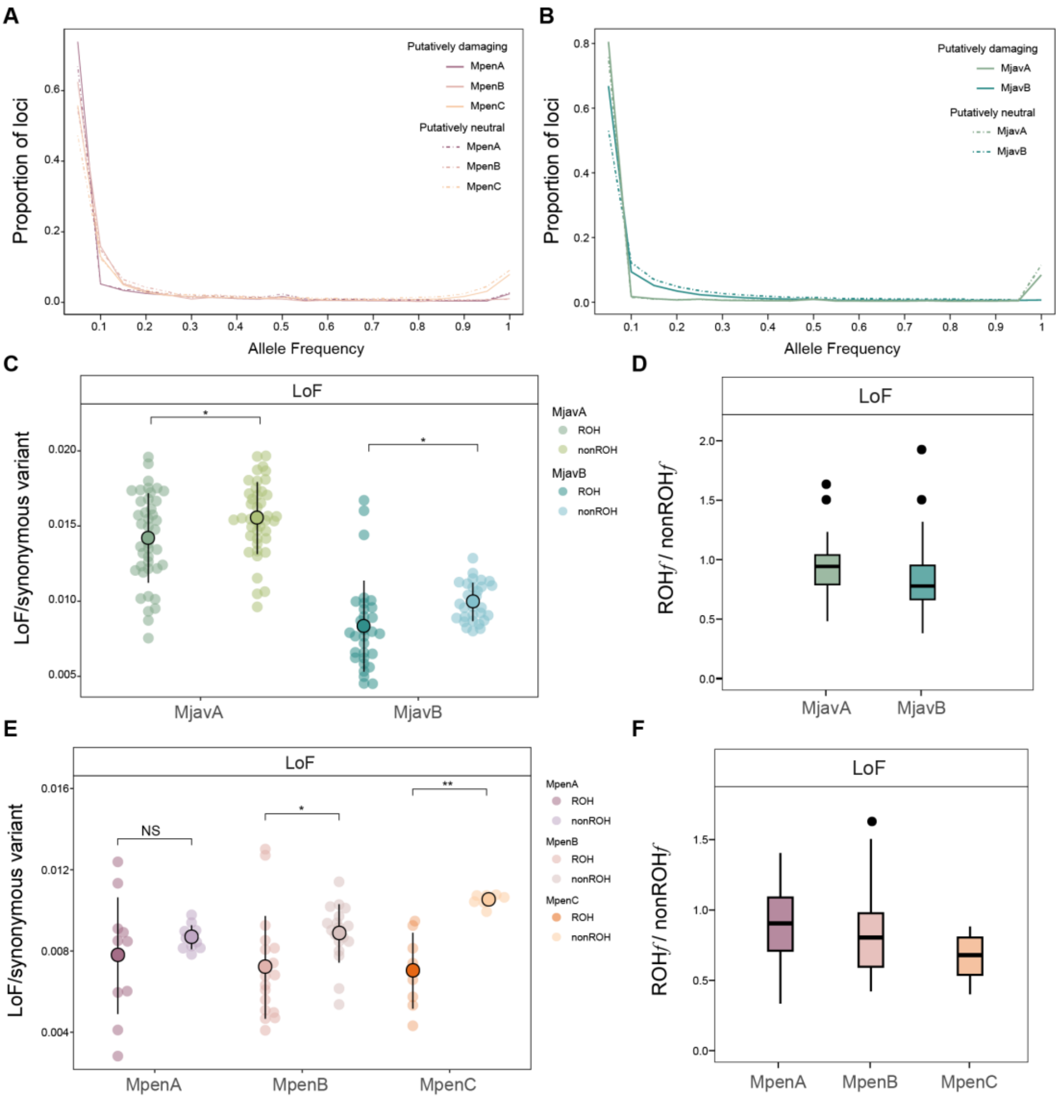

Figure 5: The SFS and genetic signals of genetic purging in pangolin populations. (A) SFS for putatively damaging (LoF and missense mutations) and neutral mutations (intergenic variants) in the MpenA, MpenB, and MpenC populations. (B) SFS for putatively damaging and neutral mutations in the MjavA and MjavB populations. (C) Dot plot showing the occurrence of LoF mutations in the two Malayan pangolin populations calculated as the ratio of the number of the mutational load to synonymous mutations in the ROH regions (ROH*f*) or nonROH (nonROH*f*) regions across the genome. Each point signifies the LoF frequency of an individual. The large dots represent the average LoF frequency for the population, while the lines indicate the standard deviation range around the mean. (D) The ratio of ROH*f* to nonROH*f* for the LoF in the two Malayan pangolin populations. The box represents the interquartile range (IQR), stretching from the first quartile (Q1) to the third quartile (Q3). The line that bisects the box indicates the median value. The whiskers extended from the box to show the variability of the data, typically reaching to the minimum and maximum values that fall within 1.5 times of the IQR from Q1 and Q3, respectively. (E) Dot plot showing the occurrence of LoF mutations in the three Chinese pangolin populations calculated as that in (C). (F) The ratio of ROH*f* to nonROH*f* for the LoF in the three Chinese pangolin populations.

Discussion

HiFi genomes improve the evaluation of genetic diversity and inbreeding

Accurate and precise evaluation of genome-wide extinction risks by measuring a series of genetic parameters is the central issue in conservation genomics and primarily depends on the quality of the reference genome [1]. However,

what genetic parameters can be improved the most with a better reference genome? Here, we showed that the two genetic parameters promoted by a higher-quality reference genome are genetic diversity (π) and inbreeding (ROH) (Fig. 3A-3C). We detected more variants across the genome based on the LG than the SG because long reads could 1) span much more complex genomic regions[1] and 2) generate much longer contigs than short reads[37, 38]. Many genomic regions that cannot be assembled by short reads can be assembled by long reads, and these regions may contain important variants. In addition, longer contigs facilitate a higher number of reads that align accurately with the reference genome. Both of these may contribute to enhancing the accuracy of genetic diversity calculations. Therefore, we do not recommend comparing the genetic diversity calculated based on the SG to that calculated based on the LG; however, it may still make sense to compare the genetic diversity that was calculated based on the SG genomes between different populations.

The estimation of inbreeding by detecting ROH across the genome highly depends on genome contiguity, because short contigs in the SG can hardly span over long ROH fragments. As predicted, the inbreeding level detected using the LG were significantly higher than those identified using the SG for ROH fragments larger than 1Mb. When we focused on ROHs larger than 100kb, this difference in F_{ROH} was still significant in the Malayan pangolin population but not in the Chinese pangolin population (Fig 3B and 3C), which we inferred should have resulted from the different contiguities of the two pangolin genomes. Indeed, the contig N50 of the SG for the Chinese pangolin (133.77 kb) was much longer than that of the SG for the Malayan pangolin (73.8 kb), allowing for the detection of ROH longer than 100 kb. In contrast, the scaffold N50 of the Malayan pangolin genome was longer than that of the Chinese pangolin genome, suggesting that contiguity contributes more than scaffold contiguity to the detection of long ROH across the genome. It is worth noting that the Chinese Pangolin displays a higher $F_{ROH>1\,Mb}$ in comparison to the Malayan Pangolin when the LG was used for ROH detection. However, this result was opposite when the SG genome was used for detection of ROH fragments. This undisputed supported that the high-quality reference genome assembled by long reads is essential for accurately determining the inbreeding level.

Genome-wide extinction risks in different pangolin populations

Although the Chinese and Malayan pangolin are listed as Critically Endangered species by the IUCN Red List, the genome-wide genetic diversity of these two species is moderate and even higher than other endangered flagship species[8, 39], such as the tiger [37], giant panda[40], golden snub-nosed monkey[41], and kākāpo[11]. In this study, we found an even higher genetic diversity than the previous reports[8, 39] for the two pangolin species (Supplementary Fig. S13, Supplementary Table S16). Although the pangolin populations have been declining for a long time, the recent population decline caused by poaching and illegal trade is more serious than ever, which has resulted in a very rapid decline in population size. Therefore, genetic drift and inbreeding may not have resulted in a substantial decrease in genetic diversity. Although the high-quality HiFi genome improved the estimation of inbreeding, the F_{ROH} in both species was still lower than many other endangered species[7, 37], indicating a relatively fine intrinsic genetic background for these pangolin populations.

We observed a much faster and sharper population decline for the MjavA population within the most recent 10,000 years compared with the other four pangolin populations (Supplementary Fig. S7), indicating that this Malayan pangolin population has a serious extinction risk. The LG-based population genomic analysis also revealed that both the genetic diversity (π_{MjavA}=0.0007) and inbreeding ($F_{ROH>100kb}$=0.55) in the MjavA Malayan population were much worse than those in other pangolin population as which may be caused by the isolation and limited gene flow with other populations, because the MjavA population is distributed across Southeast Asia [8] and the gene flow is easily separated by islands. However, we cannot precisely locate this population because of the lack of accurate sampling locations [8], which should be the subject of future conservation work. Another aspect to consider is that the Taiwan Chinese pangolin individual exhibited a very high level of inbreeding, with ROH fragments longer than 1Mb accounting for only ~0.4%, much less than the 8.04% reported in a previous study[8]. This may be attributed to the

harsh filtration of SNPs in the previous study and the overestimation of ROH longer than 1Mb. Two possibilities may explain this phenomenon: 1) the Chinese population in Taiwan Province has ever been extensively inbred, but this situation has gradually improved, and the long ROH fragments have been broken by recombination over generations; and 2) this was a descendant of some highly inbred individuals translocated by humans from mainland China to the Taiwan Province. The repeated mating with the native Taiwan pangolin population broke the long ROH segments into small fragments. However, we cannot rule out that the Taiwan population may still face serious survival risks, and more Taiwan Chinese pangolin individuals need to be added to the analysis to draw a clear conclusion.

The derived mutational load in all five pangolin populations was greater than Amur tiger and South China tiger populations, even with their lower inbreeding levels[37]. For the Chinese pangolin, the MpenC population had the highest proportion of mutational load. We hypothesized that the MpenC population represents an ancient and isolated Chinese pangolin population in Yunnan Province and is less disturbed by human activity, but has accumulated a large number of mutational load over its evolutionary history. We detected a stronger drift in the MpenC population, which can cause reduced efficacy of purifying selection to remove deleterious mutations[11]. In the Malayan pangolin, MjavA population showed low genetic diversity and high inbreeding. Although the high inbreeding in the MjavA population may promote the purging of deleterious mutations, we still observed a large amount of DHMD in this population. The significantly lower frequency of LoF inside compare with outside the ROH regionsindicates that many strongly recessive deleterious mutations remain in the nonROH regions in a heterozygous state and have not been effectively removed. This may be explained by the less efficient genetic purging in the pangolin populations.

Novel implications for the global conservation of the two pangolin species

High-volume poaching and trafficking have resulted in the overexploitation of pangolins; thus, the wild population, particularly the Chinese and Malayan pangolins, has plummeted to near extinction[8, 19-22]. Although moderate inbreeding and genetic diversity for these two species indicate a fine intrinsic genetic status, the population differentiation (F_{ST}) among the Chinese and Malayan pangolin populations remains large, which was also reported in other studies [8, 39, 42], even larger than the genetic differences of many subsoecies [43-45]. Genome-wide risks in different populations of Chinese and Malayan pangolins are also different[8, 39]. Therefore, in the effort of protecting and conserving pangolins, addressing, and managing the issues of illicit poaching and trafficking are just as important as genetic rescue efforts. Implementing timely protective and conservation measures for both the Chinese and Malayan pangolins will contribute to facilitating genetic rescue initiatives. Notably, both the genetic diversity and inbreeding of the MjavA population and the Taiwan individual are much worse than those in other pangolin populations, suggesting that the MjavA population, as well as the Taiwan Chinese pangolins may suffer more serious survival pressures than other pangolin populations, and should receive more attention and protection.

Materials and Methods

Samples, Resequencing Data and Ethics Statements

The Chinese and Malayan pangolins used for genome assembly were wild individuals rescued by the Guangdong Wildlife Rescue Center. During a routine examination, 5 mL of blood was collected with an anticoagulant tube, immediately transferred to liquid nitrogen, and stored at -80°C. The sample collection, experiment, and research design were all approved by the Institutional Review Board of BGI (BGI-IRB E22017). We strictly adhered to the guidelines provided by the BGI-IRB for all procedures. The whole-genome sequencing data of 37 Chinese pangolin and 72 Malayan pangolin individuals were downloaded from the National Center for Biotechnology Information (NCBI) and the China National GeneBank DataBase (CNGBdb) for population genomic analysis in this study[8, 16, 39].

Nucleic Acid Extraction, Library Preparation, and Sequencing

Total genomic DNA was extracted using the DNeasy Blood & Tissue Kit (Qiagen, USA) for whole genome sequencing (WGS) library preparation. Total RNA was extracted from blood using Trizol reagent (Invitrogen, USA) from blood, and 250-300 bp reverse transcribed cDNA fragments were used for DNA library construction. Two Hi-C libraries were prepared with *Dpn*II restriction endonuclease. DNA libraries were subjected to the Illumina HiSeq X Ten platform (RRID:SCR_016385) at Novogene (Tianjin, China) for paired-end sequencing. For high-molecular-weight genomic DNA, the isolation was performed using the sodium dodecyl sulfate (SDS)-based method, and purification was carried out by the Qiagen Genomic Kit. A 15k library was constructed using high-quality DNA samples (main band > 30kb) and sequenced with the PacBio Sequel II platform at Novogene (Tianjin, China).

Genome Assembly and Assessment

To estimate the genome size, a total of ~100 Gb WGS short reads were used for analysis by the jellyfish (RRID:SCR_005491) (v2.3.1) [46]. The hifiasm (RRID:SCR_021069) [24] (v0.16.1) software was used to generate the primary genome with PacBio HiFi and Hi-C sequencing data. Hifiasm utilized Hi-C sequencing data to achieve chromosome-level phasing in a method that does not require parental data[25]. This process can also phase the primary contigs at the same time into two sets of haplotigs, representing the two haploid genomes of a diploid genome. Genome redundancy was removed by the software Purge_dups (RRID:SCR_021173) [47] (v1.2.5). Then, the Hi-C sequencing reads were re-mapped to the primary genomes, after quality control by the Juicer (RRID:SCR_017226) [48] (v1.5)and the *mem* algorithm of Burrows-Wheeler Aligner (BWA, v0.7.17; RRID:SCR_010910) [49, 50]. The 3d-DNA pipeline(RRID:SCR_017227) (v190716) was finally used to concatenate and review the primary scaffolds to chromosome-scale genomes[51]. We identified the X chromosome and Y-linked regions using SRY genes and WGS short reads and confirmed that the sequencing depth of the sex chromosomes was approximately half that of the autosomes.

The genome completeness was evaluated by BUSCO (RRID:SCR_015008) (v5.2.2) software using the vertebrata_odb10 data set [52]. We conducted a Merqury (RRID:SCR_022964) [53] (release 20200430) k-mer analysis and alignment of the whole genome sequencing reads to the reference genome to evaluate the accuracy of the genome assembly. Genome regions covered by PacBio long-read greater than 10-fold were considered accurately assembled regions[54]. The identification of syntenic blocks between pangolin genomes was primarily performed by the NUCmer program in MUMmer (RRID:SCR_018171) [55] (v4.0.0rc1), followed by filtration using the delta-filter program in MUMmer (v4.0.0rc1) with parameters "-i 90 -l 5000".

Genome Annotation.

Repeat elements in the genome were annotated using *de novo* and homology-based methods. *De novo* repeats were first annotated using the LTR finder (RRID:SCR_015247) [56] (v1.0.6) and RepeatModeler2 (RRID:SCR_015027) [57] (v2.0.1), and the identified repeats were then merged into the RepBase library as known elements. Transposable elements were identified and classified using RepeatMasker (RRID:SCR_012954) (v4.0.5) with a conserved BLASTN search against the RepBase library [58]. The RepeatProteinMask program in RepeatMasker (v4.0.5) was used to identify repeat proteins [58]. The tandem repeats were annotated using Tandem Repeats Finder[59] (v4.07). Protein-coding genes were annotated using *de novo*, homology-based, and transcript-based approaches after masking the repeat elements. For the *de novo* method, we used Augustus[60] (v3.0.3), GlimmerHMM (RRID:SCR_002654) [61] (v3.0.1), and SNAP (RRID:SCR_007936) [62] (v11/29/2013) to predict the gene models. For transcript-based prediction, the transcripts were mapped to the reference genome using HISAT2 (RRID:SCR_015530)[63] (v2.1.0) and then assembled using StringTie (RRID:SCR_016323) [64] (v1.3.3b) based on clean RNA-seq data. Homology-based gene annotation was performed by using Blastall[65] (v2.2.26) with an E-value cut-off of 1e-5 towith against

the protein sequences of *Homo sapiens*, *Mus musculus*, *Canis lupus familiaris*, and *Felis catus*. The final protein-coding gene set was generated using the MAKER (RRID:SCR_005309) [66] (v3.01.03) pipeline by combining high-quality homology-based, *de novo* and RNA-seq supported genes.

Functional annotation was performed by a BLAST (RRID:SCR_004870) (v2.13.0) search against the SwissProt, TrEMBL, and Kyoto Encyclopedia of Genes and Genomes (KEGG) databases with an E-value cut-off of 1e-5. InterProScan (RRID:SCR_005829) [67] (v5.52-86.0) was used to predict motifs, domains, and Gene Ontology (GO) terms. The tRNA genes were identified using tRNAscan-SE (RRID:SCR_008637) [68] (v1.3.1), whereas the snRNA and miRNA genes were detected by searching the reference sequences against the Rfam database (RRID:SCR_007891) (Release 12.0) using the BLAST (v2.13.0) and the program cmsearch from infernal (RRID:SCR_011809) (v1.1.1) software.

Detection of Structural Variants (SVs) in the Pangolin Genome

To identify sequence differences between the parental genomes, sequence alignment was performed using Mummer (v4.0.0rc1) with the parameters 'nucmer --maxmatch -c 500 -b 500 -l 100' [55]. SVs were detected based on the alignment results using SyRi (RRID:SCR_023008) [69] (v1.3). To verify the accuracy of the detected SVs, we aligned the PacBio long reads to the reference genome using BLAST (v2.13.0) to determine whether the reads crossed the breakpoints. Moreover, we extracted 300 bp of upstream/downstream flanking sequences for each breakpoint and manually verified them using DNBSEQ short reads by IGV (RRID:SCR_011793) [70] (v2.13.3) software. To identify gene loss in the haploid genome, we screened for pseudogenes interrupted by SVs using Mummer alignment and checked whether these genes had other copies across the entire genome.

Genome-wide Variant Calling and Quality Control

The BWA *mem* algorithm (v0.7.17) [49] was applied to map the whole-genome resequencing data of 72 Malayan pangolins and 37 Chinese pangolins to each of their reference genomes with default parameters. Sentieon (RRID:SCR_025615) [71] (v202010.01) was then used to sort, reorder, and deduplicate the alignment files for variant calling. Variants were detected for each individual using the Sentieon DNAseq Haplotyper pipeline, which is similar to the Genome Analysis Toolkit (GATK) HaplotypeCaller pipeline. Joint variant calling was performed using the Sentieon DNAseq GVCFtyper with all gVCF files to generate a VCF file. To prepare for downstream analysis, the variant set was filtered to remove InDels and multiallelic variants. For variant quality control, a stringent filtering step was performed using the following parameters: "QD <2.0 || FS > 60.0 || MQ < 40.0 || MQRankSum < -12.5 || ReadPosRankSum < -8.0". Additionally, we filtered SNPs that were missed in more than 20% of the individuals in a population. We used both the short-read assembled genome (SG) and long-read assembled genome (LG) as references to generate variant sets for downstream comparison.

Population Structure Analysis

Before we performed PCA, the VCF file was converted to PLINK format using PLINK software (RRID:SCR_001757) [72] (v1.90b6.10.Genome-wide complex trait analysis (GCTA)[73] (v1.92.2) software was used for PCA analysis using the default parameters. To construct a phylogenetic tree, vcf2phylip[74] (v2.7) was used to convert the VCF file into PHYLIP format. The best substitution model was then calculated using jModelTest[75] (v2.1.10), and the maximum likelihood phylogenetic tree was constructed using IQ-TREE[76] (v1.6.12) software with default parameters. ADMIXTURE[77] (v1.3.0) was used to determine the ancestry proportion with a specified number of clusters (K) ranging from 1 to 10. For this analysis, we used both the SG and LG as the reference genome to generate two groups of results.

Population Demography Inference

SMC++[78] (v1.5.1) was used to infer the historical changes in the effective population size of the varouus pangolin populations. The SMC++ results were visualized by scaling the time to real years using a generation time of one year and a mutation rate of $\mu = 1.47 \times 10^{-8}$[8, 16] for both the Malayan and Chinese pangolins. MSMC2 (RRID:SCR_023677)[79] (v2.1.1) was used to infer the changes in the effective population size over the evolutionary history with four randomly selected individuals from each pangolin population. SNPs were first phased by Beagle (RRID:SCR_001789) [80] (v5.1) and then subjected to MSMC2 for inference of the population history. We used the SG and LG as the reference genomes to generate two groups of results for comparison.

ROH and Genetic Diversity

To detect ROH fragments, multi-individual VCF files were converted into PLINK bfile format using the PLINK (RRID:SCR_001757) [72] (v1.90b6.10) software. The ROH was then detected using the PLINK[72] (v1.90b6.10) software with the parameters "--homozyg --homozyg-window-snp 20 --homozyg-kb 10 --homozyg-density 50" [81]. ROHs shorter than 100 kb were excluded from the downstream analysis. F_{ROH} was calculated as $F_{ROH} = L_{ROH}/L_{AUTOSOME}$, where L_{ROH} represents the total length of ROHs in each genome and $L_{AUTOSOME}$ represents the total length of the autosomes. Genome-wide genetic diversity (π) was calculated using vcftools[82] (v0.1.16) using the parameters "vcftools --gzvcf vcf.gz --window-pi 500000 -out result". Genome-wide heterozygosity was calculated using vcftools[82] (v0.1.16) with "vcftools --gzvcf vcf.gz --het --out result" parameters. For ROH and genetic diversity analysis, we used both the SG and LG as reference genomes for comparison, but only the results calculated based on the LG were used for further discussion.

Mutational Load and Genetic Purging Analysis

To identify the mutational load in the protein-coding genes, the variants were first annotated using ANNOVAR (RRID:SCR_012821) [83] (v20191024) and SnpEff[84] (v.5.0e). Variants annotated as stop gained, splice acceptor variant, or splice donor by SnpEff[84] were predicted to be loss-of-function (LoF) mutations. Nonsynonymous variants with Grantham Score ⩾150 were considered deleterious mutations (dnsSNP) [85]. To determine the derived allele, the Malayan pangolin genome was split into 100 bp reads and mapped to the Chinese pangolin genome. If an allele was found within the Malayan pangolin genome and simultaneously represented the major allele (with an allele frequency exceeding 50%) within the Chinese pangolin population, it was designated as the ancestral state within the Chinese pangolin genome[86]. The same approach was used to determine the ancestral state of variants of the Malayan pangolin.

The occurrence of the mutational load in the ROH and non-ROH regions (${}_{ROH}f$ and ${}_{nonROH}f$) for each individual genome was calculated by dividing the total number of deleterious mutations (N_m) within the ROH or non-ROH region by the number of synonymous mutations in the same region (S_{ROH} and S_{nonROH}) as follows:

$${}_{ROH}f = \frac{N_m}{S_{ROH}}$$

$${}_{nonROH}f = \frac{N_m}{S_{nonROH}}$$

To estimate the relative excess of deleterious mutations in one pangolin population compare with another, we performed the Rxy analysis for dnsSNP, missense mutations, LoF, and synonymous mutations between pairs of pangolin populations [10]. We calculated the Rxy value using the following formula:

$$L_X = \frac{\sum_{i \in C} (m_X^i / s_X^i)(1 - m_Y^i / s_Y^i)}{\sum_{i \in I} (m_X^i / s_X^i)(1 - m_Y^i / s_Y^i)}$$

$$R_{X/Y} = L_X/L_Y$$

Where m_X^i represents the count of derived alleles for the above mentioned mutations observed at each site (i) within one population (X) and m_Y^i represents that in another population (Y). s_X^i and s_Y^i represent the total number of alleles at each site (i) of the population (X or Y). C represents the above-mentioned category of protein-coding sites, whereas I denotes the intergenic sites. We used the jackknife method during the calculation to obtain a standard error measurement. If Rxy = 1, both populations have the same level of derived mutation load, whereas if Rxy < 1, then population Y has more derived load than X and vice versa if Rxy > 1.

Genomic Evolutionary Rate Profiling (GERP) Scores

It is difficult to estimate the genetic load without fitness data. Therefore, we calculated the relative mutational load for each individual genome. First, we screened the derived alleles distributed in the highly conserved genome region of the two pangolin species using the genomic evolutionary rate profiling scores (GERP) method. To calculate the GERP scores, we selected the genomes of 37 species (*Acinonyx jubatus*, *Bos taurus*, *Callithrix jacchus*, *Canis lupus*, *Cavia porcellus*, *Choloepus hoffmanni*, *Dasypus novemcinctus*, *Dipodomys ordii*, *Echinops telfairi*, *Equus caballus*, *Erinaceus europaeus*, *Felis catus*, *Homo sapiens*, *Loxodonta africana*, *Lynx canadensis*, *Mus musculus*, *Myotis lucifugus*, *Ochotona princeps*, *Oryctolagus cuniculus*, *Panthera pardus orientalis*, *Panthera tigris*, *Pan troglodytes*, *Prionailurus bengalensis*, *Procavia capensis*, *Pteropus vampyrus*, *Puma concolor*, *Rattus norvegicus*, *Sorex Araneus*, *Spermophilus tridecemlineatus*, *Tupaia belangeri*, *Tursiops truncates*, *Vicugna pacos*, *Manis javanica*, *Manis pentadactyla*, *Tamandua tetradactyla*, *Ovis aries*, and *Vulpes lagopus*) for screening ultra-conserved genomic regions. We split these genomes into 100 bp reads to generate fastq files. Then, we respectively aligned these fastq files to the Malayan and Chinese pangolin genomes using the *mem* algorithm in BWA (v0.7.17-r1188) with "-B 3" parameter. GERP (RRID:SCR_000563) scores were then calculated using the gerpcol program from the GERP++[87] software based on the above-mentioned alignment files. In general, low GERP scores (<1) usually represent putatively neutral genome regions, whereas high GERP scores (>1) indicate conserved genome regions [11]. Derived alleles in more conserved genome regions (those with higher GERP scores), these alleles are likely to be more deleterious. In this study, we calculated the relative mutational load with mutations having the top 0.1% GERP scores to select more deleterious alleles distributed in highly conserved genome regions [7]. The relative mutational load was calculated by the following formula: the sum of all homozygous and heterozygous derived alleles multiplied by their conservation score over the total number of derived alleles, with the heterozygous counted as one allele and homozygous sites counted as two alleles [11]. Therefore, a higher relative mutation load indicates that a relatively larger proportion of derived alleles may be found in more conserved genomic regions.

Site-Frequency Spectrum (SFS) Analysis

For SFS analysis, we calculated the frequency of each type of mutations at every site in the various pangolin populations. We considered intergenic variants as neutral, whereas LoF and missense variants were consider putatively damaging mutations[7]. For SFS in each pangolin population, we subsampled nonmissing derived alleles from each locus for calculation[7]. Fixed (frequency=1) and missing (frequency=0) alleles were included in the SFS for the five populations. We used LG as the reference genome for SFS analysis.

Additional Files

Supplementary Fig. S1. The heatmap represents the contact matrices generated by aligning the Hi-C data to the haploid chromosome-level Chinese pangolin (a) and Malayan pangolin (b) genomes.

Supplementary Fig. S2. Estimated genome size of the Chinese and Malayan pangolin genomes by using K-mer frequency analysis with k-mer size of 17. (a) The K-mer spectra of the Chinese pangolin genome. (b) The K-mer spectra of the Malayan pangolin genome.

Supplementary Fig. S3. Sequencing depths of each pseudo-chromosome. (a) Sequencing depths of the 19 autosomes, X chromosome, and Y chromosome in the Chinese pangolin genome. (b) Sequencing depths of the 18 autosomes, X chromosome, and Y chromosome in the Malayan pangolin genome.

Supplementary Fig. S4. Comparisons of LG and SG on PCA analysis for both Chinese and Malayan pangolin populations. (a) PCA analysis of Malayan pangolin populations based on SG. (b) PCA analysis of Malayan pangolin populations based on LG. (c) PCA analysis of Chinese pangolin populations based on SG. (d) PCA analysis of Chinese pangolin populations based on LG.

Supplementary Fig. S5. Comparisons of LG and SG on the construction of phylogenetic tree for both Chinese and Malayan pangolin populations. (a) Phylogenetic tree constructed based on the SG for Malayan pangolin populations. (b) Phylogenetic tree constructed based on the LG for Malayan pangolin populations. (c) Phylogenetic tree constructed based on the SG for Chinese pangolin populations. (d) Phylogenetic tree constructed based on the LG for Chinese pangolin populations.

Supplementary Fig. S6. Comparisons of LG and SG on the admixture analysis for both Chinese and Malayan pangolin populations. (a) Genome-wide admixture analysis for three populations of Chinese pangolin based on the SG. (b) Genome-wide admixture analysis for three populations of Chinese pangolin based on the LG. (c) Genome-wide admixture analysis for two populations of Malayan pangolin based on the SG. (d) Genome-wide admixture analysis for two populations of Malayan pangolin based on the LG.

Supplementary Fig. S7. Comparison of LG and SG assembly for analyzing population history and separation in Chinese and Malayan pangolin populations. (a) The dynamics of effective population size of Malayan pangolin populations analyzed based on the SG. (b) The population size dynamics of Malayan pangolin populations analyzed based on the LG. (c) The population size dynamics of Chinese pangolin populations analyzed based on the SG. (d) The population size dynamics of Chinese pangolin populations analyzed based on the LG. (e) The divergence time between two populations of Malayan pangolin estimated based on the SG. (f) The divergence time between two populations of Malayan pangolin estimated based on the LG. (g) The divergence time among three populations of Chinese pangolin estimated based on the SG. (h) The divergence time among three populations of Chinese pangolin estimated based on the LG.

Supplementary Fig. S8. (a) The population-level ROH distribution in three Chinese pangolin populations. (b) The population-level ROH distribution in two Malayan pangolin populations.

Supplementary Fig. S9. (a)The individual-level distribution of ROH larger than 100Kb in Chinese pangolin genomes. (b) The individual-level distribution of ROH larger than 100Kb in Malayan pangolin genomes. Each row represents an individual.

Supplementary Fig. S10. Total deleterious nonsynonymous SNP (dnsSNP) (a) and missense (b) mutations at the individual level were assessed across five populations of Chinese and Malayan pangolins. The number of individual-level homozygous LoF(c), dnsSNP (d) and missense (e) mutations across the five pangolin populations. (f) The ratio of homozygous missense mutations and dnsSNPs in Chinese and Malayan pangolin populations was calculated as the formula of : 2 × homozygous sites / (2 × homozygous sites + heterozygous site).

Supplementary Fig. S11. Dot plot showed the occurrence of dnsSNPs (a) and missense mutations (b) in the two Malayan pangolin populations calculated as the ratio of the number of mutational load to synonymous mutations in the ROH regions (ROH*f*) or nonROH regions (nonROH*f*) across the genome. The ratio of ROH*f* to nonROH*f* for dnsSNP and missense mutations in Malayan pangolin populations (c).Dot plot showed the occurrence of dnsSNPs (d) and missense mutations (e) in the three Chinese pangolin populations calculated as the ratio of the number of

mutational load to synonymous mutations in the ROH regions or nonROH regions across the genome.The ratio of ROH*f* to nonROH*f* for dnsSNP and missense mutations inChinese pangolin populations (f)

Supplementary Fig. S12. (a) The Rxy ratio of derived alleles in MjavA (x) to MjavB (y) for dnsSNP, synonymous, missense and LoF. The Rxy <1 indicated the population y has more derived alleles than population x. (b) The Rxy ratio of derived alleles in x population to y population (x/y: MpenB/MpenC; MpenA/MpenC; MpenA/MpenB) for dnsSNP, synonymous, missense and LoF. The Rxy <1 indicated the population y has more derived alleles than population x.

Supplementary Fig. S13. Comparison of genome-wide π of the Malayan pangolin and Chinese pangolin with other endangered species. Abbreviations along the X-axis are as follows: CMA: Brown eared pheasant (*Crossoptilon mantchuricum*), ASI: Chinese alligator (*Alligator sinensis*), PTA: Amur tiger (*Panthera tigris altaica*), AFU: Red panda (*Ailurus fulgens*), AME: Giant panda (*Ailuropoda melanoleuca*), MJ: Malayan pangolin (*Manis javanica*), MBE: Dwarf musk deer (*Moschus berezovskii*), MP: Chinese pangolin (*Manis pentadactyla*).

Supplementary Fig. S14. K-mer spectra plot estimated by Merqury. (a) K-mer spectra plot for the haplotype-resolved chromosome-level genome of MP. (b) K-mer spectra plot for the haplotype-resolved chromosome-level genome of MJ.

Supplementary Fig. S15. The sequencing depths of the two groups of haplotigs in both MJ and MP genomes. (a, b) Alignment with DNBSEQ read. (c, d) Alignment with PacBio HiFi reads.

Supplementary Fig. S16. Pairwise differences observed between the haploid genomes of Malayan and Chinese pangolin. The sliding window was set to be 100 bp.

Supplementary Fig. S17. (a) Brief introduction and circos diagram of the two pangolin genomes. (b) Structural rearrangements between the two haplotypes of each chromosome in Malayan pangolin and Chinese pangolin genomes. (c) Dot plot between MJH1 (x-axis) and MJH2 (y-axis), plotted by pafCoordsDotPlotly. (d) Dot plot between MPH2 (x-axis) and MPH1 (y-axis), plotted by pafCoordsDotPlotly.

Supplementary Fig. S18. Validation of structural variants through contig mapping. Contigs are mapped to both the haploid genomes to verify structural variants and visualized by the IGV software. (a) The correct structural variants between the haploid genomes. The breakpoints of the SVs could be covered by complete contigs in both of the haploid genomes. (b) The incorrect structural variants between the haploid genomes. The breakpoints of the SVs in one of the two haploid genomes could not be covered by the complete contig. In the IGV screenshot, the gray bar represents the contig spanning over the SVs, and the contig corresponding SV regions were marked as the red box. All structural variants have been verified, and several are randomly displayed here.

Supplementary Fig. S19. (a) The GO enrichment result of genes distributed in structural variants of Malayan pangolin. (b) The KEGG enrichment result of genes distributed in structural variants of Malayan pangolin. (c) The GO enrichment result of genes distributed in structural variants of Chinese pangolin. (d) The KEGG enrichment result of genes distributed in structural variants of Chinese pangolin.

Supplementary Table S1. Summarized sample information in this study.

Supplementary Table S2. Information of five populations in Chinese and Malayan pangolin included in this study.

Supplementary Table S3. Statistics of genome assemblies for the Chinese pangolin and Malayan pangolin.

Supplementary Table S4. The length of each chromosome in the Chinese pangolin and Malayan pangolin genomes.

Supplementary Table S5. The overall statistics of repeats in the Chinese and Malayan pangolin genomes.

Supplementary Table S6. Statistics of the annotated genes in Chinese and Malayan pangolin genomes.

Supplementary Table S7. BUSCO analysis of genome assemblies and gene sets in this study.

Supplementary Table S8. Statistics of functional annotation for the Malayan and Chinese pangolin's gene sets.

Supplementary Table S9. Statistics of ncRNA annotation.

Supplementary Table S10. SNPs number, genetic diversity (π), heterozygosity and SNP density calculated based on LG and SG in Malayan and Chinese pangolin populations.

Supplementary Table S11. The comparison of the SG and LG for estimating ROH in Malayan pangolin and Chinese pangolin populations

Supplementary Table S12. The count and length of ROH fragments in the five populations of Malayan pangolin and Chinese pangolin.

Supplementary Table S13. The F_{ROH} in the five populations of Malayan pangolin and Chinese pangolin.

Supplementary Table S14. The total count of dnsSNPs, LoF, missense and synonymous SNPs in various populations, along with the average number of dnsSNPs, LoF, missense, and synonymous SNPs at the individual level, across different populations of the Malayan and Chinese pangolins.

Supplementary Table S15. The individual level mutational load estimates in all Chinese pangolin and Malayan pangolin populations.

Supplementary Table S16. Comparison of genome-wide nucleotide diversity (π) of the Malayan pangolin and Chinese pangolin with reference to other endangered species on the IUCN Red List.

Supplementary Table S17. Quality assessment of the MP and MJ genomes by the Merqury software.

Supplementary Table S18. The mapping rates of four types of sequencing data to genomes assembled in this study.

Supplementary Table S19. Comparison of the assembly statistics with the previously published Chinese pangolin and Malayan pangolin genomes.

Supplementary Table S20. Pairwise differences observed in comparisons between the haplotype genomes of Chinese and Malayan pangolin, the sliding window was set to be 100bp.

Supplementary Table S21. Chromosomal structural variants (>50bp) of MJH1 and MJH2, and of MPH1 and MPH2.

Supplementary Table S22. Genes distributed in the structural variants of MJ and MP genomes.

Supplementary Table S22. The KEGG enrichment result of genes distributed in the structural variants of MJ/MP.

Supplementary Table S23. The GO enrichment result of genes distributed in the structural variants of MJ/MP.

Supplementary Table S24. Functional description of pseudogenes interrupted by structural variants.

Data Availability

Bioproject and biosample for the genomic data of this study were submitted to NCBI under accession numbers PRJNA1114675. The accession number of genomes are GCA_040802235.1 and GCA_040802205.1. The data that support the findings in this study also have been deposited into CNGB Sequence Archive (CNSA) [88]of China National GeneBank DataBase (CNGBdb) [89] with accession number CNP0004630. The resequencing data in this study were retrieved from earlier studies (37 Chinese pangolins: CNP0001723, CNGBdb; PRJNA529540 and PRJNA20331, NCBI Read Archive. 72 Malayan pangolins: PRJNA529540, NCBI Read Archive) [8, 16, 39]. All additional supporting data are available in the *GigaScience* repository, GigaDB [90-92].

Abbreviations

WGS: Whole genome sequence; RNA-seq: RNA sequence; BUSCO: Benchmarking Universal Single-Copy Orthologs; HiFi: High fidelity; Hi-C: High-throughput/resolution chromosome conformation capture; PCA: Principal component analysis; SNP: Single-nucleotide polymorphism; TE: Transposable element; KEGG: Kyoto Encyclopedia of Genes and Genomes; GO: Gene Ontology; GATK: Genome Analysis Toolkit; SG: Short-read assembled genome; LG: Long-read assembled genome; ROH: Runs of homozygosity; LoF: Loss-of-function; dnsSNP: Deleterious nonsynonymous mutation; GERP: Genomic evolutionary rate profiling; SFS: Site-Frequency Spectrum.

Author Contributions

Tianming Lan, Huan Liu, Yinping Tian and Yan Hua conceived and initiated the project. Yan Hua, Jun Li, Fanghui Hou, Yue Ma,Tengcheng Que, Wenjian Liu and Kai Wang collected the samples. Jin Chen, Chuanling Yin and Yinping Tian performed DNA isolation, library preparation and genome sequencing. Haimeng Li, Minhui Shi, Boyang Liu and Qing Wang assembled the genomes and conducted the genomics analysis. Tianming Lan coordinated the genomic analysis. Tianming Lan and Haimeng Li wrote the manuscript. Sunil Kumar Sahu, Minhui Shi, Yanling Xia and Boyang Liu reviewed and edited the manuscript. Tianming Lan and Yan Hua made important contributions to the revision of the manuscript. All the authors read and approved the final manuscript.

Competing interests

The authors declare no competing interests.

Acknowledgments

This study was supported by the National Key Program of Research and Development, Ministry of Science and Technology (Grant No. 2022YFF1301500), the Guangdong Provincial Key Laboratory of Genome Read and Write (Grant No. 2017B030301011) and the Start-up Scientific Foundation of Northeast Forestry University (60201524043). This work was also supported by China National GeneBank (CNGB). We thank Kinanti Seraphina Larasati and Shiqing Wang for their help in reviewing and polishing the language. We thank Hui Liu and Tian Xia from the Shenzhen Safari Park Co., Ltd. for assisting with sample collection. Finally, we thank all the researchers (Shiqing Wang, Dongyi Yang, Jieyao Yu, Jiale Fan, Yuting Huang, Yingna Zhou, Tianlu Liu, Jiatong Cheng, Chen Lin and Shiyu Liu) involved in sample collection, genome sequencing and analysis.

References

1. Formenti G, Theissinger K, Fernandes C, Bista I, Bombarely A, Bleidorn C, et al. The era of reference genomes in conservation genomics. Trends in ecology & evolution. 2022;37 3:197-202. doi:10.1016/j.tree.2021.11.008.
2. Kitts PA, Church DM, Thibaud-Nissen F, Choi J, Hem V, Sapojnikov V, et al. Assembly: a resource for assembled genomes at NCBI. Nucleic acids research. 2016;44 D1:D73-80. doi:10.1093/nar/gkv1226.
3. Ouborg NJ, Pertoldi C, Loeschcke V, Bijlsma RK and Hedrick PW. Conservation genetics in transition to conservation genomics. Trends in genetics : TIG. 2010;26 4:177-87. doi:10.1016/j.tig.2010.01.001.
4. Brandies P, Peel E, Hogg CJ and Belov K. The Value of Reference Genomes in the Conservation of Threatened Species. Genes. 2019;10 11 doi:10.3390/genes10110846.
5. Frankham R. Genetic rescue of small inbred populations: meta-analysis reveals large and consistent benefits of gene flow. Molecular ecology. 2015;24 11:2610-8. doi:10.1111/mec.13139.
6. Weeks AR, Heinze D, Perrin L, Stoklosa J, Hoffmann AA, van Rooyen A, et al. Genetic rescue increases fitness and aids rapid recovery of an endangered marsupial population. Nature communications. 2017;8 1:1071. doi:10.1038/s41467-017-01182-3.
7. Khan A, Patel K, Shukla H, Viswanathan A, van der Valk T, Borthakur U, et al. Genomic evidence for inbreeding depression and purging of deleterious genetic variation in Indian tigers. Proceedings of the National Academy of Sciences of the United States of America. 2021;118 49 doi:10.1073/pnas.2023018118.
8. Hu JY, Hao ZQ, Frantz L, Wu SF, Chen W, Jiang YF, et al. Genomic consequences of population decline in critically endangered pangolins and their demographic histories. National science review. 2020;7 4:798-814. doi:10.1093/nsr/nwaa031.
9. von Seth J, Dussex N, Diez-Del-Molino D, van der Valk T, Kutschera VE, Kierczak M, et al. Genomic insights into the conservation status of the world's last remaining Sumatran rhinoceros populations. Nature communications. 2021;12 1:2393. doi:10.1038/s41467-021-22386-8.
10. Xue Y, Prado-Martinez J, Sudmant PH, Narasimhan V, Ayub Q, Szpak M, et al. Mountain gorilla genomes reveal the impact of long-term population decline and inbreeding. Science. 2015;348 6231:242-5. doi:10.1126/science.aaa3952.
11. Nicolas D, Tom vdV, Hernán E. M, Christopher W. W, David D-d-M, Johanna vS, et al. Population genomics of the critically endangered kākāpō. Cell Genomics. 2021;1 1:100002. doi:10.1016/j.xgen.2021.100002.
12. Saremi NF, Supple MA, Byrne A, Cahill JA, Coutinho LL, Dalen L, et al. Puma genomes from North and South America provide insights into the genomic consequences of inbreeding. Nature communications. 2019;10 1:4769. doi:10.1038/s41467-019-12741-1.
13. Xie HX, Liang XX, Chen ZQ, Li WM, Mi CR, Li M, et al. Ancient Demographics Determine the Effectiveness of Genetic Purging in Endangered Lizards. Molecular biology and evolution. 2022;39 1 doi:10.1093/molbev/msab359.
14. Hua L, Gong S, Wang F, Li W, Ge Y, Li X, et al. Captive breeding of pangolins: current status, problems and future prospects. ZooKeys. 2015; 507:99-114. doi:10.3897/zookeys.507.6970.
15. Kondrashov P and Agadjanian AK. A nearly complete skeleton ofErnanodon(Mammalia,

Palaeanodonta) from Mongolia: morphofunctional analysis. Journal of Vertebrate Paleontology. 2012;32 5:983-1001. doi:10.1080/02724634.2012.694319.

16. Choo SW, Rayko M, Tan TK, Hari R, Komissarov A, Wee WY, et al. Pangolin genomes and the evolution of mammalian scales and immunity. Genome research. 2016;26 10:1312-22.
17. Ferreira-Cardoso S, Billet G, Gaubert P, Delsuc F and Hautier L. Skull shape variation in extant pangolins (Pholidota: Manidae): allometric patterns and systematic implications. Zoological Journal of the Linnean Society. 2019; doi:10.1093/zoolinnean/zlz096.
18. Heinrich S, Wittman TA, Ross JV, Shepherd CR, Challender DWS and Cassey P. THE GLOBAL TRAFFICKING OF PANGOLINS: A comprehensive summary of seizures and trafficking routes from 2010–2015. Petaling Jaya: TRAFFIC, Southeast Asia Regional Office. 2017.
19. Zhang F, Wu S and Cen P. The past, present and future of the pangolin in Mainland China. Global Ecology and Conservation. 2022;33:e01995. doi:10.1016/j.gecco.2021.e01995.
20. Challender DWS, Harrop SR and MacMillan DC. Understanding markets to conserve trade-threatened species in CITES. Biological Conservation. 2015;187:249-59. doi:10.1016/j.biocon.2015.04.015.
21. Nijman V, Zhang MX and Shepherd CR. Pangolin trade in the Mong La wildlife market and the role of Myanmar in the smuggling of pangolins into China. Global Ecology and Conservation. 2016;5:118-26. doi:10.1016/j.gecco.2015.12.003.
22. Cheng W, Xing S and Bonebrake TC. Recent Pangolin Seizures in China Reveal Priority Areas for Intervention. Conservation Letters. 2017;10 6:757-64. doi:10.1111/conl.12339.
23. Zhang F, Yu Y, Wu S, Mahmood A, Yu J and Min Y. Reducing Pangolin Demand by Understanding Motivations for Human Consumption in Guangdong, China. Frontiers in Ecology and Evolution. 2020;8 doi:10.3389/fevo.2020.574161.
24. Cheng H, Concepcion GT, Feng X, Zhang H and Li H. Haplotype-resolved de novo assembly using phased assembly graphs with hifiasm. Nature methods. 2021;18 2:170-5.
25. Cheng H, Jarvis ED, Fedrigo O, Koepfli K-P, Urban L, Gemmell NJ, et al. Haplotype-resolved assembly of diploid genomes without parental data. Nature Biotechnology. 2022;40 9:1332-5.
26. Cao P, Dai Q, Deng C, Zhao X, Qin S, Yang J, et al. Genome-wide signatures of mammalian skin covering evolution. Science China Life Sciences. 2021;64 10:1765-80.
27. Damas J, Corbo M, Kim J, Turner-Maier J, Farré M, Larkin DM, et al. Evolution of the ancestral mammalian karyotype and syntenic regions. Proceedings of the National Academy of Sciences. 2022;119 40:e2209139119.
28. Heighton SP, Allio R, Murienne J, Salmona J, Meng H, Scornavacca C, et al. Pangolin genomes offer key insights and resources for the world's most trafficked wild mammals. bioRxiv. 2023; doi:10.1101/2023.02.16.528682.
29. Yan D, Luo X, Tang J, Xu S, Huang K, Wang X, et al. High-Quality Genomes of Pangolins: Insights into the Molecular Basis of Scale Formation and Adaption to Myrmecophagous Diet. Molecular biology and evolution. 2023;40 1 doi:10.1093/molbev/msac262.
30. Nie W, Wang J, Su W, Wang Y and Yang F. Chromosomal rearrangements underlying karyotype differences between Chinese pangolin (Manis pentadactyla) and Malayan pangolin (Manis javanica) revealed by chromosome painting. Chromosome Res. 2009;17 3:321-9. doi:10.1007/s10577-009-9027-0.

31. Rhie A, McCarthy SA, Fedrigo O, Damas J, Formenti G, Koren S, et al. Towards complete and error-free genome assemblies of all vertebrate species. Nature. 2021;592 7856:737-46.
32. Wang Q, Lan T, Li H, Sahu SK, Shi M, Zhu Y, et al. Whole-genome resequencing of Chinese pangolins reveals a population structure and provides insights into their conservation. Communications biology. 2022;5 1:821. doi:10.1038/s42003-022-03757-3.
33. Challender DW, Nash HC and Waterman C. Pangolins: science, society and conservation. Academic Press; 2019.
34. Wang Q, Lan T, Li H, Sahu SK, Shi M, Zhu Y, et al. Whole-genome resequencing of Chinese pangolins reveals a population structure and provides insights into their conservation. Communications Biology. 2022;5 1:821.
35. Wei S, Fan H, Zhou W, Huang G, Hua Y, Wu S, et al. Conservation genomics of the critically endangered Chinese pangolin. Science China Life Sciences. 2024:1-11.
36. Henn BM, Botigué LR, Bustamante CD, Clark AG and Gravel S. Estimating the mutation load in human genomes. Nature Reviews Genetics. 2015;16 6:333-43. doi:10.1038/nrg3931.
37. Zhang L, Lan T, Lin C, Fu W, Yuan Y, Lin K, et al. Chromosome-scale genomes reveal genomic consequences of inbreeding in the South China tiger: A comparative study with the Amur tiger. Molecular ecology resources. 2022; doi:10.1111/1755-0998.13669.
38. Yang S, Lan T, Zhang Y, Wang Q, Li H, Dussex N, et al. Genomic investigation of the Chinese alligator reveals wild-extinct genetic diversity and genomic consequences of their continuous decline. Molecular ecology resources. 2022; doi:10.1111/1755-0998.13702.
39. Wang Q, Lan T, Li H, Sahu SK, Shi M, Zhu Y, et al. Whole-genome resequencing of Chinese pangolins reveals a population structure and provides insights into their conservation. Communications Biology. 2022;5 1:821. doi:10.1038/s42003-022-03757-3.
40. Zhao S, Zheng P, Dong S, Zhan X, Wu Q, Guo X, et al. Whole-genome sequencing of giant pandas provides insights into demographic history and local adaptation. Nature genetics. 2013;45 1:67-71. doi:10.1038/ng.2494.
41. Kuang WM, Ming C, Li HP, Wu H, Frantz L, Roos C, et al. The Origin and Population History of the Endangered Golden Snub-Nosed Monkey (Rhinopithecus roxellana). Molecular biology and evolution. 2019;36 3:487-99. doi:10.1093/molbev/msy220.
42. Hu J, Roos C, Lv X, Kuang W and Yu L. Molecular Genetics Supports a Potential Fifth Asian Pangolin Species (Mammalia, Pholidota, Manis). Zoological science. 2020;37 6:538-43. doi:10.2108/zs200084.
43. Guang X, Lan T, Wan Q-H, Huang Y, Li H, Zhang M, et al. Chromosome-scale genomes provide new insights into subspecies divergence and evolutionary characteristics of the giant panda. Science Bulletin. 2021;66 19:2002-13. doi:10.1016/j.scib.2021.02.002.
44. Pecnerova P, Garcia-Erill G, Liu X, Nursyifa C, Waples RK, Santander CG, et al. High genetic diversity and low differentiation reflect the ecological versatility of the African leopard. Current biology : CB. 2021;31 9:1862-71 e5. doi:10.1016/j.cub.2021.01.064.
45. Carneiro M, Albert FW, Afonso S, Pereira RJ, Burbano H, Campos R, et al. The genomic architecture of population divergence between subspecies of the European rabbit. PLoS genetics. 2014;10 8:e1003519. doi:10.1371/journal.pgen.1003519.
46. Marçais G and Kingsford C. A fast, lock-free approach for efficient parallel counting of

occurrences of k-mers. Bioinformatics. 2011;27 6:764-70.
47. Guan D, McCarthy SA, Wood J, Howe K, Wang Y and Durbin R. Identifying and removing haplotypic duplication in primary genome assemblies. Bioinformatics. 2020;36 9:2896-8.
48. Durand NC, Shamim MS, Machol I, Rao SS, Huntley MH, Lander ES, et al. Juicer provides a one-click system for analyzing loop-resolution Hi-C experiments. Cell systems. 2016;3 1:95-8.
49. Li H. Aligning sequence reads, clone sequences and assembly contigs with BWA-MEM. arXiv:13033997 [q-bioGN]. 2013;0 0:3.
50. Li H and Durbin R. Fast and accurate long-read alignment with Burrows–Wheeler transform. Bioinformatics. 2010;26 5:589-95.
51. Dudchenko O, Batra SS, Omer AD, Nyquist SK, Hoeger M, Durand NC, et al. De novo assembly of the Aedes aegypti genome using Hi-C yields chromosome-length scaffolds. Science. 2017;356 6333:92-5.
52. Manni M, Berkeley MR, Seppey M, Simão FA, Zdobnov EM and Kelley J. BUSCO Update: Novel and Streamlined Workflows along with Broader and Deeper Phylogenetic Coverage for Scoring of Eukaryotic, Prokaryotic, and Viral Genomes. Molecular biology and evolution. 2021;38 10:4647-54. doi:10.1093/molbev/msab199.
53. Rhie A, Walenz BP, Koren S and Phillippy AM. Merqury: reference-free quality, completeness, and phasing assessment for genome assemblies. Genome biology. 2020;21 1 doi:10.1186/s13059-020-02134-9.
54. Qi W, Lim YW, Patrignani A, Schlapfer P, Bratus-Neuenschwander A, Gruter S, et al. The haplotype-resolved chromosome pairs of a heterozygous diploid African cassava cultivar reveal novel pan-genome and allele-specific transcriptome features. GigaScience. 2022;11:giac028. doi:10.1093/gigascience/giac028.
55. Marçais G, Delcher AL, Phillippy AM, Coston R, Salzberg SL and Zimin A. MUMmer4: A fast and versatile genome alignment system. PLoS computational biology. 2018;14 1:e1005944.
56. Xu Z and Wang H. LTR_FINDER: an efficient tool for the prediction of full-length LTR retrotransposons. Nucleic Acids Res. 2007;35 Web Server issue:W265-8. doi:10.1093/nar/gkm286.
57. Flynn JM, Hubley R, Goubert C, Rosen J, Clark AG, Feschotte C, et al. RepeatModeler2 for automated genomic discovery of transposable element families. Proceedings of the National Academy of Sciences of the United States of America. 2020;117 17:9451-7. doi:10.1073/pnas.1921046117.
58. Chen N. Using Repeat Masker to identify repetitive elements in genomic sequences. Current protocols in bioinformatics. 2004;5 1:4.10. 1-4.. 4.
59. Benson G. Tandem repeats finder: a program to analyze DNA sequences. Nucleic acids research. 1999;27 2:573-80. doi:10.1093/nar/27.2.573.
60. Stanke M, Steinkamp R, Waack S and Morgenstern B. AUGUSTUS: a web server for gene finding in eukaryotes. Nucleic acids research. 2004;32 Web Server issue:W309-12. doi:10.1093/nar/gkh379.
61. Majoros WH, Pertea M and Salzberg SL. TigrScan and GlimmerHMM: two open source ab initio eukaryotic gene-finders. Bioinformatics. 2004;20 16:2878-9. doi:10.1093/bioinformatics/bth315.

62. Korf I. Gene finding in novel genomes. BMC bioinformatics. 2004;5:59. doi:10.1186/1471-2105-5-59.

63. Kim D, Langmead B and Salzberg SL. HISAT: a fast spliced aligner with low memory requirements. Nature methods. 2015;12 4:357-60.

64. Pertea M, Pertea GM, Antonescu CM, Chang T-C, Mendell JT and Salzberg SL. StringTie enables improved reconstruction of a transcriptome from RNA-seq reads. Nature biotechnology. 2015;33 3:290-5.

65. Mount DW. Using the Basic Local Alignment Search Tool (BLAST). CSH protocols. 2007;2007:pdb top17. doi:10.1101/pdb.top17.

66. Campbell MS, Holt C, Moore B and Yandell M. Genome Annotation and Curation Using MAKER and MAKER-P. Current protocols in bioinformatics. 2014;48:4 11 1-39. doi:10.1002/0471250953.bi0411s48.

67. Jones P, Binns D, Chang H-Y, Fraser M, Li W, McAnulla C, et al. InterProScan 5: genome-scale protein function classification. Bioinformatics. 2014;30 9:1236-40. doi:10.1093/bioinformatics/btu031.

68. Lowe TM and Eddy SR. tRNAscan-SE: a program for improved detection of transfer RNA genes in genomic sequence. Nucleic acids research. 1997;25 5:955-64.

69. Goel M, Sun H, Jiao W-B and Schneeberger K. SyRI: finding genomic rearrangements and local sequence differences from whole-genome assemblies. Genome biology. 2019;20 1:1-13.

70. Robinson JT, Thorvaldsdottir H, Turner D, Mesirov JP. igv.js: an embeddable JavaScript implementation of the Integrative Genomics Viewer (IGV). Bioinformatics. 2023 Jan 1;39(1):btac830. doi: 10.1093/bioinformatics/btac830.

71. Freed D, Aldana R, Weber JA and Edwards JS. The Sentieon Genomics Tools-A fast and accurate solution to variant calling from next-generation sequence data. BioRxiv. 2017:115717.

72. Purcell S, Neale B, Todd-Brown K, Thomas L, Ferreira MA, Bender D, et al. PLINK: a tool set for whole-genome association and population-based linkage analyses. The American journal of human genetics. 2007;81 3:559-75.

73. Yang J, Lee SH, Goddard ME and Visscher PM. GCTA: a tool for genome-wide complex trait analysis. The American Journal of Human Genetics. 2011;88 1:76-82.

74. Ortiz E. vcf2phylip v2. 0: convert a VCF matrix into several matrix formats for phylogenetic analysis. URL https://doi org/105281/zenodo. 2019;2540861.

75. Darriba D, Taboada GL, Doallo R and Posada D. jModelTest 2: more models, new heuristics and parallel computing. Nature methods. 2012;9 8:772. doi:10.1038/nmeth.2109.

76. Nguyen LT, Schmidt HA, von Haeseler A and Minh BQ. IQ-TREE: a fast and effective stochastic algorithm for estimating maximum-likelihood phylogenies. Molecular biology and evolution. 2015;32 1:268-74. doi:10.1093/molbev/msu300.

77. Alexander DH, Novembre J and Lange K. Fast model-based estimation of ancestry in unrelated individuals. Genome research. 2009;19 9:1655-64.

78. Terhorst J, Kamm JA and Song YS. Robust and scalable inference of population history from hundreds of unphased whole genomes. Nature genetics. 2017;49 2:303-9.

79. Schiffels S and Durbin R. Inferring human population size and separation history from multiple genome sequences. Nature genetics. 2014;46 8:919-25. doi:10.1038/ng.3015.

80. Browning SR and Browning BL. Rapid and accurate haplotype phasing and missing-data inference for whole-genome association studies by use of localized haplotype clustering. The American Journal of Human Genetics. 2007;81 5:1084-97.
81. Dobrynin P, Liu S, Tamazian G, Xiong Z, Yurchenko AA, Krasheninnikova K, et al. Genomic legacy of the African cheetah, Acinonyx jubatus. Genome biology. 2015;16 1:1-20.
82. Danecek P, Auton A, Abecasis G, Albers CA, Banks E, DePristo MA, et al. The variant call format and VCFtools. Bioinformatics. 2011;27 15:2156-8.
83. Wang K, Li M and Hakonarson H. ANNOVAR: functional annotation of genetic variants from high-throughput sequencing data. Nucleic acids research. 2010;38 16:e164-e.
84. Cingolani P, Platts A, Wang LL, Coon M, Nguyen T, Wang L, et al. A program for annotating and predicting the effects of single nucleotide polymorphisms, SnpEff: SNPs in the genome of Drosophila melanogaster strain w1118; iso-2; iso-3. Fly. 2012;6 2:80-92.
85. Grantham R. Amino acid difference formula to help explain protein evolution. science. 1974;185 4154:862-4.
86. Feng S, Fang Q, Barnett R, Li C, Han S, Kuhlwilm M, et al. The genomic footprints of the fall and recovery of the crested ibis. Current Biology. 2019;29 2:340-9. e7.
87. Davydov EV, Goode DL, Sirota M, Cooper GM, Sidow A and Batzoglou S. Identifying a high fraction of the human genome to be under selective constraint using GERP++. PLoS computational biology. 2010;6 12:e1001025.
88. Guo X, Chen F, Gao F, Li L, Liu K, You L, et al. CNSA: a data repository for archiving omics data. Database. 2020;2020 doi:10.1093/database/baaa055.
89. Chen F, You L, Yang F, Wang L, Guo X, Gao F, et al. CNGBdb: China National GeneBank DataBase. Hereditas (Beijing). 2020;42:799-809. doi:10.16288/j.yczz.20-080.
90. Lan T, Tian Y, Shi M, Liu B, Lin Y, Xia Y, et al. Supporting data for "Enhancing inbreeding estimation and global conservation insights through HiFi assemblies of Chinese and Malayan pangolin" GigaScience Database. 2024. https://doi.org/10.5524/102632
91. L Lan T, Tian Y, Shi M, Liu B, Lin Y, Xia Y, et al. The high-quality reference genome of Chinese pangolin (Manis pentadactyla) GigaScience Database. 2024. https://doi.org/10.5524/102634
92. Lan T, Tian Y, Shi M, Liu B, Lin Y, Xia Y, et al. The high-quality reference genome of Malayan pangolin (Manis javanica) GigaScience Database. 2024. https://doi.org/10.5524/102635

Figure5

Click here to access/download;Figure;FIG5-2024.11.26.pdf

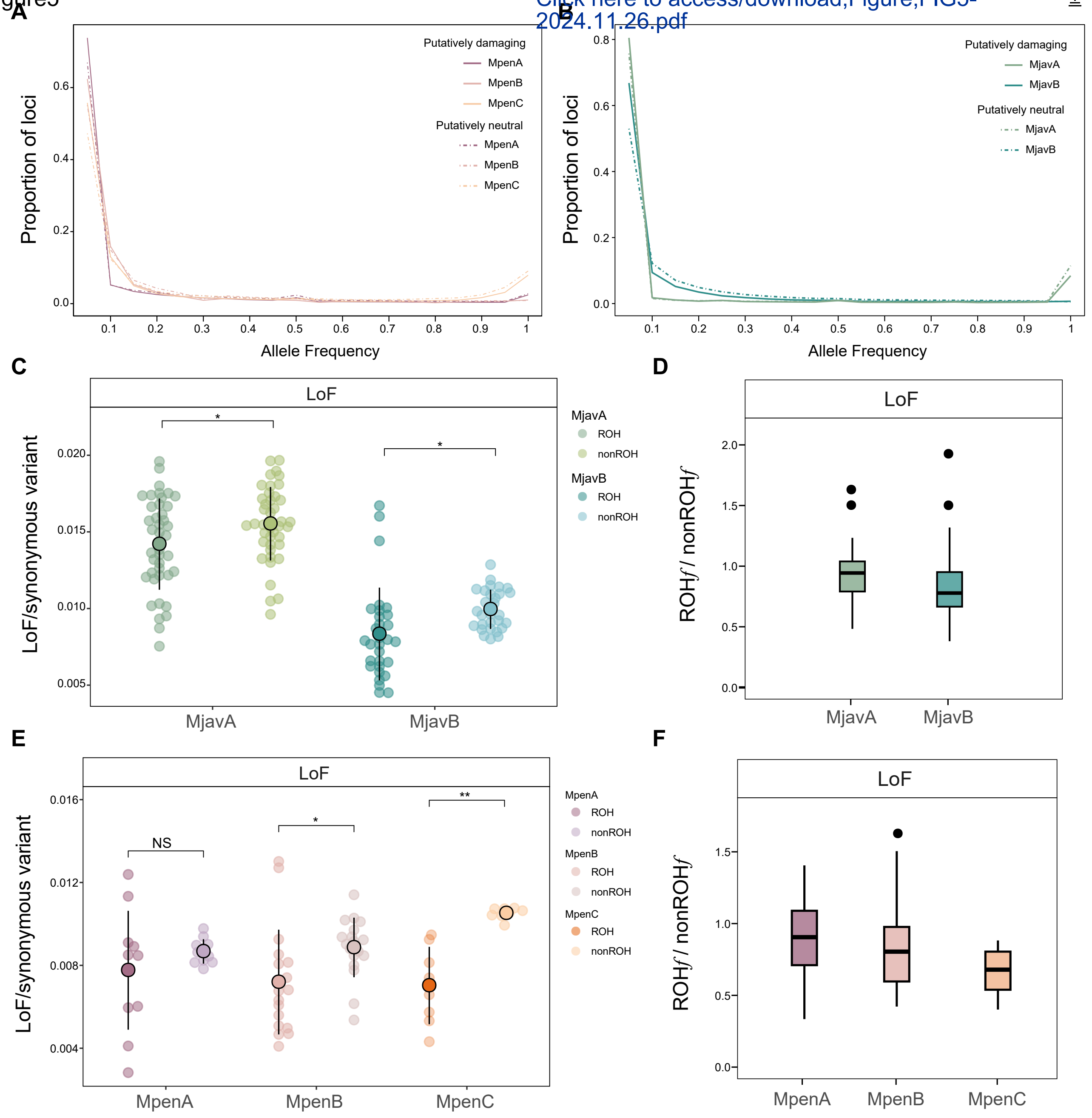

Figure1

Click here to access/download
Supplementary Material
FIG1-2024.11.26.svg

Click here to access/download
Supplementary Material
Supplemental Information-clean.11.26.docx

Click here to access/download;Personal Cover;Reviewer reports11.26.docx

Reviewer reports:

Reviewer #2: The authors have engaged positively and comprehensively with reviewer feedback, and I think the manuscript is a lot clearer as a consequence. I only have a few minor outstanding questions/comments.

MINOR COMMENTS

1. I suggest stressing the chromosome-level nature of the assembly in the title.

Response：Thank you. To address your suggestion, we have revised the title of our manuscript to better reflect the chromosome-level nature of our assembly. The new title is:

“Enhancing inbreeding estimation and global conservation insights through chromosome-level assemblies of the Chinese and Malayan pangolin”

2. Abstract - mention the HiC as well as HiFi reads.

Response：We have revised the description of Abstract in Lines 25-26.

3. L97: "all genomes (2 diploid assemblies and 4 haploid assemblies)" - are these not the same thing? Surely, the "diploid" assemblies are just the combined pair of haploid assemblies?

Response：Thank you. When we refer to "2 diploid assemblies and 4 haploid assemblies," we are indicating two different types of genome assemblies. Diploid assemblies are not simply a combined pair of haploid assemblies.

The diploid genome is the genome assembly without genome phasing, is a hybrid genome mixed with the genetic information from both parents, which represents the most common form of genome assembly. The haploid assembly, meticulously constructed with HiFi long reads and Hi-C data, is designed to distinguish the haplotype of each parent[1]. Therefore, the most important process for the assembly of the haploid genome is the phasing process to separate the genome segment from each parent. To make it clearer, I prepared a picture to show this:

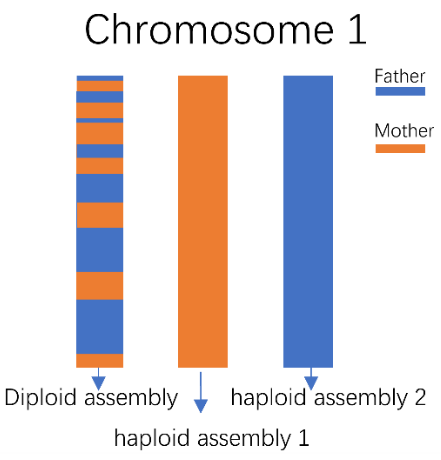

Response figure 1. Schematic diagram for the genomic sequence assembly

4. In Table 1, it would be useful to have the total assembly size and sequencing depth. Given the comparison with the short-read genomes, it would be informative to have their stats in the table too, which would really show where the improvements were made.

Response： Thank you for your suggestion to enhance Table 1 with additional statistical information. The current version of the Table 1 has been modified based on the previous reviewer's comments, and the comparison with other genomes has also been displayed in Table S19 following the reviewer's suggestions.

We have updated Table 1 to incorporate the total assembly size and sequencing depth.

5. I don't think it's strictly true to say that 10 PacBio HiFi reads ensures a reliable assembly. It could still be false duplications or contamination. 10 reads might also be a bit strict, depending on the depth of coverage. I can see what the authors are trying to do here, but wonder whether a slightly different descriptor could/should be found.

Response： The definition and calculation of the reliable assembly genome regions in the manuscript followed the previously published research[2]. Based on your suggestions, we have modified the calculation method for the reliable blocks. We consider the regions in the genome that are supported by two sequencing technologies (Hi-C and HiFi) as reliable blocks[3]. This method is more stringent, and we believe its reliability will meet the requirements of the analysis. In lines 107 and 109-110.

6. Figure 1. What are the colours in panel B? The details of Panel B are a bit lost. Having a full-width landscape plot with a different colour per chromosome might be clearer.

Response： Thanks for your comments. We have assigned a unique color to each chromosome.

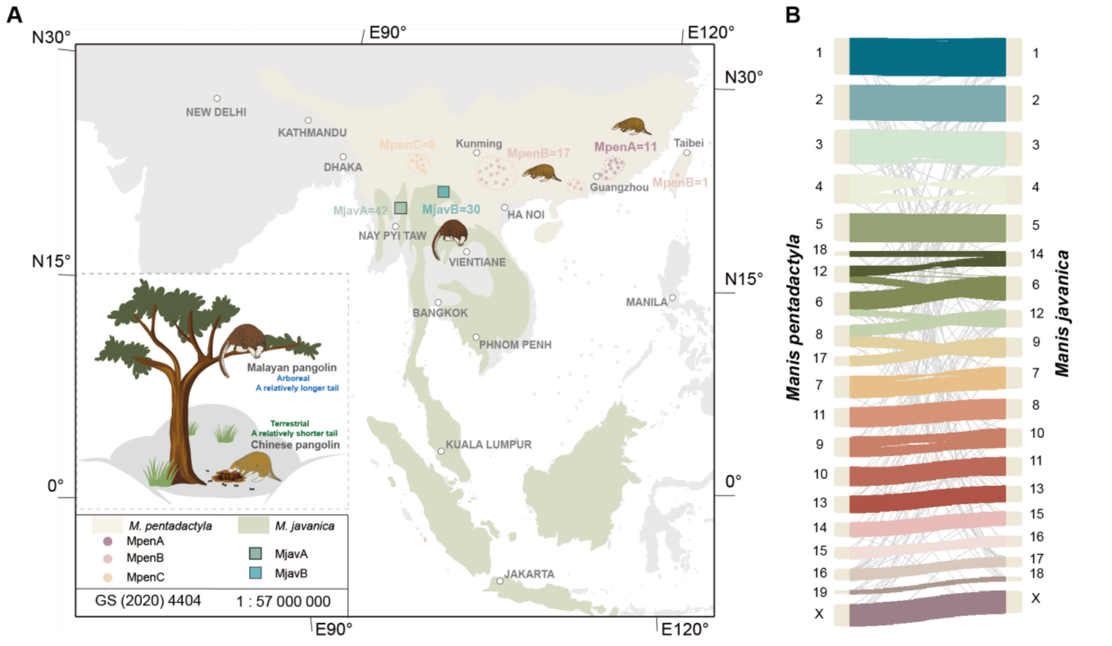

Response figure 2. Introduction to the species distribution and chromosome synteny of the Chinese and Malayan pangolins. (A) The distribution area and sampling sites of the Chinese and Malayan pangolins in this study. The circles represent sampling sites of the Chinese pangolins reported by Wang *et al*.[4] Samples without detailed locations are not shown on the map. (B) The chromosome-scale synteny analysis between the Malayan pangolin and Chinese pangolin genomes.

7. L145-150. The LG vs SG analysis is much clearer now, but with the ROH analysis I was still left feeling a little unsure of the deeper implications. Unless I have misunderstood, ROH is a relative measure of inbreeding and therefore the absolute changes are not of that much interest. Rather than ROH being bigger with LG vs SG (as expected), is the bigger finding not the apparent reversal in the relative recent inbreeding of MJ and MP as marked by ROH > 1Mb in LG (MP>MJ) compared to SG (MJ>MP)?

Response：Thank you for this comment. I am sorry that I could not well understand the "relative" you mentioned in the comment. I think the ROH is a direct measurement for the inbreeding, a longer ROH fragment can reflect a more recent inbreeding event, and the time of the inbreeding occurred could be also inferred with the known recombination rate and generation time for a species. The Froh could reflect the general inbreeding for a population, and the Froh for different length of ROHs could help track the inbreeding history of a species. The LG genome could contribute more for the accurately dating back of recent inbreeding history, which we think is the most important thing for the LG genome.

Yes, as you mentioned, one of the findings in this study is the reversal in the recent inbreeding between the MJ (Malayan Pangolin) and MP (Chinese Pangolin) population. This is indeed marked by the presence of ROH segments greater than 1Mb, where in the LG, MJ exhibits a higher frequency of such segments compared to MP (MJ>MP). Conversely, in the SG, MP has a more of these segments (MP>MJ). However, it is difficult to compare inbreeding between different species, because there are too many factors could influence the detection of ROH for different species, such as the different sequencing quality and depth, the different quality of reference genome, the different recombination rate, the different mutation rate, and so forth. Therefore, we did not discuss much about this difference. But we agree with you, this reversal is also an important observation that underscores the superior capability of high-quality reference genomes assembled from long-read sequences in uncovering the real level of inbreeding, when compared to those assembled from short-read sequences. Then, the use of long-read assemblies is crucial to avoid erroneous interpretations, such as the misconception that the Chinese pangolin is more inbred than the Malayan pangolin in the recent past, as might be suggested by findings based on short-read assemblies. By leveraging the detailed insights provided by long-read genomes, we can ensure that our conclusions regarding the inbreeding status of these species are both genuine and reliable. Following your comment, we have included additional discussion on this significant finding. In lines 310-313

8. I found the "Genetic Purging" section a little confusing and hard to follow. L253-4: "which indicated that the genetic purging in the MjavA and MjavB populations was less efficient, but stronger in the MjavA population" - less efficient but stronger than what? Can this section be simplified a little, perhaps with each result introduced in terms of what that particular result is testing and how it related to relative(/absolute?) genetic purging.

Response：Thank you for your comments regarding the "Genetic Purging" section of our manuscript. As your suggestion, we have rephrased the whole paragraph to make it clearer, and we deleted some confused and meaningless analysis from the revised manuscript to make this section simplified. Here we showed the revised content: *In the Malayan pangolin populations, the ROHf of highly deleterious mutations (LoF) was significantly lower than the nonROHf in both the MjavA and MjavB population*

(Fig. 5C). This denoted that many large-effect deleterious alleles (e.g. LoF) still existed but are masked in nonROH regions in both the two populations, indicating that the purging was less efficient to remove recessive deleterious mutations across the whole genome. Further inspection we found the difference between the ROHf and nonROHf was smaller in the MjavA population than that in the MjavB population (Fig. 5D and Supplementary Fig. S11a-S11c), indicating that the higher-level of inbreeding in the MjavA population may have facilitated the exposing of more recessive deleterious alleles to be homozygous, which has resulted in a more efficient purging of deleterious mutations in this population than in the MjavB population [5, 6].

9. Figure 5. Why are C&E and D&F visualised differently? I am not familiar with dot plots (that are not synteny plots) - the legend should explain what the different parts of the plot (lines and bubbles) mean. The boxes and whiskers should also be explained in the box plots. (i.e. what range do they represent.)

Response: Thank you for your attention to the details in our figures.

In our study, Figures 5C and 5E compared the frequency of LoF mutations (LoF/synonymous mutations) in ROH (ROHf) to that of non-ROH (nonROHf) regions within populations. Box plots (Figures 5D and 5F) was set to compare the ratio of ROHf to nonROHf of LoF mutations in the population to further clarify the differences between the LoF frequency in ROH and that in nonROH regions.

To further enhance the clarity and comprehension of this figures, we have expanded upon the legend for Figure 5 in the revised manuscript. This includes a detailed elucidation of the elements within both the dot plots and box plots. In lines 280-285.

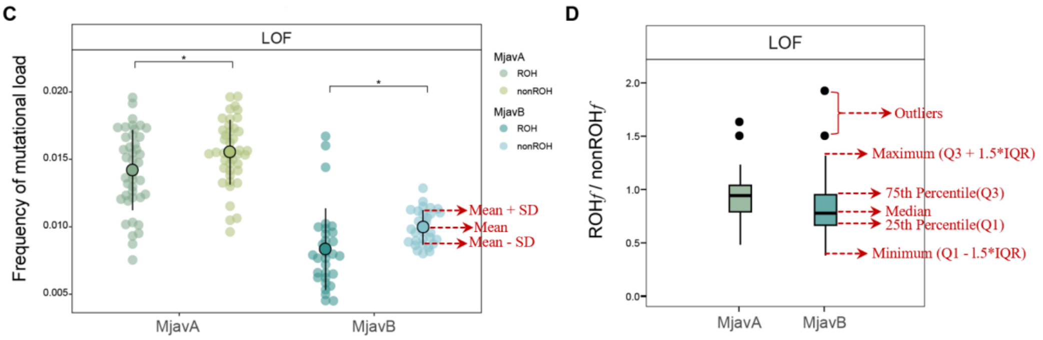

Response figure 3. (C) Dot plot showing the occurrence of LoF mutations in the two Malayan pangolin populations calculated as the ratio of the number of the mutational load to synonymous mutations in the ROH regions (ROHf) or nonROH (nonROHf) regions across the genome. Each point signifies the LoF frequency of an individual. The large dots represent the average LoF frequency for the population, while the lines indicate the standard deviation range around the mean. (D) The ratio of ROHf to nonROHf for the LoF in the two Malayan pangolin populations. The box represents the interquartile range (IQR), stretching from the first quartile (Q1) to the third quartile (Q3). The line that bisects the box indicates the median value. The whiskers extended from the box to show the variability of the data, typically reaching to the minimum and maximum values that fall within 1.5 times of the IQR from Q1 and Q3, respectively.

10. L385: Typo: "readsand"

Response：Thank you for the correction, it has been amended. In line 397

11. L469-470. Why was there the requirement for ancestral alleles to be the "predominant" allele? (This should be "Major" allele, I think.) I can see no reason why an ancestral allele could not be the minor allele.

Response：Yes, you are right here we mean the major allele, and we changed in the manuscript to avoid further misunderstanding. In line 482

Ancestral alleles are those that persist in their initial state, while derived alleles are the result of mutations that have diverged from this initial state. Under the neutral theory, it is common to consider the major allele of population as the ancestral one, especially conserved genomic regions across species[7, 8]. This is because the major allele, is more likely to be the one that has been preserved through evolutionary history, unless there is strong evidence of positive selection favoring a derived allele[9]. Another common approach to identifying ancestral alleles is by looking at the alleles present in closely related species[10]. This method is based on the assumption that mutations occur only once at a given position and that they happened after the species divergence[8]. Alleles that are shared among multiple species are more likely to be ancestral, as they have not diverged from the initial state. This shared state often corresponds to the major allele in the population [11].

While it is known that major alleles are not always ancestral, using major or common alleles as a proxy for ancestral alleles is a practical approach in the absence of a comprehensive list of ancestral alleles. Furthermore, we have integrated additional evidence from closely related species to further validate and refine our identification of ancestral alleles, ensuring greater accuracy in our determination. This inference method is widely used in genomics studies, including research on pangolins[8, 12-14].

12. L524. Why was k=17 selected? This seems very small for a vertebrate. There are only 17.2 billion unique DNA kmers, so the chance of random convergence in a >2Gbp genome is quite high. k=31 is the de facto standard, although k=21 or k=27 are also used quite often.

Response：Thank you for your comment on the choice of k-mer size in our analysis. We have updated our analysis by using k-mer size of 31 and included the results in the Fig S2.

13. L569: Typo: "derive"

Response：Thank you for your reminder. We have corrected it. In lines 578 and 580.

1. Cheng H, Concepcion GT, Feng X, Zhang H and Li H. Haplotype-resolved de novo assembly using phased assembly graphs with hifiasm. Nature methods. 2021;18 2:170-5.
2. Qi W, Lim Y-W, Patrignani A, Schläpfer P, Bratus-Neuenschwander A, Grüter S, et al. The haplotype-resolved chromosome pairs of a heterozygous diploid African cassava cultivar reveal novel pan-genome and allele-specific transcriptome features. GigaScience. 2022;11:giac028.
3. Rhie A, McCarthy SA, Fedrigo O, Damas J, Formenti G, Koren S, et al. Towards complete and error-free genome assemblies of all vertebrate species. Nature. 2021;592 7856:737-46.

4. Wang Q, Lan T, Li H, Sahu SK, Shi M, Zhu Y, et al. Whole-genome resequencing of Chinese pangolins reveals a population structure and provides insights into their conservation. Commun Biol. 2022;5 1:821. doi:10.1038/s42003-022-03757-3.
5. Nicolas D, Tom vdV, Hernán E. M, Christopher W. W, David D-d-M, Johanna vS, et al. Population genomics of the critically endangered kākāpō. Cell Genomics. 2021;1 1:100002. doi:10.1016/j.xgen.2021.100002.
6. Xue Y, Prado-Martinez J, Sudmant PH, Narasimhan V, Ayub Q, Szpak M, et al. Mountain gorilla genomes reveal the impact of long-term population decline and inbreeding. Science. 2015;348 6231:242-5. doi:10.1126/science.aaa3952.
7. Watterson G and Guess HA. Is the most frequent allele the oldest? Theoretical population biology. 1977;11 2:141-60.
8. Sabeti PC, Schaffner SF, Fry B, Lohmueller J, Varilly P, Shamovsky O, et al. Positive natural selection in the human lineage. science. 2006;312 5780:1614-20.
9. Fay JC and Wu C-I. Hitchhiking under positive Darwinian selection. Genetics. 2000;155 3:1405-13.
10. Rocha D, Billerey C, Samson F, Boichard D and Boussaha M. Identification of the putative ancestral allele of bovine single-nucleotide polymorphisms. Journal of Animal Breeding and Genetics. 2014;131 6:483-6.
11. Hacia JG, Fan J-B, Ryder O, Jin L, Edgemon K, Ghandour G, et al. Determination of ancestral alleles for human single-nucleotide polymorphisms using high-density oligonucleotide arrays. Nature genetics. 1999;22 2:164-7.
12. Naji MM, Utsunomiya YT, Sölkner J, Rosen BD and Mészáros G. Investigation of ancestral alleles in the Bovinae subfamily. BMC genomics. 2021;22:1-12.
13. Khan A, Patel K, Shukla H, Viswanathan A, van der Valk T, Borthakur U, et al. Genomic evidence for inbreeding depression and purging of deleterious genetic variation in Indian tigers. Proceedings of the National Academy of Sciences. 2021;118 49:e2023018118.
14. Hu J-Y, Hao Z-Q, Frantz L, Wu S-F, Chen W, Jiang Y-F, et al. Genomic consequences of population decline in critically endangered pangolins and their demographic histories. National Science Review. 2020;7 4:798-814.
