# Supplementary material for: Enhancing inbreeding estimation and global conservation insights through chromosome-level assemblies of the Chinese and Malayan pangolin
Source: Gigascience. 2025 Feb 14;14:giaf003. doi: 10.1093/gigascience/giaf003 (PMC11825179; doi:10.1093/gigascience/giaf003)
Supplement: giaf003_Response_to_Reviewer_Comments_Original_Submission [file giaf003_response_to_reviewer_comments_original_submission.pdf]

Author's Response To Reviewer Comments

Dear Editor and Reviewers,
Thank you for the many insightful comments and suggestions. We have made revisions to address all the comments. We have carefully considered each of your comments and have made the necessary revisions to address them thoroughly. Below, you will find a detailed list of our responses to the specific comments you raised:
Reviewer #1: The manuscript describes two novel VGP style genome assemblies of Malayan and Chinese pangolins. New assemblies provide a significant improvement to previously published short read references. Sequenced data and software used for assembly are reasonable. In addition to the genome assembly, the authors performed some population analysis using previously published resequencing data. I see no issues with the results of this part of the manuscript, but the discussion (comparison with previously published results) should be enhanced (as a part of review process). Title of the manuscript implies that such a comprehensive comparison is inside the text.
Major comments.
Q1.As I understood, no parental data was generated for genome assembly, and only HiFi + HiC data were generated from the reference individuals. It means that resulting assemblies are not completely haplotype resolved. Combination of HiFi and HiC allows to (nearly) completely resolve haplotypes only at the level of chromosomes, but chromosomal scaffolds are still mixed between haplotypes. It is a very common mistake to treat such an assembly as haplotype- resolved. I encourage authors to somehow underline it in the text, for example by adding "at chromosomal level" where it is necessary
Response: Thank you for pointing out this. Yes, our study did not generate parental data for genome assembly, we only utilized HiFi and HiC data from the same individual for genome assembly. We agree that the combination of HiFi and HiC data allows for nearly complete resolution of haplotypes at the chromosomal level, the chromosomal scaffolds are still mixed between haplotypes[1]. We appreciate your suggestion and will emphasize this point in the revised manuscript by including a clarification in the text, such as specifying “at the chromosomal level” where appropriate, to avoid any misinterpretation. Line 100.
Q2. QV scores look relatively low (<50). I would expect something like 55-60+ for such input data.
Response: Thank you for this comment. In the original version of this manuscript, we utilized short reads sequencing data to calculate the QV scores (MJ: 48.56; MJ: 49.63) by the Merqury software. In the revised manuscript, we polished the assemblies by HiFi long reads and WGS short reads, and used the high quality HiFi reads to calculate the QV scores by the Merqury software [3, 4]. The result showed that the QV score for the MP and MJ were 57.06 and 55.08, respectively, which means ~ 0.00019% and 0.00031% of base calling error rate, respectively, which could reflect a very high accuracy for our genome assembly. In addition, we have conducted an extensive search of published papers describing genomes with QV analysis by the Merqury software. We found that a number of published genomes showed QV values ranging from the 30 to 50 [5-7], which means the error rate of the base calling ranged from 0.1% to 0.001%. Therefore, we agree with you, a QV score of 55-60+ would be much better than < 50 in our previously assembled genomes.
Q3. Figure 1C is very difficult to understand. I encourage author to remove it completely or do a linear plot (or set of them) similar to Figure 1D
Response: Thank you to point out this. We have moved Figure 1C to the supplementary materials (Fig S17a) and added a linear plot here, as you suggested (Fig S17b-d).
Q4. row 162-163: "All these SVs were validated by our assembled contigs (Supplementary Fig. S9)". Figure is very unclear, and a reader may expect some results to be present on the figure and not the methods. Also I would expect some inversions encompassing whole contigs (especially at the end of chromosomes, where coverage often drops and contigs are shorter). Such inversions might be an artifacts of the HiC scaffolding and are very common. Please confirm that you have found none of them.
Response: Thanks for your suggestion. In response to the issue with the unclear figure, we have added a result description in the supplementary Fig. S18. This figure is intended to illustrate the validation of SVs by contigs mapping. For the potential issue of inversions, especially at the end of chromosome, we have meticulously re-examined our data and have made the necessary revisions to the structural variant (SV) results, we have taken great care to ensure that the SVs are accurately represented and inversions that are potentially be artifacts of HiC scaffolding have been all corrected in our analysis (Table S21).

Response figure 1. Validation of structural variants through contig mapping. Contigs are mapped to both the haploid genomes to verify structural variants and visualized by the IGV software. (a) The correct structural variants between the haploid genomes. The breakpoints of the SVs could be covered by complete contigs in both of the haploid genomes. (b) The incorrect structural variants between the haploid genomes. The breakpoints of the SVs in one of the two haploid genomes could not be covered by the complete contig. In the IGV screenshot, the gray bar represents the contig spanning over the SVs, and the contig corresponding SV regions were marked as the red box. All structural variants have been verified, and several are randomly displayed here.
Q5. row 311-316. A bit of a strange wording. "Much better contiguity, which is … longer". Please, simplify. For example by just comparing N50s in the main text
Response: Thank you. We agree that comparing the N50 values would provide a clearer and more straightforward comparison. We've revised the description here to make it more readable. Please refer to the supplementary material, lines 42-48, for the updated information.
Q6. Manuscript lacks a comparison (and even mention of) with chromosome-length assemblies of malayan and Chinese pangolins generated by DNAzoo team (upgrade of two draft assemblies from NCBI). They are available at dnazoo.org. Such a comparison makes more sense than comparing with old drafts.
Response: Thank you for your reminder. We download the genomes of Malayan pangolin and Chinese pangolin from the DNAzoo website. And we added the comparison with all assemblies and presented the result in Supplementary Table S19. By this comparison, we found that the scaffold N50 of the genomes we assembled in this study are remarkably higher than those of the genomes published by DNAzoo. This comparison further showed the superiority of our assemblies in contiguity, thank you.
Q7. Paragraphs "Genome-wide extinction risks in pangolin populations" and "Novel implications for the global conservation of these two pangolin species" confuse me a bit. Authors state that they have not generated the resequencing data, but downloaded it from public databases. Dataset set includes data from three publications (references [25, 33, 57]). Majority of it (nearly all Malayan pangolin samples and >60% of Chinese pangolin) were analyzed in [25]. All Chinese pangolin samples were analyzed in [57]. Authors cite both publications several times, but text lacks a more detailed comparison between the manuscript's results and previously published ones. It is especially crucial and interesting for Chinese pangolin as there is a 100% overlap in samples with [57]. The title of the manuscript requires an extensive comparison with previous publications in all the discussion.
Response: We appreciate your comment and we agree. One of the most important points in this this manuscript is to discuss how a high-quality genome could improve the evaluation of genetic parameters in conservation genomics or in population genomics, particularly in evaluation of genetic diversity and inbreeding by screening ROH across the genome, because a reference genome with low genome contiguity cannot well support the detection of long fragments of runs of homozygosity (ROH):
1) We found much higher genetic diversity than that reported by[8, 9], indicating that we may have detected more variants across the genome based on high-quality reference genome assembled in this study, because HiFi long reads could span much more complex genomic regions[10] and generate longer contigs [11, 12], and many genomic regions that cannot be assembled by short reads can be assembled by long reads, and these regions may contain important variants.
2) The inbreeding level (ROH detection) detected based on the reference genome we assembled in this study was significantly higher than that identified using the short reads assembled genome, particularly for ROHs larger than 1Mb, which is largely due to that the short contigs in the reference genome cannot span over long ROH fragments. By comparing with previously published short-read assembled genomes, findings in this study could tell the reader the superiority of a reference genome with better contiguity on the detection of genetic diversity and ROH fragments.
In addition, we have incorporated comparisons of our results with the results published by other studies, which can be found in many places throughout the manuscript, particularly in the results section:
1)The trajectory of the change of effective population size of Malayan pangolins presented in this study is different from that reported by Hu et al. We inferred that the MjavA (MJA in Hu et al.) population experienced a continuous population decline and the population size was lower than that of the MjavB (MJB in Hu et al.) population within the most recent 10,000 years, which could be well matched with the lower genetic diversity and higher genome-wide inbreeding in the MjavA population. But in the previous report, Hu et al,. found that the population size of the MjavA was much greater than the MjavB population from 10 kya to 3.2 kya. This difference is largely due to the different methods we used, and we did not used the method in Hu et al. because we have compared these two methods in our previous study[13] and found that this method is not as stable as the method we used in our study.

2)The parameters used in the filtering of genome-wide SNPs in Hu et al,. is too strict, SNPs with a minor allele frequency less than 0.2 will be all removed from the data set, which will overestimate the inbreeding across the population by erroneously introducing long ROH fragments (>1Mb). And in our study, we found much less long ROH fragments (>1Mb) than that in the Hu et al,. even if we used HiFi genome for reference. The parameters used for identifying the ancestral state in Wang et al,. is relatively rough by considering alleles in the Malayan pangolin genome as the ancestry state. In this study, we used the more commonly used method to identify the ancestral allele, which is more reliable. Therefore, the accumulation of derived mutational load for the Chinese pangolin populations in our study is different from that in Wang et al. We added this comparison in the maintext.
3)The genetic purging is a very important issue in the conservation genomics, genetic purging always exists in a population under purifying selection[14]. In general, deleterious alleles affecting fitness and viability are initially rare and may be hidden in the heterozygous state, but are more easily to be cleared from the population when these deleterious alleles are exposed in a homozygous state (Purging). This is an indicator for the evaluation of the extinction risk of endangered species (Response figure 2). However, in previous study (Hu et al, Wang et al and Wei et al), they did not mention this process in pangolins. In this study we discussed genetic purging in both Chinese and Malayan pangolins.

Response figure 2. The relationship between fitness and inbreeding[15].
Minor comments:
Q8. row 36-44: first paragraph is redundant and can be safely removed. It describes very basic things and facts.
Response: Thank you for your comment. We removed this paragraph in the revised manuscript. Lines 41-49.
Q9. row 60: Pangolin is a prehistoric mammal = "prehistoric" doesn't look like a good term here. Maybe 'living fossil' is better
Response: Thank you and we revised. Line 67.
Q10. row 81: remove 'recently introduced'
Response: Removed, thank you. Line 92.
Q11. row 82: "HiFi-specific assembler can generate high-quality and haplotype-resolved de novo assemblies" HiFi data itself (without HiC or parental data) can't produce haplotype-resolved assembly. Please, Correct
Response: Thank you for your careful reminder. We have modified the description to make this part more rigorous. Line 93.
Reviewer #2: This paper presents two new chromosome-level assemblies for two species of Pangolin, alongside some population genetic data from different populations of each. The quality of the genomes appears to be very high, and these data will indeed "provide valuable genomic resource for future studies on the protection and conservation for pangolins". However, this is Research Article and not a Data Note, and so it also needs a clear narrative relating to the biological insights and which aspects of the data are of most interest. This is currently hampered by numerous language errors, inconsistent formatting, and excessive reference to relatively uninteresting supplementary data. Having some clearer aims/questions beyond a better genome would be helpful in this regard.
If there is no clear message, the authors could consider a data note instead. Either way, I think a lot of the results should be consolidated and presented as Supplementary Results to emphasise the important/interesting data. There is also excessive comparison of results using their long-read and an unknown short-read genome, which should be reduced. (See detailed comments, below.) The manuscript would greatly benefit from professional editing or detailed proof-reading by a native English speaker.
Response: Thank you for your insightful comments for this manuscript. We are glad that the reviewer noticed the high quality of the chromosome-level assemblies for the two pangolin species and the potential value of our data as a resource for future conservation efforts.
We understand your concern about the need for a clearer narrative that highlights the biological insights and the most significant aspects of our data. In response to your suggestion, we have further revised the discussion section to better articulate the key research questions and the significance of our results. We removed the first part of the discussion and the description of the genome assembly in the result part to the supplementary data to help emphasize the most important findings.
Additionally, we have invited a native speaker to polish the revised manuscript and we believe that the language has been much improved.
MAJOR COMMENTS

Q12. For a Research Article, the main narrative is very unclear and the reader has to do far too much work to extract the relevant insights. The abstract hints at a "greater(?) improvement" and "different results in detecting genome-wide extinction risks being compared with short read assembled genome", though also results "were verified again" so are not actually different to previous findings? Except maybe one population (MJ2). This is all vague and confusing. Please clearly identify the key take-home messages from the new data, be explicit about it, and present a unified narrative from Abstract to Conclusions. Is the "relative mild genetic purging in pangolin populations" important? Is the purging itself the new finding, or the fact that it is relatively mild? I found the entire section (L268-294) to be rather confusing with a lack of clarity regarding population-specific versus general conclusions. The narrative would be aided greatly if the subheadings highlighted the key results/points rather than simply breaking the manuscript up into topics.
Response: Thank you for pointing out this issue, which we think could help improve this manuscript a lot. We try to answer your question and meanwhile re-organize the manuscript to make it more logic and clearer.
Yes, we obtained different results on the detection of genetic diversity, inbreeding, and accumulation of derived mutational load, based on the long-read assembled genome we showed in this study, when compared with studies reported by Hu et al, Wang et al and Wei et al. However, we did not directly compare with their studies to avoid over overemphasis this difference to showed superiority in this study? We just state our results, however, we almost didn't show duplicated results with previous study. In the revised manuscript, we added some direct comparison to make it clearer, which could be found at many places throughout the manuscript.
Actually, this manuscript mainly focused on how a high-quality genome could improve the study of conservation genomics, and this manuscript is the first time (as far as I know) to compare the short-read assembled genome and long-read assembled genome on the evaluation of genetic parameters of conservation genomics or population genomics, although the comparison may be relative limited. In this manuscript, we prepared whole paragraph to discuss this comparison (Lines 417-440).
Based on this comparison, we showed the superiority of the long-reads assembled genome to evaluate genetic diversity and inbreeding, further discussed genetic diversity and inbreeding we calculated based on the high-quality genome in this study, which could help us re-understand the genome-wide extinction risk of the two pangolin species. We also updated the result of ROH screening and accumulation of mutational load by using more appropriate parameters, please refer to Q7.
Furthermore, as you mentioned, we also explored the genetic purging in the five pangolin populations. The genetic purging always exists in a population under purifying selection[14], and is a very important issue in the conservation genomics. In general, deleterious alleles affecting fitness and viability are initially rare and may be hidden in the heterozygous state, but are more easily to be cleared from the population when these deleterious alleles are exposed in a homozygous state (Purging). This is an indicator for the evaluation of the extinction risk of endangered species. However, the genetic purging has been never discussed in (Hu et al, Wang et al and Wei et al). In this study we detected and discussed genetic purging in both Chinese and Malayan pangolin populations. Therefore, both the genetic purging and the "mild" are new findings in this study. We also revised the subheading to highlighted the key results/points in this study. Thank you again.
Q13. There are far too many supplementary figures and tables. I recommend trying to consolidate some (eg. S23-S28) into fewer multipanel figures that address the same question. Likewise, many of the tables could be combined and consolidated as the same data is presented for MJ and MP in two table, or tables with the same rows (samples) could be combined into a bigger table. Ideally, some of the descriptive results could be moved from the main manuscript body into a Supplementary Results section along with many of the references to these supplementary data. It would be helpful if the figures could be more carefully selected and organised/labelled to support the narrative.
Response: We appreciate your suggestions regarding the supplementary figures and tables. We have addressed these issues as follows: 1) We combined several groups of figures into multipanel figures, including you mentioned S23-S28. 2) We have reviewed the tables and combined those tables presenting similar data for MJ and MP into single tables where appropriate. Additionally, we merged tables with overlapping rows (samples) to create bigger tables. 3) We moved some descriptive results from the main manuscript to a Supplementary Results section to enhance the readability of the main text. We also adjust references to supplementary data accordingly. 4) We carefully selected and organized the figures, ensuring that they are well-labeled and directly support the narrative of the manuscript. We believe that the narrative of the revised manuscript has been much improved.
Q14. There is insufficient information and discussion about the previous pangolin genomes. Most

important, genome "SG" does not seem to be defined anywhere. Is this a previously published genome? Or a short-read assembly of new data? There is a bit of discussion about the previous genomes (L306-323) but the comparisons in Tables S31 and S32 are unclear - are the new genomes missing from these tables? (What are they called?) I think this should be the first Results section. As these are phased chromosome-level vertebrate genomes, assembly statistics should be presented in the context of VGP/EBP standards. There should also be data presented for both haplotypes in Table 1.
Response: Thank you for your concern. “SG” stands for “short-read assembled genome”, referring to a genome that was assembled based on the short-read sequencing technology. In this study, the SG genomes specifically referred to the genome of the Malayan pangolin of the YNU_ManPten_2.0 and of the Chinese pangolin of the YNU_ManJav_2.0 (collected from the NCBI). These two genomes have been widely used in previous studies, and we want to use this genome as a representative for the short-read assembled genome with low contiguity for comparison with the long-read assembled genome we prepared in this study. We defined it in Figure legend and Abbreviations section, we have added further explanations in the revised manuscript. Lines 242 and 957
To facilitate the reading and understanding of the differences between these published pangolin genomes and genomes assembled in this study, we added required information of our newly assembled genomes in Supplementary Tables 19. We have named our assemblies following the Vertebrate Genome Project (VGP) conventions and ToLIDs. Specifically, the Malayan pangolin genome assembly was named as mManJav1.1, and the Chinese pangolin genome assembly was named as mManPen9.1, and we have registered in the ToLIDs. In the Results section, we have provided detailed description of characteristics of our new assemblies in Table S3 and S4, and included a detailed description of assembly statistics in accordance with VGP/EBP standards (Table 1).
Q15. The SG vs LG comparison is largely a distraction and floods the paper with boring SI. It is no surprise that LG is better for SVs and long ROH. This is a research paper about pangolins, not a methods paper about LR vs SR genomes. Please reduce this to a clearer question: have there been any previous conclusions based on the other genomes that have been contradicted or (in)validated by the new genomes? Please give your new assemblies names that distinguish them from both each other and the previous assemblies - I recommend getting ToLIDs for your samples and naming the assemblies according to VGP conventions.
Response: Thank you for pointing out this. As we mentioned in the Q12, how a high-quality genome could improve the study of conservation genomics is a very important part in this manuscript. I agree with you that it is no surprise that LG is better for SVs and long ROH. As you mentioned, we can predict that a LG genome would be better than SG genome for the detection of SVs and long ROH fragments. However, many researchers may don’t know under what situation a LG genome will present a better performance than a SG genome, and to what extent a SG genome worse than a LG genome. As you could see in many conservation genomics papers, the inbreeding, genetic diversity and the accumulation of mutational load are all very very important genetic factors that could help wildlife managers (maybe the government) to make conservation decisions. An accurate evaluation of these genetic factors is a big thing in this research field. But unfortunately, I did not see a comprehensive evaluation and comparison between the SG and LG on the impact of evaluating of parameters in conservation genetics. In addition, as you know, for many people who used genome as a tool to guild conservation practice are actually not experts on genomics or freshman in this field, they need a guild to do their works? As far as I know, this is the first time we comprehensively compared the impact of the SG and LG on the evaluation of conservation genomics related parameters. Although it is not surprising that LG is better for SVs and long ROH, but we provided many detailed things other than a simple conclusion that the LG is better than the SG, which we think is very useful for wildlife conservation researchers and workers? Yes, we still agree with your points and we have tried to shorten this comparison in this revised manuscript.
Actually, in this manuscript, we almost only showed the results that were different or contradict (at least improved) with that reported in Hu et al and Wang et al:
1) We obtained different results on the detection of genetic diversity, inbreeding, and accumulation of derived mutational load, based on the long-read assembled genome we showed in this study, when compared with studies reported by Hu et al and Wang et al. Based on this comparison, we showed the superiority of the long-reads assembled genome to evaluate genetic diversity and inbreeding, further discussed genetic diversity and inbreeding based on the high-quality genome assembled in this study. Another point is that, we showed the very detail map of ROH distribution across each chromosome in the genome, which is not showed in Hu et al, Wang et al and Wei et al, and this information is very helpful for guiding the future genetic rescue of pangolin populations. For example: the breeding of Chinese pangolin in

China is very successful and there are more than 100 captive individuals by far in the breeding center, but if we consider the reintroduction program, which individual should be selected to the wild? We could then select more appropriate candidate by referring this ROH map? But not to refer a very rough or a general inbreeding coefficient.
However, we did not directly compare result in this manuscript with previous studies (Hu et al and Wang et al) to avoid over overemphasis this difference to showed superiority in this study?, because the science and technology are moving forward. Although we just state our results, we almost didn't show duplicated results which has been reported in previous studies. But you are right, in the revised manuscript, we added some direct comparison to make it clearer.
2) Furthermore, we also explored the genetic purging in the five pangolin populations, which are never discussed in Hu et al, Wang et al and Wei et al. In general, deleterious alleles affecting fitness and viability are initially rare and may be hidden in the heterozygous state, but are more easily to be cleared from the population when these deleterious alleles are exposed in a homozygous state, we could say that this process is genetic purging. The genetic purging always exists in a population under purifying selection[14], and is a very important issue in the conservation genomics.
3) In addition, we have updated the results reported in previous study as we mentioned in Q7 and Q12.
To ensure clear distinction and proper identification of our new assemblies in the context of existing genomic data, we assigned unique names to our assemblies by following your advice. We obtained ToLIDs (Taxonomic IDs of Life) for our samples (MJ: mManJav1.1; MP:mManPen9.1), which will facilitate their recognition and propagate across the scientific community. In line with the VGP (Vertebrate Genomes Project) conventions, we renamed our assemblies accordingly, and incorporated these new names into the revised manuscript and ensure they are used consistently throughout the text, tables, and figures.
Q16. Do the new genetic data support the previous population groupings? (Was this based on genetic data itself?) To my eye, the genetic data presented in Fig S15 and S16 do not entirely support the existing population definitions. This needs more discussion. Is only a subset of MJ1 truly different to MJ2? Is there a fourth subpopulation of MP including the Taiwan sample?
Response: Yes, the genetic data supported the previous population grouping and this is almost based on genetic data itself, because the sampling locations for these samples are not all very clear.
In this manuscript, we mainly focused on the comparison of the grouping results (PCA, phylogenetic tree and admixture) generated based on the SG and LG, and to explore the potential impact of the reference genome to the genetic structure analysis. We found that the two groups of results are almost the same, and were very consistent with that reported in Hu et al and Wang et al (Supplementary Fig. S4-S7). Therefore, we did not present very detailed discussion about the genetic structure in this study to avoid repeated discussion. However, we still added a short description about the Taiwan individual in Lines 224-225.
Q17. Is it possible to clarify the "variable" karyotype from ref 47? Is this biological or technical variation in results? Is both species or just Mpen? Do your results clarify the karyotype in this population - the variation is geographical, right?
Response: As description in reference 47, the Chinese pangolin has been documented to exhibit four different groups of karyotypes, with the chromosome numbers varying from 2n = 36 to 2n = 42 across published research[16-18]. Yes, we cannot confirm from the reference 47 whether the Chinese pangolin's karyotype is variable, because 1) the 2n = 36 karyotype found in India may actually belong to M. crassicaudata[19], 2) the 2n = 42 from Taiwan could be due to a counting error[18]. It appears that the variability of the Chinese pangolin's karyotype cannot be confirmed based on reference 47. Therefore, We have therefore modified this description.
It is difficult to definitively determine whether the variable karyotype of the Chinese pangolin is resulted from biological variation or technical variation. This could be attributed to the different methods used for counting chromosomes, the techniques for preparing and staining the chromosomes, or the criteria used for identifying and counting, and so forth. Some of the variation might also stem from misidentification of species. As mentioned, the 2n = 36 karyotype found in India might actually belong to M. crassicaudata rather than M. pentadactyla.
The discussion in the ref 47 is specific to M. pentadactyla, and does not mention the M. javanica or other species in the context of the variations in diploid numbers. Also, we do not have karyotype data or HiC data from individuals from different populations, so we cannot confirm whether the karyotype variation (if have) existed in different populations.
Can you also confirm that the apparent swapping of chromosome arms on Chr4 between the species is not just a scaffolding error. Are these karyotypes consistent in both haplotypes?

Response: Thank you for your reminder. We used contigs to verify whether the swapping of chromosome arms on Chr4 was a scaffolding error (Response figure 3). The verification is as follows:

Response figure 3. Contig alignment across the region confirms the accurate rearrangement of Chromosome 4 swapping.

As shown in the figure above, by aligning contigs to Chromosome 4, we can see that both the breakpoints in the Chinese pangolin genome and the Malayan pangolin genome could be covered by a complete contig, which confirmed the accuracy of the assembly at this region, verifying the presence of the swapping showed in the main figure.

Yes, the karyotypes is consistent in both haplotypes. In Fig S1, we have showed the Hi-C heatmaps for each haplotype to demonstrate that both haplotypes possess the same karyotype numbers. We also compared the haploid and diploid genomes separately, and the collinearity of all chromosomes is consistent (Response figure 4), further confirming the karyotypic concordance between the haploid and diploid genomes.

Response figure 4. (a) Synteny analysis reveals genomic consistency between haploid (MJH1) and diploid (MJG) Malayan pangolin genomes. (b) Synteny analysis reveals genomic consistency between haploid (MJH2) and diploid (MJG) Malayan pangolin genomes. (c) Synteny analysis reveals genomic consistency between haploid (MPH1) and diploid (MPG) Chinese pangolin genomes. (d) Synteny analysis reveals genomic consistency between haploid (MPH2) and diploid (MPG) Chinese pangolin genomes.

It would be good to see the contact maps (Fig S1) and synteny plots (Fig 1D) of the haplotypes against each other and have the telomeres marked to verify the orientation of arms where possible.

Response: The synteny plots between haplotypes were presented in Fig S17 (c) and (d). Following your suggestion, we have made every effort to identify the telomere status of our assembled genomes; telomeres in some chromosomes could be assembled, which are marked in Response table 1. Due to the limitations of sequencing technology, this study does not represent a complete telomere-to-telomere (T2T) pangolin genomes, therefore, telomeres sequences in some chromosomes could not be identified.

Response table 1. The distribution of telomeres in the genomes. "no" means no telomere sequence is identified, "left" means telomere sequence in the left end of the chromosome is identified, "right" represents telomere sequence in the right end of the chromosome is identified, "both" means telomere sequence in both ends of the chromosome is identified.

Is Fig S1 both haplotypes? If so, please mark the actual chromosome boundaries and orient the pairs in the same direction. Is the X pattern a pair of chromosomes inverted relative to each other, or is it interactions between chromosome arms of one haplotype?

Response: Yes, Fig S1 displayed the Hi-C heatmap of chromatin fibers from both haploid genomes. The X pattern you mentioned represents the interactions between chromosome arms of the two haplotypes, rather than a pair of chromosomes inverted relative to each other. Such interactions are common as it reveals the organization and physical contacts of chromatin in three-dimensional space. The actual chromosome boundaries you mentioned have been marked in the figure (Fig S1). The X pattern in the Hi-C heatmap is visualized solely to represent the interaction between the two sets of chromatin fibers. To avoid ambiguity and better display the karyotypes of the two haplotypes, we adjusted the sequences of the two haplotype chromosomes to make them initiate in the same direction, which could be seen in the revised Fig S1.

The parallel MP Chr5 pattern makes me think the former, but I initially assumed it was one haplotype.

Response: For the Chr5, the Hi-C interaction map reflected the interactions between the two haplotypes. In our revised version, the orientation of all pairs of haploid chromosomes have been adjusted to a better illustration of the interaction between the two haplotypes (Fig S1).

Where are the X and Y on the dot plots (Fig S7/S8)? Have both been moved into H1? It would also be clearer if Fig S3 used chromosome labels not scaffold numbers. How were the X and Y identified?

Response: In the original versions of Fig S7 and S8, the sex chromosomes were not depicted, and it was not the case that the sex chromosomes were all moved to H1 assembly. In the revised revision, we have incorporated the visualization of synteny between the sex chromosomes, which can be seen in Fig S19 (c) and (d). In this study, the assembled regions related to the Y chromosome of the Chinese pangolin and the Malayan pangolin are approximately 6Mb in length, and a complete Y chromosome assembly was not achieved, largely due to the limitations of sequencing and assembly technologies, as well as the highly repetitive and complex structure of the Y chromosome. There are relatively few homologous regions

between the X and Y chromosomes, and the collinearity regions that can be visualized are also relatively limited. Therefore, the sex chromosomes were not depicted in the original FigS7 and S8. In the revised version, we have included the collinearity between the sex chromosomes in FigS19 (c) and (d).
For clarity, we updated Fig S3 by using chromosome labels instead of scaffold numbers in the revised manuscript.
We confirmed the X chromosome and Y-linked regions by two methods: 1) The presence of the SRY gene was used to identify Y-linked regions; 2) Based on the truth that the sequencing depth and coverage of the sex chromosomes is half of that of the autosomes for a male pangolin individual, as shown in Figure S3.
For clarity, we have included a description of the identification of sex chromosomes in the methods section.
I think Fig 1C tries to present some of these data too but it is really unclear, being both too small and lacking any scales/legends.
Response: Thank you for your suggestion. We have revised Figure 1C by adjusting the panel size and adding legends and scale to clearly present the genomic information of the two pangolin genomes.
Additionally, we have moved this figure to the appendix as Fig S19(a).
Given the questions over karyotype and the risk of scaffolding errors, I would like to see a deeper analysis of the proposed sites of fusion/fission.
Response: Thank you for your comments. To minimize the likelihood of false positive fusions and fissions that may arise from errors during scaffolding, we have conducted validation for all fission and fusions by aligning contigs to the genome assembly. This additional step ensures that the proposed fusion and fission sites are supported by more evidence, thereby preventing potential inaccuracies in scaffolding. The verification results are detailed as below (Response figure 5):

Response figure 5. Validation of the fusion, fission and main breakpoints between Chinese pangolin and Malayan pangolin genomes by aligning contigs to the two reference genomes. (a) Verification of fusion and fission positions. The left panel represents the synteny relationship, the left panel shows the support of contigs against the breakpoints of the fission and fusion events, the gray bar represents the contig and the red dot represents the breakpoint. (b) Verification of the other four significant breakpoints. In the IGV screenshot, the red mark represents the relative position of the SV breakpoint in the chromosome, and the gray band represents the contig at this position.
B. MINOR COMMENTS
Q18. There are a lot of typos. Most of these just impact readability, but some make the message unclear.
Response: Thank you for pointing out this question. We have reviewed and corrected these typos.
Additionally, we invited a native speaker to polish the language throughout the manuscript to ensure clarity and improve readability.
Q19. I found the use of MJ, MP and CP to be a bit confusing at first. (MP could be Malayan Pangolin vs CP, but it isnt.) Can the MP/CP for the Chinese Pangolin be made consistent? Mjav and Mpen might be clearer.
Likewise, it would be good to be consistent with the ordering and colours used for the species and populations when presented in tables and figures.
Response: Thank you for this comment. Yes, we agree with your suggestion, the Mjav and Mpen are clearer, and we changed the population name in the revised manuscript as the Response Table 1. We have also reviewed our manuscript and ensure that the ordering and colors were consistent throughout all tables and figures.

Q20. Page 4: "the most promising strategies for genome assembling" - This is combining long-read and HiC data. Whether ONT or PacBio is better is subject to debate.
Response: Yes, we agree. It is debated that whether ONT or PacBio is the better technology for long read sequencing, and both of the two sequencing technologies are among the best strategies for genome assembly at present. Therefore, we used 'one of' in the manuscript. Thank you! Line 94.
Q21. L214: " Among the three Chinese pangolin populations, the inbreeding of the CPA and CPB populations were comparable (CPA: 0.18±0.036; CPB: 0.17±0.039), but much worse than the CPC population (FROH=0.06±0.005) (Fig. 3A and 3E, Supplementary Table S26)." Does this relate to population size and genetic diversity? The heterozygosity of the reference genomes looks low from the Merqury kmer plots. Are they representation of the population heterozygosity?
Response: Thank you for this question. The ROH fragments could well reflect the inbreeding level in a population. In general, small populations are more tend to occur the inbreeding, and an inbred population often presents low genetic diversity (π) and small population size. If a population is large enough and under the normal mating, it is almost impossible to present severe inbreeding. However, we have to keep

in mind that, the inbreeding will not directly cause the low genetic diversity, but could make the genome homolyzed, and the homolyzed genome will further expose the recessive deleterious mutations, and some of these exposed deleterious mutations will cause severe consequences, such as death before mature, reproductivity problem or malformation….. Therefore, the loss of effective individuals in a population will take the genetic diversity away, then we can often see a low genetic diversity in an inbreeding population. But this is not always true, it is a very complicated thing if we discuss it as a specific issue.
The low heterozygosity showed in the Merqury kmer plots of the reference genome could reflect the genetic diversity of a single individual (usually can represent this species), but couldn't represent the population level genetic diversity. The nucleotide diversity (π) is a genetic parameter that could well reflect the population level genetic diversity.
Q22. L218: "Noteworthy, the FROH of the Taiwan individual is much higher than all other individuals in the MP population (FROH=0.54) (Fig. 3C)". There is no MP population. There are three. Confusing. Why is this noteworthy?
Response: Thank you. The MP population we mentioned in this manuscript represents the whole Chinese pangolin populations, including the CPA (MpenA), CPB (MpenB) and CPC (MpenC) populations. We mentioned the "MP" population in several places in the manuscript. To avoid the misunderstanding and make the manuscript more readable, we changed the "MP population" to the "the three Chinese pangolin populations" throughout the manuscript. Here we say "Noteworthy" is not because of the high inbreeding level in this individual, but because almost all the ROH fragments in this individual restricted to less than 1Mb (benefiting from the detailed distribution map of ROH fragments we detected in this study), which is not a normal thing for endangered species, but is underestimated in the study of Hu et al (please refer Q7).
Q23. Fig 3. Please make the colours and ordering match. Fig 3C is not clear. The size mapping is superfluous as it is just the y-axis. Would be better to use symbols/fill to more clearly differentiate the populations/species. It is not clear from this plot why Taiwan is singled out.
Response: Thank you for this reminder. We have revised Fig 3C to make the colors and ordering match each other. We removed the size mapping, and we now used the different colors to represent different populations. Notably, the FROH for the Taiwan individual is significantly higher compared to all other individuals in the Chinese pangolin population, then we showed the Taiwan individual to help reader to trace this individual.
Q24. L390. "The Chinese pangolin and Malayan pangolin used for genome assembly were wild rescued individuals by the Guangdong Wildlife Rescue Center. " As these are proposed reference genomes, is it possible to proved any photos or metadata to validate the species IDs? What populations are they from?
Response: Thank you to point out this issue. We have discussed with people in Guangdong Wildlife Rescue Center for this issue. Unfortunately, the geographical origin of these two individuals used in this study is not very clear, as both individuals were confiscated from criminals, and these individuals have been passed around many times, making it difficult to track the detailed geographical location for these two individuals.
Q25. L410: "Then, the Hi-C sequencing reads are mapped to the primary genomes by the mem algorithm of Burrows-Wheeler Aligner (BWA, v0.7.17) [66, 67], while Hi-C data quality control was conducted by the Juicer[68] (v1.5). The 3d-DNA pipeline (v190716) was finally used to concatenate and review the scaffolds to chromosome-scale genomes[69]. " How were phased assemblies produced if only the primary genome was scaffolded? Why were both haplotypes not scaffolded independently? I am confused how you can comment on haplotype synteny if not. Was manual editing of the scaffolding performed as part of the assembly curation?
Response: Thank you for this comment. The Hi-C sequencing data was used for two times in the genome assembly: 1) Phasing: we could feed the hifiasm software with both the HiFi sequencing data and Hi-C sequencing data, and then the hifiasm software will output three genomes at the same time: the hybrid genome, and the two haploid genomes (for a diploid species). The hifiasm software could automatically phase the hybrid genome into two haploid genomes, even we have no sequencing data from the trio samples. 2) Concatenating: the Hi-C data will be again used for contaminated the scaffolds generated in the previous step to the chromosome-scale genome. We have revised the description for the assembly of the genome to make it clearer (Lines 531-534). Yes, we will manually adjust the genome according to the Hi-C interacting map.
Q26. L460. What does "SNPs that were missed" mean? No coverage? Filtered?
Response: Thank you. In the VCF (Variant Call Format) file, a genotype at a specific genome position of all individuals will be listed in a row in the VCF file (Response figure 6), and if no sequencing reads could well support the base calling in this specific genome position in an individual (due to low sequencing coverage,

low quality sequencing data, or failed base calling in complex genome regions……), we identified this as a missing genotype at this specific genome position for this individual. If the missing rate exceed 20% at a specific genome position, this row will be deleted from the VCF file. For example, there are 100 individuals in a VCF file, and the genotype at a genome position should be successfully called in more than 80 individuals, or this site will be filtered from the VCF file.

Response figure 6. A normal VCF file.

Reviewer #3: This manuscript by Lan et al. presents high-quality, haplotype-resolved, and chromosome-scale reference genomes for Chinese and Malayan pangolins, providing crucial insights into genetic diversity, inbreeding, and extinction risks. The study highlights moderate inbreeding in most populations, identifies a highly inbred Malayan pangolin population needing targeted conservation, and detects mild genetic purging. These findings contribute valuable genomic resources and new perspectives for pangolin conservation efforts. In general, the manuscript has clear logic, detailed description and comprehensive data analysis. Therefore, I recommend a minor revision before acceptance. There are some minor comments and suggestions as following：

Q27. 1. The authors used abbreviation of the Latin names for the Malayan pangolin (MJ) and Chinese pangolin (MP) in the third paragraph. However, they changed the way of abbreviation for the Chinese pangolin populations (CPA, CPB and CPC) later on. It's not a problem, but a little bit confusing for the readers to catch up the logic of describing.

Response: Thank you for pointing out this question. Yes, we agree. We used the CPA, CPB and CPC to keep consistence with the published papers, but as you mentioned, this will make the manuscript hard to be follow. Therefore, we changed the abbreviation name of the Chinese pangolin and Malayan pangolin to make them more readable (Response Table 1).

Q28. Lines 74-75, "The MJ1 population was …… diverged from the MJ1 population"???

Response: Thank you for pointing out this issue. We have corrected the description error in Lines 84.

Q29. 3. In Figure 1A, the legend is inconsistent with what have been shown in the figure. I suppose the authors used wrong colors to indicate CPA and CPC populations. Moreover, chromosome numbers, Hic_scaffold numbers in Figure 1C, Figure 1D, Figure S3 and Figure S5 are not in consistency. Please double check chr 7, chr 8, chr 20 and X chromosome in these figures.

Response: Thank you for your kind reminder. We have corrected the legend and the colors represent the population in Figure 1. In the original manuscript, Figure 1C shows the synteny between the two pangolin species, which was achieved by aligning and comparing the hybrid genomes of the two pangolin species. Figure 1D presents an analysis of the structural variation (SV) across the genomes of each pangolin species, specifically focusing on that between the two haplotype chromosomes. So, the numbering in these two figures is not consistent. We have carefully revised these figures to ensure that the chromosome and Hic_scaffold numbers are consistent across all mentioned figures. The revised figures can be found in Supplementary Figure 19a, Figure 1B, Supplementary Figure 3, and Supplementary Figure 15.

Q30. Usually, the mean depth of sequence data has an impact on the genomics analyses. In this study, the sequencing depths of the samples provided by the authors were basically between 10-20-fold, but depths of the published data of the population CPC and most of the population CPB were significantly higher than the author's own data (20-40-fold). I suggest that differences in sequencing depth should be considered when comparing the genetic characteristics of different MP populations.

Response: Thank you for this comment.

1.Yes, we agree, the sequencing depth will influence the detection of variants and then influence the following bioinformatic analysis based on these variants. We investigated how the sequencing depth impact the detection rate and accuracy for genome-wide SNPs by investigating the published papers. Current research suggests that a depth of 10X represents an optimal practical target for achieving comprehensive coverage and for the reliable discovery of variants, ensuring that the sequencing covers more than 99% of the genome[21]. This depth not only ensures broad genomic analysis but also maintains a balance between cost and information yield. As illustrated in the Response figure 7, the sequencing coverage increased with the sequencing depth, though not linearly. There was a sharp rise in coverage from 60% to 95% as depth increased from 1X to 4X. After this, coverage increased more gradually from 95% to 99% as depth increased from 5X to 10X, stabilizing at around 99% at a depth of 10X. This level of coverage closely approximates the results obtained at sequencing depths exceeding 10X, where the genome coverage exceeds 99%. Therefore, 10X seems to be an appropriate depth for accurate genotyping and population genetic studies, and may be no impact on the comparison of genetic characteristics?

Response figure 7. Coverage with sequencing depth for each downsampled genome. Whole-genome coverage as a function of sequencing depth for each downsampled genome[21].

2.To further confirm whether the different sequencing depth will influence the analysis in this study, we randomly extract reads from individuals of the three Chinese pangolin populations, and to control that all individuals reached a sequencing coverage of 10X and then re-do the analysis we performed in this study. We finally found that the results we obtained based on the 10X data are very consistent with that we calculated in this manuscript with 10 to 40X data (Response figure 8-10).

Response figure 8. PCA analysis of Malayan pangolin and Chinese pangolin populations based on SG(short-read assembled genome) and LG(long-read HiFi assembled genome) with randomly extracted 10X sequencing reads.

Response figure 9. Phylogenetic tree of Malayan pangolin and Chinese pangolin populations based on SG and LG with randomly extracted 10X sequencing reads.

Response figure 10. Admixture analysis of Malayan pangolin and Chinese pangolin populations based on SG and LG with randomly extracted 10X sequencing reads.

3.We also tested that how the sequencing coverage could influence the detection of ROH across the genome by simulating sequencing data with different sequencing depth. We found that the FROH that could be detected will be very close to the true state. Therefore, we conclude that a minimal sequencing depth of 10X could compare with that of even 40X (Response figure 11).

Response figure 11. The impact of sequencing depth on the detection of ROH across the genome. The inbreeding coefficient (FROH) as the function of different sequencing depths. The legend on the far right showed the real FROH value of the simulated individual genome.

Q31. In the result section (lines 218-221), the authors described that "the FROH of the Taiwan individuals was higher than other individuals in the MP population...... the FROH varies greatly among individuals in the CPA or CPB population". I am expecting relevant discussion on these results, which I have not found.

Response: Thank you to point out this issue. Yes, the Taiwan individual is very special among the Chinese pangolin population. The FROH in the Taiwan individual is also very strange, because this individual presented the highest FROH value but almost all ROH fragments (>99%) were less than 1Mb, which is not consistent with the value calculated by previous study (please refer Q12), and is not a very normal situation in endangered species. We have discussed the Taiwan individual in the revised manuscript in both result and discussion part. Lines 466-476.

Q32. Lines 374 and downstream contents: with regard to the conservation, there is a new publication by Wei et al., 2024 in SCIENCE CHINA Life Sciences. I recommend the authors cite this paper to see if they agree to each other in terms of MP conservation.

Response: Thank you for this reminder. The study of Wei et al., mainly explored the genetic structure and population status of Chinese pangolin populations, including genetic diversity, inbreeding level, gene flow, and mutational load. The study has conducted a comprehensive genomic analysis of 94 individuals across three populations. The research indicates that while historical climate change has played a role in shaping these populations, it is the recent human activities that have precipitously reduced the numbers pangolin populations, leading to increased inbreeding.

We did not include the samples of Wei et al, because: 1) at the outset and during the submission of this study, the aforementioned research had not yet been published, therefore it was not included in the original version for comparison and discussion. 2) Because the data is not synchronous released with the publication of the paper, we were unable to directly compare our results with their study at that time. 3) There is still ongoing debate regarding the population structure of the Chinese pangolin, and we are preparing a manuscript covering much more samples to settle down this controversy. 4) We need to reanalyze the entire article if we now added their samples in the current study, however, this could not influence much about the main conclusions of the manuscript. Therefore, this study did not prepare extensive comparison and discussions with the research of Wei et al, but we have cited this paper and make a brief comparison with their study in the revised manuscript (Line 71). We appreciate of the reviewer’s understanding.

Q33. Line 461: Please provide the accession number of reference genomes.
Response: Thank you. The accession number of reference genomes in this study are GCA_040802235.1 and GCA_040802205.1. And we have added this information in “Data Availability” section.
Q34. Figure S18, (a) should be "…based on the SG".
Response: Thank you for your attention to detail. We have modified the legend of Figure S7e.
Q35. English needs to be double checked to avoid mistakes. For example, in line 29, "with the high inbreeding and ….." , "the" should be deleted; in Line 328, "the two genetic parameters ……were……", "were" should be "are".
Response: Thank you for this comment. We have corrected all the descriptions mentioned above and also had the entire manuscript proofread by the native speaker. Line 30
1. Cheng, H., et al., Haplotype-resolved de novo assembly using phased assembly graphs with hifiasm. Nature methods, 2021. 18(2): p. 170-175.
2. Rhie, A., et al., Merqury: reference-free quality, completeness, and phasing assessment for genome assemblies. Genome biology, 2020. 21: p. 1-27.
3. Li, M., et al., Chromosome-level genome assembly of Aquilaria yunnanensis. Scientific Data, 2024. 11(1): p. 790.
4. Fitz-Gibbon, S., et al., Reference genome of California walnut, Juglans californica, and resemblance with other genomes in the order Fagales. Journal of Heredity, 2023. 114(5): p. 570-579.
5. Flack, N., et al., Chromosome-level, nanopore-only genome and allele-specific DNA methylation of Pallas's cat, Otocolobus manul. NAR Genomics and Bioinformatics, 2023. 5(2): p. lqad033.
6. Kim, H.-s., et al., KOREF_S1: phased, parental trio-binned Korean reference genome using long reads and Hi-C sequencing methods. GigaScience, 2022. 11: p. giac022.
7. Mead, A., et al., The genome assembly of Island Oak (Quercus tomentella), a relictual island tree species. Journal of Heredity, 2024. 115(2): p. 221-229.
8. Wang, Q., et al., Whole-genome resequencing of Chinese pangolins reveals a population structure and provides insights into their conservation. Commun Biol, 2022. 5(1): p. 821.
9. Hu, J.Y., et al., Genomic consequences of population decline in critically endangered pangolins and their demographic histories. Natl Sci Rev, 2020. 7(4): p. 798-814.
10. Formenti, G., et al., The era of reference genomes in conservation genomics. Trends Ecol Evol, 2022. 37(3): p. 197-202.
11. Zhang, L., et al., Chromosome-scale genomes reveal genomic consequences of inbreeding in the South China tiger: A comparative study with the Amur tiger. Mol Ecol Resour, 2022.
12. Yang, S., et al., Genomic investigation of the Chinese alligator reveals wild-extinct genetic diversity and genomic consequences of their continuous decline. Mol Ecol Resour, 2022.
13. Lan, T., et al., Large-scale genome sequencing of giant pandas improves the understanding of population structure and future conservation initiatives. Proceedings of the National Academy of Sciences, 2024. 121(36).
14. Dussex, N., et al., Purging and accumulation of genetic load in conservation. Trends in Ecology & Evolution, 2023. 38(10): p. 961-969.
15. Dussex, N., et al., Purging and accumulation of genetic load in conservation. Trends Ecol Evol, 2023. 38(10): p. 961-969.
16. Ray-Chaudhuri, S., et al., Chromosomes and the karyotype of the pangolin, Manis pentadactyla L.(Pholidota-mammalia). Experientia, 1969. 25: p. 1167-1168.
17. CHEN, Q., et al., Studies on the mitotic chromosomes and meiotic synaptonemal complexes (SC) of Chinese pangolin (Manis pentadactyla). Zoological Research, 1991. 12(3): p. 299-304.
18. Wu, S.-H., et al., Cytogenetic analysis of the Formosan pangolin, Manis pentadactyla pentadactyla (Mammalia: Pholidota). Zoological Studies, 2007. 46(4): p. 389-396.
19. NV, A., Chromosomes and karyotype of the Indian pangolin, Manis crassicaudata Gray (Pholidota-Mammalia). Cytologia, 2000. 65(4): p. 379-382.
20. Cheng, H., et al., Haplotype-resolved assembly of diploid genomes without parental data. Nature Biotechnology, 2022. 40(9): p. 1332-1335.
21. Jiang, Y., et al., Optimal sequencing depth design for whole genome re-sequencing in pigs. Bmc Bioinformatics, 2019. 20: p. 1-12.
