# Supplementary material for: Enhancing inbreeding estimation and global conservation insights through chromosome-level assemblies of the Chinese and Malayan pangolin
Source: Gigascience. 2025 Feb 14;14:giaf003. doi: 10.1093/gigascience/giaf003 (PMC11825179; doi:10.1093/gigascience/giaf003)
Supplement: giaf003_Response_to_Reviewer_Comments_Revision_1 [file giaf003_response_to_reviewer_comments_revision_1.pdf]

Author's Response To Reviewer Comments

Reviewer #2: The authors have engaged positively and comprehensively with reviewer feedback, and I think the manuscript is a lot clearer as a consequence. I only have a few minor outstanding questions/comments.
MINOR COMMENTS
1.I suggest stressing the chromosome-level nature of the assembly in the title.
Response：Thank you. To address your suggestion, we have revised the title of our manuscript to better reflect the chromosome-level nature of our assembly. The new title is:
"Enhancing inbreeding estimation and global conservation insights through chromosome-level assemblies of the Chinese and Malayan pangolin"
2. Abstract - mention the HiC as well as HiFi reads.
Response：We have revised the description of Abstract in Lines 25-26.
3.L97: "all genomes (2 diploid assemblies and 4 haploid assemblies)" - are these not the same thing? Surely, the "diploid" assemblies are just the combined pair of haploid assemblies?
Response：Thank you. When we refer to "2 diploid assemblies and 4 haploid assemblies," we are indicating two different types of genome assemblies. Diploid assemblies are not simply a combined pair of haploid assemblies.
The diploid genome is the genome assembly without genome phasing, is a hybrid genome mixed with the genetic information from both parents, which represents the most common form of genome assembly. The haploid assembly, meticulously constructed with HiFi long reads and Hi-C data, is designed to distinguish the haplotype of each parent[1]. Therefore, the most important process for the assembly of the haploid genome is the phasing process to separate the genome segment from each parent. To make it clearer, I prepared a picture to show this:

Response figure 1. Schematic diagram for the genomic sequence assembly
4.In Table 1, it would be useful to have the total assembly size and sequencing depth. Given the comparison with the short-read genomes, it would be informative to have their stats in the table too, which would really show where the improvements were made.
Response：Thank you for your suggestion to enhance Table 1 with additional statistical information. The current version of the Table 1 has been modified based on the previous reviewer's comments, and the comparison with other genomes has also been displayed in Table S19 following the reviewer's suggestions. We have updated Table 1 to incorporate the total assembly size and sequencing depth.
5.I don't think it's strictly true to say that 10 PacBio HiFi reads ensures a reliable assembly. It could still be false duplications or contamination. 10 reads might also be a bit strict, depending on the depth of coverage. I can see what the authors are trying to do here, but wonder whether a slightly different descriptor could/should be found.
Response：The definition and calculation of the reliable assembly genome regions in the manuscript followed the previously published research[2]. Based on your suggestions, we have modified the calculation method for the reliable blocks. We consider the regions in the genome that are supported by two sequencing technologies (Hi-C and HiFi) as reliable blocks[3]. This method is more stringent, and we believe its reliability will meet the requirements of the analysis. In lines 107 and 109-110.
6.Figure 1. What are the colours in panel B? The details of Panel B are a bit lost. Having a full-width landscape plot with a different colour per chromosome might be clearer.
Response：Thanks for your comments. We have assigned a unique color to each chromosome.

Response figure 2. Introduction to the species distribution and chromosome synteny of the Chinese and Malayan pangolins. (A) The distribution area and sampling sites of the Chinese and Malayan pangolins in this study. The circles represent sampling sites of the Chinese pangolins reported by Wang et al.[4] Samples without detailed locations are not shown on the map. (B) The chromosome-scale synteny analysis between the Malayan pangolin and Chinese pangolin genomes.
7.L145-150. The LG vs SG analysis is much clearer now, but with the ROH analysis I was still left feeling a little unsure of the deeper implications. Unless I have misunderstood, ROH is a relative measure of inbreeding and therefore the absolute changes are not of that much interest. Rather than ROH being bigger with LG vs SG (as expected), is the bigger finding not the apparent reversal in the relative recent

inbreeding of MJ and MP as marked by ROH > 1Mb in LG (MP>MJ) compared to SG (MJ>MP)?
Response：Thank you for this comment. I am sorry that I could not well understand the "relative" you mentioned in the comment. I think the ROH is a direct measurement for the inbreeding, a longer ROH fragment can reflect a more recent inbreeding event, and the time of the inbreeding occurred could be also inferred with the known recombination rate and generation time for a species. The Froh could reflect the general inbreeding for a population, and the Froh for different length of ROHs could help track the inbreeding history of a species. The LG genome could contribute more for the accurately dating back of recent inbreeding history, which we think is the most important thing for the LG genome.
Yes, as you mentioned, one of the findings in this study is the reversal in the recent inbreeding between the MJ (Malayan Pangolin) and MP (Chinese Pangolin) population. This is indeed marked by the presence of ROH segments greater than 1Mb, where in the LG, MJ exhibits a higher frequency of such segments compared to MP (MJ>MP). Conversely, in the SG, MP has a more of these segments (MP>MJ). However, it is difficult to compare inbreeding between different species, because there are too many factors could influence the detection of ROH for different species, such as the different sequencing quality and depth, the different quality of reference genome, the different recombination rate, the different mutation rate, and so forth. Therefore, we did not discuss much about this difference. But we agree with you, this reversal is also an important observation that underscores the superior capability of high-quality reference genomes assembled from long-read sequences in uncovering the real level of inbreeding, when compared to those assembled from short-read sequences. Then, the use of long-read assemblies is crucial to avoid erroneous interpretations, such as the misconception that the Chinese pangolin is more inbred than the Malayan pangolin in the recent past, as might be suggested by findings based on short-read assemblies. By leveraging the detailed insights provided by long-read genomes, we can ensure that our conclusions regarding the inbreeding status of these species are both genuine and reliable. Following your comment, we have included additional discussion on this significant finding. In lines 310-313
8.I found the "Genetic Purging" section a little confusing and hard to follow. L253-4: "which indicated that the genetic purging in the MjavA and MjavB populations was less efficient, but stronger in the MjavA population" - less efficient but stronger than what? Can this section be simplified a little, perhaps with each result introduced in terms of what that particular result is testing and how it related to relative(/absolute?) genetic purging.
Response：Thank you for your comments regarding the "Genetic Purging" section of our manuscript. As your suggestion, we have rephrased the whole paragraph to make it clearer, and we deleted some confused and meaningless analysis from the revised manuscript to make this section simplified. Here we showed the revised content: In the Malayan pangolin populations, the ROHf of highly deleterious mutations (LoF) was significantly lower than the nonROHf in both the MjavA and MjavB population (Fig. 5C). This denoted that many large-effect deleterious alleles (e.g. LoF) still existed but are masked in nonROH regions in both the two populations, indicating that the purging was less efficient to remove recessive deleterious mutations across the whole genome. Further inspection we found the difference between the ROHf and nonROHf was smaller in the MjavA population than that in the MjavB population (Fig. 5D and Supplementary Fig. S11a-S11c), indicating that the higher-level of inbreeding in the MjavA population may have facilitated the exposing of more recessive deleterious alleles to be homozygous, which has resulted in a more efficient purging of deleterious mutations in this population than in the MjavB population [5, 6].
9.Figure 5. Why are C&E and D&F visualised differently? I am not familiar with dot plots (that are not synteny plots) - the legend should explain what the different parts of the plot (lines and bubbles) mean. The boxes and whiskers should also be explained in the box plots. (i.e. what range do they represent.)
Response: Thank you for your attention to the details in our figures.
In our study, Figures 5C and 5E compared the frequency of LoF mutations (LoF/synonymous mutations) in ROH (ROHf) to that of non-ROH (nonROHf) regions within populations. Box plots (Figures 5D and 5F) was set to compare the ratio of ROHf to nonROHf of LoF mutations in the population to further clarify the differences between the LoF frequency in ROH and that in nonROH regions.
To further enhance the clarity and comprehension of this figures, we have expanded upon the legend for Figure 5 in the revised manuscript. This includes a detailed elucidation of the elements within both the dot plots and box plots. In lines 280-285.

Response figure 3. (C) Dot plot showing the occurrence of LoF mutations in the two Malayan pangolin populations calculated as the ratio of the number of the mutational load to synonymous mutations in the ROH regions (ROHf) or nonROH (nonROHf) regions across the genome. Each point signifies the LoF frequency of an individual. The large dots represent the average LoF frequency for the population, while

the lines indicate the standard deviation range around the mean. (D) The ratio of ROHf to nonROHf for the LoF in the two Malayan pangolin populations. The box represents the interquartile range (IQR), stretching from the first quartile (Q1) to the third quartile (Q3). The line that bisects the box indicates the median value. The whiskers extended from the box to show the variability of the data, typically reaching to the minimum and maximum values that fall within 1.5 times of the IQR from Q1 and Q3, respectively.
10.L385: Typo: "readsand"
Response：Thank you for the correction, it has been amended. In line 397
11.L469-470. Why was there the requirement for ancestral alleles to be the "predominant" allele? (This should be "Major" allele, I think.) I can see no reason why an ancestral allele could not be the minor allele.
Response：Yes, you are right here we mean the major allele, and we changed in the manuscript to avoid further misunderstanding. In line 482
Ancestral alleles are those that persist in their initial state, while derived alleles are the result of mutations that have diverged from this initial state. Under the neutral theory, it is common to consider the major allele of population as the ancestral one, especially conserved genomic regions across species[7, 8]. This is because the major allele, is more likely to be the one that has been preserved through evolutionary history, unless there is strong evidence of positive selection favoring a derived allele[9]. Another common approach to identifying ancestral alleles is by looking at the alleles present in closely related species[10]. This method is based on the assumption that mutations occur only once at a given position and that they happened after the species divergence[8]. Alleles that are shared among multiple species are more likely to be ancestral, as they have not diverged from the initial state. This shared state often corresponds to the major allele in the population [11].
While it is known that major alleles are not always ancestral, using major or common alleles as a proxy for ancestral alleles is a practical approach in the absence of a comprehensive list of ancestral alleles. Furthermore, we have integrated additional evidence from closely related species to further validate and refine our identification of ancestral alleles, ensuring greater accuracy in our determination. This inference method is widely used in genomics studies, including research on pangolins[8, 12-14].
12.L524. Why was k=17 selected? This seems very small for a vertebrate. There are only 17.2 billion unique DNA kmers, so the chance of random convergence in a >2Gbp genome is quite high. k=31 is the de facto standard, although k=21 or k=27 are also used quite often.
Response：Thank you for your comment on the choice of k-mer size in our analysis. We have updated our analysis by using k-mer size of 31 and included the results in the Fig S2.
13. L569: Typo: "derive"
Response：Thank you for your reminder. We have corrected it. In lines 578 and 580.

1. Cheng H, Concepcion GT, Feng X, Zhang H and Li H. Haplotype-resolved de novo assembly using phased assembly graphs with hifiasm. Nature methods. 2021;18 2:170-5.
2. Qi W, Lim Y-W, Patrignani A, Schläpfer P, Bratus-Neuenschwander A, Grüter S, et al. The haplotype-resolved chromosome pairs of a heterozygous diploid African cassava cultivar reveal novel pan-genome and allele-specific transcriptome features. GigaScience. 2022;11:giac028.
3. Rhie A, McCarthy SA, Fedrigo O, Damas J, Formenti G, Koren S, et al. Towards complete and error-free genome assemblies of all vertebrate species. Nature. 2021;592 7856:737-46.
4. Wang Q, Lan T, Li H, Sahu SK, Shi M, Zhu Y, et al. Whole-genome resequencing of Chinese pangolins reveals a population structure and provides insights into their conservation. Commun Biol. 2022;5 1:821. doi:10.1038/s42003-022-03757-3.
5. Nicolas D, Tom vdV, Hernán E. M, Christopher W. W, David D-d-M, Johanna vS, et al. Population genomics of the critically endangered kākāpō. Cell Genomics. 2021;1 1:100002. doi:10.1016/j.xgen.2021.100002.
6. Xue Y, Prado-Martinez J, Sudmant PH, Narasimhan V, Ayub Q, Szpak M, et al. Mountain gorilla genomes reveal the impact of long-term population decline and inbreeding. Science. 2015;348 6231:242-5. doi:10.1126/science.aaa3952.
7. Watterson G and Guess HA. Is the most frequent allele the oldest? Theoretical population biology. 1977;11 2:141-60.
8. Sabeti PC, Schaffner SF, Fry B, Lohmueller J, Varilly P, Shamovsky O, et al. Positive natural selection in the human lineage. science. 2006;312 5780:1614-20.
9. Fay JC and Wu C-I. Hitchhiking under positive Darwinian selection. Genetics. 2000;155 3:1405-13.
10. Rocha D, Billerey C, Samson F, Boichard D and Boussaha M. Identification of the putative ancestral allele of bovine single-nucleotide polymorphisms. Journal of Animal Breeding and Genetics. 2014;131

6:483-6.
11. Hacia JG, Fan J-B, Ryder O, Jin L, Edgemon K, Ghandour G, et al. Determination of ancestral alleles for human single-nucleotide polymorphisms using high-density oligonucleotide arrays. Nature genetics. 1999;22 2:164-7.
12. Naji MM, Utsunomiya YT, Sölkner J, Rosen BD and Mészáros G. Investigation of ancestral alleles in the Bovinae subfamily. BMC genomics. 2021;22:1-12.
13. Khan A, Patel K, Shukla H, Viswanathan A, van der Valk T, Borthakur U, et al. Genomic evidence for inbreeding depression and purging of deleterious genetic variation in Indian tigers. Proceedings of the National Academy of Sciences. 2021;118 49:e2023018118.
14. Hu J-Y, Hao Z-Q, Frantz L, Wu S-F, Chen W, Jiang Y-F, et al. Genomic consequences of population decline in critically endangered pangolins and their demographic histories. National Science Review. 2020;7 4:798-814.
