# Supplementary material for: Enhancing inbreeding estimation and global conservation insights through chromosome-level assemblies of the Chinese and Malayan pangolin
Source: Gigascience. 2025 Feb 14;14:giaf003. doi: 10.1093/gigascience/giaf003 (PMC11825179; doi:10.1093/gigascience/giaf003)
Supplement: giaf003_Reviewer_1_Report_Original_Submission — Sergei Kliver -- 7/24/2024 [file giaf003_reviewer_1_report_original_submission.pdf]

Reviewer Report

Title: Enhancing inbreeding estimation and global conservation insights through chromosome-level assemblies of the Chinese and Malayan pangolin

Version: Original Submission **Date:** 7/24/2024

Reviewer name: Sergei Kliver

Reviewer Comments to Author:

The manuscript describes two novel VGP style genome assemblies of Malayan and Chinese pangolins. New assemblies provide a significant improvement to previously published short read references. Sequenced data and software used for assembly are reasonable. In addition to the genome assembly, the authors performed some population analysis using previously published resequencing data. I see no issues with the results of this part of the manuscript, but the discussion (comparison with previously published results) should be enhanced (as a part of review process). Title of the manuscript implies that such a comprehensive comparison is inside the text.
Major comments.
I. As I understood, no parental data was generated for genome assembly, and only HiFi + HiC data were generated from the reference individuals. It means that resulting assemblies are not completely haplotype resolved. Combination of HiFi and HiC allows to (nearly) completely resolve haplotypes only at the level of chromosomes, but chromosomal scaffolds are still mixed between haplotypes. It is a very common mistake to treat such an assembly as haplotype- resolved. I encourage authors to somehow underline it in the text, for example by adding "at chromosomal level" where it is necessary
II. QV scores look relatively low (<50). I would expect something like 55-60+ for such input data.
III. Figure 1C is very difficult to understand. I encourage author to remove it completely or do a linear plots (or set of them) similar to Figure 1D
IV. row 162-163: "All these SVs were validated by our assembled contigs (Supplementary Fig. S9)". Figure is very unclear, and a reader may expect some results to be present on the figure and not the methods. Also I would expect some inversions encompassing whole contigs (especially at the end of chromosomes, where coverage often drops and contigs are shorter). Such inversions might be an artifacts of the HiC scaffolding and are very common. Please confirm that you have found none of them.
V. row 311-316. A bit of a strange wording. "Much better contiguity , which is <big numbers >… longer". Please, simplify. For example by just comparing N50s in the main text
VI. Manuscript lacks a comparison (and even mention of) with chromosome-length assemblies of malayan and Chinese pangolins generated by DNAzoo team (upgrade of two draft assemblies from NCBI). They are available at dnazoo.org. Such a comparison makes more sense than comparing with old drafts.
VII. Paragraphs "Genome-wide extinction risks in pangolin populations" and "Novel implications for the global conservation of these two pangolin species" confuse me a bit. Authors state that they have not generated the resequencing data, but downloaded it from public databases. Dataset set includes data from three publications (references [25, 33, 57]). Majority of it (nearly all Malayan pangolin samples and

>60% of Chinese pangolin) were analyzed in [25]. All Chinese pangolin samples were analyzed in [57]. Authors cite both publications several times, but text lacks a more detailed comparison between the manuscript's results and previously published ones. It is especially crucial and interesting for Chinese pangolin as there is a 100% overlap in samples with [57]. The title of the manuscript requires an extensive comparison with previous publications in all the discussion.
Minor comments:
row 36-44: first paragraph is redundant and can be safely removed. It describes very basic things and facts.
row 60: Pangolin is a prehistoric mammal = "prehistoric" doesn't look like a good term here. Maybe 'living fossil' is better
row 81: remove 'recently introduced'
row 82: "HiFi-specific assembler can generate high-quality and haplotype-resolved de novo assemblies" HiFi data itself (without HiC or parental data) can't produce haplotype-resolved assembly. Please, Correct

Methods

Are the methods appropriate to the aims of the study, are they well described, and are necessary controls included? Choose an item.

Conclusions

Are the conclusions adequately supported by the data shown? Choose an item.

Reporting Standards

Does the manuscript adhere to the journal's guidelines on minimum standards of reporting? Choose an item.

Choose an item.

Statistics

Are you able to assess all statistics in the manuscript, including the appropriateness of statistical tests used? Choose an item.

Quality of Written English

Please indicate the quality of language in the manuscript: Choose an item.

Declaration of Competing Interests

Please complete a declaration of competing interests, considering the following questions:

- Have you in the past five years received reimbursements, fees, funding, or salary from an organisation that may in any way gain or lose financially from the publication of this manuscript, either now or in the future?
- Do you hold any stocks or shares in an organisation that may in any way gain or lose financially from the publication of this manuscript, either now or in the future?
- Do you hold or are you currently applying for any patents relating to the content of the manuscript?
- Have you received reimbursements, fees, funding, or salary from an organization that holds or has applied for patents relating to the content of the manuscript?
- Do you have any other financial competing interests?
- Do you have any non-financial competing interests in relation to this paper?

If you can answer no to all of the above, write 'I declare that I have no competing interests' below. If your reply is yes to any, please give details below.

I declare that I have no competing interests

I agree to the open peer review policy of the journal. I understand that my name will be included on my report to the authors and, if the manuscript is accepted for publication, my named report including any attachments I upload will be posted on the website along with the authors' responses. I agree for my report to be made available under an Open Access Creative Commons CC-BY license (http://creativecommons.org/licenses/by/4.0/). I understand that any comments which I do not wish to be included in my named report can be included as confidential comments to the editors, which will not be published.

Choose an item.

To further support our reviewers, we have joined with Publons, where you can gain additional credit to further highlight your hard work (see: https://publons.com/journal/530/gigascience). On publication of this paper, your review will be automatically added to Publons, you can then choose whether or not to claim your Publons credit. I understand this statement.

Yes Choose an item.
