# Supplementary material for: Enhancing inbreeding estimation and global conservation insights through chromosome-level assemblies of the Chinese and Malayan pangolin
Source: Gigascience. 2025 Feb 14;14:giaf003. doi: 10.1093/gigascience/giaf003 (PMC11825179; doi:10.1093/gigascience/giaf003)
Supplement: giaf003_Reviewer_2_Report_Original_Submission — Richard J Edwards -- 7/30/2024 [file giaf003_reviewer_2_report_original_submission.pdf]

Reviewer Report

Title: Enhancing inbreeding estimation and global conservation insights through chromosome-level assemblies of the Chinese and Malayan pangolin

Version: Original Submission **Date:** 7/30/2024

Reviewer name: Richard J Edwards

Reviewer Comments to Author:

This paper presents two new chromosome-level assemblies for two species of Pangolin, alongside some population genetic data from different populations of each. The quality of the genomes appears to be very high, and these data will indeed "provide valuable genomic resource for future studies on the protection and conservation for pangolins". However, this is Research Article and not a Data Note, and so it also needs a clear narrative relating to the biological insights and which aspects of the data are of most interest. This is currently hampered by numerous language errors, inconsistent formatting, and excessive reference to relatively uninteresting supplementary data. Having some clearer aims/questions beyond a better genome would be helpful in this regard.
If there is no clear message, the authors could consider a data note instead. Either way, I think a lot of the results should be consolidated and presented as Supplementary Results to emphasise the important/interesting data. There is also excessive comparison of results using their long-read and an unknown short-read genome, which should be reduced. (See detailed comments, below.) The manuscript would greatly benefit from professional editing or detailed proof-reading by a native English speaker.
A. MAJOR COMMENTS
A1. For a Research Article, the main narrative is very unclear and the reader has to do far too much work to extract the relevant insights. The abstract hints at a "greater(?) improvement" and "different results in detecting genome-wide extinction risks being compared with short read assembled genome", though also results "were verified again" so are not actually different to previous findings? Except maybe one population (MJ2). This is all vague and confusing. Please clearly identify the key take-home messages from the new data, be explicit about it, and present a unified narrative from Abstract to Conclusions. Is the "relative mild genetic purging in pangolin populations" important? Is the purging itself the new finding, or the fact that it is relatively mild? I found the entire section (L268-294) to be rather confusing with a lack of clarity regarding population-specific versus general conclusions. The narrative would be aided greatly if the subheadings highlighted the key results/points rather than simply breaking the manuscript up into topics.
A2. There are far too many supplementary figures and tables. I recommend trying to consolidate some (eg. S23-S28) into fewer multipanel figures that address the same question. Likewise, many of the tables could be combined and consolidated as the same data is presented for MJ and MP in two table, or tables with the same rows (samples) could be combined into a bigger table. Ideally, some of the descriptive results could be moved from the main manuscript body into a Supplementary Results section along with many of the references to these supplementary data. It would be helpful if the figures could

be more carefully selected and organised/labelled to support the narrative.
A3. There is insufficient information and discussion about the previous pangolin genomes. Most important, genome "SG" does not seem to be defined anywhere. Is this a previously published genome? Or a short-read assembly of new data? There is a bit of discussion about the previous genomes (L306-323) but the comparisons in Tables S31 and S32 are unclear - are the new genomes missing from these tables? (What are they called?) I think this should be the first Results section. As these are phased chromosome-level vertebrate genomes, assembly statistics should be presented in the context of VGP/EBP standards. There should also be data presented for both haplotypes in Table 1.
A4. The SG vs LG comparison is largely a distraction and floods the paper with boring SI. It is no surprise that LG is better for SVs and long ROH. This is a research paper about pangolins, not a methods paper about LR vs SR genomes. Please reduce this to a clearer question: have there been any previous conclusions based on the other genomes that have been contradicted or (in)validated by the new genomes? Please give your new assemblies names that distinguish them from both each other and the previous assemblies - I recommend getting ToLIDs for your samples and naming the assemblies according to VGP conventions.
A5. Do the new genetic data support the previous population groupings? (Was this based on genetic data itself?) To my eye, the genetic data presented in Fig S15 and S16 do not entirely support the existing population definitions. This needs more discussion. Is only a subset of MJ1 truly different to MJ2? Is there a fourth subpopulation of MP including the Taiwan sample?
A6. Is it possible to clarify the "variable" karyotype from ref 47? Is this biological or technical variation in results? Is both species or just Mpen? Do your results clarify the karyotype in this population - the variation is geographical, right? Can you also confirm that the apparent swapping of chromosome arms on Chr4 between the species is not just a scaffolding error. Are these karyotypes consistent in both haplotypes? It would be good to see the contact maps (Fig S1) and synteny plots (Fig 1D) of the haplotypes against each other and have the telomeres marked to verify the orientation of arms where possible. Is Fig S1 both haplotypes? If so, please mark the actual chromosome boundaries and orient the pairs in the same direction. (Is the X pattern a pair of chromosomes inverted relative to each other, or is it interactions between chromosome arms of one haplotype? The parallel MP Chr5 pattern makes me think the former, but I initially assumed it was one haplotype. Where are the X and Y on the dot plots (Fig S7/S8)? Have both been moved into H1? It would also be clearer if Fig S3 used chromosome labels not scaffold numbers. How were the X and Y identified? I think Fig 1C tries to present some of these data too but it is really unclear, being both too small and lacking any scales/legends. Given the questions over karyotype and the risk of scaffolding errors, I would like to see a deeper analysis of the proposed sites of fusion/fission.
B. MINOR COMMENTS
B1. There are a lot of typos. Most of these just impact readability, but some make the message unclear.
B2. I found the use of MJ, MP and CP to be a bit confusing at first. (MP could be Malayan Pangolin vs CP, but it isnt.) Can the MP/CP for the Chinese Pangolin be made consistent? Mjav and Mpen might be clearer. Likewise, it would be good to be consistent with the ordering and colours used for the species and populations when presented in tables and figures.
B3. Page 4: "the most promising strategies for genome assembling" - This is combining long-read and HiC data. Whether ONT or PacBio is better is subject to debate.

B4. L214: " Among the three Chinese pangolin populations, the inbreeding of the CPA and CPB populations were comparable (CPA: 0.18Â±0.036; CPB: 0.17Â±0.039), but much worse than the CPC population (FROH=0.06Â±0.005) (Fig. 3A and 3E, Supplementary Table S26)." Does this relate to population size and genetic diversity? The heterozygosity of the reference genomes looks low from the Merqury kmer plots. Are they representation of the population heterozygosity?
B5. L218: "Noteworthy, the FROH of the Taiwan individual is much higher than all other individuals in the MP population (FROH=0.54) (Fig. 3C)". There is no MP population. There are three. Confusing. Why is this noteworthy?
B6. Fig 3. Please make the colours and ordering match. Fig 3C is not clear. The size mapping is superfluous as it is just the y-axis. Would be better to use symbols/fill to more clearly differentiate the populations/species. It is not clear from this plot why Taiwan is singled out.
B7. L390. "The Chinese pangolin and Malayan pangolin used for genome assembly were wild rescued individuals by the Guangdong Wildlife Rescue Center. " As these are proposed reference genomes, is it possible to proved any photos or metadata to validate the species IDs? What populations are they from?
B8. L410: "Then, the Hi-C sequencing reads are mapped to the primary genomes by the mem algorithm of Burrows-Wheeler Aligner (BWA, v0.7.17) [66, 67], while Hi-C data quality control was conducted by the Juicer[68] (v1.5). The 3d-DNA pipeline (v190716) was finally used to concatenate and review the scaffolds to chromosome-scale genomes[69]. " How were phased assemblies produced if only the primary genome was scaffolded? Why were both haplotypes not scaffolded independently? I am confused how you can comment on haplotype synteny if not. Was manual editing of the scaffolding performed as part of the assembly curation?
B9. L460. What does "SNPs that were missed" mean? No coverage? Filtered?

Methods

Are the methods appropriate to the aims of the study, are they well described, and are necessary controls included? Choose an item.

Conclusions

Are the conclusions adequately supported by the data shown? Choose an item.

Reporting Standards

Does the manuscript adhere to the journal's guidelines on minimum standards of reporting? Choose an item.

Choose an item.

Statistics

Are you able to assess all statistics in the manuscript, including the appropriateness of statistical tests used? Choose an item.

Quality of Written English

Please indicate the quality of language in the manuscript: Choose an item.

Declaration of Competing Interests

Please complete a declaration of competing interests, considering the following questions:

- Have you in the past five years received reimbursements, fees, funding, or salary from an organisation that may in any way gain or lose financially from the publication of this manuscript, either now or in the future?
- Do you hold any stocks or shares in an organisation that may in any way gain or lose financially from the publication of this manuscript, either now or in the future?
- Do you hold or are you currently applying for any patents relating to the content of the manuscript?
- Have you received reimbursements, fees, funding, or salary from an organization that holds or has applied for patents relating to the content of the manuscript?
- Do you have any other financial competing interests?
- Do you have any non-financial competing interests in relation to this paper?

If you can answer no to all of the above, write 'I declare that I have no competing interests' below. If your reply is yes to any, please give details below.

I declare that I have no competing interests

I agree to the open peer review policy of the journal. I understand that my name will be included on my report to the authors and, if the manuscript is accepted for publication, my named report including any attachments I upload will be posted on the website along with the authors' responses. I agree for my report to be made available under an Open Access Creative Commons CC-BY license (http://creativecommons.org/licenses/by/4.0/). I understand that any comments which I do not wish to be included in my named report can be included as confidential comments to the editors, which will not be published.

Choose an item.

To further support our reviewers, we have joined with Publons, where you can gain additional credit to further highlight your hard work (see: https://publons.com/journal/530/gigascience). On publication of this paper, your review will be automatically added to Publons, you can then choose whether or not to claim your Publons credit. I understand this statement.

Yes Choose an item.
