# Supplementary material for: Enhancing inbreeding estimation and global conservation insights through chromosome-level assemblies of the Chinese and Malayan pangolin
Source: Gigascience. 2025 Feb 14;14:giaf003. doi: 10.1093/gigascience/giaf003 (PMC11825179; doi:10.1093/gigascience/giaf003)
Supplement: giaf003_Reviewer_2_Report_Revision_1 — Richard J Edwards -- 11/3/2024 [file giaf003_reviewer_2_report_revision_1.pdf]

Reviewer Report

Title: Enhancing inbreeding estimation and global conservation insights through chromosome-level assemblies of the Chinese and Malayan pangolin

Version: Revision 1 **Date:** 11/3/2024

Reviewer name: Richard J Edwards

Reviewer Comments to Author:

The authors have engaged positively and comprehensively with reviewer feedback, and I think the manuscript is a lot clearer as a consequence. I only have a few minor outstanding questions/comments.
MINOR COMMENTS
1. I suggest stressing the chromosome-level nature of the assembly in the title.
2. Abstract - mention the HiC as well as HiFi reads.
3. L97: "all genomes (2 diploid assemblies and 4 haploid assemblies)" - are these not the same thing? Surely, the "diploid" assemblies are just the combined pair of haploid assemblies?
4. In Table 1, it would be useful to have the total assembly size and sequencing depth. Given the comparison with the short-read genomes, it would be informative to have their stats in the table too, which would really show where the improvements were made.
5. I don't think it's strictly true to say that 10 PacBio HiFi reads ensures a reliable assembly. It could still be false duplications or contamination. 10 reads might also be a bit strict, depending on the depth of coverage. I can see what the authors are trying to do here, but wonder whether a slightly different descriptor could/should be found.
6. Figure 1. What are the colours in panel B? The details of Panel B are a bit lost. Having a full-width landscape plot with a different colour per chromosome might be clearer.
7. L145-150. The LG vs SG analysis is much clearer now, but with the ROH analysis I was still left feeling a little unsure of the deeper implications. Unless I have misunderstood, ROH is a relative measure of inbreeding and therefore the absolute changes are not of that much interest. Rather than ROH being bigger with LG vs SG (as expected), is the bigger finding not the apparent reversal in the relative recent inbreeding of MJ and MP as marked by ROH > 1Mb in LG (MP>MJ) compared to SG (MJ>MP)?
8. I found the "Genetic Purging" section a little confusing and hard to follow. L253-4: "which indicated that the genetic purging in the MjavA and MjavB populations was less efficient, but stronger in the MjavA population" - less efficient but stronger than what? Can this section be simplified a little, perhaps with each result introduced in terms of what that particular result is testing and how it related to relative(/absolute?) genetic purging.
9. Figure 5. Why are C&E and D&F visualised differently? I am not familiar with dot plots (that are not synteny plots) - the legend should explain what the different parts of the plot (lines and bubbles) mean. The boxes and whiskers should also be explained in the box plots. (i.e. what range do they represent.)
10. L385: Typo: "readsand"
11. L469-470. Why was there the requirement for ancestral alleles to be the "predominant" allele? (This

should be "Major" allele, I think.) I can see no reason why an ancestral allele could not be the minor allele.
12. L524. Why was k=17 selected? This seems very small for a vertebrate. There are only 17.2 billion unique DNA kmers, so the chance of random convergence in a >2Gbp genome is quite high. k=31 is the de facto standard, although k=21 or k=27 are also used quite often.
13. L569: Typo: "derive"

Methods

Are the methods appropriate to the aims of the study, are they well described, and are necessary controls included? Choose an item.

Conclusions

Are the conclusions adequately supported by the data shown? Choose an item.

Reporting Standards

Does the manuscript adhere to the journal's guidelines on minimum standards of reporting? Choose an item.

Choose an item.

Statistics

Are you able to assess all statistics in the manuscript, including the appropriateness of statistical tests used? Choose an item.

Quality of Written English

Please indicate the quality of language in the manuscript: Choose an item.

Declaration of Competing Interests

Please complete a declaration of competing interests, considering the following questions:

- Have you in the past five years received reimbursements, fees, funding, or salary from an organisation that may in any way gain or lose financially from the publication of this manuscript, either now or in the future?
- Do you hold any stocks or shares in an organisation that may in any way gain or lose financially from the publication of this manuscript, either now or in the future?
- Do you hold or are you currently applying for any patents relating to the content of the manuscript?

- Have you received reimbursements, fees, funding, or salary from an organization that holds or has applied for patents relating to the content of the manuscript?
- Do you have any other financial competing interests?
- Do you have any non-financial competing interests in relation to this paper?

If you can answer no to all of the above, write 'I declare that I have no competing interests' below. If your reply is yes to any, please give details below.

I declare that I have no competing interests

I agree to the open peer review policy of the journal. I understand that my name will be included on my report to the authors and, if the manuscript is accepted for publication, my named report including any attachments I upload will be posted on the website along with the authors' responses. I agree for my report to be made available under an Open Access Creative Commons CC-BY license (http://creativecommons.org/licenses/by/4.0/). I understand that any comments which I do not wish to be included in my named report can be included as confidential comments to the editors, which will not be published.

Choose an item.

To further support our reviewers, we have joined with Publons, where you can gain additional credit to further highlight your hard work (see: https://publons.com/journal/530/gigascience). On publication of this paper, your review will be automatically added to Publons, you can then choose whether or not to claim your Publons credit. I understand this statement.

Yes Choose an item.
