# Supplementary material for: Enhancing inbreeding estimation and global conservation insights through chromosome-level assemblies of the Chinese and Malayan pangolin
Source: Gigascience. 2025 Feb 14;14:giaf003. doi: 10.1093/gigascience/giaf003 (PMC11825179; doi:10.1093/gigascience/giaf003)
Supplement: giaf003_Reviewer_3_Report_Original_Submission — Guilian Sheng -- 7/31/2024 [file giaf003_reviewer_3_report_original_submission.pdf]

Reviewer Report

Title: Enhancing inbreeding estimation and global conservation insights through chromosome-level assemblies of the Chinese and Malayan pangolin

Version: Original Submission **Date:** 7/31/2024

Reviewer name: Guilian Sheng

Reviewer Comments to Author:

This manuscript by Lan et al. presents high-quality, haplotype-resolved, and chromosome-scale reference genomes for Chinese and Malayan pangolins, providing crucial insights into genetic diversity, inbreeding, and extinction risks. The study highlights moderate inbreeding in most populations, identifies a highly inbred Malayan pangolin population needing targeted conservation, and detects mild genetic purging. These findings contribute valuable genomic resources and new perspectives for pangolin conservation efforts. In general, the manuscript has clear logic, detailed description and comprehensive data analysis. Therefore, I recommend a minor revision before acceptance. There are some minor comments and suggestions as followingï¼š

1. The authors used abbreviation of the Latin names for the Malayan pangolin (MJ) and Chinese pangolin (MP) in the third paragraph. However, they changed the way of abbreviation for the Chinese pangolin populations (CPA, CPB and CPC) later on. It's not a problem, but a little bit confusing for the readers to catch up the logic of describing.
2. Lines 74-75, "The MJ1 population was …… diverged from the MJ1 population"???
3. In Figure 1A, the legend is inconsistent with what have been shown in the figure. I suppose the authors used wrong colors to indicate CPA and CPC populations. Moreover, chromosome numbers, Hic_scaffold numbers in Figure 1C, Figure 1D, Figure S3 and Figure S5 are not in consistency. Please double check chr 7, chr 8, chr 20 and X chromosome in these figures.
4. Usually, the mean depth of sequence data has an impact on the genomics analyses. In this study, the sequencing depths of the samples provided by the authors were basically between 10-20-fold, but depths of the published data of the population CPC and most of the population CPB were significantly higher than the author's own data (20-40-fold). I suggest that differences in sequencing depth should be considered when comparing the genetic characteristics of different MP populations.
5. In the result section (lines 218-221), the authors described that "the FROH of the Taiwan individuals was higher than other individuals in the MP population…… the FROH varies greatly among individuals in the CPA or CPB population". I am expecting relevant discussion on these results, which I have not found.
6. Lines 374 and downstream contents: with regard to the conservation, there is a new publication by Wei et al., 2024 in SCIENCE CHINA Life Sciences. I recommend the authors cite this paper to see if they agree to each other in terms of MP conservation.
7. Line 461: Please provide the accession number of reference genomes.
8. Figure S18, (a) should be "…based on the SG".
9. English needs to be double checked to avoid mistakes. For example, in line 29, "with the high

inbreeding and ….." , "the" should be deleted; in Line 328, "the two genetic parameters ……were……", "were" should be "are".

Methods

Are the methods appropriate to the aims of the study, are they well described, and are necessary controls included? Choose an item.

Conclusions

Are the conclusions adequately supported by the data shown? Choose an item.

Reporting Standards

Does the manuscript adhere to the journal's guidelines on minimum standards of reporting? Choose an item.

Choose an item.

Statistics

Are you able to assess all statistics in the manuscript, including the appropriateness of statistical tests used? Choose an item.

Quality of Written English

Please indicate the quality of language in the manuscript: Choose an item.

Declaration of Competing Interests

Please complete a declaration of competing interests, considering the following questions:

- Have you in the past five years received reimbursements, fees, funding, or salary from an organisation that may in any way gain or lose financially from the publication of this manuscript, either now or in the future?
- Do you hold any stocks or shares in an organisation that may in any way gain or lose financially from the publication of this manuscript, either now or in the future?
- Do you hold or are you currently applying for any patents relating to the content of the manuscript?
- Have you received reimbursements, fees, funding, or salary from an organization that holds or has applied for patents relating to the content of the manuscript?
- Do you have any other financial competing interests?
- Do you have any non-financial competing interests in relation to this paper?

If you can answer no to all of the above, write 'I declare that I have no competing interests' below. If your reply is yes to any, please give details below.

I declare that I have no competing interests.

I agree to the open peer review policy of the journal. I understand that my name will be included on my report to the authors and, if the manuscript is accepted for publication, my named report including any attachments I upload will be posted on the website along with the authors' responses. I agree for my report to be made available under an Open Access Creative Commons CC-BY license (http://creativecommons.org/licenses/by/4.0/). I understand that any comments which I do not wish to be included in my named report can be included as confidential comments to the editors, which will not be published.

Choose an item.

To further support our reviewers, we have joined with Publons, where you can gain additional credit to further highlight your hard work (see: https://publons.com/journal/530/gigascience). On publication of this paper, your review will be automatically added to Publons, you can then choose whether or not to claim your Publons credit. I understand this statement.

Yes Choose an item.
